# Ratchet, swivel, tilt and roll: a complete description of subunit rotation in the ribosome

**DOI:** 10.1093/nar/gkac1211

**Published:** 2022-12-30

**Authors:** Asem Hassan, Sandra Byju, Frederico Campos Freitas, Claude Roc, Nisaa Pender, Kien Nguyen, Evelyn M Kimbrough, Jacob M Mattingly, Ruben L Gonzalez Jr., Ronaldo Junio de Oliveira, Christine M Dunham, Paul C Whitford

**Affiliations:** Department of Physics, Northeastern University, Dana Research Center 111, 360 Huntington Ave, Boston, MA 02115, USA; Center for Theoretical Biological Physics, Northeastern University, 360 Huntington Ave, Boston, MA 02115, USA; Department of Physics, Northeastern University, Dana Research Center 111, 360 Huntington Ave, Boston, MA 02115, USA; Center for Theoretical Biological Physics, Northeastern University, 360 Huntington Ave, Boston, MA 02115, USA; Laboratório de Biofísica Teórica, Departamento de Física, Instituto de Ciências Exatas, Naturais e Educação, Universidade Federal do Triângulo Mineiro, Uberaba, MG 38064-200, Brazil; Department of Physics, Northeastern University, Dana Research Center 111, 360 Huntington Ave, Boston, MA 02115, USA; Department of Physics, Northeastern University, Dana Research Center 111, 360 Huntington Ave, Boston, MA 02115, USA; Theoretical Biology and Biophysics, Los Alamos National Laboratory, Los Alamos, NM 87545, USA; Department of Biochemistry, Emory University, Rollins Research Center 4027, 1510 Clifton Rd NE, Atlanta, GA 30322, USA; Department of Chemistry, Emory University, 1515 Dickey Dr, Atlanta, GA 30322, USA; Department of Biochemistry, Emory University, Rollins Research Center 4027, 1510 Clifton Rd NE, Atlanta, GA 30322, USA; Department of Chemistry, Columbia University, New York, NY 10027, USA; Laboratório de Biofísica Teórica, Departamento de Física, Instituto de Ciências Exatas, Naturais e Educação, Universidade Federal do Triângulo Mineiro, Uberaba, MG 38064-200, Brazil; Department of Biochemistry, Emory University, Rollins Research Center 4027, 1510 Clifton Rd NE, Atlanta, GA 30322, USA; Department of Physics, Northeastern University, Dana Research Center 111, 360 Huntington Ave, Boston, MA 02115, USA; Center for Theoretical Biological Physics, Northeastern University, 360 Huntington Ave, Boston, MA 02115, USA

## Abstract

Protein synthesis by the ribosome requires large-scale rearrangements of the ‘small’ subunit (SSU; ∼1 MDa), including inter- and intra-subunit rotational motions. However, with nearly 2000 structures of ribosomes and ribosomal subunits now publicly available, it is exceedingly difficult to design experiments based on analysis of all known rotation states. To overcome this, we developed an approach where the orientation of each SSU head and body is described in terms of three angular coordinates (rotation, tilt and tilt direction) and a single translation. By considering the entire RCSB PDB database, we describe 1208 fully-assembled ribosome complexes and 334 isolated small subunits, which span >50 species. This reveals aspects of subunit rearrangements that are universal, and others that are organism/domain-specific. For example, we show that tilt-like rearrangements of the SSU body (i.e. ‘rolling’) are pervasive in both prokaryotic and eukaryotic (cytosolic and mitochondrial) ribosomes. As another example, domain orientations associated with frameshifting in bacteria are similar to those found in eukaryotic ribosomes. Together, this study establishes a common foundation with which structural, simulation, single-molecule and biochemical efforts can more precisely interrogate the dynamics of this prototypical molecular machine.

## INTRODUCTION

Many conformational changes in the ribosome are required during protein synthesis. At various stages of function, there are essential small-scale rearrangements, such as movement of a switch loop during translational EF-Tu activation (1), displacement of the 3’-CCA tail of tRNA during peptide bond formation (2) or ribosomal RNA (rRNA) base-flipping and 30S head domain closure during mRNA decoding (3). At a larger scale, the flexibility of tRNA molecules allows them to navigate an intricate series of rearrangements (10–100 Å, each) (4–6) as they are delivered to the ribosome, transition between ribosomal tRNA-binding sites and then dissociate. These steps are also often accompanied by global reorganization events in the ribosome. While such conformational rearrangements are necessary to sustain cellular life, their large scale and complex character pose a significant challenge to identifying the mechanistic properties that govern translation.

**Figure 1.**
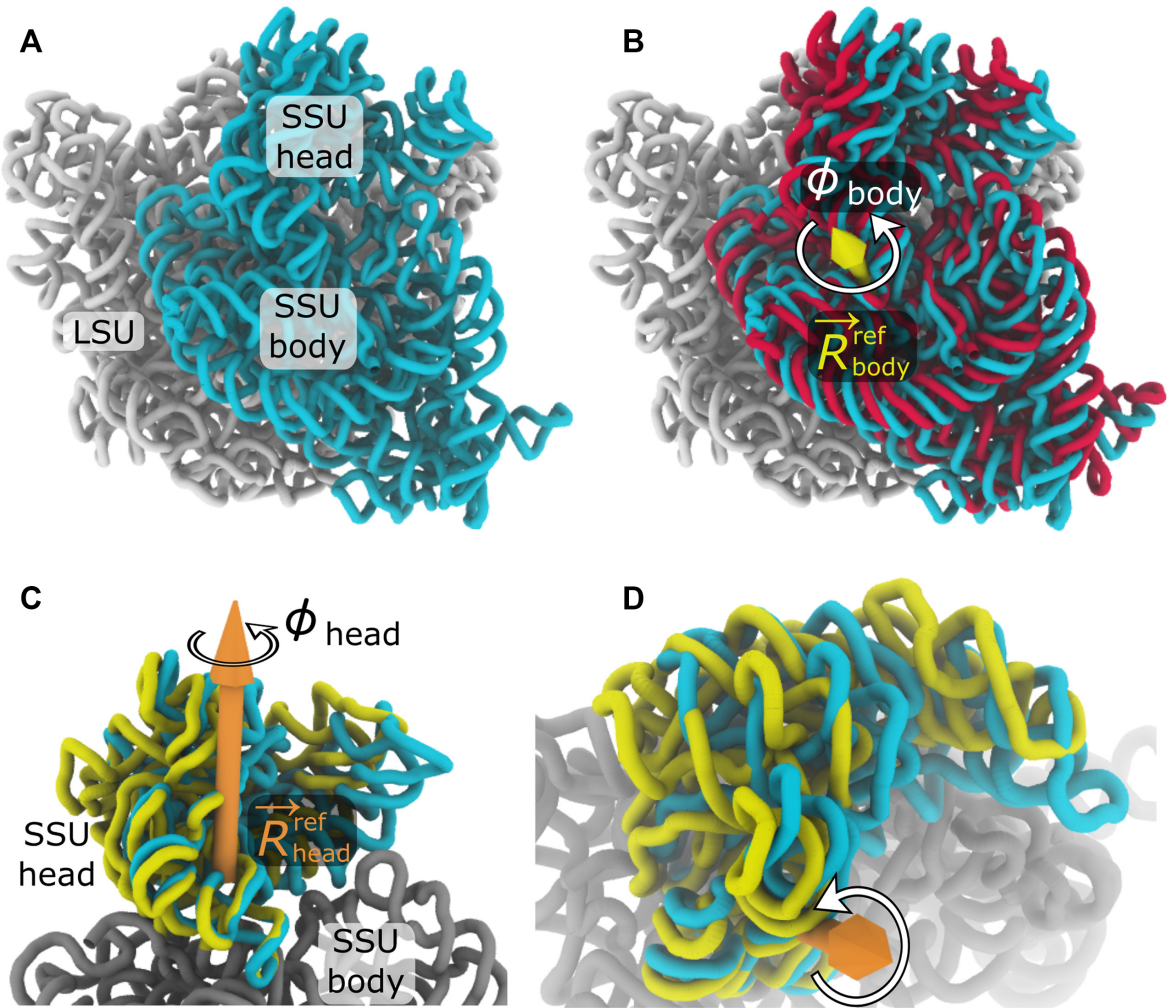
Subunit rotation in the 70S ribosome. (**A**) All ribosomes are composed of two subunits, called the large subunit (LSU; white) and the small subunit (SSU; cyan). rRNA of the bacterial ribosome is shown in a classical unrotated conformation (RCSB ID: 4V9D (32); chains DA, BA). (**B**) During the elongation cycle, the SSU rotates about an axis $\vec{R}^{\rm ref}_{\rm body}$ that is positioned within the SSU body domain. Structures of unrotated (cyan) and rotated (red; RCSB ID: 4V9D (32); chains: CA, AA) SSU rRNAs, after alignment of the LSU rRNA, were used to define the rotation axis (yellow arrow). (**C**) In addition to body rotation, there is also intrasubunit rotation of the SSU head, relative to the SSU body, which is commonly referred to as ‘swiveling.’ Structures with an unrotated head (cyan) and rotated head (yellow; RCSB ID: 4V4Q (33); chains: DB, CA), after alignment of the SSU body (gray), were used to define the rotation axis $\vec{R}^{\rm ref}_{\rm head}$ (orange). (**D**) Rotated perspective of panel C. In the Ribosome Angle Decomposition (RAD) method, ϕ_body_ and ϕ_head_ describe the extent of rotation about the body and head axes, as defined by reference *E. coli* structures. VMD (88) was used to generate all structural representations.

**Figure 2.**
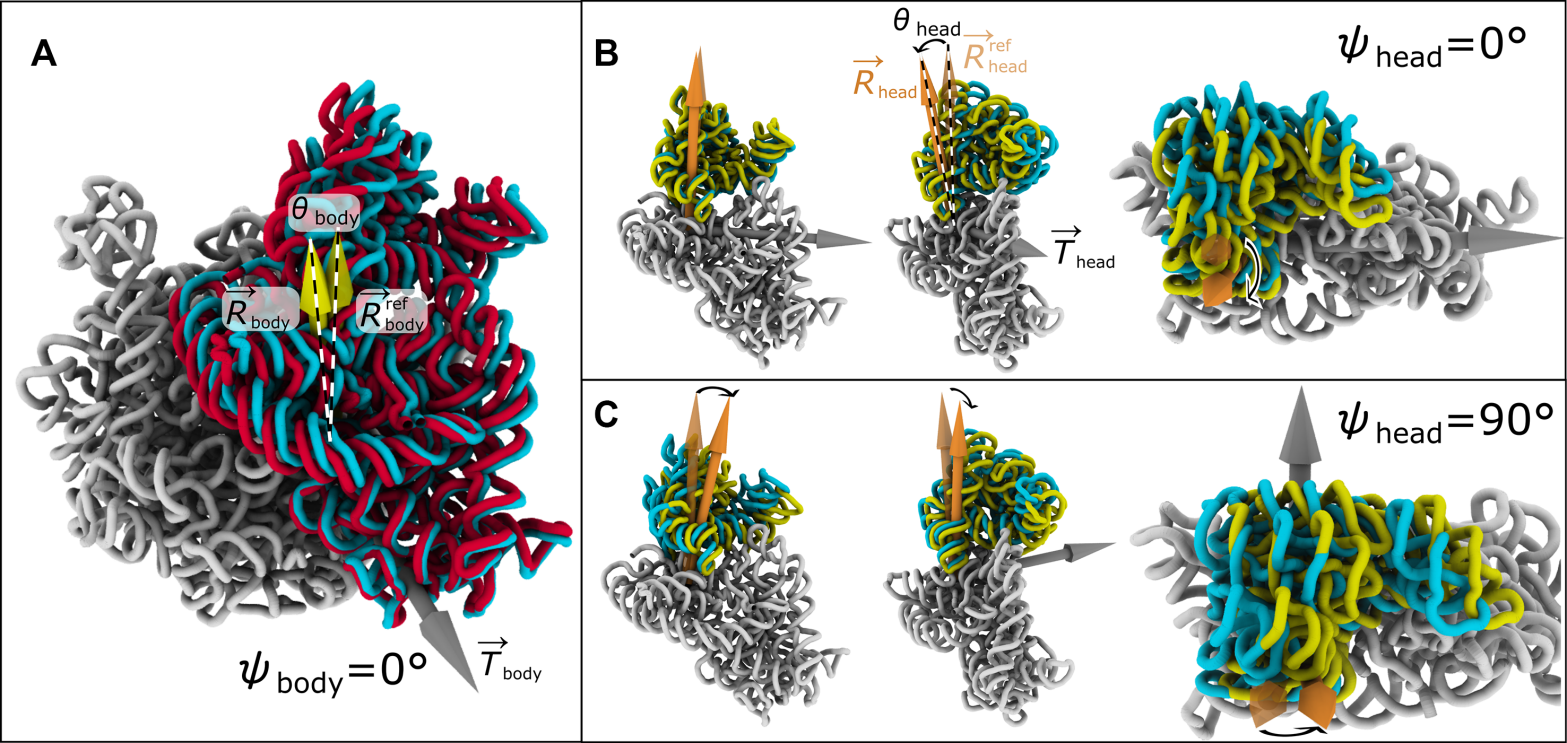
Decomposing subunit rotation, tilt and tilt direction. To fully describe the six orthogonal degrees of freedom of each rigid body (i.e. SSU body, or head), we quantify each orientation in terms of rotation about a fixed axis (ϕ), a tilt-like rotation (θ) about an orthogonal axis (in the direction ψ) and a translation vector ($\Delta \vec{x}$). For the ribosome, this allows us to decompose each SSU head and body orientation in terms of a rotation of magnitude ϕ about an internal rotation axis $\vec{R}$ and a tilt-like rotation of magnitude θ that is about the axis $\vec{T}$. $\vec{T}$ lies in the plane perpendicular to $\vec{R}$, where the direction is given in terms of an angle ψ (the direction of ψ = 0 is arbitrarily defined; See Supplementary Figure S10). (**A**) rRNA of an *E. coli* ribosome in unrotated (cyan; same as Figure 1A) and SSU-tilted (red) orientations. Upon tilting, there is a change in the direction of the body-rotation vector that is fixed to the SSU body (from $\vec{R}^{\rm ref}_{\rm body}$ to $\vec{R}_{\rm body}$). This change in direction of the rotation axis is described in terms of a second rotation about $\vec{T}_{\rm body}$ (i.e. tilt) of magnitude θ_body_. In eukaryotic ribosomes, tilt-like rotations have been described as ‘rolling’. (**B**) Head tilting shown for tilt direction ψ_head_ = 0°, which corresponds roughly to tilting about the mRNA binding track along the A, P and E sites (see Supplementary Figure S10). (**C**) Head tilting shown for ψ_head_ = 90°, which corresponds to the head moving in the direction of the A site. For additional visual comparisons of SSU body and SSU head rotation and tilting, see Supplementary Figures S1 and S2. See Movie S1 for a summary of head rotations.

Over the last 20 years, revolutionary advances in structure determination have allowed for a range of ribosomal subunit orientations to be identified. In early studies, cryogenic electron microscopy (cryo-EM) reconstructions visualized a ratchet-like rotation of the small subunit (SSU; Figure 1B), relative to the large subunit (LSU) (7). More than a decade later, studies of eukaryotic ribosomes (8) showed that the SSU may also undergo tilt-like rotation (i.e. ‘rolling’; Figure 2). Other studies have found that the ‘head’ domain of the SSU (Figure 1C, D) rotates relative to the SSU ‘body’ in prokaryotic and eukaryotic ribosomes, a motion referred to as ‘swiveling’ (9–13). The range of accessible domain motions is further highlighted by simulations of tRNA–mRNA translocation (14) and structures of tmRNA complex (15), where tilt-like rotations of the SSU head are also apparent. To complement structural studies, numerous single-molecule (16–20) and bulk measurements (21–26) have provided insights into the relationship between subunit rotation and tRNA rearrangements during translation. Together, this rapidly growing body of data is demonstrating the various ways that rotary-like rearrangements in the ribosome are integral to protein synthesis.

The complexity of rotational motions in the ribosome has inspired a number of efforts to quantitatively distinguish between rotation states. One strategy is based on Euler–Rodrigues (E–R) angles (11), where the orientation of the SSU head (or body) is described by a single rotation about a calculated axis. In simulation studies, small-scale structural fluctuations have been described by projecting interatomic vectors onto pre-defined planes (27), or by calculating the extent of rotation about a fixed axis (28). When rotation occurs about a static axis, these simulation-inspired methods will be correlated with E–R angles. However, the physical significance of each angular measure becomes ambiguous when subunit rotation occurs about multiple axes. To address this, studies have described subunit orientations by projecting inertial axes onto multiple fixed planes (29,30). To fully separate rotation and tilting, Euler angle decomposition has also been applied to describe all-atom simulations of subunit rotation in a eukaryotic ribosome (31) and tRNA translocation in bacteria (14).

At present, there is a need for an approach that can precisely quantify the similarities and differences between all possible orientations of the SSU head and body domains. While current methods are sufficient to describe subsets of structures that are related (approximately) through one-dimensional rotations (11), domain orientations are multi-dimensional. To address this, Euler angles have been applied to separately describe rotation and tilt of the SSU head (14) and body (31). Euler angles provide a natural coordinate system for quantifying rotation, however in order to fully define the position of an arbitrary rigid body (e.g. the body or head of the SSU), one must account for all three rotational quantities (rotation, tilt and tilt direction) and three translational degrees of freedom (Δ*x*, Δ*y*, Δ$z$). Even though it is well established that the structure of the ribosome can respond to a range of factors (e.g. antibiotic binding, tRNA interactions, EF binding, etc.), a complete description that accounts for all types of rearrangements is needed to systematically partition these effects.

Here, we present an intuitive description of subunit orientations that allows for direct comparison of almost every available structure of the ribosome (1208 LSU–SSU assemblies and 334 isolated SSUs). Our approach, called the Ribosome Angle Decomposition (RAD) method, provides orthogonal measures of rotation, tilt, tilt direction and translational displacement of the SSU body and head domains. Contrary to expectations, this analysis shows that tilting of the SSU body, a movement that was heretofore hypothesized to be unique to eukaryotic ribosomes, is also pervasive in bacterial ribosomes. Specifically, we find that the tilt angle in bacterial ribosomes spans a range of ∼6°, which is the same as has been reported for eukaryotic ribosomes. We also find examples where rotation-only descriptions are insufficient and relatively-large scale translational displacements (>5 Å) of the SSU body and/or head are present. Further, we show that frameshifting-associated structures of bacterial ribosomes exhibit similar domain arrangements as found in eukaryotic ribosomes. This approach allows us to quantitatively classify and compare all types of large-scale tilting rearrangements in the SSU head, from which the most distinct orientations are identified. Finally, we provide a detailed example for how the RAD method may aid the design and assessment of single-molecule FRET experiments. This study establishes a general framework for directly comparing almost any structure of the ribosome, thereby revealing which aspects of subunit motion are universal, and which are organism/domain-specific.

## MATERIALS AND METHODS

### The RAD method

The RAD method is a protocol for describing and comparing the orientations of the SSU body (Figures 1A, B and 2A) and head (Figures 1C, D and 2B, C). As described below, this approach involves the integration of new and existing algorithms. For information on the associated software, see the Data Availability statement.

The method is composed of several steps, which are detailed below. Here, the term ‘model’ is used to refer to any full set of atomic coordinates for which the rotation angles are to be calculated. For example, the coordinates may be defined by an empirically-determined structure (cryo-EM or crystallographic), or they may correspond to configurations obtained from simulation/theoretical methods. To analyze each configuration, a structure alignment step is first applied to identify the ‘cores’ of the LSU, SSU body and SSU head that are structurally similar to the reference *Escherichia coli* structure (RCSB ID: 4V9D (32); chains: DA, BA). Next, a rigid-body approximation is applied, where least-squares alignment of the cores is performed. These rigid-body descriptions are then used to decompose the orientations of the SSU body and head in terms of Euler angles and translations. Three crystallographic models were used to define (i) the reference unrotated/untilted configuration (RCSB ID: 4V9D (32); chains: DA, BA), (ii) the SSU body-rotated (untilted) conformation (RCSB ID: 4V9D (32); chains: CA, AA) and (iii) the SSU head-rotated (untilted) conformation (RCSB ID: 4V4Q (33); chains: DB, CA). Note that two of the reference structures correspond to the asymmetric subunit of RCSB entry 4V9D (32). While these are not the highest resolution structures available (3.0 and 3.5 Å), they were used for continuity with previous analyses (14,27,31). However, as described in the results, there are many alternate structures that exhibit nearly identical domain orientations. Accordingly, other structures could have been used as reference models, while introducing minimal changes in the calculated angles.

### Structure alignment

The first step is to apply a stringent protocol for identifying structurally-conserved elements (i.e. the ‘cores’) within the LSU, SSU body and SSU head. Defining a core is necessary when describing domain orientations in the ribosome (11,27,29,34), since it ensures that more flexible elements, such as the stalks, are not considered when evaluating orientation measures. To automatically identify the SSU head, we perform an initial sequence alignment to *E. coli* using the ClustalW method (35), followed by a contact-based criterion. Next, using the STAMP algorithm (36) (as implemented in the MultiSeq tool (37)), structure alignment is performed between the model and the reference *E. coli* structure, where alignment is performed separately for the rRNA of the LSU, SSU head and SSU body. For all structurally conserved regions, the corresponding *E. coli* rRNA residue numbers are then assigned to the model, while all other residues are excluded from further analysis. While STAMP is not required if the model contains *E. coli* numbering, it is recommended since this will automatically correct potential issues that can be present in the structure file (PDB, or mmCIF), such as non-standard residue numbers, missing residues, misplaced insertion codes, or variations associated with different strains. The deviations between core residues in each model and the reference *E. coli* structure are then pruned, to identify a subset of residues for which the spatial root mean-squared deviation is ∼1 Å. The final pruned set of residues will be referred to as the ‘core’ of each domain (LSU, SSU body or SSU head). For a technical description of all alignment and pruning steps, see Supplementary Methods. An example alignment is shown for a yeast ribosome in Supplementary Figure S3.

In the current study, STAMP alignment was performed with pruning to 1208 different ribosome structures (901 unique RCSB accession codes), as well as 334 isolated SSU structures and 375 isolated LSU structures. After pruning, the RMSD values (mean ± standard deviation) were 1.0, 1.1 and 1.0 (±0.2) Å for the cores of the LSU, SSU body and SSU head (calculated with respect to the reference *E. coli* model). For the LSU–SSU pairs, the mean number of core residues was 1980 (LSU), 789 (SSU body) and 360 (SSU head). All calculated values are given in Appendices A (LSU–SSU assemblies), B (isolated SSUs) and C (isolated LSUs). The small set of structures (*N* = 35) that could not be analyzed with this approach are listed in Appendix D. To further assess the reliability of each calculated angle, experimental validation statistics were evaluated for each set of core residues. Validation statistics are provided for LSU–SSU assemblies (Appendix E), isolated SSUs (Appendix F) and isolated LSUs (Appendix G).

### Rigid-body approximation

After identifying the core residues, we determine approximate rigid-body orientations of the LSU, SSU head and SSU body. To achieve this, the reference *E. coli* structure is aligned to the model via least-squares techniques, which is qualitatively consistent with earlier efforts (11,14,27–29). This alignment step provides a rigid-body orientation that describes the ‘average’ of the structurally-conserved core of each domain. Rigid-body alignment is performed separately for the P atoms of the LSU, SSU body and SSU head. These aligned configurations then serve as rigid-body approximations of each subunit.

### Calculation of rotation, tilt, tilt direction and translation

While the sequence alignment and core identification steps are similar to other protocols, the distinguishing feature of RAD is that Euler angles are used to quantify the orientation of each domain. To this end, the following process is followed separately for the SSU head and body. First, the rotation axis $\vec{R}$ is defined as an internal axis that remains fixed to each rigid body (SSU head or body; Figure 1). The tilt axis $\vec{T}$ is then defined to be perpendicular to the rotation axes of the model and the reference structure ($\vec{R}$ and $\vec{R}^{\rm ref}$; Figure 2). In terms of Euler angle conventions, $\vec{T}$ is parallel to the line of nodes. $\vec{T}$ is defined to intersect $\vec{R}^{\rm ref}$ at the point $\vec{x}_c$ (i.e. the center of rotation and tilt). Since the center of rotation is arbitrary, we define $\vec{x}_c$ as the point that minimizes any residual translational displacement between the model and the reference ($\Delta \vec{x}$). The rotation angle ϕ is then defined as the net rotation about $\vec{R}^{\rm ref}$ (Supplementary Figure S4). The tilt angle θ is defined as the angle formed between $\vec{R}$ and $\vec{R}^{\rm ref}$ (i.e. any rotation that is orthogonal to the primary rotation). The tilt direction ψ is defined as the angle between $\vec{T}$ and an arbitrarily-chosen zero-direction (perpendicular to $\vec{R}$). For the SSU body angles, the LSU rRNA core is defined as the frame of reference. For the SSU head angles, the SSU body rRNA core is the frame of reference. See Supplementary Figure S4 for the precise correspondence between conventional Euler angle definitions and RAD angles. For a comparison with previously-defined rotation measures (11,27–29), see Supporting Results, Supplementary Tables S1–S5 and Supplementary Figures S5–S7.

### Single-molecule fluorescence energy transfer (smFRET) distance calculations

To provide an example for how RAD angles can aid the design and interpretation of smFRET experiments, we compared the calculated angles with multiple intersubunit distances that have been probed in previous smFRET experiments. First, we assessed the approach of Cornish et al. (16), in which protein residues were labeled. For this, we calculated *R*_S6, L9_, defined as the distance between C_α_ atoms in residue 41 of ribosomal protein S6 and residue 11 of ribosomal protein L9. We also calculated *R*_S11, L9_, defined as the distance between C_α_ atoms of residue 11 in L9 and residue 75 in S11. In the study of Marshall et al. (17), subunit orientations were probed by labeling rRNA with fluorophore-labeled DNA oligonucleotides that were complementary to regions of rRNA in the SSU and LSU. To explore this labeling strategy we calculated *R*_h44, H101_, which is defined as the distance between the geometric centers of residues in the turns of 16S rRNA helix 44 (h44, U1450-G1453) and 23S rRNA helix 101 (H101, C2853-U2865). We calculated the distances for all *E. coli* structures that contained these residues and had a resolution of 5Å or better. This included 259 structures for *R*_S6, L9_ and *R*_S11, L9_, and 266 structures for *R*_h44, H101_.

## RESULTS

### Subunit rearrangements involve rotation and linear displacement

**Figure 3.**
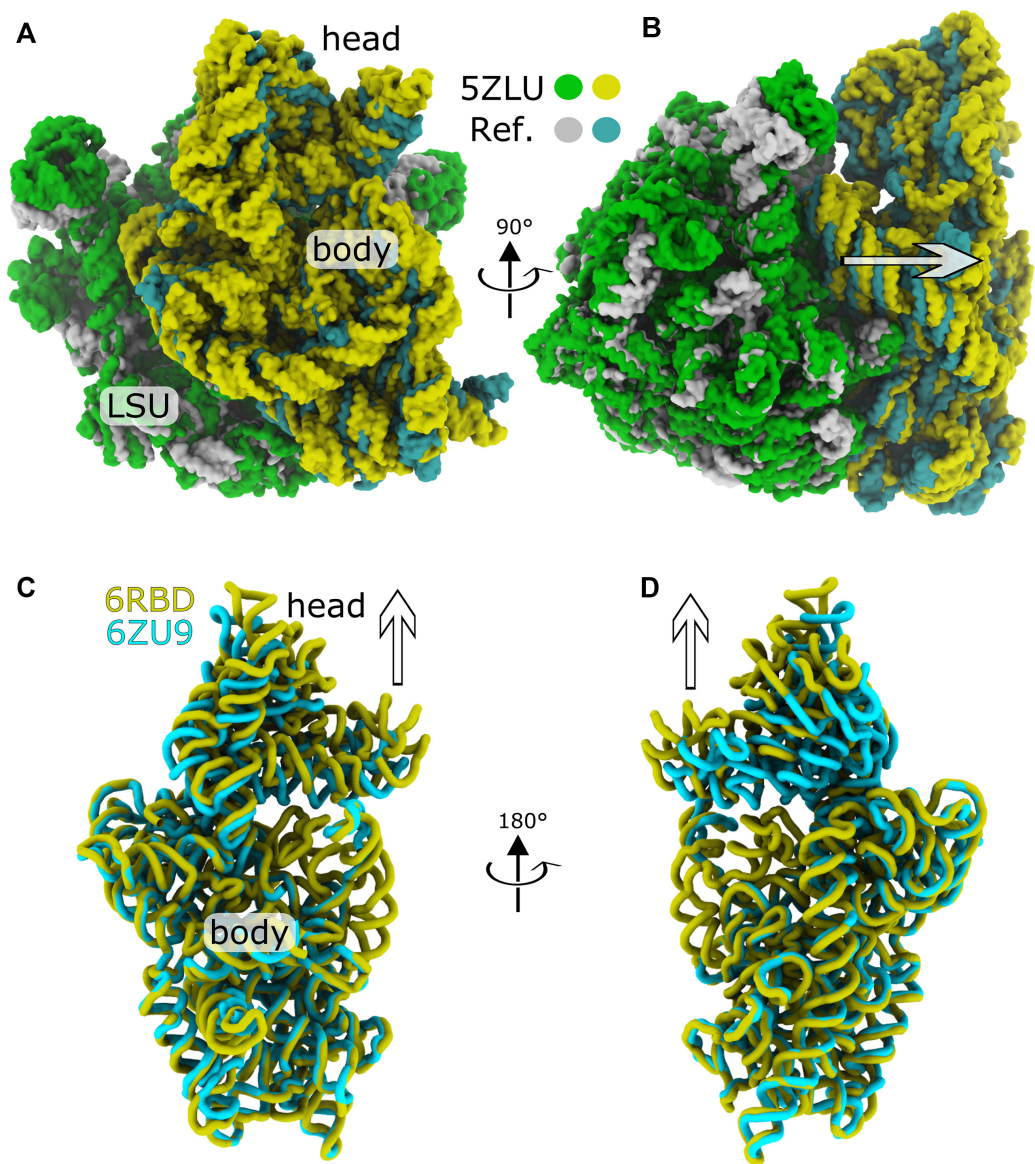
Partitioning rotations and translational displacements of ribosomal subunits. (**A**) The largest-scale translational displacement of the body (5.76 Å) was found in a cryo-EM structure of a *T*.*Thermophilus*ribosome (LSU: green; SSU: yellow; RCSB ID: 5ZLU (39)) with an ABC-F cassette protein, which binds near the E site. (**B**) Same as panel A, rotated by ∼90°. When aligned to the reference *E. coli* structure (LSU:white; SSU:cyan), the SSU is visibly displaced away (direction of the arrow) from the LSU. (**C**) For the SSU head, the largest translational displacement is found in a late assembly intermediate in a yeast ribosome (yellow; RCSB ID: 6RBD (46)), where the head is visibly extended approximately 10 Å, relative to its position in initiation complex (cyan; RCSB ID: 6ZU9 (49)). Perspective similar to panel A. (**D**) Same as panel C, rotated by ∼180°.

To compare the structures of ribosomes from across the kingdoms of life, we developed an approach that can uniquely and unambiguously describe the orientations of the ribosomal small subunit body and head. We used this strategy, called RAD, to align and map nearly every published prokaryotic and eukaryotic (cytosolic and mitochondrial) ribosome and pre-ribosome structure (1208 LSU–SSU pairs, 334 isolated SSUs and 375 isolated LSUs). In the RAD method, the orientations of the SSU body (relative to the LSU) and head (relative to the body) are described in terms of the following rigid-body rotations and translations (Movie S1):

- ϕ_body_ – primary (ratchet-like) rotation of the body (Figure 1B and Supplementary Figure S1A, B)
- θ_body_ – secondary rotation of the body (i.e. tilt/roll) in the direction ψ_body_ (Figure 2A and Supplementary Figure S1C, D)
-
$\Delta \vec{x}_{\rm body}$ – linear translational displacement of the body (e.g. Figure 3B)
- ϕ_head_ – primary (swivel-like) rotation of the head (Figure 1C, D and Supplementary Figure S2A, B)
- θ_head_ – secondary rotation of the head (i.e. tilt) in the direction ψ_head_ (Figure 2B, C and Supplementary Figure S2C–F)
-
$\Delta \vec{x}_{\rm head}$ – translational displacement of the head (e.g. Figure 3C)

Together, these coordinates describe all six rotational and translational degrees of freedom for each domain (body, or head), where the origin corresponds to a crystallographic structure of a classical/unrotated *E. coli* ribosome (RCSB ID: 4V9D (32); chains: DA, BA). The rotation axis $\vec{R}^{\rm ref}_{\rm body}$ (Figure 1B) is the axis associated with rotation between the reference classical structure (i.e. the origin) and a crystallographic model in which the body is rotated (RCSB ID: 4V9D (32); chains: CA, AA). For body orientations, the structures are first aligned based on the LSU rRNA. SSU body tilting is defined as a secondary rotation (θ_body_) that is orthogonal to the primary rotation axis (Figure 2 and Supplementary Figure S1). In accordance with standard Euler angle conventions, (38) the body tilt axis is perpendicular to the rotation axis and it forms an angle ψ_body_ with a pre-defined zero direction. For ease of interpretation, ψ_body_ = 0 is defined to be roughly in the direction of the 16S rRNA helix h44 (Figure 2A). It is important to note that, since the major axis of h44 and the body rotation axis are not perpendicular (Supplementary Figure S8), the zero tilt direction can not be defined to be parallel to h44. To complete our decomposition of the body orientation, any displacement that cannot be accounted for by rotation is then described by a single linear translation $\Delta \vec{x}_{\rm body}$. Consistent with our description of the body, the head rotation axis $\vec{R}^{\rm ref}_{\rm head}$ (Figure 1C, D) corresponds to the axis of rotation between the classical reference model and a reference crystallographic model in which the head is rotated (RCSB ID: 4V4Q (33); chains: DB, CA). Coordinates for the head (ϕ_head_, θ_head_, ψ_head_ and $\Delta \vec{x}_{\rm head}$) were defined analogously to the body coordinates. ψ_head_ = 0 corresponds to tilting of the head away from the LSU, about an axis that is roughly parallel to the mRNA binding track along the aminoacyl (A), peptidyl (P) and exit (E) tRNA binding sites (Figure 2B).

While the ribosome field commonly describes SSU subunit orientations in terms of rigid-body rotations, we identify recently-published examples where the SSU body orientation cannot be related to *E. coli* purely through domain rotations. To quantify this, we considered the scale of the translation for each body ($|\Delta \vec{x}_{\rm body}|$). If the orientation of the body can be related to the classical *E. coli* orientation through rotation and tilt, alone, then $|\Delta \vec{x}_{\rm body}|=0$. As expected from earlier analyses (11), many SSU body orientations are described well in terms of pure rotations ($|\Delta \vec{x}_{\rm body}|\leq 3$ Å for 1158 of 1208 structures). However, there are some notable exceptions, including three structures for which $|\Delta \vec{x}_{\rm body}|$ is greater than 5 Å. The largest body translation value is found in a recent structure of a *T. Thermophilus* ribosome with an ATP-binding cassette F protein bound (RCSB ID: 5ZLU (39); $|\Delta \vec{x}_{\rm body}|=5.76$ Å). In the associated manuscript, the authors noted significant rearrangements in the SSU head and peptidyl transferase center (PTC). Here, we show that, in this structural model, these rearrangements are accompanied by displacement of the SSU body away from the LSU, which may be attributed to a slight expansion (3 Å increase in the radius of gyration) of the LSU rRNA (Figure 3A, B). Since this apparent expansion was found to be uncommon, we asked whether it was a feature of the EM density, or if it arose from possible structural modeling issues. To quantify the level of agreement between each structural model and the associated EM density, we calculated the average atom inclusion value (obtained from the wwPDB validation report) for the core residues used in RAD angle calculations (Appendices E, F and G). For this structure, the core residues generally agree with the EM map, where the average inclusion values are 0.94 (LSU), 0.95 (SSU body) and 0.93 (SSU head). These values suggest the structural model properly reflects the EM data. However, interestingly, small numbers of residues were identified for each core, which indicates the presence of internal deformations of the subunits. Multiple structures of *Enterococcus faecalis* ribosomes also exhibit large body translations ($|\Delta \vec{x}_{\rm body}|=4.15-5.27$ Å; RCSB IDs: 6O8X, 6O8Y, 6O8Z, 6O90) (40), where the body is again displaced away from the LSU. Similarly, a structure of the dormant microsporidium *Vairimorpha necatrix* (RCSB ID: 6RM3 (41); $|\Delta \vec{x}_{\rm body}|=5.01$ Å) also exhibits displacement of the SSU body away from the LSU.

For the vast majority of fully-assembled ribosomes, the SSU head orientations can be related to *E. coli* through simple rotations with small translations ($|\Delta \vec{x}_{\rm head}|\leq3$ Å for 1173 structures). Four of the highest values of $|\Delta \vec{x}_{\rm head}|$ (5.38–6.98 Å) were obtained from time-resolved cryo-EM measurements of a ribosome under conditions that favor retro-translocation (RCSB IDs: 4V70, 4V73, 4V76, 4V79) (28). However, the reconstructions (42) were of limited resolution (15−17 Å), and the average EM inclusion values for the cores were modest (Appendix E). In subsequent structures of related translocation intermediates (43), the translational displacements were much smaller ($|\Delta \vec{x}_{\rm head}|=0.9-1.3$ Å). Accordingly, the larger values are likely due to artificial translations that can arise from the application of flexible-fitting methods to low-resolution EM maps. Interestingly, some higher-resolution structures (2.4–3.7 Å) also exhibit significant head translations, including ribosomes from *Enterococcus faecalis* (RCSB ID: 6O8Z (40); $|\Delta \vec{x}_{\rm head}|= 4.12$ Å) and *Acinetobacter baumannii* (RCSB ID: 7M4X (44), 7RYF (45), 7RYG, 7RYH; $|\Delta \vec{x}_{\rm head}|= 4-5$ Å).

The largest translations of the head are found in assembly intermediates of isolated SSUs. For example, $|\Delta \vec{x}_{\rm head}|>10$ Å for three eukaryotic assembly intermediates in which the head is extended along the direction of the rotation axis (RCSB ID: 6RBD (46), 6Y7C (47), 6G4W (48)). For yeast, comparison of the SSU rRNA from a late assembly intermediate (RCSB ID: 6RBD (46); $|\Delta \vec{x}_{\rm head}|=11.11$ Å) and initiation complex (RCSB ID: 6ZU9 (49); $|\Delta \vec{x}_{\rm head}|=1.09$ Å) illustrates how the head is visibly extended prior to adopting its active structure (Figure 3C, D).

Overall, this initial analysis confirms the expectation that most subunit orientations in fully-assembled ribosomes may be related through rotations (i.e. rotation about a primary *E. coli*-defined axis, followed by tilt) and minimal linear displacements. However, there are clear exceptions where a rotation-only description is insufficient, in particular for late-assembly intermediates in eukaryotic ribosomes. Accordingly, when describing different stages of function, or ribosomes from different organisms, accounting for the full range of motion requires a consistent treatment of all possible head and body displacements.

### Distribution of orientations reveals energetic signatures

**Figure 4.**
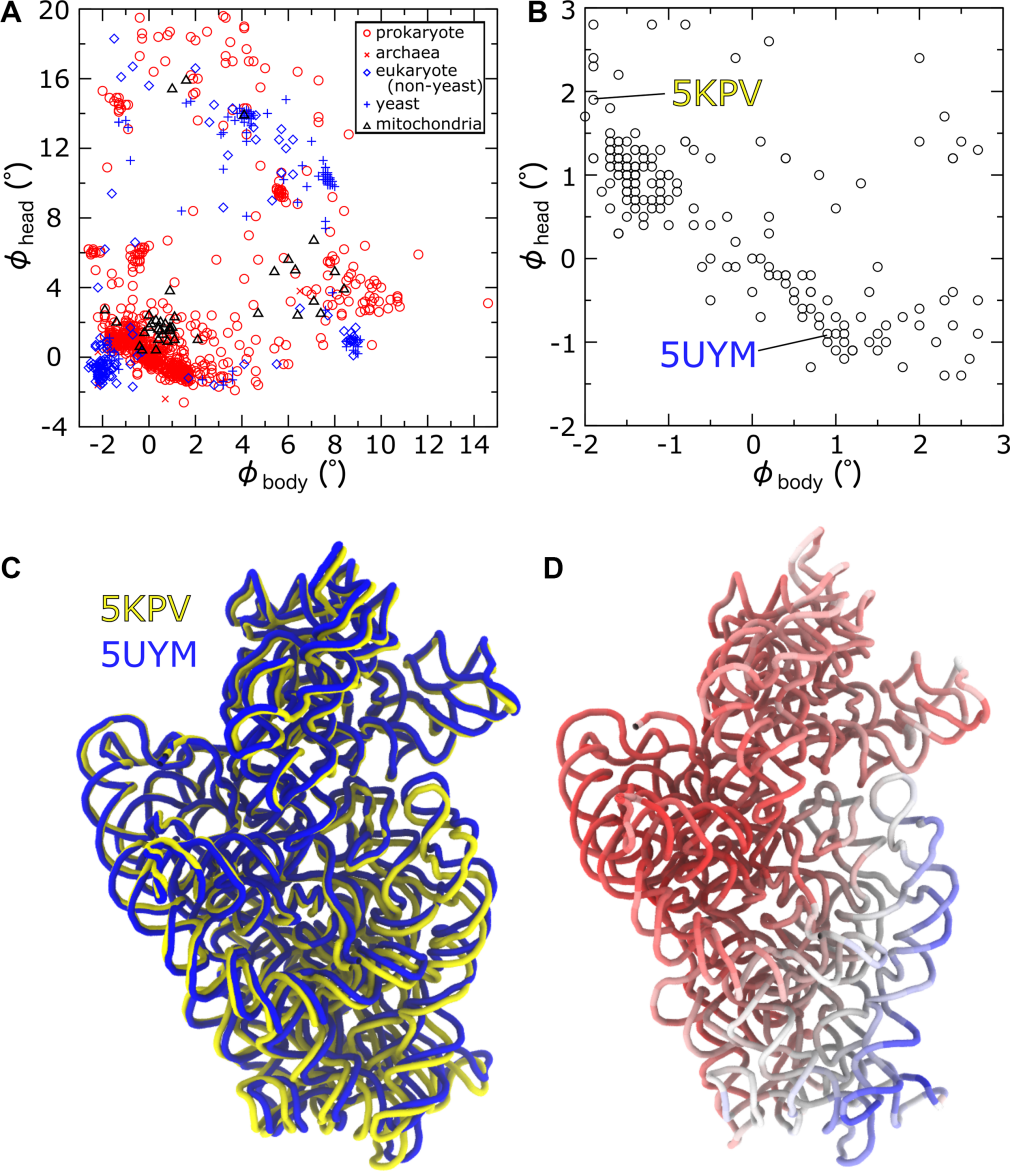
The full range of resolved body and head rotation states. (**A**) SSU body and head rotation angles (ϕ_body_ and ϕ_head_) shown for 1208 different ribosome structures. Values are separately shown for bacteria, archaea, mitoribosomes, yeast and all other cytosolic eukaryotic ribosomes. Rather than being isolated to a few highly-populated regions, there appears to be a quasi-continuous distribution represented by previously-resolved structures. (**B**) Zoomed-in view of panel A, shown only for *E. coli*. Over this range of small values of ϕ_body_ and ϕ_head_, all structures may be qualitatively described as ‘unrotated.’ However, body rotation (ϕ_body_) and head rotation (ϕ_head_) display a clear anticorrelation. (**C**) Structures of two *E. coli* SSUs, after alignment based on the cores of the LSU. For these structures there is a difference of 3° of both ϕ_body_ and ϕ_head_, though the head domains are nearly superimposed. This is the result of a relative displacement of the SSU body, about the head, where both angles must change in an anticorrelated manner. (**D**) Structure of the *E. coli* ribosome colored by the atomic displacements shown in Panel C (red to blue), which confirms the relative mobility of the body, relative to the head.

While it is common to classify different stages of rotation in terms of well-defined states, we find that the set of published structures represents a nearly continuous distribution of body and head rotation angles (Figure 4A). In the 918 bacterial ribosome structures analyzed, the body rotation angle spans 17° (ϕ_body_ ranging from -2.6° to 14.6°), where the smallest value is found for *E. coli* structures in the presence of antibiotics (RCSB ID: 4V52 (50), chain 0) or recycling factor RRF (RCSB ID: 4V54 (50), chain 0). The two largest values (14.6° and 11.6°) are for cryo-EM models (RCSB ID: 4V74, 4V73) (28) obtained under conditions that favor reverse translocation, while more recent structures with tRNA molecules in hybrid/chimeric configurations (RCSB IDs: 7ST7, 7PJU, 7PJV, 7PJX,7PJW) (43,51) exhibit rotation angles that are nearly as large (10.7–10.8°). For structures of LSU–SSU assemblies, the head angle spans a range of 24.8° (-4.7° to 20.1°). There is also a large range of head rotation angles in structures of isolated bacterial SSUs (from –2.5° to 20.2°), where the maximal value is obtained from a late intermediate during assembly (RCSB ID: 3J2H (52)). To quantify the similarities of different structures, we considered the nearest neighbor of each, as described by the rotation angles ϕ_body_ and ϕ_head_. Specifically, after ordering all structures by rotation angle, we determined the difference in angle between sequential structures. For the SSU head of bacterial ribosomes, there were only two sequential pairs that differed by >0.9° (RCSB ID: 6V3B (53) at ϕ_head_ = 11° with RCSB ID: 4V89 (54) at ϕ_head_ = 12.8°; RCSB ID: 7ASP at ϕ_head_ = −4.7° with RCSB ID: 4V4W (55) at ϕ_head_ = −2.6°). Similarly, when considering the body rotation angles, there were only two that differed by >0.5° (RCSB ID: 4V73 (56) at ϕ_body_ = 11.6° with RCSB ID: 7PJW (43) at ϕ_body_ = 10.8° or RCSB ID: 4V74 (56) at ϕ_body_ = 14.6°).

The nearly-continuous coverage of subunit rotation values suggests the energy landscape that governs rotation is likely composed of broad free-energy minima. If the ribosome were to possess deep energetic minima, then one would expect distributions that are highly populated around a few well-defined regions. In contrast to this, the nearly continuous range of rotation angles suggests that modest energetic factors (e.g. minor changes in buffer and temperature) are sufficient to shift the landscape, resulting in small-scale rotational rearrangements. With this signature in mind, one may expect rotational dynamics to be well described in terms of diffusion across a relatively smooth energy landscape that contains broad basins of attraction. This perspective is consistent with theoretical approaches for characterizing biomolecular folding (57,58), as well as ribosome assembly (59), and it has also served as a motivation for applying simplified models to simulate subunit rotation (31,60). In addition, this implied character of the landscape is consistent with studies of the ribosome using coarse-grained models (61–64) and explicit-solvent simulations (65), which have predicted that rotational motions correspond to low-energy deformations. This energetic picture has also been assumed when interpreting the dynamics in other explicit-solvent simulations (27).

To illustrate how energetic signatures can manifest in the form of structural trends, we will consider a subset of *E. coli* structures that have small body and head rotation angles (Figure 4B). These structures would generally be categorized as ‘unrotated.’ Surprisingly, the head rotation angle (ϕ_head_) and body rotation angle (ϕ_body_) appear to be anticorrelated within this ensemble. Inspection of representative structures reveals that this relationship may arise from the mobility of the body, relative to the head. That is, while the head maintains a position that is static (Figure 4C, D), with respect to the LSU, the body orientation is found to vary. This relationship suggests interactions between the LSU and SSU head are stronger (i.e. are energetically more ‘stiff’) than those associated with the SSU body.

### Body tilting is comparable in eukaryotic and prokaryotic ribosomes

**Figure 5.**
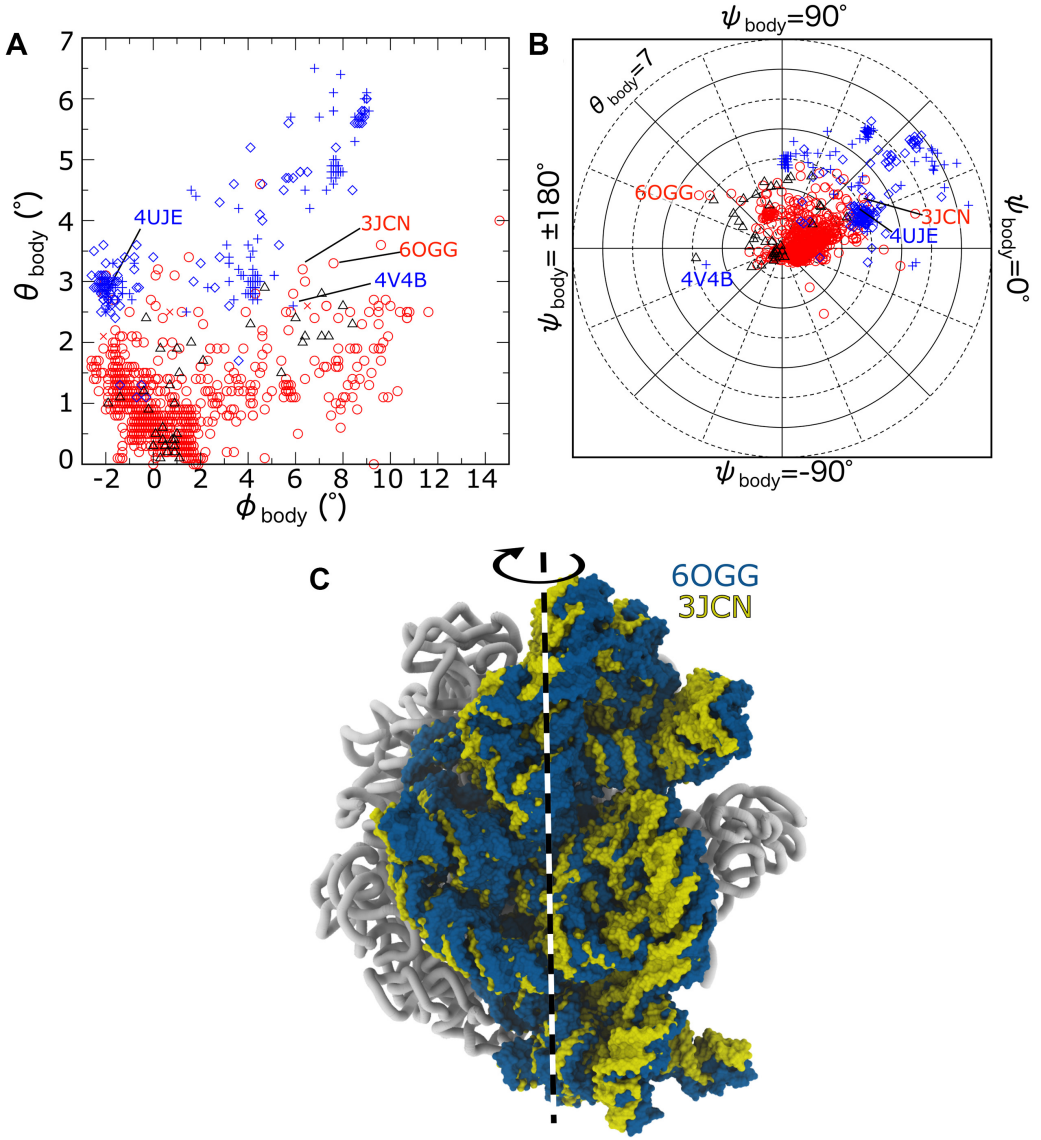
A balance between body rotation and tilt/roll is common to eukaryotic and prokaryotic ribosomes. (**A**) Body tilt/roll angle θ_body_ versus body rotation angle ϕ_body_ for all 1208 ribosome structures (symbols as in Figure 4A). While the largest tilt/roll angles (6.5°) are found in eukaryotic ribosomes (blue), tilt values for prokaryotic ribosomes (red) reach values that are nearly as large (4.5°). Interestingly, only 1/3 of the *E. coli* structures exhibit minimal tilting (θ_body_ < 1°). (**B**) Body tilt angle θ_body_ and tilt direction ψ_body_ shown in polar representation. ψ_body_ = 0 corresponds roughly to rotation about the long axis of H44 (Figure 2A). For most large values of the tilt/roll angle (>5°), the direction of tilting ranges from ψ_body_ ∼15−60°. (**C**) Body tilting/rolling is visible in prokaryotic ribosomes, as illustrated by structures of initiation (RCSB ID: 3JCN (66)) and termination complex (RCSB ID: 6OGG (67)).

We next compared the scale and distribution of body tilting in different organisms (Figure 5). Tilt-like movement of the SSU was first noted in the context of translation in eukaryotic ribosomes (8), where this type of rearrangement was referred to as subunit ‘rolling’. In that study, rolling was described as a ‘secondary rotation’ that is ‘roughly orthogonal to the well-known intersubunit rotation.’ Consistent with that qualitative designation, in our approach the primary rotation (ϕ_body_) corresponds to that of *E. coli*, while any secondary rotation (θ_body_) is orthogonal, by construction.

While rolling was presented as a unique feature of eukaryotic translation, the reported model has a body tilt angle (θ_body_) of 2.8° (RCSB ID: 4UJE (8)), which is similar to various bacterial ribosome structures (Figure 5A). In most structures, the body is tilted over a range of directions: 0 < ψ_body_ < 90° (Figure 5B). For reference, ψ_body_ = 0 corresponds to a tilt axis that is generally in the direction of the long axis of h44 (Figure 2A). As expected, all tilt directions (ψ_body_) are found for small values of θ_body_. However, structures with highly-tilted bodies are more narrowly distributed in the range ψ_body_ = 15–60°, including that of Ref. 8. This represents a relatively narrow range of tilting/rolling directions, though there is a clear spectrum of tilt values.

Our analysis provides quantitative evidence that SSU tilting/rolling motions are significant in prokaryotic ribosomes. To describe tilting/rolling differences, we defined δθ_body_ as the angle formed by the primary body rotation axes ($\vec{R}_{\rm body}$) of any two structures. Since body rotation is described as rotation about $\vec{R}_{\rm body}$, which is defined to be internal to each structure, δθ_body_ is a measure of tilt difference that is independent of the primary rotation (i.e. SSU ratchet-like rotation). If the tilt direction ψ_body_ of two structures is the same, then δθ_body_ is the difference between the corresponding tilt values. However, if the tilt directions differ, then δθ_body_ will be greater than zero, irrespective of the values of θ_body_. As a note, the only difference between θ_body_ and δθ_body_ is that the former is calculated with respect to a reference/classical structure, while the latter is obtained from the pairwise comparison of any two ribosome structures. When considering bacterial ribosomes, the largest 17 values of δθ_body_ (5.5–7.3°) were obtained for models derived from time-resolved cryo-EM measurements of the ribosome under conditions that favor reverse translocation (28,42). The next largest value of δθ_body_ was also 5.5°, which describes differences between *E. coli* structures associated with initiation (RCSB ID: 3JCN (66)) and termination (RCSB ID: 6OGG (67)) factors (Figure 5C). The significant difference in body tilt during initiation and termination highlights how rolling-like motions can facilitate translation in bacterial ribosomes.

We find that body rolling/tilting appears to be of a comparable scale in eukaryotic and bacterial ribosomes. For structures of cytosolic eukaryotic ribosomes, the largest body tilt difference was between cryo-EM structures of a vacant ribosome (RCSB ID: 4V4B (68)) and a ribosome in complex with an mRNA containing an internal ribosome entry site (IRES; RCSB ID: 5JUO (69); δθ_body_ = 9.0°). Interestingly, the tilted orientation in the vacant yeast structure is an outlier amongst cytosolic eukaryotic ribosomes (labeled 4V4B in Figure 5B), where this single model is associated with the 85 largest values of δθ_body_ (6.5–9.0°). The initial manuscript to report rolling (8) also used this structure as a reference. When considering the same two models (RCSB IDs: 4V4B (68) and 4UJE (8)), our approach indicates a tilt difference of 5.4°, which is compatible with the rolling angle that was reported (∼6°). However, one should be cautious when interpreting the precise angle derived from 4V4B. This model predated the availability of effective flexible-fitting techniques (e.g. MDFF (70)), which necessitated the use of rigid-body docking procedures, and the resolution was rather low (11.7 Å). As a result of these factors, the agreement between the structural model and EM density is modest (average core residue inclusion factors of 0.74 for the LSU and 0.75 for the SSU body. Appendix E).

To account for resolution-associated ambiguities in domain orientations, we repeated our analysis of body tilting/rolling for all structures that are at a resolution of 5 Å or better. For these higher-resolution structures, we find the maximum value of δθ_body_ is 5.5° for bacterial ribosomes (RCSB IDs: 3JCN (66) and 6OGG (67)), 5.2° for mitoribosomes (RCSB IDs: 6NU3 (71) and 7QIZ (72)) and 6.4° for cytosolic eukaryotic ribosomes (RCSB IDs: 5DGF and 6XIR (73)). Accordingly, the presented analysis demonstrates that the scale of body tilting/rolling is comparable in currently available structures of prokaryotic and eukaryotic ribosomes.

### The ribosome uses various modes of head tilting during translation

**Figure 6.**
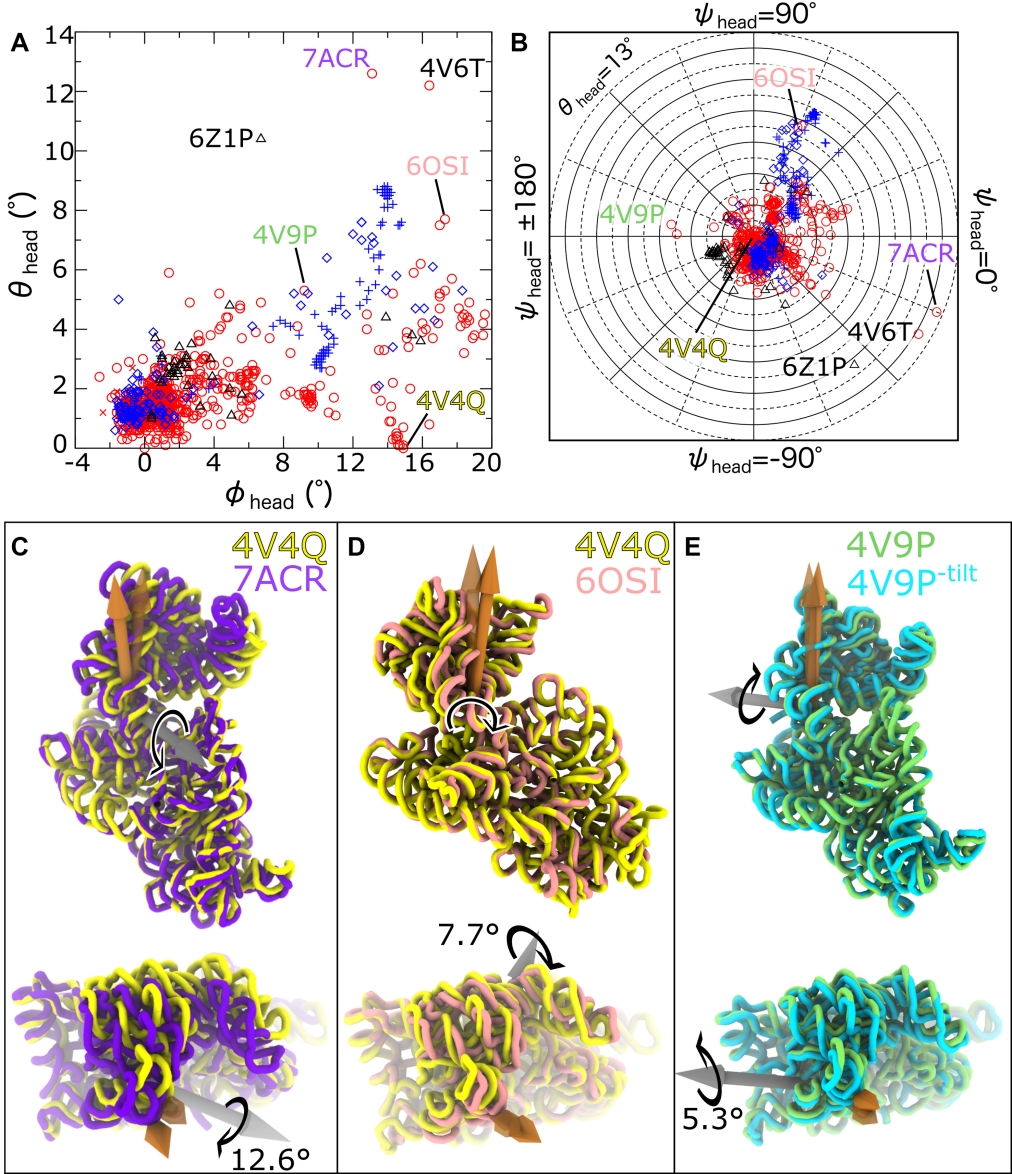
The various modes of head tilting. (**A**) SSU head tilt angle θ_head_ versus head rotation/swivel angle ϕ_head_ for the full set of 1208 ribosome structures (symbols as in Figure 4A). In prokaryotic (red) and eukaryotic (blue) ribosomes, θ_head_ reaches values as large as ∼5° for most values of ϕ_head_. For highly-rotated/swiveled head configurations (ϕ_head_ > 10°), there is only a small set of structures for which tilting is absent (θ_head_ < 1°). (**B**) Comparing tilt angles and tilt directions reveals the multiple ways in which head tilting can manifest in prokaryotic and eukaryotic ribosomes. For prokaryotes, the most highly-tilted orientations correspond to tmRNA complex (panel C, purple), where the head appears to ‘open’ roughly along the mRNA axis. The rotated and untilted *E. coli* structure is shown in yellow (RCSB ID: 4V4Q (33)). (**D**) In a frameshift-related structure (pink), the head is tilted in a direction that is perpendicular to the direction of tmRNA-associated tilting. Note: In the top panel the rotation axis is directed into the page, such that it is occluded by the SSU structure. (**E**) The head can also tilt towards the LSU (RCSB ID: 4V9P (76); green tubes). For comparison, a structural model of *E. coli* is shown, where the head is rotated and θ_head_ is set to 0 (labeled 4V9P^-tilt^; cyan).

The highly dynamic SSU head exhibits a complex range of orientations that involves rotations, as well as tilting in multiple directions. The RAD method illustrates three broad features of head dynamics. First, there is a large range of tilt angles adopted for each value of the rotation angle (Figure 6A). Second, unlike the body, large values of the head-tilt angle are found to occur along multiple directions (Figure 6B). Third, the range of head tilt values is found to be larger in prokaryotic than eukaryotic (mitochondrial or cytosolic) ribosomes, though the presence of tilting is common across kingdoms of life. Finally, there is a larger range of head tilting values in structures of isolated SSUs and pre-rRNA assembly intermediates (Supplementary Figure S9).

When considering bacterial ribosomes, published structures display head orientations that are tilted in several clearly-identifiable directions (Figure 6B). The two largest values of θ_head_ (12.6° and 10.2°) are found in *E. coli* ribosomes in post-translocation intermediate states associated with *trans*-translation (RCSB ID: 7ACR (74), 4V6T (15)). Consistent with descriptions in the original reports, these tilt-like rearrangements are roughly centered about the track that contacts the mRNA codons in the A, P and E sites (ψ_head_ ∼ −22.5° and −30.7°; Figure 6C, Movie S1). For reference, ψ_head_ = 0 corresponds to rotation about an axis that is parallel to the vector pointing from the E-site to A-site mRNA codons (Figure 2 and Supplementary Figure S10), with the head displaced away from the LSU. The next two most highly tilted head orientations (θ_head_ = 7.7° and 7.5°) are found in the *T. Thermophilus* ribosome in the presence of a so-called ‘slippery proline sequence’ (13) that is prone to frameshifting (RCSB ID: 6OSI (75)). In relation to the tmRNA-induced tilting, the tilting axis associated with this +1 frameshifting intermediate is nearly perpendicular (ψ_head_ ∼ 67° versus −22.5°; Figure 6D), where the head tilts roughly in the direction of the SSU shoulder. There are then 36 additional structures for which θ_head_ ≥ 4°, with directions that span from ψ_head_ ∼ −53.7° to 178.3°, where the direction and scale depend on the biological context. For ψ_head_ = 178.3°, the head tilts about the A/P/E-site mRNA codons, in the direction of the LSU (Figure 6E). Overall, this illustrates how quantitative mapping of structures allows one to directly isolate modes of head tilting that are associated with different stages of translation.

In terms of tilt directions, the distribution of head tilt orientations in cytosolic eukaryotic ribosomes is more homogeneous than in bacteria. Interestingly, for eukaryotic ribosomes, almost all highly-tilted conformations (θ_head_ > 4°) are tilted in the same general direction (45° < ψ_head_ < 75°). This also coincides with the tilt direction seen in *T. thermophilus* structures obtained from frameshift-prone complexes (Figure 6D), where the head tilts in the direction of the upstream mRNA and slightly away from the LSU.

As a final quantification of head tilt values and directions, we compared the relative tilt differences between bacterial and eukaryotic ribosomes. As described for body tilting (see previous section), we calculated the angle formed by the head rotation axes in different models: δθ_head_. For bacteria, the largest value of δθ_head_ was 17.7° (RCSB IDs: 4V9P (76) with 7ACR (74)). In cytosolic eukaryotic ribosomes the maximum tilt difference was 11.6° (RCSB IDs: 4U52 (77) with 6ZME (78)), and the largest difference between mitochondrial ribosomes was 13.1° (RCSB IDs: 6YDW (79) with 6Z1P (80)). While the tilt differences are larger in bacteria, this analysis reveals that head tilting is common across organisms.

### The (currently) most distinct subunit orientations

From a practical perspective, our approach can be used to quickly determine whether a newly-resolved structure represents a novel body or head orientation. Additionally, one can identify which previously-resolved structure has the most similar domain orientations. For this, it is convenient to re-express the differences in RAD angles obtained for two structures as a single E–R angle. In contrast to RAD/Euler angles, the E–R angle does not distinguish between rotation and tilting, but rather provides a 1D measure of the net difference (rotation + tilt) between two orientations. (11) Accordingly, calculating the E–R angle between a model and all published structures provides a direct method for determining if a structure is distinct (when translational displacements are small), or if similar orientations are present in any organism.

To compare all SSU body orientations, we calculated the E–R angles ($\phi ^{\rm E-R}_{\rm body}$) between all possible ribosome pairs (1208*1207/2 comparisons). Here, we define the nearest neighbor as the structure for which $\phi ^{\rm E-R}_{\rm body}$ is minimal: $\min \lbrace \phi ^{\rm E-R}_{\rm body}\rbrace$. This revealed that 57 structural models had other structures with identical orientations ($\min \lbrace \phi ^{\rm E-R}_{\rm body}\rbrace =0$). We also find that almost every published structure (1187 of 1208) has a neighbor that can be related through rotation of less than one degree ($\min \lbrace \phi ^{\rm E-R}_{\rm body}\rbrace \leq1^\circ$). Of the remaining models, $\min \lbrace \phi ^{\rm E-R}_{\rm body}\rbrace >2^\circ$ for only a single structure (RCSB ID: 4V74 (56)). However, as discussed above, this structure was obtained from a rather low-resolution (17 Å) cryo-EM reconstruction. Excluding this single outlier, our analysis shows that every published body orientation has a nearly indistinguishable neighboring structure (<2° differences).

While there is a lack of clearly distinct orientations of the SSU body within the published literature, we find a small set of distinct SSU head orientations in LSU–SSU assemblies. Similar to the analysis of the body domains, we evaluated $\min \lbrace \phi ^{\rm E-R}_{\rm head}\rbrace$ to determine the nearest neighbors for the head. There are 10 configurations for which $\min \lbrace \phi ^{\rm E-R}_{\rm head}\rbrace >2^\circ$. Of these, 6 are from structures for which the resolution is better than 4 Å. Interestingly, this set of distinct orientations spans multiple organisms. The most distinct orientation is from a mitoribosome in *Tetrahymena thermophilia* (RCSB ID: 6Z1P (80)). There are also *E. coli* structures in which the head is highly rotated during EF-G-associated translocation (RCSB ID: 4V9O (76) and 4V9P (76)), or highly tilted during tmRNA rescue (RCSB ID: 4V6T (15), 7ACR (74)). In *T. thermophilus*, the ribosome with ribosome modulation factor (RMF) in a hibernating state is nearly classical, though slightly rotated and tilted (RCSB ID: 4V8G (81)). Together, this analysis further highlights how versatility of the SSU head orientation may be used to execute distinct biological functions during protein synthesis.

### Example application to smFRET studies

In addition to aiding structural studies, the presented decomposition of subunit orientations can be leveraged to design and interpret smFRET experiments on the ribosome. When designing smFRET studies, one will typically select a small set of structures and identify residue pairs that are expected to exhibit a significant change in distance upon a conformational change of interest. While this can be an effective strategy for determining candidate labeling sites, a manually curated subset of structures may not reflect the full range of accessible configurations. Using the RAD method, one may overcome this limitation by considering all known structures at the design stage of a study. To illustrate the utility of this approach, we will assess previously-deployed smFRET labeling pairs that were designed to probe rotation of the SSU body. As described below, this can provide a more precise description of the relationship between smFRET measurements and the underlying motion of interest.

There have been several efforts to deploy smFRET probes that are sensitive to SSU body rotation. For example, Cornish et al. (16) labeled the protein pairs S6-L9 and S11-L9 (Supplementary Figure S11). The distances between these protein pairs will be denoted by *R*_S6, L9_ and *R*_S11, L9_. Based on inspection of rotated and unrotated structures, it was expected that rotation of the body would lead to an increase in *R*_S6, L9_ and a decrease in *R*_S11, L9_. In another study, Marshall et al. (17) developed a different strategy, where h44 in the SSU and H101 in the LSU were extended by several base pairs and fluorophore-labeled DNA oligonucleotides were bound to the extensions. In this approach, the distance between the turns of h44 and H101 (*R*_h44, H101_) was expected to increase upon rotation. While structural considerations were used to design both assays, the studies provided conflicting interpretations. Specifically, Cornish *et al.* reported the observation of spontaneous, thermally driven fluctuations between low- and high-FRET states when the ribosome was in a pre-translocation state. This was interpreted as signifying spontaneous rotation and back-rotation events prior to the completion of tRNA translocation. In contrast, Marshall *et al.* did not observe reversible transitions between FRET states, implying that translocation is associated with a single cycle between rotated and unrotated states, which the authors interpreted as being driven by peptide bond formation and GTP hydrolysis by translation elongation factor G, respectively.

There have been various attempts to reconcile the conflicting observations provided by the studies of Cornish and Marshall. As an example, it was argued by Chen et al. (82) that the probes used by Cornish et al. were reporting on head motion, rather than body motion. To explore this argument, we used RAD analysis of all *E. coli* structures (5Å resolution and better) to examine the relationship between probe distances and rotation of the head and body. As a note, we only considered the distances between the labeled sites in each experiment, which does not account for other factors that may be influenced by the experimental design (e.g. fluorophore interactions, mutations, buffer differences, etc.).

**Figure 7.**
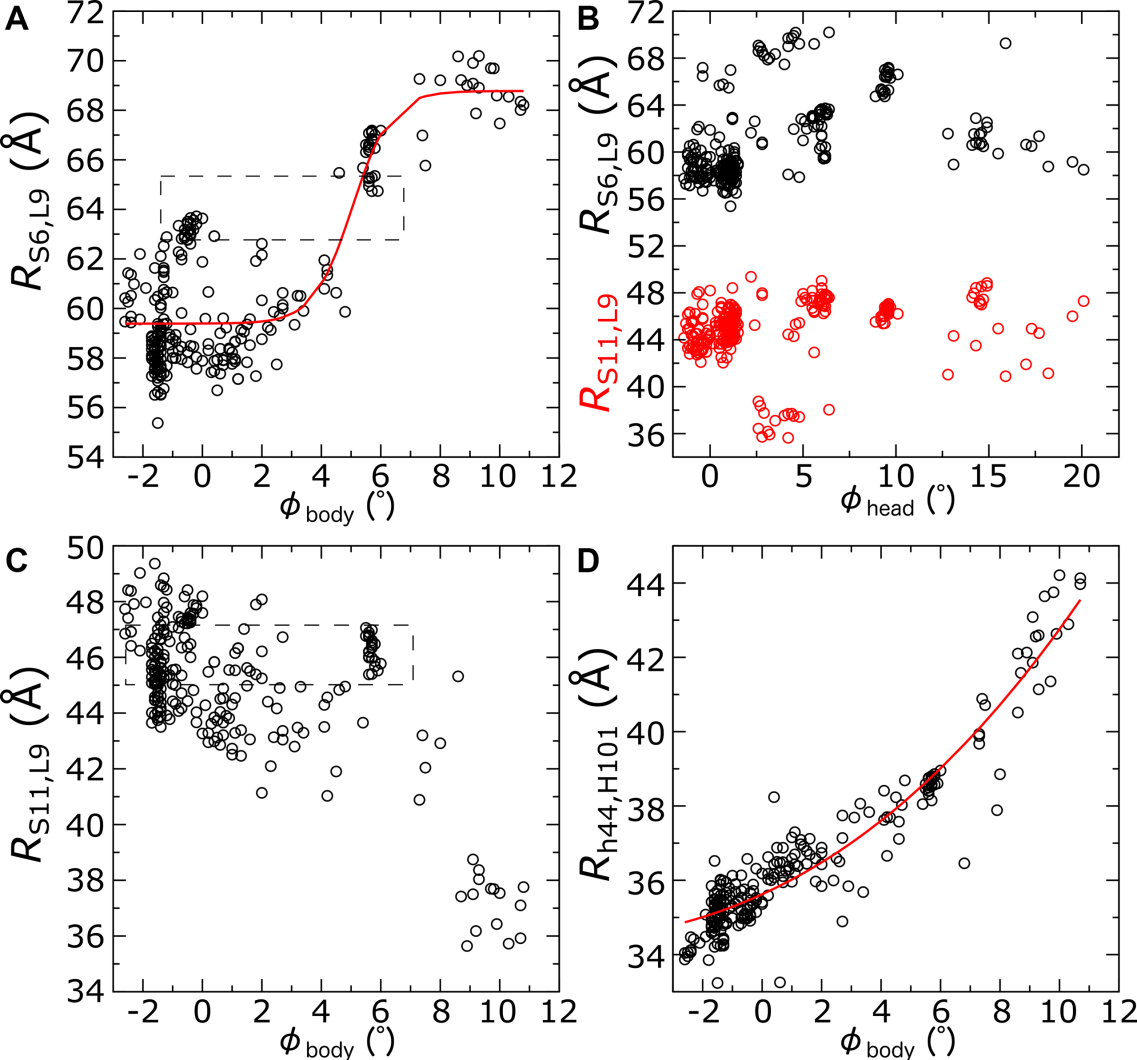
Relating rotation states to single-molecule observables. To assess the utility of specific single-molecule probes, we evaluated the distances between the corresponding labeling sites (Supplementary Figure S11) and the rotation angles. (**A**) Distance between residues in S6 and L9 that were labeled by Cornish *et al.* (16) (*R*_S6, L9_), versus the body rotation angle ϕ_body_. Plot shows all structures of *E. coli* ribosomes that included the labeled residues and had a resolution of 5Å or better. A sigmoidal function (tanh ) was fit (red), which indicates these probes are likely to be most responsive to transitions between intermediate (∼4°) and highly rotated (∼10°) states. However, intermediate FRET signals could arise from unrotated or partially rotated conformations (boxed). (**B**) *R*_S6, L9_ and the distance between S11 and L9 labeling sites (*R*_S11, L9_), versus the head rotation angle ϕ_head_. Consistent with the interpretation of Cornish *et al.* (16), neither distance is correlated with head rotation. (**C**) *R*_S11, L9_ as a function of body rotation. While a narrow range of *R*_S11, L9_ values can be found for ϕ_body_ values that span from –2° to 6° (boxed), there is a sharp decrease in distance as the ribosome reaches larger body rotation angles. Similar to the S6–L9 pair, the S11–L9 pair is most responsive over a very small range of rotation angles. (**D**) Distance between the turns of h44 and H101 (*R*_h44, H101_), which was probed in the study of Marshall *et al.* (17). *R*_h44, H101_ is correlated with rotation across the full range of ϕ_body_ values. A power law fit is shown in red, whereas a sigmoidal function did not fit the data well.

Our assessment of available structures shows that the probes used by Cornish *et al.* and Marshall *et al.* exhibit different degrees of correlation with (and sensitivity to) body rotation. In the case of the S6–L9 pair, there is a visible correlation between *R*_S6, L9_ and the body rotation angle ϕ_body_ (Figure 7A), which supports the interpretation of Cornish et al. This analysis indicates that one may confidently assign high-FRET signals (short distances) to an unrotated state and low-FRET signals (large distances) to a fully rotated state. However, mid-FRET values (intermediate distances; boxed in Figure 7A) should be compatible with unrotated (ϕ_body_ < 2°) and partially rotated (ϕ_body_ ≈ 6°) conformations. We fit a sigmoidal function (tanh ) to the data and find this pair is most responsive to rotation changes that are in the range 4° < ϕ_body_ < 7°. To address the proposal of Chen et al. (i.e. that these probes report on head motion), we also compared *R*_S6, L9_ with head rotation. We find no correlation of *R*_S6, L9_ with ϕ_head_ (Figure 7B), again consistent with the interpretation of Cornish *et al.*

We also considered the distance between S11 and L9. In agreement with the interpretation of Cornish et al., body rotation and *R*_S11, L9_ are anticorrelated (Figure 7C). However, for intermediate distances (e.g. 45Å < *R*_S11, L9_ < 47Å), ϕ_body_ varies from −2° to 6° (boxed in Figure 7C), though *R*_S11, L9_ exhibits a sharp decrease at ϕ_body_ ∼ 8°. Accordingly, similar to the S6–L9 probes, mid-FRET values obtained with S11–L9 are compatible with unrotated and partially rotated (ϕ_body_ ≈ 6°) conformations, while high-FRET values (short distances) would only be compatible with highly rotated (ϕ_body_ ≈ 10°) conformations. Similar to *R*_S6, L9_, we find that *R*_S11, L9_ is not correlated with head rotation (Figure 7B).

Of the distances considered, *R*_h44, H101_ exhibits the clearest correlation with body rotation (Figure 7D). Unlike *R*_S6, L9_, *R*_h44, H101_ increases nearly monotonically with ϕ_body_. Thus, if one were to directly monitor the distance between the centers of these two helices, and the subunits were to move as rigid bodies, one could expect the signal to be responsive to changes across the full range of rotation states, as proposed by Marshall *et al.*

Taken together, our analysis provides evidence that the study of Cornish et al. measured body motion, rather than head motion, though additional considerations are needed to fully reconcile the controversy with Marshall et al. To this end, it is important to address the potential influence of molecular flexibility on smFRET observations. Here, we considered the distance between h44 and H101, though the approach of Marshall et al. used mutant ribosomes with extended helices, for which structural data is not available. It is reasonable to expect these extended regions to exhibit larger-scale structural fluctuations than the shorter native helices. In addition, theoretical models predict that the native helices are also very flexible, such that body rotation and *R*_h44, H101_ are only weakly coupled (83). This may be further exacerbated by the flexibility in and around proteins S6, S11 and L9, making it reasonable to expect differential dynamics along *R*_S6, L9_ and *R*_h44, H101_.

The above example illustrates how a comprehensive assessment of known structures, along with a systematic mapping of orientations, may be used to better understand the significance of measured FRET signals. In future efforts, this approach may be applied prior to designing a new experimental assay, and it may be used to study ribosomes of any organism for which multiple structures are available, including eukaryotic (cytosolic or mitochondrial) ribosomes.

## DISCUSSION

In the current study, we systematically analyzed and compared almost every published ribosome structure. While the speed at which ribosome structures are being reported is already astounding (>200 structures in 2021), advances and accessibility of structure determination methods are likely to further accelerate these efforts. This will exacerbate the need for a robust approach that can describe, compare and categorize an ever-expanding set of resolved biological states.

The current study provides a foundation for comparative analysis of subunit orientations that may be further developed in a number of directions. The most obvious immediate challenge will be to identify, classify and compare tRNA configurations in different models. There are also opportunities to adapt the techniques presented here to automatically detect and describe ribosomal proteins, or to combine RAD angles with available protein classifications (84). The aim would be for the field to move away from considering manually selected subsets of available structures. Instead, as we have shown for subunit orientations, one may envision being able to rapidly query information on any number of ribosomal structural features. This will certainly have much utility in experimental efforts, in particular when designing labeling strategies for smFRET experiments. In addition to aiding the design of experiments, these advances would also allow one to easily identify all structures that are compatible with a given FRET measurement.

One limitation of the current approach is that it relies on a few assumptions regarding the composition of a ribosome. Specifically, the current protocols assume each ribosome contains two major rRNA strands (>500 residues long), one for the LSU and SSU. However, there are some examples of published ribosome structures that do not satisfy this condition. Specifically, in *Euglena gracilis* (RCSB ID: 6ZJ3 (85)) the ribosomal rRNA is composed of 14 mid-length RNA molecules, which differs dramatically from the composition in bacteria and most eukaryotes. Similarly, mitoribosomes in trypanosomes (e.g. RCSB IDs: 6HIV (86), 7AOR (87)) have smaller rRNA molecules, where much of the rRNA scaffolding has been replaced with protein elements. For these cases, the currently deployed structure-alignment strategies are insufficient, since the rRNA motifs have diverged significantly from bacteria. Nonetheless, it will be valuable to establish generalizations of the proposed structural metrics for quantitative comparison of these atypical ribosomal architectures.

To conclude, as the ribosome field continues to advance, it is becoming increasingly difficult to rigorously determine if a new model is similar to the vast number of known structures. However, it is essential that common metrics and analysis pipelines are available, in order to pose deeper questions about the factors that regulate ribosome dynamics. Here, we have demonstrated how a general strategy can be used to consistently analyze essentially every known structure of the ribosome. While this has corroborated qualitative descriptions that have been reported in the literature, it also provides quantitative evidence for previously-unrecognized aspects of subunit dynamics, such as the notable scale of tilting/rolling of the SSU body in bacterial ribosomes. With this foundation, it is now possible to precisely describe and compare all subunit orientations, and thereby determine which structural features are universal, and which are organism/domain-specific.

## DATA AVAILABILITY

To facilitate reproducibility of all results, the RAD analysis pipeline is implemented in a plugin for the Visual Molecular Dynamics (VMD) (88) software package. VMD is a powerful visualization and analysis program that can be used for experimental structures, as well as simulated trajectories. The plugin and additional analysis modules are freely available for anonymous download/access at https://github.com/Whitford/radtool and http://www.radtool.org.

## Supplementary Material

gkac1211_Supplemental_Files
