# Supplementary material for: Ratchet, swivel, tilt and roll: a complete description of subunit rotation in the ribosome
Source: Nucleic Acids Res. 2022 Dec 30;51(2):919–34. doi: 10.1093/nar/gkac1211 (PMC9881166; doi:10.1093/nar/gkac1211)
Supplement: gkac1211_Supplemental_Files [file gkac1211_supplemental_files.zip › AppendixA.pdf]

Appendix A: Ribosome rotation angles calculated with the RAD method - Assembled LSU-SSU pairs

Below are tables that provide all 1208 calculated sets of subunit orientations, obtained from 901 RCSB accession codes.
For each entry, the organism name, reference, method and resolution were extracted directly from the RCSB database query system, using the following protocol:

-*Organism names*: From the query system, each entry has a list of `polymer_entities`. The longest nucleic acid chain was determined through the `entity_poly.rcsb_entity_polymer_type` and `entity_poly.rcsb_sample_sequence_length` values of each polymer in the RCSB entry. In the tables below, the organism name corresponds to the `rcsb_entity_source_organism.[0].ncbi_scientific_name` value for the longest nucleic acid chain. While many ribosome structures have molecular elements from multiple organisms, this protocol was applied, so that the organism name corresponds to that of the LSU rRNA.

-*Experimental details*: For each entry in the RCSB database, the method listed is from the `exptl.[0].method` element, while the resolution listed in the table is `rcsb_entry_info.resolution_combined.[0]`.

-*Mitoribosomes*: Since there is not a specific entry that indicates whether a ribosome is a mitoribosome, the following process was used to identify mitoribosomes: If the `struct.title` element of the RCSB entry contained "mitoribo" or "mitochond" (case-insensitive), then a * is included in the tables to indicate the structure is a mitoribosome.

-*References*: References are given in the following format: Last name of first author (year) *Journal*
The listed citations are obtained from the RCSB query system by checking the array of references given under the `citation` element for the entry. The value of `rcsb_is_primary` was checked for each citation listed. If Yes was found, the reference was used for the table. For space considerations, only one author is listed for each reference. The author listed in each table entry corresponds to the `rcsb_authors.[0]` element. If the journal or year is not available in the RCSB system, it is omitted from the table.
Abbreviations: In the table, the following acronyms are used for journal names:

- AAC: Antimicrobial Agents and Chemotherapy
- ACIEE: Angew Chem Int Ed Engl
- JMB: Journal of Molecular Biology
- NAR: Nucleic Acid Research
- NCB: Nature Chemical Biology
- NComm: Nature Communications
- NMb: Nature Microbiology
- NSMB: Nature Structural and Molecular Biology
- PNAS: Proceedings of the National Academy of Sciences USA

Notes: When using VMD 1.9.4 (pre-release alpha version), the RADtool -download option was unable to automatically calculate angles for the following RCSB entries: 1ML5, 3DG0, 3DG2, 3DG4, 3DG5, 4V42, 4V47, 4V48, 4V5Z, 6XIQ, 7NWH, 7NWI. For 1ML5, 3DG0, 3DG2, 3DG4, 3DG5, 4V42, 4V47, 4V48 and 4V5Z, the method was unable to automatically identify the subunits, since they only contained P atoms. However, it was called manually with flags to indicate the chain IDs, and all angles were calculated. For 6XIQ, 7NWH, 7NWI, RADtool -download was able to automatically process the structures when using VMD v1.9.3. However, when using VMD 1.9.4 (pre-release alpha version), these entries failed, due to an issue with chain identification when using the CIF file format. We anticipate this issue will be resolved prior to the official release of VMD 1.9.4. If a tilt angle is less then 0.1, the tilt direction is listed as ND (not defined). Entries 4V8M, 5T2A, 6HRM and 6ZJ3 contain complete ribosomes, but the RAD method was unable to successfully perform structure alignment for the LSU rRNA. Accordingly, only head angles could be calculated, and those values are provided in Appendix B.

Table 1 of 24

PDB		BODY				HEAD				PRUNED			RMSD			EXP DETAILS		ORGANISM		REFERENCE
ID	chains	ϕ_{body}	θ_{body}	ψ_{body}	$\lvert\Delta\vec{x}_{\text{body}}\rvert$	ϕ_{head}	θ_{head}	ψ_{head}	$\lvert\Delta\vec{x}_{\text{head}}\rvert$	LSU	body	head	LSU	body	head	method	res.	name	mito	
1ML5	a,A	-1.5	0.8	22.1	0.956	1.1	0.3	-116.2	0.746	1154	597	212	1.37	1.39	1.35	EM	14.0	E. coli		Klaholz, et al. (2003) *Nature*[1]
1VVJ	RA,QA	-0.3	0.7	-42.1	0.065	0.2	1.0	-23.0	0.137	2122	888	418	0.96	0.92	0.92	XRAY	3.4	T. thermophilus HB8		Maehigashi, et al. (2014) *PNAS*[2]
1VVJ	YA,XA	1.2	0.3	-105.7	0.500	-0.5	1.5	-14.2	0.252	2092	863	409	0.96	0.98	0.88	XRAY	3.4	T. thermophilus HB8		Maehigashi, et al. (2014) *PNAS*[2]
1VY4	BA,AA	1.4	0.3	-27.5	0.706	-0.7	1.4	-28.0	0.042	2101	865	419	0.94	0.94	0.84	XRAY	2.6	T. thermophilus HB8		Polikanov, et al. (2014) *NSMB*[3]
1VY4	DA,CA	-0.3	0.7	-24.6	0.176	0.5	1.1	-43.6	0.116	2131	893	414	0.94	0.89	0.89	XRAY	2.6	T. thermophilus HB8		Polikanov, et al. (2014) *NSMB*[3]
1VY5	BA,AA	1.4	0.3	-24.7	0.599	-0.8	1.4	-24.4	0.090	2089	862	420	0.95	0.92	0.84	XRAY	2.5	T. thermophilus HB8		Polikanov, et al. (2014) *NSMB*[3]
1VY5	DA,CA	-0.4	0.7	-25.3	0.151	0.4	1.1	-39.6	0.143	2123	893	412	0.95	0.91	0.86	XRAY	2.5	T. thermophilus HB8		Polikanov, et al. (2014) *NSMB*[3]
1VY6	BA,AA	1.4	0.3	-59.4	0.709	-0.9	1.5	-28.2	0.067	2084	863	417	0.95	0.94	0.83	XRAY	2.9	T. thermophilus HB8		Polikanov, et al. (2014) *NSMB*[3]
1VY6	DA,CA	-0.7	0.6	-30.5	0.148	1.0	1.3	-50.1	0.255	2107	886	412	0.94	0.94	0.89	XRAY	2.9	T. thermophilus HB8		Polikanov, et al. (2014) *NSMB*[3]
1VY7	BA,AA	1.4	0.2	-64.0	0.948	-0.8	1.6	-29.3	0.666	2028	866	413	1.00	0.98	0.82	XRAY	2.8	T. thermophilus HB8		Polikanov, et al. (2014) *NSMB*[3]
1VY7	DA,CA	-0.7	0.7	-35.5	0.707	1.1	1.2	-54.0	0.554	2079	879	412	1.01	0.95	0.91	XRAY	2.8	T. thermophilus HB8		Polikanov, et al. (2014) *NSMB*[3]
3DG0	B,A	7.8	2.4	33.9	2.292	5.4	1.1	-45.2	1.326	1650	740	303	1.44	1.34	1.21	EM	10.8	E. coli		Gao, et al. *To be published*
3DG2	B,A	0.8	1.1	30.8	1.106	1.0	1.0	-161.6	0.600	1488	581	237	1.41	1.36	1.26	EM	10.0	E. coli		Gao, et al. *To be published*
3DG4	B,A	-0.8	1.5	13.4	1.270	2.8	1.1	-161.5	0.223	1632	596	241	1.41	1.30	1.40	EM	12.8	E. coli		Gao, et al. *To be published*
3DG5	B,A	5.8	2.5	29.3	2.240	5.7	1.0	-83.9	2.251	1011	512	223	1.41	1.32	1.28	EM	15.5	E. coli		Gao, et al. *To be published*
3J6X	2S,1S	4.2	3.1	58.5	1.319	8.1	4.2	48.8	1.877	1029	520	249	1.36	1.34	1.26	EM	6.1	S. cerevisiae W303		Koh, et al. (2014) *PNAS*[4]
3J6Y	2S,1S	1.4	2.5	46.5	1.278	8.4	4.1	49.4	1.251	1144	566	242	1.37	1.29	1.27	EM	6.1	S. cerevisiae W303		Koh, et al. (2014) *PNAS*[4]
3J77	2S,1S	8.5	5.3	33.2	0.997	0.9	3.1	-59.9	1.103	1226	524	236	1.33	1.28	1.34	EM	6.2	S. cerevisiae		Svidritskiy, et al. (2014) *Structure*[5]
3J78	2S,1S	-2.0	2.8	21.1	0.980	-0.5	1.6	-75.9	1.031	1265	538	258	1.33	1.27	1.29	EM	6.3	S. cerevisiae		Svidritskiy, et al. (2014) *Structure*[5]
3J7P	5,S2	6.2	4.8	40.2	0.793	10.5	6.4	64.7	0.287	1407	586	238	1.21	1.22	1.27	EM	3.5	Sus scrofa		Voorhees, et al. (2014) *Cell*[6]
3J7R	5,S2	8.6	5.6	36.7	0.309	1.0	1.2	7.8	0.646	1410	586	256	1.21	1.22	1.28	EM	3.9	Sus scrofa		Voorhees, et al. (2014) *Cell*[6]
3J9M	A,AA	0.0	0.3	-73.7	2.383	2.4	3.0	-159.6	1.768	1010	338	202	1.02	1.09	1.14	EM	3.5	Homo sapiens	*	Amunts, et al. (2015) *Science*[7]
3J9W	BA,AA	-1.4	2.1	7.5	0.584	1.0	1.5	-130.5	1.506	2303	913	421	1.04	1.00	0.89	EM	3.9	B. subtilis subsp. subtilis str. 168		Sohmen, et al. (2015) *NComm*[8]
3J9Y	A,a	-1.4	1.1	9.4	0.459	0.8	2.7	-79.2	0.771	2624	989	446	0.63	0.86	0.75	EM	3.9	E. coli		Arenz, et al. (2015) *PNAS*[9]
3J9Z	LA,SA	-1.7	1.5	15.3	0.714	1.1	2.6	-90.3	1.317	2572	981	431	0.78	0.91	0.93	EM	3.6	E. coli		Li, et al. (2015) *Sci Adv*[10]
3JA1	LA,SA	8.6	1.9	35.0	0.981	4.6	2.0	-40.6	0.747	2493	906	411	0.93	0.98	1.10	EM	3.6	E. coli		Li, et al. (2015) *Sci Adv*[10]
3JAG	5,9	-2.0	2.8	29.1	1.249	-1.5	1.4	-84.1	1.098	1447	627	297	1.20	1.17	1.12	EM	3.6	Oryctolagus cuniculus		Brown, et al. (2015) *Nature*[11]
3JAH	5,9	-2.1	2.9	28.8	1.270	-1.4	1.4	-87.7	1.134	1429	631	296	1.20	1.19	1.13	EM	3.5	Oryctolagus cuniculus		Brown, et al. (2015) *Nature*[11]
3JAI	5,9	-2.0	2.8	30.6	1.206	-1.5	1.5	-81.2	1.164	1435	631	292	1.20	1.15	1.10	EM	3.6	Oryctolagus cuniculus		Brown, et al. (2015) *Nature*[11]
3JAJ	5,S2	-2.0	2.8	24.8	1.275	-0.2	1.2	-64.3	1.496	1421	590	263	1.23	1.22	1.25	EM	3.8	Oryctolagus cuniculus		Voorhees, et al. (2015) *Elife*[12]
3JAN	5,S2	-2.1	2.9	20.5	1.520	-0.2	1.2	-64.3	1.496	1422	590	263	1.23	1.22	1.25	EM	3.8	Oryctolagus cuniculus		Voorhees, et al. (2015) *Elife*[12]
3JBN	AA,A	-1.4	2.6	20.2	1.279	-0.4	2.5	-89.7	1.168	1274	588	146	1.31	1.28	1.26	EM	4.7	Plasmodium falciparum 3D7		Sun, et al. (2015) *NAR*[13]
3JBO	AA,A	8.4	4.5	37.3	1.606	1.5	1.7	-52.7	1.061	1286	561	140	1.30	1.27	1.21	EM	5.8	Plasmodium falciparum 3D7		Sun, et al. (2015) *NAR*[13]
3JBP	AA,A	-2.2	2.5	16.4	1.448	4.0	2.2	115.2	1.573	1336	594	137	1.27	1.23	1.21	EM	6.7	Plasmodium falciparum 3D7		Sun, et al. (2015) *NAR*[13]
3JBU	b,A	-1.6	1.3	6.6	2.397	1.3	2.0	-93.0	2.624	2111	767	377	1.32	1.32	1.22	EM	3.6	E. coli K-12		Zhang, et al. (2015) *Elife*[14]
3JBV	b,A	8.8	2.3	21.2	2.160	5.2	1.9	-31.7	1.176	2235	859	398	1.27	1.17	1.12	EM	3.3	E. coli K-12		Zhang, et al. (2015) *Elife*[14]
3JCD	A,a	-1.5	1.3	10.4	1.651	1.1	1.5	-99.7	1.671	2527	970	425	1.10	1.07	0.99	EM	3.7	E. coli K-12		Zhang, et al. (2016) *NSMB*[15]
3JCE	A,a	2.2	1.2	22.8	0.840	-1.0	1.6	-38.2	1.038	2523	835	438	1.04	1.20	0.89	EM	3.2	E. coli K-12		Zhang, et al. (2016) *NSMB*[15]
3JCJ	A,g	2.0	1.6	36.9	0.307	2.4	1.8	-17.7	0.715	2628	1033	462	0.81	0.73	0.74	EM	3.7	E. coli		Sprink, et al. (2016) *Sci Adv*[16]
3JCN	A,a	6.3	3.2	31.9	0.776	4.8	1.8	-34.2	0.653	2636	1059	462	0.71	0.62	0.66	EM	4.6	E. coli		Sprink, et al. (2016) *Sci Adv*[16]
4L47	YA,XA	1.2	0.2	-93.3	0.642	-0.6	1.6	-19.9	0.358	2093	867	416	0.97	1.03	0.88	XRAY	3.2	T. thermophilus HB8		Maehigashi, et al. (2014) *PNAS*[2]
4L47	RA,QA	-0.2	0.7	-31.7	0.250	0.0	1.2	-29.3	0.432	2130	886	418	0.98	0.94	0.91	XRAY	3.2	T. thermophilus HB8		Maehigashi, et al. (2014) *PNAS*[2]
4L71	YA,XA	1.2	0.4	-107.6	0.909	-0.5	1.6	-16.7	0.402	2084	847	414	1.02	1.04	0.88	XRAY	3.9	T. thermophilus HB8		Maehigashi, et al. (2014) *PNAS*[2]
4L71	RA,QA	-0.2	0.5	-49.3	0.599	0.0	1.1	-36.1	0.539	2124	893	415	0.98	0.95	0.91	XRAY	3.9	T. thermophilus HB8		Maehigashi, et al. (2014) *PNAS*[2]
4LEL	YA,XA	1.1	0.4	-142.4	0.200	-0.5	1.4	-15.3	0.440	2072	867	411	0.99	1.05	0.89	XRAY	3.9	T. thermophilus HB8		Maehigashi, et al. (2014) *PNAS*[2]
4LEL	RA,QA	0.0	0.5	-65.7	0.145	-0.2	1.2	-34.8	0.361	2103	883	412	0.99	0.98	0.93	XRAY	3.9	T. thermophilus HB8		Maehigashi, et al. (2014) *PNAS*[2]
4LFZ	YA,XA	1.0	0.2	-85.9	0.751	-0.6	1.4	-22.7	0.177	2109	892	413	0.97	0.95	0.89	XRAY	3.9	T. thermophilus HB8		Maehigashi, et al. (2014) *PNAS*[2]
4LFZ	RA,QA	-0.6	0.7	-30.9	0.112	0.7	1.0	-37.7	0.275	2121	893	416	0.96	0.95	0.95	XRAY	3.9	T. thermophilus HB8		Maehigashi, et al. (2014) *PNAS*[2]
4LNT	YA,XA	1.2	0.3	-89.6	0.641	-0.6	1.5	-20.8	0.222	2096	874	413	0.96	1.00	0.84	XRAY	2.9	T. thermophilus HB8		Maehigashi, et al. (2014) *PNAS*[2]
4LNT	RA,QA	-0.2	0.6	-36.6	0.247	0.0	0.9	-22.9	0.366	2118	893	416	0.96	0.92	0.88	XRAY	2.9	T. thermophilus HB8		Maehigashi, et al. (2014) *PNAS*[2]
4LSK	YA,XA	1.1	0.3	-97.1	0.735	-0.5	1.4	-19.7	0.263	2068	869	413	0.98	1.01	0.88	XRAY	3.5	T. thermophilus HB8		Maehigashi, et al. (2014) *PNAS*[2]
4LSK	RA,QA	0.0	0.6	-35.3	0.434	-0.3	1.0	-31.6	0.272	2112	889	415	0.97	0.94	0.91	XRAY	3.5	T. thermophilus HB8		Maehigashi, et al. (2014) *PNAS*[2]

Table 2 of 24

PDB		BODY				HEAD				PRUNED			RMSD			EXP DETAILS		ORGANISM		REFERENCE
ID	chains	ϕ_{body}	θ_{body}	ψ_{body}	$\lvert\Delta\vec{x}_{\text{body}}\rvert$	ϕ_{head}	θ_{head}	ψ_{head}	$\lvert\Delta\vec{x}_{\text{head}}\rvert$	LSU	body	head	LSU	body	head	method	res.	name	mito	
4LT8	YA,XA	1.1	0.3	-102.5	0.411	-0.5	1.5	-19.1	0.113	2087	877	414	0.95	1.00	0.84	XRAY	3.1	T. thermophilus HB8		Maehigashi, et al. (2014) *PNAS*[2]
4LT8	RA,QA	-0.2	0.7	-39.1	0.175	0.0	0.9	-20.2	0.229	2115	893	416	0.95	0.91	0.87	XRAY	3.1	T. thermophilus HB8		Maehigashi, et al. (2014) *PNAS*[2]
4P6F	YA,XA	1.6	0.4	-139.8	0.497	-0.7	1.7	-28.5	0.242	2085	845	412	0.98	1.06	0.85	XRAY	3.6	T. thermophilus HB8		Washington, et al. (2014) *ACS Chem Biol*[17]
4P6F	RA,QA	0.4	0.3	-84.6	0.165	-0.7	1.6	-46.3	0.096	2112	876	413	0.95	1.02	0.88	XRAY	3.6	T. thermophilus HB8		Washington, et al. (2014) *ACS Chem Biol*[17]
4P70	YA,XA	1.1	0.4	-97.2	0.674	-0.5	1.6	-18.6	0.143	2096	869	410	0.97	0.99	0.87	XRAY	3.7	T. thermophilus HB8		Maehigashi, et al. (2014) *PNAS*[2]
4P70	RA,QA	-0.2	0.6	-43.8	0.237	-0.1	0.9	-21.9	0.196	2123	886	420	0.95	0.91	0.90	XRAY	3.7	T. thermophilus HB8		Maehigashi, et al. (2014) *PNAS*[2]
4TUA	RA,QA	-0.1	0.6	-35.0	0.342	-0.1	0.9	-25.9	0.292	2107	887	416	0.96	0.92	0.89	XRAY	3.6	T. thermophilus HB8		Fagan, et al. (2014) *RNA*[18]
4TUA	YA,XA	1.1	0.3	-87.3	0.652	-0.4	1.5	-20.8	0.132	2089	870	412	0.97	1.01	0.86	XRAY	3.6	T. thermophilus HB8		Fagan, et al. (2014) *RNA*[18]
4TUB	RA,QA	0.0	0.5	-47.4	0.257	-0.1	1.1	-30.4	0.186	2125	885	420	0.95	0.96	0.90	XRAY	3.6	T. thermophilus HB8		Fagan, et al. (2014) *RNA*[18]
4TUB	YA,XA	1.1	0.3	-108.5	0.635	-0.3	1.6	-15.6	0.092	2093	867	411	0.98	1.02	0.85	XRAY	3.6	T. thermophilus HB8		Fagan, et al. (2014) *RNA*[18]
4TUC	RA,QA	-0.2	0.7	-35.3	0.148	0.0	0.8	-38.2	0.309	2104	885	417	0.98	0.92	0.90	XRAY	3.6	T. thermophilus HB8		Fagan, et al. (2014) *RNA*[18]
4TUC	YA,XA	1.0	0.3	-121.2	0.410	-0.6	1.2	-22.9	0.309	2092	888	412	0.97	0.94	0.86	XRAY	3.6	T. thermophilus HB8		Fagan, et al. (2014) *RNA*[18]
4TUD	RA,QA	0.1	0.5	-28.0	0.474	-0.5	1.2	-45.9	0.364	2116	879	417	0.97	0.99	0.90	XRAY	3.6	T. thermophilus HB8		Fagan, et al. (2014) *RNA*[18]
4TUD	YA,XA	1.4	0.1	-46.8	0.895	-1.0	1.5	-25.7	0.347	2098	851	411	0.98	1.03	0.88	XRAY	3.6	T. thermophilus HB8		Fagan, et al. (2014) *RNA*[18]
4TUE	RA,QA	0.1	0.5	-39.0	0.352	-0.4	1.4	-42.2	0.302	2125	879	416	0.96	0.98	0.89	XRAY	3.5	T. thermophilus HB8		Fagan, et al. (2014) *RNA*[18]
4TUE	YA,XA	1.5	0.2	-140.1	0.107	-0.9	1.5	-24.7	0.272	2117	858	413	0.96	1.03	0.89	XRAY	3.5	T. thermophilus HB8		Fagan, et al. (2014) *RNA*[18]
4U1U	BA,AA	5.6	1.2	118.0	1.530	9.3	1.8	46.2	0.628	2643	922	377	0.76	1.15	0.76	XRAY	3.0	E. coli str. K-12 substr. MDS42		Noeske, et al. (2014) *AAC*[19]
4U1U	DA,CA	-0.6	0.9	34.9	1.387	5.5	2.3	61.5	0.750	2860	1060	412	0.58	0.61	0.66	XRAY	3.0	E. coli str. K-12 substr. MDS42		Noeske, et al. (2014) *AAC*[19]
4U1V	BA,AA	5.9	1.1	114.2	1.165	8.9	1.8	42.8	0.591	2650	948	387	0.61	1.11	0.79	XRAY	3.0	E. coli str. K-12 substr. MDS42		Noeske, et al. (2014) *AAC*[19]
4U1V	DA,CA	-0.4	0.7	49.7	0.473	5.5	2.5	67.9	0.677	2850	1060	421	0.64	0.63	0.78	XRAY	3.0	E. coli str. K-12 substr. MDS42		Noeske, et al. (2014) *AAC*[19]
4U20	BA,AA	5.7	1.2	117.7	1.302	9.4	1.8	45.4	0.440	2660	926	373	0.61	1.12	0.71	XRAY	2.9	E. coli str. K-12 substr. MDS42		Noeske, et al. (2014) *AAC*[19]
4U20	DA,CA	-0.4	0.7	39.7	0.512	5.1	2.4	57.8	0.684	2860	1060	449	0.59	0.58	0.87	XRAY	2.9	E. coli str. K-12 substr. MDS42		Noeske, et al. (2014) *AAC*[19]
4U24	BA,AA	5.6	1.2	108.8	1.111	9.4	1.7	44.0	0.301	2668	935	395	0.51	1.10	0.78	XRAY	2.9	E. coli str. K-12 substr. MDS42		Noeske, et al. (2014) *AAC*[19]
4U24	DA,CA	-0.4	0.9	51.5	0.273	5.6	2.4	62.1	0.568	2799	1050	435	0.66	0.59	0.90	XRAY	2.9	E. coli str. K-12 substr. MDS42		Noeske, et al. (2014) *AAC*[19]
4U25	BA,AA	5.6	1.3	107.2	1.261	9.3	1.6	47.7	0.383	2657	915	387	0.58	1.09	0.76	XRAY	2.9	E. coli str. K-12 substr. MDS42		Noeske, et al. (2014) *AAC*[19]
4U25	DA,CA	-0.7	1.0	37.1	0.594	5.6	2.3	60.5	0.687	2839	1052	429	0.67	0.64	0.83	XRAY	2.9	E. coli str. K-12 substr. MDS42		Noeske, et al. (2014) *AAC*[19]
4U26	BA,AA	5.6	1.2	120.6	1.219	9.6	1.7	42.4	0.205	2675	925	390	0.55	1.11	0.75	XRAY	2.8	E. coli str. K-12 substr. MDS42		Noeske, et al. (2014) *AAC*[19]
4U26	DA,CA	-0.6	0.8	44.9	0.246	5.5	2.4	60.0	0.707	2801	1050	437	0.70	0.61	0.90	XRAY	2.8	E. coli str. K-12 substr. MDS42		Noeske, et al. (2014) *AAC*[19]
4U27	BA,AA	5.7	1.2	113.4	1.241	9.2	1.8	43.6	0.390	2665	922	389	0.63	1.12	0.77	XRAY	2.8	E. coli str. K-12 substr. MDS42		Noeske, et al. (2014) *AAC*[19]
4U27	DA,CA	-0.5	0.8	41.5	0.587	5.5	2.3	62.2	0.777	2840	1052	432	0.70	0.65	0.84	XRAY	2.8	E. coli str. K-12 substr. MDS42		Noeske, et al. (2014) *AAC*[19]
4U3M	1,2	4.3	2.8	88.2	0.866	14.0	8.7	63.6	0.265	1360	589	256	1.25	1.17	1.11	XRAY	3.0	S. cerevisiae S288C		Garreau de Loubresse, et al. (2014) *Nature*[20]
4U3M	5,6	7.7	4.8	53.9	0.461	10.1	2.9	31.5	0.372	1359	589	263	1.26	1.20	1.13	XRAY	3.0	S. cerevisiae S288C		Garreau de Loubresse, et al. (2014) *Nature*[20]
4U3N	1,2	4.2	2.9	88.4	0.842	14.0	8.7	63.9	0.433	1381	594	256	1.24	1.17	1.10	XRAY	3.2	S. cerevisiae S288C		Garreau de Loubresse, et al. (2014) *Nature*[20]
4U3N	5,6	7.8	4.9	54.0	0.407	9.9	3.0	34.0	0.638	1388	588	268	1.26	1.19	1.14	XRAY	3.2	S. cerevisiae S288C		Garreau de Loubresse, et al. (2014) *Nature*[20]
4U3U	1,2	4.3	2.9	88.9	0.846	14.1	8.6	63.7	0.320	1373	599	259	1.25	1.17	1.12	XRAY	2.9	S. cerevisiae S288C		Garreau de Loubresse, et al. (2014) *Nature*[20]
4U3U	5,6	7.7	4.8	53.3	0.436	10.0	3.0	32.8	0.687	1373	589	268	1.26	1.19	1.14	XRAY	2.9	S. cerevisiae S288C		Garreau de Loubresse, et al. (2014) *Nature*[20]
4U4N	1,2	4.3	2.8	90.5	0.991	13.8	8.7	62.7	0.446	1399	590	260	1.24	1.15	1.11	XRAY	3.1	S. cerevisiae S288C		Garreau de Loubresse, et al. (2014) *Nature*[20]
4U4N	5,6	7.7	4.8	54.0	0.627	10.3	3.2	38.3	0.577	1403	594	269	1.25	1.19	1.14	XRAY	3.1	S. cerevisiae S288C		Garreau de Loubresse, et al. (2014) *Nature*[20]
4U4O	1,2	3.8	3.1	84.9	1.016	14.0	8.1	65.9	0.529	1394	597	256	1.25	1.17	1.16	XRAY	3.6	S. cerevisiae S288C		Garreau de Loubresse, et al. (2014) *Nature*[20]
4U4O	5,6	7.7	5.0	52.3	0.584	10.5	4.0	53.0	0.515	1401	595	268	1.26	1.20	1.15	XRAY	3.6	S. cerevisiae S288C		Garreau de Loubresse, et al. (2014) *Nature*[20]
4U4Q	1,2	4.3	2.9	87.9	0.983	13.7	8.7	64.2	0.355	1383	594	262	1.25	1.16	1.12	XRAY	3.0	S. cerevisiae S288C		Garreau de Loubresse, et al. (2014) *Nature*[20]
4U4Q	5,6	7.7	4.9	54.0	0.528	10.3	3.3	40.0	0.472	1391	594	265	1.26	1.18	1.14	XRAY	3.0	S. cerevisiae S288C		Garreau de Loubresse, et al. (2014) *Nature*[20]
4U4R	1,2	4.4	3.0	88.7	0.900	14.0	8.8	63.8	0.380	1370	600	261	1.25	1.17	1.13	XRAY	2.8	S. cerevisiae S288C		Garreau de Loubresse, et al. (2014) *Nature*[20]
4U4R	5,6	7.8	4.9	53.7	0.420	9.9	2.8	30.7	0.633	1385	593	267	1.26	1.19	1.14	XRAY	2.8	S. cerevisiae S288C		Garreau de Loubresse, et al. (2014) *Nature*[20]
4U4U	1,2	4.3	2.8	88.7	0.919	13.8	8.7	64.0	0.338	1397	589	261	1.25	1.16	1.11	XRAY	3.0	S. cerevisiae S288C		Garreau de Loubresse, et al. (2014) *Nature*[20]
4U4U	5,6	7.8	4.8	53.7	0.568	10.3	3.2	38.5	0.349	1380	600	264	1.25	1.20	1.13	XRAY	3.0	S. cerevisiae S288C		Garreau de Loubresse, et al. (2014) *Nature*[20]
4U4Y	1,2	4.2	2.7	87.7	0.772	13.9	8.7	63.2	0.290	1342	592	259	1.26	1.17	1.12	XRAY	3.2	S. cerevisiae S288C		Garreau de Loubresse, et al. (2014) *Nature*[20]
4U4Y	5,6	7.7	4.7	52.7	0.446	10.3	3.2	38.9	0.478	1331	588	264	1.27	1.20	1.13	XRAY	3.2	S. cerevisiae S288C		Garreau de Loubresse, et al. (2014) *Nature*[20]
4U4Z	1,2	4.3	2.9	87.3	1.109	13.7	8.7	63.9	0.408	1406	595	262	1.24	1.16	1.12	XRAY	3.1	S. cerevisiae S288C		Garreau de Loubresse, et al. (2014) *Nature*[20]
4U4Z	5,6	7.7	4.8	53.7	0.620	10.3	3.1	36.3	0.672	1406	589	265	1.24	1.18	1.13	XRAY	3.1	S. cerevisiae S288C		Garreau de Loubresse, et al. (2014) *Nature*[20]
4U50	1,2	4.1	2.9	86.4	1.068	13.8	8.5	64.7	0.592	1405	594	263	1.23	1.14	1.11	XRAY	3.2	S. cerevisiae S288C		Garreau de Loubresse, et al. (2014) *Nature*[20]
4U50	5,6	7.6	4.9	54.5	0.664	10.9	3.7	51.3	0.714	1413	595	260	1.24	1.17	1.14	XRAY	3.2	S. cerevisiae S288C		Garreau de Loubresse, et al. (2014) *Nature*[20]

PDB		BODY				HEAD				PRUNED			RMSD			EXP DETAILS		ORGANISM		REFERENCE
ID	chains	ϕ_{body}	θ_{body}	ψ_{body}	$\lvert\Delta\vec{x}_{\text{body}}\rvert$	ϕ_{head}	θ_{head}	ψ_{head}	$\lvert\Delta\vec{x}_{\text{head}}\rvert$	LSU	body	head	LSU	body	head	method	res.	name	mito	
4U51	1,2	4.2	2.8	88.3	0.921	13.9	8.6	63.9	0.430	1394	590	261	1.26	1.16	1.12	XRAY	3.2	S. cerevisiae S288C		Garreau de Loubresse, et al. (2014) *Nature*[20]
4U51	5,6	7.7	4.8	54.7	0.501	10.6	3.4	45.1	0.474	1383	589	261	1.25	1.19	1.14	XRAY	3.2	S. cerevisiae S288C		Garreau de Loubresse, et al. (2014) *Nature*[20]
4U52	1,2	4.4	3.0	88.8	0.943	13.8	8.8	63.9	0.532	1402	596	260	1.25	1.16	1.12	XRAY	3.0	S. cerevisiae S288C		Garreau de Loubresse, et al. (2014) *Nature*[20]
4U52	5,6	7.8	4.9	55.5	0.508	9.9	3.0	32.6	0.537	1386	594	262	1.24	1.17	1.12	XRAY	3.0	S. cerevisiae S288C		Garreau de Loubresse, et al. (2014) *Nature*[20]
4U53	1,2	4.1	2.8	85.8	0.945	13.9	8.5	64.8	0.406	1406	590	263	1.25	1.15	1.12	XRAY	3.3	S. cerevisiae S288C		Garreau de Loubresse, et al. (2014) *Nature*[20]
4U53	5,6	7.6	4.9	54.0	0.574	10.9	3.5	47.3	0.514	1401	596	260	1.25	1.18	1.11	XRAY	3.3	S. cerevisiae S288C		Garreau de Loubresse, et al. (2014) *Nature*[20]
4U55	1,2	4.3	2.9	87.9	1.032	13.8	8.7	63.6	0.525	1408	592	260	1.26	1.16	1.11	XRAY	3.2	S. cerevisiae S288C		Garreau de Loubresse, et al. (2014) *Nature*[20]
4U55	5,6	7.7	4.8	54.5	0.562	10.4	3.2	41.6	0.510	1390	592	266	1.25	1.19	1.14	XRAY	3.2	S. cerevisiae S288C		Garreau de Loubresse, et al. (2014) *Nature*[20]
4U56	1,2	4.3	3.0	88.8	1.019	13.8	8.7	64.6	0.365	1372	598	258	1.26	1.19	1.14	XRAY	3.5	S. cerevisiae S288C		Garreau de Loubresse, et al. (2014) *Nature*[20]
4U56	5,6	7.8	4.9	53.6	0.615	10.2	2.9	38.7	0.344	1368	599	264	1.27	1.22	1.15	XRAY	3.5	S. cerevisiae S288C		Garreau de Loubresse, et al. (2014) *Nature*[20]
4U6F	1,2	4.3	3.0	89.1	0.966	14.0	8.7	63.8	0.320	1372	599	261	1.24	1.18	1.13	XRAY	3.1	S. cerevisiae S288C		Garreau de Loubresse, et al. (2014) *Nature*[20]
4U6F	5,6	7.8	4.8	54.2	0.451	9.9	2.8	31.8	0.621	1372	591	264	1.25	1.20	1.13	XRAY	3.1	S. cerevisiae S288C		Garreau de Loubresse, et al. (2014) *Nature*[20]
4UG0	L5,S2	-2.1	2.9	22.4	1.749	-0.3	1.3	-73.1	1.443	1268	568	252	1.28	1.28	1.22	EM	3.6	Homo sapiens		Khatter, et al. (2015) *Nature*[21]
4UJC	A2,C1	-2.1	3.6	23.6	2.451	-0.4	0.9	-44.4	1.776	1155	535	264	1.35	1.30	1.26	EM	9.5	Oryctolagus cuniculus		Yamamoto, et al. (2014) *NSMB*[22]
4UJD	A2,C1	4.1	5.2	55.0	3.141	-0.4	0.9	-44.9	1.785	1160	533	264	1.35	1.30	1.26	EM	8.9	Oryctolagus cuniculus		Yamamoto, et al. (2014) *NSMB*[22]
4UJE	A2,B1	-1.8	2.8	26.4	1.161	-0.4	0.9	-44.9	1.785	1155	533	264	1.35	1.30	1.26	EM	6.9	Oryctolagus cuniculus		Budkevich, et al. (2014) *Cell*[23]
4V3P	L1,S1	-1.6	2.4	18.1	1.903	-0.7	1.0	-108.1	0.718	1380	567	242	1.21	1.25	1.27	EM	34.0	Triticum aestivum		Myasnikov, et al. (2014) *NComm*[24]
4V42	BA,AA	-1.5	0.8	22.1	0.955	1.1	0.3	-116.2	0.746	1155	597	212	1.37	1.39	1.35	XRAY	5.5	T. thermophilus		Yusupov, et al. (2001) *Science*[25]
4V47	A0,BA	2.5	1.0	139.2	0.621	1.4	5.9	-39.0	4.301	261	149	128	1.33	1.39	1.18	EM	12.3	E. coli		Gao, et al. (2003) *Cell*[26]
4V48	A0,BA	-0.5	0.9	22.0	3.849	-0.5	1.2	-60.7	3.624	561	317	175	1.50	1.44	1.27	EM	11.5	E. coli		Gao, et al. (2003) *Cell*[26]
4V49	B0,AA	-1.8	0.9	11.4	0.410	0.8	0.6	-175.2	0.785	1872	831	397	1.25	1.12	0.92	XRAY	8.7	E. coli		Vila-Sanjurjo, et al. (2003) *PNAS*[27]
4V4A	B0,AA	-1.3	1.2	4.3	0.763	0.4	0.9	-154.4	0.920	1860	846	401	1.25	1.00	0.97	XRAY	9.5	E. coli		Vila-Sanjurjo, et al. (2003) *PNAS*[27]
4V4B	B3,AA	5.9	2.6	-167.9	1.790	14.8	7.6	44.7	1.744	1265	379	295	1.17	0.88	0.84	EM	11.7	S. cerevisiae		Spahn, et al. (2004) *EMBO J*[28]
4V4G	BB,AA	-1.9	1.0	17.8	0.762	2.4	0.4	76.5	2.213	1225	589	329	1.43	1.38	1.32	XRAY	11.5	E. coli		Vila-Sanjurjo, et al. (2004) *NSMB*[29]
4V4G	DB,CA	-1.9	1.4	1.7	1.733	2.3	1.0	121.7	1.815	1263	562	330	1.43	1.39	1.32	XRAY	11.5	E. coli		Vila-Sanjurjo, et al. (2004) *NSMB*[29]
4V4G	FB,EA	-1.2	1.0	20.4	1.468	1.2	1.3	144.0	0.449	1245	495	330	1.42	1.41	1.32	XRAY	11.5	E. coli		Vila-Sanjurjo, et al. (2004) *NSMB*[29]
4V4G	HB,GA	-2.0	0.9	66.4	0.743	1.7	0.9	-115.9	1.288	1268	571	329	1.42	1.39	1.32	XRAY	11.5	E. coli		Vila-Sanjurjo, et al. (2004) *NSMB*[29]
4V4G	JB,IA	-1.1	1.2	48.9	0.780	0.7	1.0	178.4	0.802	1271	462	330	1.43	1.40	1.32	XRAY	11.5	E. coli		Vila-Sanjurjo, et al. (2004) *NSMB*[29]
4V4H	BB,AA	-2.5	1.6	25.0	1.194	6.0	2.1	142.8	0.593	2702	981	438	0.67	0.94	0.85	XRAY	3.5	E. coli		Schuwirth, et al. (2006) *NSMB*[30]
4V4H	DB,CA	-1.4	0.1	-105.3	0.570	14.9	0.1	-104.7	0.128	2644	987	394	0.64	0.90	0.84	XRAY	3.5	E. coli		Schuwirth, et al. (2006) *NSMB*[30]
4V4I	w,y	-0.5	0.4	-8.0	0.425	-0.9	0.7	130.0	0.500	1705	701	304	1.27	1.30	1.33	XRAY	3.7	T. thermophilus HB27		Korostelev, et al. (2006) *Cell*[31]
4V4J	w,y	-0.5	0.5	-8.7	0.433	-1.0	0.3	147.7	0.652	1845	741	314	1.22	1.23	1.28	XRAY	3.8	T. thermophilus HB27		Korostelev, et al. (2007) *PNAS*[32]
4V4N	A1,B2	0.7	2.5	43.9	1.127	-2.4	1.2	-59.9	1.225	1266	564	303	1.29	1.28	1.27	EM	9.0	Methanocaldococcus jannaschii		Park, et al. (2013) *Nature*[33]
4V4P	AA,BA	-1.4	0.8	-12.4	1.073	1.1	0.7	178.2	0.757	2050	840	396	1.10	1.19	1.05	XRAY	5.5	T. thermophilus HB8		Jenner, et al. (2005) *Science*[34]
4V4Q	BB,AA	-2.4	1.6	24.5	1.199	6.0	2.1	142.5	0.595	2701	981	439	0.67	0.93	0.85	XRAY	3.5	E. coli		Schuwirth, et al. (2005) *Science*[35]
4V4Q	DB,CA	-1.3	0.1	-104.6	0.571	14.9	0.0	ND	0.104	2636	988	389	0.63	0.89	0.82	XRAY	3.5	E. coli		Schuwirth, et al. (2005) *Science*[35]
4V4R	BA,AA	-0.3	0.6	-9.1	0.864	0.7	0.3	-4.6	0.745	2027	851	378	1.14	1.14	1.14	XRAY	5.9	T. thermophilus HB8		Petry, et al. (2005) *Cell*[36]
4V4S	BA,AA	-0.5	0.8	-5.5	1.112	0.6	0.2	-29.5	0.845	2021	821	366	1.16	1.16	1.13	XRAY	6.8	T. thermophilus HB8		Petry, et al. (2005) *Cell*[36]
4V4T	BA,AA	-0.2	0.9	-6.4	0.650	0.3	0.3	81.3	0.753	2049	856	377	1.13	1.12	1.13	XRAY	6.5	T. thermophilus HB8		Petry, et al. (2005) *Cell*[36]
4V4V	B0,AA	0.9	0.9	7.5	2.190	-0.5	0.8	-14.9	3.273	394	273	134	1.45	1.45	1.29	EM	15.0	E. coli		Mitra, et al. (2006) *Mol Cell*[37]
4V4W	B0,AA	1.5	0.9	-28.6	1.828	-2.6	2.4	-38.4	0.878	554	378	157	1.48	1.29	1.26	EM	15.0	E. coli		Mitra, et al. (2006) *Mol Cell*[37]
4V4X	BA,AA	-1.4	1.0	-8.4	1.259	1.1	0.7	176.5	0.703	2039	830	386	1.12	1.20	1.08	XRAY	5.0	T. thermophilus HB8		Yusupova, et al. (2006) *Nature*[38]
4V4Y	BA,AA	-1.2	1.0	-9.1	1.154	0.9	0.7	167.4	0.796	2017	831	380	1.14	1.16	1.10	XRAY	5.5	T. thermophilus HB8		Yusupova, et al. (2006) *Nature*[38]
4V4Z	BA,AA	-1.5	0.9	-12.5	1.181	1.2	0.8	-179.7	0.703	2066	833	386	1.08	1.21	1.07	XRAY	4.5	T. thermophilus HB8		Yusupova, et al. (2006) *Nature*[38]
4V50	BB,AA	-1.9	1.3	18.3	1.025	2.8	1.0	-95.3	0.874	2726	982	440	0.67	0.79	0.83	XRAY	3.2	E. coli		Berk, et al. (2006) *PNAS*[39]
4V50	DB,CA	-1.2	1.2	26.2	1.047	2.8	1.0	-93.7	0.862	2667	982	440	0.65	0.79	0.83	XRAY	3.2	E. coli		Berk, et al. (2006) *PNAS*[39]
4V51	BA,AA	0.9	0.8	-3.6	0.529	-1.1	1.1	-61.2	0.542	2099	861	384	0.99	0.96	0.99	XRAY	2.8	T. thermophilus HB8		Selmer, et al. (2006) *Science*[40]
4V51	DA,CA	1.9	0.5	29.8	0.524	-1.6	1.2	-52.0	0.604	2100	855	383	0.99	1.01	0.99	XRAY	2.8	T. thermophilus HB8		Selmer, et al. (2006) *Science*[40]
4V52	BB,AA	-2.6	1.7	23.5	1.173	6.2	2.0	144.8	0.730	2705	977	417	0.65	0.94	0.80	XRAY	3.2	E. coli		Borovinskaya, et al. (2007) *NSMB*[41]
4V52	DB,CA	-1.5	0.1	-77.5	0.436	14.7	0.1	-159.3	0.268	2659	981	413	0.65	0.92	0.80	XRAY	3.2	E. coli		Borovinskaya, et al. (2007) *NSMB*[41]
4V53	BB,AA	-2.4	1.7	21.5	1.135	6.2	1.7	143.0	0.656	2702	986	417	0.65	0.92	0.81	XRAY	3.5	E. coli		Borovinskaya, et al. (2007) *NSMB*[41]
4V53	DB,CA	-1.3	0.2	-58.8	0.452	14.6	0.1	-136.9	0.244	2639	987	412	0.64	0.89	0.81	XRAY	3.5	E. coli		Borovinskaya, et al. (2007) *NSMB*[41]

PDB		BODY				HEAD				PRUNED			RMSD			EXP DETAILS		ORGANISM		REFERENCE
ID	chains	ϕ_{body}	θ_{body}	ψ_{body}	$\lvert\Delta\vec{x}_{\text{body}}\rvert$	ϕ_{head}	θ_{head}	ψ_{head}	$\lvert\Delta\vec{x}_{\text{head}}\rvert$	LSU	body	head	LSU	body	head	method	res.	name	mito	
4V54	BB,AA	-2.6	1.6	26.1	1.052	6.2	2.0	143.1	0.480	2678	984	416	0.58	0.92	0.78	XRAY	3.3	E. coli		Borovinskaya, et al. (2007) *NSMB*[41]
4V54	DB,CA	-1.3	0.6	-149.5	0.581	14.6	0.4	11.0	0.178	2648	987	412	0.59	0.89	0.79	XRAY	3.3	E. coli		Borovinskaya, et al. (2007) *NSMB*[41]
4V55	BB,AA	-2.1	1.5	21.9	0.958	6.1	1.4	133.9	0.562	2697	993	416	0.62	0.91	0.80	XRAY	4.0	E. coli		Borovinskaya, et al. (2007) *NSMB*[41]
4V55	DB,CA	-1.0	0.4	-138.5	0.566	14.5	0.4	2.0	0.105	2642	994	411	0.62	0.88	0.81	XRAY	4.0	E. coli		Borovinskaya, et al. (2007) *NSMB*[41]
4V56	BB,AA	-2.5	1.4	22.7	1.096	5.9	1.6	132.1	0.922	2707	982	435	0.66	0.95	0.87	XRAY	3.9	E. coli		Borovinskaya, et al. (2007) *ACS Chem Biol*[42]
4V56	DB,CA	-1.3	0.3	-109.8	0.665	14.2	0.9	-81.0	0.548	2646	985	397	0.66	0.93	0.84	XRAY	3.9	E. coli		Borovinskaya, et al. (2007) *ACS Chem Biol*[42]
4V57	BB,AA	-2.4	1.4	19.4	1.148	6.1	1.7	132.8	0.929	2710	984	430	0.64	0.95	0.84	XRAY	3.5	E. coli		Borovinskaya, et al. (2007) *ACS Chem Biol*[42]
4V57	DB,CA	-1.3	0.3	-99.6	0.708	14.1	0.9	-88.5	0.602	2644	986	402	0.63	0.93	0.83	XRAY	3.5	E. coli		Borovinskaya, et al. (2007) *ACS Chem Biol*[42]
4V5A	BA,AA	1.6	0.3	3.6	0.837	-0.3	1.5	-44.3	0.498	2103	861	396	1.02	0.99	0.97	XRAY	3.5	T. thermophilus HB8		Weixlbaumer, et al. (2007) *NSMB*[43]
4V5A	DA,CA	2.2	0.2	38.3	0.589	-0.2	1.7	-35.9	0.381	2101	859	397	1.02	1.01	0.98	XRAY	3.5	T. thermophilus HB8		Weixlbaumer, et al. (2007) *NSMB*[43]
4V5B	AB,BA	-2.3	1.7	16.5	0.689	5.0	1.9	136.1	0.809	2698	975	420	0.75	1.01	1.02	XRAY	3.7	E. coli		Bingel-Erlenmeyer, et al. (2008) *Nature*[44]
4V5B	CB,DA	-1.2	0.5	-27.7	0.363	14.3	0.3	162.1	0.561	2637	980	380	0.75	0.92	1.02	XRAY	3.7	E. coli		Bingel-Erlenmeyer, et al. (2008) *Nature*[44]
4V5C	BA,AA	1.4	0.4	-21.7	0.685	-0.9	1.5	-43.3	0.375	2115	863	401	0.98	0.96	0.91	XRAY	3.3	T. thermophilus HB8		Voorhees, et al. (2009) *NSMB*[45]
4V5C	DA,CA	2.1	0.1	24.0	0.362	-0.9	1.8	-30.9	0.115	2116	859	407	0.98	1.00	0.93	XRAY	3.3	T. thermophilus HB8		Voorhees, et al. (2009) *NSMB*[45]
4V5D	BA,AA	1.6	0.6	22.3	0.410	-1.1	1.0	-44.3	0.187	2120	870	410	0.94	0.99	0.91	XRAY	3.5	T. thermophilus HB8		Voorhees, et al. (2009) *NSMB*[45]
4V5D	DA,CA	0.3	1.0	1.5	0.348	-0.4	0.9	-53.4	0.145	2116	882	410	0.94	0.93	0.91	XRAY	3.5	T. thermophilus HB8		Voorhees, et al. (2009) *NSMB*[45]
4V5E	BA,AA	-0.6	0.2	-37.0	0.617	0.9	0.9	-38.5	0.446	2139	897	413	0.96	0.94	0.93	XRAY	3.5	T. thermophilus HB8		Weixlbaumer, et al. (2008) *Science*[46]
4V5E	DA,CA	0.8	0.3	-154.1	0.634	0.6	1.2	0.2	0.383	2143	902	410	0.97	0.93	0.92	XRAY	3.5	T. thermophilus HB8		Weixlbaumer, et al. (2008) *Science*[46]
4V5F	BA,AA	0.6	0.5	-172.2	0.396	0.7	1.7	-35.1	0.669	2095	888	409	1.04	1.02	0.88	XRAY	3.6	T. thermophilus HB8		Gao, et al. (2009) *Science*[47]
4V5F	DA,CA	0.6	0.2	-75.5	1.130	0.4	1.7	-26.3	0.585	2092	886	410	1.03	1.02	0.88	XRAY	3.6	T. thermophilus HB8		Gao, et al. (2009) *Science*[47]
4V5G	BA,AA	0.6	0.4	-31.4	0.822	-0.6	1.0	-37.4	0.066	2071	865	408	1.01	1.00	0.92	XRAY	3.6	T. thermophilus HB8		Schmeing, et al. (2009) *Science*[48]
4V5G	DA,CA	0.6	0.6	-14.5	0.662	-0.9	1.1	-19.4	0.191	2069	866	411	1.00	1.00	0.93	XRAY	3.6	T. thermophilus HB8		Schmeing, et al. (2009) *Science*[48]
4V5H	BB,AA	-1.1	1.3	14.9	1.537	0.8	1.5	-104.2	1.526	2507	920	413	1.07	1.12	1.01	EM	5.8	E. coli		Seidelt, et al. (2009) *Science*[49]
4V5J	BA,AA	-0.4	0.8	-8.2	0.785	1.1	1.3	-41.1	0.584	2128	888	405	0.95	1.00	0.94	XRAY	3.1	T. thermophilus HB8		Jin, et al. (2010) *PNAS*[50]
4V5J	DA,CA	0.6	0.4	-0.8	0.619	0.3	1.5	-45.5	0.479	2130	895	405	0.95	0.93	0.94	XRAY	3.1	T. thermophilus HB8		Jin, et al. (2010) *PNAS*[50]
4V5K	BA,AA	-0.8	0.4	-30.4	0.355	1.1	1.5	-59.8	0.618	2126	888	401	0.92	0.99	0.97	XRAY	3.2	T. thermophilus HB8		Ng, et al. (2010) *NSMB*[51]
4V5K	DA,CA	0.2	0.4	-115.3	0.673	0.4	1.5	-25.3	0.691	2126	896	402	0.92	0.93	0.98	XRAY	3.2	T. thermophilus HB8		Ng, et al. (2010) *NSMB*[51]
4V5L	BA,AA	0.8	0.8	-2.4	1.027	-0.9	1.1	-31.2	0.300	2094	861	399	0.98	1.01	0.84	XRAY	3.1	T. thermophilus HB8		Voorhees, et al. (2010) *Science*[52]
4V5M	BA,AA	6.8	2.4	35.5	1.962	3.8	2.2	-31.5	1.492	2071	844	403	1.08	1.18	0.92	EM	7.8	T. thermophilus HB8		Ratje, et al. (2010) *Nature*[53]
4V5N	BA,AA	3.5	1.4	67.4	1.773	18.1	4.7	25.1	2.578	2062	829	365	1.04	1.12	0.98	EM	7.6	T. thermophilus HB8		Ratje, et al. (2010) *Nature*[53]
4V5P	BA,AA	0.2	0.2	-45.1	0.377	0.3	0.7	-30.6	0.177	2144	903	415	0.96	0.88	0.84	XRAY	3.1	T. thermophilus HB8		Schmeing, et al. (2011) *NSMB*[54]
4V5P	DA,CA	0.3	0.8	-18.3	0.343	-0.3	0.7	-22.8	0.210	2148	901	414	0.96	0.88	0.83	XRAY	3.1	T. thermophilus HB8		Schmeing, et al. (2011) *NSMB*[54]
4V5Q	BA,AA	0.5	0.2	-43.0	0.439	0.0	0.7	-27.2	0.263	2159	902	414	0.97	0.91	0.85	XRAY	3.1	T. thermophilus HB8		Schmeing, et al. (2011) *NSMB*[54]
4V5Q	DA,CA	0.6	0.6	-16.3	0.378	-0.6	0.8	-24.4	0.181	2152	897	413	0.96	0.94	0.84	XRAY	3.1	T. thermophilus HB8		Schmeing, et al. (2011) *NSMB*[54]
4V5R	BA,AA	0.6	0.6	-23.3	0.327	-0.6	0.9	-23.1	0.225	2154	894	416	0.97	0.92	0.84	XRAY	3.1	T. thermophilus HB8		Schmeing, et al. (2011) *NSMB*[54]
4V5R	DA,CA	0.5	0.2	-42.3	0.437	-0.2	0.7	-25.0	0.264	2153	895	417	0.97	0.91	0.85	XRAY	3.1	T. thermophilus HB8		Schmeing, et al. (2011) *NSMB*[54]
4V5S	BA,AA	0.6	0.2	-28.2	0.672	-0.2	0.7	-17.5	0.444	2137	891	419	0.99	0.92	0.87	XRAY	3.1	T. thermophilus HB8		Schmeing, et al. (2011) *NSMB*[54]
4V5S	DA,CA	0.7	0.6	-13.4	0.623	-0.7	0.9	-20.5	0.392	2140	891	414	0.98	0.96	0.86	XRAY	3.1	T. thermophilus HB8		Schmeing, et al. (2011) *NSMB*[54]
4V5Y	BB,AA	-2.3	1.2	25.4	0.902	6.0	1.2	139.7	0.433	2704	982	420	0.63	0.92	0.81	XRAY	4.5	E. coli		Borovinskaya, et al. (2007) *NSMB*[41]
4V5Y	DB,CA	-0.9	0.5	-137.4	0.599	14.5	0.5	34.3	0.148	2655	985	414	0.63	0.90	0.81	XRAY	4.5	E. coli		Borovinskaya, et al. (2007) *NSMB*[41]
4V5Z	B0,AA	-1.4	1.3	-13.9	1.262	-1.5	5.0	-29.6	1.978	1437	733	368	1.17	0.97	0.91	EM	8.7	Canis lupus familiaris		Chandramouli, et al. (2008) *Structure*[55]
4V61	BA,AA	-0.6	2.9	-9.3	2.659	6.6	1.8	143.5	0.464	2359	946	387	1.04	0.97	0.92	EM	9.4	Spinacia oleracea		Sharma, et al. (2007) *PNAS*[56]
4V63	BA,AA	-0.6	0.7	-36.4	0.566	0.8	1.2	-51.0	0.282	2113	878	407	0.98	0.93	0.92	XRAY	3.2	T. thermophilus HB27		Laurberg, et al. (2008) *Nature*[57]
4V63	DA,CA	-0.1	0.3	-70.5	0.435	0.8	1.2	-50.8	0.279	2112	878	407	0.98	0.93	0.92	XRAY	3.2	T. thermophilus HB27		Laurberg, et al. (2008) *Nature*[57]
4V64	BB,AA	-2.4	1.5	23.4	1.127	6.0	1.7	142.5	0.512	2713	974	410	0.64	0.92	0.84	XRAY	3.5	E. coli		Borovinskaya, et al. (2008) *RNA*[58]
4V64	DB,CA	-1.3	0.1	-49.7	0.518	14.5	0.3	138.0	0.189	2647	977	402	0.63	0.89	0.83	XRAY	3.5	E. coli		Borovinskaya, et al. (2008) *RNA*[58]
4V65	BB,A1	1.0	0.4	150.3	2.460	-1.1	0.5	-40.4	2.701	406	157	91	1.52	1.42	1.47	EM	9.0	E. coli		Devkota, et al. *To be published*
4V66	BB,A1	0.7	1.1	73.6	3.457	-1.3	0.3	-119.4	1.498	404	164	90	1.50	1.40	1.49	EM	9.0	E. coli		Devkota, et al. *To be published*
4V67	BA,AA	-0.6	0.5	-40.1	0.492	0.9	1.3	-40.1	0.421	2139	889	412	0.96	0.94	0.92	XRAY	3.0	T. thermophilus		Korostelev, et al. (2008) *PNAS*[59]
4V67	DA,CA	0.1	0.3	-62.8	0.484	0.9	1.3	-39.7	0.416	2132	889	412	0.96	0.94	0.92	XRAY	3.0	T. thermophilus		Korostelev, et al. (2008) *PNAS*[59]
4V68	BA,AA	0.8	1.0	15.9	0.312	-0.8	0.6	-79.0	0.604	2104	861	384	0.99	0.96	0.99	EM	6.4	T. thermophilus		Schuette, et al. (2009) *EMBO J*[60]
4V69	BB,AA	0.8	1.0	16.1	0.804	-0.8	1.0	-54.7	0.882	2381	844	405	1.19	1.21	1.16	EM	6.7	E. coli		Villa, et al. (2009) *PNAS*[61]

PDB		BODY				HEAD				PRUNED			RMSD			EXP DETAILS		ORGANISM		REFERENCE
ID	chains	ϕ_{body}	θ_{body}	ψ_{body}	$\lvert\Delta\vec{x}_{\text{body}}\rvert$	ϕ_{head}	θ_{head}	ψ_{head}	$\lvert\Delta\vec{x}_{\text{head}}\rvert$	LSU	body	head	LSU	body	head	method	res.	name	mito	
4V6A	DA,CA	-0.7	0.6	-12.5	0.143	1.1	0.8	-74.4	0.558	2128	894	404	0.94	0.92	0.90	XRAY	3.1	T. thermophilus HB8		Blaha, et al. (2009) *Science*[62]
4V6A	BA,AA	0.4	0.3	-94.7	1.001	0.4	0.9	-30.9	0.313	2078	885	410	0.97	0.89	0.92	XRAY	3.1	T. thermophilus HB8		Blaha, et al. (2009) *Science*[62]
4V6C	BA,AA	5.7	1.3	112.1	1.305	9.6	1.7	38.0	0.119	2677	910	406	0.65	1.12	0.80	XRAY	3.2	E. coli		Zhang, et al. (2009) *Science*[63]
4V6C	DA,CA	-0.3	0.6	54.7	0.537	6.0	2.5	55.5	0.653	2715	1009	412	0.75	0.67	1.05	XRAY	3.2	E. coli		Zhang, et al. (2009) *Science*[63]
4V6D	BA,AA	5.8	1.1	98.0	1.414	-0.4	1.9	-31.5	0.403	2662	918	439	0.66	1.12	0.78	XRAY	3.8	E. coli		Zhang, et al. (2009) *Science*[63]
4V6D	DA,CA	-1.6	0.8	36.1	0.490	2.2	0.3	58.0	0.863	2709	1007	425	0.71	0.65	0.82	XRAY	3.8	E. coli		Zhang, et al. (2009) *Science*[63]
4V6E	BA,AA	6.0	1.1	107.9	1.597	-0.4	1.5	-60.6	0.469	2681	909	433	0.67	1.12	0.80	XRAY	3.7	E. coli		Zhang, et al. (2009) *Science*[63]
4V6E	DA,CA	-0.5	0.3	45.2	0.421	1.2	0.1	31.5	0.628	2694	1006	418	0.81	0.65	0.87	XRAY	3.7	E. coli		Zhang, et al. (2009) *Science*[63]
4V6F	DA,CA	1.7	0.3	-19.1	0.916	-0.8	1.2	-24.9	0.241	2098	864	409	0.94	0.95	0.86	XRAY	3.1	T. thermophilus		Jenner, et al. (2010) *NSMB*[64]
4V6F	AA,BA	0.3	0.7	-20.4	0.412	-0.3	0.5	-31.5	0.204	2113	897	410	0.93	0.90	0.88	XRAY	3.1	T. thermophilus		Jenner, et al. (2010) *NSMB*[64]
4V6G	DA,CA	1.2	0.3	-75.3	0.816	-0.6	1.3	-21.8	0.326	2090	875	423	0.99	0.96	0.95	XRAY	3.5	T. thermophilus HB8		Jenner, et al. (2010) *NSMB*[64]
4V6G	BA,AA	-0.6	0.6	-32.4	0.550	1.2	1.1	-45.7	0.492	2109	886	412	0.98	1.01	0.97	XRAY	3.5	T. thermophilus HB8		Jenner, et al. (2010) *NSMB*[64]
4V6I	DA,CA	-1.3	3.0	15.4	0.744	-0.7	0.9	-119.6	0.577	1346	540	224	1.24	1.27	1.26	EM	8.8	S. cerevisiae		Armache, et al. (2010) *PNAS*[65]
4V6K	AB,BA	1.4	1.0	30.9	1.348	-1.0	1.4	-58.5	1.003	2092	834	367	1.27	1.27	1.20	EM	8.2	E. coli K-12		Agirrezabala, et al. (2011) *EMBO J*[66]
4V6L	BB,AA	2.3	0.7	35.2	1.611	-1.4	1.8	-27.2	1.309	1679	613	279	1.34	1.35	1.30	EM	13.2	E. coli K-12		Agirrezabala, et al. (2011) *EMBO J*[66]
4V6M	B8,AA	-1.3	1.3	14.4	0.946	1.1	1.8	-95.7	1.101	2541	957	415	0.96	1.02	1.07	EM	7.1	E. coli DH1		Frauenfeld, et al. (2011) *NSMB*[67]
4V6N	AB,BA	2.0	0.7	43.7	1.348	1.4	1.5	-25.2	0.776	1662	514	266	1.34	1.36	1.38	EM	12.1	E. coli		Agirrezabala, et al. (2012) *PNAS*[68]
4V6O	BB,AA	3.4	0.2	56.6	1.484	1.4	3.0	5.4	0.148	1349	478	206	1.39	1.38	1.47	EM	14.7	E. coli		Agirrezabala, et al. (2012) *PNAS*[68]
4V6P	BB,AA	6.1	0.5	40.5	0.912	3.2	3.6	39.9	1.522	1215	409	135	1.40	1.40	1.43	EM	13.5	E. coli		Agirrezabala, et al. (2012) *PNAS*[68]
4V6Q	BB,AA	8.5	1.3	34.9	1.295	3.8	2.8	-23.3	0.654	1494	457	222	1.38	1.35	1.45	EM	11.5	E. coli		Agirrezabala, et al. (2012) *PNAS*[68]
4V6R	BB,AA	7.7	1.0	53.0	1.238	3.0	2.2	23.7	1.382	1489	421	171	1.36	1.38	1.37	EM	11.5	E. coli		Agirrezabala, et al. (2012) *PNAS*[68]
4V6S	AB,BA	-0.2	1.2	-21.1	2.786	2.4	2.3	-3.4	0.845	1471	518	214	1.39	1.41	1.40	EM	13.1	E. coli		Agirrezabala, et al. (2012) *PNAS*[68]
4V6T	BA,AA	6.4	1.4	53.1	0.817	16.4	12.2	-30.7	1.250	2626	985	404	0.55	0.77	0.76	EM	8.3	E. coli		Ramrath, et al. (2012) *Nature*[69]
4V6U	B1,A2	-2.1	2.1	27.7	0.920	-0.7	2.7	-88.4	1.383	1624	668	334	1.21	1.15	1.18	EM	6.6	Pyrococcus furiosus DSM 3638		Armache, et al. (2013) *NAR*[70]
4V6V	BA,AA	-1.7	1.8	16.6	0.319	1.8	2.9	-85.0	0.961	2203	865	393	1.19	1.16	1.22	EM	9.8	E. coli		Li, et al. (2013) *NComm*[71]
4V6W	A5,B2	2.8	4.3	27.9	0.799	14.5	5.8	57.2	1.654	1300	539	117	1.30	1.26	1.25	EM	6.0	Drosophila melanogaster		Anger, et al. (2013) *Nature*[72]
4V6X	A5,B2	5.7	4.7	39.3	0.835	13.1	7.1	73.0	0.442	1339	570	240	1.25	1.22	1.24	EM	5.0	Homo sapiens		Anger, et al. (2013) *Nature*[72]
4V6Y	BA,AA	3.5	0.7	-30.1	2.616	-1.5	0.9	42.5	1.018	890	389	172	1.46	1.33	1.36	EM	12.0	E. coli K-12		Bock, et al. (2013) *NSMB*[73]
4V6Z	BA,AA	0.1	1.0	9.4	0.408	-0.7	0.4	171.8	2.269	969	492	136	1.46	1.39	1.42	EM	12.0	E. coli K-12		Bock, et al. (2013) *NSMB*[73]
4V70	BA,AA	4.5	4.6	14.6	3.166	3.0	1.6	-114.0	6.981	729	367	110	1.43	1.38	1.34	EM	17.0	E. coli K-12		Bock, et al. (2013) *NSMB*[73]
4V71	BA,AA	4.7	0.1	46.0	3.724	-1.6	2.7	-18.4	2.876	490	363	177	1.41	1.38	1.43	EM	20.0	E. coli K-12		Bock, et al. (2013) *NSMB*[73]
4V72	BA,AA	9.6	3.6	39.7	2.156	0.7	0.7	-12.0	2.587	753	392	163	1.44	1.39	1.40	EM	13.0	E. coli K-12		Bock, et al. (2013) *NSMB*[73]
4V73	BA,AA	11.6	2.5	27.4	2.516	5.9	2.1	-103.6	5.389	664	351	140	1.46	1.45	1.38	EM	15.0	E. coli K-12		Bock, et al. (2013) *NSMB*[73]
4V74	BA,AA	14.6	4.0	-8.5	4.028	3.1	3.8	-47.0	3.341	489	284	161	1.45	1.42	1.38	EM	17.0	E. coli K-12		Bock, et al. (2013) *NSMB*[73]
4V75	BA,AA	0.1	2.6	-57.5	2.242	1.4	2.4	26.3	1.595	1008	326	146	1.43	1.38	1.43	EM	12.0	E. coli K-12		Bock, et al. (2013) *NSMB*[73]
4V76	BA,AA	0.2	3.2	23.8	1.908	2.6	4.9	178.3	6.864	726	442	155	1.40	1.41	1.31	EM	17.0	E. coli K-12		Bock, et al. (2013) *NSMB*[73]
4V77	BA,AA	0.1	3.1	28.0	1.421	3.1	3.6	-86.3	1.508	664	435	165	1.49	1.40	1.46	EM	17.0	E. coli K-12		Bock, et al. (2013) *NSMB*[73]
4V78	BA,AA	-1.1	1.6	-54.8	2.069	0.6	2.6	167.0	2.143	611	382	143	1.39	1.41	1.38	EM	20.0	E. coli K-12		Bock, et al. (2013) *NSMB*[73]
4V79	BA,AA	-0.7	2.0	60.2	1.416	4.1	2.2	-125.1	5.979	491	284	129	1.42	1.40	1.42	EM	15.0	E. coli K-12		Bock, et al. (2013) *NSMB*[73]
4V7A	BA,AA	1.5	3.4	-0.7	1.773	-0.1	2.2	107.9	1.035	1245	516	262	1.38	1.36	1.40	EM	9.0	E. coli K-12		Bock, et al. (2013) *NSMB*[73]
4V7B	BA,AA	3.3	1.2	44.4	0.512	16.9	4.2	4.3	1.323	2578	897	430	0.71	1.13	0.82	EM	6.8	E. coli K-12		Ramrath, et al. (2013) *PNAS*[74]
4V7C	BA,AA	9.5	1.9	19.0	0.410	2.7	2.2	-28.1	0.438	2549	888	433	1.00	1.16	1.14	EM	7.6	E. coli		Brilot, et al. (2013) *PNAS*[75]
4V7D	AA,BA	10.4	2.5	7.5	0.432	3.3	3.0	-33.2	0.483	2561	899	433	0.92	1.14	1.08	EM	7.6	E. coli		Brilot, et al. (2013) *PNAS*[75]
4V7E	Aa,Ad	-1.5	3.0	24.2	0.767	-0.4	0.8	-85.9	1.246	1345	568	274	1.23	1.28	1.26	EM	5.5	Triticum aestivum		Gogala, et al. (2014) *Nature*[76]
4V7H	B5,AA	5.7	5.6	17.9	2.751	10.6	3.8	66.1	2.396	153	358	198	1.47	1.37	1.27	EM	8.9	Thermomyces lanuginosus		Taylor, et al. (2009) *Structure*[77]
4V7I	A8,BA	-0.8	1.3	6.1	1.237	1.4	2.7	-74.2	1.493	1701	669	276	1.38	1.40	1.38	EM	9.6	E. coli		Gumbart, et al. (2009) *Structure*[78]
4V7J	AA,Aa	-0.3	0.5	-41.7	0.196	0.0	1.8	-55.5	0.421	2141	890	404	0.93	0.99	0.90	XRAY	3.3	T. thermophilus HB8		Neubauer, et al. (2009) *Cell*[79]
4V7J	BA,Ba	0.1	0.3	-87.6	0.642	-0.2	1.8	-55.1	0.557	2142	895	408	0.93	0.96	0.91	XRAY	3.3	T. thermophilus HB8		Neubauer, et al. (2009) *Cell*[79]
4V7K	AA,Aa	-0.3	0.6	-26.9	0.318	0.0	1.6	-56.2	0.347	2137	893	408	0.92	0.98	0.93	XRAY	3.6	T. thermophilus HB8		Neubauer, et al. (2009) *Cell*[79]
4V7K	BA,Ba	0.2	0.3	-61.6	0.515	-0.3	1.6	-56.0	0.476	2129	897	408	0.92	0.96	0.93	XRAY	3.6	T. thermophilus HB8		Neubauer, et al. (2009) *Cell*[79]
4V7L	BA,AA	1.6	0.4	-22.8	0.655	-0.8	1.4	-25.4	0.352	2025	839	405	1.03	1.03	0.91	XRAY	3.0	T. thermophilus HB8		Stanley, et al. (2010) *NSMB*[80]
4V7L	DA,CA	0.4	0.6	-16.8	1.169	-0.5	1.0	-27.2	0.239	2090	878	408	1.03	0.97	0.99	XRAY	3.0	T. thermophilus HB8		Stanley, et al. (2010) *NSMB*[80]

PDB		BODY				HEAD				PRUNED			RMSD			EXP DETAILS		ORGANISM		REFERENCE
ID	chains	ϕ_{body}	θ_{body}	ψ_{body}	$\lvert\Delta\vec{x}_{\text{body}}\rvert$	ϕ_{head}	θ_{head}	ψ_{head}	$\lvert\Delta\vec{x}_{\text{head}}\rvert$	LSU	body	head	LSU	body	head	method	res.	name	mito	
4V7M	BA,AA	1.6	0.3	-33.1	0.927	-0.9	1.5	-27.7	0.243	2110	870	413	0.95	0.94	0.86	XRAY	3.5	T. thermophilus HB8		Stanley, et al. (2010) *NSMB*[80]
4V7M	DA,CA	0.7	0.5	-31.6	0.626	-0.7	1.4	-34.0	0.292	2095	885	414	0.95	0.91	0.89	XRAY	3.5	T. thermophilus HB8		Stanley, et al. (2010) *NSMB*[80]
4V7P	BA,AA	-0.6	0.5	-43.3	0.443	0.7	1.6	-51.2	0.631	2132	884	415	0.99	0.98	0.98	XRAY	3.6	T. thermophilus HB27		Korostelev, et al. (2010) *EMBO J*[81]
4V7P	CA,DA	0.1	0.1	-86.9	0.654	0.7	1.6	-51.3	0.630	2133	885	415	0.99	0.98	0.98	XRAY	3.6	T. thermophilus HB27		Korostelev, et al. (2010) *EMBO J*[81]
4V7R	B1,A1	3.4	2.7	85.4	0.737	13.8	8.1	64.9	0.442	1281	585	255	1.28	1.22	1.15	XRAY	4.0	S. cerevisiae S288C		Ben-Shem, et al. (2010) *Science*[82]
4V7R	D1,C1	7.3	4.5	54.7	0.615	10.4	3.2	34.8	0.521	1283	579	254	1.28	1.22	1.15	XRAY	4.0	S. cerevisiae S288C		Ben-Shem, et al. (2010) *Science*[82]
4V7S	BA,AA	5.6	1.2	114.3	1.317	9.5	1.6	41.0	0.231	2671	923	405	0.61	1.11	0.78	XRAY	3.3	E. coli		Dunkle, et al. (2010) *PNAS*[83]
4V7S	DA,CA	-0.2	0.5	66.9	0.291	6.3	2.6	61.1	0.671	2710	1012	405	0.73	0.63	1.02	XRAY	3.3	E. coli		Dunkle, et al. (2010) *PNAS*[83]
4V7T	BA,AA	5.5	1.3	107.7	1.322	9.6	1.4	43.5	0.348	2670	928	406	0.61	1.10	0.78	XRAY	3.2	E. coli		Dunkle, et al. (2010) *PNAS*[83]
4V7T	DA,CA	-0.2	0.5	57.3	0.492	6.2	2.4	61.5	0.492	2717	1008	411	0.76	0.65	1.05	XRAY	3.2	E. coli		Dunkle, et al. (2010) *PNAS*[83]
4V7U	BA,AA	5.6	1.3	109.2	1.365	9.5	1.6	45.9	0.340	2670	930	402	0.61	1.10	0.80	XRAY	3.1	E. coli		Dunkle, et al. (2010) *PNAS*[83]
4V7U	DA,CA	-0.4	0.7	46.8	0.572	6.2	2.6	55.1	0.592	2720	1011	405	0.76	0.68	1.05	XRAY	3.1	E. coli		Dunkle, et al. (2010) *PNAS*[83]
4V7V	BA,AA	5.6	1.3	107.0	1.114	9.4	1.5	45.4	0.201	2666	930	401	0.57	1.09	0.79	XRAY	3.3	E. coli		Dunkle, et al. (2010) *PNAS*[83]
4V7V	DA,CA	-0.3	0.5	54.6	0.635	6.3	2.5	59.8	0.537	2712	1011	407	0.74	0.66	1.04	XRAY	3.3	E. coli		Dunkle, et al. (2010) *PNAS*[83]
4V7W	BA,AA	1.0	0.7	-4.4	0.325	1.2	1.7	-33.0	0.030	2135	880	400	0.94	0.88	0.95	XRAY	3.0	T. thermophilus HB8		Bulkley, et al. (2010) *PNAS*[84]
4V7W	DA,CA	-0.8	1.1	-2.0	0.329	1.8	0.6	-62.3	0.111	2130	878	401	0.93	0.88	0.95	XRAY	3.0	T. thermophilus HB8		Bulkley, et al. (2010) *PNAS*[84]
4V7X	BA,AA	1.1	0.6	-7.6	0.408	1.2	1.8	-30.3	0.210	2129	885	396	0.94	0.90	0.96	XRAY	3.0	T. thermophilus HB8		Bulkley, et al. (2010) *PNAS*[84]
4V7X	DA,CA	-0.9	1.0	-3.2	0.256	2.0	0.6	-70.9	0.411	2136	886	397	0.94	0.90	0.97	XRAY	3.0	T. thermophilus HB8		Bulkley, et al. (2010) *PNAS*[84]
4V7Y	BA,AA	0.7	0.9	4.2	0.147	1.6	1.6	-32.9	0.245	2126	883	403	0.95	0.92	1.00	XRAY	3.0	T. thermophilus HB8		Bulkley, et al. (2010) *PNAS*[84]
4V7Y	DA,CA	-0.8	1.2	0.3	0.491	1.8	0.3	-85.1	0.433	2131	884	403	0.95	0.92	1.00	XRAY	3.0	T. thermophilus HB8		Bulkley, et al. (2010) *PNAS*[84]
4V7Z	BA,AA	0.9	0.8	-7.5	2.141	1.3	1.8	-29.6	1.343	1941	859	399	1.14	1.02	0.97	XRAY	3.1	T. thermophilus HB8		Bulkley, et al. (2010) *PNAS*[84]
4V7Z	DA,CA	-0.8	1.1	0.4	1.187	1.9	0.6	-62.9	1.194	1937	863	399	1.13	1.03	0.97	XRAY	3.1	T. thermophilus HB8		Bulkley, et al. (2010) *PNAS*[84]
4V83	BA,AA	-1.0	0.9	-3.0	0.826	1.8	0.5	-29.6	0.676	2107	889	393	0.95	1.00	0.99	XRAY	3.5	T. thermophilus HB27		Zhu, et al. (2011) *PNAS*[85]
4V83	DA,CA	-0.4	0.7	5.3	0.735	2.2	1.3	-4.2	0.621	2108	891	393	0.95	1.00	0.99	XRAY	3.5	T. thermophilus HB27		Zhu, et al. (2011) *PNAS*[85]
4V84	BA,AA	-1.0	1.0	-5.5	0.371	2.0	0.8	-26.0	0.292	2120	892	401	0.92	0.92	0.96	XRAY	3.4	T. thermophilus HB27		Zhu, et al. (2011) *PNAS*[85]
4V84	DA,CA	-0.3	0.6	2.9	0.196	1.9	1.5	-14.3	0.242	2113	892	401	0.92	0.91	0.96	XRAY	3.4	T. thermophilus HB27		Zhu, et al. (2011) *PNAS*[85]
4V85	BA,AA	7.3	2.6	44.1	0.920	13.8	3.3	72.2	0.611	2595	967	383	0.75	0.94	0.87	XRAY	3.2	E. coli		Zhou, et al. (2012) *RNA*[86]
4V87	AA,BA	1.4	0.3	-25.2	0.786	-0.8	1.3	-22.5	0.187	2110	871	408	0.94	0.94	0.84	XRAY	3.1	T. thermophilus HB8		Demeshkina, et al. (2012) *Nature*[87]
4V87	DA,CA	-0.1	0.9	-26.7	0.334	-0.1	0.8	-27.5	0.117	2135	893	412	0.94	0.87	0.86	XRAY	3.1	T. thermophilus HB8		Demeshkina, et al. (2012) *Nature*[87]
4V88	A1,A2	4.1	2.8	88.4	0.820	14.0	8.7	63.4	0.340	1365	595	264	1.25	1.17	1.13	XRAY	3.0	S. cerevisiae		Ben-Shem, et al. (2011) *Science*[88]
4V88	A5,A6	7.7	4.8	52.8	0.414	9.9	3.0	33.9	0.615	1358	586	251	1.26	1.19	1.13	XRAY	3.0	S. cerevisiae		Ben-Shem, et al. (2011) *Science*[88]
4V89	BA,AA	8.6	0.9	23.1	0.799	12.8	4.5	53.6	0.646	2559	965	386	0.81	0.96	0.86	XRAY	3.7	E. coli		Zhou, et al. (2012) *RNA*[86]
4V8A	AA,DA	-0.5	1.1	-4.5	0.189	1.4	0.6	-60.1	0.069	2117	887	410	0.93	0.88	0.95	XRAY	3.2	T. thermophilus HB8		Bulkley, et al. (2012) *JMB*[89]
4V8A	BA,CA	1.0	0.8	4.3	0.740	0.9	1.6	-30.9	0.303	2099	870	414	0.96	0.89	0.94	XRAY	3.2	T. thermophilus HB8		Bulkley, et al. (2012) *JMB*[89]
4V8B	BA,AA	1.4	0.3	-22.2	0.733	-0.8	1.4	-19.0	0.197	2104	868	410	0.94	0.95	0.85	XRAY	3.0	T. thermophilus HB8		Demeshkina, et al. (2012) *Nature*[87]
4V8B	DA,CA	-0.2	0.8	-28.1	0.272	0.3	1.0	-28.8	0.124	2145	893	406	0.94	0.87	0.88	XRAY	3.0	T. thermophilus HB8		Demeshkina, et al. (2012) *Nature*[87]
4V8C	AA,CA	1.4	0.3	-35.4	0.961	-0.8	1.4	-20.3	0.180	2091	859	409	0.93	0.99	0.84	XRAY	3.3	T. thermophilus HB8		Demeshkina, et al. (2012) *Nature*[87]
4V8C	BA,DA	0.1	0.8	-32.0	0.380	-0.1	0.9	-19.0	0.070	2127	894	406	0.94	0.91	0.83	XRAY	3.3	T. thermophilus HB8		Demeshkina, et al. (2012) *Nature*[87]
4V8D	BA,AA	1.4	0.3	-14.5	0.638	-1.1	1.2	-30.3	0.370	2114	866	407	0.95	0.96	0.84	XRAY	3.0	T. thermophilus HB8		Demeshkina, et al. (2012) *Nature*[87]
4V8D	DA,CA	0.1	0.8	-26.5	0.412	-0.6	0.6	-35.0	0.420	2134	893	412	0.95	0.90	0.87	XRAY	3.0	T. thermophilus HB8		Demeshkina, et al. (2012) *Nature*[87]
4V8E	AA,BA	1.6	0.3	-23.1	0.743	-1.1	1.4	-29.0	0.277	2109	857	409	0.96	0.98	0.87	XRAY	3.3	T. thermophilus HB8		Demeshkina, et al. (2012) *Nature*[87]
4V8E	CA,DA	-0.2	0.8	-29.0	0.459	-0.2	0.9	-38.1	0.360	2137	893	405	0.97	0.91	0.89	XRAY	3.3	T. thermophilus HB8		Demeshkina, et al. (2012) *Nature*[87]
4V8F	AA,BA	1.6	0.4	-29.7	0.819	-1.2	1.4	-26.0	0.298	2115	856	408	0.94	0.98	0.85	XRAY	3.3	T. thermophilus HB8		Demeshkina, et al. (2012) *Nature*[87]
4V8F	DA,CA	-0.2	0.8	-30.2	0.428	-0.2	0.9	-44.7	0.249	2130	895	405	0.93	0.88	0.88	XRAY	3.3	T. thermophilus HB8		Demeshkina, et al. (2012) *Nature*[87]
4V8G	DA,CA	-0.8	1.1	7.7	0.543	1.5	0.7	-113.9	0.960	2139	888	386	0.94	0.95	1.04	XRAY	3.0	T. thermophilus HB8		Polikanov, et al. (2012) *Science*[90]
4V8G	BA,AA	0.8	0.7	4.7	0.459	6.7	4.9	1.1	0.391	2133	889	290	0.94	0.94	1.30	XRAY	3.0	T. thermophilus HB8		Polikanov, et al. (2012) *Science*[90]
4V8H	BA,AA	1.1	0.5	-3.0	0.573	4.3	4.0	-6.5	0.437	2113	887	381	0.92	0.91	1.15	XRAY	3.1	T. thermophilus HB8		Polikanov, et al. (2012) *Science*[90]
4V8H	DA,CA	-0.7	0.9	-10.9	0.097	3.3	1.9	-4.6	0.537	2116	887	375	0.92	0.89	1.18	XRAY	3.1	T. thermophilus HB8		Polikanov, et al. (2012) *Science*[90]
4V8I	BA,AA	1.5	0.6	4.0	0.370	-1.2	1.4	-58.9	0.727	2116	862	414	0.95	0.94	0.86	XRAY	2.7	T. thermophilus		Polikanov, et al. (2012) *Science*[90]
4V8I	DA,CA	-0.9	1.0	1.5	0.379	1.0	1.3	-82.5	0.304	2116	886	410	0.94	0.93	0.94	XRAY	2.7	T. thermophilus		Polikanov, et al. (2012) *Science*[90]
4V8J	BA,AA	0.7	0.5	-8.1	0.647	-0.8	1.5	-48.6	0.142	2115	854	411	0.99	1.01	0.91	XRAY	3.9	T. thermophilus HB8		Fagan, et al. (2013) *PNAS*[91]
4V8J	DA,CA	1.7	0.0	ND	0.297	-1.0	1.5	-35.6	0.246	2112	837	409	0.98	1.07	0.89	XRAY	3.9	T. thermophilus HB8		Fagan, et al. (2013) *PNAS*[91]

PDB		BODY				HEAD				PRUNED			RMSD			EXP DETAILS		ORGANISM		REFERENCE
ID	chains	ϕ_{body}	θ_{body}	ψ_{body}	$\lvert\Delta\vec{x}_{\mathrm{body}}\rvert$	ϕ_{head}	θ_{head}	ψ_{head}	$\lvert\Delta\vec{x}_{\mathrm{head}}\rvert$	LSU	body	head	LSU	body	head	method	res.	name	mito	
4V8N	BA,AA	1.3	0.4	1.5	0.640	-1.0	1.4	-44.1	0.545	2118	861	399	0.95	1.01	0.95	XRAY	3.1	T. thermophilus HB8		Voorhees, et al. (2013) *NSMB*[92]
4V8N	DA,CA	-0.1	1.0	5.3	0.706	-0.4	1.3	-58.0	0.472	2112	870	401	0.95	0.98	0.96	XRAY	3.1	T. thermophilus HB8		Voorhees, et al. (2013) *NSMB*[92]
4V8O	BA,AA	9.5	2.5	5.1	1.282	3.5	2.2	-32.8	0.133	2053	861	405	1.05	1.06	0.98	XRAY	3.8	T. thermophilus HB8		Jin, et al. (2011) *PNAS*[93]
4V8Q	AA,BA	0.4	0.3	-100.2	0.344	0.1	0.6	-27.3	0.385	2136	887	407	0.94	0.90	0.85	XRAY	3.1	T. thermophilus HB8		Neubauer, et al. (2012) *Science*[94]
4V8U	BA,AA	0.4	0.3	-158.4	0.320	0.6	1.3	-32.3	0.361	2119	896	414	1.00	0.95	0.88	XRAY	3.7	T. thermophilus HB8		Feng, et al. (2013) *PLoS One*[95]
4V8U	DA,CA	0.5	0.3	-40.1	0.583	0.2	1.4	-25.5	0.295	2117	892	413	0.99	0.95	0.88	XRAY	3.7	T. thermophilus HB8		Feng, et al. (2013) *PLoS One*[95]
4V8X	BA,AA	-0.8	0.5	-23.6	0.606	1.1	0.8	-48.6	0.736	2124	889	410	0.95	1.02	0.91	XRAY	3.4	T. thermophilus HB8		Feng, et al. (2013) *NAR*[96]
4V8X	DA,CA	-0.1	0.5	-140.2	0.787	0.9	0.9	-15.8	0.598	2115	891	407	0.95	0.99	0.91	XRAY	3.4	T. thermophilus HB8		Feng, et al. (2013) *NAR*[96]
4V8Y	B5,B2	5.1	3.1	41.6	0.347	14.0	8.7	63.4	0.340	1358	595	264	1.26	1.17	1.13	EM	4.3	S. cerevisiae		Fernandez, et al. (2013) *Science*[97]
4V8Z	B5,B2	5.1	3.1	41.6	0.347	14.0	8.7	63.4	0.340	1358	595	264	1.26	1.17	1.13	EM	6.6	S. cerevisiae		Fernandez, et al. (2013) *Science*[97]
4V90	BA,AA	7.0	1.5	40.7	1.001	3.6	1.4	-37.5	0.797	2132	830	406	0.97	1.07	0.97	XRAY	3.0	T. thermophilus HB8		Chen, et al. (2013) *NSMB*[98]
4V95	BA,AA	1.2	0.5	-9.6	0.277	-0.6	1.3	-42.5	0.192	2129	879	406	0.95	0.93	0.84	XRAY	3.2	T. thermophilus HB8		Gagnon, et al. (2012) *Science*[99]
4V95	DA,CA	-0.6	0.9	-9.4	0.345	1.0	1.0	-55.7	0.353	2160	888	407	0.94	0.94	0.91	XRAY	3.2	T. thermophilus HB8		Gagnon, et al. (2012) *Science*[99]
4V97	DA,CA	1.6	0.1	-14.4	0.531	-0.8	1.4	-30.1	0.549	2104	833	413	1.01	1.12	0.92	XRAY	3.5	T. thermophilus HB8		Fagan, et al. (2013) *PNAS*[91]
4V97	BA,AA	0.4	0.5	-19.0	0.580	-0.6	1.5	-50.6	0.419	2109	863	407	1.00	1.05	0.92	XRAY	3.5	T. thermophilus HB8		Fagan, et al. (2013) *PNAS*[91]
4V9A	BA,AA	1.4	0.1	-92.8	0.654	-0.7	1.5	-25.2	0.058	2116	859	409	0.97	0.96	0.85	XRAY	3.3	T. thermophilus HB8		Jenner, et al. (2013) *PNAS*[100]
4V9A	DA,CA	-0.9	0.6	-40.2	0.245	1.3	1.2	-52.1	0.337	2115	884	406	0.95	0.98	0.99	XRAY	3.3	T. thermophilus HB8		Jenner, et al. (2013) *PNAS*[100]
4V9B	BA,AA	1.5	0.2	-126.6	0.186	-1.1	1.5	-28.2	0.108	2117	875	409	0.94	0.94	0.88	XRAY	3.1	T. thermophilus HB8		Jenner, et al. (2013) *PNAS*[100]
4V9B	DA,CA	-0.8	0.6	-36.9	0.072	1.1	1.1	-55.8	0.333	2125	886	411	0.94	0.96	0.96	XRAY	3.1	T. thermophilus HB8		Jenner, et al. (2013) *PNAS*[100]
4V9C	BA,AA	2.0	1.0	-146.1	0.734	-0.6	1.5	-10.7	0.606	2771	1019	453	0.64	0.98	0.73	XRAY	3.3	E. coli K-12		Wang, et al. (2012) *NSMB*[101]
4V9C	DA,CA	7.5	0.8	-171.9	0.862	0.7	1.6	-0.3	0.819	2619	896	421	0.61	1.14	0.84	XRAY	3.3	E. coli K-12		Wang, et al. (2012) *NSMB*[101]
4V9D	CA,AA	9.3	0.0	ND	0.191	2.7	1.7	-33.1	0.364	2651	988	446	0.54	0.77	0.72	XRAY	3.0	E. coli		Dunkle, et al. (2011) *Science*[102]
4V9D	DA,BA	0.0	0.0	ND	0.000	0.0	0.0	ND	0.000	2896	1060	462	0.00	0.00	0.00	XRAY	3.0	E. coli		Dunkle, et al. (2011) *Science*[102]
4V9H	BA,AA	7.2	1.6	36.2	1.086	3.9	1.6	-30.1	1.138	2084	815	404	0.96	1.05	0.94	XRAY	2.9	T. thermophilus HB8		Tourigny, et al. (2013) *Science*[103]
4V9I	BA,AA	1.3	0.5	6.0	0.200	-0.1	1.7	-51.4	0.449	2083	870	409	1.01	0.96	0.89	XRAY	3.3	T. thermophilus HB8		Fernandez, et al. (2013) *Nature*[104]
4V9I	DA,CA	-0.5	0.8	-9.2	0.762	0.8	2.1	-64.4	0.276	2124	895	402	1.00	1.00	0.94	XRAY	3.3	T. thermophilus HB8		Fernandez, et al. (2013) *Nature*[104]
4V9J	BA,AA	4.1	1.1	82.7	1.143	19.0	3.8	25.1	0.752	1903	807	324	1.21	1.15	1.17	XRAY	3.9	T. thermophilus HB27		Zhou, et al. (2013) *Science*[105]
4V9J	DA,CA	4.1	1.2	79.2	1.246	18.9	3.7	28.8	1.008	1951	824	326	1.21	1.14	1.13	XRAY	3.9	T. thermophilus HB27		Zhou, et al. (2013) *Science*[105]
4V9K	BA,AA	2.1	0.1	18.8	0.295	16.0	3.9	34.3	1.045	2074	850	349	1.06	1.04	1.00	XRAY	3.5	T. thermophilus HB27		Zhou, et al. (2013) *Science*[105]
4V9K	DA,CA	2.0	0.1	15.3	0.234	15.2	3.4	37.5	1.045	2067	854	363	1.05	1.02	1.05	XRAY	3.5	T. thermophilus HB27		Zhou, et al. (2013) *Science*[105]
4V9L	BA,AA	1.9	0.1	-150.2	0.528	16.0	4.2	39.5	0.939	2051	840	345	1.09	1.07	1.09	XRAY	3.5	T. thermophilus HB27		Zhou, et al. (2013) *Science*[105]
4V9L	DA,CA	1.9	0.1	-90.8	1.118	15.1	3.9	43.5	0.642	2070	857	352	1.08	1.06	1.07	XRAY	3.5	T. thermophilus HB27		Zhou, et al. (2013) *Science*[105]
4V9M	BA,AA	1.8	0.0	ND	0.844	16.2	4.0	37.5	1.034	2058	842	345	1.10	1.08	1.09	XRAY	4.0	T. thermophilus HB27		Zhou, et al. (2013) *Science*[105]
4V9M	DA,CA	1.9	0.0	ND	1.228	15.1	3.7	44.1	0.678	2067	845	349	1.09	1.06	1.09	XRAY	4.0	T. thermophilus HB27		Zhou, et al. (2013) *Science*[105]
4V9N	BA,AA	-0.8	0.7	-26.1	0.658	1.2	1.0	-44.1	0.784	2119	881	414	0.97	0.97	0.96	XRAY	3.4	T. thermophilus HB27		Santos, et al. (2013) *Structure*[106]
4V9N	DA,CA	-0.3	0.3	-71.9	0.521	0.9	1.0	-32.0	0.682	2116	882	413	0.97	0.97	0.95	XRAY	3.4	T. thermophilus HB27		Santos, et al. (2013) *Structure*[106]
4V9O	AA,BA	4.1	1.0	31.4	0.460	4.5	2.5	-5.4	0.820	2624	899	431	0.72	1.12	0.82	XRAY	2.9	E. coli K-12		Pulk, et al. (2013) *Science*[107]
4V9O	CA,DA	4.7	0.9	70.7	0.569	5.6	1.6	-8.7	0.799	2623	812	413	0.75	1.07	0.93	XRAY	2.9	E. coli K-12		Pulk, et al. (2013) *Science*[107]
4V9O	EA,FA	2.9	0.9	39.0	0.946	5.6	1.4	-0.6	0.757	2620	985	430	0.72	0.90	0.77	XRAY	2.9	E. coli K-12		Pulk, et al. (2013) *Science*[107]
4V9O	GA,HA	6.8	1.7	82.6	1.628	10.7	2.6	120.6	0.830	2563	938	361	0.76	0.93	1.10	XRAY	2.9	E. coli K-12		Pulk, et al. (2013) *Science*[107]
4V9P	AA,BA	4.6	1.6	80.6	0.483	8.1	1.8	105.7	1.203	2647	965	405	0.68	0.89	0.79	XRAY	2.9	E. coli K-12		Pulk, et al. (2013) *Science*[107]
4V9P	CA,DA	4.3	1.3	58.6	0.278	7.0	2.3	48.5	1.218	2624	783	413	0.66	1.05	0.80	XRAY	2.9	E. coli K-12		Pulk, et al. (2013) *Science*[107]
4V9P	EA,FA	-1.8	1.5	19.3	0.618	10.9	1.7	-135.9	0.778	2659	1002	422	0.67	0.79	0.79	XRAY	2.9	E. coli K-12		Pulk, et al. (2013) *Science*[107]
4V9P	GA,HA	7.9	1.0	44.9	0.525	9.2	5.3	171.8	0.798	2568	955	392	0.69	0.82	0.82	XRAY	2.9	E. coli K-12		Pulk, et al. (2013) *Science*[107]
4V9Q	AA,BA	-0.7	0.7	-9.2	0.822	1.1	1.0	-81.1	0.760	2120	891	407	0.95	0.95	0.91	XRAY	3.4	T. thermophilus HB27		Svidritskiy, et al. (2013) *PNAS*[108]
4V9Q	CA,DA	0.3	0.2	-19.2	0.371	0.1	0.8	-66.0	0.608	2116	891	407	0.94	0.94	0.91	XRAY	3.4	T. thermophilus HB27		Svidritskiy, et al. (2013) *PNAS*[108]
4V9R	BA,AA	1.0	0.4	-27.7	0.449	-0.3	1.2	-26.9	0.100	2121	883	413	0.93	0.88	0.84	XRAY	3.0	T. thermophilus		Bulkley, et al. (2014) *Cell Rep*[109]
4V9R	DA,CA	-0.7	0.8	-16.2	0.245	1.0	0.8	-48.2	0.295	2137	886	417	0.92	0.92	0.87	XRAY	3.0	T. thermophilus		Bulkley, et al. (2014) *Cell Rep*[109]
4V9S	BA,AA	0.9	0.4	-29.3	0.326	-0.2	1.2	-31.2	0.123	2105	885	413	0.93	0.89	0.84	XRAY	3.1	T. thermophilus HB8		Bulkley, et al. (2014) *Cell Rep*[109]
4V9S	DA,CA	-0.7	0.8	-15.5	0.352	1.0	0.8	-48.5	0.252	2133	893	415	0.92	0.93	0.86	XRAY	3.1	T. thermophilus HB8		Bulkley, et al. (2014) *Cell Rep*[109]
4W29	BA,AA	3.2	0.8	48.3	1.259	19.6	4.5	44.1	1.706	1735	767	320	1.27	1.22	1.19	XRAY	3.8	T. thermophilus HB27		Zhou, et al. (2014) *Science*[110]
4W29	DA,CA	3.3	0.9	53.9	1.247	19.5	4.2	44.4	1.871	1761	780	315	1.26	1.25	1.21	XRAY	3.8	T. thermophilus HB27		Zhou, et al. (2014) *Science*[110]

Table 8 of 24

PDB		BODY				HEAD				PRUNED			RMSD			EXP DETAILS		ORGANISM		REFERENCE
ID	chains	ϕ_{body}	θ_{body}	ψ_{body}	$\|\Delta\vec{x}_{\text{body}}\|$	ϕ_{head}	θ_{head}	ψ_{head}	$\|\Delta\vec{x}_{\text{head}}\|$	LSU	body	head	LSU	body	head	method	res.	name	mito	
4W2E	A,a	-1.6	1.1	-3.5	0.957	1.4	0.9	136.9	0.956	2156	878	415	0.94	1.07	0.83	XRAY	2.9	T. thermophilus		Gagnon, et al. (2014) *Science*[111]
4W2F	BA,AA	1.5	0.5	-19.6	0.633	-0.9	1.4	-26.7	0.170	2104	858	414	0.96	0.94	0.83	XRAY	2.4	T. thermophilus HB8		Polikanov, et al. (2014) *Mol Cell*[112]
4W2F	DA,CA	0.2	0.8	-21.4	0.295	-0.4	1.0	-25.4	0.192	2143	894	411	0.94	0.89	0.86	XRAY	2.4	T. thermophilus HB8		Polikanov, et al. (2014) *Mol Cell*[112]
4W2G	BA,AA	1.6	0.3	-18.5	0.681	-0.9	1.5	-23.0	0.176	2096	859	415	0.94	0.96	0.82	XRAY	2.5	T. thermophilus HB8		Polikanov, et al. (2014) *Mol Cell*[112]
4W2G	DA,CA	0.2	0.7	-26.3	0.222	-0.3	1.1	-24.5	0.087	2114	892	417	0.94	0.90	0.85	XRAY	2.5	T. thermophilus HB8		Polikanov, et al. (2014) *Mol Cell*[112]
4W2H	BA,AA	1.1	0.4	-51.1	0.853	-0.4	1.5	-29.2	0.429	2088	876	417	0.95	0.87	0.84	XRAY	2.7	T. thermophilus HB8		Polikanov, et al. (2014) *Mol Cell*[112]
4W2H	DA,CA	-0.5	0.7	-21.0	0.192	1.1	1.0	-42.2	0.264	2120	892	415	0.92	0.88	0.90	XRAY	2.7	T. thermophilus HB8		Polikanov, et al. (2014) *Mol Cell*[112]
4W2I	BA,AA	1.4	0.2	-96.5	1.046	-0.8	1.6	-27.1	0.656	2060	857	414	0.99	0.96	0.81	XRAY	2.7	T. thermophilus HB8		Polikanov, et al. (2014) *Mol Cell*[113]
4W2I	DA,CA	0.1	0.4	-23.6	0.709	-0.4	1.1	-23.9	0.583	2072	880	412	0.96	0.96	0.82	XRAY	2.7	T. thermophilus HB8		Polikanov, et al. (2014) *Mol Cell*[113]
4W4G	RA,QA	-0.8	0.7	-8.8	0.555	1.7	2.0	-62.1	0.953	2114	889	408	0.99	1.06	0.95	XRAY	3.3	T. thermophilus HB8		Schureck, et al. (2015) *PNAS*[114]
4W4G	YA,XA	-0.7	0.6	-160.6	0.704	1.8	1.5	-33.7	0.450	2116	896	408	0.98	0.98	0.89	XRAY	3.3	T. thermophilus HB8		Schureck, et al. (2015) *PNAS*[114]
4WF1	BA,AA	5.8	1.2	110.3	1.359	9.2	1.9	45.7	0.739	2658	931	375	0.63	1.11	0.78	XRAY	3.1	E. coli str. K-12 substr. MG1655		Olivier, et al. (2014) *PNAS*[115]
4WF1	DA,CA	-0.7	0.8	31.2	0.878	4.9	2.4	56.4	0.435	2836	1056	446	0.70	0.73	0.94	XRAY	3.1	E. coli str. K-12 substr. MG1655		Olivier, et al. (2014) *PNAS*[115]
4WOI	BA,AA	7.4	1.1	-173.7	0.368	1.1	1.4	-20.6	0.877	2623	869	443	0.71	1.14	0.78	XRAY	3.0	E. coli str. K-12 substr. MDS42		Wasserman, et al. (2015) *NComm*[116]
4WOI	CA,DA	1.8	0.8	-152.3	0.589	-0.7	1.2	-9.6	0.699	2770	1032	452	0.64	0.92	0.62	XRAY	3.0	E. coli str. K-12 substr. MDS42		Wasserman, et al. (2015) *NComm*[116]
4WPO	AA,BA	0.8	0.3	-28.8	0.349	-0.3	1.3	-35.8	0.168	2114	886	420	0.94	0.89	0.84	XRAY	2.8	T. thermophilus HB8		Lin, et al. (2015) *Cell*[117]
4WPO	CA,DA	0.4	0.4	-32.8	0.286	0.0	1.2	-31.5	0.076	2136	888	415	0.94	0.88	0.85	XRAY	2.8	T. thermophilus HB8		Lin, et al. (2015) *Cell*[117]
4WQ1	1H,13	1.5	0.2	-26.1	0.737	-1.0	1.4	-27.8	0.254	2112	873	408	0.93	0.97	0.83	XRAY	3.1	T. thermophilus HB8		Rozov, et al. (2015) *NComm*[118]
4WQ1	14,1G	0.0	0.7	-31.4	0.139	-0.3	0.9	-40.5	0.205	2137	884	403	0.94	0.87	0.84	XRAY	3.1	T. thermophilus HB8		Rozov, et al. (2015) *NComm*[118]
4WQF	AA,BA	1.4	0.2	41.0	0.445	0.8	1.7	-40.7	0.403	2096	875	409	0.92	0.89	0.87	XRAY	2.8	T. thermophilus HB8		Lin, et al. (2015) *Cell*[117]
4WQF	CA,DA	0.9	0.3	-8.0	0.261	0.9	1.5	-34.3	0.146	2120	888	418	0.94	0.89	0.92	XRAY	2.8	T. thermophilus HB8		Lin, et al. (2015) *Cell*[117]
4WQR	1H,13	1.3	0.1	-59.2	0.646	-0.9	1.4	-22.4	0.341	2125	862	410	0.96	0.97	0.83	XRAY	3.1	T. thermophilus HB8		Rozov, et al. (2015) *NComm*[118]
4WQR	14,1G	-0.1	0.7	-32.8	0.312	0.1	0.8	-40.9	0.279	2122	890	412	0.94	0.89	0.93	XRAY	3.1	T. thermophilus HB8		Rozov, et al. (2015) *NComm*[118]
4WQU	AA,BA	0.5	0.4	11.6	0.470	0.5	1.0	-56.3	0.473	2111	878	416	0.94	0.93	0.87	XRAY	2.8	T. thermophilus HB8		Lin, et al. (2015) *Cell*[117]
4WQU	CA,DA	0.2	0.6	-7.3	0.261	0.7	0.8	-50.6	0.365	2130	884	408	0.95	0.93	0.92	XRAY	2.8	T. thermophilus HB8		Lin, et al. (2015) *Cell*[117]
4WQY	AA,BA	0.9	0.2	0.1	0.203	0.4	1.7	-40.6	0.236	2093	881	417	0.94	0.91	0.88	XRAY	2.8	T. thermophilus HB8		Lin, et al. (2015) *Cell*[117]
4WQY	CA,DA	0.4	0.5	-17.5	0.239	0.4	1.6	-34.1	0.037	2110	891	414	0.94	0.89	0.91	XRAY	2.8	T. thermophilus HB8		Lin, et al. (2015) *Cell*[117]
4WR6	1H,13	1.5	0.1	-36.9	0.678	-0.9	1.4	-23.6	0.315	2107	856	407	0.95	0.99	0.84	XRAY	3.0	T. thermophilus HB8		Rozov, et al. (2015) *NComm*[118]
4WR6	14,1G	-0.3	0.7	-32.8	0.244	0.2	0.8	-48.8	0.365	2123	878	404	0.96	0.92	0.92	XRAY	3.0	T. thermophilus HB8		Rozov, et al. (2015) *NComm*[118]
4WRA	1H,13	1.4	0.2	-62.7	0.832	-0.9	1.4	-24.8	0.375	2091	844	410	0.99	1.04	0.87	XRAY	3.0	T. thermophilus HB8		Rozov, et al. (2015) *NComm*[118]
4WRA	14,1G	0.1	0.7	-34.9	0.549	-0.4	0.9	-47.5	0.419	2105	877	408	1.00	0.97	0.88	XRAY	3.0	T. thermophilus HB8		Rozov, et al. (2015) *NComm*[118]
4WRO	1H,13	1.5	0.1	-68.7	0.682	-0.9	1.4	-24.2	0.226	2100	857	407	0.94	0.97	0.82	XRAY	3.0	T. thermophilus HB8		Rozov, et al. (2015) *NComm*[118]
4WRO	14,1G	-0.5	0.6	-33.2	0.209	0.9	1.2	-49.9	0.290	2136	883	403	0.94	0.91	0.95	XRAY	3.0	T. thermophilus HB8		Rozov, et al. (2015) *NComm*[118]
4WSD	1H,13	1.4	0.2	-69.1	0.743	-0.7	1.5	-21.4	0.230	2099	847	412	0.96	1.02	0.83	XRAY	3.0	T. thermophilus HB8		Rozov, et al. (2015) *NComm*[118]
4WSD	14,1G	-0.2	0.7	-35.9	0.396	0.4	1.2	-42.5	0.236	2112	880	401	0.96	0.90	0.93	XRAY	3.0	T. thermophilus HB8		Rozov, et al. (2015) *NComm*[118]
4WSM	1H,13	1.3	0.5	13.0	0.350	-0.7	1.5	-28.3	0.117	2103	849	411	0.95	1.01	0.86	XRAY	3.3	T. thermophilus HB8		Rozov, et al. (2015) *NComm*[118]
4WSM	14,1G	-0.3	0.9	-12.1	0.309	0.0	1.1	-53.6	0.262	2127	889	411	0.95	0.90	0.91	XRAY	3.3	T. thermophilus HB8		Rozov, et al. (2015) *NComm*[118]
4WT1	1H,13	1.4	0.2	-36.6	0.672	-0.9	1.4	-23.2	0.308	2112	855	407	0.97	1.00	0.85	XRAY	3.0	T. thermophilus HB8		Rozov, et al. (2015) *NComm*[118]
4WT1	14,1G	-0.1	0.6	-31.4	0.478	0.2	1.0	-49.5	0.251	2131	882	396	0.94	0.91	0.96	XRAY	3.0	T. thermophilus HB8		Rozov, et al. (2015) *NComm*[118]
4WT8	C1,Ab	0.0	0.8	5.3	2.927	-0.3	0.9	-64.3	1.673	1657	778	395	1.31	1.24	1.17	XRAY	3.4	T. thermophilus HB8		Amunts, et al. (2015) *JMB*[119]
4WT8	D1,Bb	1.5	0.7	-12.5	1.521	-0.8	1.1	-44.8	1.637	1618	645	387	1.30	1.24	1.15	XRAY	3.4	T. thermophilus HB8		Amunts, et al. (2015) *JMB*[119]
4WU1	1H,13	1.6	0.3	9.4	0.460	-0.8	1.5	-29.1	0.204	2113	866	407	0.93	0.94	0.86	XRAY	3.2	T. thermophilus HB8		Rozov, et al. (2015) *NComm*[118]
4WU1	14,1G	-0.8	0.8	-23.7	0.259	1.4	1.2	-53.6	0.571	2128	884	407	0.95	0.96	0.93	XRAY	3.2	T. thermophilus HB8		Rozov, et al. (2015) *NComm*[118]
4WWW	RA,QA	5.5	1.1	117.3	1.278	9.7	1.5	36.1	0.172	2676	932	402	0.62	1.12	0.76	XRAY	3.1	E. coli str. K-12 substr. MG1655		Llano-Sotelo, et al. (2010) *AAC*[120]
4WWW	YA,XA	0.0	0.5	77.2	0.219	6.4	2.6	68.9	0.718	2721	1012	410	0.75	0.62	1.00	XRAY	3.1	E. coli str. K-12 substr. MG1655		Llano-Sotelo, et al. (2010) *AAC*[120]
4WZD	1H,13	1.5	0.2	-0.7	0.681	-0.8	1.5	-33.4	0.160	2122	863	411	0.93	0.93	0.87	XRAY	3.1	T. thermophilus HB8		Rozov, et al. (2015) *NComm*[118]
4WZD	14,1G	-0.9	0.7	-21.7	0.124	1.2	1.2	-64.4	0.473	2146	891	413	0.92	0.93	0.92	XRAY	3.1	T. thermophilus HB8		Rozov, et al. (2015) *NComm*[118]
4WZO	1H,13	1.5	0.2	-74.4	0.647	-0.7	1.5	-20.2	0.313	2116	863	409	0.96	0.99	0.86	XRAY	3.3	T. thermophilus HB8		Rozov, et al. (2015) *NComm*[118]
4WZO	14,1G	-0.7	0.6	-34.3	0.269	1.2	1.3	-52.9	0.592	2123	881	401	0.95	0.98	0.95	XRAY	3.3	T. thermophilus HB8		Rozov, et al. (2015) *NComm*[118]
4XEJ	A23S,A16S	-1.1	1.0	-4.5	0.552	1.8	0.3	-67.0	0.454	2081	876	407	0.97	0.95	0.93	XRAY	3.8	T. thermophilus HB27		Colussi, et al. (2015) *Nature*[121]
4XEJ	B23S,B16S	-0.3	0.8	2.2	0.805	1.8	1.0	-4.4	0.563	2049	889	406	1.00	0.93	0.94	XRAY	3.8	T. thermophilus HB27		Colussi, et al. (2015) *Nature*[121]
4Y4O	1A,1a	1.7	0.7	9.1	0.566	-1.4	1.4	-60.0	0.854	2120	856	418	0.95	0.99	0.86	XRAY	2.3	T. thermophilus HB8		Polikanov, et al. (2015) *NSMB*[122]

PDB		BODY				HEAD				PRUNED			RMSD			EXP DETAILS		ORGANISM		REFERENCE
ID	chains	ϕ_{body}	θ_{body}	ψ_{body}	$\lvert\Delta\vec{x}_{\text{body}}\rvert$	ϕ_{head}	θ_{head}	ψ_{head}	$\lvert\Delta\vec{x}_{\text{head}}\rvert$	LSU	body	head	LSU	body	head	method	res.	name	mito	
4Y4O	2A,2a	-1.0	1.0	-4.1	0.318	1.4	1.4	-79.6	0.235	2147	880	418	0.94	0.97	0.90	XRAY	2.3	T. thermophilus HB8		Polikanov, et al. (2015) *NSMB*[122]
4Y4P	1A,1a	1.4	0.3	-21.2	0.498	-0.8	1.4	-26.6	0.150	2095	864	415	0.96	0.95	0.84	XRAY	2.5	T. thermophilus HB8		Polikanov, et al. (2015) *NSMB*[122]
4Y4P	2A,2a	-0.2	0.7	-22.5	0.281	0.2	1.1	-33.5	0.153	2118	891	412	0.94	0.90	0.87	XRAY	2.5	T. thermophilus HB8		Polikanov, et al. (2015) *NSMB*[122]
4YBB	DA,AA	5.7	1.2	115.2	1.308	9.5	1.7	41.8	0.197	2677	916	393	0.60	1.10	0.74	XRAY	2.1	E. coli K-12		Noeske, et al. (2015) *NSMB*[123]
4YBB	CA,BA	-0.5	0.8	44.7	0.462	5.9	2.5	58.0	0.755	2767	1032	432	0.65	0.61	0.89	XRAY	2.1	E. coli K-12		Noeske, et al. (2015) *NSMB*[123]
4YPB	RA,QA	-0.6	0.8	-10.6	0.498	1.6	1.7	-62.5	0.809	2124	896	409	0.95	1.01	0.94	XRAY	3.4	T. thermophilus HB8		Schureck, et al. (2015) *PNAS*[114]
4YPB	YA,XA	-0.7	0.4	-139.6	0.739	1.6	1.5	-39.1	0.372	2122	898	410	0.95	0.93	0.88	XRAY	3.4	T. thermophilus HB8		Schureck, et al. (2015) *PNAS*[114]
4YZV	RA,QA	-0.7	0.8	-13.7	0.595	1.6	1.8	-62.1	0.917	2121	891	409	0.98	1.03	0.94	XRAY	3.1	T. thermophilus HB8		Schureck, et al. (2015) *PNAS*[114]
4YZV	YA,XA	-0.9	0.4	-132.4	0.875	1.7	1.7	-41.6	0.553	2121	894	407	0.97	0.97	0.90	XRAY	3.1	T. thermophilus HB8		Schureck, et al. (2015) *PNAS*[114]
4Z3S	1A,1a	1.4	0.3	-30.5	0.720	-0.7	1.6	-28.8	0.117	2094	858	413	0.95	0.97	0.81	XRAY	2.6	T. thermophilus HB8		Polikanov, et al. (2015) *Mol Cell*[124]
4Z3S	2A,2a	0.1	0.7	-26.6	0.221	-0.2	1.3	-40.8	0.120	2123	889	417	0.94	0.89	0.85	XRAY	2.6	T. thermophilus HB8		Polikanov, et al. (2015) *Mol Cell*[124]
4Z8C	2A,2a	-0.8	0.7	-17.8	0.450	1.2	0.9	-46.8	0.313	2154	895	406	0.94	0.95	0.87	XRAY	2.9	T. thermophilus HB8		Roy, et al. (2015) *NSMB*[125]
4Z8C	1A,1a	0.9	0.4	-56.6	0.492	-0.2	1.4	-29.4	0.111	2097	885	410	0.95	0.90	0.82	XRAY	2.9	T. thermophilus HB8		Roy, et al. (2015) *NSMB*[125]
4ZER	2A,2a	-0.7	0.8	-17.9	0.508	1.2	1.0	-49.9	0.251	2140	891	417	0.95	0.96	0.86	XRAY	3.1	T. thermophilus HB8		Seefeldt, et al. (2015) *NSMB*[126]
4ZER	1A,1a	1.0	0.3	-33.8	0.470	-0.3	1.4	-34.2	0.148	2093	876	414	0.95	0.90	0.84	XRAY	3.1	T. thermophilus HB8		Seefeldt, et al. (2015) *NSMB*[126]
4ZSN	RA,QA	-0.7	0.8	-14.1	0.516	1.6	1.8	-64.4	0.889	2112	888	410	0.95	1.00	0.94	XRAY	3.6	T. thermophilus HB8		Schureck, et al. (2016) *NAR*[127]
4ZSN	YA,XA	-0.9	0.4	-137.2	0.790	1.7	1.7	-41.4	0.449	2121	897	409	0.96	0.94	0.88	XRAY	3.6	T. thermophilus HB8		Schureck, et al. (2016) *NAR*[127]
5A9Z	AA,BA	5.9	2.8	63.7	0.414	0.7	0.3	-166.8	0.487	1987	817	358	1.14	1.17	1.11	EM	4.7	T. thermophilus HB8		Kumar, et al. (2015) *PNAS*[128]
5AA0	AA,BA	3.9	2.5	75.6	1.753	0.7	0.3	-164.5	0.498	1986	817	357	1.14	1.17	1.11	EM	5.0	T. thermophilus HB8		Kumar, et al. (2015) *PNAS*[128]
5AFI	A,a	1.1	0.9	12.6	0.610	-1.0	1.3	-56.1	0.526	2624	990	446	0.63	0.88	0.75	EM	2.9	E. coli		Fischer, et al. (2015) *Nature*[129]
5AJ0	A2,B1	-2.0	2.9	23.0	1.600	-0.7	1.1	-59.7	1.684	1345	586	283	1.24	1.25	1.19	EM	3.5	Homo sapiens		Behrmann, et al. (2015) *Cell*[130]
5AJ4	BA,AA	0.8	0.4	145.8	1.968	0.9	2.3	-137.4	1.654	1041	368	201	1.00	1.12	1.11	EM	3.8	Sus scrofa	*	Greber, et al. (2015) *Science*[131]
5CZP	YA,XA	0.2	0.6	-21.6	0.122	0.1	1.2	-36.4	0.383	2162	895	416	0.95	0.90	0.86	XRAY	3.3	T. thermophilus HB8		Pierson, et al. (2016) *Cell Rep*[132]
5CZP	RA,QA	-0.5	1.0	-15.8	0.496	0.3	0.9	-42.5	0.363	2173	883	418	0.92	0.90	0.86	XRAY	3.3	T. thermophilus HB8		Pierson, et al. (2016) *Cell Rep*[132]
5D8B	VC,UC	-0.6	0.7	-11.8	0.918	1.0	1.4	-79.5	1.012	2076	885	402	1.04	1.02	0.99	XRAY	3.6	T. thermophilus HB27		Svidritskiy, et al. (2015) *Structure*[133]
5D8B	ZC,YC	0.2	0.2	-29.5	0.207	0.7	1.3	-61.0	0.886	2085	886	402	1.05	1.02	0.99	XRAY	3.6	T. thermophilus HB27		Svidritskiy, et al. (2015) *Structure*[133]
5DAT	5,6	7.5	4.8	53.6	0.726	10.6	3.2	37.7	0.821	1387	592	265	1.23	1.17	1.14	XRAY	3.1	S. cerevisiae S288C		Melnikov, et al. *To be published*
5DAT	1,2	4.2	3.0	87.9	1.003	13.9	8.6	64.1	0.533	1387	597	258	1.24	1.16	1.11	XRAY	3.1	S. cerevisiae S288C		Melnikov, et al. *To be published*
5DC3	5,6	7.5	4.7	52.1	0.629	10.2	3.2	33.1	0.952	1291	571	269	1.26	1.21	1.17	XRAY	3.2	S. cerevisiae S288C		Melnikov, et al. (2016) *JMB*[134]
5DC3	1,2	4.0	2.8	86.5	0.823	14.0	8.5	62.5	0.295	1305	581	258	1.28	1.21	1.14	XRAY	3.2	S. cerevisiae S288C		Melnikov, et al. (2016) *JMB*[134]
5DFE	YA,XA	0.5	0.5	-32.8	0.359	-0.1	1.3	-36.4	0.324	2150	891	417	0.94	0.90	0.86	XRAY	3.1	T. thermophilus HB8		Pierson, et al. (2016) *Cell Rep*[132]
5DFE	RA,QA	-0.5	0.9	-16.2	0.573	0.3	0.9	-47.8	0.338	2159	876	417	0.93	0.89	0.84	XRAY	3.1	T. thermophilus HB8		Pierson, et al. (2016) *Cell Rep*[132]
5DGE	1,2	3.9	2.8	88.8	1.003	14.3	8.2	61.5	0.242	1338	588	259	1.25	1.19	1.13	XRAY	3.5	S. cerevisiae S288C		Melnikov, et al. *To be published*
5DGE	5,6	7.6	4.6	52.5	0.486	10.2	3.2	26.7	1.035	1338	582	260	1.25	1.21	1.13	XRAY	3.5	S. cerevisiae S288C		Melnikov, et al. *To be published*
5DGF	5,6	7.7	4.8	53.4	0.572	10.1	3.1	27.1	0.932	1398	583	265	1.23	1.18	1.13	XRAY	3.3	S. cerevisiae S288C		Melnikov, et al. *To be published*
5DGF	1,2	4.1	3.0	90.3	1.095	14.0	8.4	62.2	0.597	1422	608	261	1.24	1.19	1.12	XRAY	3.3	S. cerevisiae S288C		Melnikov, et al. *To be published*
5DGV	1,2	4.2	3.0	88.0	1.264	14.2	8.6	63.9	0.322	1364	602	258	1.25	1.19	1.12	XRAY	3.1	S. cerevisiae S288C		Melnikov, et al. *To be published*
5DGV	5,6	8.0	4.8	54.4	0.548	9.8	2.8	23.7	0.529	1358	580	263	1.25	1.19	1.14	XRAY	3.1	S. cerevisiae S288C		Melnikov, et al. *To be published*
5DOX	1A,1a	1.6	0.9	3.5	0.596	-0.5	0.9	-36.0	0.169	2102	859	411	0.93	0.96	0.95	XRAY	3.1	T. thermophilus HB8		Polikanov, et al. (2015) *Mol Cell*[124]
5DOX	2A,2a	-0.8	1.1	-0.2	0.254	1.5	0.5	-76.7	0.546	2156	888	412	0.93	0.92	0.99	XRAY	3.1	T. thermophilus HB8		Polikanov, et al. (2015) *Mol Cell*[124]
5DOY	1A,1a	1.4	0.3	-33.0	1.055	-0.8	1.6	-29.9	0.475	2075	863	414	0.96	0.95	0.83	XRAY	2.6	T. thermophilus HB8		Polikanov, et al. (2015) *Mol Cell*[124]
5DOY	2A,2a	-0.1	0.7	-20.8	0.308	0.1	1.1	-41.4	0.390	2103	887	415	0.93	0.87	0.88	XRAY	2.6	T. thermophilus HB8		Polikanov, et al. (2015) *Mol Cell*[124]
5E7K	14,1G	0.0	0.6	-42.6	0.496	-0.1	0.9	-33.1	0.419	2097	878	412	1.00	0.95	0.92	XRAY	3.2	T. thermophilus HB8		Rozov, et al. (2016) *NComm*[135]
5E7K	1H,13	1.3	0.2	-124.3	0.474	-0.6	1.4	-18.6	0.356	2086	863	416	0.99	1.01	0.86	XRAY	3.2	T. thermophilus HB8		Rozov, et al. (2016) *NComm*[135]
5E81	1H,13	1.3	0.2	-93.7	0.445	-0.8	1.3	-18.3	0.187	2094	866	416	0.94	0.94	0.82	XRAY	3.0	T. thermophilus HB8		Rozov, et al. (2016) *NComm*[135]
5E81	14,1G	0.0	0.7	-40.9	0.191	-0.3	0.7	-23.0	0.237	2120	893	414	0.94	0.89	0.88	XRAY	3.0	T. thermophilus HB8		Rozov, et al. (2016) *NComm*[135]
5EL4	14,1G	-0.6	0.6	-41.8	0.113	1.2	1.2	-53.7	0.176	2111	886	411	0.93	0.91	0.96	XRAY	3.1	T. thermophilus HB8		Rozov, et al. (2016) *NComm*[135]
5EL4	1H,13	1.6	0.2	-93.2	0.520	-0.8	1.6	-23.4	0.118	2087	857	415	0.94	0.99	0.84	XRAY	3.1	T. thermophilus HB8		Rozov, et al. (2016) *NComm*[135]
5EL5	14,1G	-0.4	0.7	-34.2	0.056	0.4	1.0	-49.3	0.294	2116	881	394	0.95	0.90	0.99	XRAY	3.1	T. thermophilus HB8		Rozov, et al. (2016) *NComm*[135]
5EL5	1H,13	1.5	0.1	-42.6	0.814	-0.8	1.3	-27.3	0.269	2084	862	409	0.95	0.99	0.86	XRAY	3.1	T. thermophilus HB8		Rozov, et al. (2016) *NComm*[135]
5EL6	14,1G	0.0	0.6	-53.5	0.198	-0.1	1.1	-36.1	0.133	2099	887	406	0.95	0.93	0.88	XRAY	3.1	T. thermophilus HB8		Rozov, et al. (2016) *NComm*[135]
5EL6	1H,13	1.4	0.3	-110.3	0.319	-0.6	1.5	-19.6	0.147	2067	850	413	0.95	1.02	0.83	XRAY	3.1	T. thermophilus HB8		Rozov, et al. (2016) *NComm*[135]

PDB		BODY				HEAD				PRUNED			RMSD			EXP DETAILS		ORGANISM		REFERENCE
ID	chains	ϕ_{body}	θ_{body}	ψ_{body}	$\lvert\Delta\vec{x}_{\text{body}}\rvert$	ϕ_{head}	θ_{head}	ψ_{head}	$\lvert\Delta\vec{x}_{\text{head}}\rvert$	LSU	body	head	LSU	body	head	method	res.	name	mito	
5EL7	14,1G	0.1	0.6	-46.5	0.423	-0.3	0.8	-33.6	0.449	2096	873	408	0.98	0.94	0.89	XRAY	3.1	T. thermophilus HB8		Rozov, et al. (2016) *NComm*[135]
5EL7	1H,13	1.4	0.2	-87.5	0.641	-0.7	1.3	-24.4	0.360	2089	851	417	0.98	1.04	0.87	XRAY	3.1	T. thermophilus HB8		Rozov, et al. (2016) *NComm*[135]
5F8K	1A,1a	1.4	0.3	-37.9	0.636	-0.9	1.5	-31.0	0.194	2088	868	419	0.97	0.95	0.85	XRAY	2.8	T. thermophilus HB8		Seefeldt, et al. (2016) *NAR*[136]
5F8K	2A,2a	-0.7	0.6	-26.1	0.434	1.3	1.2	-55.3	0.487	2151	893	416	0.97	0.99	0.89	XRAY	2.8	T. thermophilus HB8		Seefeldt, et al. (2016) *NAR*[136]
5FCI	5,6	7.7	4.8	55.0	0.542	10.2	3.0	39.7	0.665	1371	581	268	1.27	1.18	1.18	XRAY	3.4	S. cerevisiae S288C		Mailliot, et al. (2016) *JMB*[137]
5FCI	1,2	4.4	3.1	87.6	0.872	13.8	8.5	64.5	0.521	1362	591	257	1.26	1.18	1.12	XRAY	3.4	S. cerevisiae S288C		Mailliot, et al. (2016) *JMB*[137]
5FCJ	5,6	7.7	4.9	54.3	0.516	10.1	2.7	37.3	0.623	1388	595	268	1.26	1.18	1.16	XRAY	3.1	S. cerevisiae S288C		Mailliot, et al. (2016) *JMB*[137]
5FCJ	1,2	4.3	3.1	86.2	0.967	14.0	8.5	64.5	0.539	1381	605	259	1.25	1.18	1.13	XRAY	3.1	S. cerevisiae S288C		Mailliot, et al. (2016) *JMB*[137]
5FDU	1A,1a	1.4	0.7	3.5	0.516	-1.3	1.2	-56.0	0.627	2112	867	410	0.94	0.91	0.85	XRAY	2.9	T. thermophilus HB8		Seefeldt, et al. (2016) *NAR*[136]
5FDU	2A,2a	-0.8	1.0	-2.1	0.234	0.9	1.0	-78.6	0.199	2144	890	419	0.91	0.93	0.89	XRAY	2.9	T. thermophilus HB8		Seefeldt, et al. (2016) *NAR*[136]
5FDV	1A,1a	1.4	0.6	6.3	0.468	-1.3	1.2	-56.2	0.657	2108	870	412	0.93	0.91	0.86	XRAY	2.8	T. thermophilus HB8		Seefeldt, et al. (2016) *NAR*[136]
5FDV	2A,2a	-0.7	1.1	-6.8	0.249	0.8	1.0	-71.5	0.223	2153	887	415	0.92	0.95	0.88	XRAY	2.8	T. thermophilus HB8		Seefeldt, et al. (2016) *NAR*[136]
5H5U	A,h	-0.7	1.2	12.0	1.492	1.3	2.2	-78.0	1.416	2528	971	419	0.99	0.98	0.88	EM	3.0	E. coli K-12		Ma, et al. (2017) *Nature*[138]
5HAU	1A,1a	0.8	0.2	-8.2	0.171	0.5	1.9	-44.6	0.100	2102	883	416	0.94	0.89	0.87	XRAY	3.0	T. thermophilus HB8		Gagnon, et al. (2016) *NAR*[139]
5HAU	2A,2a	0.2	0.5	-20.2	0.249	0.8	1.6	-45.0	0.125	2116	887	418	0.95	0.90	0.88	XRAY	3.0	T. thermophilus HB8		Gagnon, et al. (2016) *NAR*[139]
5HCP	2A,2a	-0.6	0.8	-18.5	0.274	1.0	0.9	-40.7	0.217	2151	888	407	0.94	0.93	0.89	XRAY	2.9	T. thermophilus HB8		Gagnon, et al. (2016) *NAR*[139]
5HCP	1A,1a	1.2	0.4	-32.3	0.556	-0.6	1.3	-29.3	0.057	2112	884	411	0.95	0.91	0.84	XRAY	2.9	T. thermophilus HB8		Gagnon, et al. (2016) *NAR*[139]
5HCQ	2A,2a	-0.7	0.8	-19.3	0.366	1.1	0.9	-41.3	0.265	2155	895	411	0.94	0.95	0.90	XRAY	2.8	T. thermophilus HB8		Gagnon, et al. (2016) *NAR*[139]
5HCQ	1A,1a	1.3	0.3	-31.8	0.565	-0.8	1.4	-28.8	0.068	2113	871	410	0.95	0.90	0.82	XRAY	2.8	T. thermophilus HB8		Gagnon, et al. (2016) *NAR*[139]
5HCR	2A,2a	-0.7	0.8	-18.2	0.314	1.1	1.0	-44.7	0.238	2157	896	405	0.94	0.95	0.89	XRAY	2.8	T. thermophilus HB8		Gagnon, et al. (2016) *NAR*[139]
5HCR	1A,1a	1.3	0.4	-23.3	0.538	-0.7	1.3	-29.0	0.035	2113	872	411	0.95	0.90	0.84	XRAY	2.8	T. thermophilus HB8		Gagnon, et al. (2016) *NAR*[139]
5HD1	2A,2a	-0.6	0.8	-17.7	0.264	1.0	0.9	-38.3	0.241	2160	890	407	0.94	0.93	0.91	XRAY	2.7	T. thermophilus HB8		Gagnon, et al. (2016) *NAR*[139]
5HD1	1A,1a	1.2	0.3	-40.0	0.567	-0.6	1.4	-27.6	0.048	2114	877	408	0.94	0.90	0.82	XRAY	2.7	T. thermophilus HB8		Gagnon, et al. (2016) *NAR*[139]
5I4L	1,2	4.4	2.7	86.1	0.851	13.4	8.7	62.7	0.507	1413	594	265	1.24	1.16	1.13	XRAY	3.1	S. cerevisiae S288C		Prokhorova, et al. (2016) *Sci Rep*[140]
5I4L	5,6	7.6	4.8	54.0	0.646	10.5	3.3	40.2	0.591	1394	603	269	1.24	1.19	1.15	XRAY	3.1	S. cerevisiae S288C		Prokhorova, et al. (2016) *Sci Rep*[140]
5IB7	1H,13	1.4	0.2	-78.6	0.622	-0.8	1.5	-16.9	0.160	2095	871	415	0.93	0.98	0.85	XRAY	3.0	T. thermophilus HB8		Rozov, et al. (2016) *NAR*[141]
5IB7	14,1G	-0.2	0.7	-35.0	0.183	0.3	0.9	-34.4	0.153	2116	892	405	0.93	0.88	0.93	XRAY	3.0	T. thermophilus HB8		Rozov, et al. (2016) *NAR*[141]
5IB8	1H,13	1.4	0.2	-84.2	0.490	-0.8	1.4	-20.6	0.109	2094	880	414	0.92	0.95	0.84	XRAY	3.1	T. thermophilus HB8		Rozov, et al. (2016) *NAR*[141]
5IB8	14,1G	-0.7	0.7	-32.3	0.061	1.0	1.2	-51.2	0.332	2129	886	404	0.92	0.93	0.93	XRAY	3.1	T. thermophilus HB8		Rozov, et al. (2016) *NAR*[141]
5IBB	1H,13	1.3	0.2	-88.2	0.530	-0.8	1.4	-20.3	0.189	2101	871	414	0.93	0.93	0.84	XRAY	3.0	T. thermophilus HB8		Rozov, et al. (2016) *NAR*[141]
5IBB	14,1G	0.1	0.7	-39.4	0.205	-0.2	0.8	-20.0	0.126	2107	898	409	0.92	0.88	0.89	XRAY	3.0	T. thermophilus HB8		Rozov, et al. (2016) *NAR*[141]
5IMQ	D,A	2.3	1.2	32.6	0.582	-1.4	0.8	-60.0	0.175	2044	800	388	1.06	1.18	1.07	EM	3.8	T. thermophilus HB8		Kumar, et al. (2016) *J Biol Chem*[142]
5IMR	D,A	4.6	1.8	61.3	0.696	-1.4	1.2	4.9	0.526	1758	701	252	1.30	1.27	1.36	EM	5.7	T. thermophilus HB8		Kumar, et al. (2016) *J Biol Chem*[142]
5IQR	1,2	-1.5	1.1	15.9	1.841	1.2	1.1	-104.0	1.501	2527	974	435	1.21	1.05	0.91	EM	3.0	E. coli K-12		Brown, et al. (2016) *Nature*[143]
5IT7	5,2	5.8	5.7	32.3	0.803	10.2	4.1	65.3	0.672	1359	537	264	1.24	1.23	1.18	EM	3.6	Kluyveromyces lactis		Murray, et al. (2016) *Elife*[144]
5IT8	DA,AA	5.6	1.2	111.8	1.405	9.6	1.6	44.1	0.448	2662	921	396	0.69	1.13	0.77	XRAY	3.1	E. coli K-12		Cocozaki, et al. (2016) *PNAS*[145]
5IT8	CA,BA	-0.4	0.6	46.4	0.669	6.0	2.5	57.9	0.952	2764	1030	432	0.72	0.65	0.83	XRAY	3.1	E. coli K-12		Cocozaki, et al. (2016) *PNAS*[145]
5J30	RA,QA	-0.5	0.9	-19.9	0.468	0.6	1.5	-55.2	0.318	2151	873	419	0.93	0.92	0.87	XRAY	3.2	T. thermophilus HB8		Pierson, et al. (2016) *Cell Rep*[132]
5J30	YA,XA	0.1	0.4	-25.4	0.048	0.3	1.7	-48.2	0.315	2131	891	417	0.94	0.92	0.88	XRAY	3.2	T. thermophilus HB8		Pierson, et al. (2016) *Cell Rep*[132]
5J3C	RA,QA	-0.6	0.8	-18.7	0.727	0.6	1.4	-56.6	0.246	2131	879	418	0.93	0.92	0.88	XRAY	3.0	T. thermophilus HB8		Pierson, et al. (2016) *Cell Rep*[132]
5J3C	YA,XA	0.2	0.5	-28.2	0.030	0.3	1.8	-43.2	0.183	2129	889	417	0.96	0.93	0.88	XRAY	3.0	T. thermophilus HB8		Pierson, et al. (2016) *Cell Rep*[132]
5J4B	1A,1a	1.6	0.4	-17.0	0.512	-0.8	1.4	-27.3	0.362	2082	840	415	0.99	0.99	0.86	XRAY	2.6	T. thermophilus HB8		Melnikov, et al. (2016) *NAR*[146]
5J4B	2A,2a	0.1	0.8	-23.0	0.568	-0.1	1.2	-36.4	0.386	2108	883	410	0.98	0.93	0.87	XRAY	2.6	T. thermophilus HB8		Melnikov, et al. (2016) *NAR*[146]
5J4C	1A,1a	1.5	0.3	-26.4	0.768	-0.6	1.5	-26.6	0.053	2080	847	413	0.95	0.97	0.82	XRAY	2.8	T. thermophilus HB8		Melnikov, et al. (2016) *NAR*[146]
5J4C	2A,2a	0.2	0.7	-32.4	0.219	-0.2	1.3	-39.4	0.082	2113	889	410	0.94	0.90	0.84	XRAY	2.8	T. thermophilus HB8		Melnikov, et al. (2016) *NAR*[146]
5J4D	B,A	0.1	0.3	-38.4	0.287	0.1	1.5	-61.3	0.520	2116	892	400	0.93	0.91	0.87	XRAY	3.1	T. thermophilus HB27		Svidritskiy, et al. (2016) *JMB*[147]
5J4D	GB,FB	-0.5	0.7	-19.9	0.483	0.8	1.4	-76.6	0.670	2123	891	401	0.93	0.92	0.88	XRAY	3.1	T. thermophilus HB27		Svidritskiy, et al. (2016) *JMB*[147]
5J5B	DA,AA	5.7	1.3	113.7	1.289	9.6	1.7	40.5	0.165	2677	921	392	0.59	1.10	0.73	XRAY	2.8	E. coli K-12		Cocozaki, et al. (2016) *PNAS*[145]
5J5B	CA,BA	-0.4	0.8	37.0	0.753	5.9	2.5	56.8	0.767	2768	1030	433	0.64	0.59	0.82	XRAY	2.8	E. coli K-12		Cocozaki, et al. (2016) *PNAS*[145]
5J7L	DA,AA	5.8	1.3	106.7	1.349	9.4	1.8	43.9	0.489	2663	917	394	0.64	1.12	0.78	XRAY	3.0	E. coli K-12		Cocozaki, et al. (2016) *PNAS*[145]
5J7L	CA,BA	-0.6	0.9	42.4	0.651	5.9	2.6	56.9	0.746	2768	1029	435	0.68	0.71	0.86	XRAY	3.0	E. coli K-12		Cocozaki, et al. (2016) *PNAS*[145]
5J88	DA,AA	5.7	1.2	112.5	1.258	9.6	1.8	38.5	0.240	2664	913	392	0.64	1.14	0.89	XRAY	3.3	E. coli		Cocozaki, et al. (2016) *PNAS*[145]

PDB		BODY				HEAD				PRUNED			RMSD			EXP DETAILS		ORGANISM		REFERENCE
ID	chains	ϕ_{body}	θ_{body}	ψ_{body}	$\lvert\Delta\vec{x}_{\text{body}}\rvert$	ϕ_{head}	θ_{head}	ψ_{head}	$\lvert\Delta\vec{x}_{\text{head}}\rvert$	LSU	body	head	LSU	body	head	method	res.	name	mito	
5J88	CA,BA	-0.8	0.8	55.2	0.451	6.2	2.8	54.3	0.666	2757	1025	423	0.72	0.67	0.94	XRAY	3.3	E. coli		Cocozaki, et al. (2016) *PNAS*[145]
5J8A	DA,AA	5.7	1.3	104.8	1.361	9.6	1.8	48.1	0.415	2665	907	393	0.64	1.12	0.78	XRAY	3.1	E. coli		Cocozaki, et al. (2016) *PNAS*[145]
5J8A	CA,BA	-0.5	0.8	55.3	0.438	5.9	2.5	56.9	0.725	2767	1031	433	0.67	0.65	0.84	XRAY	3.1	E. coli		Cocozaki, et al. (2016) *PNAS*[145]
5J8B	A,a	0.9	1.0	-23.3	1.057	-1.3	0.6	4.4	0.136	2129	884	409	0.97	0.88	0.80	XRAY	2.6	T. thermophilus HB8		Gagnon, et al. (2016) *PNAS*[148]
5J91	DA,AA	5.7	1.2	109.8	1.215	9.6	1.7	40.5	0.165	2677	921	392	0.59	1.10	0.73	XRAY	3.0	E. coli		Cocozaki, et al. (2016) *PNAS*[145]
5J91	CA,BA	-0.5	0.8	41.5	0.698	5.9	2.5	56.8	0.767	2768	1030	433	0.64	0.59	0.82	XRAY	3.0	E. coli		Cocozaki, et al. (2016) *PNAS*[145]
5JC9	DA,AA	5.6	1.3	92.4	1.270	10.1	2.1	39.6	0.426	2657	903	391	0.66	1.10	0.77	XRAY	3.0	E. coli K-12		Cocozaki, et al. (2016) *PNAS*[145]
5JC9	CA,BA	-0.8	1.3	26.0	1.005	5.8	2.2	63.6	0.808	2769	1031	438	0.71	0.72	0.88	XRAY	3.0	E. coli K-12		Cocozaki, et al. (2016) *PNAS*[145]
5JTE	BA,AA	0.2	1.2	-3.5	0.466	0.3	1.1	-48.2	0.263	2758	1028	431	0.73	0.74	0.84	EM	3.6	E. coli		Arenz, et al. (2016) *NComm*[149]
5JU8	BA,AA	-1.4	1.2	6.4	0.349	0.6	1.3	-90.8	1.139	2758	1031	428	0.72	0.72	0.87	EM	3.6	E. coli K-12		Arenz, et al. (2016) *NComm*[149]
5JUO	B,A	6.8	6.5	26.0	0.775	9.8	4.5	35.4	0.562	1222	509	212	1.33	1.33	1.36	EM	4.0	S. cerevisiae		Abeyrathne, et al. (2016) *Elife*[150]
5JUP	B,A	1.6	4.5	-6.1	1.147	14.6	7.5	52.3	1.127	1383	500	248	1.23	1.28	1.20	EM	3.5	S. cerevisiae		Abeyrathne, et al. (2016) *Elife*[150]
5JUS	B,A	1.8	4.4	-7.7	1.068	14.7	7.5	52.8	1.208	1185	485	241	1.33	1.34	1.30	EM	4.2	S. cerevisiae		Abeyrathne, et al. (2016) *Elife*[150]
5JUT	B,A	-0.8	3.3	15.5	0.648	11.3	5.1	47.6	0.651	1289	559	225	1.29	1.25	1.32	EM	4.0	S. cerevisiae		Abeyrathne, et al. (2016) *Elife*[150]
5JUU	B,A	-1.6	2.9	11.2	0.882	-0.2	2.3	-56.7	1.282	1356	593	274	1.27	1.24	1.25	EM	4.0	S. cerevisiae		Abeyrathne, et al. (2016) *Elife*[150]
5KCR	1A,1a	-1.3	1.4	9.8	0.667	0.5	1.4	-88.8	1.283	2678	1031	438	0.60	0.76	0.88	EM	3.6	E. coli K-12		Arenz, et al. (2016) *PNAS*[151]
5KCS	1A,1a	-1.4	1.1	9.8	0.429	0.9	2.7	-79.6	0.735	2622	980	445	0.79	0.94	0.88	EM	3.9	E. coli K-12		Arenz, et al. (2016) *PNAS*[151]
5KPS	28,27	-1.4	1.3	14.0	0.557	1.3	1.5	-96.4	0.811	2612	995	441	0.71	0.74	0.81	EM	3.9	E. coli K-12		Loveland, et al. (2016) *Elife*[152]
5KPV	27,26	-1.9	1.1	16.2	0.630	1.9	1.3	-105.2	0.917	2613	1000	440	0.75	0.82	0.85	EM	4.1	E. coli K-12		Loveland, et al. (2016) *Elife*[152]
5KPW	27,26	-1.3	1.0	13.2	0.686	1.3	0.9	-104.9	0.750	2610	1000	435	0.74	0.72	0.78	EM	3.9	E. coli K-12		Loveland, et al. (2016) *Elife*[152]
5KPX	27,26	0.4	0.7	9.8	0.835	-0.2	1.0	-52.9	0.818	2607	984	443	0.75	0.85	0.82	EM	3.9	E. coli K-12		Loveland, et al. (2016) *Elife*[152]
5L3P	A,a	-1.4	1.1	8.6	0.447	1.1	2.1	-78.1	0.693	2617	979	436	0.79	0.94	0.89	EM	3.7	E. coli K-12		Arenz, et al. (2016) *NAR*[153]
5LI0	A,a	-0.9	1.6	21.8	1.856	0.3	2.2	-107.1	2.043	2053	812	383	1.27	1.30	1.15	EM	3.8	Staph. aureus subsp. aureus NCTC 8325		Khusainov, et al. (2016) *NAR*[154]
5LKS	L5,S2	-1.8	3.0	22.7	1.680	-0.2	1.0	-70.3	1.403	1253	565	258	1.30	1.28	1.30	EM	3.6	Homo sapiens		Myasnikov, et al. (2016) *NComm*[155]
5LYB	1,2	4.3	2.9	87.6	0.935	13.9	8.7	62.9	0.451	1378	592	259	1.25	1.17	1.12	XRAY	3.2	S. cerevisiae		Melnikov, et al. (2016) *EMBO Rep*[156]
5LYB	5,6	7.7	4.8	53.5	0.535	9.9	2.9	24.6	0.808	1390	580	266	1.26	1.19	1.13	XRAY	3.2	S. cerevisiae		Melnikov, et al. (2016) *EMBO Rep*[156]
5LZA	A,a	-1.4	1.4	16.8	0.622	1.1	1.8	-96.7	0.980	2617	989	439	0.78	0.84	0.88	EM	3.6	E. coli		Fischer, et al. (2016) *Nature*[157]
5LZB	A,a	-1.9	1.4	11.2	0.939	1.2	1.8	-101.5	1.120	2593	967	431	0.96	1.01	1.05	EM	5.3	E. coli		Fischer, et al. (2016) *Nature*[157]
5LZC	A,a	-1.5	1.5	12.8	0.846	0.9	1.6	-99.4	0.986	2580	975	432	0.92	0.97	1.00	EM	4.8	E. coli		Fischer, et al. (2016) *Nature*[157]
5LZD	A,a	1.0	0.9	16.7	0.136	-0.9	1.2	-52.8	0.473	2608	977	439	0.74	0.96	0.78	EM	3.4	E. coli		Fischer, et al. (2016) *Nature*[157]
5LZE	A,a	0.7	0.6	13.7	0.076	-0.4	1.4	-54.6	0.396	2621	953	440	0.74	0.92	0.84	EM	3.5	E. coli		Fischer, et al. (2016) *Nature*[157]
5LZF	A,a	9.6	1.9	20.7	1.032	3.2	2.3	-31.4	0.984	2512	927	417	0.94	1.07	1.04	EM	4.6	E. coli		Fischer, et al. (2016) *Nature*[157]
5LZS	5,9	-0.7	3.6	22.7	0.739	-1.7	0.6	-58.5	1.359	1434	625	294	1.19	1.17	1.11	EM	3.3	Oryctolagus cuniculus		Shao, et al. (2016) *Cell*[158]
5LZT	5,9	-2.0	3.1	19.0	1.455	-1.3	1.2	-83.6	1.185	1418	611	294	1.20	1.16	1.12	EM	3.6	Oryctolagus cuniculus		Shao, et al. (2016) *Cell*[158]
5LZU	5,9	-2.3	3.0	22.8	1.495	-1.4	1.3	-82.4	1.193	1403	619	293	1.20	1.16	1.09	EM	3.8	Oryctolagus cuniculus		Shao, et al. (2016) *Cell*[158]
5LZV	5,9	-2.1	2.8	30.2	1.271	-1.4	1.4	-82.4	1.162	1411	623	294	1.24	1.16	1.10	EM	3.4	Oryctolagus cuniculus		Shao, et al. (2016) *Cell*[158]
5LZW	5,9	-2.3	2.9	17.7	1.658	-0.6	2.0	-72.0	1.383	1408	590	294	1.21	1.23	1.11	EM	3.5	Oryctolagus cuniculus		Shao, et al. (2016) *Cell*[158]
5LZX	5,9	-2.4	2.9	16.9	1.703	-0.6	1.8	-71.7	1.430	1405	592	296	1.21	1.23	1.12	EM	3.7	Oryctolagus cuniculus		Shao, et al. (2016) *Cell*[158]
5LZY	5,9	-2.4	2.9	15.7	1.672	-0.4	1.9	-68.2	1.430	1397	584	293	1.20	1.23	1.13	EM	4.0	Oryctolagus cuniculus		Shao, et al. (2016) *Cell*[158]
5LZZ	5,9	-2.5	2.9	16.2	1.705	-0.3	1.9	-70.2	1.454	1417	580	294	1.20	1.23	1.12	EM	3.5	Oryctolagus cuniculus		Shao, et al. (2016) *Cell*[158]
5M1J	14,22	-2.0	2.5	18.5	1.431	-0.3	2.4	-60.3	1.475	1355	571	295	1.26	1.25	1.19	EM	3.3	S. cerevisiae		Hilal, et al. (2016) *NComm*[159]
5MC6	BQ,2	2.3	2.9	69.1	1.193	-1.3	1.5	-31.2	1.236	1376	570	270	1.23	1.21	1.17	EM	3.8	S. cerevisiae S288C		Schmidt, et al. (2016) *Science*[160]
5MDV	1,2	-1.5	1.6	9.9	0.488	0.9	2.2	-88.8	0.987	2673	1003	440	0.62	0.69	0.76	EM	3.0	E. coli		James, et al. (2016) *Science*[161]
5MDW	1,2	-1.7	1.2	13.3	0.429	1.2	2.0	-90.1	1.076	2651	1004	438	0.60	0.77	0.76	EM	3.1	E. coli		James, et al. (2016) *Science*[161]
5MDY	1,2	-1.7	1.0	16.0	0.638	1.5	1.8	-93.3	1.112	2654	1002	437	0.68	0.81	0.79	EM	3.4	E. coli K-12		James, et al. (2016) *Science*[161]
5MDZ	1,2	-1.6	1.3	12.5	0.468	1.0	2.0	-92.6	1.018	2653	1003	439	0.60	0.74	0.75	EM	3.1	E. coli		James, et al. (2016) *Science*[161]
5MEI	1,A	4.0	3.0	86.5	0.949	14.0	8.4	64.7	0.469	1390	600	259	1.24	1.16	1.12	XRAY	3.5	S. cerevisiae S288C		McClary, et al. (2017) *Cell Chem Biol*[162]
5MEI	AR,6	7.8	4.8	54.7	0.424	10.3	3.1	40.3	0.472	1405	591	261	1.25	1.17	1.13	XRAY	3.5	S. cerevisiae S288C		McClary, et al. (2017) *Cell Chem Biol*[162]
5MGP	A,a	-1.6	1.6	9.7	0.529	1.2	2.1	-91.2	1.109	2648	1001	441	0.68	0.73	0.85	EM	3.1	E. coli		Huter, et al. (2017) *Nature*[163]
5MMM	A,a	-0.3	1.1	55.5	1.810	0.3	1.5	-79.2	2.211	2064	862	426	1.09	1.16	0.96	EM	3.4	Spinacia oleracea		Bieri, et al. (2017) *EMBO J*[164]
5MRC	A,aa	4.1	2.3	85.3	0.523	13.9	4.4	39.8	0.970	1517	561	292	1.10	1.09	1.08	EM	3.2	S. cerevisiae	*	Desai, et al. (2017) *Science*[165]
5MRE	A,aa	6.0	2.4	83.1	0.576	5.6	1.8	-25.4	0.379	1516	561	313	1.10	1.09	1.13	EM	3.8	S. cerevisiae	*	Desai, et al. (2017) *Science*[165]

PDB		BODY				HEAD				PRUNED			RMSD			EXP DETAILS		ORGANISM		REFERENCE
ID	chains	ϕ_{body}	θ_{body}	ψ_{body}	$\lvert\Delta\vec{x}_{\text{body}}\rvert$	ϕ_{head}	θ_{head}	ψ_{head}	$\lvert\Delta\vec{x}_{\text{head}}\rvert$	LSU	body	head	LSU	body	head	method	res.	name	mito	
5MRF	A,aa	1.0	1.9	46.8	1.448	15.4	3.8	50.8	0.820	1518	561	294	1.10	1.09	1.09	EM	5.0	S. cerevisiae	*	Desai, et al. (2017) *Science*[165]
5MYJ	BA,AA	-1.2	1.3	16.6	0.658	0.4	1.9	-90.8	2.104	2089	670	256	1.18	1.29	1.37	EM	5.6	Lact. lactis subsp. cremoris MG1363		Franken, et al. (2017) *NComm*[166]
5ND8	A,a	-1.1	1.5	37.6	2.762	1.0	2.8	-99.1	2.770	1937	751	380	1.31	1.31	1.21	EM	3.7	Staph. aureus subsp. aureus NCTC 8325		Khusainov, et al. (2017) *EMBO J*[167]
5ND9	A,a	7.7	1.7	72.1	1.958	4.7	4.4	-53.7	2.725	1899	722	365	1.30	1.30	1.23	EM	3.7	Staph. aureus subsp. aureus NCTC 8325		Khusainov, et al. (2017) *EMBO J*[167]
5NDG	5,6	4.1	3.4	69.4	1.475	13.5	6.5	68.0	0.308	1384	616	261	1.26	1.19	1.13	XRAY	3.7	S. cerevisiae		Prokhorova, et al. (2017) *PNAS*[168]
5NDG	1,2	4.4	3.7	61.0	1.304	13.3	5.5	70.2	0.196	1377	603	264	1.25	1.19	1.12	XRAY	3.7	S. cerevisiae		Prokhorova, et al. (2017) *PNAS*[168]
5NDJ	1H,13	1.5	0.2	-9.1	0.693	-0.8	1.1	-25.7	0.062	2105	858	409	0.95	0.99	0.82	XRAY	3.1	T. thermophilus HB8		Prokhorova, et al. (2017) *PNAS*[168]
5NDJ	14,1G	0.2	0.7	-24.5	0.452	-0.5	0.6	-55.2	0.182	2132	893	415	0.94	0.93	0.85	XRAY	3.1	T. thermophilus HB8		Prokhorova, et al. (2017) *PNAS*[168]
5NDK	1H,13	1.5	0.3	-23.3	0.790	-0.8	1.2	-25.9	0.100	2101	854	409	0.93	0.99	0.81	XRAY	3.0	T. thermophilus HB8		Prokhorova, et al. (2017) *PNAS*[168]
5NDK	14,1G	0.2	0.7	-27.0	0.328	-0.5	0.6	-46.2	0.102	2136	895	414	0.93	0.91	0.85	XRAY	3.0	T. thermophilus HB8		Prokhorova, et al. (2017) *PNAS*[168]
5NDV	5,6	6.6	4.2	56.1	0.461	11.0	4.7	44.2	0.840	1379	588	270	1.24	1.20	1.12	XRAY	3.3	S. cerevisiae S288C		Prokhorova, et al. (2017) *PNAS*[168]
5NDV	1,2	4.0	3.6	83.8	1.253	13.7	7.6	70.0	0.080	1376	606	257	1.25	1.17	1.10	XRAY	3.3	S. cerevisiae S288C		Prokhorova, et al. (2017) *PNAS*[168]
5NDW	1,2	4.2	3.5	62.8	0.872	12.4	5.6	69.6	0.400	1378	613	260	1.25	1.18	1.10	XRAY	3.7	S. cerevisiae S288C		Prokhorova, et al. (2017) *PNAS*[168]
5NDW	5,6	4.2	3.2	76.6	1.173	12.9	6.7	65.4	0.378	1390	606	258	1.26	1.14	1.10	XRAY	3.7	S. cerevisiae S288C		Prokhorova, et al. (2017) *PNAS*[168]
5NGM	AA,Aa	7.8	1.6	75.5	0.931	5.5	4.7	-40.1	1.812	2221	784	355	1.02	1.16	1.00	EM	2.9	Staph. aureus		Matzov, et al. (2017) *NComm*[169]
5NJT	U,A	-1.5	1.7	12.8	0.659	2.0	1.3	-88.1	1.504	2338	891	416	1.04	1.09	0.98	EM	3.8	B. subtilis subsp. subtilis str. 168		Beckert, et al. (2017) *EMBO J*[170]
5NP6	Y,D	-1.5	1.4	17.9	1.860	1.4	1.9	-95.6	1.889	2358	909	416	1.23	1.20	1.04	EM	3.6	E. coli		Agirrezabala, et al. (2017) *Sci Adv*[171]
5NWY	N,0	-1.6	1.5	11.7	0.480	1.2	1.8	-100.4	1.149	2643	1007	439	0.60	0.71	0.71	EM	2.9	E. coli K-12		Su, et al. (2017) *Elife*[172]
5O2R	A,a	-0.7	0.9	5.6	0.378	0.4	1.4	-73.1	0.391	2647	982	440	0.72	0.73	0.82	EM	3.4	E. coli K-12		Florin, et al. (2017) *NSMB*[173]
5O61	A,BA	0.2	1.4	-14.2	0.977	-0.1	1.7	-71.3	1.169	2296	911	411	1.07	1.05	0.90	EM	3.3	Mycolicibacterium smegmatis MC2 155		Hentschel, et al. (2017) *Cell Rep*[174]
5OBM	5,6	7.5	4.9	52.8	0.774	11.3	4.8	52.4	0.832	1399	600	268	1.25	1.21	1.11	XRAY	3.4	S. cerevisiae S288C		Prokhorova, et al. (2017) *PNAS*[168]
5OBM	1,2	3.7	3.1	84.7	1.120	13.6	7.5	64.3	0.545	1400	618	259	1.24	1.17	1.12	XRAY	3.4	S. cerevisiae S288C		Prokhorova, et al. (2017) *PNAS*[168]
5ON6	1,A	4.1	3.0	87.1	1.046	14.1	8.5	65.1	0.479	1394	607	258	1.26	1.18	1.10	XRAY	3.1	S. cerevisiae S288C		Pellegrino, et al. (2018) *Structure*[175]
5ON6	AR,6	7.9	4.8	55.1	0.493	10.1	3.0	35.7	0.339	1399	587	262	1.26	1.17	1.12	XRAY	3.1	S. cerevisiae S288C		Pellegrino, et al. (2018) *Structure*[175]
5OT7	4,1	2.7	1.3	74.2	0.762	2.4	1.3	-45.0	0.656	2086	804	211	1.10	1.16	1.35	EM	3.8	T. thermophilus HB8		Mace, et al. (2018) *NAR*[176]
5T2C	A,AA	-1.5	3.1	24.7	1.937	-0.6	1.1	-88.9	1.491	1100	498	222	1.37	1.33	1.30	EM	3.6	Homo sapiens		Zhang, et al. (2016) *NComm*[177]
5T7V	B,A	0.7	1.4	27.7	2.790	-1.3	1.6	-95.7	3.392	1370	534	198	1.43	1.37	1.33	EM	3.6	Staph. aureus		Belousoff, et al. (2017) *mBio*[178]
5TBW	1,A	4.3	3.1	86.5	1.036	14.0	8.7	64.3	0.433	1279	584	254	1.28	1.20	1.11	XRAY	3.0	S. cerevisiae S288C		Konst, et al. (2017) *Nat Chem*[179]
5TBW	AR,sR	7.6	5.0	53.4	0.736	10.2	2.7	34.4	0.444	1283	579	264	1.28	1.21	1.15	XRAY	3.0	S. cerevisiae S288C		Konst, et al. (2017) *Nat Chem*[179]
5TCU	B,A	1.4	1.2	17.1	2.125	-1.3	0.9	-84.1	2.307	1641	630	352	1.37	1.31	1.23	EM	3.9	Staph. aureus subsp. aureus NCTC 8325		Belousoff, et al. (2017) *mBio*[178]
5TGA	1,2	4.2	2.9	87.3	0.752	13.9	8.5	62.6	0.550	1397	599	259	1.24	1.17	1.11	XRAY	3.3	S. cerevisiae		Melnikov, et al. (2016) *EMBO Rep*[156]
5TGA	5,6	7.8	4.8	53.6	0.485	9.9	3.0	27.1	0.720	1402	588	266	1.25	1.19	1.14	XRAY	3.3	S. cerevisiae		Melnikov, et al. (2016) *EMBO Rep*[156]
5TGM	1,2	4.4	3.1	89.0	1.144	13.7	8.6	63.7	0.738	1394	609	257	1.24	1.18	1.12	XRAY	3.5	S. cerevisiae		Melnikov, et al. (2016) *EMBO Rep*[156]
5TGM	5,6	7.9	4.8	54.9	0.520	9.9	2.9	23.3	0.656	1390	582	265	1.24	1.18	1.13	XRAY	3.5	S. cerevisiae		Melnikov, et al. (2016) *EMBO Rep*[156]
5U4I	A,a	-1.5	1.3	19.1	1.350	1.1	1.9	-92.7	1.075	2464	867	418	1.04	1.13	1.06	EM	3.5	E. coli		Zeng, et al. (2017) *Nature*[180]
5U9F	01,A	-1.5	1.3	11.6	0.503	1.0	1.8	-94.5	0.694	2631	979	435	0.79	0.94	0.93	EM	3.2	E. coli		Demo, et al. (2017) *Elife*[181]
5U9G	01,A	-1.7	1.2	10.4	0.489	1.3	1.8	-95.1	0.701	2606	968	434	0.78	0.97	0.89	EM	3.2	E. coli		Demo, et al. (2017) *Elife*[181]
5UQ7	A,a	-0.5	0.9	21.1	0.788	1.1	1.1	-81.5	1.287	1943	815	404	1.14	1.07	0.99	EM	3.5	T. thermophilus HB8		Zhang, et al. (2018) *Structure*[182]
5UQ8	A,a	-0.5	1.0	22.7	0.783	1.2	1.2	-88.5	1.239	1946	823	404	1.13	1.06	0.98	EM	3.2	T. thermophilus HB8		Zhang, et al. (2018) *Structure*[182]
5UYK	01,A	-1.6	1.0	9.8	0.446	1.2	1.8	-84.4	0.817	2626	994	442	0.81	0.89	0.95	EM	3.9	E. coli K-12		Loveland, et al. (2017) *Nature*[183]
5UYL	01,A	-1.6	0.9	11.2	0.410	1.2	1.7	-89.1	0.819	2636	995	448	0.76	0.86	0.89	EM	3.6	E. coli K-12		Loveland, et al. (2017) *Nature*[183]
5UYM	01,A	0.9	0.8	8.6	0.383	-0.9	1.5	-56.2	0.374	2624	986	446	0.69	0.93	0.80	EM	3.2	E. coli K-12		Loveland, et al. (2017) *Nature*[183]
5UYN	01,A	-1.6	1.0	15.4	0.239	1.2	1.8	-92.2	0.709	2635	997	443	0.83	0.89	0.95	EM	4.0	E. coli K-12		Loveland, et al. (2017) *Nature*[183]
5UYP	01,A	-1.6	1.0	12.3	0.268	1.4	1.9	-90.9	0.682	2637	990	445	0.78	0.89	0.90	EM	3.9	E. coli K-12		Loveland, et al. (2017) *Nature*[183]
5UYQ	01,A	0.2	0.7	2.7	0.142	-0.2	1.4	-60.0	0.289	2608	975	448	0.77	0.90	0.87	EM	3.8	E. coli K-12		Loveland, et al. (2017) *Nature*[183]
5V8I	1A,1a	1.2	0.7	2.1	0.384	-1.2	1.4	-61.4	0.737	2122	855	418	0.99	0.97	0.87	XRAY	3.2	T. thermophilus HB27		Murphy, et al. *To be published*
5V8I	2A,2a	-0.9	0.9	-6.2	0.888	1.2	1.5	-80.8	0.514	2178	871	419	1.01	1.04	0.94	XRAY	3.2	T. thermophilus HB27		Murphy, et al. *To be published*
5V93	A,a	0.6	1.4	-5.2	0.777	0.6	1.6	-62.2	0.553	2192	851	389	1.19	1.14	1.15	EM	4.0	Mycobacterium tuberculosis		Yang, et al. (2017) *NAR*[184]
5VP2	1A,1a	1.4	0.3	0.8	0.607	-0.8	1.3	-31.5	0.175	2108	852	415	0.97	0.97	0.85	XRAY	2.8	T. thermophilus HB8		Osterman, et al. (2017) *NAR*[185]
5VP2	2A,2a	0.0	0.9	-19.8	0.229	0.1	1.0	-38.3	0.200	2110	887	408	0.97	0.94	0.89	XRAY	2.8	T. thermophilus HB8		Osterman, et al. (2017) *NAR*[185]
5VPO	YA,XA	1.5	0.8	11.9	0.207	0.4	1.6	-18.0	0.251	2101	869	413	0.96	0.97	0.86	XRAY	3.3	T. thermophilus HB8		Hong, et al. (2018) *PNAS*[186]
5VPO	RA,QA	-0.9	1.0	-2.9	0.460	1.8	0.8	-42.4	0.596	2128	890	416	0.95	0.95	0.92	XRAY	3.3	T. thermophilus HB8		Hong, et al. (2018) *PNAS*[186]

Table 13 of 24

PDB		BODY				HEAD				PRUNED			RMSD			EXP DETAILS		ORGANISM		REFERENCE
ID	chains	ϕ_{body}	θ_{body}	ψ_{body}	$\lvert\Delta\vec{x}_{\text{body}}\rvert$	ϕ_{head}	θ_{head}	ψ_{head}	$\lvert\Delta\vec{x}_{\text{head}}\rvert$	LSU	body	head	LSU	body	head	method	res.	name	mito	
5VPP	YA,XA	0.8	1.0	5.1	0.252	18.5	4.2	45.4	1.695	2103	866	336	0.98	1.04	1.10	XRAY	3.9	T. thermophilus HB8		Hong, et al. (2018) *PNAS*[186]
5VPP	RA,QA	-0.3	1.4	1.1	0.622	18.3	4.4	50.7	1.967	2120	867	340	0.98	1.02	1.14	XRAY	3.9	T. thermophilus HB8		Hong, et al. (2018) *PNAS*[186]
5W4K	1A,1a	1.4	0.3	-20.0	0.634	-0.8	1.4	-30.5	0.066	2097	856	411	0.96	0.97	0.81	XRAY	2.7	T. thermophilus HB8		Metelev, et al. (2017) *NCB*[187]
5W4K	2A,2a	0.0	0.8	-23.7	0.251	-0.1	1.1	-35.4	0.037	2113	888	409	0.95	0.90	0.84	XRAY	2.7	T. thermophilus HB8		Metelev, et al. (2017) *NCB*[187]
5WDT	A,a	1.0	0.7	7.9	0.274	-0.7	1.3	-46.0	0.422	2583	963	420	0.68	0.93	0.82	EM	3.0	E. coli		Fislage, et al. (2018) *NAR*[188]
5WE4	A,a	0.6	0.6	4.7	0.290	-0.6	1.4	-45.9	0.342	2599	970	421	0.63	0.88	0.77	EM	3.1	E. coli		Fislage, et al. (2018) *NAR*[188]
5WE6	A,a	0.5	0.8	11.7	0.150	-0.5	1.2	-49.9	0.201	2578	940	407	0.70	0.91	0.91	EM	3.4	E. coli		Fislage, et al. (2018) *NAR*[188]
5WF0	A,a	-1.5	1.2	13.1	0.395	1.2	1.6	-92.8	0.946	2594	988	425	0.67	0.81	0.80	EM	3.6	E. coli		Fislage, et al. (2018) *NAR*[188]
5WFK	A,a	-1.7	1.2	10.9	0.425	0.9	1.6	-94.1	0.919	2587	984	426	0.70	0.84	0.84	EM	3.4	E. coli		Fislage, et al. (2018) *NAR*[188]
5WFS	A,a	0.4	0.6	4.5	0.195	-0.3	1.4	-48.6	0.343	2597	955	423	0.62	0.91	0.76	EM	3.0	E. coli		Fislage, et al. (2018) *NAR*[188]
5WIS	1A,1a	1.3	0.3	-21.4	0.540	-0.8	1.4	-29.3	0.127	2098	855	419	0.97	0.97	0.85	XRAY	2.7	T. thermophilus HB8		Almutairi, et al. (2017) *NAR*[189]
5WIS	2A,2a	-0.1	0.8	-22.2	0.245	0.1	1.0	-40.6	0.167	2111	890	411	0.95	0.91	0.85	XRAY	2.7	T. thermophilus HB8		Almutairi, et al. (2017) *NAR*[189]
5WIT	1A,1a	1.2	0.3	-47.4	0.559	-0.6	1.4	-28.5	0.253	2087	854	418	0.98	0.99	0.85	XRAY	2.6	T. thermophilus HB8		Almutairi, et al. (2017) *NAR*[189]
5WIT	2A,2a	0.1	0.6	-36.3	0.243	-0.2	1.2	-35.7	0.223	2101	883	419	0.96	0.93	0.90	XRAY	2.6	T. thermophilus HB8		Almutairi, et al. (2017) *NAR*[189]
5X8P	A,a	-0.7	1.1	49.2	0.934	1.3	1.0	-73.0	1.222	2104	895	419	1.03	1.04	0.90	EM	3.4	Spinacia oleracea		Ahmed, et al. (2017) *NAR*[190]
5ZEB	A,a	0.2	1.4	-15.7	0.409	-0.1	1.7	-67.3	1.124	2347	908	411	0.97	1.03	0.91	EM	3.4	Mycolicibacterium smegmatis MC2 155		Mishra, et al. (2018) *Sci Rep*[191]
5ZEP	A,a	0.1	1.4	-8.2	0.953	-0.1	1.7	-67.2	1.127	2346	908	411	0.97	1.03	0.91	EM	3.4	Mycolicibacterium smegmatis MC2 155		Mishra, et al. (2018) *Sci Rep*[191]
5ZLU	V,G	0.8	2.0	49.1	5.759	2.8	1.7	-68.2	3.910	502	300	230	1.47	1.49	1.40	EM	3.6	T. thermophilus HB8		Su, et al. (2018) *PNAS*[192]
6B4V	B,A	0.2	0.8	2.2	0.741	0.3	1.7	-53.3	0.760	2121	888	393	0.99	0.99	0.97	XRAY	3.4	T. thermophilus HB27		Svidritskiy, et al. (2018) *JMB*[193]
6B4V	FB,EB	-0.5	1.3	3.4	1.218	1.0	1.5	-70.8	0.891	2122	887	393	0.98	0.98	0.97	XRAY	3.4	T. thermophilus HB27		Svidritskiy, et al. (2018) *JMB*[193]
6BOH	B,A	0.0	0.2	-64.6	0.319	0.9	1.8	-39.4	0.280	2114	890	401	0.94	0.90	0.95	XRAY	3.4	T. thermophilus HB27		Svidritskiy, et al. (2018) *Structure*[194]
6BOH	GB,FB	-0.8	1.0	-3.3	0.299	1.6	1.4	-50.7	0.193	2109	889	400	0.93	0.90	0.94	XRAY	3.4	T. thermophilus HB27		Svidritskiy, et al. (2018) *Structure*[194]
6BOK	B,A	0.1	0.3	-82.5	0.617	0.3	1.9	-57.0	0.262	2117	901	403	0.93	0.89	0.89	XRAY	3.5	T. thermophilus HB27		Svidritskiy, et al. (2018) *Structure*[194]
6BOK	EB,DB	-0.5	0.6	-31.4	0.090	0.7	1.9	-71.9	0.453	2117	903	403	0.92	0.90	0.89	XRAY	3.5	T. thermophilus HB27		Svidritskiy, et al. (2018) *Structure*[194]
6BU8	01,A	0.7	0.6	0.7	0.344	-0.4	1.4	-44.5	0.323	2606	971	445	0.84	0.99	0.93	EM	3.5	E. coli K-12		Loveland, et al. (2018) *Methods*[195]
6BUW	YA,XA	1.7	0.1	-83.8	0.769	-1.2	1.4	-30.3	0.243	2094	869	410	0.96	1.02	0.89	XRAY	3.5	T. thermophilus HB8		Hoffer, et al. (2019) *NAR*[196]
6BUW	RA,QA	-0.6	0.5	-25.7	0.313	0.8	1.2	-55.3	0.290	2121	888	418	0.94	0.94	0.94	XRAY	3.5	T. thermophilus HB8		Hoffer, et al. (2019) *NAR*[196]
6BY1	DA,BA	2.0	0.1	20.8	0.142	-0.8	1.3	-16.7	0.559	2641	950	421	0.92	1.13	1.05	XRAY	3.9	E. coli K-12		Amiri, et al. (2019) *RNA*[197]
6BY1	CA,AA	1.4	0.7	4.8	0.046	-0.4	1.4	-27.3	0.423	2624	919	429	0.86	1.06	1.07	XRAY	3.9	E. coli K-12		Amiri, et al. (2019) *RNA*[197]
6BZ6	YA,XA	1.7	0.2	-135.8	0.220	-1.1	1.6	-35.4	0.227	2087	863	417	0.97	1.00	0.88	XRAY	3.2	T. thermophilus HB8		Hoffer, et al. (2019) *NAR*[196]
6BZ6	RA,QA	-0.3	0.3	-21.4	0.160	0.5	1.4	-56.6	0.395	2114	862	418	0.95	0.94	0.96	XRAY	3.2	T. thermophilus HB8		Hoffer, et al. (2019) *NAR*[196]
6BZ7	YA,XA	1.4	0.2	-148.2	0.232	-0.8	1.2	-19.5	0.375	2079	849	411	1.01	1.06	0.93	XRAY	3.7	T. thermophilus HB8		Hoffer, et al. (2019) *NAR*[196]
6BZ7	RA,QA	0.2	0.3	-66.3	0.333	-0.5	1.3	-51.1	0.172	2126	867	410	0.97	1.02	0.95	XRAY	3.7	T. thermophilus HB8		Hoffer, et al. (2019) *NAR*[196]
6BZ8	YA,XA	1.5	0.2	-158.6	0.148	-1.0	1.4	-30.4	0.304	2095	854	413	0.99	1.03	0.93	XRAY	3.7	T. thermophilus HB8		Hoffer, et al. (2019) *NAR*[196]
6BZ8	RA,QA	0.4	0.5	-14.2	0.368	-0.6	1.1	-54.8	0.274	2130	862	413	0.98	0.97	0.93	XRAY	3.7	T. thermophilus HB8		Hoffer, et al. (2019) *NAR*[196]
6C4I	A,a	-1.6	1.7	10.5	0.234	1.2	2.1	-88.8	0.622	2667	1000	440	0.70	0.71	0.80	EM	3.2	E. coli		Zeng, et al. (2018) *Sci Rep*[198]
6C5L	BA,AA	-0.5	0.7	-23.9	0.621	1.0	1.4	-49.3	0.440	2114	881	402	0.99	1.01	1.04	XRAY	3.2	T. thermophilus		Zeng, et al. (2018) *Sci Rep*[198]
6C5L	DA,CA	0.3	0.4	-16.1	0.227	0.4	1.5	-38.9	0.560	2084	883	405	1.01	1.03	1.01	XRAY	3.2	T. thermophilus		Zeng, et al. (2018) *Sci Rep*[198]
6CAE	1A,1a	1.4	0.2	-15.4	0.792	-0.7	1.8	-44.2	0.163	2104	874	419	0.94	0.96	0.82	XRAY	2.6	T. thermophilus HB8		Pantel, et al. (2018) *Mol Cell*[199]
6CAE	2A,2a	-0.3	0.7	-20.4	0.205	0.6	1.6	-49.4	0.086	2128	890	415	0.94	0.89	0.86	XRAY	2.6	T. thermophilus HB8		Pantel, et al. (2018) *Mol Cell*[199]
6CFJ	1A,1a	1.4	0.3	-25.8	0.606	-0.8	1.5	-32.1	0.080	2096	859	422	0.97	0.97	0.87	XRAY	2.8	T. thermophilus HB8		Tereshchenkov, et al. (2018) *JMB*[200]
6CFJ	2A,2a	0.0	0.8	-25.2	0.191	-0.1	1.1	-30.1	0.137	2123	887	410	0.96	0.92	0.88	XRAY	2.8	T. thermophilus HB8		Tereshchenkov, et al. (2018) *JMB*[200]
6CFK	1A,1a	1.6	0.7	5.7	0.365	-1.4	1.3	-58.4	0.725	2102	849	411	0.95	0.98	0.85	XRAY	2.7	T. thermophilus HB8		Tereshchenkov, et al. (2018) *JMB*[200]
6CFK	2A,2a	-1.0	1.1	-3.9	0.567	1.1	1.2	-79.9	0.431	2163	881	410	0.95	0.98	0.92	XRAY	2.7	T. thermophilus HB8		Tereshchenkov, et al. (2018) *JMB*[200]
6CFL	1A,1a	1.6	0.7	5.1	0.300	-1.4	1.3	-60.4	0.737	2094	841	413	0.97	0.99	0.87	XRAY	2.6	T. thermophilus HB8		Tereshchenkov, et al. (2018) *JMB*[200]
6CFL	2A,2a	-1.0	1.1	-2.5	0.754	1.2	1.3	-83.0	0.464	2155	886	415	0.96	1.00	0.95	XRAY	2.6	T. thermophilus HB8		Tereshchenkov, et al. (2018) *JMB*[200]
6CZR	1A,1a	1.3	0.9	12.8	0.342	1.4	2.2	-28.9	0.327	2107	873	413	0.97	0.99	0.98	XRAY	3.1	T. thermophilus HB8		Serrano, et al. (2020) *ACIEE*
6CZR	2A,2a	-0.6	1.2	-2.8	0.218	1.8	0.7	-28.6	0.108	2157	883	409	0.98	1.00	0.99	XRAY	3.1	T. thermophilus HB8		Serrano, et al. (2020) *ACIEE*
6D90	5,2	-2.2	3.1	12.9	3.966	-0.5	1.9	-85.7	3.055	696	379	236	1.47	1.42	1.33	EM	3.2	Oryctolagus cuniculus		Pisareva, et al. (2018) *Elife*[201]
6D9J	5,2	-0.7	2.9	23.0	3.629	16.7	4.3	55.1	3.713	565	307	176	1.49	1.45	1.39	EM	3.2	Oryctolagus cuniculus		Pisareva, et al. (2018) *Elife*[201]
6DNC	B,A	-1.0	1.0	1.9	0.445	0.4	2.0	-76.6	0.557	2649	979	442	0.80	0.82	0.86	EM	3.7	E. coli		Svidritskiy, et al. (2018) *J Biol Chem*[202]
6DZI	A,h	-0.6	1.5	2.4	1.550	0.0	1.7	-104.4	0.922	2176	823	361	1.18	1.22	1.17	EM	3.5	Mycolicibacterium smegmatis MC2 155		Li, et al. (2018) *PNAS*[203]

PDB		BODY				HEAD				PRUNED			RMSD			EXP DETAILS		ORGANISM		REFERENCE
ID	chains	ϕ_{body}	θ_{body}	ψ_{body}	$\lvert\Delta\vec{x}_{\text{body}}\rvert$	ϕ_{head}	θ_{head}	ψ_{head}	$\lvert\Delta\vec{x}_{\text{head}}\rvert$	LSU	body	head	LSU	body	head	method	res.	name	mito	
6ENF	A,a	-1.6	1.4	10.3	0.679	1.1	1.7	-96.7	1.089	2594	973	438	0.86	0.94	0.91	EM	3.2	E. coli		Huter, et al. (2017) *Mol Cell*[204]
6ENJ	A,a	0.4	0.8	0.3	0.183	-0.2	1.2	-53.6	0.448	2621	869	439	0.85	0.97	0.87	EM	3.7	E. coli		Huter, et al. (2017) *Mol Cell*[204]
6ENU	A,a	-1.4	1.4	10.0	0.591	0.9	1.6	-98.8	1.142	2617	982	440	0.70	0.80	0.77	EM	3.1	E. coli		Huter, et al. (2017) *Mol Cell*[204]
6ERI	AA,BA	-0.5	1.3	43.7	2.294	0.1	1.9	-94.2	2.393	1991	828	410	1.12	1.21	0.97	EM	3.0	Spinacia oleracea		Boerema, et al. (2018) *Nat Plants*[205]
6FKR	1A,1a	1.4	0.7	10.7	0.415	-1.3	1.2	-60.1	0.630	2109	865	415	0.93	0.92	0.86	XRAY	3.2	T. thermophilus HB8		Mardirossian, et al. (2018) *Cell Chem Biol*[206]
6FKR	2A,2a	-0.8	1.1	-4.2	0.277	0.7	1.1	-79.1	0.117	2158	887	409	0.93	0.94	0.84	XRAY	3.2	T. thermophilus HB8		Mardirossian, et al. (2018) *Cell Chem Biol*[206]
6FXC	AA,Aa	7.7	1.6	76.0	0.933	5.5	4.8	-40.2	1.819	2221	776	355	1.02	1.15	1.00	EM	6.8	Staph. aureus		Matzov, et al. (2017) *NComm*[169]
6FXC	BA,Ba	7.8	1.6	75.9	0.928	5.5	4.7	-40.2	1.825	2221	774	355	1.02	1.15	1.00	EM	6.8	Staph. aureus		Matzov, et al. (2017) *NComm*[169]
6GAW	BA,AA	0.7	1.3	38.3	2.967	1.9	2.5	-137.6	1.763	1047	410	207	0.99	1.08	1.08	EM	3.2	Sus scrofa	*	Kummer, et al. (2018) *Nature*[207]
6GQ1	1,2	7.6	5.8	28.4	2.171	7.4	4.1	60.5	1.586	1231	518	197	1.35	1.31	1.39	EM	4.4	S. cerevisiae S288C		Pellegrino, et al. (2018) *JMB*[208]
6GQB	1,2	7.6	5.8	28.4	2.098	2.7	1.8	-40.1	1.739	1216	544	228	1.34	1.31	1.33	EM	3.9	S. cerevisiae S288C		Pellegrino, et al. (2018) *JMB*[208]
6GQV	1,2	7.6	6.1	27.3	2.340	7.8	4.3	63.8	1.805	1156	525	167	1.36	1.34	1.44	EM	4.0	S. cerevisiae		Pellegrino, et al. (2018) *JMB*[208]
6GSJ	1H,13	1.4	0.3	-130.8	0.085	-0.8	1.5	-19.1	0.151	2088	867	414	0.93	0.96	0.83	XRAY	3.0	T. thermophilus HB8		Rozov, et al. (2018) *NAR*[209]
6GSJ	14,1G	-0.1	0.6	-45.6	0.101	-0.1	0.9	-35.8	0.188	2113	894	409	0.93	0.89	0.88	XRAY	3.0	T. thermophilus HB8		Rozov, et al. (2018) *NAR*[209]
6GSK	1H,13	1.5	0.2	-112.1	0.333	-0.8	1.5	-19.6	0.185	2100	868	414	0.94	0.96	0.87	XRAY	3.4	T. thermophilus HB8		Rozov, et al. (2018) *NAR*[209]
6GSK	14,1G	-0.6	0.6	-34.2	0.108	0.7	1.1	-46.0	0.347	2125	890	403	0.94	0.92	0.95	XRAY	3.4	T. thermophilus HB8		Rozov, et al. (2018) *NAR*[209]
6GSL	1H,13	1.4	0.2	-81.8	0.571	-0.9	1.5	-27.2	0.234	2117	868	414	0.95	0.97	0.88	XRAY	3.2	T. thermophilus HB8		Rozov, et al. (2018) *NAR*[209]
6GSL	14,1G	-0.3	0.7	-38.3	0.154	-0.1	1.0	-47.3	0.412	2123	888	406	0.94	0.90	0.93	XRAY	3.2	T. thermophilus HB8		Rozov, et al. (2018) *NAR*[209]
6GWT	A,a	0.2	0.5	-21.3	0.407	0.5	1.6	-37.1	0.523	2596	967	441	0.91	0.98	0.92	EM	3.8	E. coli		Graf, et al. (2018) *NComm*[210]
6GXM	A,a	1.3	0.8	-0.8	0.197	0.9	2.1	-34.7	0.541	2590	959	442	0.92	1.04	0.94	EM	3.8	E. coli		Graf, et al. (2018) *NComm*[210]
6GXN	A,a	4.6	2.1	131.0	1.210	1.0	2.1	-32.0	0.573	2590	921	442	0.92	1.11	0.93	EM	3.9	E. coli		Graf, et al. (2018) *NComm*[210]
6GXO	A,a	8.9	1.7	30.8	0.935	4.2	2.0	-37.0	1.039	2549	894	402	0.92	1.12	1.08	EM	3.9	E. coli		Graf, et al. (2018) *NComm*[210]
6GXP	A,a	7.8	1.7	114.1	1.314	3.8	3.8	-63.1	1.057	2550	902	390	0.94	1.12	1.14	EM	4.4	E. coli		Graf, et al. (2018) *NComm*[210]
6GZ3	A2,B1	2.0	3.3	36.0	1.504	16.6	6.1	67.9	0.783	1368	587	251	1.26	1.23	1.24	EM	3.6	Oryctolagus cuniculus		Flis, et al. (2018) *Cell Rep*[211]
6GZ4	A2,B1	-1.5	3.3	19.2	1.140	18.3	5.3	68.8	0.705	1377	591	253	1.24	1.22	1.22	EM	3.6	Oryctolagus cuniculus		Flis, et al. (2018) *Cell Rep*[211]
6GZ5	A2,B1	-2.0	3.0	22.1	1.388	-0.8	1.9	-66.4	1.647	1382	588	266	1.26	1.27	1.22	EM	3.5	Oryctolagus cuniculus		Flis, et al. (2018) *Cell Rep*[211]
6GZQ	A1,A2	-0.3	1.1	28.1	2.808	1.2	0.6	-71.5	2.081	1384	592	313	1.41	1.36	1.27	EM	3.3	T. thermophilus HB8		Flygaard, et al. (2018) *NComm*[212]
6GZX	A1,A3	-0.4	1.3	27.9	2.480	1.3	0.5	-115.3	2.424	1052	431	196	1.44	1.41	1.41	EM	4.6	T. thermophilus HB8		Flygaard, et al. (2018) *NComm*[212]
6GZX	A2,A4	-0.4	1.1	26.2	2.446	1.4	0.8	-73.9	2.296	1024	438	185	1.45	1.41	1.44	EM	4.6	T. thermophilus HB8		Flygaard, et al. (2018) *NComm*[212]
6GZZ	A1,A3	3.7	1.4	99.6	3.667	2.3	3.1	-29.0	1.891	1282	531	213	1.41	1.31	1.39	EM	4.1	T. thermophilus HB8		Flygaard, et al. (2018) *NComm*[212]
6GZZ	A2,A4	3.8	1.2	103.1	3.690	1.8	3.0	-29.0	1.838	1275	519	219	1.40	1.29	1.38	EM	4.1	T. thermophilus HB8		Flygaard, et al. (2018) *NComm*[212]
6H4N	A,a	-1.6	1.8	15.9	0.565	0.3	2.2	-110.6	1.116	2611	989	429	0.72	0.78	0.80	EM	3.0	E. coli BW25113		Beckert, et al. (2018) *NMb*[213]
6H58	A,a	-1.6	1.8	15.9	0.565	0.3	2.2	-110.6	1.116	2611	989	429	0.72	0.78	0.80	EM	7.9	E. coli BW25113		Beckert, et al. (2018) *NMb*[213]
6H58	AA,aa	-1.6	1.8	15.9	0.565	0.3	2.2	-110.6	1.115	2611	989	429	0.72	0.78	0.80	EM	7.9	E. coli BW25113		Beckert, et al. (2018) *NMb*[213]
6HA1	A,a	-1.6	1.9	12.5	0.314	1.2	1.6	-116.5	1.355	2355	912	422	0.96	0.96	0.83	EM	3.1	B. subtilis subsp. subtilis str. 168		Crowe-McAuliffe, et al. (2018) *PNAS*[214]
6HA8	A,a	0.9	3.1	48.4	1.419	2.2	1.6	-121.4	0.685	2299	874	419	0.97	1.10	0.88	EM	3.5	B. subtilis subsp. subtilis str. 168		Crowe-McAuliffe, et al. (2018) *PNAS*[214]
6HCF	52,A1	-1.6	2.7	21.0	3.488	-1.1	1.4	-81.4	2.719	698	389	229	1.48	1.39	1.36	EM	3.9	Oryctolagus cuniculus		Juszkiewicz, et al. (2018) *Mol Cell*[215]
6HCJ	51,A2	8.8	5.8	38.8	2.993	1.2	0.8	5.8	2.311	663	398	199	1.47	1.42	1.36	EM	3.8	Oryctolagus cuniculus		Juszkiewicz, et al. (2018) *Mol Cell*[215]
6HCM	52,A1	-1.6	2.7	21.0	3.488	-1.1	1.4	-81.4	2.719	698	389	229	1.48	1.39	1.36	EM	6.8	Oryctolagus cuniculus		Juszkiewicz, et al. (2018) *Mol Cell*[215]
6HCQ	51,A2	8.8	5.8	38.8	2.993	1.2	0.8	5.8	2.311	663	398	199	1.47	1.42	1.36	EM	6.5	Oryctolagus cuniculus		Juszkiewicz, et al. (2018) *Mol Cell*[215]
6HHQ	1,A	4.5	3.0	87.2	0.986	13.9	8.7	64.5	0.380	1395	601	256	1.25	1.18	1.11	XRAY	3.1	S. cerevisiae		Pellegrino, et al. (2019) *NAR*[216]
6HHQ	AR,sR	7.7	4.9	53.7	0.508	10.2	2.7	32.6	0.628	1394	588	263	1.25	1.18	1.12	XRAY	3.1	S. cerevisiae		Pellegrino, et al. (2019) *NAR*[216]
6HTQ	A,a	-0.8	1.6	3.8	0.575	-0.2	0.9	-133.6	1.168	2288	911	412	1.08	1.00	0.93	EM	4.5	B. subtilis subsp. subtilis str. 168		Pausch, et al. (2020) *Cell Rep*[217]
6I7O	BQ,2	-1.9	2.8	18.4	1.338	-0.2	1.8	-62.8	1.193	1387	601	276	1.27	1.21	1.18	EM	5.3	S. cerevisiae		Ikeuchi, et al. (2019) *EMBO J*[218]
6I7O	YQ,2b	9.0	6.0	33.0	1.023	0.6	1.3	-32.9	1.178	1363	577	281	1.28	1.22	1.21	EM	5.3	S. cerevisiae		Ikeuchi, et al. (2019) *EMBO J*[218]
6I7V	DA,AA	5.7	1.2	122.8	1.012	9.6	2.0	42.2	0.288	2694	949	394	0.59	1.09	0.77	XRAY	2.9	E. coli		Lilleorg, et al. (2018) *Biochimie*[219]
6I7V	CA,BA	-0.7	0.8	18.5	0.599	5.2	1.9	66.6	0.725	2761	1028	431	0.80	0.72	0.96	XRAY	2.9	E. coli		Lilleorg, et al. (2018) *Biochimie*[219]
6IP5	1A,2m	-2.1	2.9	19.1	2.094	-0.8	1.1	-79.0	1.650	1201	552	266	1.33	1.31	1.27	EM	3.9	Homo sapiens		Yokoyama, et al. (2019) *Mol Cell*[220]
6IP6	1A,2m	-2.0	2.9	24.3	2.098	-0.7	1.0	-82.9	1.882	1198	546	263	1.33	1.29	1.28	EM	4.5	Homo sapiens		Yokoyama, et al. (2019) *Mol Cell*[220]
6IP8	1A,2m	-2.2	3.1	19.1	2.122	-1.2	1.0	-83.9	1.758	1185	562	276	1.31	1.30	1.29	EM	3.9	Homo sapiens		Yokoyama, et al. (2019) *Mol Cell*[220]
6LKQ	t,s	7.3	2.6	43.8	0.923	13.5	3.3	71.2	0.630	2584	963	377	0.77	0.95	0.95	XRAY	3.1	E. coli		Zhang, et al. (2020) *PNAS*[221]
6MTB	5,9	-2.1	3.1	21.8	1.323	-1.1	1.3	-81.9	1.287	1424	607	260	1.21	1.19	1.14	EM	3.6	Oryctolagus cuniculus		Brown, et al. (2018) *Elife*[222]

PDB		BODY				HEAD				PRUNED			RMSD			EXP DETAILS		ORGANISM		REFERENCE
ID	chains	ϕ_{body}	θ_{body}	ψ_{body}	$\lvert\Delta\vec{x}_{\text{body}}\rvert$	ϕ_{head}	θ_{head}	ψ_{head}	$\lvert\Delta\vec{x}_{\text{head}}\rvert$	LSU	body	head	LSU	body	head	method	res.	name	mito	
6MTC	5,9	-2.2	3.1	22.5	1.373	-1.1	1.8	-86.5	1.077	1422	613	266	1.22	1.20	1.13	EM	3.4	Oryctolagus cuniculus		Brown, et al. (2018) *Elife*[222]
6MTD	5,9	-1.2	3.4	21.2	0.950	16.1	4.7	70.0	0.561	1408	606	255	1.21	1.20	1.14	EM	3.3	Oryctolagus cuniculus		Brown, et al. (2018) *Elife*[222]
6MTE	5,9	5.6	4.7	41.7	0.841	12.5	7.6	71.1	0.222	1401	607	224	1.21	1.25	1.19	EM	3.4	Oryctolagus cuniculus		Brown, et al. (2018) *Elife*[222]
6N1D	A23S,A16S	1.8	0.9	29.2	0.934	18.4	4.5	23.0	1.237	1998	831	341	1.12	1.05	1.10	XRAY	3.2	T. thermophilus HB27		Zhou, et al. (2019) *PNAS*[223]
6N1D	B23S,B16S	2.5	1.1	118.4	0.405	18.7	4.9	20.5	0.880	2054	868	336	1.03	1.04	1.14	XRAY	3.2	T. thermophilus HB27		Zhou, et al. (2019) *PNAS*[223]
6N9E	1A,1a	0.5	0.4	-90.8	0.782	0.0	1.1	-20.9	0.369	2072	869	420	1.01	1.01	0.95	XRAY	3.7	T. thermophilus		Melnikov, et al. (2019) *NAR*[224]
6N9E	2A,2a	-1.0	0.9	-19.8	0.729	1.3	0.8	-64.6	0.692	2133	889	414	0.99	1.04	0.95	XRAY	3.7	T. thermophilus		Melnikov, et al. (2019) *NAR*[224]
6N9F	1A,1a	0.4	0.3	-80.7	0.637	0.1	1.0	-22.6	0.102	2095	880	418	0.96	0.94	0.90	XRAY	3.7	T. thermophilus		Melnikov, et al. (2019) *NAR*[224]
6N9F	2A,2a	-1.0	0.9	-17.2	0.310	1.3	0.8	-63.4	0.534	2145	885	416	0.97	0.98	0.93	XRAY	3.7	T. thermophilus		Melnikov, et al. (2019) *NAR*[224]
6ND5	1A,1a	1.4	0.3	-28.1	0.807	-0.8	1.5	-29.2	0.255	2094	871	412	0.94	0.95	0.83	XRAY	2.6	T. thermophilus HB8		Svetlov, et al. (2019) *RNA*[225]
6ND5	2A,2a	-0.3	0.8	-25.5	0.387	0.5	1.2	-40.5	0.235	2124	887	410	0.95	0.87	0.91	XRAY	2.6	T. thermophilus HB8		Svetlov, et al. (2019) *RNA*[225]
6ND6	1A,1a	1.4	0.2	-41.5	0.807	-0.7	1.5	-26.9	0.106	2119	866	415	0.96	0.94	0.86	XRAY	2.9	T. thermophilus HB8		Svetlov, et al. (2019) *RNA*[225]
6ND6	2A,2a	-0.2	0.8	-23.3	0.124	0.2	1.1	-26.5	0.068	2134	897	409	0.94	0.91	0.91	XRAY	2.9	T. thermophilus HB8		Svetlov, et al. (2019) *RNA*[225]
6NDK	RA,QA	-0.1	0.3	-67.2	0.052	0.0	1.3	-49.8	0.130	2115	888	409	0.98	0.99	1.05	XRAY	3.6	T. thermophilus HB8		Nguyen, et al. (2019) *J Biol Chem*[226]
6NDK	YA,XA	1.2	0.4	-135.6	0.476	-0.5	1.6	-30.2	0.124	2080	873	422	1.00	1.02	0.93	XRAY	3.6	T. thermophilus HB8		Nguyen, et al. (2019) *J Biol Chem*[226]
6NSH	YA,XA	1.4	0.9	16.5	0.391	0.3	1.3	-30.5	0.180	2105	866	409	0.95	0.95	0.97	XRAY	3.4	T. thermophilus HB8		Hoffer, et al. (2020) *Elife*[227]
6NSH	RA,QA	-0.8	1.2	-0.8	0.502	1.6	0.8	-49.4	0.488	2140	878	411	0.95	0.99	1.03	XRAY	3.4	T. thermophilus HB8		Hoffer, et al. (2020) *Elife*[227]
6NTA	YA,XA	1.5	0.9	16.8	0.429	0.2	1.5	-28.5	0.093	2102	868	415	0.96	0.97	0.96	XRAY	3.1	T. thermophilus HB8		Hoffer, et al. (2020) *Elife*[227]
6NTA	RA,QA	-0.9	1.2	-0.8	0.487	1.7	1.0	-52.7	0.408	2132	879	404	0.97	1.00	1.01	XRAY	3.1	T. thermophilus HB8		Hoffer, et al. (2020) *Elife*[227]
6NU2	A,AA	7.1	2.1	128.0	2.022	3.2	1.4	-131.8	1.477	799	219	66	1.30	1.39	1.34	EM	3.9	Homo sapiens	*	Koripella, et al. (2019) *PNAS*[228]
6NU3	A,AA	4.7	2.9	-173.5	1.660	2.5	2.1	145.2	3.138	846	128	71	1.24	1.47	1.40	EM	4.4	Homo sapiens	*	Koripella, et al. (2019) *PNAS*[228]
6NUO	YA,XA	0.7	0.6	-12.4	0.367	0.5	1.3	-40.3	0.241	2115	891	411	0.95	0.93	0.97	XRAY	3.2	T. thermophilus HB8		Hoffer, et al. (2020) *Elife*[227]
6NUO	RA,QA	-0.7	1.0	-8.0	0.392	1.4	0.7	-49.9	0.445	2158	885	404	0.98	1.01	1.03	XRAY	3.2	T. thermophilus HB8		Hoffer, et al. (2020) *Elife*[227]
6NWY	YA,XA	0.9	1.0	6.8	0.362	18.7	4.3	44.9	1.771	2087	866	339	0.98	1.00	1.08	XRAY	3.5	T. thermophilus HB8		Hoffer, et al. (2020) *Elife*[227]
6NWY	RA,QA	-0.3	1.5	3.1	0.661	18.6	4.4	50.3	1.940	2113	868	330	1.00	1.02	1.11	XRAY	3.5	T. thermophilus HB8		Hoffer, et al. (2020) *Elife*[227]
6O3M	YA,XA	0.7	0.5	-13.7	0.319	0.7	1.3	-32.1	0.430	2100	884	415	0.99	0.95	0.92	XRAY	4.0	T. thermophilus HB8		Hoffer, et al. (2020) *Elife*[227]
6O3M	RA,QA	-0.6	0.8	-9.5	0.286	1.3	0.8	-50.2	0.575	2123	874	415	0.99	1.00	1.01	XRAY	4.0	T. thermophilus HB8		Hoffer, et al. (2020) *Elife*[227]
6O8W	A,a	0.4	2.4	10.9	3.712	18.4	3.2	41.9	1.898	1033	426	316	1.47	1.37	1.31	EM	3.5	Enterococcus faecalis		Murphy, et al. (2020) *Sci Rep*[229]
6O8X	A,a	-2.0	3.1	53.8	5.270	15.3	2.0	-125.1	2.717	1026	372	268	1.49	1.39	1.32	EM	3.7	Enterococcus faecalis		Murphy, et al. (2020) *Sci Rep*[229]
6O8Y	A,a	-1.7	2.7	36.1	4.938	14.9	0.7	-99.8	3.371	975	366	268	1.49	1.42	1.38	EM	4.1	Enterococcus faecalis		Murphy, et al. (2020) *Sci Rep*[229]
6O8Z	A,a	-1.7	2.0	31.3	4.604	0.7	2.3	-115.6	4.119	1073	395	353	1.48	1.40	1.31	EM	3.5	Enterococcus faecalis		Murphy, et al. (2020) *Sci Rep*[229]
6O90	A,a	2.1	2.3	60.9	4.152	13.6	1.6	14.1	2.288	1033	425	331	1.50	1.37	1.28	EM	3.5	Enterococcus faecalis		Murphy, et al. (2020) *Sci Rep*[229]
6O97	1A,1a	1.5	0.2	-20.2	0.936	-0.8	1.7	-32.1	0.213	2113	853	415	0.97	1.03	0.83	XRAY	2.8	T. thermophilus HB8		Matsushita, et al. (2019) *J Am Chem Soc*[230]
6O97	2A,2a	0.2	0.7	-26.2	0.464	-0.2	1.3	-31.7	0.217	2110	881	412	0.96	0.94	0.86	XRAY	2.8	T. thermophilus HB8		Matsushita, et al. (2019) *J Am Chem Soc*[230]
6O9J	B,a	-2.1	1.9	15.0	1.046	6.0	2.1	142.5	0.595	2701	981	439	0.67	0.93	0.85	EM	3.9	E. coli		Kaledhonkar, et al. (2019) *Nature*[231]
6O9K	A,a	2.3	1.7	56.6	1.688	1.7	1.5	-53.5	1.281	2378	903	391	1.22	1.17	1.13	EM	4.0	E. coli		Kaledhonkar, et al. (2019) *Nature*[231]
6OF1	1A,1a	1.5	0.2	-51.8	0.941	-0.7	1.6	-29.2	0.416	2071	866	410	0.96	0.99	0.81	XRAY	2.8	T. thermophilus HB8		Khabibullina, et al. (2019) *AAC*[232]
6OF1	2A,2a	0.0	0.7	-32.5	0.713	0.1	1.3	-34.4	0.431	2103	891	414	0.96	0.90	0.85	XRAY	2.8	T. thermophilus HB8		Khabibullina, et al. (2019) *AAC*[232]
6OF6	YA,XA	1.4	0.2	-54.9	0.795	-0.7	1.3	-22.0	0.251	2105	881	412	0.95	0.99	0.89	XRAY	3.2	T. thermophilus HB8		Nguyen, et al. (2020) *PNAS*[233]
6OF6	RA,QA	-0.1	0.7	-30.3	0.487	-0.2	0.8	-33.5	0.348	2109	898	414	0.94	0.93	0.92	XRAY	3.2	T. thermophilus HB8		Nguyen, et al. (2020) *PNAS*[233]
6OFX	1,3	2.4	0.5	19.7	1.030	-0.8	1.9	-67.8	0.570	2432	864	426	0.95	1.19	1.03	EM	3.3	E. coli K-12		Svidritskiy, et al. (2019) *Elife*[234]
6OG7	1,3	1.0	1.3	-7.8	1.044	0.6	1.8	-61.3	0.601	2415	910	431	0.96	1.06	1.05	EM	3.3	E. coli K-12		Svidritskiy, et al. (2019) *Elife*[234]
6OGF	1,3	5.4	1.7	109.5	1.391	0.5	2.3	-58.1	0.675	2348	830	414	1.00	1.18	1.12	EM	3.9	E. coli		Svidritskiy, et al. (2019) *Elife*[234]
6OGG	1,3	7.6	3.3	147.4	2.182	2.1	3.1	-48.6	0.788	2078	747	320	1.25	1.31	1.32	EM	4.2	E. coli		Svidritskiy, et al. (2019) *Elife*[234]
6OGI	1,3	8.0	1.1	142.3	1.301	5.6	3.7	-35.9	0.762	2362	823	376	0.96	1.20	1.24	EM	3.4	E. coli		Svidritskiy, et al. (2019) *Elife*[234]
6OJ2	YA,XA	1.3	0.3	-30.2	0.675	-0.8	1.2	-21.9	0.307	2106	881	411	0.99	1.00	0.94	XRAY	3.2	T. thermophilus HB8		Nguyen, et al. (2020) *PNAS*[233]
6OJ2	RA,QA	-0.4	0.8	-24.7	0.525	0.4	0.8	-40.5	0.378	2114	886	412	0.99	0.98	0.98	XRAY	3.2	T. thermophilus HB8		Nguyen, et al. (2020) *PNAS*[233]
6OLE	t,S2	8.9	5.7	38.5	0.842	0.9	1.2	0.2	0.897	1332	597	248	1.25	1.22	1.23	EM	3.1	Homo sapiens		Li, et al. (2019) *NSMB*[235]
6OLF	t,S2	8.7	5.6	39.5	0.768	0.9	1.1	-8.6	1.089	1290	585	232	1.27	1.26	1.29	EM	3.9	Homo sapiens		Li, et al. (2019) *NSMB*[235]
6OLG	A2,B1	-1.8	3.0	24.9	1.523	-0.9	0.9	-90.3	1.353	1322	589	277	1.28	1.26	1.23	EM	3.4	Homo sapiens		Li, et al. (2019) *NSMB*[235]
6OLI	t,S2	8.7	5.6	38.9	0.890	1.0	1.2	-6.1	1.073	1334	594	245	1.24	1.22	1.20	EM	3.5	Homo sapiens		Li, et al. (2019) *NSMB*[235]
6OLZ	A2,B1	-1.7	3.0	24.0	1.660	-1.1	0.7	-53.7	1.566	1309	571	273	1.28	1.27	1.26	EM	3.9	Homo sapiens		Li, et al. (2019) *NSMB*[235]

PDB		BODY				HEAD				PRUNED			RMSD			EXP DETAILS		ORGANISM		REFERENCE
ID	chains	ϕ_{body}	θ_{body}	ψ_{body}	$\lvert\Delta\vec{x}_{\text{body}}\rvert$	ϕ_{head}	θ_{head}	ψ_{head}	$\lvert\Delta\vec{x}_{\text{head}}\rvert$	LSU	body	head	LSU	body	head	method	res.	name	mito	
6OM0	t,S2	8.8	5.7	39.1	0.924	1.0	1.3	2.3	0.845	1332	582	241	1.25	1.21	1.22	EM	3.1	Homo sapiens		Li, et al. (2019) *NSMB*[235]
6OM6	1,2	-1.5	1.6	16.9	0.316	0.8	1.4	-102.6	0.830	2598	1003	408	0.62	0.72	0.80	EM	3.1	E. coli		Aron, et al. (2021) *NComm*[236]
6OM7	t,S2	8.7	5.7	39.4	0.940	1.4	1.2	-6.6	0.647	1298	574	234	1.27	1.23	1.21	EM	3.7	Homo sapiens		Li, et al. (2019) *NSMB*[235]
6OPE	YA,XA	1.2	0.4	-51.9	0.691	-0.7	1.4	-22.8	0.038	2095	871	413	0.96	0.99	0.88	XRAY	3.1	T. thermophilus HB8		Nguyen, et al. (2020) *PNAS*[233]
6OPE	RA,QA	-0.1	0.8	-31.7	0.132	0.0	1.0	-23.9	0.067	2121	882	412	0.97	0.93	0.93	XRAY	3.1	T. thermophilus HB8		Nguyen, et al. (2020) *PNAS*[233]
6ORD	RA,QA	0.1	0.7	-35.8	0.532	-0.1	1.3	-27.0	0.188	2118	883	420	0.96	0.95	0.85	XRAY	3.1	T. thermophilus HB8		Nguyen, et al. (2020) *PNAS*[233]
6ORD	YA,XA	1.2	0.3	-70.4	0.617	-0.3	1.4	-27.0	0.247	2103	874	422	0.97	1.00	0.86	XRAY	3.1	T. thermophilus HB8		Nguyen, et al. (2020) *PNAS*[233]
6ORE	1,2	-1.4	1.3	14.2	0.588	1.3	1.4	-101.6	0.903	2638	1002	434	0.60	0.69	0.73	EM	2.9	E. coli		Fu, et al. (2019) *NComm*[237]
6ORL	1,2	-0.5	0.7	-22.3	0.652	0.0	1.9	-89.5	0.525	2541	942	425	0.77	0.90	0.90	EM	3.5	E. coli		Fu, et al. (2019) *NComm*[237]
6OSI	YA,XA	0.7	0.7	5.8	0.192	17.0	7.5	68.2	1.894	2101	853	260	1.00	1.03	1.37	XRAY	4.1	T. thermophilus HB8		Hoffer, et al. (2020) *Elife*[227]
6OSI	RA,QA	-0.4	1.2	-6.7	0.384	17.3	7.7	66.5	1.811	2125	811	246	0.99	1.12	1.34	XRAY	4.1	T. thermophilus HB8		Hoffer, et al. (2020) *Elife*[227]
6OSK	1,2	-0.2	0.8	-9.6	0.311	-0.1	1.7	-86.3	0.211	2595	983	439	0.71	0.77	0.79	EM	3.6	E. coli		Fu, et al. (2019) *NComm*[237]
6OSQ	1,2	-0.7	1.1	8.3	0.175	0.6	1.4	-77.3	0.113	2630	1003	432	0.62	0.60	0.73	EM	3.5	E. coli		Fu, et al. (2019) *NComm*[237]
6OST	1,2	-1.2	1.2	10.9	0.338	1.1	1.5	-87.6	0.585	2614	991	430	0.75	0.84	0.86	EM	4.2	E. coli		Fu, et al. (2019) *NComm*[237]
6OT3	1,2	-1.0	1.4	11.9	1.091	1.0	1.2	-87.8	1.238	2582	961	424	0.93	0.95	0.84	EM	3.9	E. coli		Fu, et al. (2019) *NComm*[237]
6OTR	RA,QA	-0.3	0.7	-19.1	0.344	1.4	1.7	-52.4	0.467	2101	890	406	0.99	1.00	0.96	XRAY	3.1	T. thermophilus HB8		Pavelich, et al. (2019) *NAR*[238]
6OTR	YA,XA	-0.4	0.8	-150.2	0.485	1.5	1.5	-13.4	0.314	2099	895	404	0.98	0.94	0.88	XRAY	3.1	T. thermophilus HB8		Pavelich, et al. (2019) *NAR*[238]
6OUO	1,2	-1.0	1.4	11.9	1.092	1.0	1.2	-87.8	1.238	2582	961	424	0.93	0.95	0.84	EM	3.7	E. coli		Fu, et al. (2019) *NComm*[237]
6OXA	RA,QA	-0.5	0.9	-9.3	0.488	1.2	1.1	-52.1	0.654	2088	879	406	0.99	1.05	0.93	XRAY	3.2	T. thermophilus HB8		Pavelich, et al. (2019) *NAR*[238]
6OXA	YA,XA	-0.6	0.6	-153.2	0.643	1.2	1.0	2.7	0.587	2096	897	406	0.98	0.99	0.86	XRAY	3.2	T. thermophilus HB8		Pavelich, et al. (2019) *NAR*[238]
6OXI	RA,QA	-0.4	0.8	-13.9	0.436	1.1	1.4	-54.2	0.669	2120	895	403	0.96	1.00	0.96	XRAY	3.5	T. thermophilus HB8		Pavelich, et al. (2019) *NAR*[238]
6OXI	YA,XA	-0.6	0.4	-127.0	0.765	1.2	1.4	-30.6	0.395	2118	897	401	0.96	0.94	0.90	XRAY	3.5	T. thermophilus HB8		Pavelich, et al. (2019) *NAR*[238]
6P5I	5,2	-1.6	3.0	26.9	0.819	9.4	5.2	53.6	0.663	1457	608	255	1.19	1.17	1.14	EM	3.1	Oryctolagus cuniculus		Acosta-Reyes, et al. (2019) *EMBO J*[239]
6P5J	5,2	3.2	2.6	66.5	0.985	8.6	5.2	52.3	0.830	1427	589	245	1.19	1.19	1.20	EM	3.1	Oryctolagus cuniculus		Acosta-Reyes, et al. (2019) *EMBO J*[239]
6P5K	5,2	5.3	3.6	55.7	0.151	9.0	4.8	54.9	0.891	1427	614	259	1.19	1.20	1.16	EM	3.1	Oryctolagus cuniculus		Acosta-Reyes, et al. (2019) *EMBO J*[239]
6P5N	5,2	-2.2	3.0	17.3	2.135	-0.9	2.0	-69.3	1.688	1267	554	290	1.28	1.27	1.22	EM	3.2	Oryctolagus cuniculus		Acosta-Reyes, et al. (2019) *EMBO J*[239]
6Q8Y	BQ,2	-1.9	2.6	19.0	1.278	-0.5	1.4	-43.5	1.334	1400	601	293	1.22	1.21	1.16	EM	3.1	S. cerevisiae		Tesina, et al. (2019) *NSMB*[240]
6Q95	1,2	-0.3	0.5	8.2	1.732	1.6	1.1	-87.8	1.639	1765	806	392	1.26	1.16	1.01	EM	3.7	T. thermophilus HB8		Rae, et al. (2019) *Science*[241]
6Q97	1,2	-1.1	1.1	6.5	2.087	1.3	1.4	-71.5	2.030	2096	896	416	1.34	1.25	1.08	EM	3.9	E. coli		Rae, et al. (2019) *Science*[241]
6Q98	1,2	-1.3	2.1	17.4	0.503	4.3	5.2	-22.5	0.201	2558	981	341	0.95	1.00	1.15	EM	4.3	E. coli		Rae, et al. (2019) *Science*[241]
6Q9A	1,2	-1.7	1.6	12.0	0.426	1.4	1.9	-103.9	1.154	2614	995	423	0.76	0.86	0.87	EM	3.7	E. coli		Rae, et al. (2019) *Science*[241]
6QNQ	14,1G	-0.6	0.5	-39.2	0.560	1.2	1.0	-38.9	0.522	2095	889	394	1.00	1.03	1.02	XRAY	3.5	T. thermophilus HB8		Rozov, et al. (2019) *NComm*[242]
6QNQ	1H,13	1.2	0.3	-86.7	0.827	-0.7	1.3	-19.4	0.446	2090	879	406	1.00	0.98	0.94	XRAY	3.5	T. thermophilus HB8		Rozov, et al. (2019) *NComm*[242]
6QNR	1H,13	1.7	0.3	-20.3	0.900	-0.8	1.1	-18.7	0.165	2091	866	408	0.93	0.95	0.83	XRAY	3.1	T. thermophilus HB8		Rozov, et al. (2019) *NComm*[242]
6QNR	14,1G	0.3	0.7	-22.8	0.399	-0.3	0.5	-35.9	0.276	2113	890	410	0.92	0.89	0.88	XRAY	3.1	T. thermophilus HB8		Rozov, et al. (2019) *NComm*[242]
6QZP	L5,S2	-1.9	3.0	22.2	1.647	-0.1	1.4	-65.9	1.470	1390	584	254	1.23	1.18	1.16	EM	2.9	Homo sapiens		Natchiar, et al. (2017) *Nature*[243]
6R5Q	5,K	-2.2	3.0	21.1	1.284	-1.1	1.0	-84.6	1.145	1425	615	289	1.23	1.21	1.17	EM	3.0	Oryctolagus cuniculus		Shanmuganathan, et al. (2019) *Elife*[244]
6R6G	5,K	-2.1	3.1	23.1	1.310	-1.1	1.2	-86.0	1.174	1392	614	289	1.26	1.24	1.20	EM	3.7	Oryctolagus cuniculus		Shanmuganathan, et al. (2019) *Elife*[244]
6R6P	5,K	9.0	6.0	39.1	0.315	0.9	1.1	25.8	0.507	1392	589	273	1.24	1.22	1.25	EM	3.1	Oryctolagus cuniculus		Shanmuganathan, et al. (2019) *Elife*[244]
6R7Q	5,K	-2.2	2.9	21.9	1.316	-1.0	1.1	-79.8	1.251	1434	606	288	1.20	1.18	1.12	EM	3.9	Oryctolagus cuniculus		Shanmuganathan, et al. (2019) *Elife*[244]
6RM3	L50,S60	3.6	1.7	67.0	5.010	14.3	3.4	43.9	3.766	345	219	160	1.49	1.46	1.37	EM	3.4	Vairimorpha necatrix		Barandun, et al. (2019) *NMb*[245]
6S0X	A,a	7.8	1.9	106.6	2.228	7.2	3.7	-42.4	3.573	1530	435	238	1.26	1.35	1.39	EM	2.4	Staph. aureus		Halfon, et al. (2019) *Sci Rep*[246]
6S13	A,a	6.4	3.0	143.7	4.356	8.8	4.1	-22.3	3.858	1453	180	77	1.33	1.46	1.47	EM	3.6	Staph. aureus		Halfon, et al. (2019) *Sci Rep*[246]
6S47	AA,BA	-1.7	3.3	22.2	1.289	1.1	2.3	-45.6	0.666	1445	611	282	1.24	1.18	1.12	EM	3.3	S. cerevisiae		Kasari, et al. (2019) *NAR*[247]
6SGC	54,A1	-2.0	2.9	23.4	1.384	-0.9	1.1	-83.7	1.416	1409	612	294	1.22	1.18	1.12	EM	2.8	Oryctolagus cuniculus		Chandrasekaran, et al. (2019) *NSMB*[248]
6SKF	BA,Aa	-2.2	2.8	7.8	3.150	-1.6	1.9	-121.3	1.923	911	442	215	1.44	1.36	1.33	EM	3.0	Thermococcus kodakarensis		Sas-Chen, et al. (2020) *Nature*[249]
6SKG	BA,Aa	6.5	2.6	52.4	2.879	3.8	2.1	-59.4	3.000	902	433	225	1.45	1.36	1.33	EM	2.6	Thermococcus kodakarensis		Sas-Chen, et al. (2020) *Nature*[249]
6SNT	1,2	-1.9	2.7	16.7	1.120	-0.6	1.4	-61.7	1.228	1413	615	299	1.23	1.20	1.16	EM	2.8	S. cerevisiae		Matsuo, et al. (2020) *NSMB*[250]
6SPF	A,a	5.6	1.4	71.8	0.938	0.9	3.5	-37.0	0.922	2406	744	91	0.80	1.20	1.26	EM	2.9	Pseudomonas aeruginosa		Halfon, et al. (2019) *PNAS*[251]
6SPG	A,a	-2.2	1.5	20.4	3.277	2.3	1.8	-93.7	2.931	1267	577	352	1.49	1.40	1.33	EM	3.3	Pseudomonas aeruginosa		Halfon, et al. (2019) *PNAS*[251]
6SV4	BQ,2	-1.8	2.7	19.4	1.039	-0.7	1.3	-65.7	1.200	1373	594	286	1.28	1.22	1.23	EM	3.3	S. cerevisiae		Matsuo, et al. (2020) *NSMB*[250]
6SV4	YQ,2b	8.9	6.0	33.7	0.826	0.3	1.1	-28.8	1.061	1342	584	285	1.25	1.23	1.22	EM	3.3	S. cerevisiae		Matsuo, et al. (2020) *NSMB*[250]

PDB		BODY				HEAD				PRUNED			RMSD			EXP DETAILS		ORGANISM		REFERENCE
ID	chains	ϕ_{body}	θ_{body}	ψ_{body}	$\lvert\Delta\vec{x}_{\text{body}}\rvert$	ϕ_{head}	θ_{head}	ψ_{head}	$\lvert\Delta\vec{x}_{\text{head}}\rvert$	LSU	body	head	LSU	body	head	method	res.	name	mito	
6SV4	ZQ,2c	8.8	5.7	27.7	1.035	0.4	1.2	-34.5	0.864	1353	487	260	1.27	1.24	1.23	EM	3.3	S. cerevisiae		Matsuo, et al. (2020) *NSMB*[250]
6SZS	A,a	-1.7	1.9	11.5	0.331	0.6	2.7	-96.0	1.156	2607	997	437	0.63	0.74	0.79	EM	3.1	E. coli K-12		Shimokawa-Chiba, et al. (2019) *NComm*[252]
6T4Q	C1,C2	-1.9	2.7	18.9	1.246	-0.6	1.4	-50.7	1.364	1400	605	295	1.22	1.20	1.15	EM	2.6	S. cerevisiae		Tesina, et al. (2020) *EMBO J*[253]
6T7I	C1,C2	-1.9	2.7	19.5	1.196	-1.0	1.4	-63.7	1.175	1356	597	297	1.26	1.24	1.18	EM	3.2	S. cerevisiae		Tesina, et al. (2020) *EMBO J*[253]
6T7T	C1,C2	-1.9	2.9	16.0	1.254	-0.7	1.2	-67.1	1.237	1409	600	297	1.25	1.22	1.17	EM	3.1	S. cerevisiae S288C		Tesina, et al. (2020) *EMBO J*[253]
6T83	1b,2b	-1.9	3.0	16.9	1.198	-0.7	1.2	-76.3	1.207	1346	593	286	1.28	1.23	1.21	EM	4.0	S. cerevisiae		Tesina, et al. (2020) *EMBO J*[253]
6T83	Aa,a	-1.5	2.7	19.9	1.169	0.3	1.3	-45.3	1.081	1326	588	258	1.27	1.23	1.20	EM	4.0	S. cerevisiae		Tesina, et al. (2020) *EMBO J*[253]
6TB3	BQ,2	-1.7	2.9	18.9	1.200	-0.8	1.4	-71.9	1.129	1441	610	301	1.23	1.20	1.14	EM	2.8	S. cerevisiae S288C		Buschauer, et al. (2020) *Science*[254]
6TBV	23S1,16S1	-1.4	1.3	6.9	1.693	1.1	1.7	-89.9	1.880	2541	963	431	1.11	1.07	0.92	EM	2.7	E. coli K-12		Herrero Del Valle, et al. (2020) *NMb*[255]
6TC3	23S1,16S1	-1.4	1.3	7.0	1.782	1.1	1.7	-90.2	1.958	2521	962	431	1.15	1.10	0.93	EM	2.7	E. coli K-12		Herrero Del Valle, et al. (2020) *NMb*[255]
6TH6	BA,Aa	-2.2	3.0	17.4	3.455	0.3	2.1	-77.5	3.077	902	440	260	1.46	1.36	1.32	EM	2.5	Thermococcus kodakarensis KOD1		Sas-Chen, et al. (2020) *Nature*[249]
6TNU	BQ,2	3.2	3.3	75.3	1.495	-1.4	1.0	-19.9	1.048	1378	594	280	1.24	1.25	1.18	EM	3.1	S. cerevisiae		Buschauer, et al. (2020) *Science*[254]
6UCQ	1A,1a	1.4	0.8	25.7	0.235	2.3	2.1	-31.3	0.153	2120	866	403	0.98	0.96	1.02	XRAY	3.5	T. thermophilus HB8		Zhou, et al. (2020) *NSMB*[256]
6UCQ	2A,2a	0.6	0.7	-4.9	0.607	3.1	2.3	-23.7	0.127	2094	873	391	0.98	0.97	1.10	XRAY	3.5	T. thermophilus HB8		Zhou, et al. (2020) *NSMB*[256]
6UO1	1A,1a	1.4	0.2	-25.4	1.045	-0.8	1.6	-28.1	0.370	2083	863	418	0.96	1.01	0.87	XRAY	3.0	T. thermophilus HB8		Eyler, et al. (2019) *PNAS*[257]
6UO1	2A,2a	-0.1	0.7	-27.5	0.673	0.0	1.3	-39.4	0.382	2100	885	413	0.96	0.93	0.93	XRAY	3.0	T. thermophilus HB8		Eyler, et al. (2019) *PNAS*[257]
6UZ7	5,2	6.4	4.5	27.9	3.126	8.9	3.8	39.7	2.379	822	410	250	1.45	1.40	1.33	EM	3.6	Kluyveromyces lactis		Huang, et al. (2020) *PNAS*[258]
6V39	AN1,sN1	2.6	1.0	68.4	3.486	-0.1	3.0	-106.0	3.772	1461	574	315	1.45	1.33	1.21	EM	3.0	Acinetobacter baumannii AB0057		Morgan, et al. (2020) *mBio*[259]
6V3A	AN1,sN1	4.2	1.0	61.5	3.628	14.3	2.2	91.0	3.800	1503	573	332	1.45	1.32	1.18	EM	2.8	Acinetobacter baumannii AB0057		Morgan, et al. (2020) *mBio*[259]
6V3B	AN1,sN1	4.8	1.9	90.5	3.479	11.0	1.1	-154.3	3.312	1487	563	331	1.46	1.32	1.20	EM	2.9	Acinetobacter baumannii AB0057		Morgan, et al. (2020) *mBio*[259]
6VLZ	A,AA	8.0	2.6	68.0	3.015	4.9	2.0	-159.2	2.421	737	265	132	1.31	1.41	1.38	EM	3.0	Homo sapiens	*	Koripella, et al. (2020) *NComm*[260]
6VMI	A,AA	0.9	1.0	154.2	2.908	3.8	3.0	-160.5	3.143	738	298	132	1.32	1.39	1.38	EM	3.0	Homo sapiens	*	Koripella, et al. (2020) *NComm*[260]
6VU3	a,D	-1.4	1.4	15.7	2.709	1.0	2.0	-92.6	1.034	2612	992	435	0.61	0.74	0.75	EM	3.7	E. coli		Wang, et al. (2020) *Science*[261]
6VWL	2,1	9.7	1.1	23.0	1.184	4.4	4.1	-38.0	0.767	2461	760	367	0.77	1.15	1.14	EM	3.1	E. coli		Bao, et al. (2020) *Elife*[262]
6VWM	2,1	2.7	1.9	17.6	0.775	1.3	2.0	-62.4	1.081	2488	680	401	0.85	1.14	1.10	EM	3.4	E. coli		Bao, et al. (2020) *Elife*[262]
6VWN	2,1	1.1	1.9	-4.5	0.823	-1.2	0.6	-73.6	0.631	2494	701	404	0.83	1.11	1.08	EM	3.4	E. coli		Bao, et al. (2020) *Elife*[262]
6VYQ	a,D	-1.4	1.4	15.7	2.709	1.0	2.0	-92.6	1.034	2612	992	435	0.61	0.74	0.75	EM	3.7	E. coli		Wang, et al. (2020) *Science*[261]
6VYR	a,D	-1.4	1.4	15.7	2.709	1.0	2.0	-92.6	1.034	2612	992	435	0.61	0.74	0.75	EM	3.8	E. coli		Wang, et al. (2020) *Science*[261]
6VYS	a,D	-1.4	1.4	15.7	2.709	1.0	2.0	-92.6	1.034	2612	992	435	0.61	0.74	0.75	EM	3.7	E. coli		Wang, et al. (2020) *Science*[261]
6VYT	a,D	-1.4	1.4	15.7	2.709	1.0	2.0	-92.6	1.034	2612	992	435	0.61	0.74	0.75	EM	14.0	E. coli		Wang, et al. (2020) *Science*[261]
6VYU	a,D	-1.4	1.4	15.7	2.710	1.0	2.0	-92.6	1.034	2613	992	435	0.61	0.74	0.75	EM	7.0	E. coli		Wang, et al. (2020) *Science*[261]
6VYW	a,D	-1.4	1.4	15.7	2.710	1.0	2.0	-92.6	1.034	2613	992	435	0.61	0.74	0.75	EM	7.0	E. coli		Wang, et al. (2020) *Science*[261]
6VYX	a,D	-1.4	1.4	15.6	2.707	1.0	2.0	-92.5	1.029	2613	993	434	0.61	0.74	0.75	EM	9.9	E. coli		Wang, et al. (2020) *Science*[261]
6VYY	a,D	-1.4	1.4	15.6	2.707	1.0	2.0	-92.5	1.028	2613	993	435	0.61	0.74	0.75	EM	9.9	E. coli		Wang, et al. (2020) *Science*[261]
6VYZ	a,D	-1.4	1.4	15.6	2.707	1.0	2.0	-92.5	1.028	2613	993	435	0.61	0.74	0.75	EM	9.9	E. coli		Wang, et al. (2020) *Science*[261]
6VZ2	a,D	-1.4	1.4	15.6	2.708	1.0	2.0	-92.5	1.028	2613	994	435	0.61	0.74	0.75	EM	10.0	E. coli		Wang, et al. (2020) *Science*[261]
6VZ3	a,D	-1.4	1.4	15.6	2.708	1.0	2.0	-92.5	1.028	2613	994	435	0.61	0.74	0.75	EM	8.9	E. coli		Wang, et al. (2020) *Science*[261]
6VZ5	a,D	-1.4	1.4	15.6	2.708	1.0	2.0	-92.5	1.028	2613	994	435	0.61	0.74	0.75	EM	8.9	E. coli		Wang, et al. (2020) *Science*[261]
6VZ7	a,D	-1.4	1.4	15.7	2.710	1.0	2.0	-92.6	1.034	2613	992	435	0.61	0.74	0.75	EM	7.0	E. coli		Wang, et al. (2020) *Science*[261]
6VZJ	a,D	-1.4	1.4	15.7	2.709	1.0	2.0	-92.6	1.034	2612	992	435	0.61	0.74	0.75	EM	4.1	E. coli		Wang, et al. (2020) *Science*[261]
6W6P	A,a	0.2	1.7	47.8	4.502	16.4	0.8	13.1	2.636	1094	448	358	1.47	1.38	1.28	EM	2.9	Enterococcus faecalis OG1RF		Murphy, et al. (2020) *Sci Rep*[229]
6WD0	1,3	-1.2	1.4	9.3	0.252	0.7	1.6	-98.0	0.790	2618	992	445	0.72	0.84	0.88	EM	3.0	E. coli		Loveland, et al. (2020) *Nature*[263]
6WD1	1,3	8.7	1.4	23.2	0.484	4.8	2.9	-40.9	0.185	2591	953	440	0.80	0.98	0.97	EM	3.3	E. coli		Loveland, et al. (2020) *Nature*[263]
6WD2	1,3	-1.5	0.9	6.5	0.422	0.9	1.7	-97.0	0.773	2618	983	441	0.81	0.94	0.90	EM	3.6	E. coli		Loveland, et al. (2020) *Nature*[263]
6WD3	1,3	-1.4	0.9	6.5	0.305	0.7	1.6	-94.3	0.618	2630	982	439	0.80	0.89	0.89	EM	3.6	E. coli		Loveland, et al. (2020) *Nature*[263]
6WD4	1,3	-1.5	0.9	6.7	0.449	0.8	1.8	-98.5	0.765	2619	977	445	0.82	0.94	0.94	EM	3.7	E. coli		Loveland, et al. (2020) *Nature*[263]
6WD5	1,3	-1.4	0.9	6.7	0.408	0.7	1.8	-96.4	0.700	2621	977	441	0.81	0.93	0.91	EM	3.6	E. coli		Loveland, et al. (2020) *Nature*[263]
6WD6	1,3	-1.5	0.9	8.8	0.397	0.7	1.8	-96.3	0.742	2617	982	441	0.83	0.93	0.92	EM	3.7	E. coli K-12		Loveland, et al. (2020) *Nature*[263]
6WD7	1,3	-1.5	1.2	8.2	0.414	0.8	1.7	-99.5	0.840	2613	977	447	0.88	0.98	0.95	EM	3.9	E. coli		Loveland, et al. (2020) *Nature*[263]
6WD8	1,3	-0.3	0.8	10.5	0.227	-0.1	1.4	-81.4	0.262	2622	984	440	0.83	0.86	0.93	EM	3.7	E. coli		Loveland, et al. (2020) *Nature*[263]
6WD9	1,3	0.6	0.7	8.3	0.259	-0.7	1.3	-67.7	0.132	2614	985	442	0.83	0.96	0.93	EM	3.7	E. coli		Loveland, et al. (2020) *Nature*[263]
6WDA	1,3	0.0	0.7	6.1	0.188	-0.4	1.4	-78.6	0.113	2618	982	441	0.84	0.91	0.97	EM	3.8	E. coli		Loveland, et al. (2020) *Nature*[263]

PDB		BODY				HEAD				PRUNED			RMSD			EXP DETAILS		ORGANISM		REFERENCE
ID	chains	ϕ_{body}	θ_{body}	ψ_{body}	$\lvert\Delta\vec{x}_{\text{body}}\rvert$	ϕ_{head}	θ_{head}	ψ_{head}	$\lvert\Delta\vec{x}_{\text{head}}\rvert$	LSU	body	head	LSU	body	head	method	res.	name	mito	
6WDB	1,3	2.3	1.2	35.7	0.568	-0.5	1.2	-46.0	0.585	2593	946	446	0.82	1.08	0.89	EM	4.0	E. coli		Loveland, et al. (2020) *Nature*[263]
6WDC	1,3	2.4	1.2	34.0	0.534	-0.4	1.3	-37.4	0.582	2587	921	443	0.90	1.11	0.96	EM	4.2	E. coli		Loveland, et al. (2020) *Nature*[263]
6WDD	1,3	2.7	1.0	36.4	0.612	-0.5	1.4	-27.2	0.510	2618	953	445	0.77	1.10	0.88	EM	3.2	E. coli		Loveland, et al. (2020) *Nature*[263]
6WDE	1,3	1.6	0.8	6.9	0.437	-0.8	1.3	-40.9	0.491	2619	964	442	0.74	1.02	0.84	EM	3.0	E. coli		Loveland, et al. (2020) *Nature*[263]
6WDF	1,3	9.9	1.1	21.1	0.531	2.6	2.5	-28.9	0.656	2591	943	443	0.82	1.08	0.99	EM	3.3	E. coli K-12		Loveland, et al. (2020) *Nature*[263]
6WDG	1,3	10.3	1.2	16.8	0.649	2.8	2.8	-31.7	0.770	2588	935	444	0.82	1.08	0.99	EM	3.3	E. coli K-12		Loveland, et al. (2020) *Nature*[263]
6WDH	1,3	-0.3	1.1	5.5	0.372	0.5	1.6	-76.6	0.219	2597	973	440	0.94	0.99	0.99	EM	4.3	E. coli		Loveland, et al. (2020) *Nature*[263]
6WDI	1,3	3.2	0.8	52.7	0.067	-1.1	1.6	-35.5	0.450	2602	899	443	0.93	1.14	0.99	EM	4.0	E. coli		Loveland, et al. (2020) *Nature*[263]
6WDJ	1,3	-1.1	1.2	7.3	0.571	0.9	1.5	-90.7	0.666	2609	974	443	0.89	0.93	0.95	EM	3.7	E. coli K-12		Loveland, et al. (2020) *Nature*[263]
6WDK	1,3	3.1	0.8	46.0	0.117	-1.1	1.3	-36.7	0.473	2601	925	446	0.88	1.13	0.95	EM	3.6	E. coli		Loveland, et al. (2020) *Nature*[263]
6WDL	1,3	-0.2	1.0	8.2	0.273	0.2	1.4	-75.3	0.283	2601	978	444	0.88	0.93	0.95	EM	3.7	E. coli K-12		Loveland, et al. (2020) *Nature*[263]
6WDM	1,3	1.3	0.7	8.2	0.059	-0.7	1.3	-46.7	0.395	2608	956	443	0.86	1.01	0.93	EM	3.6	E. coli		Loveland, et al. (2020) *Nature*[263]
6WNV	4,3	-1.6	2.0	27.4	0.775	4.2	1.3	-65.6	0.459	2457	966	417	0.83	0.84	1.09	EM	3.5	E. coli		Huang, et al. (2020) *NComm*[264]
6WNW	4,3	0.1	0.9	17.2	0.160	0.0	1.2	-67.5	0.335	2618	900	445	0.73	0.85	0.82	EM	3.2	E. coli		Huang, et al. (2020) *NComm*[264]
6WOO	5,2	3.6	4.2	68.9	2.855	-0.8	1.4	-64.9	0.957	1449	592	294	1.24	1.18	1.12	EM	2.9	S. cerevisiae		Wang, et al. *To be published*
6X6T	a,D	-1.4	1.4	15.7	2.709	1.0	2.0	-92.6	1.034	2612	992	435	0.61	0.74	0.75	EM	3.2	E. coli		Wang, et al. (2020) *Science*[261]
6X7F	a,D	-1.4	1.4	15.7	2.710	1.0	2.0	-92.6	1.034	2613	992	435	0.61	0.74	0.75	EM	3.5	E. coli		Wang, et al. (2020) *Science*[261]
6X7K	a,D	-1.4	1.4	15.7	2.710	1.0	2.0	-92.6	1.034	2613	992	435	0.61	0.74	0.75	EM	3.1	E. coli		Wang, et al. (2020) *Science*[261]
6X9Q	a,D	-1.4	1.4	15.7	2.710	1.0	2.0	-92.6	1.034	2613	992	435	0.61	0.74	0.75	EM	4.8	E. coli		Wang, et al. (2020) *Science*[261]
6XA1	L5,S2	-2.1	2.9	21.1	1.690	-1.3	1.0	-70.8	1.661	1362	599	222	1.24	1.20	1.19	EM	2.8	Homo sapiens		Li, et al. (2020) *NComm*[265]
6XDQ	a,D	-1.4	1.4	15.7	2.709	1.0	2.0	-92.6	1.034	2612	992	435	0.61	0.74	0.75	EM	3.7	E. coli		Wang, et al. (2020) *Science*[261]
6XDR	a,D	-1.4	1.4	15.7	2.710	1.0	2.0	-92.6	1.034	2613	992	435	0.61	0.74	0.75	EM	4.7	E. coli		Wang, et al. (2020) *Science*[261]
6XGF	a,D	-1.4	1.4	15.7	2.709	1.0	2.0	-92.6	1.034	2612	992	435	0.61	0.74	0.75	EM	5.0	E. coli		Wang, et al. (2020) *Science*[261]
6XHV	1A,1a	1.6	0.4	-29.4	1.336	-0.8	1.3	-25.9	0.759	2043	863	413	1.01	1.01	0.83	XRAY	2.4	T. thermophilus HB8		Svetlov, et al. (2021) *NCB*[266]
6XHV	2A,2a	0.2	0.7	-31.4	0.813	-0.1	0.9	-27.0	0.804	2059	888	411	1.00	0.95	0.87	XRAY	2.4	T. thermophilus HB8		Svetlov, et al. (2021) *NCB*[266]
6XHW	1A,1a	1.4	0.3	-41.3	0.569	-0.6	1.4	-24.6	0.319	2071	853	414	1.00	1.00	0.85	XRAY	2.5	T. thermophilus HB8		Svetlov, et al. (2021) *NCB*[266]
6XHW	2A,2a	0.2	0.5	-39.5	0.464	-0.3	1.1	-33.9	0.265	2100	879	417	0.98	0.93	0.87	XRAY	2.5	T. thermophilus HB8		Svetlov, et al. (2021) *NCB*[266]
6XHX	1A,1a	1.7	0.6	5.8	0.483	-1.3	1.4	-58.9	0.817	2100	857	415	0.95	0.99	0.87	XRAY	2.5	T. thermophilus HB8		Svetlov, et al. (2021) *NCB*[266]
6XHX	2A,2a	-1.0	0.9	-6.6	0.403	1.3	1.3	-75.2	0.302	2145	888	418	0.95	1.00	0.94	XRAY	2.5	T. thermophilus HB8		Svetlov, et al. (2021) *NCB*[266]
6XHY	1A,1a	1.4	0.3	-33.2	0.743	-0.7	1.4	-25.9	0.101	2087	864	417	0.95	0.95	0.82	XRAY	2.6	T. thermophilus HB8		Svetlov, et al. (2021) *NCB*[266]
6XHY	2A,2a	0.1	0.7	-36.1	0.194	-0.1	1.0	-25.2	0.110	2106	894	414	0.94	0.91	0.84	XRAY	2.6	T. thermophilus HB8		Svetlov, et al. (2021) *NCB*[266]
6XII	a,D	-1.4	1.4	15.7	2.709	1.0	2.0	-92.6	1.034	2612	992	435	0.61	0.74	0.75	EM	7.0	E. coli		Wang, et al. (2020) *Science*[261]
6XIJ	a,D	-1.4	1.4	15.7	2.709	1.0	2.0	-92.6	1.034	2612	992	435	0.61	0.74	0.75	EM	8.0	E. coli		Wang, et al. (2020) *Science*[261]
6XIQ	1,2	9.1	5.8	30.2	2.722	1.0	1.0	-30.6	1.864	536	431	208	1.44	1.30	1.25	EM	4.2	S. cerevisiae		Zhou, et al. (2020) *PNAS*[267]
6XIR	1,2	7.9	6.4	13.0	3.751	3.7	2.0	-19.0	1.595	1143	435	121	1.30	1.32	1.37	EM	3.2	S. cerevisiae		Zhou, et al. (2020) *PNAS*[267]
6XQD	1A,1a	1.4	0.3	-1.3	1.090	-0.6	1.4	-30.8	0.568	2054	869	412	1.00	0.95	0.86	XRAY	2.8	T. thermophilus HB8		Batool, et al. (2020) *PNAS*[268]
6XQD	2A,2a	-0.6	0.9	-21.8	1.118	1.2	1.3	-50.5	0.590	2069	881	409	1.01	0.94	0.89	XRAY	2.8	T. thermophilus HB8		Batool, et al. (2020) *PNAS*[268]
6XQE	1A,1a	1.4	0.3	-37.7	0.678	-0.7	1.6	-36.4	0.170	2085	863	414	0.98	0.95	0.88	XRAY	3.0	T. thermophilus HB8		Batool, et al. (2020) *PNAS*[268]
6XQE	2A,2a	-0.7	0.7	-15.3	0.557	1.3	1.4	-62.4	0.392	2117	888	415	0.96	0.97	0.96	XRAY	3.0	T. thermophilus HB8		Batool, et al. (2020) *PNAS*[268]
6XU6	A5,B2	-1.8	2.5	24.5	1.521	0.7	1.8	-84.3	0.904	1371	578	128	1.22	1.20	1.16	EM	3.5	Drosophila melanogaster		Hopes, et al. (2021) *NAR*[269]
6XU7	A5,B2	-1.5	2.8	27.5	1.431	0.2	1.5	-84.7	1.301	1229	526	132	1.34	1.31	1.25	EM	4.9	Drosophila melanogaster		Hopes, et al. (2021) *NAR*[269]
6XU8	A5,B2	-1.8	2.8	25.8	1.550	0.9	1.8	-91.0	0.921	1407	564	132	1.23	1.18	1.21	EM	3.0	Drosophila melanogaster		Hopes, et al. (2021) *NAR*[269]
6XYW	1,2	0.3	1.9	48.4	1.736	0.4	1.1	-138.8	0.578	1854	730	41	1.09	1.22	0.78	EM	3.9	Arabidopsis thaliana	*	Waltz, et al. (2020) *Nat Plants*[270]
6XZA	A2,A1	-0.6	0.5	-0.1	0.692	-0.1	1.2	-66.9	0.544	2634	986	436	0.63	0.76	0.68	EM	2.7	E. coli K-12		Pichkur, et al. (2020) *RNA*[271]
6XZB	A2,A1	0.2	0.7	12.4	0.389	-0.1	1.2	-66.9	0.544	2634	986	436	0.63	0.76	0.68	EM	2.5	E. coli K-12		Pichkur, et al. (2020) *RNA*[271]
6Y0G	L5,S2	2.8	3.4	77.5	1.644	-1.6	1.0	-25.8	1.278	1364	599	282	1.23	1.20	1.15	EM	3.2	Homo sapiens		Bhaskar, et al. (2020) *Cell Rep*[272]
6Y2L	L5,S2	-2.0	3.0	19.5	1.666	-0.8	1.2	-78.6	1.337	1381	589	290	1.24	1.19	1.14	EM	3.0	Homo sapiens		Bhaskar, et al. (2020) *Cell Rep*[272]
6Y57	L5,S2	8.5	5.6	38.9	0.879	0.9	1.4	-19.5	1.187	1335	599	283	1.24	1.23	1.18	EM	3.5	Homo sapiens		Bhaskar, et al. (2020) *Cell Rep*[272]
6Y69	A,a	-1.9	1.2	13.6	0.799	1.2	2.0	-100.2	1.215	2602	992	445	0.67	0.75	0.81	EM	2.9	E. coli K-12		Osterman, et al. (2020) *NCB*[273]
6YDP	BA,AA	1.1	1.5	151.6	1.655	1.0	3.1	-122.1	2.143	1017	353	197	1.00	1.09	1.15	EM	3.0	Sus scrofa	*	Kummer, et al. (2020) *EMBO J*[274]
6YDW	BA,AA	1.6	2.0	144.5	1.613	15.9	3.6	79.2	1.894	1011	350	185	1.11	1.17	1.25	EM	4.2	Sus scrofa	*	Kummer, et al. (2020) *EMBO J*[274]
6YEF	A,a	-1.0	1.6	13.0	3.078	0.2	2.2	-109.2	3.042	1550	637	384	1.39	1.40	1.17	EM	3.2	Staph. aureus subsp. aureus NCTC 8325		Golubev, et al. (2020) *FEBS Lett*[275]

PDB		BODY				HEAD				PRUNED			RMSD			EXP DETAILS		ORGANISM		REFERENCE
ID	chains	ϕ_{body}	θ_{body}	ψ_{body}	$\lvert\Delta\vec{x}_{\text{body}}\rvert$	ϕ_{head}	θ_{head}	ψ_{head}	$\lvert\Delta\vec{x}_{\text{head}}\rvert$	LSU	body	head	LSU	body	head	method	res.	name	mito	
6YSR	A,a	-1.5	1.7	10.7	0.222	1.0	2.0	-104.3	0.981	2622	998	438	0.70	0.77	0.78	EM	3.1	E. coli		Chan, et al. (2020) *NComm*[276]
6YSS	A,a	-1.4	1.7	9.4	0.202	0.9	2.0	-101.3	0.877	2627	1002	434	0.69	0.68	0.73	EM	2.6	E. coli		Chan, et al. (2020) *NComm*[276]
6YST	A,a	2.0	1.6	16.8	0.821	18.2	4.1	22.9	1.391	2605	917	350	0.72	1.03	0.99	EM	3.2	E. coli		Chan, et al. (2020) *NComm*[276]
6YSU	A,a	-0.8	1.5	15.3	0.388	1.4	1.5	-102.4	0.380	2597	983	420	0.78	0.78	0.89	EM	3.7	E. coli		Chan, et al. (2020) *NComm*[276]
6YWE	A,aa	5.4	1.5	106.1	2.036	4.9	4.8	-65.3	0.727	1336	470	218	1.16	1.23	1.14	EM	3.0	Neurospora crassa	*	Itoh, et al. (2020) *NComm*[277]
6YWX	A,aa	-1.9	1.0	161.8	2.581	2.7	3.5	-78.1	0.486	1349	532	221	1.15	1.23	1.18	EM	3.1	Neurospora crassa OR74A	*	Itoh, et al. (2020) *NComm*[277]
6YWY	A,aa	-1.4	1.1	176.7	2.370	2.0	3.4	-72.0	0.405	1364	539	224	1.11	1.21	1.16	EM	3.0	Neurospora crassa	*	Itoh, et al. (2020) *NComm*[277]
6Z1P	Ab,Bb	7.1	2.8	144.9	3.550	6.7	10.4	-52.0	0.216	1116	235	225	1.28	1.35	1.29	EM	3.7	Tetrahymena thermophila SB210	*	Tobiasson, et al. (2020) *Elife*[278]
6Z6J	C1,C2	-1.8	2.9	19.6	1.187	-0.2	1.8	-49.3	1.344	1427	603	292	1.24	1.21	1.18	EM	3.4	S. cerevisiae S288C		Wells, et al. (2020) *PLoS Biol*[279]
6Z6K	C1,C2	-1.7	2.9	20.1	1.037	0.1	1.5	-45.3	1.277	1433	606	293	1.24	1.20	1.19	EM	3.4	S. cerevisiae S288C		Wells, et al. (2020) *PLoS Biol*[279]
6Z6L	L5,S2	-2.1	2.7	19.8	1.741	-0.6	1.7	-67.1	1.558	1345	575	285	1.25	1.20	1.13	EM	3.0	Homo sapiens		Wells, et al. (2020) *PLoS Biol*[279]
6Z6M	L5,S2	6.2	4.8	40.7	1.236	12.5	7.0	75.1	1.132	1352	560	260	1.26	1.21	1.15	EM	3.1	Homo sapiens		Wells, et al. (2020) *PLoS Biol*[279]
6Z6N	L5,S2	-1.9	2.7	20.9	1.746	-1.1	1.9	-64.7	1.654	1357	572	280	1.25	1.23	1.20	EM	2.9	Homo sapiens		Wells, et al. (2020) *PLoS Biol*[279]
6ZM5	A,AA	0.9	0.2	-164.2	2.975	1.6	2.5	-158.3	2.287	975	351	198	1.06	1.18	1.12	EM	2.9	Homo sapiens	*	Itoh, et al. (2021) *Science*[280]
6ZM6	A,AA	0.9	0.3	-152.7	3.001	1.5	2.5	-155.7	2.165	981	354	198	1.06	1.18	1.11	EM	2.6	Homo sapiens	*	Itoh, et al. (2021) *Science*[280]
6ZM7	L5,S2	-2.1	2.9	18.9	1.970	0.7	3.4	-57.1	1.896	1283	569	283	1.26	1.25	1.20	EM	2.7	Homo sapiens		Thoms, et al. (2020) *Science*[281]
6ZME	L5,S2	-2.0	2.6	26.3	1.761	0.5	3.9	-57.0	1.895	1299	573	280	1.26	1.26	1.18	EM	3.0	Homo sapiens		Thoms, et al. (2020) *Science*[281]
6ZMI	L5,S2	-2.3	3.0	20.5	2.014	0.7	2.5	-69.1	1.700	1300	569	288	1.27	1.23	1.19	EM	2.6	Homo sapiens		Thoms, et al. (2020) *Science*[281]
6ZMO	L5,S2	-2.3	3.1	19.8	2.114	0.6	2.4	-69.1	1.644	1289	561	289	1.26	1.24	1.19	EM	3.1	Homo sapiens		Thoms, et al. (2020) *Science*[281]
6ZS9	XA,AA	7.4	2.1	95.1	1.975	2.5	2.7	-154.7	1.685	993	331	173	1.09	1.26	1.23	EM	4.0	Homo sapiens	*	Aibara, et al. (2020) *Elife*[282]
6ZSA	XA,AA	1.0	0.5	-124.4	2.469	1.7	2.4	-158.2	1.739	996	337	177	1.10	1.21	1.19	EM	4.0	Homo sapiens	*	Aibara, et al. (2020) *Elife*[282]
6ZSB	XA,AA	0.5	0.3	-107.3	2.344	2.0	2.8	-156.8	1.843	987	340	175	1.14	1.24	1.19	EM	4.5	Homo sapiens	*	Aibara, et al. (2020) *Elife*[282]
6ZSC	XA,AA	0.3	0.1	-88.9	2.373	2.1	2.8	-159.9	1.594	1000	344	180	1.07	1.22	1.24	EM	3.5	Homo sapiens	*	Aibara, et al. (2020) *Elife*[282]
6ZSD	XA,AA	0.4	0.4	-123.4	2.456	1.6	2.6	-149.2	1.766	993	344	183	1.09	1.20	1.19	EM	3.7	Homo sapiens	*	Aibara, et al. (2020) *Elife*[282]
6ZSE	XA,AA	6.4	2.1	100.2	2.062	2.4	3.1	-156.2	1.888	969	332	172	1.13	1.24	1.24	EM	5.0	Homo sapiens	*	Aibara, et al. (2020) *Elife*[282]
6ZSG	XA,AA	0.9	0.4	-142.9	2.454	1.7	2.7	-158.0	1.927	979	337	179	1.09	1.21	1.17	EM	4.0	Homo sapiens	*	Aibara, et al. (2020) *Elife*[282]
6ZTJ	BA,AA	1.5	0.5	3.7	0.279	-1.1	1.5	-35.1	0.314	2617	979	442	0.65	0.92	0.74	EM	3.4	E. coli		Webster, et al. (2020) *Science*[283]
6ZTL	BA,AA	1.8	0.6	12.1	0.428	-1.3	1.4	-39.0	0.263	2606	962	436	0.70	1.01	0.80	EM	3.5	E. coli		Webster, et al. (2020) *Science*[283]
6ZTM	BA,AA	2.5	0.7	24.1	0.525	-1.4	1.2	-45.4	0.311	2606	960	443	0.67	1.00	0.75	EM	3.3	E. coli		Webster, et al. (2020) *Science*[283]
6ZTN	BA,AA	1.6	0.7	13.9	1.557	-1.0	1.2	-41.1	1.792	2317	845	432	1.31	1.23	1.07	EM	3.9	E. coli		Webster, et al. (2020) *Science*[283]
6ZTO	BA,AA	1.2	0.5	0.5	0.153	-1.1	1.5	-39.4	0.222	2621	992	438	0.62	0.86	0.71	EM	3.0	E. coli		Webster, et al. (2020) *Science*[283]
6ZTP	BA,AA	1.2	0.5	0.5	0.153	-1.1	1.5	-39.4	0.222	2621	992	438	0.62	0.86	0.71	EM	3.0	E. coli		Webster, et al. (2020) *Science*[283]
6ZU1	BA,AA	1.2	0.5	0.5	0.153	-1.1	1.5	-39.4	0.222	2621	992	438	0.62	0.86	0.71	EM	3.0	E. coli		Webster, et al. (2020) *Science*[283]
6ZU5	L50,S60	-0.8	3.4	30.6	0.677	1.7	1.1	40.2	0.744	1437	583	281	1.18	1.16	1.17	EM	2.9	Paranosema locustae		Ehrenbolger, et al. (2020) *PLoS Biol*[284]
6ZVK	e2,K3	-2.0	3.3	21.4	1.923	-0.7	2.3	-79.2	1.499	1266	585	272	1.29	1.31	1.25	EM	3.5	Oryctolagus cuniculus		Abaeva, et al. (2020) *Cell Rep*[285]
7A01	e2,K3	6.5	4.8	37.3	1.227	2.8	2.0	-35.0	1.949	1246	453	206	1.28	1.36	1.33	EM	3.6	Oryctolagus cuniculus		Abaeva, et al. (2020) *Cell Rep*[285]
7A5F	A3,A6	0.1	0.5	-124.4	2.018	1.9	2.6	-155.6	1.511	1018	396	197	1.06	1.15	1.09	EM	4.4	Homo sapiens	*	Desai, et al. (2020) *Science*[286]
7A5G	A3,A6	0.0	0.3	-143.2	1.949	1.7	2.9	-156.4	1.631	1009	392	194	1.05	1.13	1.13	EM	4.3	Homo sapiens	*	Desai, et al. (2020) *Science*[286]
7A5I	A3,A6	0.6	0.2	-148.6	2.059	1.3	2.5	-162.4	1.406	1012	381	196	1.00	1.12	1.11	EM	3.7	Homo sapiens	*	Desai, et al. (2020) *Science*[286]
7A5K	A3,A6	2.1	1.7	148.3	1.488	1.0	2.2	-158.4	1.633	999	392	174	1.01	1.15	1.30	EM	3.7	Homo sapiens	*	Desai, et al. (2020) *Science*[286]
7ABZ	1,2	0.6	1.4	1.5	1.103	-0.2	1.1	-122.8	1.009	2449	881	393	0.94	1.09	1.12	EM	3.2	E. coli K-12		Guyomar, et al. (2021) *NComm*[287]
7AC7	1,2	-1.1	0.9	7.3	1.071	1.0	1.1	-97.1	1.133	2572	977	430	0.94	1.02	0.94	EM	3.1	E. coli K-12		Guyomar, et al. (2021) *NComm*[287]
7ACJ	1,2	-1.6	2.2	20.4	1.207	4.8	4.4	-12.6	0.558	2503	977	409	1.00	0.97	1.10	EM	3.2	E. coli K-12		Guyomar, et al. (2021) *NComm*[287]
7ACR	1,2	-0.9	2.1	23.6	1.079	13.1	12.6	-22.5	1.249	2492	941	367	1.05	1.11	1.12	EM	3.4	E. coli K-12		Guyomar, et al. (2021) *NComm*[287]
7ASO	Y,X	3.0	1.9	53.6	2.881	-1.7	1.8	109.8	3.752	832	163	77	1.48	1.46	1.40	EM	3.1	Staph. aureus		Cimicata, et al. *To be published*
7ASP	Y,X	5.6	2.5	132.9	4.956	-4.7	0.7	-65.4	6.116	925	101	44	1.45	1.54	1.57	EM	2.9	Staph. aureus		Cimicata, et al. *To be published*
7AZO	23SA,16SA	1.7	0.2	-23.5	0.900	-0.8	1.2	-23.6	0.194	2115	875	410	0.93	1.00	0.84	XRAY	3.3	T. thermophilus HB8		Zhang, et al. *To be published*
7AZO	23SB,16SB	0.2	0.8	-28.7	0.269	-0.3	0.6	-31.2	0.240	2134	901	414	0.94	0.91	0.90	XRAY	3.3	T. thermophilus HB8		Zhang, et al. *To be published*
7AZS	23SA,16SA	1.7	0.3	-0.6	0.644	-0.9	1.1	-31.0	0.058	2121	868	413	0.96	1.00	0.87	XRAY	3.1	T. thermophilus HB8		Zhang, et al. *To be published*
7AZS	23SB,16SB	0.2	0.8	-23.1	0.418	-0.4	0.6	-46.6	0.157	2128	894	413	0.94	0.90	0.90	XRAY	3.1	T. thermophilus HB8		Zhang, et al. *To be published*
7B5K	A,a	0.7	0.8	6.1	1.309	-0.2	1.1	-30.6	1.543	2488	903	433	1.21	1.14	0.91	EM	2.9	E. coli K-12		Albers, et al. (2021) *NComm*
7B7D	LA,2	-1.3	2.9	26.8	1.209	-0.1	1.1	-60.2	1.452	1411	614	291	1.24	1.21	1.16	EM	3.3	S. cerevisiae S288C		Ranjan, et al. *To be published*

Table 20 of 24

PDB		BODY				HEAD				PRUNED			RMSD			EXP DETAILS		ORGANISM		REFERENCE
ID	chains	ϕ_{body}	θ_{body}	ψ_{body}	$\lvert\Delta\vec{x}_{\text{body}}\rvert$	ϕ_{head}	θ_{head}	ψ_{head}	$\lvert\Delta\vec{x}_{\text{head}}\rvert$	LSU	body	head	LSU	body	head	method	res.	name	mito	
7CPJ	A,a	-1.5	1.3	11.3	0.205	0.5	1.7	-102.5	0.998	2622	985	443	0.61	0.78	0.76	EM	3.3	E. coli		Fujita, et al. (2022) *RNA*[288]
7CPU	L5,S2	8.8	5.6	38.8	0.059	0.9	1.4	-16.8	1.128	1433	615	290	1.18	1.18	1.13	EM	2.8	Mus musculus		Huo, et al. *To be published*
7CPV	L5,S2	8.7	5.7	39.2	0.129	1.0	1.2	-20.8	1.077	1435	615	292	1.18	1.20	1.16	EM	3.0	Mus musculus		Huo, et al. *To be published*
7D6Z	A,f	-1.0	1.3	8.7	1.902	0.7	1.8	-88.8	1.942	2493	952	433	1.15	1.11	0.95	EM	3.4	E. coli		Akbar, et al. (2021) *Structure*[289]
7D80	A,B	3.7	0.8	60.2	1.975	-1.9	1.7	-28.3	1.200	2410	657	387	1.23	1.27	1.13	EM	4.1	E. coli		Akbar, et al. (2021) *Structure*[289]
7JIL	1,2	-1.8	1.8	23.0	2.264	0.5	2.4	-124.1	2.313	1957	801	358	1.25	1.27	1.20	EM	2.8	Flavobacterium johnsoniae		Jha, et al. (2021) *NAR*[290]
7JQL	1A,1a	1.5	0.4	-9.5	0.573	-0.8	1.4	-35.7	0.096	2096	863	417	0.96	0.95	0.86	XRAY	3.0	T. thermophilus HB8		Mardirossian, et al. (2020) *J Med Chem*[291]
7JQL	2A,2a	-0.8	0.8	-16.8	0.277	1.3	1.4	-54.4	0.269	2113	888	412	0.95	0.98	0.89	XRAY	3.0	T. thermophilus HB8		Mardirossian, et al. (2020) *J Med Chem*[291]
7JQM	1A,1a	1.6	0.3	-26.1	0.757	-0.9	1.5	-33.4	0.179	2083	858	419	0.96	0.99	0.86	XRAY	3.0	T. thermophilus HB8		Mardirossian, et al. (2020) *J Med Chem*[291]
7JQM	2A,2a	-0.8	0.6	-18.3	0.240	1.3	1.4	-56.5	0.321	2112	882	409	0.95	0.96	0.91	XRAY	3.0	T. thermophilus HB8		Mardirossian, et al. (2020) *J Med Chem*[291]
7JSS	1,3	2.6	0.8	32.8	0.743	-1.2	1.2	-83.4	0.547	2485	852	410	0.95	1.18	1.07	EM	3.7	E. coli K-12		Carbone, et al. (2020) *NComm*[292]
7JSW	1,3	2.7	1.0	31.1	0.665	-0.9	1.3	-93.7	0.587	2444	819	402	0.94	1.20	1.11	EM	3.8	E. coli K-12		Carbone, et al. (2020) *NComm*[292]
7JSZ	1,3	3.4	0.9	53.2	0.810	-1.2	1.3	-75.6	0.631	2455	819	392	0.94	1.24	1.07	EM	3.7	E. coli K-12		Carbone, et al. (2020) *NComm*[292]
7JT1	1,3	0.5	1.2	4.2	0.347	-0.4	1.5	-88.7	0.239	2612	941	437	0.77	0.87	0.92	EM	3.3	E. coli K-12		Carbone, et al. (2020) *NComm*[292]
7JT2	1,3	0.3	1.2	6.7	0.314	-0.2	1.7	-98.3	0.308	2602	942	441	0.81	0.91	1.01	EM	3.5	E. coli K-12		Carbone, et al. (2020) *NComm*[292]
7JT3	1,3	4.2	1.2	27.0	0.461	12.8	4.0	21.2	1.691	2591	885	423	0.85	1.08	1.03	EM	3.7	E. coli K-12		Carbone, et al. (2020) *NComm*[292]
7K00	a,A	0.7	0.6	0.4	0.313	-0.6	1.3	-44.0	0.532	2625	995	429	0.63	0.82	0.70	EM	2.0	E. coli		Watson, et al. (2020) *Elife*[293]
7K50	1,3	-0.9	1.1	8.2	0.854	0.8	1.2	-84.1	1.093	2587	965	432	0.92	0.93	0.93	EM	3.4	E. coli K-12		Demo, et al. (2021) *NComm*[294]
7K51	1,3	4.8	1.1	43.0	0.749	15.5	5.9	16.6	1.741	2566	764	329	0.95	1.22	1.20	EM	3.5	E. coli K-12		Demo, et al. (2021) *NComm*[294]
7K52	1,3	0.8	1.0	15.3	0.631	-0.7	1.6	-59.6	0.759	2585	917	437	0.92	1.03	1.02	EM	3.4	E. coli K-12		Demo, et al. (2021) *NComm*[294]
7K53	1,3	-1.3	1.1	16.3	1.061	1.3	1.3	-91.8	1.287	2590	976	445	0.88	0.98	0.91	EM	3.2	E. coli K-12		Demo, et al. (2021) *NComm*[294]
7K54	1,3	4.1	1.3	34.1	0.763	14.3	5.2	12.8	2.235	2587	856	376	0.91	1.19	1.16	EM	3.2	E. coli K-12		Demo, et al. (2021) *NComm*[294]
7K55	1,3	-0.5	1.2	13.4	1.014	0.4	2.0	-78.0	1.218	2585	866	435	0.94	1.00	1.03	EM	3.3	E. coli K-12		Demo, et al. (2021) *NComm*[294]
7KGB	A,a	-0.2	1.4	-16.0	1.616	-0.1	1.4	-78.8	1.535	2154	858	411	1.12	1.10	0.90	EM	2.7	Mycobacterium tuberculosis H37Rv		Cui, et al. *To be published*
7L08	A,AA	8.4	2.3	105.0	2.273	3.9	2.4	-130.3	2.071	933	372	158	1.15	1.28	1.28	EM	3.5	Homo sapiens	*	Koripella, et al. (2021) *NComm*[295]
7LH5	BA,AA	0.0	0.7	-24.7	0.338	-0.5	0.9	-38.6	0.174	2128	881	390	0.94	0.88	0.86	XRAY	3.3	T. thermophilus HB8		Golkar, et al. (2021) *Commun Biol*[296]
7LH5	DA,CA	1.3	0.3	-9.6	0.791	-1.1	1.2	-37.3	0.195	2089	861	402	0.93	0.92	0.88	XRAY	3.3	T. thermophilus HB8		Golkar, et al. (2021) *Commun Biol*[296]
7LS1	A2,m2	0.0	3.4	29.6	0.884	15.6	4.7	65.1	0.773	1343	521	209	1.26	1.26	1.28	EM	3.3	Mus musculus		Smith, et al. (2021) *NComm*[297]
7LS2	A2,m2	5.9	4.7	39.4	0.911	12.0	7.2	66.7	0.336	1351	563	216	1.24	1.28	1.26	EM	3.1	Mus musculus		Smith, et al. (2021) *NComm*[297]
7LV0	1,3	9.1	2.5	17.2	1.613	4.5	2.0	-32.4	0.550	2530	956	428	0.91	0.94	0.93	EM	3.2	E. coli K-12		Demo, et al. (2021) *NComm*[294]
7M4W	A,a	1.9	2.5	54.8	4.379	8.4	1.9	-49.0	3.414	1574	720	359	1.42	1.38	1.13	EM	2.5	Acinetobacter baumannii AB0057		Zhang, et al. (2021) *mBio*[298]
7M4X	A,a	0.1	1.4	41.8	3.989	0.4	2.3	-76.5	4.002	1532	722	383	1.42	1.40	1.16	EM	2.7	Acinetobacter baumannii AB0057		Zhang, et al. (2021) *mBio*[298]
7M4Y	A,a	3.2	1.6	64.3	4.286	13.3	2.0	-25.3	3.166	1579	723	354	1.42	1.38	1.08	EM	2.5	Acinetobacter baumannii AB0057		Zhang, et al. (2021) *mBio*[298]
7M4Z	A,a	0.8	1.8	34.4	4.056	-0.9	2.9	-81.6	3.385	1569	741	358	1.43	1.38	1.18	EM	2.9	Acinetobacter baumannii AB0057		Zhang, et al. (2021) *mBio*[298]
7M5D	1,2	-0.5	0.7	14.9	0.379	0.4	1.3	-69.0	0.287	2669	1001	431	0.70	0.67	0.78	EM	2.8	E. coli		Zhang *To be published*
7MD7	1A,1a	1.7	0.7	3.5	0.384	-1.4	1.3	-56.5	0.658	2095	852	416	0.97	1.00	0.87	XRAY	2.8	T. thermophilus HB8		Chen, et al. (2021) *Antibiotics (Basel)*[299]
7MD7	2A,2a	-1.0	0.9	-2.6	0.610	1.3	1.3	-79.5	0.411	2147	880	421	0.95	0.97	0.96	XRAY	2.8	T. thermophilus HB8		Chen, et al. (2021) *Antibiotics (Basel)*[299]
7MDZ	5,9	-2.0	3.0	24.9	1.186	-1.1	1.0	-83.1	1.278	1419	618	283	1.20	1.21	1.15	EM	3.2	Oryctolagus cuniculus		Koga, et al. (2021) *J Am Chem Soc*[300]
7MPI	A1,B5	3.2	3.4	40.1	0.454	12.9	5.3	68.7	0.430	1394	599	251	1.24	1.20	1.13	EM	3.0	S. cerevisiae		Zhao, et al. (2022) *Structure*[301]
7MPJ	A1,B5	3.1	3.6	41.7	0.518	12.8	5.0	69.0	0.407	1433	620	231	1.21	1.16	1.10	EM	2.7	S. cerevisiae		Zhao, et al. (2022) *Structure*[301]
7MSC	A,a	-0.1	1.3	-10.6	1.844	0.7	1.7	-85.8	1.582	2147	856	412	1.15	1.15	0.98	EM	3.0	Mycobacterium tuberculosis H37Rv		Cui, et al. (2022) *NComm*[302]
7MSH	A,a	4.2	0.8	73.0	2.162	-0.1	2.2	-34.3	0.815	2079	792	407	1.19	1.21	1.06	EM	3.2	Mycobacterium tuberculosis H37Rv		Cui, et al. (2022) *NComm*[302]
7MSM	A,a	0.0	1.3	-12.8	1.716	0.7	1.2	-85.4	1.528	2127	865	407	1.15	1.14	0.97	EM	2.8	Mycobacterium tuberculosis H37Rv		Cui, et al. (2022) *NComm*[302]
7MSZ	A,a	3.1	0.2	23.4	1.718	-1.2	1.3	-34.0	1.049	2074	795	405	1.17	1.19	1.05	EM	3.1	Mycobacterium tuberculosis H37Rv		Cui, et al. (2022) *NComm*[302]
7MT2	A,a	-0.4	1.4	-14.8	1.751	0.3	2.0	-78.0	1.561	2147	859	408	1.14	1.15	0.97	EM	2.8	Mycobacterium tuberculosis H37Rv		Cui, et al. (2022) *NComm*[302]
7MT3	A,a	9.5	2.6	15.0	2.373	2.8	2.3	-45.3	0.946	2082	816	410	1.16	1.20	0.99	EM	2.8	Mycobacterium tuberculosis H37Rv		Cui, et al. (2022) *NComm*[302]
7MT7	A,a	-0.4	1.5	-14.4	1.763	0.2	2.1	-75.6	1.573	2171	868	417	1.12	1.12	0.96	EM	2.7	Mycobacterium tuberculosis H37Rv		Cui, et al. (2022) *NComm*[302]
7N1P	23,16	1.5	0.4	5.8	0.233	-0.9	1.6	-38.2	0.419	2608	979	440	0.59	0.91	0.71	EM	2.3	E. coli K-12		Rundlet, et al. (2021) *Nature*[303]
7N2C	23,16	7.3	1.6	70.5	0.833	15.9	6.2	16.4	1.015	2548	924	387	0.68	0.95	0.83	EM	2.7	E. coli K-12		Rundlet, et al. (2021) *Nature*[303]
7N2U	23,16	9.8	2.7	16.7	1.504	4.2	2.2	-33.1	0.551	2537	964	438	0.65	0.70	0.80	EM	2.5	E. coli K-12		Rundlet, et al. (2021) *Nature*[303]
7N2V	23,16	9.3	2.7	21.2	1.738	6.4	3.9	-41.7	0.626	2517	934	435	0.71	0.82	0.83	EM	2.5	E. coli K-12		Rundlet, et al. (2021) *Nature*[303]
7N30	23,16	9.1	1.3	47.0	0.728	2.6	2.3	-25.0	0.858	2558	962	438	0.66	1.00	0.80	EM	2.7	E. coli K-12		Rundlet, et al. (2021) *Nature*[303]

Table 21 of 24

PDB		BODY				HEAD				PRUNED			RMSD			EXP DETAILS		ORGANISM		REFERENCE
ID	chains	ϕ_{body}	θ_{body}	ψ_{body}	$\lvert\Delta\vec{x}_{\text{body}}\rvert$	ϕ_{head}	θ_{head}	ψ_{head}	$\lvert\Delta\vec{x}_{\text{head}}\rvert$	LSU	body	head	LSU	body	head	method	res.	name	mito	
7N31	23,16	-1.4	1.2	13.3	0.720	1.1	2.0	-88.2	1.131	2600	995	444	0.60	0.74	0.73	EM	2.7	E. coli K-12		Rundlet, et al. (2021) *Nature*[303]
7N8B	A1,B5	3.2	3.3	39.6	0.445	10.8	4.6	53.6	0.420	1423	609	246	1.23	1.19	1.08	EM	3.0	S. cerevisiae		Zhao, et al. (2022) *Structure*[301]
7NBU	a,A	-1.6	1.3	9.3	0.320	1.4	1.9	-93.9	1.124	2642	995	433	0.64	0.80	0.76	EM	3.1	E. coli K-12		Mansour, et al. (2022) *NComm*[304]
7NHK	A,a	4.6	2.2	94.1	0.821	-0.8	0.8	-61.6	0.501	2202	839	413	1.02	1.17	0.91	EM	2.9	Enterococcus faecalis		Crowe-McAuliffe, et al. (2021) *NComm*[305]
7NHL	A,a	-0.1	2.7	37.7	1.342	2.4	1.2	-110.1	1.154	2266	891	404	1.06	1.10	0.92	EM	3.1	Staph. aureus subsp. aureus NCTC 8325		Crowe-McAuliffe, et al. (2021) *NComm*[305]
7NHM	A,a	-0.8	1.6	18.0	0.874	0.5	1.9	-107.0	1.801	2334	901	407	1.01	1.06	0.90	EM	3.1	Staph. aureus subsp. aureus NCTC 8325		Crowe-McAuliffe, et al. (2021) *NComm*[305]
7NHN	A,a	4.3	2.8	77.7	0.844	0.3	1.5	6.2	0.651	2241	844	407	1.03	1.18	0.91	EM	2.9	Listeria monocytogenes EGD-e		Crowe-McAuliffe, et al. (2021) *NComm*[305]
7NQH	BA,AA	-0.4	1.2	-148.4	2.322	0.6	3.7	-110.2	1.573	1030	358	201	1.06	1.17	1.16	EM	3.5	Sus scrofa	*	Kummer, et al. (2021) *Mol Cell*[306]
7NQL	BA,AA	-0.2	0.9	-140.4	2.246	1.4	2.7	-126.2	1.750	1042	347	198	1.05	1.13	1.14	EM	3.4	Sus scrofa	*	Kummer, et al. (2021) *Mol Cell*[306]
7NRC	LA,S2	3.6	3.2	81.7	1.605	-1.3	1.1	-19.6	1.196	1385	597	284	1.25	1.20	1.16	EM	3.9	S. cerevisiae S288C		Pochopien, et al. (2021) *PNAS*[307]
7NRD	LA,S2	8.5	5.7	36.1	0.877	0.6	1.0	-22.2	0.576	1329	608	287	1.26	1.19	1.20	EM	4.4	S. cerevisiae S288C		Pochopien, et al. (2021) *PNAS*[307]
7NSI	BA,AA	6.3	2.0	116.1	1.807	5.0	1.1	-123.8	1.739	1026	352	194	1.08	1.15	1.18	EM	4.6	Sus scrofa	*	Kummer, et al. (2021) *Mol Cell*[306]
7NSJ	BA,AA	0.4	0.6	159.0	2.269	1.0	3.0	-124.4	2.055	1029	349	198	1.07	1.10	1.19	EM	3.9	Sus scrofa	*	Kummer, et al. (2021) *Mol Cell*[306]
7NSO	A,a	-1.6	1.3	10.5	0.815	1.0	1.9	-95.3	1.282	2616	994	436	0.78	0.83	0.83	EM	2.9	E. coli		Beckert, et al. (2021) *NComm*[308]
7NSP	A,a	0.9	1.1	11.2	0.494	-1.0	1.3	-52.5	0.463	2590	972	434	0.89	0.97	0.89	EM	3.5	E. coli		Beckert, et al. (2021) *NComm*[308]
7NSQ	A,a	1.1	0.5	0.7	0.235	-0.9	1.4	-38.3	0.249	2626	990	440	0.69	0.92	0.79	EM	3.1	E. coli		Beckert, et al. (2021) *NComm*[308]
7NWG	51,A2	8.9	5.8	38.2	3.301	1.7	1.0	11.8	2.406	615	376	190	1.49	1.43	1.36	EM	3.8	Oryctolagus cuniculus		Powers, et al. (2021) *NAR*[309]
7NWH	5,9	5.5	4.5	39.7	3.525	-0.4	1.3	139.2	2.240	622	339	166	1.48	1.45	1.37	EM	4.1	Oryctolagus cuniculus		Powers, et al. (2021) *NAR*[309]
7NWI	5,9	-2.0	2.8	13.1	3.344	-0.4	1.2	-69.7	2.698	621	370	214	1.47	1.43	1.31	EM	3.1	Oryctolagus cuniculus		Powers, et al. (2021) *NAR*[309]
7NWT	1,2	-1.5	1.6	10.5	1.670	1.0	1.8	-95.5	1.904	2592	990	420	1.03	1.02	0.85	EM	2.7	E. coli		Hill, et al. (2021) *NComm*
7NWW	1,2	-1.7	1.6	17.8	0.804	1.3	2.0	-101.9	1.084	2607	1001	430	0.76	0.84	0.79	EM	3.0	E. coli		Agirrezabala, et al. (2022) *EMBO J*[310]
7O19	BA,AA	-1.3	1.4	9.6	1.805	0.7	1.7	-100.2	1.842	2543	967	436	1.14	1.07	0.96	EM	2.9	E. coli K-12		van der Stel, et al. (2021) *NComm*[311]
7O1A	BA,AA	-1.4	1.4	8.2	1.802	1.0	1.7	-93.5	1.951	2518	965	430	1.16	1.09	0.95	EM	2.4	E. coli K-12		van der Stel, et al. (2021) *NComm*[311]
7O1C	BA,AA	-1.2	1.5	10.1	1.961	0.9	1.8	-95.5	1.821	2509	960	435	1.19	1.11	0.96	EM	2.6	E. coli K-12		van der Stel, et al. (2021) *NComm*[311]
7O5B	X,A	-1.2	1.8	10.1	1.594	0.9	1.5	-126.5	1.901	2221	828	369	1.11	1.17	1.06	EM	3.3	B. subtilis subsp. subtilis str. 168		Czech, et al. (2022) *NComm*[312]
7O7Y	B5,A2	-2.1	3.0	21.2	1.412	-1.1	1.2	-70.6	1.709	1421	603	226	1.20	1.17	1.16	EM	2.2	Oryctolagus cuniculus		Bhatt, et al. (2021) *Science*[313]
7O7Z	B5,A2	-2.1	2.9	20.6	1.412	-1.1	1.3	-67.6	1.699	1416	599	225	1.20	1.17	1.16	EM	2.4	Oryctolagus cuniculus		Bhatt, et al. (2021) *Science*[313]
7O80	B5,A2	-2.1	2.7	31.4	1.003	-1.6	1.6	-78.5	1.360	1436	633	209	1.21	1.17	1.15	EM	2.9	Oryctolagus cuniculus		Bhatt, et al. (2021) *Science*[313]
7O81	B5,A2	8.8	5.8	40.2	0.020	0.1	1.3	-14.7	1.354	1374	619	201	1.22	1.21	1.19	EM	3.1	Oryctolagus cuniculus		Bhatt, et al. (2021) *Science*[313]
7OG4	XA,AA	1.1	0.1	119.3	2.237	2.3	2.8	-159.0	1.650	985	342	178	1.08	1.21	1.22	EM	3.8	Homo sapiens	*	Aibara, et al. (2020) *Elife*[282]
7OIF	1,2	-1.7	1.6	18.4	0.809	1.3	1.9	-102.3	1.096	2616	1001	428	0.76	0.83	0.79	EM	3.0	E. coli		Agirrezabala, et al. (2022) *EMBO J*[310]
7OIG	1,2	-1.7	1.5	18.5	0.817	1.3	1.9	-103.2	1.139	2607	1000	428	0.79	0.85	0.81	EM	3.2	E. coli		Agirrezabala, et al. (2022) *EMBO J*[310]
7OII	1,2	-1.5	1.4	15.4	0.929	1.2	1.9	-93.4	1.264	2613	1001	433	0.76	0.83	0.83	EM	3.0	E. coli		Agirrezabala, et al. (2022) *EMBO J*[310]
7OIZ	a,A	-1.2	1.2	6.1	1.088	0.6	1.6	-89.3	1.175	2607	951	442	0.87	0.96	0.86	EM	2.9	E. coli K-12		Su, et al. (2021) *NAR*[314]
7OJ0	a,A	-1.3	1.3	9.5	0.717	1.2	1.8	-89.1	0.811	2639	990	439	0.70	0.80	0.83	EM	3.5	E. coli K-12		Su, et al. (2021) *NAR*[314]
7OLC	1,2	-2.1	3.0	0.2	3.884	1.1	2.3	-53.2	2.952	617	357	230	1.47	1.39	1.33	EM	2.9	Chaetomium thermophilum var. thermophilum DSM 1495		Kisonaite, et al. (2022) *NComm*[315]
7OLD	1,2	4.6	4.0	27.0	3.895	13.9	5.3	71.1	2.865	608	380	214	1.47	1.38	1.31	EM	3.0	Chaetomium thermophilum var. thermophilum DSM 1495		Kisonaite, et al. (2022) *NComm*[315]
7OSA	25S,18S	8.9	5.8	20.5	1.642	1.0	2.8	-46.2	1.526	1404	575	287	1.24	1.20	1.12	XRAY	3.0	S. cerevisiae		Djumagulov, et al. (2021) *Nature*[316]
7OSM	25S,18S	7.0	5.7	27.2	1.371	12.4	4.8	58.2	0.373	1390	594	259	1.23	1.18	1.20	XRAY	3.0	S. cerevisiae		Djumagulov, et al. (2021) *Nature*[316]
7OT5	1,2	-1.5	1.4	16.2	0.917	1.2	1.9	-93.5	1.232	2609	998	433	0.77	0.83	0.82	EM	2.9	E. coli		Agirrezabala, et al. (2022) *EMBO J*[310]
7OTC	A,a	3.6	1.2	38.3	0.133	17.0	4.7	11.5	1.333	2604	967	373	0.68	0.91	0.91	EM	2.9	E. coli BL21(DE3)		Wieland, et al. (2022) *PNAS*[317]
7OYA	51,22	-1.8	2.6	23.1	1.376	0.5	1.3	-8.7	1.340	1380	597	286	1.22	1.20	1.20	EM	3.2	Danio rerio		Leesch, et al. *To be published*
7OYB	51,22	3.4	4.6	45.7	0.362	11.6	4.5	73.7	0.183	1422	590	221	1.18	1.19	1.17	EM	2.4	Danio rerio		Leesch, et al. *To be published*
7OYC	51,22	4.5	4.1	44.0	0.715	13.2	6.9	68.1	0.343	1415	603	256	1.20	1.17	1.17	EM	2.4	Xenopus laevis		Leesch, et al. *To be published*
7OYD	5,9	4.6	4.6	39.3	1.580	12.5	7.6	71.1	0.222	1322	607	224	1.24	1.25	1.19	EM	2.3	Oryctolagus cuniculus		Leesch, et al. *To be published*
7P3K	a,A	-1.4	1.2	12.6	1.109	0.6	1.6	-89.7	1.185	2607	948	442	0.87	0.96	0.86	EM	2.9	E. coli K-12		Su, et al. (2021) *NAR*[314]
7P48	A,a	1.2	2.5	54.9	3.490	2.2	1.7	-113.2	2.581	1462	699	377	1.40	1.36	1.20	EM	2.9	Staph. aureus		Mohamad, et al. (2022) *NAR*[318]
7P6Z	3,5	2.7	1.1	105.7	1.850	-0.7	1.0	177.0	1.700	2087	752	384	1.15	1.19	1.11	EM	3.5	Mycoplasma pneumoniae M129		Xue, et al. (2022) *Nature*[319]
7P7Q	A,a	-0.7	2.1	25.2	1.077	2.3	1.2	-110.0	1.501	2305	923	419	0.93	0.93	0.83	EM	2.4	Enterococcus faecalis		Crowe-McAuliffe, et al. (2022) *NComm*[320]
7P7R	A,a	-1.0	2.5	24.6	1.228	2.1	1.3	-108.5	1.303	2299	912	417	0.94	0.96	0.82	EM	2.9	Enterococcus faecalis V583		Crowe-McAuliffe, et al. (2022) *NComm*[320]
7P7S	A,a	-0.9	2.1	29.7	1.205	2.4	1.5	-101.1	1.440	2303	916	414	0.94	0.99	0.85	EM	3.0	Enterococcus faecalis		Crowe-McAuliffe, et al. (2022) *NComm*[320]
7P7T	A,a	3.8	1.9	105.4	1.405	-0.8	0.8	-2.6	0.712	2292	865	416	0.94	1.20	0.78	EM	2.9	Enterococcus faecalis		Crowe-McAuliffe, et al. (2022) *NComm*[320]

PDB		BODY				HEAD				PRUNED			RMSD			EXP DETAILS		ORGANISM		REFERENCE
ID	chains	ϕ_{body}	θ_{body}	ψ_{body}	$\lvert\Delta\vec{x}_{\text{body}}\rvert$	ϕ_{head}	θ_{head}	ψ_{head}	$\lvert\Delta\vec{x}_{\text{head}}\rvert$	LSU	body	head	LSU	body	head	method	res.	name	mito	
7P7U	A,a	-1.3	1.9	14.6	0.647	0.8	1.8	-118.0	1.731	2337	912	416	0.93	0.94	0.83	EM	3.1	Enterococcus faecalis		Crowe-McAuliffe, et al. (2022) *NComm*[320]
7PAH	3,5	1.2	0.7	132.6	1.662	0.0	1.0	-146.4	1.658	1694	708	315	1.33	1.30	1.28	EM	9.5	Mycoplasma pneumoniae M129		Xue, et al. (2022) *Nature*[319]
7PAI	3,5	0.6	0.6	90.4	1.681	0.4	1.1	-166.1	1.664	1831	750	351	1.32	1.26	1.26	EM	6.7	Mycoplasma pneumoniae M129		Xue, et al. (2022) *Nature*[319]
7PAJ	3,5	-0.7	0.5	50.6	1.783	0.9	1.9	-165.9	1.765	1782	751	360	1.31	1.27	1.21	EM	7.3	Mycoplasma pneumoniae M129		Xue, et al. (2022) *Nature*[319]
7PAK	3,5	-0.8	0.5	45.6	1.549	1.0	1.9	-161.2	1.885	1848	678	337	1.28	1.27	1.26	EM	5.3	Mycoplasma pneumoniae M129		Xue, et al. (2022) *Nature*[319]
7PAL	3,5	3.2	1.4	110.0	2.056	-0.4	0.8	174.9	1.632	2028	738	372	1.19	1.19	1.14	EM	4.7	Mycoplasma pneumoniae M129		Xue, et al. (2022) *Nature*[319]
7PAM	3,5	8.2	1.3	65.3	1.927	3.3	1.2	-80.8	1.479	1818	741	340	1.29	1.27	1.24	EM	6.8	Mycoplasma pneumoniae M129		Xue, et al. (2022) *Nature*[319]
7PAN	3,5	9.6	1.7	27.3	2.264	4.0	1.4	-72.8	1.598	1603	659	304	1.32	1.31	1.30	EM	9.7	Mycoplasma pneumoniae M129		Xue, et al. (2022) *Nature*[319]
7PAO	3,5	9.3	1.9	35.3	2.375	3.2	1.3	-86.6	1.636	1799	723	344	1.29	1.24	1.25	EM	7.0	Mycoplasma pneumoniae M129		Xue, et al. (2022) *Nature*[319]
7PAQ	3,5	8.4	1.4	50.1	2.311	6.2	2.0	-60.4	1.679	1687	662	310	1.32	1.30	1.30	EM	8.9	Mycoplasma pneumoniae M129		Xue, et al. (2022) *Nature*[319]
7PAR	3,5	5.0	1.2	90.0	1.892	16.6	3.7	3.8	1.875	1730	702	289	1.32	1.27	1.38	EM	8.2	Mycoplasma pneumoniae M129		Xue, et al. (2022) *Nature*[319]
7PAS	3,5	8.3	1.9	23.2	1.031	2.4	2.8	-6.5	1.130	853	288	110	1.45	1.44	1.42	EM	16.0	Mycoplasma pneumoniae M129		Xue, et al. (2022) *Nature*[319]
7PH9	3,5	0.1	0.5	74.2	1.802	0.2	1.6	-151.4	1.712	1652	702	318	1.34	1.31	1.28	EM	8.7	Mycoplasma pneumoniae M129		Xue, et al. (2022) *Nature*[319]
7PHA	3,5	-0.8	0.3	51.3	1.813	1.1	1.6	-152.3	1.642	1564	648	313	1.36	1.35	1.31	EM	8.5	Mycoplasma pneumoniae M129		Xue, et al. (2022) *Nature*[319]
7PHB	3,5	3.4	1.5	119.4	2.164	-0.3	1.0	-170.1	1.721	1945	708	368	1.22	1.21	1.18	EM	4.9	Mycoplasma pneumoniae M129		Xue, et al. (2022) *Nature*[319]
7PHC	3,5	7.9	0.9	84.5	2.441	3.0	1.1	-82.7	1.303	1386	491	239	1.41	1.38	1.40	EM	9.9	Mycoplasma pneumoniae M129		Xue, et al. (2022) *Nature*[319]
7PI8	3,5	1.4	0.9	112.8	1.568	0.3	0.9	165.9	1.589	1486	621	279	1.36	1.34	1.36	EM	8.9	Mycoplasma pneumoniae M129		Xue, et al. (2022) *Nature*[319]
7PI9	3,5	-0.9	0.6	43.8	1.729	1.1	1.9	-165.4	1.814	1717	679	329	1.34	1.36	1.25	EM	6.3	Mycoplasma pneumoniae M129		Xue, et al. (2022) *Nature*[319]
7PIA	3,5	7.9	0.6	92.1	2.034	5.8	2.1	-36.6	1.020	1176	374	163	1.41	1.39	1.43	EM	13.6	Mycoplasma pneumoniae M129		Xue, et al. (2022) *Nature*[319]
7PIB	3,5	8.4	1.1	98.8	1.819	8.4	3.2	-20.2	1.894	1854	695	328	1.24	1.26	1.26	EM	4.7	Mycoplasma pneumoniae M129		Xue, et al. (2022) *Nature*[319]
7PIC	3,5	8.5	1.9	53.4	2.247	4.4	0.8	-121.2	1.993	1290	498	233	1.40	1.37	1.41	EM	9.1	Mycoplasma pneumoniae M129		Xue, et al. (2022) *Nature*[319]
7PIO	3,5	0.6	0.5	106.7	1.847	0.3	1.3	-137.0	1.496	1653	668	306	1.34	1.33	1.35	EM	9.5	Mycoplasma pneumoniae M129		Xue, et al. (2022) *Nature*[319]
7PIP	3,5	-0.8	0.4	56.0	1.734	1.2	1.6	-145.2	1.662	1721	722	309	1.34	1.31	1.33	EM	9.3	Mycoplasma pneumoniae M129		Xue, et al. (2022) *Nature*[319]
7PIQ	3,5	3.1	1.2	106.5	2.288	0.3	0.7	-49.1	1.691	1621	633	243	1.34	1.34	1.40	EM	9.7	Mycoplasma pneumoniae M129		Xue, et al. (2022) *Nature*[319]
7PIR	3,5	8.1	1.1	61.2	2.059	3.6	0.8	-86.0	0.815	1329	459	200	1.41	1.43	1.39	EM	12.1	Mycoplasma pneumoniae M129		Xue, et al. (2022) *Nature*[319]
7PIS	3,5	8.6	1.6	29.4	2.345	3.2	2.6	-89.9	1.365	1262	404	206	1.39	1.43	1.38	EM	15.0	Mycoplasma pneumoniae M129		Xue, et al. (2022) *Nature*[319]
7PIT	3,5	9.7	2.0	28.4	2.462	3.4	2.4	-82.7	1.753	1826	702	339	1.29	1.28	1.23	EM	5.7	Mycoplasma pneumoniae M129		Xue, et al. (2021) *Biorxiv*
7PJS	A,a	1.1	0.6	-1.2	0.792	-0.8	1.6	-37.8	0.943	2631	979	434	0.62	0.92	0.75	EM	2.4	E. coli		Petrychenko, et al. (2021) *NComm*
7PJT	A,a	9.9	2.1	17.8	0.827	3.2	2.5	-32.3	1.134	2526	885	398	1.01	1.16	1.14	EM	6.0	E. coli		Petrychenko, et al. (2021) *NComm*[321]
7PJU	A,a	10.7	2.4	8.0	1.284	3.4	3.0	-34.2	1.423	2550	948	419	0.71	0.98	0.89	EM	9.5	E. coli K-12		Petrychenko, et al. (2021) *NComm*[321]
7PJV	A,a	10.7	2.5	8.1	1.298	3.5	3.0	-34.8	1.416	2552	943	423	0.73	0.98	0.90	EM	3.1	E. coli K-12		Petrychenko, et al. (2021) *NComm*[321]
7PJW	A,a	10.8	2.5	7.5	1.372	2.9	3.1	-33.9	1.347	2549	941	417	0.81	1.03	0.95	EM	4.0	E. coli		Petrychenko, et al. (2021) *NComm*[321]
7PJX	A,a	10.7	2.4	7.5	1.277	3.5	3.1	-34.9	1.419	2553	935	424	0.73	0.98	0.90	EM	6.5	E. coli		Petrychenko, et al. (2021) *NComm*[321]
7PJY	A,a	4.5	1.5	28.3	1.061	17.0	5.2	16.7	1.237	2598	877	377	0.70	1.10	0.89	EM	3.1	E. coli K-12		Petrychenko, et al. (2021) *NComm*[321]
7PJZ	A,a	4.3	1.6	28.0	0.906	17.0	5.1	15.9	1.320	2552	817	362	0.96	1.17	1.06	EM	6.0	E. coli		Petrychenko, et al. (2021) *NComm*[321]
7PWO	1,2	-2.6	3.1	18.3	2.926	-0.6	1.6	-86.6	3.196	912	456	242	1.42	1.39	1.25	EM	2.8	Giardia lamblia ATCC 50803		Hiregange, et al. (2022) *NAR*[322]
7PZY	1,A	-1.3	2.8	17.8	0.918	13.5	6.0	71.7	0.731	1402	608	262	1.22	1.17	1.16	EM	2.3	Candida albicans SC5314		Zgadzay, et al. (2022) *Sci Adv*[323]
7Q08	1,A	-0.9	2.7	25.7	0.888	13.2	6.4	75.4	0.804	1395	601	255	1.22	1.21	1.23	EM	2.6	Candida albicans SC5314		Zgadzay, et al. (2022) *Sci Adv*[323]
7Q0F	1,A	-1.0	2.8	25.4	0.821	13.6	6.5	76.9	0.695	1415	610	260	1.23	1.21	1.20	EM	2.6	Candida albicans SC5314		Zgadzay, et al. (2022) *Sci Adv*[323]
7Q0P	1,A	-1.3	2.8	17.8	0.919	13.5	6.0	71.8	0.750	1402	608	260	1.22	1.17	1.16	EM	2.8	Candida albicans SC5314		Zgadzay, et al. (2022) *Sci Adv*[323]
7Q0R	1,A	-1.3	2.8	17.8	0.918	13.5	6.0	71.8	0.750	1402	608	260	1.22	1.17	1.16	EM	2.7	Candida albicans SC5314		Zgadzay, et al. (2022) *Sci Adv*[323]
7QEP	1,3	2.6	2.9	61.7	4.301	13.5	2.1	36.3	2.750	532	352	186	1.46	1.46	1.32	EM	2.7	Encephalitozoon cuniculi GB-M1		Nicholson, et al. (2022) *NComm*
7QG8	N,0	0.4	2.0	26.0	3.570	1.2	1.8	-99.7	1.130	2638	999	438	0.60	0.70	0.71	EM	4.0	E. coli K-12		Saito, et al. (2022) *Nature*[324]
7QGG	t,S2	8.7	5.6	38.4	1.691	1.0	1.3	-2.5	1.370	1152	526	223	1.33	1.31	1.25	EM	2.9	Rattus norvegicus		Kipper, et al. *To be published*
7QGH	N,0	2.4	1.9	38.0	2.566	1.2	1.8	-100.0	1.144	2638	1008	438	0.60	0.72	0.71	EM	4.5	E. coli K-12		Saito, et al. (2022) *Nature*[324]
7QGN	N,0	0.4	2.0	26.0	3.570	1.2	1.8	-100.0	1.130	2638	999	439	0.60	0.70	0.71	EM	3.4	E. coli		Saito, et al. (2022) *Nature*[324]
7QGR	N,0	2.4	1.9	38.0	2.565	1.2	1.8	-100.3	1.144	2638	1008	439	0.60	0.72	0.71	EM	5.7	E. coli		Saito, et al. (2022) *Nature*[324]
7QGU	A,W	-0.4	2.0	14.2	1.275	1.0	1.5	-130.6	1.508	2295	913	421	1.04	1.00	0.89	EM	4.8	B. subtilis		Saito, et al. (2022) *Nature*[324]
7QH4	A,W	2.3	1.4	43.5	1.049	1.0	1.5	-130.5	1.506	2289	913	421	1.04	1.00	0.89	EM	5.5	B. subtilis		Saito, et al. (2022) *Nature*[324]
7QI4	A,AA	0.5	0.3	-139.6	2.690	1.5	2.5	-149.8	1.805	1009	342	203	1.00	1.16	1.09	EM	2.2	Homo sapiens	*	Singh, et al. *To be published*
7QIZ	2,S2	-0.3	2.4	25.6	1.368	0.4	1.0	15.4	1.255	1345	598	249	1.23	1.15	1.18	EM	2.4	Solanum lycopersicum	*	Cottilli, et al. *To be published*
7QV1	V,a	1.2	1.4	65.8	2.115	-0.1	1.9	-107.6	1.663	2183	834	383	1.18	1.20	1.19	EM	3.5	B. subtilis subsp. subtilis str. 168		Cerullo, et al. (2022) *Nature*[325]

PDB		BODY				HEAD				PRUNED			RMSD			EXP DETAILS		ORGANISM		REFERENCE
ID	chains	ϕ_{body}	θ_{body}	ψ_{body}	$\lvert\Delta\vec{x}_{\text{body}}\rvert$	ϕ_{head}	θ_{head}	ψ_{head}	$\lvert\Delta\vec{x}_{\text{head}}\rvert$	LSU	body	head	LSU	body	head	method	res.	name	mito	
7QV2	V,a	8.3	2.5	46.2	1.872	2.5	1.2	-77.6	1.115	2190	824	408	1.16	1.18	1.04	EM	3.5	B. subtilis subsp. subtilis str. 168		Cerullo, et al. (2022) *Nature*[325]
7QV3	V,a	1.2	1.4	65.8	2.115	-0.1	1.9	-107.6	1.663	2180	834	383	1.18	1.20	1.19	EM	5.1	B. subtilis subsp. subtilis str. 168		Cerullo, et al. (2022) *Nature*[325]
7QVP	L5,S2	-2.2	2.8	19.4	1.840	-0.8	1.1	-78.5	1.563	1354	590	287	1.25	1.22	1.14	EM	3.0	Homo sapiens		Narita, et al. *To be published*
7QVP	L6,S3	8.7	5.6	40.5	1.054	0.9	1.2	-9.5	1.066	1329	585	281	1.26	1.23	1.18	EM	3.0	Homo sapiens		Narita, et al. *To be published*
7R81	A1,A2	1.7	3.0	49.6	1.087	-1.2	1.1	-15.8	1.145	1331	609	287	1.27	1.21	1.20	EM	2.7	Neurospora crassa		Shen, et al. (2021) *PNAS*[326]
7RQ8	1A,1a	1.5	0.4	-5.3	0.464	-0.9	1.3	-29.6	0.266	2102	855	414	0.96	0.96	0.84	XRAY	2.5	T. thermophilus HB8		Mitcheltree, et al. (2021) *Nature*[327]
7RQ8	2A,2a	-0.2	0.8	-17.1	0.309	0.3	1.1	-42.7	0.305	2115	891	414	0.95	0.92	0.89	XRAY	2.5	T. thermophilus HB8		Mitcheltree, et al. (2021) *Nature*[327]
7RQ9	1A,1a	1.5	0.5	-24.3	1.203	-0.8	1.4	-28.8	0.369	2087	858	414	0.97	0.93	0.85	XRAY	2.6	T. thermophilus HB8		Mitcheltree, et al. (2021) *Nature*[327]
7RQ9	2A,2a	0.1	0.7	-16.9	0.478	-0.1	0.9	-35.2	0.317	2115	895	410	0.95	0.93	0.90	XRAY	2.6	T. thermophilus HB8		Mitcheltree, et al. (2021) *Nature*[327]
7RQA	1A,1a	1.6	0.8	9.6	0.568	-1.4	1.3	-55.9	0.682	2126	860	415	0.95	0.96	0.89	XRAY	2.4	T. thermophilus HB8		Syroegin, et al. (2022) *NSMB*[328]
7RQA	2A,2a	-1.0	1.0	-6.3	0.233	1.1	1.1	-69.2	0.111	2161	886	413	0.96	0.97	0.91	XRAY	2.4	T. thermophilus HB8		Syroegin, et al. (2022) *NSMB*[328]
7RQB	1A,1a	1.7	0.6	3.6	0.572	-1.3	1.4	-54.6	0.778	2127	858	417	0.96	0.97	0.85	XRAY	2.5	T. thermophilus HB8		Syroegin, et al. (2022) *NSMB*[328]
7RQB	2A,2a	-1.1	0.9	-8.4	0.330	1.5	1.4	-74.4	0.346	2155	883	418	0.95	0.97	0.91	XRAY	2.5	T. thermophilus HB8		Syroegin, et al. (2022) *NSMB*[328]
7RQC	1A,1a	1.7	0.6	4.3	0.503	-1.3	1.3	-57.5	0.825	2119	853	421	0.98	0.99	0.88	XRAY	2.5	T. thermophilus HB8		Syroegin, et al. (2022) *NSMB*[328]
7RQC	2A,2a	-1.1	1.0	-4.4	0.531	1.4	1.4	-77.1	0.282	2133	881	421	0.96	1.00	0.94	XRAY	2.5	T. thermophilus HB8		Syroegin, et al. (2022) *NSMB*[328]
7RQD	1A,1a	1.6	0.6	7.7	0.376	-1.2	1.3	-53.2	0.697	2121	860	412	0.96	0.97	0.87	XRAY	2.5	T. thermophilus HB8		Syroegin, et al. (2022) *NSMB*[328]
7RQD	2A,2a	-1.1	1.0	-5.3	0.488	1.4	1.3	-76.9	0.402	2141	875	416	0.96	0.99	0.94	XRAY	2.5	T. thermophilus HB8		Syroegin, et al. (2022) *NSMB*[328]
7RQE	1A,1a	1.6	0.6	2.3	0.435	-1.3	1.4	-56.0	0.767	2125	853	421	0.98	0.99	0.89	XRAY	2.4	T. thermophilus HB8		Syroegin, et al. (2022) *NSMB*[328]
7RQE	2A,2a	-1.1	1.0	-5.4	0.588	1.4	1.4	-75.6	0.456	2144	879	418	0.97	1.00	0.94	XRAY	2.4	T. thermophilus HB8		Syroegin, et al. (2022) *NSMB*[328]
7RR5	C1,C2	3.2	3.0	73.2	2.093	-1.6	1.3	-19.4	1.448	1150	566	281	1.32	1.29	1.22	EM	3.2	S. cerevisiae		Zeng, et al. (2021) *Cell Rep*[329]
7RYF	A,a	2.8	1.1	75.4	4.160	0.9	2.2	-85.9	4.817	1293	537	335	1.46	1.35	1.18	EM	2.6	Acinetobacter baumannii AB0057		Morgan, et al. (2022) *mBio*[330]
7RYG	A,a	3.6	1.3	78.7	4.241	14.2	1.4	100.3	4.078	1317	553	336	1.45	1.35	1.15	EM	2.4	Acinetobacter baumannii AB0057		Morgan, et al. (2022) *mBio*[330]
7RYH	A,a	5.7	2.2	106.9	4.185	10.6	1.2	-144.7	4.070	1295	540	340	1.46	1.34	1.17	EM	2.4	Acinetobacter baumannii AB0057		Morgan, et al. (2022) *mBio*[330]
7S1G	I,C	-1.6	1.3	5.9	0.879	1.0	1.6	-97.3	1.271	2613	996	435	0.76	0.81	0.78	EM	2.5	E. coli		Tsai, et al. (2022) *NSMB*[331]
7S1H	I,C	-1.4	1.5	11.6	0.689	1.1	1.7	-90.3	1.259	2609	998	435	0.77	0.78	0.81	EM	2.4	E. coli		Tsai, et al. (2022) *NSMB*[331]
7S1I	I,C	-1.6	1.4	6.5	0.960	1.2	1.6	-97.5	1.255	2610	992	435	0.77	0.81	0.76	EM	2.5	E. coli		Tsai, et al. (2022) *NSMB*[331]
7S1J	I,C	-1.6	1.5	11.2	0.853	1.2	1.7	-96.9	1.347	2609	998	433	0.79	0.82	0.83	EM	2.5	E. coli		Tsai, et al. (2022) *NSMB*[331]
7S1K	I,C	-1.6	1.4	10.8	0.838	1.1	1.7	-98.2	1.396	2602	995	435	0.83	0.85	0.80	EM	2.4	E. coli		Tsai, et al. (2022) *NSMB*[331]
7SA4	1,2	-1.7	1.5	14.7	0.374	1.1	2.2	-95.8	1.079	2630	997	434	0.74	0.87	0.74	EM	2.5	E. coli		Tian, et al. (2022) *PNAS*[332]
7SFR	A,a	-0.1	1.5	-10.7	1.888	-0.1	1.4	-80.9	1.759	2091	827	387	1.17	1.19	1.15	EM	2.6	Mycobacterium tuberculosis		Zhang *To be published*
7SS9	1,3	-0.4	1.3	15.2	0.526	19.5	3.8	14.7	1.346	2524	881	322	1.04	1.12	1.21	EM	3.9	E. coli K-12		Carbone, et al. (2021) *NComm*[333]
7SSD	1,3	3.3	1.2	27.0	0.487	17.3	5.3	14.6	1.272	2595	847	365	0.83	1.21	1.11	EM	3.3	E. coli K-12		Carbone, et al. (2021) *NComm*[333]
7SSL	1,3	10.0	2.6	12.9	1.470	4.0	2.8	-35.6	0.589	2499	923	379	0.96	1.06	1.14	EM	3.8	E. coli K-12		Carbone, et al. (2021) *NComm*[333]
7SSN	1,3	9.5	2.3	13.4	1.602	4.2	2.3	-36.1	0.787	2555	933	431	0.86	0.92	0.93	EM	3.2	E. coli K-12		Carbone, et al. (2021) *NComm*[333]
7SSO	1,3	9.2	1.5	16.5	1.307	3.1	2.1	-31.3	0.959	2560	929	435	0.85	0.97	0.95	EM	3.2	E. coli K-12		Carbone, et al. (2021) *NComm*[333]
7SSW	1,3	-0.4	1.5	12.4	0.529	20.1	3.8	9.7	1.970	2539	911	322	0.99	1.07	1.23	EM	3.8	E. coli K-12		Carbone, et al. (2021) *NComm*[333]
7ST2	1,3	-1.5	1.2	11.3	0.505	1.0	1.9	-91.0	0.984	2626	993	448	0.62	0.74	0.75	EM	2.9	E. coli K-12		Carbone, et al. (2021) *NComm*[333]
7ST6	1,3	-0.9	1.0	9.2	0.767	0.9	1.0	-82.0	0.809	2616	979	437	0.89	0.86	0.86	EM	3.0	E. coli K-12		Carbone, et al. (2021) *NComm*[333]
7ST7	1,3	10.7	2.5	8.1	1.276	3.2	2.9	-27.8	0.506	2539	960	433	0.80	0.99	0.90	EM	3.2	E. coli K-12		Carbone, et al. (2021) *NComm*[333]
7TOQ	A25S,A18S	7.7	5.2	37.8	0.342	2.4	1.7	-22.8	0.947	1388	554	258	1.24	1.29	1.26	EM	3.1	Oryctolagus cuniculus		Loveland, et al. (2022) *NComm*[334]
7TOR	A28S,A18S	-2.5	3.5	8.0	2.866	-0.5	0.8	-81.6	1.270	1359	597	284	1.26	1.24	1.21	EM	2.9	Oryctolagus cuniculus		Loveland, et al. (2022) *NComm*[334]
7TOS	23S,16S	-1.4	1.5	15.4	0.436	1.5	2.0	-85.6	0.700	2624	993	445	0.74	0.78	0.86	EM	2.9	E. coli		Loveland, et al. (2022) *NComm*[334]
7U2H	1A,1a	1.4	0.4	-34.1	0.547	-0.7	1.5	-32.9	0.366	2073	848	418	1.00	0.99	0.89	XRAY	2.5	T. thermophilus HB8		Syroegin, et al. (2022) *NAR*[335]
7U2H	2A,2a	-0.3	0.7	-24.1	0.635	0.3	1.1	-37.8	0.421	2102	889	413	0.99	0.96	0.91	XRAY	2.5	T. thermophilus HB8		Syroegin, et al. (2022) *NAR*[335]
7U2I	1A,1a	1.4	0.4	-25.5	0.482	-0.6	1.4	-31.6	0.285	2086	847	420	0.98	0.98	0.90	XRAY	2.5	T. thermophilus HB8		Syroegin, et al. (2022) *NAR*[335]
7U2I	2A,2a	-0.3	0.8	-26.4	0.506	0.4	1.1	-32.8	0.333	2115	884	414	0.98	0.94	0.92	XRAY	2.5	T. thermophilus HB8		Syroegin, et al. (2022) *NAR*[335]
7U2J	1A,1a	1.3	0.3	-37.6	0.631	-0.6	1.4	-28.3	0.088	2116	878	417	0.93	0.90	0.85	XRAY	2.5	T. thermophilus HB8		Syroegin, et al. (2022) *NAR*[335]
7U2J	2A,2a	-0.2	0.8	-26.0	0.085	0.3	1.0	-24.9	0.069	2131	888	416	0.93	0.89	0.89	XRAY	2.5	T. thermophilus HB8		Syroegin, et al. (2022) *NAR*[335]
7UCJ	5,9	-2.2	2.8	17.8	2.289	-0.5	1.1	-73.7	1.809	1214	557	251	1.29	1.29	1.23	EM	3.1	Oryctolagus cuniculus		Arango, et al. (2022) *Mol Cell*[336]
7UCK	5,9	-2.1	2.8	19.2	2.151	-0.4	1.3	-69.0	1.821	1210	559	252	1.29	1.28	1.21	EM	2.8	Oryctolagus cuniculus		Arango, et al. (2022) *Mol Cell*[336]
7UG7	23,16	4.2	0.8	44.2	0.215	17.7	5.7	13.5	1.351	2603	896	365	0.64	1.12	0.89	EM	2.6	E. coli		Wieland, et al. (2022) *PNAS*[317]
7UNR	A,a	0.5	1.2	19.2	2.098	-1.2	1.3	-79.2	2.286	2045	804	416	1.32	1.28	1.11	EM	2.9	Pseudomonas aeruginosa PAO1		Basu, et al. (2022) *NComm*

Table 24 of 24

PDB		BODY				HEAD				PRUNED			RMSD			EXP DETAILS		ORGANISM		REFERENCE
ID	chains	ϕ_{body}	θ_{body}	ψ_{body}	$\lvert\Delta\vec{x}_{\text{body}}\rvert$	ϕ_{head}	θ_{head}	ψ_{head}	$\lvert\Delta\vec{x}_{\text{head}}\rvert$	LSU	body	head	LSU	body	head	method	res.	name	mito	
7UNU	A,a	-1.6	1.4	13.0	2.543	0.6	1.7	-105.9	2.539	2055	821	411	1.32	1.32	1.11	EM	2.9	Pseudomonas aeruginosa PAO1		Basu, et al. (2022) *NComm*
7UNV	A,a	2.0	1.4	58.2	2.663	1.9	1.8	-37.3	2.022	2050	774	410	1.30	1.30	1.12	EM	2.7	Pseudomonas aeruginosa PAO1		Basu, et al. (2022) *NComm*
7UNW	A,a	1.8	1.4	50.5	2.712	1.8	1.8	-37.7	2.009	2065	777	407	1.31	1.30	1.14	EM	2.6	Pseudomonas aeruginosa PAO1		Basu, et al. (2022) *NComm*
7ZJW	L5,S2	-0.8	3.2	26.8	1.158	-0.4	1.2	9.0	0.915	1374	629	198	1.22	1.20	1.19	EM	2.8	Oryctolagus cuniculus		Hilal, et al. (2022) *Science*[337]
7ZJX	L5,S2	-1.9	2.9	23.2	0.947	6.2	4.1	61.8	0.218	1440	604	187	1.19	1.16	1.14	EM	3.1	Oryctolagus cuniculus		Hilal, et al. (2022) *Science*[337]
7ZW0	LA,2	9.0	6.1	31.5	1.588	0.2	1.6	-40.2	1.272	1338	588	294	1.27	1.19	1.16	EM	2.4	S. cerevisiae W303		Li, et al. (2022) *Mol Cell*[338]
8CVJ	1A,1a	1.4	0.5	-25.7	0.509	-0.7	1.3	-21.0	0.464	2029	830	412	1.04	1.02	0.89	XRAY	2.4	T. thermophilus HB8		Syroegin, et al. (2022) *Nat Chem*
8CVJ	2A,2a	0.1	0.7	-23.7	0.890	-0.1	0.9	-17.8	0.554	2082	892	413	1.02	0.98	0.92	XRAY	2.4	T. thermophilus HB8		Syroegin, et al. (2022) *Nat Chem*
8CVK	1A,1a	1.4	0.4	-16.0	0.673	-0.6	1.3	-23.7	0.340	2085	856	412	0.99	0.97	0.88	XRAY	2.5	T. thermophilus HB8		Syroegin, et al. (2022) *Nat Chem*
8CVK	2A,2a	0.1	0.8	-27.2	0.275	0.0	0.9	-18.0	0.412	2094	887	414	1.01	0.96	0.91	XRAY	2.5	T. thermophilus HB8		Syroegin, et al. (2022) *Nat Chem*
8CVL	1A,1a	1.5	0.4	-22.4	0.783	-0.8	1.4	-22.8	0.082	2087	862	412	0.95	0.97	0.82	XRAY	2.3	T. thermophilus HB8		Syroegin, et al. (2022) *Nat Chem*
8CVL	2A,2a	0.1	0.7	-30.4	0.261	-0.1	1.0	-24.2	0.053	2114	889	413	0.94	0.89	0.85	XRAY	2.3	T. thermophilus HB8		Syroegin, et al. (2022) *Nat Chem*

References

[1] Bruno P. Klaholz, Tillmann Pape, Andrey V. Zavialov, Alexander G. Myasnikov, Elena V. Orlova, Bente Vestergaard, Måns Ehrenberg, and Marin van Heel. Structure of the Escherichia coli ribosomal termination complex with release factor 2. *Nature*, 421(6918):90–94, jan 2003.

[2] Tatsuya Maehigashi, Jack A. Dunkle, Stacey J. Miles, and Christine M. Dunham. Structural insights into +1 frameshifting promoted by expanded or modification-deficient anticodon stem loops. *Proceedings of the National Academy of Sciences*, 111(35):12740–12745, aug 2014.

[3] Yury S Polikanov, Thomas A Steitz, and C Axel Innis. A proton wire to couple aminoacyl-tRNA accommodation and peptide-bond formation on the ribosome. *Nature Structural & Molecular Biology*, 21(9):787–793, aug 2014.

[4] Cha San Koh, Axel F. Brilot, Nikolaus Grigorieff, and Andrei A. Korostelev. Taura syndrome virus IRES initiates translation by binding its tRNA-mRNA–like structural element in the ribosomal decoding center. *Proceedings of the National Academy of Sciences*, 111(25):9139–9144, jun 2014.

[5] Egor Svidritskiy, Axel F. Brilot, Cha San Koh, Nikolaus Grigorieff, and Andrei A. Korostelev. Structures of Yeast 80S Ribosome-tRNA Complexes in the Rotated and Nonrotated Conformations. *Structure*, 22(8):1210–1218, aug 2014.

[6] Rebecca M. Voorhees, Israel S. Fernández, Sjors H.W. Scheres, and Ramanujan S. Hegde. Structure of the Mammalian Ribosome-Sec61 Complex to 3.4 Å Resolution. *Cell*, 157(7):1632–1643, jun 2014.

[7] Alexey Amunts, Alan Brown, Jaan Toots, Sjors H. W. Scheres, and V. Ramakrishnan. The structure of the human mitochondrial ribosome. *Science*, 348(6230):95–98, apr 2015.

[8] Daniel Sohmen, Shinobu Chiba, Naomi Shimokawa-Chiba, C. Axel Innis, Otto Berninghausen, Roland Beckmann, Koreaki Ito, and Daniel N. Wilson. Structure of the Bacillus subtilis 70S ribosome reveals the basis for species-specific stalling. *Nature Communications*, 6(1), apr 2015.

[9] Stefan Arenz, Fabian Nguyen, Roland Beckmann, and Daniel N. Wilson. Cryo-EM structure of the tetracycline resistance protein TetM in complex with a translating ribosome at 3.9-Å resolution. *Proceedings of the National Academy of Sciences*, 112(17):5401–5406, apr 2015.

[10] Wen Li, Zheng Liu, Ravi Kiran Koripella, Robert Langlois, Suparna Sanyal, and Joachim Frank. Activation of GTP hydrolysis in mRNA-tRNA translocation by elongation factor G. *Science Advances*, 1(4), may 2015.

[11] Alan Brown, Sichen Shao, Jason Murray, Ramanujan S. Hegde, and V. Ramakrishnan. Structural basis for stop codon recognition in eukaryotes. *Nature*, 524(7566):493–496, aug 2015.

[12] Rebecca M Voorhees and Ramanujan S Hegde. Structures of the scanning and engaged states of the mammalian SRP-ribosome complex. *eLife*, 4, jul 2015.

[13] Ming Sun, Wen Li, Karin Blomqvist, Sanchaita Das, Yaser Hashem, Jeffrey D. Dvorin, and Joachim Frank. Dynamical features of thePlasmodium falciparumribosome during translation. *Nucleic Acids Research*, page gkv991, oct 2015.

[14] Jun Zhang, Xijiang Pan, Kaige Yan, Shan Sun, Ning Gao, and Sen-Fang Sui. Mechanisms of ribosome stalling by SecM at multiple elongation steps. *eLife*, 4, dec 2015.

[15] Dejiu Zhang, Kaige Yan, Guangqiao Liu, Guangtao Song, Jiejian Luo, Yi Shi, Erchao Cheng, Shan Wu, Taijiao Jiang, Jizhong Lou, Ning Gao, and Yan Qin. EF4 disengages the peptidyl-tRNA CCA end and facilitates back-translocation on the 70S ribosome. *Nature Structural & Molecular Biology*, 23(2):125–131, jan 2016.

[16] Thiemo Sprink, David J. F. Ramrath, Hiroshi Yamamoto, Kaori Yamamoto, Justus Loerke, Jochen Ismer, Peter W. Hildebrand, Patrick Scheerer, Jörg Bürger, Thorsten Mielke, and Christian M. T. Spahn. Structures of ribosome-bound initiation factor 2 reveal the mechanism of subunit association. *Science Advances*, 2(3), mar 2016.

[17] Arren Z. Washington, Derek B. Benicewicz, Joshua C. Canzoneri, Crystal E. Fagan, Sandra C. Mwakwari, Tatsuya Maehigashi, Christine M. Dunham, and Adegboyega K. Oyelere. Macrolide-Peptide Conjugates as Probes of the Path of Travel of the Nascent Peptides through the Ribosome. *ACS Chemical Biology*, 9(11):2621–2631, sep 2014.

[18] Crystal E. Fagan, Tatsuya Maehigashi, Jack A. Dunkle, Stacey J. Miles, and Christine M. Dunham. Structural insights into translational recoding by frameshift suppressor tRNA^{S}ufJ. *RNA*, 20(12):1944–1954, oct 2014.

[19] Jonas Noeske, Jian Huang, Nelson B. Olivier, Robert A. Giacobbe, Mark Zambrowski, and Jamie H. D. Cate. Synergy of Streptogramin Antibiotics Occurs Independently of Their Effects on Translation. *Antimicrobial Agents and Chemotherapy*, 58(9):5269–5279, sep 2014.

[20] Nicolas Garreau de Loubresse, Irina Prokhorova, Wolf Holtkamp, Marina V. Rodnina, Gulnara Yusupova, and Marat Yusupov. Structural basis for the inhibition of the eukaryotic ribosome. *Nature*, 513(7519):517–522, sep 2014.

[21] Heena Khatter, Alexander G. Myasnikov, S. Kundhavai Natchiar, and Bruno P. Klaholz. Structure of the human 80S ribosome. *Nature*, 520(7549):640–645, apr 2015.

[22] Hiroshi Yamamoto, Anett Unbehaun, Justus Loerke, Elmar Behrmann, Marianne Collier, Jörg Bürger, Thorsten Mielke, and Christian M T Spahn. Structure of the mammalian 80S initiation complex with initiation factor 5B on HCV-IRES RNA. *Nature Structural & Molecular Biology*, 21(8):721–727, jul 2014.

[23] Tatyana V. Budkevich, Jan Giesebrecht, Elmar Behrmann, Justus Loerke, David J.F. Ramrath, Thorsten Mielke, Jochen Ismer, Peter W. Hildebrand, Chang-Shung Tung, Knud H. Nierhaus, Karissa Y. Sanbonmatsu, and Christian M.T. Spahn. Regulation of the Mammalian Elongation Cycle by Subunit Rolling: A Eukaryotic-Specific Ribosome Rearrangement. *Cell*, 158(1):121–131, jul 2014.

[24] Alexander G. Myasnikov, Zhanna A. Afonina, Jean-François Ménétret, Vladimir A. Shirokov, Alexander S. Spirin, and Bruno P. Klaholz. The molecular structure of the left-handed supra-molecular helix of eukaryotic polyribosomes. *Nature Communications*, 5(1), nov 2014.

[25] Marat M. Yusupov, Gulnara Zh. Yusupova, Albion Baucom, Kate Lieberman, Thomas N. Earnest, J. H. D. Cate, and Harry F. Noller. Crystal Structure of the Ribosome at 5.5 Å Resolution. *Science*, 292(5518):883–896, may 2001.

[26] Haixiao Gao, Jayati Sengupta, Mikel Valle, Andrei Korostelev, Narayanan Eswar, Scott M. Stagg, Patrick Van Roey, Rajendra K. Agrawal, Stephen C. Harvey, Andrej Sali, Michael S. Chapman, and Joachim Frank. Study of the Structural Dynamics of the E. coli 70S Ribosome Using Real-Space Refinement. *Cell*, 113(6):789–801, jun 2003.

[27] Antón Vila-Sanjurjo, William K. Ridgeway, Veysel Seymaner, Wen Zhang, Steve Santoso, Kexin Yu, and Jamie H. Doudna Cate. X-ray crystal structures of the WT and a hyper-accurate ribosome from Escherichia coli. *Proceedings of the National Academy of Sciences*, 100(15):8682–8687, jul 2003.

[28] Christian MT Spahn, Maria G Gomez-Lorenzo, Robert A Grassucci, Rene Jørgensen, Gregers R Andersen, Roland Beckmann, Pawel A Penczek, Juan PG Ballesta, and Joachim Frank. Domain movements of elongation factor eEF2 and the eukaryotic 80S ribosome facilitate tRNA translocation. *The EMBO Journal*, 23(5):1008–1019, feb 2004.

[29] Antón Vila-Sanjurjo, Barbara-S Schuwirth, Cathy W Hau, and Jamie H D Cate. Structural basis for the control of translation initiation during stress. *Nature Structural & Molecular Biology*, 11(11):1054–1059, oct 2004.

[30] Barbara S Schuwirth, J Michael Day, Cathy W Hau, Gary R Janssen, Albert E Dahlberg, Jamie H Doudna Cate, and Antón Vila-Sanjurjo. Structural analysis of kasugamycin inhibition of translation. *Nature Structural & Molecular Biology*, 13(10):879–886, sep 2006.

[31] Andrei Korostelev, Sergei Trakhanov, Martin Laurberg, and Harry F. Noller. Crystal Structure of a 70S Ribosome-tRNA Complex Reveals Functional Interactions and Rearrangements. *Cell*, 126(6):1065–1077, sep 2006.

[32] Andrei Korostelev, Sergei Trakhanov, Haruichi Asahara, Martin Laurberg, Laura Lancaster, and Harry F. Noller. Interactions and dynamics of the Shine–Dalgarno helix in the 70S ribosome. *Proceedings of the National Academy of Sciences*, 104(43):16840–16843, oct 2007.

[33] Eunyong Park, Jean-François Ménétret, James C. Gumbart, Steven J. Ludtke, Weikai Li, Andrew Whynot, Tom A. Rapoport, and Christopher W. Akey. Structure of the SecY channel during initiation of protein translocation. *Nature*, 506(7486):102–106, oct 2013.

[34] Lasse Jenner, Pascale Romby, Bernard Rees, Clemens Schulze-Briese, Mathias Springer, Chantal Ehresmann, Bernard Ehresmann, Dino Moras, Gulnara Yusupova, and Marat Yusupov. Translational Operator of mRNA on the Ribosome: How Repressor Proteins Exclude Ribosome Binding. *Science*, 308(5718):120–123, apr 2005.

[35] Barbara S. Schuwirth, Maria A. Borovinskaya, Cathy W. Hau, Wen Zhang, Antón Vila-Sanjurjo, James M. Holton, and Jamie H. Doudna Cate. Structures of the Bacterial Ribosome at 3.5 å Resolution. *Science*, 310(5749):827–834, nov 2005.

[36] Sabine Petry, Ditlev E. Brodersen, Frank V. Murphy, Christine M. Dunham, Maria Selmer, Michael J. Tarry, Ann C. Kelley, and V. Ramakrishnan. Crystal Structures of the Ribosome in Complex with Release Factors RF1 and RF2 Bound to a Cognate Stop Codon. *Cell*, 123(7):1255–1266, dec 2005.

[37] Kakoli Mitra, Christiane Schaffitzel, Felcy Fabiola, Michael S. Chapman, Nenad Ban, and Joachim Frank. Elongation Arrest by SecM via a Cascade of Ribosomal RNA Rearrangements. *Molecular Cell*, 22(4):533–543, may 2006.

[38] Gulnara Yusupova, Lasse Jenner, Bernard Rees, Dino Moras, and Marat Yusupov. Structural basis for messenger RNA movement on the ribosome. *Nature*, 444(7117):391–394, oct 2006.

[39] Veysel Berk, Wen Zhang, Raj D. Pai, and Jamie H. D. Cate. Structural basis for mRNA and tRNA positioning on the ribosome. *Proceedings of the National Academy of Sciences*, 103(43):15830–15834, oct 2006.

[40] Maria Selmer, Christine M. Dunham, Frank V. Murphy, Albert Weixlbaumer, Sabine Petry, Ann C. Kelley, John R. Weir, and V. Ramakrishnan. Structure of the 70 S Ribosome Complexed with mRNA and tRNA. *Science*, 313(5795):1935–1942, sep 2006.

[41] Maria A Borovinskaya, Raj D Pai, Wen Zhang, Barbara S Schuwirth, James M Holton, Go Hirokawa, Hideko Kaji, Akira Kaji, and Jamie H Doudna Cate. Structural basis for aminoglycoside inhibition of bacterial ribosome recycling. *Nature Structural & Molecular Biology*, 14(8):727–732, jul 2007.

[42] Maria A. Borovinskaya, Shinichiro Shoji, James M. Holton, Kurt Fredrick, and Jamie H. D. Cate. A Steric Block in Translation Caused by the Antibiotic Spectinomycin. *ACS Chemical Biology*, 2(8):545–552, aug 2007.

[43] Albert Weixlbaumer, Sabine Petry, Christine M Dunham, Maria Selmer, Ann C Kelley, and V Ramakrishnan. Crystal structure of the ribosome recycling factor bound to the ribosome. *Nature Structural & Molecular Biology*, 14(8):733–737, jul 2007.

[44] Rouven Bingel-Erlenmeyer, Rebecca Kohler, Günter Kramer, Arzu Sandikci, Snježana Antoli
'c, Timm Maier, Christiane Schaffitzel, Brigitte Wiedmann, Bernd Bukau, and Nenad Ban. A peptide deformylase–ribosome complex reveals mechanism of nascent chain processing. *Nature*, 452(7183):108–111, feb 2008.

[45] Rebecca M Voorhees, Albert Weixlbaumer, David Loakes, Ann C Kelley, and V Ramakrishnan. Insights into substrate stabilization from snapshots of the peptidyl transferase center of the intact 70S ribosome. *Nature Structural & Molecular Biology*, 16(5):528–533, apr 2009.

[46] Albert Weixlbaumer, Hong Jin, Cajetan Neubauer, Rebecca M. Voorhees, Sabine Petry, Ann C. Kelley, and Venki Ramakrishnan. Insights into Translational Termination from the Structure of RF2 Bound to the Ribosome. *Science*, 322(5903):953–956, nov 2008.

[47] Yong-Gui Gao, Maria Selmer, Christine M. Dunham, Albert Weixlbaumer, Ann C. Kelley, and V. Ramakrishnan. The Structure of the Ribosome with Elongation Factor G Trapped in the Posttranslocational State. *Science*, 326(5953):694–699, oct 2009.

[48] T. Martin Schmeing, Rebecca M. Voorhees, Ann C. Kelley, Yong-Gui Gao, Frank V. Murphy, John R. Weir, and V. Ramakrishnan. The Crystal Structure of the Ribosome Bound to EF-Tu and Aminoacyl-tRNA. *Science*, 326(5953):688–694, oct 2009.

[49] Birgit Seidelt, C. Axel Innis, Daniel N. Wilson, Marco Gartmann, Jean-Paul Armache, Elizabeth Villa, Leonardo G. Trabuco, Thomas Becker, Thorsten Mielke, Klaus Schulten, Thomas A. Steitz, and Roland Beckmann. Structural Insight into Nascent Polypeptide Chain–Mediated Translational Stalling. *Science*, 326(5958):1412–1415, dec 2009.

[50] Hong Jin, Ann C. Kelley, David Loakes, and V. Ramakrishnan. Structure of the 70S ribosome bound to release factor 2 and a substrate analog provides insights into catalysis of peptide release. *Proceedings of the National Academy of Sciences*, 107(19):8593–8598, apr 2010.

[51] C Leong Ng, Kathrin Lang, Nicola A G Meenan, Amit Sharma, Ann C Kelley, Colin Kleanthous, and V Ramakrishnan. Structural basis for 16S ribosomal RNA cleavage by the cytotoxic domain of colicin E3. *Nature Structural & Molecular Biology*, 17(10):1241–1246, sep 2010.

[52] Rebecca M. Voorhees, T. Martin Schmeing, Ann C. Kelley, and V. Ramakrishnan. The Mechanism for Activation of GTP Hydrolysis on the Ribosome. *Science*, 330(6005):835–838, nov 2010.

[53] Andreas H. Ratje, Justus Loerke, Aleksandra Mikolajka, Matthias Brünner, Peter W. Hildebrand, Agata L. Starosta, Alexandra Dönhöfer, Sean R. Connell, Paola Fucini, Thorsten Mielke, Paul C. Whitford, Jos
'e N. Onuchic, Yanan Yu, Karissa Y. Sanbonmatsu, Roland K. Hartmann, Pawel A. Penczek, Daniel N. Wilson, and Christian M. T. Spahn. Head swivel on the ribosome facilitates translocation by means of intra-subunit tRNA hybrid sites. *Nature*, 468(7324):713–716, dec 2010.

[54] T Martin Schmeing, Rebecca M Voorhees, Ann C Kelley, and V Ramakrishnan. How mutations in tRNA distant from the anticodon affect the fidelity of decoding. *Nature Structural & Molecular Biology*, 18(4):432–436, mar 2011.

[55] Preethi Chandramouli, Maya Topf, Jean-François Ménétret, Narayanan Eswar, Jamie J. Cannone, Robin R. Gutell, Andrej Sali, and Christopher W. Akey. Structure of the Mammalian 80S Ribosome at 8.7 Å Resolution. *Structure*, 16(4):535–548, apr 2008.

[56] Manjuli R. Sharma, Daniel N. Wilson, Partha P. Datta, Chandana Barat, Frank Schluenzen, Paola Fucini, and Rajendra K. Agrawal. Cryo-EM study of the spinach chloroplast ribosome reveals the structural and functional roles of plastid-specific ribosomal proteins. *Proceedings of the National Academy of Sciences*, 104(49):19315–19320, dec 2007.

[57] Martin Laurberg, Haruichi Asahara, Andrei Korostelev, Jianyu Zhu, Sergei Trakhanov, and Harry F. Noller. Structural basis for translation termination on the 70S ribosome. *Nature*, 454(7206):852–857, jul 2008.

[58] Maria A. Borovinskaya, Shinichiro Shoji, Kurt Fredrick, and Jamie H.D. Cate. Structural basis for hygromycin B inhibition of protein biosynthesis. *RNA*, 14(8):1590–1599, jun 2008.

[59] Andrei Korostelev, Haruichi Asahara, Laura Lancaster, Martin Laurberg, Alexander Hirschi, Jianyu Zhu, Sergei Trakhanov, William G. Scott, and Harry F. Noller. Crystal structure of a translation termination complex formed with release factor RF2. *Proceedings of the National Academy of Sciences*, 105(50):19684–19689, dec 2008.

[60] Jan-Christian Schuette, Frank V Murphy, Ann C Kelley, John R Weir, Jan Giesebrecht, Sean R Connell, Justus Loerke, Thorsten Mielke, Wei Zhang, Pawel A Penczek, V Ramakrishnan, and Christian M T Spahn. GTPase activation of elongation factor EF-Tu by the ribosome during decoding. *The EMBO Journal*, 28(6):755–765, feb 2009.

[61] Elizabeth Villa, Jayati Sengupta, Leonardo G. Trabuco, Jamie LeBarron, William T. Baxter, Tanvir R. Shaikh, Robert A. Grassucci, Poul Nissen, Måns Ehrenberg, Klaus Schulten, and Joachim Frank. Ribosome-induced changes in elongation factor Tu conformation control GTP hydrolysis. *Proceedings of the National Academy of Sciences*, 106(4):1063–1068, jan 2009.

[62] Gregor Blaha, Robin E. Stanley, and Thomas A. Steitz. Formation of the First Peptide Bond: The Structure of EF-P Bound to the 70 S Ribosome. *Science*, 325(5943):966–970, aug 2009.

[63] Wen Zhang, Jack A. Dunkle, and Jamie H. D. Cate. Structures of the Ribosome in Intermediate States of Ratcheting. *Science*, 325(5943):1014–1017, aug 2009.

[64] Lasse B Jenner, Natalia Demeshkina, Gulnara Yusupova, and Marat Yusupov. Structural aspects of messenger RNA reading frame maintenance by the ribosome. *Nature Structural & Molecular Biology*, 17(5):555–560, apr 2010.

[65] Jean-Paul Armache, Alexander Jarasch, Andreas M. Anger, Elizabeth Villa, Thomas Becker, Shashi Bhushan, Fabrice Jossinet, Michael Habeck, Gülcin Dindar, Sibylle Franckenberg, Viter Marquez, Thorsten Mielke, Michael Thomm, Otto Berninghausen, Birgitta Beatrix, Johannes Söding, Eric Westhof, Daniel N. Wilson, and Roland Beckmann. Cryo-EM structure and rRNA model of a translating eukaryotic 80S ribosome at 5.5-Å resolution. *Proceedings of the National Academy of Sciences*, 107(46):19748–19753, oct 2010.

[66] Xabier Agirrezabala, Eduard Schreiner, Leonardo G Trabuco, Jianlin Lei, Rodrigo F Ortiz-Meoz, Klaus Schulten, Rachel Green, and Joachim Frank. Structural insights into cognate versus near-cognate discrimination during decoding. *The EMBO Journal*, 30(8):1497–1507, mar 2011.

[67] Jens Frauenfeld, James Gumbart, Eli O van der Sluis, Soledad Funes, Marco Gartmann, Birgitta Beatrix, Thorsten Mielke, Otto Berninghausen, Thomas Becker, Klaus Schulten, and Roland Beckmann. Cryo-EM structure of the ribosome–SecYE complex in the membrane environment. *Nature Structural & Molecular Biology*, 18(5):614–621, apr 2011.

[68] Xabier Agirrezabala, Hstau Y. Liao, Eduard Schreiner, Jie Fu, Rodrigo F. Ortiz-Meoz, Klaus Schulten, Rachel Green, and Joachim Frank. Structural characterization of mRNA-tRNA translocation intermediates. *Proceedings of the National Academy of Sciences*, 109(16):6094–6099, mar 2012.

[69] David J. F. Ramrath, Hiroshi Yamamoto, Kristian Rother, Daniela Wittek, Markus Pech, Thorsten Mielke, Justus Loerke, Patrick Scheerer, Pavel Ivanov, Yoshika Teraoka, Olga Shpanchenko, Knud H. Nierhaus, and Christian M. T. Spahn. The complex of tmRNA–SmpB and EF-G on translocating ribosomes. *Nature*, 485(7399):526–529, may 2012.

[70] Jean-Paul Armache, Andreas M. Anger, Viter M
'arquez, Sibylle Franckenberg, Thomas Fröhlich, Elizabeth Villa, Otto Berninghausen, Michael Thomm, Georg J. Arnold, Roland Beckmann, and Daniel N. Wilson. Promiscuous behaviour of archaeal ribosomal proteins: Implications for eukaryotic ribosome evolution. *Nucleic Acids Research*, 41(2):1284–1293, dec 2013.

[71] Wen Li, Gemma C. Atkinson, Nehal S. Thakor, Ülar Allas, Chuao chao Lu, Kwok-Yan Chan, Tanel Tenson, Klaus Schulten, Kevin S. Wilson, Vasili Hauryliuk, and Joachim Frank. Mechanism of tetracycline resistance by ribosomal protection protein Tet(O). *Nature Communications*, 4(1), feb 2013.

[72] Andreas M. Anger, Jean-Paul Armache, Otto Berninghausen, Michael Habeck, Marion Subklewe, Daniel N. Wilson, and Roland Beckmann. Structures of the human and Drosophila 80S ribosome. *Nature*, 497(7447):80–85, may 2013.

[73] Lars V Bock, Christian Blau, Gunnar F Schröder, Iakov I Davydov, Niels Fischer, Holger Stark, Marina V Rodnina, Andrea C Vaiana, and Helmut Grubmüller. Energy barriers and driving forces in tRNA translocation through the ribosome. *Nature Structural & Molecular Biology*, 20(12):1390–1396, nov 2013.

[74] David J. F. Ramrath, Laura Lancaster, Thiemo Sprink, Thorsten Mielke, Justus Loerke, Harry F. Noller, and Christian M. T. Spahn. Visualization of two transfer RNAs trapped in transit during elongation factor G-mediated translocation. *Proceedings of the National Academy of Sciences*, 110(52):20964–20969, dec 2013.

[75] Axel F. Brilot, Andrei A. Korostelev, Dmitri N. Ermolenko, and Nikolaus Grigorieff. Structure of the ribosome with elongation factor G trapped in the pretranslocation state. *Proceedings of the National Academy of Sciences*, 110(52):20994–20999, dec 2013.

[76] Marko Gogala, Thomas Becker, Birgitta Beatrix, Jean-Paul Armache, Clara Barrio-Garcia, Otto Berninghausen, and Roland Beckmann. Structures of the Sec61 complex engaged in nascent peptide translocation or membrane insertion. *Nature*, 506(7486):107–110, feb 2014.

[77] Derek J. Taylor, Batsal Devkota, Andrew D. Huang, Maya Topf, Eswar Narayanan, Andrej Sali, Stephen C. Harvey, and Joachim Frank. Comprehensive Molecular Structure of the Eukaryotic Ribosome. *Structure*, 17(12):1591–1604, dec 2009.

[78] James Gumbart, Leonardo G. Trabuco, Eduard Schreiner, Elizabeth Villa, and Klaus Schulten. Regulation of the Protein-Conducting Channel by a Bound Ribosome. *Structure*, 17(11):1453–1464, nov 2009.

[79] Cajetan Neubauer, Yong-Gui Gao, Kasper R. Andersen, Christine M. Dunham, Ann C. Kelley, Jendrik Hentschel, Kenn Gerdes, V. Ramakrishnan, and Ditlev E. Brodersen. The Structural Basis for mRNA Recognition and Cleavage by the Ribosome-Dependent Endonuclease RelE. *Cell*, 139(6):1084–1095, dec 2009.

[80] Robin E Stanley, Gregor Blaha, Robert L Grodzicki, Michael D Strickler, and Thomas A Steitz. The structures of the anti-tuberculosis antibiotics viomycin and capreomycin bound to the 70S ribosome. *Nature Structural & Molecular Biology*, 17(3):289–293, feb 2010.

[81] Andrei Korostelev, Jianyu Zhu, Haruichi Asahara, and Harry F Noller. Recognition of the amber UAG stop codon by release factor RF1. *The EMBO Journal*, 29(15):2577–2585, jun 2010.

[82] Adam Ben-Shem, Lasse Jenner, Gulnara Yusupova, and Marat Yusupov. Crystal Structure of the Eukaryotic Ribosome. *Science*, 330(6008):1203–1209, nov 2010.

[83] Jack A. Dunkle, Liqun Xiong, Alexander S. Mankin, and Jamie H. D. Cate. Structures of the Escherichia coli ribosome with antibiotics bound near the peptidyl transferase center explain spectra of drug action. *Proceedings of the National Academy of Sciences*, 107(40):17152–17157, sep 2010.

[84] David Bulkley, C. Axel Innis, Gregor Blaha, and Thomas A. Steitz. Revisiting the structures of several antibiotics bound to the bacterial ribosome. *Proceedings of the National Academy of Sciences*, 107(40):17158–17163, sep 2010.

[85] Jianyu Zhu, Andrei Korostelev, David A. Costantino, John P. Donohue, Harry F. Noller, and Jeffrey S. Kieft. Crystal structures of complexes containing domains from two viral internal ribosome entry site (IRES) RNAs bound to the 70S ribosome. *Proceedings of the National Academy of Sciences*, 108(5):1839–1844, jan 2011.

[86] Jie Zhou, Laura Lancaster, Sergei Trakhanov, and Harry F. Noller. Crystal structure of release factor RF3 trapped in the GTP state on a rotated conformation of the ribosome. *RNA*, 18(2):230–240, dec 2012.

[87] Natalia Demeshkina, Lasse Jenner, Eric Westhof, Marat Yusupov, and Gulnara Yusupova. A new understanding of the decoding principle on the ribosome. *Nature*, 484(7393):256–259, mar 2012.

[88] Adam Ben-Shem, Nicolas Garreau de Loubresse, Sergey Melnikov, Lasse Jenner, Gulnara Yusupova, and Marat Yusupov. The Structure of the Eukaryotic Ribosome at 3.0 Å Resolution. *Science*, 334(6062):1524–1529, dec 2011.

[89] David Bulkley, Francis Johnson, and Thomas A. Steitz. The Antibiotic Thermorubin Inhibits Protein Synthesis by Binding to Inter-Subunit Bridge B2a of the Ribosome. *Journal of Molecular Biology*, 416(4):571–578, mar 2012.

[90] Yury S. Polikanov, Gregor M. Blaha, and Thomas A. Steitz. How Hibernation Factors RMF, HPF, and YfiA Turn Off Protein Synthesis. *Science*, 336(6083):915–918, may 2012.

[91] Crystal E. Fagan, Jack A. Dunkle, Tatsuya Maehigashi, Mai N. Dang, Aishwarya Devaraj, Stacey J. Miles, Daoming Qin, Kurt Fredrick, and Christine M. Dunham. Reorganization of an intersubunit bridge induced by disparate 16S ribosomal ambiguity mutations mimics an EF-Tu-bound state. *Proceedings of the National Academy of Sciences*, 110(24):9716–9721, apr 2013.

[92] Rebecca M Voorhees, Debabrata Mandal, Cajetan Neubauer, Caroline Köhrer, Uttam L RajBhandary, and V Ramakrishnan. The structural basis for specific decoding of AUA by isoleucine tRNA on the ribosome. *Nature Structural & Molecular Biology*, 20(5):641–643, mar 2013.

[93] Hong Jin, Ann C. Kelley, and V. Ramakrishnan. Crystal structure of the hybrid state of ribosome in complex with the guanosine triphosphatase release factor 3. *Proceedings of the National Academy of Sciences*, 108(38):15798–15803, sep 2011.

[94] Cajetan Neubauer, Reynald Gillet, Ann C. Kelley, and V. Ramakrishnan. Decoding in the Absence of a Codon by tmRNA and SmpB in the Ribosome. *Science*, 335(6074):1366–1369, mar 2012.

[95] Shu Feng, Yun Chen, and Yong-Gui Gao. Crystal Structure of 70S Ribosome with Both Cognate tRNAs in the E and P Sites Representing an Authentic Elongation Complex. *PLoS ONE*, 8(3):e58829, mar 2013.

[96] Shu Feng, Yun Chen, Katsuhiko Kamada, Han Wang, Kai Tang, Meitian Wang, and Yong-Gui Gao. YoeB–ribosome structure: a canonical RNase that requires the ribosome for its specific activity. *Nucleic Acids Research*, 41(20):9549–9556, aug 2013.

[97] Israel S. Fernández, Xiao-Chen Bai, Tanweer Hussain, Ann C. Kelley, Jon R. Lorsch, V. Ramakrishnan, and Sjors H. W. Scheres. Molecular Architecture of a Eukaryotic Translational Initiation Complex. *Science*, 342(6160), nov 2013.

[98] Yun Chen, Shu Feng, Veerendra Kumar, Rya Ero, and Yong-Gui Gao. Structure of EF-G–ribosome complex in a pretranslocation state. *Nature Structural & Molecular Biology*, 20(9):1077–1084, aug 2013.

[99] Matthieu G. Gagnon, Sai V. Seetharaman, David Bulkley, and Thomas A. Steitz. Structural Basis for the Rescue of Stalled Ribosomes: Structure of YaeJ Bound to the Ribosome. *Science*, 335(6074):1370–1372, mar 2012.

[100] Lasse Jenner, Agata L. Starosta, Daniel S. Terry, Aleksandra Mikolajka, Liudmila Filonava, Marat Yusupov, Scott C. Blanchard, Daniel N. Wilson, and Gulnara Yusupova. Structural basis for potent inhibitory activity of the antibiotic tigecycline during protein synthesis. *Proceedings of the National Academy of Sciences*, 110(10):3812–3816, feb 2013.

[101] Leyi Wang, Arto Pulk, Michael R Wasserman, Michael B Feldman, Roger B Altman, Jamie H Doudna Cate, and Scott C Blanchard. Allosteric control of the ribosome by small-molecule antibiotics. *Nature Structural & Molecular Biology*, 19(9):957–963, aug 2012.

[102] Jack A. Dunkle, Leyi Wang, Michael B. Feldman, Arto Pulk, Vincent B. Chen, Gary J. Kapral, Jonas Noeske, Jane S. Richardson, Scott C. Blanchard, and Jamie H. Doudna Cate. Structures of the Bacterial Ribosome in Classical and Hybrid States of tRNA Binding. *Science*, 332(6032):981–984, may 2011.

[103] David S. Tourigny, Israel S. Fernández, Ann C. Kelley, and V. Ramakrishnan. Elongation Factor G Bound to the Ribosome in an Intermediate State of Translocation. *Science*, 340(6140), jun 2013.

[104] Israel S. Fernández, Chyan Leong Ng, Ann C. Kelley, Guowei Wu, Yi-Tao Yu, and V. Ramakrishnan. Unusual base pairing during the decoding of a stop codon by the ribosome. *Nature*, 500(7460):107–110, jun 2013.

[105] Jie Zhou, Laura Lancaster, John Paul Donohue, and Harry F. Noller. Crystal Structures of EF-G–Ribosome Complexes Trapped in Intermediate States of Translocation. *Science*, 340(6140), jun 2013.

[106] Natalia Santos, Jianyu Zhu, John Paul Donohue, Andrei A. Korostelev, and Harry F. Noller. Crystal Structure of the 70S Ribosome Bound with the Q253P Mutant Form of Release Factor RF2. *Structure*, 21(7):1258–1263, jul 2013.

[107] Arto Pulk and Jamie H. D. Cate. Control of Ribosomal Subunit Rotation by Elongation Factor G. *Science*, 340(6140), jun 2013.

[108] Egor Svidritskiy, Clarence Ling, Dmitri N. Ermolenko, and Andrei A. Korostelev. Blasticidin S inhibits translation by trapping deformed tRNA on the ribosome. *Proceedings of the National Academy of Sciences*, 110(30):12283–12288, jul 2013.

[109] David Bulkley, Letizia Brandi, Yury S. Polikanov, Attilio Fabbretti, Michael O'Connor, Claudio O. Gualerzi, and Thomas A. Steitz. The Antibiotics Dityromycin and GE82832 Bind Protein S12 and Block EF-G-Catalyzed Translocation. *Cell Reports*, 6(2):357–365, jan 2014.

[110] Jie Zhou, Laura Lancaster, John Paul Donohue, and Harry F. Noller. How the ribosome hands the A-site tRNA to the P site during EF-G–catalyzed translocation. *Science*, 345(6201):1188–1191, sep 2014.

[111] Matthieu G. Gagnon, Jinzhong Lin, David Bulkley, and Thomas A. Steitz. Crystal structure of elongation factor 4 bound to a clockwise ratcheted ribosome. *Science*, 345(6197):684–687, aug 2014.

[112] Yury S. Polikanov, Ilya A. Osterman, Teresa Szal, Vadim N. Tashlitsky, Marina V. Serebryakova, Pavel Kusochek, David Bulkley, Irina A. Malanicheva, Tatyana A. Efimenko, Olga V. Efremenkova, Andrey L. Konevega, Karen J. Shaw, Alexey A. Bogdanov, Marina V. Rodnina, Olga A. Dontsova, Alexander S. Mankin, Thomas A. Steitz, and Petr V. Sergiev. Amicoumacin A Inhibits Translation by Stabilizing mRNA Interaction with the Ribosome. *Molecular Cell*, 56(4):531–540, nov 2014.

[113] Yury S. Polikanov, Teresa Szal, Fuyan Jiang, Pulkit Gupta, Ryoichi Matsuda, Masataka Shiozuka, Thomas A. Steitz, Nora Vázquez-Laslop, and Alexander S. Mankin. Negamycin Interferes with Decoding and Translocation by Simultaneous Interaction with rRNA and tRNA. *Molecular Cell*, 56(4):541–550, nov 2014.

[114] Marc A. Schureck, Jack A. Dunkle, Tatsuya Maehigashi, Stacey J. Miles, and Christine M. Dunham. Defining the mRNA recognition signature of a bacterial toxin protein. *Proceedings of the National Academy of Sciences*, 112(45):13862–13867, oct 2015.

[115] Nelson B. Olivier, Roger B. Altman, Jonas Noeske, Gregory S. Basarab, Erin Code, Andrew D. Ferguson, Ning Gao, Jian Huang, Manuel F. Juette, Stephania Livchak, Matthew D. Miller, D. Bryan Prince, Jamie H. D. Cate, Ed T. Buurman, and Scott C. Blanchard. Negamycin induces translational stalling and miscoding by binding to the small subunit head domain of the Escherichia coli ribosome. *Proceedings of the National Academy of Sciences*, 111(46):16274–16279, nov 2014.

[116] Michael R. Wasserman, Arto Pulk, Zhou Zhou, Roger B. Altman, John C. Zinder, Keith D. Green, Sylvie Garneau-Tsodikova, Jamie H. Doudna Cate, and Scott C. Blanchard. Chemically related 4,5-linked aminoglycoside antibiotics drive subunit rotation in opposite directions. *Nature Communications*, 6(1), jul 2015.

[117] Jinzhong Lin, Matthieu G. Gagnon, David Bulkley, and Thomas A. Steitz. Conformational Changes of Elongation Factor G on the Ribosome during tRNA Translocation. *Cell*, 160(1-2):219–227, jan 2015.

[118] Alexey Rozov, Natalia Demeshkina, Eric Westhof, Marat Yusupov, and Gulnara Yusupova. Structural insights into the translational infidelity mechanism. *Nature Communications*, 6(1), jun 2015.

[119] Alexey Amunts, Karol Fiedorczuk, Thao T. Truong, Josephine Chandler, E. Peter Greenberg, and V. Ramakrishnan. Bactobolin A Binds to a Site on the 70S Ribosome Distinct from Previously Seen Antibiotics. *Journal of Molecular Biology*, 427(4):753–755, feb 2015.

[120] Beatriz Llano-Sotelo, Jack Dunkle, Dorota Klepacki, Wen Zhang, Prabhavathi Fernandes, Jamie H. D. Cate, and Alexander S. Mankin. Binding and Action of CEM-101, a New Fluoroketolide Antibiotic That Inhibits Protein Synthesis. *Antimicrobial Agents and Chemotherapy*, 54(12):4961–4970, dec 2010.

[121] Timothy M. Colussi, David A. Costantino, Jianyu Zhu, John Paul Donohue, Andrei A. Korostelev, Zane A. Jaafar, Terra-Dawn M. Plank, Harry F. Noller, and Jeffrey S. Kieft. Initiation of translation in bacteria by a structured eukaryotic IRES RNA. *Nature*, 519(7541):110–113, feb 2015.

[122] Yury S Polikanov, Sergey V Melnikov, Dieter Söll, and Thomas A Steitz. Structural insights into the role of rRNA modifications in protein synthesis and ribosome assembly. *Nature Structural & Molecular Biology*, 22(4):342–344, mar 2015.

[123] Jonas Noeske, Michael R Wasserman, Daniel S Terry, Roger B Altman, Scott C Blanchard, and Jamie H D Cate. High-resolution structure of the Escherichia coli ribosome. *Nature Structural & Molecular Biology*, 22(4):336–341, mar 2015.

[124] Yury S. Polikanov, Agata L. Starosta, Manuel F. Juette, Roger B. Altman, Daniel S. Terry, Wanli Lu, Benjamin J. Burnett, George Dinos, Kevin A. Reynolds, Scott C. Blanchard, Thomas A. Steitz, and Daniel N. Wilson. Distinct tRNA Accommodation Intermediates Observed on the Ribosome with the Antibiotics Hygromycin A and A201A. *Molecular Cell*, 58(5):832–844, jun 2015.

[125] Raktim N Roy, Ivan B Lomakin, Matthieu G Gagnon, and Thomas A Steitz. The mechanism of inhibition of protein synthesis by the proline-rich peptide oncocin. *Nature Structural & Molecular Biology*, 22(6):466–469, may 2015.

[126] A Carolin Seefeldt, Fabian Nguyen, Stéphanie Antunes, Natacha Pérébaskine, Michael Graf, Stefan Arenz, K Kishore Inampudi, Céline Douat, Gilles Guichard, Daniel N Wilson, and C Axel Innis. The proline-rich antimicrobial peptide Onc112 inhibits translation by blocking and destabilizing the initiation complex. *Nature Structural & Molecular Biology*, 22(6):470–475, may 2015.

[127] Marc A. Schureck, Adrienne Repack, Stacey J. Miles, Jhomar Marquez, and Christine M. Dunham. Mechanism of endonuclease cleavage by the HigB toxin. *Nucleic Acids Research*, 44(16):7944–7953, jul 2016.

[128] Veerendra Kumar, Yun Chen, Rya Ero, Tofayel Ahmed, Jackie Tan, Zhe Li, Andrew See Weng Wong, Shashi Bhushan, and Yong-Gui Gao. Structure of BipA in GTP form bound to the ratcheted ribosome. *Proceedings of the National Academy of Sciences*, 112(35):10944–10949, aug 2015.

[129] Niels Fischer, Piotr Neumann, Andrey L. Konevega, Lars V. Bock, Ralf Ficner, Marina V. Rodnina, and Holger Stark. Structure of the E. coli ribosome–EF-Tu complex at <3 Å resolution by Cs-corrected cryo-EM. *Nature*, 520(7548):567–570, feb 2015.

[130] Elmar Behrmann, Justus Loerke, Tatyana V. Budkevich, Kaori Yamamoto, Andrea Schmidt, Pawel A. Penczek, Matthijn R. Vos, Jörg Bürger, Thorsten Mielke, Patrick Scheerer, and Christian M.T. Spahn. Structural Snapshots of Actively Translating Human Ribosomes. *Cell*, 161(4):845–857, may 2015.

[131] Basil J. Greber, Philipp Bieri, Marc Leibundgut, Alexander Leitner, Ruedi Aebersold, Daniel Boehringer, and Nenad Ban. The complete structure of the 55 S mammalian mitochondrial ribosome. *Science*, 348(6232):303–308, apr 2015.

[132] William E. Pierson, Eric D. Hoffer, Hannah E. Keedy, Carrie L. Simms, Christine M. Dunham, and Hani S. Zaher. Uniformity of Peptide Release Is Maintained by Methylation of Release Factors. *Cell Reports*, 17(1):11–18, sep 2016.

[133] Egor Svidritskiy and Andrei A. Korostelev. Ribosome Structure Reveals Preservation of Active Sites in the Presence of a P-Site Wobble Mismatch. *Structure*, 23(11):2155–2161, nov 2015.

[134] Sergey Melnikov, Justine Mailliot, Byung-Sik Shin, Lukas Rigger, Gulnara Yusupova, Ronald Micura, Thomas E. Dever, and Marat Yusupov. Crystal Structure of Hypusine-Containing Translation Factor eIF5A Bound to a Rotated Eukaryotic Ribosome. *Journal of Molecular Biology*, 428(18):3570–3576, sep 2016.

[135] Alexey Rozov, Natalia Demeshkina, Iskander Khusainov, Eric Westhof, Marat Yusupov, and Gulnara Yusupova. Novel base-pairing interactions at the tRNA wobble position crucial for accurate reading of the genetic code. *Nature Communications*, 7(1), jan 2016.

[136] A. Carolin Seefeldt, Michael Graf, Natacha Pérébaskine, Fabian Nguyen, Stefan Arenz, Mario Mardirossian, Marco Scocchi, Daniel N. Wilson, and C. Axel Innis. Structure of the mammalian antimicrobial peptide Bac7(1–16) bound within the exit tunnel of a bacterial ribosome. *Nucleic Acids Research*, 44(5):2429–2438, jan 2016.

[137] Justine Mailliot, Nicolas Garreau de Loubresse, Gulnara Yusupova, Arturas Meskauskas, Jonathan D. Dinman, and Marat Yusupov. Crystal Structures of the uL3 Mutant Ribosome: Illustration of the Importance of Ribosomal Proteins for Translation Efficiency. *Journal of Molecular Biology*, 428(10):2195–2202, may 2016.

[138] Chengying Ma, Daisuke Kurita, Ningning Li, Yan Chen, Hyouta Himeno, and Ning Gao. Mechanistic insights into the alternative translation termination by ArfA and RF2. *Nature*, 541(7638):550–553, dec 2017.

[139] Matthieu G. Gagnon, Raktim N. Roy, Ivan B. Lomakin, Tanja Florin, Alexander S. Mankin, and Thomas A. Steitz. Structures of proline-rich peptides bound to the ribosome reveal a common mechanism of protein synthesis inhibition. *Nucleic Acids Research*, 44(5):2439–2450, jan 2016.

[140] Irina V. Prokhorova, Kseniya A. Akulich, Desislava S. Makeeva, Ilya A. Osterman, Dmitry A. Skvortsov, Petr V. Sergiev, Olga A. Dontsova, Gulnara Yusupova, Marat M. Yusupov, and Sergey E. Dmitriev. Amicoumacin A induces cancer cell death by targeting the eukaryotic ribosome. *Scientific Reports*, 6(1), jun 2016.

[141] Alexey Rozov, Eric Westhof, Marat Yusupov, and Gulnara Yusupova. The ribosome prohibits the G•U wobble geometry at the first position of the codon–anticodon helix. *Nucleic Acids Research*, page gkw431, may 2016.

[142] Veerendra Kumar, Rya Ero, Tofayel Ahmed, Kwok Jian Goh, Yin Zhan, Shashi Bhushan, and Yong-Gui Gao. Structure of the GTP Form of Elongation Factor 4 (EF4) Bound to the Ribosome. *Journal of Biological Chemistry*, 291(25):12943–12950, jun 2016.

[143] Alan Brown, Israel S. Fernández, Yuliya Gordiyenko, and V. Ramakrishnan. Ribosome-dependent activation of stringent control. *Nature*, 534(7606):277–280, may 2016.

[144] Jason Murray, Christos G Savva, Byung-Sik Shin, Thomas E Dever, V Ramakrishnan, and Israel S Fernández. Structural characterization of ribosome recruitment and translocation by type IV IRES. *eLife*, 5, may 2016.

[145] Alexis I. Cocozaki, Roger B. Altman, Jian Huang, Ed T. Buurman, Steven L. Kazmirski, Peter Doig, D. Bryan Prince, Scott C. Blanchard, Jamie H. D. Cate, and Andrew D. Ferguson. Resistance mutations generate divergent antibiotic susceptibility profiles against translation inhibitors. *Proceedings of the National Academy of Sciences*, 113(29):8188–8193, jul 2016.

[146] Sergey V. Melnikov, Dieter Söll, Thomas A. Steitz, and Yury S. Polikanov. Insights into RNA binding by the anticancer drug cisplatin from the crystal structure of cisplatin-modified ribosome. *Nucleic Acids Research*, 44(10):4978–4987, apr 2016.

[147] Egor Svidritskiy, Rohini Madireddy, and Andrei A. Korostelev. Structural Basis for Translation Termination on a Pseudouridylated Stop Codon. *Journal of Molecular Biology*, 428(10):2228–2236, may 2016.

[148] Matthieu G. Gagnon, Jinzhong Lin, and Thomas A. Steitz. Elongation factor 4 remodels the A-site tRNA on the ribosome. *Proceedings of the National Academy of Sciences*, 113(18):4994–4999, apr 2016.

[149] Stefan Arenz, Lars V. Bock, Michael Graf, C. Axel Innis, Roland Beckmann, Helmut Grubmüller, Andrea C. Vaiana, and Daniel N. Wilson. A combined cryo-EM and molecular dynamics approach reveals the mechanism of ErmBL-mediated translation arrest. *Nature Communications*, 7(1), jul 2016.

[150] Priyanka D Abeyrathne, Cha San Koh, Timothy Grant, Nikolaus Grigorieff, and Andrei A Korostelev. Ensemble cryo-EM uncovers inchworm-like translocation of a viral IRES through the ribosome. *eLife*, 5, may 2016.

[151] Stefan Arenz, Manuel F. Juette, Michael Graf, Fabian Nguyen, Paul Huter, Yury S. Polikanov, Scott C. Blanchard, and Daniel N. Wilson. Structures of the orthosomycin antibiotics avilamycin and evernimicin in complex with the bacterial 70S ribosome. *Proceedings of the National Academy of Sciences*, 113(27):7527–7532, jun 2016.

[152] Anna B Loveland, Eugene Bah, Rohini Madireddy, Ying Zhang, Axel F Brilot, Nikolaus Grigorieff, and Andrei A Korostelev. Ribosome•RelA structures reveal the mechanism of stringent response activation. *eLife*, 5, jul 2016.

[153] Stefan Arenz, Maha Abdelshahid, Daniel Sohmen, Roshani Payoe, Agata L. Starosta, Otto Berninghausen, Vasili Hauryliuk, Roland Beckmann, and Daniel N. Wilson. The stringent factor RelA adopts an open conformation on the ribosome to stimulate ppGpp synthesis. *Nucleic Acids Research*, 44(13):6471–6481, may 2016.

[154] Iskander Khusainov, Quentin Vicens, Anthony Bochler, François Grosse, Alexander Myasnikov, Jean-François Ménétret, Johana Chicher, Stefano Marzi, Pascale Romby, Gulnara Yusupova, Marat Yusupov, and Yaser Hashem. Structure of the 70S ribosome from human pathogenStaphylococcus aureus. *Nucleic Acids Research*, page gkw933, oct 2016.

[155] Alexander G. Myasnikov, S. Kundhavai Natchiar, Marielle Nebout, Isabelle Hazemann, Véronique Imbert, Heena Khatter, Jean-François Peyron, and Bruno P. Klaholz. Structure–function insights reveal the human ribosome as a cancer target for antibiotics. *Nature Communications*, 7(1), sep 2016.

[156] Sergey Melnikov, Justine Mailliot, Lukas Rigger, Sandro Neuner, Byung-Sik Shin, Gulnara Yusupova, Thomas E Dever, Ronald Micura, and Marat Yusupov. Molecular insights into protein synthesis with proline residues. *EMBO reports*, 17(12):1776–1784, nov 2016.

[157] Niels Fischer, Piotr Neumann, Lars V. Bock, Cristina Maracci, Zhe Wang, Alena Paleskava, Andrey L. Konevega, Gunnar F Schröder, Helmut Grubmüller, Ralf Ficner, Marina V. Rodnina, and Holger Stark. The pathway to GTPase activation of elongation factor SelB on the ribosome. *Nature*, 540(7631):80–85, nov 2016.

[158] Sichen Shao, Jason Murray, Alan Brown, Jack Taunton, V. Ramakrishnan, and Ramanujan S. Hegde. Decoding Mammalian Ribosome-mRNA States by Translational GTPase Complexes. *Cell*, 167(5):1229–1240.e15, nov 2016.

[159] Tarek Hilal, Hiroshi Yamamoto, Justus Loerke, Jörg Bürger, Thorsten Mielke, and Christian M.T. Spahn. Structural insights into ribosomal rescue by Dom34 and Hbs1 at near-atomic resolution. *Nature Communications*, 7(1), dec 2016.

[160] Christian Schmidt, Eva Kowalinski, Vivekanandan Shanmuganathan, Quentin Defenouillère, Katharina Braunger, André Heuer, Markus Pech, Abdelkader Namane, Otto Berninghausen, Micheline Fromont-Racine, Alain Jacquier, Elena Conti, Thomas Becker, and Roland Beckmann. The cryo-EM structure of a ribosome–Ski2-Ski3-Ski8 helicase complex. *Science*, 354(6318):1431–1433, dec 2016.

[161] Nathan R. James, Alan Brown, Yuliya Gordiyenko, and V. Ramakrishnan. Translational termination without a stop codon. *Science*, 354(6318):1437–1440, dec 2016.

[162] Brandon McClary, Boris Zinshteyn, Mélanie Meyer, Morgan Jouanneau, Simone Pellegrino, Gulnara Yusupova, Anthony Schuller, Jeremy Chris P. Reyes, Junyan Lu, Zufeng Guo, Safiat Ayinde, Cheng Luo, Yongjun Dang, Daniel Romo, Marat Yusupov, Rachel Green, and Jun O. Liu. Inhibition of Eukaryotic Translation by the Antitumor Natural Product Agelastatin A. *Cell Chemical Biology*, 24(5):605–613.e5, may 2017.

[163] Paul Huter, Claudia Müller, Bertrand Beckert, Stefan Arenz, Otto Berninghausen, Roland Beckmann, and Daniel N. Wilson. Structural basis for ArfA–RF2-mediated translation termination on mRNAs lacking stop codons. *Nature*, 541(7638):546–549, dec 2017.

[164] Philipp Bieri, Marc Leibundgut, Martin Saurer, Daniel Boehringer, and Nenad Ban. The complete structure of the chloroplast 70S ribosome in complex with translation factor pY. *The EMBO Journal*, 36(4):475–486, dec 2017.

[165] Nirupa Desai, Alan Brown, Alexey Amunts, and V. Ramakrishnan. The structure of the yeast mitochondrial ribosome. *Science*, 355(6324):528–531, feb 2017.

[166] Linda E. Franken, Gert T. Oostergetel, Tjaard Pijning, Pranav Puri, Valentina Arkhipova, Egbert J. Boekema, Bert Poolman, and Albert Guskov. A general mechanism of ribosome dimerization revealed by single-particle cryo-electron microscopy. *Nature Communications*, 8(1), sep 2017.

[167] Iskander Khusainov, Quentin Vicens, Rustam Ayupov, Konstantin Usachev, Alexander Myasnikov, Angelita Simonetti, Shamil Validov, Bruno Kieffer, Gulnara Yusupova, Marat Yusupov, and Yaser Hashem. Structures and dynamics of hibernating ribosomes from Staphylococcus aureus mediated by intermolecular interactions of HPF. *The EMBO Journal*, 36(14):2073–2087, jun 2017.

[168] Irina Prokhorova, Roger B. Altman, Muminjon Djumagulov, Jaya P. Shrestha, Alexandre Urzhumtsev, Angelica Ferguson, Cheng-Wei Tom Chang, Marat Yusupov, Scott C. Blanchard, and Gulnara Yusupova. Aminoglycoside interactions and impacts on the eukaryotic ribosome. *Proceedings of the National Academy of Sciences*, 114(51), dec 2017.

[169] Donna Matzov, Shintaro Aibara, Arnab Basu, Ella Zimmerman, Anat Bashan, Mee-Ngan F. Yap, Alexey Amunts, and Ada E. Yonath. The cryo-EM structure of hibernating 100S ribosome dimer from pathogenic Staphylococcus aureus. *Nature Communications*, 8(1), sep 2017.

[170] Bertrand Beckert, Maha Abdelshahid, Heinrich Schäfer, Wieland Steinchen, Stefan Arenz, Otto Berninghausen, Roland Beckmann, Gert Bange, Kürşad Turgay, and Daniel N Wilson. Structure of the Bacillus subtilis hibernating 100S ribosome reveals the basis for 70S dimerization. *The EMBO Journal*, 36(14):2061–2072, may 2017.

[171] Xabier Agirrezabala, Ekaterina Samatova, Mariia Klimova, Miguel Zamora, David Gil-Carton, Marina V. Rodnina, and Mikel Valle. Ribosome rearrangements at the onset of translational bypassing. *Science Advances*, 3(6), jun 2017.

[172] Ting Su, Jingdong Cheng, Daniel Sohmen, Rickard Hedman, Otto Berninghausen, Gunnar von Heijne, Daniel N Wilson, and Roland Beckmann. The force-sensing peptide VemP employs extreme compaction and secondary structure formation to induce ribosomal stalling. *eLife*, 6, may 2017.

[173] Tanja Florin, Cristina Maracci, Michael Graf, Prajwal Karki, Dorota Klepacki, Otto Berninghausen, Roland Beckmann, Nora Vázquez-Laslop, Daniel N Wilson, Marina V Rodnina, and Alexander S Mankin. An antimicrobial peptide that inhibits translation by trapping release factors on the ribosome. *Nature Structural & Molecular Biology*, 24(9):752–757, jul 2017.

[174] Jendrik Hentschel, Chloe Burnside, Ingrid Mignot, Marc Leibundgut, Daniel Boehringer, and Nenad Ban. The Complete Structure of the Mycobacterium smegmatis 70S Ribosome. *Cell Reports*, 20(1):149–160, jul 2017.

[175] Simone Pellegrino, Mélanie Meyer, Christiane Zorbas, Soumaya A. Bouchta, Kritika Saraf, Stephen C. Pelly, Gulnara Yusupova, Antonio Evidente, Véronique Mathieu, Alexander Kornienko, Denis L.J. Lafontaine, and Marat Yusupov. The Amaryllidaceae Alkaloid Haemanthamine Binds the Eukaryotic Ribosome to Repress Cancer Cell Growth. *Structure*, 26(3):416–425.e4, mar 2018.

[176] Kevin Macé, Emmanuel Giudice, Sophie Chat, and Reynald Gillet. The structure of an elongation factor G-ribosome complex captured in the absence of inhibitors. *Nucleic Acids Research*, 46(6):3211–3217, feb 2018.

[177] Xing Zhang, Mason Lai, Winston Chang, Iris Yu, Ke Ding, Jan Mrazek, Hwee L. Ng, Otto O. Yang, Dmitri A. Maslov, and Z. Hong Zhou. Structures and stabilization of kinetoplastid-specific split rRNAs revealed by comparing leishmanial and human ribosomes. *Nature Communications*, 7(1), oct 2016.

[178] Matthew J. Belousoff, Zohar Eyal, Mazdak Radjainia, Tofayel Ahmed, Rebecca S. Bamert, Donna Matzov, Anat Bashan, Ella Zimmerman, Satabdi Mishra, David Cameron, Hans Elmlund, Anton Y. Peleg, Shashi Bhushan, Trevor Lithgow, and Ada Yonath. Structural Basis for Linezolid Binding Site Rearrangement in the Staphylococcus aureus Ribosome. *mBio*, 8(3), jul 2017.

[179] Zef A. Könst, Anne R. Szklarski, Simone Pellegrino, Sharon E. Michalak, M
'elanie Meyer, Camila Zanette, Regina Cencic, Sangkil Nam, Vamsee K. Voora, David A. Horne, Jerry Pelletier, David L. Mobley, Gulnara Yusupova, Marat Yusupov, and Christopher D. Vanderwal. Synthesis facilitates an understanding of the structural basis for translation inhibition by the lissoclimides. *Nature Chemistry*, 9(11):1140–1149, jul 2017.

[180] Fuxing Zeng, Yanbo Chen, Jonathan Remis, Mrinal Shekhar, James C. Phillips, Emad Tajkhorshid, and Hong Jin. Structural basis of co-translational quality control by ArfA and RF2 bound to ribosome. *Nature*, 541(7638):554–557, jan 2017.

[181] Gabriel Demo, Egor Svidritskiy, Rohini Madireddy, Ruben Diaz-Avalos, Timothy Grant, Nikolaus Grigorieff, Duncan Sousa, and Andrei A Korostelev. Mechanism of ribosome rescue by ArfA and RF2. *eLife*, 6, mar 2017.

[182] Yan Zhang, Samuel Hong, Ajchareeya Ruangprasert, Georgios Skiniotis, and Christine M. Dunham. Alternative Mode of E-Site tRNA Binding in the Presence of a Downstream mRNA Stem Loop at the Entrance Channel. *Structure*, 26(3):437–445.e3, mar 2018.

[183] Anna B. Loveland, Gabriel Demo, Nikolaus Grigorieff, and Andrei A. Korostelev. Ensemble cryo-EM elucidates the mechanism of translation fidelity. *Nature*, 546(7656):113–117, may 2017.

[184] Kailu Yang, Jeng-Yih Chang, Zhicheng Cui, Xiaojun Li, Ran Meng, Lijun Duan, Jirapat Thongchol, Joanita Jakana, Christoph M. Huwe, James C. Sacchettini, and Junjie Zhang. Structural insights into species-specific features of the ribosome from the human pathogen Mycobacterium tuberculosis. *Nucleic Acids Research*, 45(18):10884–10894, sep 2017.

[185] Ilya A. Osterman, Nelli F. Khabibullina, Ekaterina S. Komarova, Pavel Kasatsky, Victor G. Kartsev, Alexey A. Bogdanov, Olga A. Dontsova, Andrey L. Konevega, Petr V. Sergiev, and Yury S. Polikanov. Madumycin II inhibits peptide bond formation by forcing the peptidyl transferase center into an inactive state. *Nucleic Acids Research*, 45(12):7507–7514, may 2017.

[186] Samuel Hong, S. Sunita, Tatsuya Maehigashi, Eric D. Hoffer, Jack A. Dunkle, and Christine M. Dunham. Mechanism of tRNA-mediated +1 ribosomal frameshifting. *Proceedings of the National Academy of Sciences*, 115(44):11226–11231, sep 2018.

[187] Mikhail Metelev, Ilya A Osterman, Dmitry Ghilarov, Nelli F Khabibullina, Alexander Yakimov, Konstantin Shabalin, Irina Utkina, Dmitry Y Travin, Ekaterina S Komarova, Marina Serebryakova, Tatyana Artamonova, Mikhail Khodorkovskii, Andrey L Konevega, Petr V Sergiev, Konstantin Severinov, and Yury S Polikanov. Klebsazolicin inhibits 70S ribosome by obstructing the peptide exit tunnel. *Nature Chemical Biology*, 13(10):1129–1136, aug 2017.

[188] Marcus Fislage, Jingji Zhang, Zuben Patrick Brown, Chandra Sekhar Mandava, Suparna Sanyal, Måns Ehrenberg, and Joachim Frank. Cryo-EM shows stages of initial codon selection on the ribosome by aa-tRNA in ternary complex with GTP and the GTPase-deficient EF-TuH84A. *Nucleic Acids Research*, 46(11):5861–5874, may 2018.

[189] Mashal M. Almutairi, Maxim S. Svetlov, Douglas A. Hansen, Nelli F. Khabibullina, Dorota Klepacki, Han-Young Kang, David H. Sherman, Nora Vázquez-Laslop, Yury S. Polikanov, and Alexander S. Mankin. Co-produced natural ketolides methymycin and pikromycin inhibit bacterial growth by preventing synthesis of a limited number of proteins. *Nucleic Acids Research*, 45(16):9573–9582, jul 2017.

[190] Tofayel Ahmed, Jian Shi, and Shashi Bhushan. Unique localization of the plastid-specific ribosomal proteins in the chloroplast ribosome small subunit provides mechanistic insights into the chloroplastic translation. *Nucleic Acids Research*, 45(14):8581–8595, jun 2017.

[191] Satabdi Mishra, Tofayel Ahmed, Anu Tyagi, Jian Shi, and Shashi Bhushan. Structures of Mycobacterium smegmatis 70S ribosomes in complex with HPF, tmRNA, and P-tRNA. *Scientific Reports*, 8(1), sep 2018.

[192] Weixin Su, Veerendra Kumar, Yichen Ding, Rya Ero, Aida Serra, Benjamin Sian Teck Lee, Andrew See Weng Wong, Jian Shi, Siu Kwan Sze, Liang Yang, and Yong-Gui Gao. Ribosome protection by antibiotic resistance ATP-binding cassette protein. *Proceedings of the National Academy of Sciences*, 115(20):5157–5162, apr 2018.

[193] Egor Svidritskiy and Andrei A. Korostelev. Mechanism of Inhibition of Translation Termination by Blasticidin S. *Journal of Molecular Biology*, 430(5):591–593, mar 2018.

[194] Egor Svidritskiy and Andrei A. Korostelev. Conformational Control of Translation Termination on the 70S Ribosome. *Structure*, 26(6):821–828.e3, jun 2018.

[195] Anna B. Loveland and Andrei A. Korostelev. Structural dynamics of protein S1 on the 70S ribosome visualized by ensemble cryo-EM. *Methods*, 137:55–66, mar 2018.

[196] Eric D Hoffer, Tatsuya Maehigashi, Kurt Fredrick, and Christine M Dunham. Ribosomal ambiguity (ram) mutations promote the open (off) to closed (on) transition and thereby increase miscoding. *Nucleic Acids Research*, 47(3):1557–1563, nov 2019.

[197] Hossein Amiri and Harry F. Noller. Structural evidence for product stabilization by the ribosomal mRNA helicase. *RNA*, 25(3):364–375, dec 2019.

[198] Fuxing Zeng and Hong Jin. Conformation of methylated GGQ in the Peptidyl Transferase Center during Translation Termination. *Scientific Reports*, 8(1), feb 2018.

[199] Lucile Pantel, Tanja Florin, Malgorzata Dobosz-Bartoszek, Emilie Racine, Matthieu Sarciaux, Marine Serri, Jessica Houard, Jean-Marc Campagne, Renata Marcia de Figueiredo, Camille Midrier, Sophie Gaudriault, Alain Givaudan, Anne Lanois, Steve Forst, André Aumelas, Christelle Cotteaux-Lautard, Jean-Michel Bolla, Carina Vingsbo Lundberg, Douglas L. Huseby, Diarmaid Hughes, Philippe Villain-Guillot, Alexander S. Mankin, Yury S. Polikanov, and Maxime Gualtieri. Odilorhabdins, Antibacterial Agents that Cause Miscoding by Binding at a New Ribosomal Site. *Molecular Cell*, 70(1):83–94.e7, apr 2018.

[200] Andrey G. Tereshchenkov, Malgorzata Dobosz-Bartoszek, Ilya A. Osterman, James Marks, Vasilina A. Sergeeva, Pavel Kasatsky, Ekaterina S. Komarova, Andrey N. Stavrianidi, Igor A. Rodin, Andrey L. Konevega, Petr V. Sergiev, Natalia V. Sumbatyan, Alexander S. Mankin, Alexey A. Bogdanov, and Yury S. Polikanov. Binding and Action of Amino Acid Analogs of Chloramphenicol upon the Bacterial Ribosome. *Journal of Molecular Biology*, 430(6):842–852, mar 2018.

[201] Vera P Pisareva, Andrey V Pisarev, and Israel S Fernández. Dual tRNA mimicry in the Cricket Paralysis Virus IRES uncovers an unexpected similarity with the Hepatitis C Virus IRES. *eLife*, 7, jun 2018.

[202] Egor Svidritskiy, Gabriel Demo, and Andrei A. Korostelev. Mechanism of premature translation termination on a sense codon. *Journal of Biological Chemistry*, 293(32):12472–12479, aug 2018.

[203] Yunlong Li, Manjuli R. Sharma, Ravi K. Koripella, Yong Yang, Prem S. Kaushal, Qishan Lin, Joseph T. Wade, Todd A. Gray, Keith M. Derbyshire, Rajendra K. Agrawal, and Anil K. Ojha. Zinc depletion induces ribosome hibernation in mycobacteria. *Proceedings of the National Academy of Sciences*, 115(32):8191–8196, jul 2018.

[204] Paul Huter, Stefan Arenz, Lars V. Bock, Michael Graf, Jan Ole Frister, Andre Heuer, Lauri Peil, Agata L. Starosta, Ingo Wohlgemuth, Frank Peske, Jiř
’ı Nov
’aček, Otto Berninghausen, Helmut Grubmüller, Tanel Tenson, Roland Beckmann, Marina V. Rodnina, Andrea C. Vaiana, and Daniel N. Wilson. Structural Basis for Polyproline-Mediated Ribosome Stalling and Rescue by the Translation Elongation Factor EF-P. *Molecular Cell*, 68(3):515–527.e6, nov 2017.

[205] Annemarie Perez Boerema, Shintaro Aibara, Bijoya Paul, Victor Tobiasson, Dari Kimanius, Björn O. Forsberg, Karin Wallden, Erik Lindahl, and A. Amunts. Structure of the chloroplast ribosome with chl-RRF and hibernation-promoting factor. *Nature Plants*, 4(4):212–217, apr 2018.

[206] Mario Mardirossian, Natacha P
’er
’ebaskine, Monica Benincasa, Stefano Gambato, Sven Hofmann, Paul Huter, Claudia Müller, Kai Hilpert, C. Axel Innis, Alessandro Tossi, and Daniel N. Wilson. The Dolphin Proline-Rich Antimicrobial Peptide Tur1A Inhibits Protein Synthesis by Targeting the Bacterial Ribosome. *Cell Chemical Biology*, 25(5):530–539.e7, may 2018.

[207] Eva Kummer, Marc Leibundgut, Oliver Rackham, Richard G. Lee, Daniel Boehringer, Aleksandra Filipovska, and Nenad Ban. Unique features of mammalian mitochondrial translation initiation revealed by cryo-EM. *Nature*, 560(7717):263–267, aug 2018.

[208] Simone Pellegrino, Natalia Demeshkina, Eder Mancera-Martinez, Sergey Melnikov, Angelita Simonetti, Alexander Myasnikov, Marat Yusupov, Gulnara Yusupova, and Yaser Hashem. Structural Insights into the Role of Diphthamide on Elongation Factor 2 in mRNA Reading-Frame Maintenance. *Journal of Molecular Biology*, 430(17):2677–2687, aug 2018.

[209] Alexey Rozov, Philippe Wolff, Henri Grosjean, Marat Yusupov, Gulnara Yusupova, and Eric Westhof. Tautomeric G•U pairs within the molecular ribosomal grip and fidelity of decoding in bacteria. *Nucleic Acids Research*, 46(14):7425–7435, jun 2018.

[210] Michael Graf, Paul Huter, Cristina Maracci, Miroslav Peterek, Marina V. Rodnina, and Daniel N. Wilson. Visualization of translation termination intermediates trapped by the Apidaecin 137 peptide during RF3-mediated recycling of RF1. *Nature Communications*, 9(1), aug 2018.

[211] Julia Flis, Mikael Holm, Emily J. Rundlet, Justus Loerke, Tarek Hilal, Marylena Dabrowski, Jörg Bürger, Thorsten Mielke, Scott C. Blanchard, Christian M.T. Spahn, and Tatyana V. Budkevich. tRNA Translocation by the Eukaryotic 80S Ribosome and the Impact of GTP Hydrolysis. *Cell Reports*, 25(10):2676–2688.e7, dec 2018.

[212] Rasmus Kock Flygaard, Niels Boegholm, Marat Yusupov, and Lasse B. Jenner. Cryo-EM structure of the hibernating Thermus thermophilus 100S ribosome reveals a protein-mediated dimerization mechanism. *Nature Communications*, 9(1), oct 2018.

[213] Bertrand Beckert, Martin Turk, Andreas Czech, Otto Berninghausen, Roland Beckmann, Zoya Ignatova, Jürgen M. Plitzko, and Daniel N. Wilson. Structure of a hibernating 100S ribosome reveals an inactive conformation of the ribosomal protein S1. *Nature Microbiology*, 3(10):1115–1121, sep 2018.

[214] Caillan Crowe-McAuliffe, Michael Graf, Paul Huter, Hiraku Takada, Maha Abdelshahid, Jiří Nováček, Victoriia Murina, Gemma C. Atkinson, Vasili Hauryliuk, and Daniel N. Wilson. Structural basis for antibiotic resistance mediated by the Bacillus subtilis ABCF ATPase VmlR. *Proceedings of the National Academy of Sciences*, 115(36):8978–8983, aug 2018.

[215] Szymon Juszkiewicz, Viswanathan Chandrasekaran, Zhewang Lin, Sebastian Kraatz, V. Ramakrishnan, and Ramanujan S. Hegde. ZNF598 Is a Quality Control Sensor of Collided Ribosomes. *Molecular Cell*, 72(3):469–481.e7, nov 2018.

[216] Simone Pellegrino, M
'elanie Meyer, Zef A Könst, Mikael Holm, Vamsee K Voora, Daniya Kashinskaya, Camila Zanette, David L Mobley, Gulnara Yusupova, Chris D Vanderwal, Scott C Blanchard, and Marat Yusupov. Understanding the role of intermolecular interactions between lissoclimides and the eukaryotic ribosome. *Nucleic Acids Research*, 47(6):3223–3232, feb 2019.

[217] Patrick Pausch, Maha Abdelshahid, Wieland Steinchen, Heinrich Schäfer, Fabio Lino Gratani, Sven-Andreas Freibert, Christiane Wolz, Kürşad Turgay, Daniel N. Wilson, and Gert Bange. Structural Basis for Regulation of the Opposing (p)ppGpp Synthetase and Hydrolase within the Stringent Response Orchestrator Rel. *Cell Reports*, 32(11):108157, sep 2020.

[218] Ken Ikeuchi, Petr Tesina, Yoshitaka Matsuo, Takato Sugiyama, Jingdong Cheng, Yasushi Saeki, Keiji Tanaka, Thomas Becker, Roland Beckmann, and Toshifumi Inada. Collided ribosomes form a unique structural interface to induce Hel2-driven quality control pathways. *The EMBO Journal*, 38(5), jan 2019.

[219] Silva Lilleorg, Kaspar Reier, Arto Pulk, Aivar Liiv, Triin Tammsalu, Lauri Peil, Jamie H.D. Cate, and Jaanus Remme. Bacterial ribosome heterogeneity: Changes in ribosomal protein composition during transition into stationary growth phase. *Biochimie*, 156:169–180, jan 2018.

[220] Takeshi Yokoyama, Kodai Machida, Wakana Iwasaki, Tomoaki Shigeta, Madoka Nishimoto, Mari Takahashi, Ayako Sakamoto, Mayumi Yonemochi, Yoshie Harada, Hideki Shigematsu, Mikako Shirouzu, Hisashi Tadakuma, Hiroaki Imataka, and Takuhiro Ito. HCV IRES Captures an Actively Translating 80S Ribosome. *Molecular Cell*, 74(6):1205–1214.e8, jun 2019.

[221] Ling Zhang, Ying-Hui Wang, Xing Zhang, Laura Lancaster, Jie Zhou, and Harry F. Noller. The structural basis for inhibition of ribosomal translocation by viomycin. *Proceedings of the National Academy of Sciences*, 117(19):10271–10277, apr 2020.

[222] Alan Brown, Matthew R Baird, Matthew CJ Yip, Jason Murray, and Sichen Shao. Structures of translationally inactive mammalian ribosomes. *eLife*, 7, oct 2018.

[223] Jie Zhou, Laura Lancaster, John Paul Donohue, and Harry F. Noller. Spontaneous ribosomal translocation of mRNA and tRNAs into a chimeric hybrid state. *Proceedings of the National Academy of Sciences*, 116(16):7813–7818, apr 2019.

[224] Sergey V Melnikov, Nelli F Khabibullina, Elisabeth Mairhofer, Oscar Vargas-Rodriguez, Noah M Reynolds, Ronald Micura, Dieter Söll, and Yury S Polikanov. Mechanistic insights into the slow peptide bond formation with D-amino acids in the ribosomal active site. *Nucleic Acids Research*, 47(4):2089–2100, dec 2019.

[225] Maxim S. Svetlov, Elena Plessa, Chih-Wei Chen, Anthony Bougas, Marios G. Krokidis, George P. Dinos, and Yury S. Polikanov. High-resolution crystal structures of ribosome-bound chloramphenicol and erythromycin provide the ultimate basis for their competition. *RNA*, 25(5):600–606, feb 2019.

[226] Ha An Nguyen, Eric D. Hoffer, and Christine M. Dunham. Importance of a tRNA anticodon loop modification and a conserved, noncanonical anticodon stem pairing in tRNACGGPro for decoding. *Journal of Biological Chemistry*, 294(14):5281–5291, apr 2019.

[227] Eric D Hoffer, Samuel Hong, S Sunita, Tatsuya Maehigashi, Ruben L Gonzalez, Paul C Whitford, and Christine M Dunham. Structural insights into mRNA reading frame regulation by tRNA modification and slippery codon–anticodon pairing. *eLife*, 9, oct 2020.

[228] Ravi K. Koripella, Manjuli R. Sharma, Paul Risteff, Pooja Keshavan, and Rajendra K. Agrawal. Structural insights into unique features of the human mitochondrial ribosome recycling. *Proceedings of the National Academy of Sciences*, 116(17):8283–8288, apr 2019.

[229] Eileen L. Murphy, Kavindra V. Singh, Bryant Avila, Torsten Kleffmann, Steven T. Gregory, Barbara E. Murray, Kurt L. Krause, Reza Khayat, and Gerwald Jogl. Cryo-electron microscopy structure of the 70S ribosome from Enterococcus faecalis. *Scientific Reports*, 10(1), oct 2020.

[230] Takahiko Matsushita, Girish C. Sati, Nuwan Kondasinghe, Michael G. Pirrone, Takayuki Kato, Prabuddha Waduge, Harshitha Santhosh Kumar, Adrian Cortes Sanchon, Malgorzata Dobosz-Bartoszek, Dimitri Shcherbakov, Mario Juhas, Sven N. Hobbie, Thomas Schrepfer, Christine S. Chow, Yury S. Polikanov, Jochen Schacht, Andrea Vasella, Erik C. Böttger, and David Crich. Design, Multigram Synthesis, and in Vitro and in Vivo Evaluation of Propylamycin: A Semisynthetic 4,5-Deoxystreptamine Class Aminoglycoside for the Treatment of Drug-Resistant Enterobacteriaceae and Other Gram-Negative Pathogens. *Journal of the American Chemical Society*, 141(12):5051–5061, feb 2019.

[231] Sandip Kaledhonkar, Ziao Fu, Kelvin Caban, Wen Li, Bo Chen, Ming Sun, Ruben L. Gonzalez, and Joachim Frank. Late steps in bacterial translation initiation visualized using time-resolved cryo-EM. *Nature*, 570(7761):400–404, may 2019.

[232] Nelli F. Khabibullina, Andrey G. Tereshchenkov, Ekaterina S. Komarova, Egor A. Syroegin, Dmitrii I. Shiriaev, Alena Paleskava, Victor G. Kartsev, Alexey A. Bogdanov, Andrey L. Konevega, Olga A. Dontsova, Petr V. Sergiev, Ilya A. Osterman, and Yury S. Polikanov. Structure of Dirithromycin Bound to the Bacterial Ribosome Suggests New Ways for Rational Improvement of Macrolides. *Antimicrobial Agents and Chemotherapy*, 63(6), jun 2019.

[233] Ha An Nguyen, S. Sunita, and Christine M. Dunham. Disruption of evolutionarily correlated tRNA elements impairs accurate decoding. *Proceedings of the National Academy of Sciences*, 117(28):16333–16338, jun 2020.

[234] Egor Svidritskiy, Gabriel Demo, Anna B Loveland, Chen Xu, and Andrei A Korostelev. Extensive ribosome and RF2 rearrangements during translation termination. *eLife*, 8, sep 2019.

[235] Wenfei Li, Fred R. Ward, Kim F. McClure, Stacey Tsai-Lan Chang, Elizabeth Montabana, Spiros Liras, Robert G. Dullea, and Jamie H. D. Cate. Structural basis for selective stalling of human ribosome nascent chain complexes by a drug-like molecule. *Nature Structural & Molecular Biology*, 26(6):501–509, jun 2019.

[236] Zachary D. Aron, Atousa Mehrani, Eric D. Hoffer, Kristie L. Connolly, Pooja Srinivas, Matthew C. Torhan, John N. Alumasa, Mynthia Cabrera, Divya Hosangadi, Jay S. Barbor, Steven C. Cardinale, Steven M. Kwasny, Lucas R. Morin, Michelle M. Butler, Timothy J. Opperman, Terry L. Bowlin, Ann Jerse, Scott M. Stagg, Christine M. Dunham, and Kenneth C. Keiler. trans-Translation inhibitors bind to a novel site on the ribosome and clear Neisseria gonorrhoeae in vivo. *Nature Communications*, 12(1), mar 2021.

[237] Ziao Fu, Gabriele Indrisiunaite, Sandip Kaledhonkar, Binita Shah, Ming Sun, Bo Chen, Robert A. Grassucci, Måns Ehrenberg, and Joachim Frank. The structural basis for release-factor activation during translation termination revealed by time-resolved cryogenic electron microscopy. *Nature Communications*, 10(1), jun 2019.

[238] Ian J Pavelich, Tatsuya Maehigashi, Eric D Hoffer, Ajchareeya Ruangprasert, Stacey J Miles, and Christine M Dunham. Monomeric YoeB toxin retains RNase activity but adopts an obligate dimeric form for thermal stability. *Nucleic Acids Research*, 47(19):10400–10413, sep 2019.

[239] Francisco Acosta-Reyes, Ritam Neupane, Joachim Frank, and Israel S Fernández. The Israeli acute paralysis virus IRES captures host ribosomes by mimicking a ribosomal state with hybrid tRNAs. *The EMBO Journal*, 38(21), oct 2019.

[240] Petr Tesina, Elisabeth Heckel, Jingdong Cheng, Micheline Fromont-Racine, Robert Buschauer, Lukas Kater, Birgitta Beatrix, Otto Berninghausen, Alain Jacquier, Thomas Becker, and Roland Beckmann. Structure of the 80S ribosome–Xrn1 nuclease complex. *Nature Structural & Molecular Biology*, 26(4):275–280, mar 2019.

[241] Christopher D. Rae, Yuliya Gordiyenko, and V. Ramakrishnan. How a circularized tmRNA moves through the ribosome. *Science*, 363(6428):740–744, feb 2019.

[242] Alexey Rozov, Iskander Khusainov, Kamel El Omari, Ramona Duman, Vitaliy Mykhaylyk, Marat Yusupov, Eric Westhof, Armin Wagner, and Gulnara Yusupova. Importance of potassium ions for ribosome structure and function revealed by long-wavelength X-ray diffraction. *Nature Communications*, 10(1), jun 2019.

[243] S. Kundhavai Natchiar, Alexander G. Myasnikov, Hanna Kratzat, Isabelle Hazemann, and Bruno P. Klaholz. Visualization of chemical modifications in the human 80S ribosome structure. *Nature*, 551(7681):472–477, nov 2017.

[244] Vivekanandan Shanmuganathan, Nina Schiller, Anastasia Magoulopoulou, Jingdong Cheng, Katharina Braunger, Florian Cymer, Otto Berninghausen, Birgitta Beatrix, Kenji Kohno, Gunnar von Heijne, and Roland Beckmann. Structural and mutational analysis of the ribosome-arresting human XBP1u. *eLife*, 8, jun 2019.

[245] Jonas Barandun, Mirjam Hunziker, Charles R. Vossbrinck, and Sebastian Klinge. Evolutionary compaction and adaptation visualized by the structure of the dormant microsporidian ribosome. *Nature Microbiology*, 4(11):1798–1804, jul 2019.

[246] Yehuda Halfon, Donna Matzov, Zohar Eyal, Anat Bashan, Ella Zimmerman, Jette Kjeldgaard, Hanne Ingmer, and Ada Yonath. Exit tunnel modulation as resistance mechanism of S. aureus erythromycin resistant mutant. *Scientific Reports*, 9(1), aug 2019.

[247] Villu Kasari, Agnieszka A Pochopien, Tõnu Margus, Victoriia Murina, Kathryn Turnbull, Yang Zhou, Tracy Nissan, Michael Graf, Jiří Nováček, Gemma C Atkinson, Marcus J O Johansson, Daniel N Wilson, and Vasili Hauryliuk. A role for the Saccharomyces cerevisiae ABCF protein New1 in translation termination/recycling. *Nucleic Acids Research*, 47(16):8807–8820, jul 2019.

[248] Viswanathan Chandrasekaran, Szymon Juszkiewicz, Junhong Choi, Joseph D. Puglisi, Alan Brown, Sichen Shao, V. Ramakrishnan, and Ramanujan S. Hegde. Mechanism of ribosome stalling during translation of a poly(A) tail. *Nature Structural & Molecular Biology*, 26(12):1132–1140, nov 2019.

[249] Aldema Sas-Chen, Justin M. Thomas, Donna Matzov, Masato Taoka, Kellie D. Nance, Ronit Nir, Keri M. Bryson, Ran Shachar, Geraldy L. S. Liman, Brett W. Burkhart, Supuni Thalalla Gamage, Yuko Nobe, Chloe A. Briney, Michaella J. Levy, Ryan T. Fuchs, G. Brett Robb, Jesse Hartmann, Sunny Sharma, Qishan Lin, Laurence Florens, Michael P. Washburn, Toshiaki Isobe, Thomas J. Santangelo, Moran Shalev-Benami, Jordan L. Meier, and Schraga Schwartz. Dynamic RNA acetylation revealed by quantitative cross-evolutionary mapping. *Nature*, 583(7817):638–643, jun 2020.

[250] Yoshitaka Matsuo, Petr Tesina, Shizuka Nakajima, Masato Mizuno, Akinori Endo, Robert Buschauer, Jingdong Cheng, Okuto Shounai, Ken Ikeuchi, Yasushi Saeki, Thomas Becker, Roland Beckmann, and Toshifumi Inada. RQT complex dissociates ribosomes collided on endogenous RQC substrate SDD1. *Nature Structural & Molecular Biology*, 27(4):323–332, mar 2020.

[251] Yehuda Halfon, Alicia Jimenez-Fernandez, Ruggero La Rosa, Rocio Espinosa Portero, Helle Krogh Johansen, Donna Matzov, Zohar Eyal, Anat Bashan, Ella Zimmerman, Matthew Belousoff, Søren Molin, and Ada Yonath. Structure of Pseudomonas aeruginosa ribosomes from an aminoglycoside-resistant clinical isolate. *Proceedings of the National Academy of Sciences*, 116(44):22275–22281, oct 2019.

[252] Naomi Shimokawa-Chiba, Claudia Müller, Keigo Fujiwara, Bertrand Beckert, Koreaki Ito, Daniel N. Wilson, and Shinobu Chiba. Release factor-dependent ribosome rescue by BrfA in the Gram-positive bacterium Bacillus subtilis. *Nature Communications*, 10(1), nov 2019.

[253] Petr Tesina, Laura N Lessen, Robert Buschauer, Jingdong Cheng, Colin Chih-Chien Wu, Otto Berninghausen, Allen R Buskirk, Thomas Becker, Roland Beckmann, and Rachel Green. Molecular mechanism of translational stalling by inhibitory codon combinations and poly(A) tracts. *The EMBO Journal*, 39(3), dec 2020.

[254] Robert Buschauer, Yoshitaka Matsuo, Takato Sugiyama, Ying-Hsin Chen, Najwa Alhusaini, Thomas Sweet, Ken Ikeuchi, Jingdong Cheng, Yasuko Matsuki, Risa Nobuta, Andrea Gilmozzi, Otto Berninghausen, Petr Tesina, Thomas Becker, Jeff Coller, Toshifumi Inada, and Roland Beckmann. The Ccr4-Not complex monitors the translating ribosome for codon optimality. *Science*, 368(6488), apr 2020.

[255] Alba Herrero del Valle, Britta Seip, Iñaki Cervera-Marzal, Gu
'enaël Sacheau, A. Carolin Seefeldt, and C. Axel Innis. Ornithine capture by a translating ribosome controls bacterial polyamine synthesis. *Nature Microbiology*, 5(4):554–561, feb 2020.

[256] Dejian Zhou, Takehito Tanzawa, Jinzhong Lin, and Matthieu G. Gagnon. Structural basis for ribosome recycling by RRF and tRNA. *Nature Structural & Molecular Biology*, 27(1):25–32, dec 2020.

[257] Daniel E. Eyler, Monika K. Franco, Zahra Batool, Monica Z. Wu, Michelle L. Dubuke, Malgorzata Dobosz-Bartoszek, Joshua D. Jones, Yury S. Polikanov, Bijoyita Roy, and Kristin S. Koutmou. Pseudouridinylation of mRNA coding sequences alters translation. *Proceedings of the National Academy of Sciences*, 116(46):23068–23074, oct 2019.

[258] Bridget Y. Huang and Israel S. Fernández. Long-range interdomain communications in eIF5B regulate GTP hydrolysis and translation initiation. *Proceedings of the National Academy of Sciences*, 117(3):1429–1437, jan 2020.

[259] Christopher E. Morgan, Wei Huang, Susan D. Rudin, Derek J. Taylor, James E. Kirby, Robert A. Bonomo, and Edward W. Yu. Cryo-electron Microscopy Structure of the Acinetobacter baumannii 70S Ribosome and Implications for New Antibiotic Development. *mBio*, 11(1), feb 2020.

[260] Ravi Kiran Koripella, Manjuli R. Sharma, Kalpana Bhargava, Partha P. Datta, Prem S. Kaushal, Pooja Keshavan, Linda L. Spremulli, Nilesh K. Banavali, and Rajendra K. Agrawal. Structures of the human mitochondrial ribosome bound to EF-G1 reveal distinct features of mitochondrial translation elongation. *Nature Communications*, 11(1), jul 2020.

[261] Chengyuan Wang, Vadim Molodtsov, Emre Firlar, Jason T. Kaelber, Gregor Blaha, Min Su, and Richard H. Ebright. Structural basis of transcription-translation coupling. *Science*, 369(6509):1359–1365, aug 2020.

[262] Chen Bao, Sarah Loerch, Clarence Ling, Andrei A Korostelev, Nikolaus Grigorieff, and Dmitri N Ermolenko. mRNA stem-loops can pause the ribosome by hindering A-site tRNA binding. *eLife*, 9, may 2020.

[263] Anna B. Loveland, Gabriel Demo, and Andrei A. Korostelev. Cryo-EM of elongating ribosome with EF-Tu•GTP elucidates tRNA proofreading. *Nature*, 584(7822):640–645, jul 2020.

[264] Shijie Huang, Nikolay A. Aleksashin, Anna B. Loveland, Dorota Klepacki, Kaspar Reier, Amira Kefi, Teresa Szal, Jaanus Remme, Luc Jaeger, Nora Vázquez-Laslop, Andrei A. Korostelev, and Alexander S. Mankin. Ribosome engineering reveals the importance of 5S rRNA autonomy for ribosome assembly. *Nature Communications*, 11(1), jun 2020.

[265] Wenfei Li, Stacey Tsai-Lan Chang, Fred. R. Ward, and Jamie H. D. Cate. Selective inhibition of human translation termination by a drug-like compound. *Nature Communications*, 11(1), oct 2020.

[266] Maxim S. Svetlov, Egor A. Syroegin, Elena V. Aleksandrova, Gemma C. Atkinson, Steven T. Gregory, Alexander S. Mankin, and Yury S. Polikanov. Structure of Erm-modified 70S ribosome reveals the mechanism of macrolide resistance. *Nature Chemical Biology*, 17(4):412–420, jan 2021.

[267] Ye Zhou, Panagiotis L. Kastritis, Shannon E. Dougherty, Jonathan Bouvette, Allen L. Hsu, Laura Burbaum, Shyamal Mosalaganti, Stefan Pfeffer, Wim J. H. Hagen, Friedrich Förster, Mario J. Borgnia, Christine Vogel, Martin Beck, Alberto Bartesaghi, and Gustavo M. Silva. Structural impact of K63 ubiquitin on yeast translocating ribosomes under oxidative stress. *Proceedings of the National Academy of Sciences*, 117(36):22157–22166, aug 2020.

[268] Zahra Batool, Ivan B. Lomakin, Yury S. Polikanov, and Christopher G. Bunick. Sarecycline interferes with tRNA accommodation and tethers mRNA to the 70S ribosome. *Proceedings of the National Academy of Sciences*, 117(34):20530–20537, aug 2020.

[269] Tayah Hopes, Karl Norris, Michaela Agapiou, Charley G P McCarthy, Philip A Lewis, Mary J O'Connell, Juan Fontana, and Julie L Aspden. Ribosome heterogeneity in Drosophila melanogaster gonads through paralog-switching. *Nucleic Acids Research*, 50(4):2240–2257, jul 2021.

[270] Florent Waltz, Heddy Soufari, Anthony Bochler, Philippe Giegé, and Yaser Hashem. Cryo-EM structure of the RNA-rich plant mitochondrial ribosome. *Nature Plants*, 6(4):377–383, apr 2020.

[271] Evgeny B. Pichkur, Alena Paleskava, Andrey G. Tereshchenkov, Pavel Kasatsky, Ekaterina S. Komarova, Dmitrii I. Shiriaev, Alexey A. Bogdanov, Olga A. Dontsova, Ilya A. Osterman, Petr V. Sergiev, Yury S. Polikanov, Alexander G. Myasnikov, and Andrey L. Konevega. Insights into the improved macrolide inhibitory activity from the high-resolution cryo-EM structure of dirithromycin bound to the E. coli 70S ribosome. *RNA*, 26(6):715–723, mar 2020.

[272] Varun Bhaskar, Alexandra Graff-Meyer, Andreas D. Schenk, Simone Cavadini, Ottilie von Loeffelholz, S. Kundhavai Natchiar, Caroline G. Artus-Revel, Hans-Rudolf Hotz, Gabriel Bretones, Bruno P. Klaholz, and Jeffrey A. Chao. Dynamics of uS19 C-Terminal Tail during the Translation Elongation Cycle in Human Ribosomes. *Cell Reports*, 31(1):107473, apr 2020.

[273] Ilya A. Osterman, Maximiliane Wieland, Tinashe P. Maviza, Kseniya A. Lashkevich, Dmitrii A. Lukianov, Ekaterina S. Komarova, Yuliya V. Zakalyukina, Robert Buschauer, Dmitrii I. Shiriaev, Semen A. Leyn, Jaime E. Zlamal, Mikhail V. Biryukov, Dmitry A. Skvortsov, Vadim N. Tashlitsky, Vladimir I. Polshakov, Jingdong Cheng, Yury S. Polikanov, Alexey A. Bogdanov, Andrei L. Osterman, Sergey E. Dmitriev, Roland Beckmann, Olga A. Dontsova, Daniel N. Wilson, and Petr V. Sergiev. Tetracenomycin X inhibits translation by binding within the ribosomal exit tunnel. *Nature Chemical Biology*, 16(10):1071–1077, jun 2020.

[274] Eva Kummer and Nenad Ban. Structural insights into mammalian mitochondrial translation elongation catalyzed by mt EFG 1. *The EMBO Journal*, 39(15), jun 2020.

[275] Alexander Golubev, Bulat Fatkhullin, Iskander Khusainov, Lasse Jenner, Azat Gabdulkhakov, Shamil Validov, Gulnara Yusupova, Marat Yusupov, and Konstantin Usachev. Cryo-EM structure of the ribosome functional complex of the human pathogen Staphylococcus aureus at 3.2 Å resolution. *FEBS Letters*, 594(21):3551–3567, sep 2020.

[276] Kai-Hsin Chan, Valentyn Petrychenko, Claudia Mueller, Cristina Maracci, Wolf Holtkamp, Daniel N. Wilson, Niels Fischer, and Marina V. Rodnina. Mechanism of ribosome rescue by alternative ribosome-rescue factor B. *Nature Communications*, 11(1), aug 2020.

[277] Yuzuru Itoh, Andreas Naschberger, Narges Mortezaei, Johannes M. Herrmann, and Alexey Amunts. Analysis of translating mitoribosome reveals functional characteristics of translation in mitochondria of fungi. *Nature Communications*, 11(1), oct 2020.

[278] Victor Tobiasson and Alexey Amunts. Ciliate mitoribosome illuminates evolutionary steps of mitochondrial translation. *eLife*, 9, jun 2020.

[279] Jennifer N. Wells, Robert Buschauer, Timur Mackens-Kiani, Katharina Best, Hanna Kratzat, Otto Berninghausen, Thomas Becker, Wendy Gilbert, Jingdong Cheng, and Roland Beckmann. Structure and function of yeast Lso2 and human CCDC124 bound to hibernating ribosomes. *PLOS Biology*, 18(7):e3000780, jul 2020.

[280] Yuzuru Itoh, Juni Andréll, Austin Choi, Uwe Richter, Priyanka Maiti, Robert B. Best, Antoni Barrientos, Brendan J. Battersby, and Alexey Amunts. Mechanism of membrane-tethered mitochondrial protein synthesis. *Science*, 371(6531):846–849, feb 2021.

[281] Matthias Thoms, Robert Buschauer, Michael Ameismeier, Lennart Koepke, Timo Denk, Maximilian Hirschenberger, Hanna Kratzat, Manuel Hayn, Timur Mackens-Kiani, Jingdong Cheng, Jan H. Straub, Christina M. Stürzel, Thomas Fröhlich, Otto Berninghausen, Thomas Becker, Frank Kirchhoff, Konstantin M. J. Sparrer, and Roland Beckmann. Structural basis for translational shutdown and immune evasion by the Nsp1 protein of SARS-CoV-2. *Science*, 369(6508):1249–1255, sep 2020.

[282] Shintaro Aibara, Vivek Singh, Angelika Modelska, and Alexey Amunts. Structural basis of mitochondrial translation. *eLife*, 9, aug 2020.

[283] Michael William Webster, Maria Takacs, Chengjin Zhu, Vita Vidmar, Ayesha Eduljee, Mo'men Abdelkareem, and Albert Weixlbaumer. Structural basis of transcription-translation coupling and collision in bacteria. *Science*, 369(6509):1355–1359, aug 2020.

[284] Kai Ehrenbolger, Nathan Jespersen, Himanshu Sharma, Yuliya Y. Sokolova, Yuri S. Tokarev, Charles R. Vossbrinck, and Jonas Barandun. Differences in structure and hibernation mechanism highlight diversification of the microsporidian ribosome. *PLOS Biology*, 18(10):e3000958, oct 2020.

[285] Irina S. Abaeva, Quentin Vicens, Anthony Bochler, Heddy Soufari, Angelita Simonetti, Tatyana V. Pestova, Yaser Hashem, and Christopher U.T. Hellen. The Halastavi árva Virus Intergenic Region IRES Promotes Translation by the Simplest Possible Initiation Mechanism. *Cell Reports*, 33(10):108476, dec 2020.

[286] Nirupa Desai, Hanting Yang, Viswanathan Chandrasekaran, Razina Kazi, Michal Minczuk, and V. Ramakrishnan. Elongational stalling activates mitoribosome-associated quality control. *Science*, 370(6520):1105–1110, nov 2020.

[287] Charlotte Guyomar, Gaetano D'Urso, Sophie Chat, Emmanuel Giudice, and Reynald Gillet. Structures of tmRNA and SmpB as they transit through the ribosome. *Nature Communications*, 12(1), aug 2021.

[288] Tomoya Fujita, Takeshi Yokoyama, Mikako Shirouzu, Hideki Taguchi, Takuhiro Ito, and Shintaro Iwasaki. The landscape of translational stall sites in bacteria revealed by monosome and disome profiling. *RNA*, 28(3):290–302, dec 2022.

[289] Shirin Akbar, Sayan Bhakta, and Jayati Sengupta. Structural insights into the interplay of protein biogenesis factors with the 70S ribosome. *Structure*, 29(7):755–767.e4, jul 2021.

[290] Vikash Jha, Bappaditya Roy, Dushyant Jahagirdar, Zakkary A McNutt, Elan A Shatoff, Bethany L Boleratz, Dean E Watkins, Ralf Bundschuh, Kaustuv Basu, Joaquin Ortega, and Kurt Fredrick. Structural basis of sequestration of the anti-Shine-Dalgarno sequence in the Bacteroidetes ribosome. *Nucleic Acids Research*, 49(1):547–567, dec 2021.

[291] Mario Mardirossian, Riccardo Sola, Bertrand Beckert, Erica Valencic, Dominic W. P. Collis, Jure Borišek, Federica Armas, Adriana Di Stasi, Jan Buchmann, Egor A. Syroegin, Yury S. Polikanov, Alessandra Magistrato, Kai Hilpert, Daniel N. Wilson, and Marco Scocchi. Peptide Inhibitors of Bacterial Protein Synthesis with Broad Spectrum and SbmA-Independent Bactericidal Activity against Clinical Pathogens. *Journal of Medicinal Chemistry*, 63(17):9590–9602, jul 2020.

[292] Christine E. Carbone, Gabriel Demo, Rohini Madireddy, Egor Svidritskiy, and Andrei A. Korostelev. ArfB can displace mRNA to rescue stalled ribosomes. *Nature Communications*, 11(1), nov 2020.

[293] Zoe L Watson, Fred R Ward, Raphaël M
'eheust, Omer Ad, Alanna Schepartz, Jillian F Banfield, and Jamie HD Cate. Structure of the bacterial ribosome at 2 Å resolution. *eLife*, 9, sep 2020.

[294] Gabriel Demo, Howard B. Gamper, Anna B. Loveland, Isao Masuda, Christine E. Carbone, Egor Svidritskiy, Ya-Ming Hou, and Andrei A. Korostelev. Structural basis for +1 ribosomal frameshifting during EF-G-catalyzed translocation. *Nature Communications*, 12(1), jul 2021.

[295] Ravi Kiran Koripella, Ayush Deep, Ekansh K. Agrawal, Pooja Keshavan, Nilesh K. Banavali, and Rajendra K. Agrawal. Distinct mechanisms of the human mitoribosome recycling and antibiotic resistance. *Nature Communications*, 12(1), jun 2021.

[296] Tolou Golkar, Angelia V. Bassenden, Krishnagopal Maiti, Dev P. Arya, T. Martin Schmeing, and Albert M. Berghuis. Structural basis for plazomicin antibiotic action and resistance. *Communications Biology*, 4(1), jun 2021.

[297] Patrick R. Smith, Sarah Loerch, Nikesh Kunder, Alexander D. Stanowick, Tzu-Fang Lou, and Zachary T. Campbell. Functionally distinct roles for eEF2K in the control of ribosome availability and p-body abundance. *Nature Communications*, 12(1), nov 2021.

[298] Zhemin Zhang, Christopher E. Morgan, Robert A. Bonomo, and Edward W. Yu. Cryo-EM Determination of Eravacycline-Bound Structures of the Ribosome and the Multidrug Efflux Pump AdeJ of Acinetobacter baumannii. *mBio*, 12(3), jun 2021.

[299] Chih-Wei Chen, Julia A. Pavlova, Dmitrii A. Lukianov, Andrey G. Tereshchenkov, Gennady I. Makarov, Zimfira Z. Khairullina, Vadim N. Tashlitsky, Alena Paleskava, Andrey L. Konevega, Alexey A. Bogdanov, Ilya A. Osterman, Natalia V. Sumbatyan, and Yury S. Polikanov. Binding and Action of Triphenylphosphonium Analog of Chloramphenicol upon the Bacterial Ribosome. *Antibiotics*, 10(4):390, apr 2021.

[300] Yumi Koga, Eileen M. Hoang, Yongho Park, Alexander F.A. Keszei, Jason Murray, Sichen Shao, and Brian B. Liau. Discovery of C13-Aminobenzoyl Cycloheximide Derivatives that Potently Inhibit Translation Elongation. *Journal of the American Chemical Society*, 143(34):13473–13477, aug 2021.

[301] Yu Zhao, Jay Rai, Hongguo Yu, and Hong Li. CryoEM structures of pseudouridine-free ribosome suggest impacts of chemical modifications on ribosome conformations. *Structure*, 30(7):983–992.e5, jul 2022.

[302] Zhicheng Cui, Xiaojun Li, Joonyoung Shin, Howard Gamper, Ya-Ming Hou, James C. Sacchettini, and Junjie Zhang. Interplay between an ATP-binding cassette F protein and the ribosome from Mycobacterium tuberculosis. *Nature Communications*, 13(1), jan 2022.

[303] Emily J. Rundlet, Mikael Holm, Magdalena Schacherl, S. Kundhavai Natchiar, Roger B. Altman, Christian M. T. Spahn, Alexander G. Myasnikov, and Scott C. Blanchard. Structural basis of early translocation events on the ribosome. *Nature*, 595(7869):741–745, jul 2021.

[304] Moise Mansour, Emmanuel Giudice, Xibing Xu, Hatice Akarsu, Patricia Bordes, Valérie Guillet, Donna-Joe Bigot, Nawel Slama, Gaetano D'urso, Sophie Chat, Peter Redder, Laurent Falquet, Lionel Mourey, Reynald Gillet, and Pierre Genevaux. Substrate recognition and cryo-EM structure of the ribosome-bound TAC toxin of Mycobacterium tuberculosis. *Nature Communications*, 13(1), may 2022.

[305] Caillan Crowe-McAuliffe, Victoriia Murina, Kathryn Jane Turnbull, Marje Kasari, Merianne Mohamad, Christine Polte, Hiraku Takada, Karolis Vaitkevicius, Jörgen Johansson, Zoya Ignatova, Gemma C. Atkinson, Alex J. O'Neill, Vasili Hauryliuk, and Daniel N. Wilson. Structural basis of ABCF-mediated resistance to pleuromutilin, lincosamide, and streptogramin A antibiotics in Gram-positive pathogens. *Nature Communications*, 12(1), jun 2021.

[306] Eva Kummer, Katharina Noel Schubert, Tanja Schoenhut, Alain Scaiola, and Nenad Ban. Structural basis of translation termination, rescue, and recycling in mammalian mitochondria. *Molecular Cell*, 81(12):2566–2582.e6, jun 2021.

[307] Agnieszka A. Pochopien, Bertrand Beckert, Sergo Kasvandik, Otto Berninghausen, Roland Beckmann, Tanel Tenson, and Daniel N. Wilson. Structure of Gcn1 bound to stalled and colliding 80S ribosomes. *Proceedings of the National Academy of Sciences*, 118(14), mar 2021.

[308] Bertrand Beckert, Elodie C. Leroy, Shanmugapriya Sothiselvam, Lars V. Bock, Maxim S. Svetlov, Michael Graf, Stefan Arenz, Maha Abdelshahid, Britta Seip, Helmut Grubmüller, Alexander S. Mankin, C. Axel Innis, Nora V
'azquez-Laslop, and Daniel N. Wilson. Structural and mechanistic basis for translation inhibition by macrolide and ketolide antibiotics. *Nature Communications*, 12(1), jul 2021.

[309] Kyle T Powers, Flint Stevenson-Jones, Sathish K N Yadav, Beate Amthor, Joshua C Bufton, Ufuk Borucu, Dakang Shen, Jonas P Becker, Daria Lavysh, Matthias W Hentze, Andreas E Kulozik, Gabriele Neu-Yilik, and Christiane Schaffitzel. Blasticidin S inhibits mammalian translation and enhances production of protein encoded by nonsense mRNA. *Nucleic Acids Research*, 49(13):7665–7679, jun 2021.

[310] Xabier Agirrezabala, Ekaterina Samatova, Meline Macher, Marija Liutkute, Manisankar Maiti, David Gil-Carton, Jiri Novacek, Mikel Valle, and Marina V Rodnina. A switch from α-helical to β-strand conformation during co-translational protein folding. *The EMBO Journal*, 41(4), jan 2022.

[311] Anne-Xander van der Stel, Emily R. Gordon, Arnab Sengupta, Allyson K. Martínez, Dorota Klepacki, Thomas N. Perry, Alba Herrero del Valle, Nora Vázquez-Laslop, Matthew S. Sachs, Luis R. Cruz-Vera, and C. Axel Innis. Structural basis for the tryptophan sensitivity of TnaC-mediated ribosome stalling. *Nature Communications*, 12(1), sep 2021.

[312] Laura Czech, Christopher-Nils Mais, Hanna Kratzat, Pinku Sarmah, Pietro Giammarinaro, Sven-Andreas Freibert, Hanna Folke Esser, Joanna Musial, Otto Berninghausen, Wieland Steinchen, Roland Beckmann, Hans-Georg Koch, and Gert Bange. Inhibition of SRP-dependent protein secretion by the bacterial alarmone (p)ppGpp. *Nature Communications*, 13(1), feb 2022.

[313] Pramod R. Bhatt, Alain Scaiola, Gary Loughran, Marc Leibundgut, Annika Kratzel, Romane Meurs, René Dreos, Kate M. O'Connor, Angus McMillan, Jeffrey W. Bode, Volker Thiel, David Gatfield, John F. Atkins, and Nenad Ban. Structural basis of ribosomal frameshifting during translation of the SARS-CoV-2 RNA genome. *Science*, 372(6548):1306–1313, jun 2021.

[314] Ting Su, Renuka Kudva, Thomas Becker, Robert Buschauer, Tobias Komar, Otto Berninghausen, Gunnar von Heijne, Jingdong Cheng, and Roland Beckmann. Structural basis of l-tryptophan-dependent inhibition of release factor 2 by the TnaC arrest peptide. *Nucleic Acids Research*, 49(16):9539–9547, aug 2021.

[315] Miglė Kišonaitė, Klemens Wild, Karine Lapouge, Thomas Ruppert, and Irmgard Sinning. High-resolution structures of a thermophilic eukaryotic 80S ribosome reveal atomistic details of translocation. *Nature Communications*, 13(1), jan 2022.

[316] Muminjon Djumagulov, Natalia Demeshkina, Lasse Jenner, Alexey Rozov, Marat Yusupov, and Gulnara Yusupova. Accuracy mechanism of eukaryotic ribosome translocation. *Nature*, 600(7889):543–546, dec 2021.

[317] Maximiliane Wieland, Mikael Holm, Emily J. Rundlet, Martino Morici, Timm O. Koller, Tinashe P. Maviza, Domen Pogorevc, Ilya A. Osterman, Rolf Müller, Scott C. Blanchard, and Daniel N. Wilson. The cyclic octapeptide antibiotic argyrin B inhibits translation by trapping EF-G on the ribosome during translocation. *Proceedings of the National Academy of Sciences*, 119(19), may 2022.

[318] Merianne Mohamad, David Nicholson, Chayan Kumar Saha, Vasili Hauryliuk, Thomas A Edwards, Gemma C Atkinson, Neil A Ranson, and Alex J O'Neill. Sal-type ABC-F proteins: intrinsic and common mediators of pleuromutilin resistance by target protection in staphylococci. *Nucleic Acids Research*, 50(4):2128–2142, feb 2022.

[319] Liang Xue, Swantje Lenz, Maria Zimmermann-Kogadeeva, Dimitry Tegunov, Patrick Cramer, Peer Bork, Juri Rappsilber, and Julia Mahamid. Visualizing translation dynamics at atomic detail inside a bacterial cell. *Nature*, 610(7930):205–211, sep 2022.

[320] Caillan Crowe-McAuliffe, Victoriia Murina, Kathryn Jane Turnbull, Susanne Huch, Marje Kasari, Hiraku Takada, Lilit Nersisyan, Arnfinn Sundsfjord, Kristin Hegstad, Gemma C. Atkinson, Vicent Pelechano, Daniel N. Wilson, and Vasili Hauryliuk. Structural basis for PoxtA-mediated resistance to phenicol and oxazolidinone antibiotics. *Nature Communications*, 13(1), apr 2022.

[321] Valentyn Petrychenko, Bee-Zen Peng, Ana C. de A. P. Schwarzer, Frank Peske, Marina V. Rodnina, and Niels Fischer. Structural mechanism of GTPase-powered ribosome-tRNA movement. *Nature Communications*, 12(1), oct 2021.

[322] Disha-Gajanan Hiregange, Andre Rivalta, Tanaya Bose, Elinor Breiner-Goldstein, Sarit Samiya, Giuseppe Cimicata, Liudmila Kulakova, Ella Zimmerman, Anat Bashan, Osnat Herzberg, and Ada Yonath. Cryo-EM structure of the ancient eukaryotic ribosome from the human parasite Giardia lamblia. *Nucleic Acids Research*, 50(3):1770–1782, jan 2022.

[323] Yury Zgadzay, Olga Kolosova, Artem Stetsenko, Cheng Wu, David Bruchlen, Konstantin Usachev, Shamil Validov, Lasse Jenner, Andrey Rogachev, Gulnara Yusupova, Matthew S. Sachs, Albert Guskov, and Marat Yusupov. E-site drug specificity of the human pathogen Candida albicans ribosome. *Science Advances*, 8(21), may 2022.

[324] Kazuki Saito, Hanna Kratzat, Annabelle Campbell, Robert Buschauer, A. Maxwell Burroughs, Otto Berninghausen, L. Aravind, Rachel Green, Roland Beckmann, and Allen R. Buskirk. Ribosome collisions induce mRNA cleavage and ribosome rescue in bacteria. *Nature*, 603(7901):503–508, mar 2022.

[325] Federico Cerullo, Sebastian Filbeck, Pratik Rajendra Patil, Hao-Chih Hung, Haifei Xu, Julia Vornberger, Florian W. Hofer, Jaro Schmitt, Guenter Kramer, Bernd Bukau, Kay Hofmann, Stefan Pfeffer, and Claudio A. P. Joazeiro. Bacterial ribosome collision sensing by a MutS DNA repair ATPase paralogue. *Nature*, 603(7901):509–514, mar 2022.

[326] Lunda Shen, Zhaoming Su, Kailu Yang, Cheng Wu, Thomas Becker, Deborah Bell-Pedersen, Junjie Zhang, and Matthew S. Sachs. Structure of the translating Neurospora ribosome arrested by cycloheximide. *Proceedings of the National Academy of Sciences*, 118(48), nov 2021.

[327] Matthew J. Mitcheltree, Amarnath Pisipati, Egor A. Syroegin, Katherine J. Silvestre, Dorota Klepacki, Jeremy D. Mason, Daniel W. Terwilliger, Giambattista Testolin, Aditya R. Pote, Kelvin J. Y. Wu, Richard Porter Ladley, Kelly Chatman, Alexander S. Mankin, Yury S. Polikanov, and Andrew G. Myers. A synthetic antibiotic class overcoming bacterial multidrug resistance. *Nature*, 599(7885):507–512, oct 2021.

[328] Egor A. Syroegin, Laurin Flemmich, Dorota Klepacki, Nora Vazquez-Laslop, Ronald Micura, and Yury S. Polikanov. Structural basis for the context-specific action of the classic peptidyl transferase inhibitor chloramphenicol. *Nature Structural & Molecular Biology*, 29(2):152–161, feb 2022.

[329] Fuxing Zeng, Xin Li, Melissa Pires-Alves, Xin Chen, Christopher W. Hawk, and Hong Jin. Conserved heterodimeric GTPase Rbg1/Tma46 promotes efficient translation in eukaryotic cells. *Cell Reports*, 37(4):109877, oct 2021.

[330] Christopher E. Morgan, Zhemin Zhang, Robert A. Bonomo, and Edward W. Yu. An Analysis of the Novel Fluorocycline TP-6076 Bound to Both the Ribosome and Multidrug Efflux Pump AdeJ from Acinetobacter baumannii. *mBio*, 13(1), feb 2022.

[331] Kaitlyn Tsai, Vanja Stojković, D. John Lee, Iris D. Young, Teresa Szal, Dorota Klepacki, Nora Vázquez-Laslop, Alexander S. Mankin, James S. Fraser, and Danica Galonić Fujimori. Structural basis for context-specific inhibition of translation by oxazolidinone antibiotics. *Nature Structural & Molecular Biology*, 29(2):162–171, feb 2022.

[332] Yannan Tian, Fuxing Zeng, Adrika Raybarman, Shirin Fatma, Amy Carruthers, Qingrong Li, and Raven H. Huang. Sequential rescue and repair of stalled and damaged ribosome by bacterial PrfH and RtcB. *Proceedings of the National Academy of Sciences*, 119(29), jul 2022.

[333] Christine E. Carbone, Anna B. Loveland, Howard B. Gamper, Ya-Ming Hou, Gabriel Demo, and Andrei A. Korostelev. Time-resolved cryo-EM visualizes ribosomal translocation with EF-G and GTP. *Nature Communications*, 12(1), dec 2021.

[334] Anna B. Loveland, Egor Svidritskiy, Denis Susorov, Soojin Lee, Alexander Park, Sarah Zvornicanin, Gabriel Demo, Fen-Biao Gao, and Andrei A. Korostelev. Ribosome inhibition by C9ORF72-ALS/FTD-associated poly-PR and poly-GR proteins revealed by cryo-EM. *Nature Communications*, 13(1), may 2022.

[335] Egor A Syroegin, Elena V Aleksandrova, and Yury S Polikanov. Structural basis for the inability of chloramphenicol to inhibit peptide bond formation in the presence of A-site glycine. *Nucleic Acids Research*, 50(13):7669–7679, jun 2022.

[336] Daniel Arango, David Sturgill, Renbin Yang, Tapan Kanai, Paulina Bauer, Jyoti Roy, Ziqiu Wang, Masaki Hosogane, Sarah Schiffers, and Shalini Oberdoerffer. Direct epitranscriptomic regulation of mammalian translation initiation through N4-acetylcytidine. *Molecular Cell*, 82(15):2797–2814.e11, aug 2022.

[337] Tarek Hilal, Benjamin Y. Killam, Milica Grozdanovic, Malgorzata Dobosz-Bartoszek, Justus Loerke, Jorg Burger, Thorsten Mielke, Paul R. Copeland, Miljan Simonovic, and Christian M. T. Spahn. Structure of the mammalian ribosome as it decodes the selenocysteine UGA codon. *Science*, 376(6599):1338–1343, jun 2022.

[338] Sihan Li, Ken Ikeuchi, Misaki Kato, Robert Buschauer, Takato Sugiyama, Shungo Adachi, Hideo Kusano, Tohru Natsume, Otto Berninghausen, Yoshitaka Matsuo, Thomas Becker, Roland Beckmann, and Toshifumi Inada. Sensing of individual stalled 80S ribosomes by Fap1 for nonfunctional rRNA turnover. *Molecular Cell*, 82(18):3424–3437.e8, sep 2022.
