# Supplementary material for: Ratchet, swivel, tilt and roll: a complete description of subunit rotation in the ribosome
Source: Nucleic Acids Res. 2022 Dec 30;51(2):919–34. doi: 10.1093/nar/gkac1211 (PMC9881166; doi:10.1093/nar/gkac1211)
Supplement: gkac1211_Supplemental_Files [file gkac1211_supplemental_files.zip › AppendixB.pdf]

Appendix B: Ribosome rotation angles calculated with the RAD method: SSU-only models

Below are tables that provide all 334 calculated sets of subunit orientations, obtained from 325 RCSB accession codes.
For each entry, the organism name, reference, method and resolution were extracted directly from the RCSB database query system according the following protocol:

-*Organism names*: From the query system, each entry has a list of `polymer_entities`. The longest nucleic acid chain was determined through the `entity_poly.rcsb_entity_polymer_type` and `entity_poly.rcsb_sample_sequence_length` values of each polymer in the RCSB entry. In the tables below, the organism name corresponds to the `rcsb_entity_source_organism.[0].ncbi_scientific_name` value for the longest nucleic acid chain. While many ribosome structures have molecular elements from multiple organisms, this protocol was applied, so that the organism name corresponds to that of the SSU rRNA.

-*Experimental details*: For each entry in the RCSB database, the method listed is from the `exptl.[0].method` element, while the resolution listed in the table is `rcsb_entry_info.resolution_combined.[0]`.

-*Mitoribosomes*: Since there is not a specific entry that indicates whether a ribosome is a mitoribosome, the following process was used to identify mitoribosomes: If the `struct.title` element of the RCSB entry contained “mitoribo” or “mitochond” (case-insensitive), then a * is included in the tables to indicate the structure is a mitoribosome.

-*References*: References are given in the following format: Last name of first author (year) *Journal*
The listed citations are obtained from the RCSB query system by checking the array of references given under the `citation` element for the entry. The value of `rcsb_is_primary` was checked for each citation listed. If Yes was found, the reference was used for the table. For space considerations, only one author is listed for each reference. The author listed in each table entry corresponds to the `rcsb_authors.[0]` element. If the journal or year is not available in the RCSB system, it is omitted from the table.

Abbreviations: In the table, the following acronyms are used for journal names:

- AAC: Antimicrobial Agents and Chemotherapy
- ACDBC: Acta Crystallogr D Biol Crystallogr
- ACIEE: Angew Chem Int Ed Engl
- JMB: Journal of Molecular Biology
- NAR: Nucleic Acid Research
- NCB: Nature Chemical Biology
- NComm: Nature Communications
- NMb: Nature Microbiology
- NSMB: Nature Structural and Molecular Biology
- PNAS: Proceedings of the National Academy of Sciences USA

Notes: The RADtool -download option was unable to automatically calculate angles for the following RCSB entries: 1JGO, 1JGP, 1JGQ. The RAD method was unable to automatically identify the SSU rRNA in these structures, since they only contain P atoms. However, the method was used by manually providing the chain IDs, and all angles were calculated. If the tilt angle is 0.0, the tilt direction is listed as ND (not defined). Entries 4V8M, 5T2A, 6HRM and 6ZJ3 contain complete ribosomes, but the RAD method was unable to successfully perform structure alignment for the LSU rRNA. Accordingly, only head angles could be calculated, and those values are included in this file.

Table 1 of 7

PDB		HEAD				PRUNED		RMSD		EXP DETAILS		ORGANISM		REFERENCE
ID	chain	ϕ_{head}	θ_{head}	ψ_{head}	$\lvert\Delta\vec{x}_{\text{head}}\rvert$	body	head	body	head	method	res.	name	mito	
1FJG	A	1.6	4.0	-34.4	0.185	819	407	1.03	0.86	XRAY	3.0	T. thermophilus		Carter, et al. (2000) *Nature*[1]
1FKA	A	2.7	3.7	-16.0	0.715	457	171	1.42	1.35	XRAY	3.3	T. thermophilus		Schluenzen, et al. (2000) *Cell*[2]
1HNW	A	1.7	3.5	-23.5	0.398	827	409	1.02	0.87	XRAY	3.4	T. thermophilus		Brodersen, et al. (2000) *Cell*[3]
1HNX	A	1.8	3.5	-23.5	0.410	825	410	1.02	0.90	XRAY	3.4	T. thermophilus		Brodersen, et al. (2000) *Cell*[3]
1HNZ	A	1.9	3.6	-24.1	0.614	829	408	1.00	0.88	XRAY	3.3	T. thermophilus		Brodersen, et al. (2000) *Cell*[3]
1HR0	A	1.4	3.5	-24.1	0.462	809	409	0.99	0.86	XRAY	3.2	T. thermophilus		Carter, et al. (2001) *Science*[4]
1I94	A	3.7	3.6	-8.5	0.446	804	393	1.07	0.93	XRAY	3.2	T. thermophilus		Pioletti, et al. (2001) *EMBO J*[5]
1I95	A	3.7	3.7	-10.0	0.443	792	392	1.12	0.96	XRAY	4.5	T. thermophilus		Pioletti, et al. (2001) *EMBO J*[5]
1I96	A	3.7	3.7	-8.6	0.401	802	396	1.09	0.94	XRAY	4.2	T. thermophilus		Pioletti, et al. (2001) *EMBO J*[5]
1I97	A	3.7	3.7	-10.2	0.539	777	395	1.11	1.04	XRAY	4.5	T. thermophilus		Pioletti, et al. (2001) *EMBO J*[5]
1IBK	A	1.3	3.7	-30.1	0.316	826	407	0.99	0.89	XRAY	3.3	T. thermophilus		Ogle, et al. (2001) *Science*[6]
1IBL	A	0.3	3.3	-11.3	0.227	822	413	1.04	0.88	XRAY	3.1	T. thermophilus		Ogle, et al. (2001) *Science*[6]
1IBM	A	1.6	3.5	-19.5	0.294	828	410	1.01	0.91	XRAY	3.3	T. thermophilus		Ogle, et al. (2001) *Science*[6]
1J5E	A	1.7	3.8	-25.5	0.387	821	407	1.00	0.91	XRAY	3.0	T. thermophilus		Wimberly, et al. (2000) *Nature*[7]
1JGO	A	1.1	0.3	-116.2	0.746	597	212	1.39	1.35	XRAY	5.6	T. thermophilus		Yusupova, et al. (2001) *Cell*[8]
1JGP	A	1.1	0.3	-116.2	0.746	597	212	1.39	1.35	XRAY	7.0	T. thermophilus		Yusupova, et al. (2001) *Cell*[8]
1JGQ	A	1.1	0.3	-116.2	0.746	597	212	1.39	1.35	XRAY	5.0	T. thermophilus		Yusupova, et al. (2001) *Cell*[8]
1N32	A	0.4	3.2	-10.0	0.351	823	414	1.04	0.89	XRAY	3.0	T. thermophilus		Ogle, et al. (2002) *Cell*[9]
1N33	A	1.0	3.5	-8.0	0.179	824	411	1.04	0.96	XRAY	3.4	T. thermophilus		Ogle, et al. (2002) *Cell*[9]
1N34	A	2.8	4.0	9.0	0.492	825	376	1.02	1.03	XRAY	3.8	T. thermophilus		Ogle, et al. (2002) *Cell*[9]
1N36	A	3.6	4.2	16.7	0.278	831	366	1.05	1.04	XRAY	3.6	T. thermophilus		Ogle, et al. (2002) *Cell*[9]
1XMO	A	1.4	3.6	-17.3	0.457	803	406	1.02	0.91	XRAY	3.2	T. thermophilus HB27		Murphy, et al. (2004) *NSMB*[10]
1XMQ	A	1.0	3.6	-7.0	0.397	804	410	1.03	0.92	XRAY	3.0	T. thermophilus HB8		Murphy, et al. (2004) *NSMB*[10]
1XNQ	A	1.4	3.7	-17.1	0.400	811	408	1.01	0.91	XRAY	3.0	T. thermophilus		Murphy, et al. (2004) *NSMB*[11]
1XNR	A	1.2	3.3	-17.1	0.323	815	409	1.00	0.91	XRAY	3.1	T. thermophilus		Murphy, et al. (2004) *NSMB*[11]
2E5L	A	4.4	4.6	-11.5	0.297	832	402	1.02	0.95	XRAY	3.3	T. thermophilus		Kaminishi, et al. (2007) *Structure*[12]
2F4V	A	1.5	3.8	-20.6	0.153	815	413	0.99	0.99	XRAY	3.8	T. thermophilus		Murray, et al. (2006) *Chem Biol*[13]
2HHH	A	4.4	4.6	-11.8	0.536	834	400	1.00	0.91	XRAY	3.4	T. thermophilus HB8		Schluenzen, et al. (2006) *NSMB*[14]
2UU9	A	0.9	3.7	-6.0	0.368	826	416	1.04	0.91	XRAY	3.1	T. thermophilus HB8		Weixlbaumer, et al. (2007) *NSMB*[15]
2UUA	A	1.3	3.6	-4.7	0.229	828	417	1.01	0.94	XRAY	2.9	T. thermophilus HB8		Weixlbaumer, et al. (2007) *NSMB*[15]
2UUB	A	0.8	3.5	3.4	0.251	824	412	1.05	0.88	XRAY	2.8	T. thermophilus HB8		Weixlbaumer, et al. (2007) *NSMB*[15]
2UUC	A	1.1	3.7	-0.7	0.333	825	413	1.03	0.91	XRAY	3.1	T. thermophilus HB8		Weixlbaumer, et al. (2007) *NSMB*[15]
2UXB	A	1.9	3.8	4.2	0.266	829	408	0.99	0.94	XRAY	3.1	T. thermophilus HB8		Dunham, et al. (2007) *RNA*[16]
2UXC	A	1.5	3.7	-2.0	0.294	824	414	1.01	0.92	XRAY	2.9	T. thermophilus HB8		Dunham, et al. (2007) *RNA*[16]
2UXD	A	1.7	3.9	7.9	0.166	807	407	0.99	0.95	XRAY	3.2	T. thermophilus HB8		Dunham, et al. (2007) *RNA*[16]
2VQE	A	1.5	3.6	-12.5	0.332	833	413	0.99	0.91	XRAY	2.5	T. thermophilus HB8		Kurata, et al. (2008) *J Biol Chem*[17]
2VQF	A	1.6	3.7	-13.5	0.349	830	410	0.98	0.92	XRAY	2.9	T. thermophilus HB8		Kurata, et al. (2008) *J Biol Chem*[17]
2YKR	A	6.9	10.4	-19.8	2.006	227	126	1.46	1.40	EM	9.8	E. coli DH5[alpha]		Guo, et al. (2011) *PNAS*[18]
2ZM6	A	3.8	4.5	-13.0	0.104	827	400	1.02	0.95	XRAY	3.3	T. thermophilus		Kaminishi, et al. *To be published*
3J28	N	11.9	1.9	-6.2	2.128	326	81	1.38	1.39	EM	12.9	E. coli K-12		Guo, et al. (2013) *NAR*[19]
3J29	N	15.4	23.8	-42.9	3.936	319	88	1.44	1.35	EM	14.0	E. coli K-12		Guo, et al. (2013) *NAR*[19]
3J2A	N	6.8	12.8	-1.9	5.673	343	101	1.45	1.40	EM	13.1	E. coli K-12		Guo, et al. (2013) *NAR*[19]
3J2B	N	10.7	15.2	-47.9	3.309	266	126	1.39	1.40	EM	13.6	E. coli K-12		Guo, et al. (2013) *NAR*[19]
3J2D	N	9.7	6.4	-74.4	6.542	153	84	1.41	1.41	EM	18.7	E. coli		Guo, et al. (2013) *NAR*[19]
3J2E	N	13.5	9.2	2.2	3.709	298	131	1.45	1.37	EM	15.3	E. coli		Guo, et al. (2013) *NAR*[19]
3J2F	N	12.5	11.0	-27.2	3.746	302	124	1.39	1.32	EM	17.6	E. coli		Guo, et al. (2013) *NAR*[19]
3J2G	N	14.1	16.5	-16.0	7.150	216	83	1.38	1.43	EM	16.5	E. coli		Guo, et al. (2013) *NAR*[19]
3J2H	N	20.2	5.9	136.7	4.641	197	88	1.48	1.41	EM	18.8	E. coli		Guo, et al. (2013) *NAR*[19]
3J7A	A	4.8	2.6	114.2	0.652	619	139	1.16	1.21	EM	3.2	Plasmodium falciparum 3D7		Wong, et al. (2014) *Elife*[20]
3J80	2	10.7	5.6	46.3	0.328	577	251	1.27	1.19	EM	3.8	Kluyveromyces lactis		Hussain, et al. (2014) *Cell*[21]
3J81	2	-0.2	0.9	-56.5	0.848	574	291	1.27	1.23	EM	4.0	Kluyveromyces lactis		Hussain, et al. (2014) *Cell*[21]
3JAM	2	8.6	4.8	48.2	0.996	605	264	1.20	1.15	EM	3.5	Kluyveromyces lactis		Llacer, et al. (2015) *Mol Cell*[22]

PDB		HEAD				PRUNED		RMSD		EXP DETAILS		ORGANISM		REFERENCE
ID	chain	ϕ_{head}	θ_{head}	ψ_{head}	$\lvert\Delta\vec{x}_{\text{head}}\rvert$	body	head	body	head	method	res.	name	mito	
3JAP	2	-0.1	1.1	-66.6	0.500	566	276	1.29	1.25	EM	4.9	Kluyveromyces lactis		Llacer, et al. (2015) *Mol Cell*[22]
3JAQ	2	5.7	11.9	-7.7	1.561	565	254	1.33	1.25	EM	6.0	Kluyveromyces lactis		Llacer, et al. (2015) *Mol Cell*[22]
3JD5	A	3.1	1.9	-155.0	2.718	267	121	1.39	1.37	EM	7.0	Bos taurus	*	Kaushal, et al. (2014) *PNAS*[23]
3OTO	A	3.8	4.1	20.9	0.538	817	388	1.04	0.96	XRAY	3.7	T. thermophilus HB8		Demirci, et al. (2010) *RNA*[24]
3T1H	A	1.2	3.7	-12.5	0.260	813	409	1.02	0.93	XRAY	3.1	T. thermophilus HB8		Vendeix, et al. (2012) *JMB*[25]
3T1Y	A	1.2	3.6	-7.2	0.422	836	413	1.05	0.94	XRAY	2.8	T. thermophilus HB8		Vendeix, et al. (2012) *JMB*[25]
4A2I	A	6.0	2.1	142.5	0.595	981	439	0.93	0.85	EM	16.5	E. coli		Jomaa, et al. (2011) *RNA*[26]
4ADV	A	8.1	17.5	5.3	3.261	888	431	0.91	0.85	EM	13.5	E. coli		Boehringer, et al. (2012) *J Biol Chem*[27]
4AQY	A	0.9	3.3	-3.5	0.129	790	408	1.02	0.93	XRAY	3.5	T. thermophilus		Matt, et al. (2012) *PNAS*[28]
4B3M	A	1.0	3.5	-13.2	0.242	832	407	1.01	0.90	XRAY	2.9	T. thermophilus HB8		Perez-Fernandez, et al. (2014) *NComm*[29]
4B3R	A	0.9	3.4	-7.4	0.155	825	412	0.99	0.91	XRAY	3.0	T. thermophilus HB8		Perez-Fernandez, et al. (2014) *NComm*[29]
4B3S	A	0.8	3.6	0.6	0.223	824	413	1.02	0.91	XRAY	3.1	T. thermophilus HB8		Perez-Fernandez, et al. (2014) *NComm*[29]
4B3T	A	0.7	3.7	2.2	0.098	820	414	1.04	0.92	XRAY	3.0	T. thermophilus HB8		Perez-Fernandez, et al. (2014) *NComm*[29]
4BTS	AA	10.3	5.1	7.1	1.265	526	259	1.20	1.16	XRAY	3.7	Tetrahymena thermophila		Weisser, et al. (2013) *NSMB*[30]
4BTS	BA	7.2	1.7	71.9	1.589	565	274	1.21	1.19	XRAY	3.7	Tetrahymena thermophila		Weisser, et al. (2013) *NSMB*[30]
4BTS	CA	12.8	4.3	60.4	1.207	535	265	1.19	1.17	XRAY	3.7	Tetrahymena thermophila		Weisser, et al. (2013) *NSMB*[30]
4BTS	DA	4.5	4.0	-23.5	1.243	543	268	1.18	1.20	XRAY	3.7	Tetrahymena thermophila		Weisser, et al. (2013) *NSMB*[30]
4D5L	1	-0.4	0.9	-44.4	1.776	535	264	1.30	1.26	EM	9.0	Oryctolagus cuniculus		Muhs, et al. (2015) *Mol Cell*[31]
4D61	1	-0.4	0.9	-44.4	1.776	535	264	1.30	1.26	EM	9.0	Oryctolagus cuniculus		Muhs, et al. (2015) *Mol Cell*[31]
4DR1	A	2.6	3.4	6.6	0.414	824	408	1.04	1.00	XRAY	3.6	T. thermophilus HB8		Demirci, et al. (2013) *NComm*[32]
4DR2	A	1.5	3.9	-31.9	0.572	834	404	1.01	0.90	XRAY	3.2	T. thermophilus HB8		Demirci, et al. (2013) *NComm*[32]
4DR3	A	4.2	4.0	24.4	0.425	797	390	1.08	0.98	XRAY	3.3	T. thermophilus HB8		Demirci, et al. (2013) *NComm*[32]
4DR4	A	0.4	3.3	-15.9	0.188	825	413	1.06	0.89	XRAY	4.0	T. thermophilus HB8		Demirci, et al. (2013) *NComm*[32]
4DR5	A	1.5	3.7	-20.0	0.394	834	412	1.01	0.88	XRAY	3.5	T. thermophilus HB8		Demirci, et al. (2013) *NComm*[32]
4DR6	A	2.5	3.8	-2.8	0.175	821	407	1.02	0.92	XRAY	3.3	T. thermophilus HB8		Demirci, et al. (2013) *NComm*[32]
4DR7	A	2.7	3.9	4.3	0.128	827	401	1.01	0.94	XRAY	3.8	T. thermophilus HB8		Demirci, et al. (2013) *NComm*[32]
4DUY	A	1.7	3.4	-18.4	0.564	825	410	1.00	0.90	XRAY	3.4	T. thermophilus HB8		Demirci, et al. *To be published*
4DUZ	A	2.6	3.4	16.6	0.678	817	404	1.04	1.00	XRAY	3.7	T. thermophilus HB8		Demirci, et al. *To be published*
4DV0	A	2.0	3.5	-0.6	0.420	831	406	1.02	0.96	XRAY	3.9	T. thermophilus HB8		Demirci, et al. *To be published*
4DV1	A	3.3	4.0	14.8	0.250	814	382	1.07	1.03	XRAY	3.8	T. thermophilus HB8		Demirci, et al. *To be published*
4DV2	A	3.2	3.9	19.6	0.201	808	387	1.08	1.00	XRAY	3.6	T. thermophilus HB8		Demirci, et al. *To be published*
4DV3	A	4.7	4.4	41.2	0.735	786	391	1.09	1.00	XRAY	3.5	T. thermophilus HB8		Demirci, et al. *To be published*
4DV4	A	2.2	3.2	-3.3	0.597	820	401	1.06	0.94	XRAY	3.7	T. thermophilus HB8		Demirci, et al. *To be published*
4DV5	A	4.4	3.9	33.0	0.708	792	395	1.10	1.02	XRAY	3.7	T. thermophilus HB8		Demirci, et al. *To be published*
4DV6	A	1.6	3.6	-24.9	0.369	825	413	1.01	0.90	XRAY	3.3	T. thermophilus HB8		Demirci, et al. *To be published*
4DV7	A	4.2	3.9	31.1	0.469	790	396	1.07	1.01	XRAY	3.3	T. thermophilus HB8		Demirci, et al. *To be published*
4GKJ	A	0.5	3.3	-18.8	0.284	823	410	1.07	0.88	XRAY	3.3	T. thermophilus HB8		Cantara, et al. (2013) *PNAS*[33]
4GKK	A	0.8	3.7	-14.6	0.261	832	412	1.06	0.90	XRAY	3.2	T. thermophilus HB8		Cantara, et al. (2013) *PNAS*[33]
4JI0	A	2.9	3.6	10.6	0.369	822	398	1.05	0.98	XRAY	3.5	T. thermophilus HB8		Demirci, et al. (2013) *RNA*[34]
4JI1	A	4.7	4.2	37.4	0.614	794	396	1.07	0.96	XRAY	3.1	T. thermophilus HB8		Demirci, et al. (2013) *RNA*[34]
4JI2	A	2.7	3.3	7.4	0.368	824	402	1.03	0.97	XRAY	3.6	T. thermophilus HB8		Demirci, et al. (2013) *RNA*[34]
4JI3	A	4.9	4.2	40.4	0.748	795	388	1.07	0.96	XRAY	3.4	T. thermophilus HB8		Demirci, et al. (2013) *RNA*[34]
4JI4	A	2.6	3.4	6.2	0.341	820	405	1.04	1.00	XRAY	3.7	T. thermophilus HB8		Demirci, et al. (2013) *RNA*[34]
4JI5	A	2.3	2.8	-29.8	0.372	831	411	1.05	0.92	XRAY	3.9	T. thermophilus HB8		Demirci, et al. (2013) *RNA*[34]
4JI6	A	2.4	2.8	-28.3	0.723	811	414	1.11	0.96	XRAY	3.5	T. thermophilus HB8		Demirci, et al. (2013) *RNA*[34]
4JI7	A	2.4	3.1	-31.1	0.301	813	417	1.07	0.92	XRAY	3.5	T. thermophilus HB8		Demirci, et al. (2013) *RNA*[34]
4JI8	A	2.5	3.4	-35.8	0.375	831	416	1.07	0.92	XRAY	3.7	T. thermophilus HB8		Demirci, et al. (2013) *RNA*[34]
4JV5	A	1.3	3.4	-3.1	0.533	792	407	1.06	0.95	XRAY	3.2	T. thermophilus HB8		Fernandez, et al. (2013) *Nature*[35]
4JYA	A	1.1	3.6	-9.1	0.203	826	414	1.02	0.91	XRAY	3.1	T. thermophilus HB8		Fernandez, et al. (2013) *Nature*[35]
4K0K	A	1.1	3.3	-5.6	0.196	826	410	1.02	0.91	XRAY	3.4	T. thermophilus HB8		Fernandez, et al. (2013) *Nature*[35]
4KHP	A	1.4	3.9	-33.7	0.393	824	409	0.99	0.88	XRAY	3.1	T. thermophilus HB8		Tourigny, et al. (2013) *JMB*[36]
4KVB	A	2.6	2.9	-26.7	0.378	768	420	1.13	0.98	XRAY	4.2	T. thermophilus HB27		Connetti, et al. *To be published*

Table 3 of 7

PDB		HEAD				PRUNED		RMSD		EXP DETAILS		ORGANISM		REFERENCE
ID	chain	ϕ_{head}	θ_{head}	ψ_{head}	$\lvert\Delta\vec{x}_{\text{head}}\rvert$	body	head	body	head	method	res.	name	mito	
4KZX	i	5.5	2.3	124.6	1.048	544	240	1.23	1.24	XRAY	7.8	Oryctolagus cuniculus		Lomakin, et al. (2013) *Nature*[37]
4KZY	i	2.3	2.2	155.2	0.913	547	240	1.22	1.24	XRAY	7.0	Oryctolagus cuniculus		Lomakin, et al. (2013) *Nature*[37]
4KZZ	i	0.7	1.9	-155.9	1.055	551	240	1.22	1.24	XRAY	7.0	Oryctolagus cuniculus		Lomakin, et al. (2013) *Nature*[37]
4LF4	A	1.7	3.4	-22.2	0.342	815	411	1.01	0.90	XRAY	3.3	T. thermophilus HB8		Demirci, et al. (2013) *To be published*
4LF5	A	3.4	3.9	14.6	0.371	809	389	1.03	0.95	XRAY	3.8	T. thermophilus HB8		Demirci, et al. (2013) *To be published*
4LF6	A	1.9	4.0	-24.8	0.366	809	407	1.01	0.94	XRAY	3.3	T. thermophilus HB8		Demirci, et al. (2013) *To be published*
4LF7	A	1.8	4.0	-33.3	0.415	814	410	1.06	0.92	XRAY	3.1	T. thermophilus HB8		Demirci, et al. (2013) *To be published*
4LF8	A	1.8	4.0	-33.3	0.415	814	410	1.06	0.92	XRAY	3.1	T. thermophilus HB8		Demirci, et al. (2013) *To be published*
4LF9	A	1.4	3.2	-26.2	0.393	812	414	1.01	0.92	XRAY	3.3	T. thermophilus HB8		Demirci, et al. (2013) *To be published*
4LFA	A	1.9	3.4	-11.0	0.313	816	408	1.05	0.95	XRAY	3.6	T. thermophilus HB8		Demirci, et al. (2013) *To be published*
4LFB	A	1.3	3.6	-26.9	0.247	816	415	1.01	0.94	XRAY	3.0	T. thermophilus HB8		Demirci, et al. (2013) *To be published*
4LFC	A	1.2	3.1	-19.9	0.364	825	409	1.00	0.92	XRAY	3.6	T. thermophilus HB8		Demirci, et al. (2013) *To be published*
4NXM	A	2.3	3.2	-3.7	0.457	820	404	1.03	0.94	XRAY	3.6	T. thermophilus HB8		Demirci, et al. *To be published*
4NXN	A	4.1	3.9	29.8	0.569	790	395	1.07	1.00	XRAY	3.5	T. thermophilus HB8		Demirci, et al. *To be published*
4OX9	A	2.1	3.6	-20.7	0.476	818	407	1.03	0.92	XRAY	3.8	T. thermophilus		Dunkle, et al. (2014) *PNAS*[38]
4UER	A	10.7	6.1	33.9	1.071	595	264	1.17	1.13	EM	6.5	Lachancea kluyveri		Aylett, et al. (2015) *NSMB*[39]
4V5O	AA	-0.2	2.5	-47.5	1.059	549	293	1.26	1.19	XRAY	3.9	Tetrahymena thermophila		Rabl, et al. (2011) *Science*[40]
4V5O	BA	1.4	0.4	-26.1	0.898	567	295	1.23	1.20	XRAY	3.9	Tetrahymena thermophila		Rabl, et al. (2011) *Science*[40]
4V8M	AA	-1.9	1.6	-109.1	0.437	464	126	1.35	1.32	EM	5.6	Trypanosoma brucei		Hashem, et al. (2013) *Nature*[41]
4V92	A2	9.4	4.5	56.7	1.519	557	210	1.26	1.27	EM	3.7	Kluyveromyces lactis		Fernandez, et al. (2014) *Cell*[42]
4X62	A	0.3	3.4	-10.5	0.058	808	416	1.02	0.90	XRAY	3.4	T. thermophilus HB8		Choi, et al. (2016) *NSMB*[43]
4X64	A	0.5	3.5	-14.2	0.171	826	415	1.04	0.89	XRAY	3.4	T. thermophilus HB8		Choi, et al. (2016) *NSMB*[43]
4X65	A	0.5	3.5	-15.0	0.025	821	414	1.04	0.89	XRAY	3.3	T. thermophilus HB8		Choi, et al. (2016) *NSMB*[43]
4X66	A	0.4	3.5	-14.8	0.061	816	414	1.04	0.90	XRAY	3.4	T. thermophilus HB8		Choi, et al. (2016) *NSMB*[43]
4YHH	A	3.9	4.9	-8.5	0.179	817	398	1.02	0.98	XRAY	3.4	T. thermophilus HB8		Schedlbauer, et al. (2015) *AAC*[44]
4YY3	A	1.8	3.8	-25.2	0.404	823	407	1.00	0.91	XRAY	3.6	T. thermophilus HB8		Schureck, et al. (2016) *RNA*[45]
5A2Q	2	15.1	6.9	16.9	0.876	602	236	1.20	1.23	EM	3.9	Homo sapiens		Quade, et al. (2015) *NComm*[46]
5AJ3	A	1.7	1.9	-135.8	1.538	378	203	1.13	1.09	EM	3.6	Sus scrofa	*	Greber, et al. (2015) *Science*[47]
5BR8	A	1.3	3.4	-34.0	0.274	831	410	1.00	0.86	XRAY	3.4	T. thermophilus HB8		Sierra, et al. *To be published*
5FLX	1	14.3	9.4	8.4	1.365	580	283	1.30	1.26	EM	3.9	Oryctolagus cuniculus		Yamamoto, et al. (2015) *EMBO J*[48]
5IT9	2	9.0	3.8	63.4	0.845	561	263	1.25	1.17	EM	3.8	Kluyveromyces lactis		Murray, et al. (2016) *Elife*[49]
5IWA	A	4.2	4.3	-15.7	0.282	824	402	1.04	0.92	XRAY	3.5	T. thermophilus HB8		Fabbretti, et al. (2016) *PNAS*[50]
5JB3	2	5.6	5.2	-8.5	1.073	622	291	1.18	1.16	EM	5.3	Pyrococcus abyssi GE5		Coureux, et al. (2016) *NComm*[51]
5JBH	2	-0.3	1.5	-51.5	1.300	622	292	1.18	1.17	EM	5.3	Pyrococcus abyssi GE5		Coureux, et al. (2016) *NComm*[51]
5K0Y	A	0.3	1.2	-48.0	1.553	548	270	1.33	1.26	EM	5.8	Oryctolagus cuniculus		Simonetti, et al. (2016) *Mol Cell*[52]
5LMN	A	2.2	2.3	-38.1	1.909	729	403	1.23	1.00	EM	3.5	T. thermophilus HB8		Hussain, et al. (2016) *Cell*[53]
5LMO	A	11.2	7.2	8.6	1.841	730	387	1.21	1.10	EM	4.3	T. thermophilus HB8		Hussain, et al. (2016) *Cell*[53]
5LMP	A	9.2	11.0	-21.1	1.887	731	400	1.22	1.04	EM	5.3	T. thermophilus HB8		Hussain, et al. (2016) *Cell*[53]
5LMQ	A	12.1	9.7	-21.5	1.702	718	364	1.20	1.07	EM	4.2	T. thermophilus HB8		Hussain, et al. (2016) *Cell*[53]
5LMR	A	15.3	5.7	21.4	2.178	732	359	1.24	1.10	EM	4.5	T. thermophilus HB8		Hussain, et al. (2016) *Cell*[53]
5LMS	A	3.4	3.3	-28.0	1.411	746	394	1.21	1.12	EM	5.1	T. thermophilus HB8		Hussain, et al. (2016) *Cell*[53]
5LMT	A	2.1	2.2	-47.5	1.593	752	404	1.21	0.98	EM	4.2	T. thermophilus HB8		Hussain, et al. (2016) *Cell*[53]
5LMU	A	1.9	2.0	-56.4	1.685	740	407	1.22	0.99	EM	4.0	T. thermophilus HB8		Hussain, et al. (2016) *Cell*[53]
5LMV	A	1.9	1.9	-53.8	1.637	740	409	1.24	0.99	EM	4.9	T. thermophilus HB8		Hussain, et al. (2016) *Cell*[53]
5ME0	A	8.6	16.4	-0.4	3.265	916	393	1.10	0.74	EM	13.5	E. coli K-12		Lopez-Alonso, et al. (2017) *NAR*[54]
5ME1	A	7.8	6.8	-33.9	5.569	916	393	1.10	0.74	EM	13.5	E. coli K-12		Lopez-Alonso, et al. (2017) *NAR*[54]
5MMJ	a	0.5	1.5	-71.4	2.103	863	427	1.16	0.95	EM	3.6	Spinacia oleracea		Bieri, et al. (2017) *EMBO J*[55]
5MY1	A	6.0	2.1	142.5	0.595	981	439	0.93	0.85	EM	7.6	E. coli K-12		Kohler, et al. (2017) *Science*[56]
5NO2	A	9.1	9.2	-30.7	0.514	876	379	1.01	1.08	EM	5.2	E. coli K-12		Lopez-Alonso, et al. (2017) *NAR*[57]
5NO3	A	9.6	10.1	-34.8	0.588	881	363	1.00	1.10	EM	5.2	E. coli K-12		Lopez-Alonso, et al. (2017) *NAR*[57]
5NO4	A	8.0	8.1	-22.4	1.057	871	392	0.99	1.07	EM	5.2	E. coli K-12		Lopez-Alonso, et al. (2017) *NAR*[57]
5O5J	A	-0.1	1.7	-67.4	1.122	908	411	1.04	0.91	EM	3.5	Mycolicibacterium smegmatis MC2 155		Hentschel, et al. (2017) *Cell Rep*[58]

Table 4 of 7

PDB		HEAD				PRUNED		RMSD		EXP DETAILS		ORGANISM		REFERENCE
ID	chain	ϕ_{head}	θ_{head}	ψ_{head}	$\lvert\Delta\vec{x}_{\text{head}}\rvert$	body	head	body	head	method	res.	name	mito	
5OA3	2	8.9	5.2	28.0	1.431	564	239	1.25	1.23	EM	4.3	Homo sapiens		Weisser, et al. (2017) *Mol Cell*[59]
5OPT	E	-0.8	1.3	-87.7	1.855	560	188	1.29	1.23	EM	4.0	Trypanosoma cruzi strain CL Brener		Brito Querido, et al. (2017) *Structure*[60]
5T2A	2	-1.0	1.3	-70.4	1.542	595	205	1.19	1.21	EM	2.9	Leishmania donovani		Zhang, et al. (2016) *NComm*[61]
5UZ4	A	6.8	8.7	1.0	0.547	755	374	1.18	1.09	EM	5.8	E. coli		Razi, et al. (2017) *PNAS*[62]
5VYC	i1	5.5	2.9	87.3	0.462	566	252	1.28	1.22	XRAY	6.0	Homo sapiens		Lomakin, et al. (2017) *Cell Rep*[63]
5VYC	i2	5.2	3.4	84.4	1.265	568	252	1.29	1.22	XRAY	6.0	Homo sapiens		Lomakin, et al. (2017) *Cell Rep*[63]
5VYC	i3	4.6	2.7	68.2	1.135	572	252	1.28	1.22	XRAY	6.0	Homo sapiens		Lomakin, et al. (2017) *Cell Rep*[63]
5VYC	i4	4.2	1.9	77.2	0.783	578	252	1.28	1.22	XRAY	6.0	Homo sapiens		Lomakin, et al. (2017) *Cell Rep*[63]
5VYC	i5	5.4	3.4	69.8	0.869	567	252	1.30	1.22	XRAY	6.0	Homo sapiens		Lomakin, et al. (2017) *Cell Rep*[63]
5VYC	i6	7.4	4.8	51.6	0.786	575	252	1.29	1.23	XRAY	6.0	Homo sapiens		Lomakin, et al. (2017) *Cell Rep*[63]
5WNP	A	0.9	3.3	-8.1	0.253	829	416	1.03	0.92	XRAY	3.3	T. thermophilus HB8		Choi, et al. (2018) *NSMB*[64]
5WNQ	A	2.5	3.6	11.1	0.313	812	407	1.01	0.98	XRAY	3.5	T. thermophilus HB8		Choi, et al. (2018) *NSMB*[64]
5WNR	A	3.1	3.6	14.1	0.348	813	407	1.06	0.99	XRAY	3.5	T. thermophilus HB8		Choi, et al. (2018) *NSMB*[64]
5WNS	A	2.9	3.6	15.0	0.285	804	407	1.06	0.99	XRAY	3.5	T. thermophilus HB8		Choi, et al. (2018) *NSMB*[64]
5WNT	A	0.4	3.5	-2.1	0.223	827	416	1.04	0.91	XRAY	3.3	T. thermophilus HB8		Choi, et al. (2018) *NSMB*[64]
5WNU	A	0.9	3.4	-6.4	0.271	828	414	1.01	0.92	XRAY	3.4	T. thermophilus HB8		Choi, et al. (2018) *NSMB*[64]
5WNV	A	0.6	3.5	-2.9	0.265	826	416	1.03	0.91	XRAY	3.3	T. thermophilus HB8		Choi, et al. (2018) *NSMB*[64]
5X8R	a	1.3	1.0	-73.0	1.222	895	419	1.04	0.90	EM	3.7	Spinacia oleracea		Ahmed, et al. (2017) *NAR*[65]
5XXU	2	-0.2	1.3	-74.5	1.564	562	280	1.28	1.20	EM	3.4	Toxoplasma gondii		Li, et al. (2017) *Cell Res*[66]
5XYI	2	-0.3	1.3	-71.6	1.669	557	305	1.30	1.18	EM	3.4	Trichomonas vaginalis		Li, et al. (2017) *Cell Res*[66]
5XYU	A	-2.5	1.5	-90.0	1.512	690	329	1.17	1.05	EM	3.5	Mycolicibacterium smegmatis MC2 155		Li, et al. (2018) *Protein Cell*[67]
5ZEU	a	-0.1	1.7	-67.4	1.124	908	411	1.03	0.91	EM	3.7	Mycolicibacterium smegmatis MC2 155		Mishra, et al. (2018) *Sci Rep*[68]
6AWB	A	7.7	10.4	20.6	1.861	336	273	1.41	1.34	EM	6.7	E. coli		Demo, et al. (2017) *Elife*[69]
6AWC	A	8.3	10.9	16.6	1.705	415	285	1.43	1.34	EM	7.9	E. coli		Demo, et al. (2017) *Elife*[69]
6AWD	A	8.3	9.9	8.6	2.080	418	212	1.42	1.38	EM	8.1	E. coli		Demo, et al. (2017) *Elife*[69]
6AZ1	1	-1.6	0.6	-90.8	1.016	628	165	1.20	1.12	EM	2.7	Leishmania donovani		Shalev-Benami, et al. (2017) *NComm*[70]
6CAO	A	0.7	3.2	-17.4	0.126	824	415	1.05	0.89	XRAY	3.5	T. thermophilus HB8		Dao, et al. (2018) *RNA*[71]
6CAP	A	0.8	2.9	-14.6	0.227	816	411	1.02	0.89	XRAY	3.4	T. thermophilus HB8		DeMirci *To be published*
6CAQ	A	0.7	2.9	-13.8	0.193	816	411	1.01	0.87	XRAY	3.4	T. thermophilus HB8		DeMirci *To be published*
6CAR	A	1.4	2.9	-12.9	0.196	821	413	0.98	0.89	XRAY	3.4	T. thermophilus HB8		O'Sullivan, et al. (2018) *NAR*[72]
6CAS	A	1.6	2.9	-7.6	0.142	825	409	0.99	0.89	XRAY	3.5	T. thermophilus HB8		O'Sullivan, et al. (2018) *NAR*[72]
6DTI	A	1.5	3.7	-18.0	0.504	792	405	1.04	0.96	XRAY	3.5	T. thermophilus HB8		Vangaveti, et al. (2020) *JMB*[73]
6DZK	A	0.5	1.7	-93.1	1.074	806	381	1.20	1.12	EM	3.6	Mycolicibacterium smegmatis MC2 155		Li, et al. (2018) *PNAS*[74]
6EML	2	-5.7	7.4	24.0	8.808	499	192	1.28	1.24	EM	3.6	S. cerevisiae S288C		Heuer, et al. (2017) *Elife*[75]
6FAI	2	-2.8	8.7	15.9	7.222	496	198	1.23	1.21	EM	3.4	S. cerevisiae S288C		Scaiola, et al. (2018) *EMBO J*[76]
6FEC	A	0.3	1.0	-54.8	1.182	515	247	1.33	1.27	EM	6.3	Homo sapiens		Eliseev, et al. (2018) *NAR*[77]
6FYX	2	-0.6	1.3	-72.2	1.558	526	279	1.25	1.18	EM	3.0	Kluyveromyces lactis NRRL Y-1140		Llacer, et al. (2018) *Elife*[78]
6FYY	2	-0.6	1.2	-75.4	1.554	532	281	1.26	1.18	EM	3.0	Kluyveromyces lactis NRRL Y-1140		Llacer, et al. (2018) *Elife*[78]
6G18	2	-5.7	11.4	26.1	4.775	518	190	1.29	1.15	EM	3.6	Homo sapiens		Ameismeier, et al. (2018) *Nature*[79]
6G4S	2	-8.3	2.6	19.7	8.460	499	103	1.35	1.16	EM	4.0	Homo sapiens		Ameismeier, et al. (2018) *Nature*[79]
6G4W	2	-8.6	3.3	42.7	12.154	451	101	1.33	1.26	EM	4.5	Homo sapiens		Ameismeier, et al. (2018) *Nature*[79]
6G51	2	-0.6	12.9	35.0	5.218	511	200	1.29	1.24	EM	4.1	Homo sapiens		Ameismeier, et al. (2018) *Nature*[79]
6G53	2	2.7	18.0	20.2	6.705	511	200	1.29	1.24	EM	4.5	Homo sapiens		Ameismeier, et al. (2018) *Nature*[79]
6G5H	2	8.0	5.2	53.6	0.912	594	258	1.25	1.22	EM	3.6	Homo sapiens		Ameismeier, et al. (2018) *Nature*[79]
6G5I	2	5.6	13.1	49.3	3.732	495	246	1.30	1.22	EM	3.5	Homo sapiens		Ameismeier, et al. (2018) *Nature*[79]
6GAZ	AA	2.3	2.3	-135.4	1.807	367	208	1.10	1.08	EM	3.1	Sus scrofa	*	Kummer, et al. (2018) *Nature*[80]
6GSM	2	6.4	12.0	-7.4	1.345	531	202	1.34	1.28	EM	5.2	Kluyveromyces lactis NRRL Y-1140		Llacer, et al. (2021) *NAR*[81]
6GSN	2	0.0	0.9	-61.6	0.722	555	276	1.31	1.25	EM	5.8	Kluyveromyces lactis NRRL Y-1140		Llacer, et al. (2021) *NAR*[81]
6HRM	1	1.7	1.9	-81.6	2.081	931	418	1.18	1.05	EM	3.0	E. coli		Schmied, et al. (2018) *Nature*[82]
6MKN	A	1.7	3.7	-17.6	0.595	801	407	1.04	0.97	XRAY	3.5	T. thermophilus HB8		Vangaveti, et al. (2020) *JMB*[73]
6MPF	A	1.0	3.4	-14.2	0.254	833	415	1.04	0.90	XRAY	3.3	T. thermophilus HB8		Vangaveti, et al. (2020) *JMB*[73]
6MPI	A	1.7	3.6	-18.0	0.462	803	413	1.03	0.95	XRAY	3.3	T. thermophilus HB8		Vangaveti, et al. (2020) *JMB*[73]

Table 5 of 7

PDB		HEAD				PRUNED		RMSD		EXP DETAILS		ORGANISM		REFERENCE
ID	chain	ϕ_{head}	θ_{head}	ψ_{head}	$\lvert\Delta\vec{x}_{\text{head}}\rvert$	body	head	body	head	method	res.	name	mito	
6NEQ	A	12.8	2.3	34.3	3.344	347	150	1.27	1.37	EM	3.3	Bos taurus	*	Koripella, et al. (2019) *iScience*[83]
6NF8	A	10.9	2.4	75.0	3.816	328	121	1.32	1.30	EM	3.5	Bos taurus	*	Koripella, et al. (2019) *iScience*[83]
6NQB	A	7.3	7.4	-7.8	2.589	713	301	1.13	1.39	EM	3.8	E. coli		Razi, et al. (2019) *NAR*[84]
6NY6	A	2.2	3.1	0.9	0.446	826	408	1.03	0.95	XRAY	3.7	T. thermophilus HB8		Pavelich, et al. (2019) *NAR*[85]
6O7K	g	1.9	1.9	-72.6	1.206	906	420	1.07	1.01	EM	4.2	E. coli		Kaledhonkar, et al. (2019) *Nature*[86]
6OKK	A	4.8	2.6	114.2	0.652	619	139	1.16	1.21	EM	3.3	Plasmodium falciparum 3D7		Wong, et al. (2014) *Elife*[20]
6P4G	2	9.1	4.4	48.6	0.768	604	262	1.19	1.12	EM	3.1	Oryctolagus cuniculus		Acosta-Reyes, et al. (2019) *EMBO J*[87]
6P4H	2	6.9	4.1	63.8	0.712	610	271	1.20	1.14	EM	3.2	Oryctolagus cuniculus		Acosta-Reyes, et al. (2019) *EMBO J*[87]
6RBD	2	-3.8	6.0	7.9	11.117	472	186	1.26	1.31	EM	3.5	S. cerevisiae S288C		Mitterer, et al. (2019) *NComm*[88]
6RBE	2	8.2	11.5	27.6	4.831	498	221	1.31	1.28	EM	3.8	S. cerevisiae S288C		Mitterer, et al. (2019) *NComm*[88]
6RW4	A	3.5	1.7	-160.7	2.803	348	198	1.19	1.15	EM	3.0	Homo sapiens	*	Khawaja, et al. (2020) *NComm*[89]
6RW5	A	3.7	2.0	-160.7	2.866	324	194	1.24	1.13	EM	3.1	Homo sapiens	*	Khawaja, et al. (2020) *NComm*[89]
6SPC	a	1.5	2.8	-43.6	0.410	851	96	0.99	1.17	EM	3.0	Pseudomonas aeruginosa		Halfon, et al. (2019) *PNAS*[90]
6SPE	a	2.3	1.8	-93.7	2.931	577	352	1.40	1.33	EM	3.6	Pseudomonas aeruginosa		Halfon, et al. (2019) *PNAS*[90]
6SW9	2	-2.3	1.4	-77.6	3.286	345	170	1.43	1.36	EM	4.2	Pyrococcus abyssi GE5		Coureux, et al. (2020) *Commun Biol*[91]
6SWC	2	-2.2	1.7	-68.0	2.910	355	194	1.43	1.40	EM	3.3	Pyrococcus abyssi GE5		Coureux, et al. (2020) *Commun Biol*[91]
6TMF	A	6.2	3.1	20.5	0.862	627	296	1.13	1.18	EM	2.8	Thermococcus celer Vu 13 = JCM 8558		Nurenberg-Goloub, et al. (2020) *EMBO J*[92]
6V3E	sN1	10.7	5.7	-39.8	2.769	551	317	1.32	1.25	EM	4.4	Acinetobacter baumannii		Morgan, et al. (2020) *mBio*[93]
6W2S	A	12.3	7.4	35.5	1.473	546	247	1.27	1.17	EM	3.0	Oryctolagus cuniculus		Neupane, et al. (2020) *Elife*[94]
6W2T	a	14.3	10.3	4.0	1.657	541	250	1.26	1.19	EM	3.4	Oryctolagus cuniculus		Neupane, et al. (2020) *Elife*[94]
6W6K	A	6.4	3.5	-15.7	2.301	749	368	1.27	1.25	EM	3.6	E. coli K-12		Jahagirdar, et al. (2020) *RNA*[95]
6W77	A	11.3	14.8	38.9	4.937	767	361	1.16	1.19	EM	3.6	E. coli K-12		Jahagirdar, et al. (2020) *RNA*[95]
6W7M	A	9.2	12.9	17.8	3.563	679	323	1.28	1.19	EM	3.8	E. coli K-12		Jahagirdar, et al. (2020) *RNA*[95]
6W7N	A	9.3	8.3	19.5	3.271	756	366	1.18	1.23	EM	3.4	E. coli K-12		Jahagirdar, et al. (2020) *RNA*[95]
6WDR	2	11.5	11.3	42.1	4.650	248	173	1.42	1.38	EM	3.7	S. cerevisiae S288C		Rai, et al. (2021) *RNA*[96]
6XE0	W	1.3	1.3	-81.6	1.871	856	388	1.19	1.15	EM	6.8	E. coli K-12		Washburn, et al. (2020) *iScience*[97]
6Y7C	2	-5.0	5.4	29.1	11.461	367	104	1.37	1.41	EM	3.8	S. cerevisiae S288C		Shayan, et al. (2020) *Molecules*[98]
6YAL	2	-0.3	1.3	-90.1	2.178	494	258	1.37	1.26	EM	3.0	Oryctolagus cuniculus		Simonetti, et al. (2020) *Cell Rep*[99]
6YAM	2	-0.3	1.4	-90.2	2.179	492	258	1.37	1.26	EM	3.6	Oryctolagus cuniculus		Simonetti, et al. (2020) *Cell Rep*[99]
6YAN	2	0.0	1.5	-72.9	2.481	471	259	1.39	1.28	EM	3.5	Oryctolagus cuniculus		Simonetti, et al. (2020) *Cell Rep*[99]
6YW5	aa	3.2	3.9	-66.9	0.537	506	224	1.20	1.16	EM	2.9	Neurospora crassa OR74A	*	Itoh, et al. (2020) *NComm*[100]
6ZCE	A	12.0	6.8	43.6	1.206	589	246	1.25	1.26	EM	5.3	S. cerevisiae S288C		Kratzat, et al. (2021) *EMBO J*[101]
6ZJ3	S1	-1.5	0.7	-28.6	1.006	653	165	1.16	1.10	EM	3.1	Euglena gracilis		Matzov, et al. (2020) *NAR*[102]
6ZLW	2	6.4	5.3	-6.1	2.028	574	264	1.24	1.17	EM	2.6	Homo sapiens		Thoms, et al. (2020) *Science*[103]
6ZMT	2	6.2	12.2	-8.3	2.159	424	263	1.32	1.20	EM	3.0	Homo sapiens		Thoms, et al. (2020) *Science*[103]
6ZMW	A	9.7	6.0	44.6	1.086	582	227	1.21	1.25	EM	3.7	Homo sapiens		Brito Querido, et al. (2020) *Science*[104]
6ZN5	2	2.0	2.5	-50.0	1.967	543	279	1.28	1.21	EM	3.2	Homo sapiens		Thoms, et al. (2020) *Science*[103]
6ZOJ	2	7.0	3.8	10.8	2.518	490	256	1.33	1.24	EM	2.8	Homo sapiens		Schubert, et al. (2020) *NSMB*[105]
6ZON	2	9.7	6.5	40.9	1.913	569	171	1.23	1.38	EM	3.0	Homo sapiens		Thoms, et al. (2020) *Science*[103]
6ZP4	2	5.4	13.9	-10.6	1.927	576	257	1.21	1.13	EM	2.9	Homo sapiens		Thoms, et al. (2020) *Science*[103]
6ZU9	2	-0.2	0.9	-48.0	1.085	594	290	1.21	1.19	EM	6.2	S. cerevisiae S288C		Kratzat, et al. (2021) *EMBO J*[101]
6ZUO	2	3.4	13.7	39.5	4.051	477	271	1.30	1.21	EM	3.1	Homo sapiens		Plassart, et al. (2021) *Elife*[106]
6ZV6	2	4.9	4.6	32.8	1.759	555	267	1.23	1.20	EM	2.9	Homo sapiens		Plassart, et al. (2021) *Elife*[106]
6ZVH	2	-0.4	1.1	-64.2	1.526	576	291	1.22	1.17	EM	2.9	Homo sapiens		Sinha, et al. (2020) *Elife*[107]
6ZVI	h	0.8	1.3	-21.8	1.126	558	290	1.24	1.15	EM	3.0	S. cerevisiae		Sinha, et al. (2020) *Elife*[107]
6ZVJ	2	12.3	6.9	54.0	1.441	548	244	1.28	1.23	EM	3.8	Homo sapiens		Kratzat, et al. (2021) *EMBO J*[101]
6ZXD	2	-0.2	16.4	10.5	5.479	461	251	1.33	1.18	EM	3.2	Homo sapiens		Ameismeier, et al. (2020) *Nature*[108]
6ZXE	2	2.7	11.0	43.7	3.772	436	225	1.31	1.25	EM	3.0	Homo sapiens		Ameismeier, et al. (2020) *Nature*[108]
6ZXF	2	5.9	5.1	76.0	3.278	513	250	1.38	1.21	EM	3.7	Homo sapiens		Ameismeier, et al. (2020) *Nature*[108]
6ZXG	2	3.9	5.1	43.3	2.455	565	195	1.27	1.23	EM	2.6	Homo sapiens		Ameismeier, et al. (2020) *Nature*[108]
6ZXH	2	3.9	5.0	44.9	2.514	559	180	1.23	1.25	EM	2.7	Homo sapiens		Ameismeier, et al. (2020) *Nature*[108]
7A09	2	6.2	13.4	-7.9	1.934	572	252	1.28	1.19	EM	3.5	Homo sapiens		Kratzat, et al. (2021) *EMBO J*[101]

Table 6 of 7

PDB		HEAD				PRUNED		RMSD		EXP DETAILS		ORGANISM		REFERENCE
ID	chain	ϕ_{head}	θ_{head}	ψ_{head}	$\lvert\Delta\vec{x}_{\text{head}}\rvert$	body	head	body	head	method	res.	name	mito	
7A1G	2	12.0	8.0	37.9	1.824	577	260	1.22	1.17	EM	3.0	S. cerevisiae S288C		Kratzat, et al. (2021) *EMBO J*[101]
7ASE	0	4.3	17.0	-18.2	3.309	476	131	1.35	1.32	EM	3.3	Trypanosoma cruzi		Bochler, et al. (2020) *Cell Rep*[109]
7BOE	A	9.2	9.2	-25.4	0.490	949	411	0.79	0.83	EM	2.9	E. coli K-12		Schedlbauer, et al. (2021) *Sci Adv*[110]
7BOH	A	6.6	13.4	10.8	2.267	910	414	0.77	0.84	EM	2.8	E. coli K-12		Schedlbauer, et al. (2021) *Sci Adv*[110]
7DUG	A	0.7	2.9	-14.2	0.239	818	409	1.04	0.91	XRAY	3.8	T. thermophilus HB8		Demirci *To be published*
7DUH	A	0.6	2.9	-10.7	0.241	810	407	1.04	0.93	XRAY	3.8	T. thermophilus HB8		Demirci *To be published*
7DUI	A	0.7	3.0	-11.7	0.228	817	409	1.02	0.90	XRAY	3.6	T. thermophilus HB8		Demirci *To be published*
7DUJ	A	0.7	3.1	-12.6	0.193	816	408	1.04	0.92	XRAY	3.8	T. thermophilus HB8		Demirci *To be published*
7DUK	A	0.7	3.1	-7.8	0.238	797	408	1.06	0.95	XRAY	3.6	T. thermophilus HB8		Demirci *To be published*
7DUL	A	0.7	3.1	-11.6	0.085	815	408	1.04	0.92	XRAY	3.6	T. thermophilus HB8		DeMirci, et al. *To be published*
7JQB	A	0.6	2.5	-63.9	1.310	593	280	1.21	1.17	EM	2.7	Oryctolagus cuniculus		Yuan, et al. (2020) *Mol Cell*[111]
7JQC	A	6.2	3.1	30.9	1.609	602	214	1.22	1.25	EM	3.3	Oryctolagus cuniculus		Yuan, et al. (2020) *Mol Cell*[111]
7K5I	2	6.1	5.7	-3.6	3.578	487	253	1.32	1.25	EM	2.9	Homo sapiens		Shi, et al. (2020) *Biorxiv*[112]
7KWG	a	1.4	1.6	-69.9	0.580	800	372	1.07	1.09	EM	3.8	Staph. aureus subsp. aureus NCTC 8325		Belinite, et al. (2021) *Front Mol Biosci*[113]
7M4U	a	6.8	4.4	-30.3	1.750	719	365	1.35	1.12	EM	2.7	Acinetobacter baumannii AB0057		Zhang, et al. (2021) *mBio*[114]
7NAR	A	8.1	12.0	-31.8	1.086	890	399	1.06	0.78	EM	3.0	E. coli K-12		Schedlbauer, et al. (2021) *Sci Adv*[110]
7NAT	A	9.1	15.4	15.1	2.004	785	398	0.88	0.89	EM	3.6	E. coli K-12		Schedlbauer, et al. (2021) *Sci Adv*[110]
7NAU	A	9.7	15.2	22.8	2.235	825	398	0.92	0.87	EM	3.8	E. coli K-12		Schedlbauer, et al. (2021) *Sci Adv*[110]
7NAV	A	8.5	15.1	12.7	2.107	814	404	1.01	1.00	EM	4.8	E. coli K-12		Schedlbauer, et al. (2021) *Sci Adv*[110]
7NAX	A	10.3	15.2	26.3	2.621	867	412	0.77	0.87	EM	3.0	E. coli		Schedlbauer, et al. (2021) *Sci Adv*[110]
7OE0	A	11.3	13.6	9.3	2.251	825	375	0.85	1.14	EM	2.7	E. coli BW25113		Maksimova, et al. (2021) *Int J Mol Sci*[115]
7OE1	A	11.5	12.8	-24.7	0.725	891	295	0.95	1.25	EM	3.0	E. coli str. K-12 substr. MG1655		Maksimova, et al. (2021) *Int J Mol Sci*[115]
7OOC	5	-0.3	0.7	-167.9	1.719	789	393	1.12	1.08	EM	3.7	Mycoplasma pneumoniae M129		Xue, et al. (2022) *Nature*[116]
7P2E	A	3.5	1.4	-170.1	2.780	329	197	1.20	1.10	EM	2.4	Homo sapiens	*	Singh, et al. *To be published*
7PNT	A	1.1	8.4	11.3	3.747	286	156	1.23	1.16	EM	3.2	Mus musculus	*	Itoh, et al. (2022) *Nature*[117]
7PNU	A	-1.2	9.6	8.3	4.414	308	105	1.17	1.11	EM	3.1	Mus musculus	*	Itoh, et al. (2022) *Nature*[117]
7PNV	A	1.8	1.7	-0.3	3.629	348	205	1.08	1.12	EM	3.1	Mus musculus	*	Itoh, et al. (2022) *Nature*[117]
7PNW	A	5.4	0.2	-44.1	2.911	345	205	1.10	1.14	EM	3.1	Mus musculus	*	Itoh, et al. (2022) *Nature*[117]
7PNX	A	1.1	6.1	-4.5	3.830	304	198	1.17	1.08	EM	2.8	Homo sapiens	*	Itoh, et al. (2022) *Nature*[117]
7PNY	A	2.4	0.7	73.5	3.099	341	200	1.13	1.09	EM	3.1	Homo sapiens	*	Itoh, et al. (2022) *Nature*[117]
7PNZ	A	2.6	0.6	82.9	3.160	345	201	1.12	1.10	EM	3.1	Homo sapiens	*	Itoh, et al. (2022) *Nature*[117]
7PO0	A	2.9	1.5	61.2	3.552	346	200	1.13	1.09	EM	2.9	Homo sapiens	*	Itoh, et al. (2022) *Nature*[117]
7PO1	A	2.9	2.1	-144.0	1.767	316	202	1.10	1.10	EM	2.9	Homo sapiens	*	Itoh, et al. (2022) *Nature*[117]
7PO2	A	1.9	2.5	-140.8	1.399	331	206	1.15	1.08	EM	3.1	Homo sapiens	*	Itoh, et al. (2022) *Nature*[117]
7PO3	A	2.8	2.7	-143.8	1.673	323	204	1.13	1.12	EM	2.9	Homo sapiens	*	Itoh, et al. (2022) *Nature*[117]
7PWF	2	-0.7	1.4	-90.6	2.950	471	244	1.39	1.23	EM	2.9	Giardia lamblia ATCC 50803		Hiregange, et al. (2022) *NAR*[118]
7QP6	A	6.6	12.5	-7.4	0.866	595	230	1.22	1.26	EM	4.7	Homo sapiens		Yi, et al. (2022) *NAR*[119]
7QP7	A	-0.5	1.4	-89.7	0.815	616	259	1.20	1.13	EM	3.7	Homo sapiens		Yi, et al. (2022) *NAR*[119]
7SYG	2	4.9	4.0	53.9	2.162	476	195	1.36	1.35	EM	4.3	Oryctolagus cuniculus		Brown, et al. *To be published*
7SYH	2	4.8	3.9	53.5	1.947	465	194	1.39	1.34	EM	4.6	Oryctolagus cuniculus		Brown, et al. *To be published*
7SYI	2	4.9	3.7	48.2	2.051	486	215	1.38	1.40	EM	4.5	Oryctolagus cuniculus		Brown, et al. *To be published*
7SYJ	2	5.5	4.2	49.7	1.838	464	207	1.37	1.38	EM	4.8	Oryctolagus cuniculus		Brown, et al. *To be published*
7SYK	2	5.7	4.6	51.7	1.930	490	220	1.35	1.37	EM	4.2	Oryctolagus cuniculus		Brown, et al. *To be published*
7SYL	2	6.2	4.3	44.9	2.003	496	215	1.37	1.35	EM	4.5	Oryctolagus cuniculus		Brown, et al. *To be published*
7SYM	2	11.0	6.5	39.5	1.958	464	224	1.38	1.31	EM	4.8	Oryctolagus cuniculus		Brown, et al. *To be published*
7SYN	2	14.9	7.0	18.2	1.951	487	230	1.35	1.28	EM	4.0	Oryctolagus cuniculus		Brown, et al. *To be published*
7SYO	2	14.2	10.5	-2.4	2.074	437	209	1.39	1.30	EM	4.6	Oryctolagus cuniculus		Brown, et al. (2022) *EMBO J*[120]
7SYP	2	14.7	9.6	3.3	1.975	488	238	1.34	1.28	EM	4.0	Oryctolagus cuniculus		Brown, et al. (2022) *EMBO J*[120]
7SYQ	2	14.6	9.5	3.6	2.147	503	237	1.34	1.23	EM	3.8	Oryctolagus cuniculus		Brown, et al. (2022) *EMBO J*[120]
7SYR	2	-0.3	1.2	-74.7	1.967	523	272	1.32	1.25	EM	3.6	Oryctolagus cuniculus		Brown, et al. (2022) *EMBO J*[120]
7SYS	2	0.0	1.5	-75.7	2.039	518	275	1.32	1.27	EM	3.5	Oryctolagus cuniculus		Brown, et al. (2022) *EMBO J*[120]
7SYT	2	-0.2	1.4	-70.1	2.024	503	260	1.34	1.30	EM	4.4	Oryctolagus cuniculus		Brown, et al. *To be published*

Table 7 of 7

PDB		HEAD				PRUNED		RMSD		EXP DETAILS		ORGANISM		REFERENCE
ID	chain	ϕ_{head}	θ_{head}	ψ_{head}	$\lvert\Delta\vec{x}_{\text{head}}\rvert$	body	head	body	head	method	res.	name	mito	
7SYU	2	0.2	1.3	-68.1	2.001	486	253	1.34	1.27	EM	4.6	Oryctolagus cuniculus		Brown, et al. *To be published*
7SYV	2	14.3	9.5	2.5	2.065	486	233	1.36	1.24	EM	3.9	Oryctolagus cuniculus		Brown, et al. (2022) *EMBO J*[120]
7SYW	2	-0.6	1.8	-76.5	1.962	510	270	1.32	1.27	EM	3.7	Oryctolagus cuniculus		Brown, et al. *To be published*
7SYX	2	-0.2	2.0	-75.5	1.932	501	270	1.32	1.26	EM	3.7	Oryctolagus cuniculus		Brown, et al. *To be published*
7TQL	2	-0.8	1.7	-73.1	1.582	570	278	1.24	1.18	EM	3.4	Homo sapiens		Lapointe, et al. (2022) *Nature*[121]
7UPH	I	5.6	2.2	65.8	1.361	756	394	1.37	1.19	EM	4.2	E. coli		Kim, et al. (2022) *NCB*[122]
7V2L	A	12.6	12.8	-25.8	1.763	734	366	1.19	0.99	EM	3.3	T. thermophilus HB8		Singh, et al. (2022) *ACS Chem Biol*[123]
7V2M	A	13.4	13.0	-26.6	2.042	659	359	1.19	1.01	EM	3.4	T. thermophilus HB8		Singh, et al. (2022) *ACS Chem Biol*[123]
7V2N	A	14.4	5.4	6.5	1.742	654	348	1.15	1.08	EM	3.6	T. thermophilus HB8		Singh, et al. (2022) *ACS Chem Biol*[123]
7V2O	A	5.1	5.0	-45.5	1.906	671	379	1.20	1.07	EM	3.5	T. thermophilus HB8		Singh, et al. (2022) *ACS Chem Biol*[123]
7V2P	A	3.1	2.3	-54.6	1.358	697	400	1.13	1.09	EM	3.3	T. thermophilus HB8		Singh, et al. (2022) *ACS Chem Biol*[123]
7V2Q	A	13.4	13.4	-18.5	2.322	632	367	1.15	1.06	EM	3.2	T. thermophilus HB8		Singh, et al. (2022) *ACS Chem Biol*[123]
7WTT	2	-8.3	3.4	39.1	12.841	309	152	1.31	1.12	EM	3.1	Homo sapiens		Cheng, et al. *To be published*
7WTU	2	-8.1	3.7	40.0	12.799	315	153	1.32	1.16	EM	3.0	Homo sapiens		Cheng, et al. *To be published*
7WTV	2	-9.1	0.3	-66.1	14.518	438	145	1.35	1.17	EM	3.5	Homo sapiens		Cheng, et al. *To be published*
7WTX	2	-7.8	4.4	26.1	8.789	493	156	1.29	1.17	EM	3.1	Homo sapiens		Cheng, et al. *To be published*
7WTZ	2	-7.7	5.4	22.2	7.931	491	167	1.29	1.19	EM	3.0	Homo sapiens		Cheng, et al. *To be published*
7WU0	2	-7.4	5.4	23.8	7.988	485	166	1.31	1.19	EM	3.3	Homo sapiens		Cheng, et al. *To be published*
7ZAG	2	-2.0	1.6	-88.9	2.677	411	228	1.38	1.34	EM	2.8	Pyrococcus abyssi GE5		Kazan, et al. (2022) *NAR*[124]
7ZAH	2	-2.4	1.8	-87.9	2.678	405	228	1.36	1.35	EM	2.7	Pyrococcus abyssi GE5		Kazan, et al. (2022) *NAR*[124]
7ZAI	2	-2.1	1.7	-87.6	2.639	409	228	1.37	1.34	EM	2.6	Pyrococcus abyssi GE5		Kazan, et al. (2022) *NAR*[124]
7ZHG	2	-1.7	1.3	-95.2	2.603	456	230	1.39	1.30	EM	2.2	Pyrococcus abyssi GE5		Kazan, et al. (2022) *NAR*[124]

References

[1] Andrew P. Carter, William M. Clemons, Ditlev E. Brodersen, Robert J. Morgan-Warren, Brian T. Wimberly, and V. Ramakrishnan. Functional insights from the structure of the 30S ribosomal subunit and its interactions with antibiotics. *Nature*, 407(6802):340–348, sep 2000.

[2] Frank Schluenzen, Ante Tocilj, Raz Zarivach, Joerg Harms, Marco Gluehmann, Daniela Janell, Anat Bashan, Heike Bartels, Ilana Agmon, François Franceschi, and Ada Yonath. Structure of Functionally Activated Small Ribosomal Subunit at 3.3 Å Resolution. *Cell*, 102(5):615–623, sep 2000.

[3] Ditlev E. Brodersen, William M. Clemons, Andrew P. Carter, Robert J. Morgan-Warren, Brian T. Wimberly, and V. Ramakrishnan. The Structural Basis for the Action of the Antibiotics Tetracycline, Pactamycin, and Hygromycin B on the 30S Ribosomal Subunit. *Cell*, 103(7):1143–1154, dec 2000.

[4] Andrew P. Carter, William M. Clemons, Ditlev E. Brodersen, Robert J. Morgan-Warren, Thomas Hartsch, Brian T. Wimberly, and V. Ramakrishnan. Crystal Structure of an Initiation Factor Bound to the 30 S Ribosomal Subunit. *Science*, 291(5503):498–501, jan 2001.

[5] M. Pioletti. Crystal structures of complexes of the small ribosomal subunit with tetracycline, edeine and IF3. *The EMBO Journal*, 20(8):1829–1839, apr 2001.

[6] James M. Ogle, Ditlev E. Brodersen, William M. Clemons, Michael J. Tarry, Andrew P. Carter, and V. Ramakrishnan. Recognition of Cognate Transfer RNA by the 30 S Ribosomal Subunit. *Science*, 292(5518):897–902, may 2001.

[7] Brian T. Wimberly, Ditlev E. Brodersen, William M. Clemons, Robert J. Morgan-Warren, Andrew P. Carter, Clemens Vonrhein, Thomas Hartsch, and V. Ramakrishnan. Structure of the 30S ribosomal subunit. *Nature*, 407(6802):327–339, sep 2000.

[8] Gulnara Zh. Yusupova, Marat M. Yusupov, J.H.D. Cate, and Harry F. Noller. The Path of Messenger RNA through the Ribosome. *Cell*, 106(2):233–241, jul 2001.

[9] James M. Ogle, Frank V. Murphy, Michael J. Tarry, and V. Ramakrishnan. Selection of tRNA by the Ribosome Requires a Transition from an Open to a Closed Form. *Cell*, 111(5):721–732, nov 2002.

[10] Frank V Murphy, Venki Ramakrishnan, Andrzej Malkiewicz, and Paul F Agris. The role of modifications in codon discrimination by tRNALysUUU. *Nature Structural & Molecular Biology*, 11(12):1186–1191, nov 2004.

[11] Frank V Murphy and V Ramakrishnan. Structure of a purine-purine wobble base pair in the decoding center of the ribosome. *Nature Structural & Molecular Biology*, 11(12):1251–1252, nov 2004.

[12] Tatsuya Kaminishi, Daniel N. Wilson, Chie Takemoto, Joerg M. Harms, Masahito Kawazoe, Frank Schluenzen, Kyoko Hanawa-Suetsugu, Mikako Shirouzu, Paola Fucini, and Shigeyuki Yokoyama. A Snapshot of the 30S Ribosomal Subunit Capturing mRNA via the Shine-Dalgarno Interaction. *Structure*, 15(3):289–297, mar 2007.

[13] James B. Murray, Samy O. Meroueh, Rupert J.M. Russell, Georg Lentzen, Jalal Haddad, and Shahriar Mobashery. Interactions of Designer Antibiotics and the Bacterial Ribosomal Aminoacyl-tRNA Site. *Chemistry & Biology*, 13(2):129–138, feb 2006.

[14] Frank Schluenzen, Chie Takemoto, Daniel N Wilson, Tatsuya Kaminishi, Joerg M Harms, Kyoko Hanawa-Suetsugu, Witold Szaflarski, Masahito Kawazoe, Mikako Shirouzu, Knud H Nierhaus, Shigeyuki Yokoyama, and Paola Fucini. The antibiotic kasugamycin mimics mRNA nucleotides to destabilize tRNA binding and inhibit canonical translation initiation. *Nature Structural & Molecular Biology*, 13(10):871–878, sep 2006.

[15] Albert Weixlbaumer, Frank V Murphy, Agnieszka Dziergowska, Andrzej Malkiewicz, Franck A P Vendeix, Paul F Agris, and V Ramakrishnan. Mechanism for expanding the decoding capacity of transfer RNAs by modification of uridines. *Nature Structural & Molecular Biology*, 14(6):498–502, may 2007.

[16] Christine M. Dunham, Maria Selmer, Steven S. Phelps, Ann C. Kelley, Tsutomu Suzuki, Simpson Joseph, and V. Ramakrishnan. Structures of tRNAs with an expanded anticodon loop in the decoding center of the 30S ribosomal subunit. *RNA*, 13(6):817–823, apr 2007.

[17] Shinya Kurata, Albert Weixlbaumer, Takashi Ohtsuki, Tomomi Shimazaki, Takeshi Wada, Yohei Kirino, Kazuyuki Takai, Kimitsuna Watanabe, V. Ramakrishnan, and Tsutomu Suzuki. Modified Uridines with C5-methylene Substituents at the First Position of the tRNA Anticodon Stabilize U·G Wobble Pairing during Decoding. *Journal of Biological Chemistry*, 283(27):18801–18811, jul 2008.

[18] Qiang Guo, Yi Yuan, Yanji Xu, Boya Feng, Liang Liu, Kai Chen, Ming Sun, Zhixiu Yang, Jianlin Lei, and Ning Gao. Structural basis for the function of a small GTPase RsgA on the 30S ribosomal subunit maturation revealed by cryoelectron microscopy. *Proceedings of the National Academy of Sciences*, 108(32):13100–13105, jul 2011.

[19] Qiang Guo, Simon Goto, Yuling Chen, Boya Feng, Yanji Xu, Akira Muto, Hyouta Himeno, Haiteng Deng, Jianlin Lei, and Ning Gao. Dissecting the in vivo assembly of the 30S ribosomal subunit reveals the role of RimM and general features of the assembly process. *Nucleic Acids Research*, 41(4):2609–2620, jan 2013.

[20] Wilson Wong, Xiao chen Bai, Alan Brown, Israel S Fernandez, Eric Hanssen, Melanie Condron, Yan Hong Tan, Jake Baum, and Sjors HW Scheres. Cryo-EM structure of the Plasmodium falciparum 80S ribosome bound to the anti-protozoan drug emetine. *eLife*, 3, jun 2014.

[21] Tanweer Hussain, Jose L. Llácer, Israel S. Fernández, Antonio Munoz, Pilar Martin-Marcos, Christos G. Savva, Jon R. Lorsch, Alan G. Hinnebusch, and V. Ramakrishnan. Structural Changes Enable Start Codon Recognition by the Eukaryotic Translation Initiation Complex. *Cell*, 159(3):597–607, oct 2014.

[22] Jose L. Llácer, Tanweer Hussain, Laura Marler, Colin Echeverría Aitken, Anil Thakur, Jon R. Lorsch, Alan G. Hinnebusch, and V. Ramakrishnan. Conformational Differences between Open and Closed States of the Eukaryotic Translation Initiation Complex. *Molecular Cell*, 59(3):399–412, aug 2015.

[23] Prem S. Kaushal, Manjuli R. Sharma, Timothy M. Booth, Emdadul M. Haque, Chang-Shung Tung, Karissa Y. Sanbonmatsu, Linda L. Spremulli, and Rajendra K. Agrawal. Cryo-EM structure of the small subunit of the mammalian mitochondrial ribosome. *Proceedings of the National Academy of Sciences*, 111(20):7284–7289, may 2014.

[24] Hasan Demirci, Frank Murphy, Riccardo Belardinelli, Ann C. Kelley, V. Ramakrishnan, Steven T. Gregory, Albert E. Dahlberg, and Gerwald Jogl. Modification of 16S ribosomal RNA by the KsgA methyltransferase restructures the 30S subunit to optimize ribosome function. *RNA*, 16(12):2319–2324, oct 2010.

[25] Franck A.P. Vendeix, Frank V. Murphy, William A. Cantara, Grażyna Leszczyńska, Estella M. Gustilo, Brian Sproat, Andrzej Malkiewicz, and Paul F. Agris. Human tRNALys3UUU Is Pre-Structured by Natural Modifications for Cognate and Wobble Codon Binding through Keto–Enol Tautomerism. *Journal of Molecular Biology*, 416(4):467–485, mar 2012.

[26] Ahmad Jomaa, Geordie Stewart, Jason A. Mears, Inga Kireeva, Eric D. Brown, and Joaquin Ortega. Cryo-electron microscopy structure of the 30S subunit in complex with the YjeQ biogenesis factor. *RNA*, 17(11):2026–2038, sep 2011.

[27] Daniel Boehringer, Heather C. O'Farrell, Jason P. Rife, and Nenad Ban. Structural Insights into Methyltransferase KsgA Function in 30S Ribosomal Subunit Biogenesis. *Journal of Biological Chemistry*, 287(13):10453–10459, mar 2012.

[28] Tanja Matt, Chyan Leong Ng, Kathrin Lang, Su-Hua Sha, Rashid Akbergenov, Dmitri Shcherbakov, Martin Meyer, Stefan Duscha, Jing Xie, Srinivas R. Dubbaka, D
'eborah Perez-Fernandez, Andrea Vasella, V. Ramakrishnan, Jochen Schacht, and Erik C. Böttger. Dissociation of antibacterial activity and aminoglycoside ototoxicity in the 4-monosubstituted 2-deoxystreptamine apramycin. *Proceedings of the National Academy of Sciences*, 109(27):10984–10989, jun 2012.

[29] D
'eborah Perez-Fernandez, Dmitri Shcherbakov, Tanja Matt, Ng Chyan Leong, Iwona Kudyba, Stefan Duscha, Heithem Boukari, Rashmi Patak, Srinivas Reddy Dubbaka, Kathrin Lang, Martin Meyer, Rashid Akbergenov, Pietro Freihofer, Swapna Vaddi, Pia Thommes, V. Ramakrishnan, Andrea Vasella, and Erik C. Böttger. 4'-O-substitutions determine selectivity of aminoglycoside antibiotics. *Nature Communications*, 5(1), jan 2014.

[30] Melanie Weisser, Felix Voigts-Hoffmann, Julius Rabl, Marc Leibundgut, and Nenad Ban. The crystal structure of the eukaryotic 40S ribosomal subunit in complex with eIF1 and eIF1A. *Nature Structural & Molecular Biology*, 20(8):1015–1017, jul 2013.

[31] Margarita Muhs, Tarek Hilal, Thorsten Mielke, Maxim A. Skabkin, Karissa Y. Sanbonmatsu, Tatyana V. Pestova, and Christian M.T. Spahn. Cryo-EM of Ribosomal 80S Complexes with Termination Factors Reveals the Translocated Cricket Paralysis Virus IRES. *Molecular Cell*, 57(3):422–432, feb 2015.

[32] Hasan Demirci, Frank Murphy, Eileen Murphy, Steven T. Gregory, Albert E. Dahlberg, and Gerwald Jogl. A structural basis for streptomycin-induced misreading of the genetic code. *Nature Communications*, 4(1), jan 2013.

[33] William A. Cantara, Frank V. Murphy, Hasan Demirci, and Paul F. Agris. Expanded use of sense codons is regulated by modified cytidines in tRNA. *Proceedings of the National Academy of Sciences*, 110(27):10964–10969, jun 2013.

[34] Hasan Demirci, Leyi Wang, Frank V. Murphy, Eileen L. Murphy, Jennifer F. Carr, Scott C. Blanchard, Gerwald Jogl, Albert E. Dahlberg, and Steven T. Gregory. The central role of protein S12 in organizing the structure of the decoding site of the ribosome. *RNA*, 19(12):1791–1801, oct 2013.

[35] Israel S. Fernández, Chyan Leong Ng, Ann C. Kelley, Guowei Wu, Yi-Tao Yu, and V. Ramakrishnan. Unusual base pairing during the decoding of a stop codon by the ribosome. *Nature*, 500(7460):107–110, jun 2013.

[36] David S. Tourigny, Israel S. Fernández, Ann C. Kelley, Ramkrishna Reddy Vakiti, Amit Kumar Chattopadhyay, Stéphane Dorich, Stephen Hanessian, and V. Ramakrishnan. Crystal Structure of a Bioactive Pactamycin Analog Bound to the 30S Ribosomal Subunit. *Journal of Molecular Biology*, 425(20):3907–3910, oct 2013.

[37] Ivan B. Lomakin and Thomas A. Steitz. The initiation of mammalian protein synthesis and mRNA scanning mechanism. *Nature*, 500(7462):307–311, jul 2013.

[38] Jack A. Dunkle, Kellie Vinal, Pooja M. Desai, Natalia Zelinskaya, Miloje Savic, Dayne M. West, Graeme L. Conn, and Christine M. Dunham. Molecular recognition and modification of the 30S ribosome by the aminoglycoside-resistance methyltransferase NpmA. *Proceedings of the National Academy of Sciences*, 111(17):6275–6280, apr 2014.

[39] Christopher H S Aylett, Daniel Boehringer, Jan P Erzberger, Tanja Schaefer, and Nenad Ban. Structure of a Yeast 40S–eIF1–eIF1A–eIF3–eIF3j initiation complex. *Nature Structural & Molecular Biology*, 22(3):269–271, feb 2015.

[40] Julius Rabl, Marc Leibundgut, Sandro F. Ataide, Andrea Haag, and Nenad Ban. Crystal Structure of the Eukaryotic 40 S Ribosomal Subunit in Complex with Initiation Factor 1. *Science*, 331(6018):730–736, feb 2011.

[41] Yaser Hashem, Amedee des Georges, Jie Fu, Sarah N. Buss, Fabrice Jossinet, Amy Jobe, Qin Zhang, Hstau Y. Liao, Robert A. Grassucci, Chandrajit Bajaj, Eric Westhof, Susan Madison-Antenucci, and Joachim Frank. High-resolution cryo-electron microscopy structure of the Trypanosoma brucei ribosome. *Nature*, 494(7437):385–389, feb 2013.

[42] Israel S. Fernández, Xiao-Chen Bai, Garib Murshudov, Sjors H.W. Scheres, and V. Ramakrishnan. Initiation of Translation by Cricket Paralysis Virus IRES Requires Its Translocation in the Ribosome. *Cell*, 157(4):823–831, may 2014.

[43] Junhong Choi, Ka-Weng Ieong, Hasan Demirci, Jin Chen, Alexey Petrov, Arjun Prabhakar, Seán E O'Leary, Dan Dominissini, Gideon Rechavi, S Michael Soltis, Måns Ehrenberg, and Joseph D Puglisi. N6-methyladenosine in mRNA disrupts tRNA selection and translation-elongation dynamics. *Nature Structural & Molecular Biology*, 23(2):110–115, jan 2016.

[44] Andreas Schedlbauer, Tatsuya Kaminishi, Borja Ochoa-Lizarralde, Neha Dhimole, Shu Zhou, Jorge P. López-Alonso, Sean R. Connell, and Paola Fucini. Structural Characterization of an Alternative Mode of Tigecycline Binding to the Bacterial Ribosome. *Antimicrobial Agents and Chemotherapy*, 59(5):2849–2854, may 2015.

[45] Marc A. Schureck, Tatsuya Maehigashi, Stacey J. Miles, Jhomar Marquez, and Christine M. Dunham. mRNA bound to the 30S subunit is a HigB toxin substrate. *RNA*, 22(8):1261–1270, jun 2016.

[46] Nick Quade, Daniel Boehringer, Marc Leibundgut, Joop van den Heuvel, and Nenad Ban. Cryo-EM structure of Hepatitis C virus IRES bound to the human ribosome at 3.9-Å resolution. *Nature Communications*, 6(1), jul 2015.

[47] Basil J. Greber, Philipp Bieri, Marc Leibundgut, Alexander Leitner, Ruedi Aebersold, Daniel Boehringer, and Nenad Ban. The complete structure of the 55 S mammalian mitochondrial ribosome. *Science*, 348(6232):303–308, apr 2015.

[48] Hiroshi Yamamoto, Marianne Collier, Justus Loerke, Jochen Ismer, Andrea Schmidt, Tarek Hilal, Thiemo Sprink, Kaori Yamamoto, Thorsten Mielke, Jörg Bürger, Tanvir R Shaikh, Marylena Dabrowski, Peter W Hildebrand, Patrick Scheerer, and Christian MT Spahn. Molecular architecture of the ribosome-bound Hepatitis C Virus internal ribosomal entry site RNA. *The EMBO Journal*, 34(24):3042–3058, nov 2015.

[49] Jason Murray, Christos G Savva, Byung-Sik Shin, Thomas E Dever, V Ramakrishnan, and Israel S Fernández. Structural characterization of ribosome recruitment and translocation by type IV IRES. *eLife*, 5, may 2016.

[50] Attilio Fabbretti, Andreas Schedlbauer, Letizia Brandi, Tatsuya Kaminishi, Anna Maria Giuliodori, Raffaella Garofalo, Borja Ochoa-Lizarralde, Chie Takemoto, Shigeyuki Yokoyama, Sean R. Connell, Claudio O. Gualerzi, and Paola Fucini. Inhibition of translation initiation complex formation by GE81112 unravels a 16S rRNA structural switch involved in P-site decoding. *Proceedings of the National Academy of Sciences*, 113(16), apr 2016.

[51] Pierre-Damien Coureux, Christine Lazennec-Schurdevin, Auriane Monestier, Eric Larquet, Lionel Cladière, Bruno P. Klaholz, Emmanuelle Schmitt, and Yves Mechulam. Cryo-EM study of start codon selection during archaeal translation initiation. *Nature Communications*, 7(1), nov 2016.

[52] Angelita Simonetti, Jailson Brito Querido, Alexander G. Myasnikov, Eder Mancera-Martinez, Adeline Renaud, Lauriane Kuhn, and Yaser Hashem. eIF3 Peripheral Subunits Rearrangement after mRNA Binding and Start-Codon Recognition. *Molecular Cell*, 63(2):206–217, jul 2016.

[53] Tanweer Hussain, Jose L. Llácer, Brian T. Wimberly, Jeffrey S. Kieft, and V. Ramakrishnan. Large-Scale Movements of IF3 and tRNA during Bacterial Translation Initiation. *Cell*, 167(1):133–144.e13, sep 2016.

[54] Jorge P. López-Alonso, Attilio Fabbretti, Tatsuya Kaminishi, Idoia Iturrioz, Letizia Brandi, David Gil-Carton, Claudio O. Gualerzi, Paola Fucini, and Sean R. Connell. Structure of a 30S pre-initiation complex stalled by GE81112 reveals structural parallels in bacterial and eukaryotic protein synthesis initiation pathways. *Nucleic Acids Research*, page gkw1251, dec 2017.

[55] Philipp Bieri, Marc Leibundgut, Martin Saurer, Daniel Boehringer, and Nenad Ban. The complete structure of the chloroplast 70S ribosome in complex with translation factor pY. *The EMBO Journal*, 36(4):475–486, dec 2017.

[56] R. Kohler, R. A. Mooney, D. J. Mills, R. Landick, and P. Cramer. Architecture of a transcribing-translating expressome. *Science*, 356(6334):194–197, apr 2017.

[57] Jorge Pedro López-Alonso, Tatsuya Kaminishi, Takeshi Kikuchi, Yuya Hirata, Idoia Iturrioz, Neha Dhimole, Andreas Schedlbauer, Yoichi Hase, Simon Goto, Daisuke Kurita, Akira Muto, Shu Zhou, Chieko Naoe, Deryck J. Mills, David Gil-Carton, Chie Takemoto, Hyouta Himeno, Paola Fucini, and Sean R. Connell. RsgA couples the maturation state of the 30S ribosomal decoding center to activation of its GTPase pocket. *Nucleic Acids Research*, 45(11):6945–6959, may 2017.

[58] Jendrik Hentschel, Chloe Burnside, Ingrid Mignot, Marc Leibundgut, Daniel Boehringer, and Nenad Ban. The Complete Structure of the Mycobacterium smegmatis 70S Ribosome. *Cell Reports*, 20(1):149–160, jul 2017.

[59] Melanie Weisser, Tanja Schäfer, Marc Leibundgut, Daniel Böhringer, Christopher Herbert Stanley Aylett, and Nenad Ban. Structural and Functional Insights into Human Re-initiation Complexes. *Molecular Cell*, 67(3):447–456.e7, aug 2017.

[60] Jailson Brito Querido, Eder Mancera-Martínez, Quentin Vicens, Anthony Bochler, Johana Chicher, Angelita Simonetti, and Yaser Hashem. The cryo-EM Structure of a Novel 40S Kinetoplastid-Specific Ribosomal Protein. *Structure*, 25(12):1785–1794.e3, dec 2017.

[61] Xing Zhang, Mason Lai, Winston Chang, Iris Yu, Ke Ding, Jan Mrazek, Hwee L. Ng, Otto O. Yang, Dmitri A. Maslov, and Z. Hong Zhou. Structures and stabilization of kinetoplastid-specific split rRNAs revealed by comparing leishmanial and human ribosomes. *Nature Communications*, 7(1), oct 2016.

[62] Aida Razi, Alba Guarné, and Joaquin Ortega. The cryo-EM structure of YjeQ bound to the 30S subunit suggests a fidelity checkpoint function for this protein in ribosome assembly. *Proceedings of the National Academy of Sciences*, 114(17), apr 2017.

[63] Ivan B. Lomakin, Elena A. Stolboushkina, Anand T. Vaidya, Chenguang Zhao, Maria B. Garber, Sergey E. Dmitriev, and Thomas A. Steitz. Crystal Structure of the Human Ribosome in Complex with DENR-MCT-1. *Cell Reports*, 20(3):521–528, jul 2017.

[64] Junhong Choi, Gabriele Indrisiunaite, Hasan DeMirci, Ka-Weng Ieong, Jinfan Wang, Alexey Petrov, Arjun Prabhakar, Gideon Rechavi, Dan Dominissini, Chuan He, Måns Ehrenberg, and Joseph D. Puglisi. 2'-O-methylation in mRNA disrupts tRNA decoding during translation elongation. *Nature Structural & Molecular Biology*, 25(3):208–216, feb 2018.

[65] Tofayel Ahmed, Jian Shi, and Shashi Bhushan. Unique localization of the plastid-specific ribosomal proteins in the chloroplast ribosome small subunit provides mechanistic insights into the chloroplastic translation. *Nucleic Acids Research*, 45(14):8581–8595, jun 2017.

[66] Zhifei Li, Qiang Guo, Lvqin Zheng, Yongsheng Ji, Yi-Ting Xie, De-Hua Lai, Zhao-Rong Lun, Xun Suo, and Ning Gao. Cryo-EM structures of the 80S ribosomes from human parasites Trichomonas vaginalis and Toxoplasma gondii. *Cell Research*, 27(10):1275–1288, aug 2017.

[67] Zhifei Li, Xueliang Ge, Yixiao Zhang, Lvqin Zheng, Suparna Sanyal, and Ning Gao. Cryo-EM structure of Mycobacterium smegmatis ribosome reveals two unidentified ribosomal proteins close to the functional centers. *Protein & Cell*, sep 2018.

[68] Satabdi Mishra, Tofayel Ahmed, Anu Tyagi, Jian Shi, and Shashi Bhushan. Structures of Mycobacterium smegmatis 70S ribosomes in complex with HPF, tmRNA, and P-tRNA. *Scientific Reports*, 8(1), sep 2018.

[69] Gabriel Demo, Aviram Rasouly, Nikita Vasilyev, Vladimir Svetlov, Anna B Loveland, Ruben Diaz-Avalos, Nikolaus Grigorieff, Evgeny Nudler, and Andrei A Korostelev. Structure of RNA polymerase bound to ribosomal 30S subunit. *eLife*, 6, oct 2017.

[70] Moran Shalev-Benami, Yan Zhang, Haim Rozenberg, Yuko Nobe, Masato Taoka, Donna Matzov, Ella Zimmerman, Anat Bashan, Toshiaki Isobe, Charles L. Jaffe, Ada Yonath, and Georgios Skiniotis. Atomic resolution snapshot of Leishmania ribosome inhibition by the aminoglycoside paromomycin. *Nature Communications*, 8(1), nov 2017.

[71] E. Han Dao, Frédéric Poitevin, Raymond G. Sierra, Cornelius Gati, Yashas Rao, Halil Ibrahim Ciftci, Fulya Akşit, Alex McGurk, Trevor Obrinski, Paul Mgbam, Brandon Hayes, Casper De Lichtenberg, Fatima Pardo-Avila, Nicholas Corsepius, Lindsey Zhang, Matthew H. Seaberg, Mark S. Hunter, Mengling Liang, Jason E. Koglin, Soichi Wakatsuki, and Hasan Demirci. Structure of the 30S ribosomal decoding complex at ambient temperature. *RNA*, 24(12):1667–1676, aug 2018.

[72] Mary E O'Sullivan, Frédéric Poitevin, Raymond G Sierra, Cornelius Gati, E Han Dao, Yashas Rao, Fulya Aksit, Halilibrahim Ciftci, Nicholas Corsepius, Robert Greenhouse, Brandon Hayes, Mark S Hunter, Mengling Liang, Alex McGurk, Paul Mbgam, Trevor Obrinsky, Fátima Pardo-Avila, Matthew H Seaberg, Alan G Cheng, Anthony J Ricci, and Hasan DeMirci. Aminoglycoside ribosome interactions reveal novel conformational states at ambient temperature. *Nucleic Acids Research*, 46(18):9793–9804, aug 2018.

[73] Sweta Vangaveti, William A. Cantara, Jessica L. Spears, Hasan DeMirci, Frank V. Murphy, Sri V. Ranganathan, Kathryn L. Sarachan, and Paul F. Agris. A Structural Basis for Restricted Codon Recognition Mediated by 2-thiocytidine in tRNA Containing a Wobble Position Inosine. *Journal of Molecular Biology*, 432(4):913–929, feb 2020.

[74] Yunlong Li, Manjuli R. Sharma, Ravi K. Koripella, Yong Yang, Prem S. Kaushal, Qishan Lin, Joseph T. Wade, Todd A. Gray, Keith M. Derbyshire, Rajendra K. Agrawal, and Anil K. Ojha. Zinc depletion induces ribosome hibernation in mycobacteria. *Proceedings of the National Academy of Sciences*, 115(32):8191–8196, jul 2018.

[75] André Heuer, Emma Thomson, Christian Schmidt, Otto Berninghausen, Thomas Becker, Ed Hurt, and Roland Beckmann. Cryo-EM structure of a late pre-40S ribosomal subunit from Saccharomyces cerevisiae. *eLife*, 6, nov 2017.

[76] Alain Scaiola, Cohue Pe na, Melanie Weisser, Daniel Böhringer, Marc Leibundgut, Purnima Klingauf-Nerurkar, Stefan Gerhardy, Vikram Govind Panse, and Nenad Ban. Structure of a eukaryotic cytoplasmic pre-40S ribosomal subunit. *The EMBO Journal*, 37(7), feb 2018.

[77] Boris Eliseev, Lahari Yeramala, Alexander Leitner, Manikandan Karuppasamy, Etienne Raimondeau, Karine Huard, Elena Alkalaeva, Ruedi Aebersold, and Christiane Schaffitzel. Structure of a human cap-dependent 48S translation pre-initiation complex. *Nucleic Acids Research*, 46(5):2678–2689, feb 2018.

[78] Jose Luis Llácer, Tanweer Hussain, Adesh K Saini, Jagpreet Singh Nanda, Sukhvir Kaur, Yuliya Gordiyenko, Rakesh Kumar, Alan G Hinnebusch, Jon R Lorsch, and V Ramakrishnan. Translational initiation factor eIF5 replaces eIF1 on the 40S ribosomal subunit to promote start-codon recognition. *eLife*, 7, nov 2018.

[79] Michael Ameismeier, Jingdong Cheng, Otto Berninghausen, and Roland Beckmann. Visualizing late states of human 40S ribosomal subunit maturation. *Nature*, 558(7709):249–253, jun 2018.

[80] Eva Kummer, Marc Leibundgut, Oliver Rackham, Richard G. Lee, Daniel Boehringer, Aleksandra Filipovska, and Nenad Ban. Unique features of mammalian mitochondrial translation initiation revealed by cryo-EM. *Nature*, 560(7717):263–267, aug 2018.

[81] Jose L Llácer, Tanweer Hussain, Jinsheng Dong, Laura Villamayor, Yuliya Gordiyenko, and Alan G Hinnebusch. Large-scale movement of eIF3 domains during translation initiation modulate start codon selection. *Nucleic Acids Research*, 49(20):11491–11511, oct 2021.

[82] Wolfgang H. Schmied, Zakir Tnimov, Chayasith Uttamapinant, Christopher D. Rae, Stephen D. Fried, and Jason W. Chin. Controlling orthogonal ribosome subunit interactions enables evolution of new function. *Nature*, 564(7736):444–448, dec 2018.

[83] Ravi K. Koripella, Manjuli R. Sharma, Md. Emdadul Haque, Paul Risteff, Linda L. Spremulli, and Rajendra K. Agrawal. Structure of Human Mitochondrial Translation Initiation Factor 3 Bound to the Small Ribosomal Subunit. *iScience*, 12:76–86, feb 2019.

[84] Aida Razi, Joseph H Davis, Yumeng Hao, Dushyant Jahagirdar, Brett Thurlow, Kaustuv Basu, Nikhil Jain, Josue Gomez-Blanco, Robert A Britton, Javier Vargas, Alba Guarné, Sarah A Woodson, James R Williamson, and Joaquin Ortega. Role of Era in assembly and homeostasis of the ribosomal small subunit. *Nucleic Acids Research*, 47(15):8301–8317, jul 2019.

[85] Ian J Pavelich, Tatsuya Maehigashi, Eric D Hoffer, Ajchareeya Ruangprasert, Stacey J Miles, and Christine M Dunham. Monomeric YoeB toxin retains RNase activity but adopts an obligate dimeric form for thermal stability. *Nucleic Acids Research*, 47(19):10400–10413, sep 2019.

[86] Sandip Kaledhonkar, Ziao Fu, Kelvin Caban, Wen Li, Bo Chen, Ming Sun, Ruben L. Gonzalez, and Joachim Frank. Late steps in bacterial translation initiation visualized using time-resolved cryo-EM. *Nature*, 570(7761):400–404, may 2019.

[87] Francisco Acosta-Reyes, Ritam Neupane, Joachim Frank, and Israel S Fernández. The Israeli acute paralysis virus IRES captures host ribosomes by mimicking a ribosomal state with hybrid tRNAs. *The EMBO Journal*, 38(21), oct 2019.

[88] Valentin Mitterer, Ramtin Shayan, Sébastien Ferreira-Cerca, Guillaume Murat, Tanja Enne, Dana Rinaldi, Sarah Weigl, Hajrija Omanic, Pierre-Emmanuel Gleizes, Dieter Kressler, Celia Plisson-Chastang, and Brigitte Pertschy. Conformational proofreading of distant 40S ribosomal subunit maturation events by a long-range communication mechanism. *Nature Communications*, 10(1), jun 2019.

[89] Anas Khawaja, Yuzuru Itoh, Cristina Remes, Henrik Spåhr, Olessya Yukhnovets, Henning Höfig, Alexey Amunts, and Joanna Rorbach. Distinct pre-initiation steps in human mitochondrial translation. *Nature Communications*, 11(1), jun 2020.

[90] Yehuda Halfon, Alicia Jimenez-Fernandez, Ruggero La Rosa, Rocio Espinosa Portero, Helle Krogh Johansen, Donna Matzov, Zohar Eyal, Anat Bashan, Ella Zimmerman, Matthew Belousoff, Søren Molin, and Ada Yonath. Structure of Pseudomonas aeruginosa ribosomes from an aminoglycoside-resistant clinical isolate. *Proceedings of the National Academy of Sciences*, 116(44):22275–22281, oct 2019.

[91] Pierre-Damien Coureux, Christine Lazennec-Schurdevin, Sophie Bourcier, Yves Mechulam, and Emmanuelle Schmitt. Cryo-EM study of an archaeal 30S initiation complex gives insights into evolution of translation initiation. *Communications Biology*, 3(1), feb 2020.

[92] Elina Nürenberg-Goloub, Hanna Kratzat, Holger Heinemann, Andr
'e Heuer, Peter Kötter, Otto Berninghausen, Thomas Becker, Robert Tamp
'e, and Roland Beckmann. Molecular analysis of the ribosome recycling factor ABCE 1 bound to the 30S post-splitting complex. *The EMBO Journal*, 39(9), feb 2020.

[93] Christopher E. Morgan, Wei Huang, Susan D. Rudin, Derek J. Taylor, James E. Kirby, Robert A. Bonomo, and Edward W. Yu. Cryo-electron Microscopy Structure of the Acinetobacter baumannii 70S Ribosome and Implications for New Antibiotic Development. *mBio*, 11(1), feb 2020.

[94] Ritam Neupane, Vera P Pisareva, Carlos F Rodriguez, Andrey V Pisarev, and Israel S Fernández. A complex IRES at the 5'-UTR of a viral mRNA assembles a functional 48S complex via an uAUG intermediate. *eLife*, 9, apr 2020.

[95] Dushyant Jahagirdar, Vikash Jha, Kaustuv Basu, Josue Gomez-Blanco, Javier Vargas, and Joaquin Ortega. Alternative conformations and motions adopted by 30S ribosomal subunits visualized by cryo-electron microscopy. *RNA*, 26(12):2017–2030, sep 2020.

[96] Jay Rai, Melissa D. Parker, Haina Huang, Stefan Choy, Homa Ghalei, Matthew C. Johnson, Katrin Karbstein, and M. Elizabeth Stroupe. An open interface in the pre-80S ribosome coordinated by ribosome assembly factors Tsr1 and Dim1 enables temporal regulation of Fap7. *RNA*, 27(2):221–233, nov 2021.

[97] Robert S. Washburn, Philipp K. Zuber, Ming Sun, Yaser Hashem, Bingxin Shen, Wen Li, Sho Harvey, Francisco J. Acosta Reyes, Max E. Gottesman, Stefan H. Knauer, and Joachim Frank. Escherichia coli NusG Links the Lead Ribosome with the Transcription Elongation Complex. *iScience*, 23(8):101352, aug 2020.

[98] Ramtin Shayan, Dana Rinaldi, Natacha Larburu, Laura Plassart, Stéphanie Balor, David Bouyssié, Simon Lebaron, Julien Marcoux, Pierre-Emmanuel Gleizes, and Célia Plisson-Chastang. Good Vibrations: Structural Remodeling of Maturing Yeast Pre-40S Ribosomal Particles Followed by Cryo-Electron Microscopy. *Molecules*, 25(5):1125, mar 2020.

[99] Angelita Simonetti, Ewelina Guca, Anthony Bochler, Lauriane Kuhn, and Yaser Hashem. Structural Insights into the Mammalian Late-Stage Initiation Complexes. *Cell Reports*, 31(1):107497, apr 2020.

[100] Yuzuru Itoh, Andreas Naschberger, Narges Mortezaei, Johannes M. Herrmann, and Alexey Amunts. Analysis of translating mitoribosome reveals functional characteristics of translation in mitochondria of fungi. *Nature Communications*, 11(1), oct 2020.

[101] Hanna Kratzat, Timur Mackens-Kiani, Michael Ameismeier, Mia Potocnjak, Jingdong Cheng, Estelle Dacheux, Abdelkader Namane, Otto Berninghausen, Franz Herzog, Micheline Fromont-Racine, Thomas Becker, and Roland Beckmann. A structural inventory of native ribosomal ABCE1-43S pre-initiation complexes. *The EMBO Journal*, 40(1), dec 2021.

[102] Donna Matzov, Masato Taoka, Yuko Nobe, Yoshio Yamauchi, Yehuda Halfon, Nofar Asis, Ella Zimermann, Haim Rozenberg, Anat Bashan, Shashi Bhushan, Toshiaki Isobe, Michael W Gray, Ada Yonath, and Moran Shalev-Benami. Cryo-EM structure of the highly atypical cytoplasmic ribosome of Euglena gracilis. *Nucleic Acids Research*, 48(20):11750–11761, oct 2020.

[103] Matthias Thoms, Robert Buschauer, Michael Ameismeier, Lennart Koepke, Timo Denk, Maximilian Hirschenberger, Hanna Kratzat, Manuel Hayn, Timur Mackens-Kiani, Jingdong Cheng, Jan H. Straub, Christina M. Stürzel, Thomas Fröhlich, Otto Berninghausen, Thomas Becker, Frank Kirchhoff, Konstantin M. J. Sparrer, and Roland Beckmann. Structural basis for translational shutdown and immune evasion by the Nsp1 protein of SARS-CoV-2. *Science*, 369(6508):1249–1255, sep 2020.

[104] Jailson Brito Querido, Masaaki Sokabe, Sebastian Kraatz, Yuliya Gordiyenko, J. Mark Skehel, Christopher S. Fraser, and V. Ramakrishnan. Structure of a human 48 S translational initiation complex. *Science*, 369(6508):1220–1227, sep 2020.

[105] Katharina Schubert, Evangelos D. Karousis, Ahmad Jomaa, Alain Scaiola, Blanca Echeverria, Lukas-Adrian Gurzeler, Marc Leibundgut, Volker Thiel, Oliver Mühlemann, and Nenad Ban. SARS-CoV-2 Nsp1 binds the ribosomal mRNA channel to inhibit translation. *Nature Structural & Molecular Biology*, 27(10):959–966, sep 2020.

[106] Laura Plassart, Ramtin Shayan, Christian Montellese, Dana Rinaldi, Natacha Larburu, Carole Pichereaux, Carine Froment, Simon Lebaron, Marie-Françoise O'Donohue, Ulrike Kutay, Julien Marcoux, Pierre-Emmanuel Gleizes, and Celia Plisson-Chastang. The final step of 40S ribosomal subunit maturation is controlled by a dual key lock. *eLife*, 10, apr 2021.

[107] Niladri K Sinha, Alban Ordureau, Katharina Best, James A Saba, Boris Zinshteyn, Elayanambi Sundaramoorthy, Amit Fulzele, Danielle M Garshott, Timo Denk, Matthias Thoms, Joao A Paulo, J Wade Harper, Eric J Bennett, Roland Beckmann, and Rachel Green. EDF1 coordinates cellular responses to ribosome collisions. *eLife*, 9, aug 2020.

[108] Michael Ameismeier, Ivo Zemp, Jasmin van den Heuvel, Matthias Thoms, Otto Berninghausen, Ulrike Kutay, and Roland Beckmann. Structural basis for the final steps of human 40S ribosome maturation. *Nature*, 587(7835):683–687, nov 2020.

[109] Anthony Bochler, Jailson Brito Querido, Terezie Prilepskaja, Heddy Soufari, Angelita Simonetti, Mayara Lucia Del Cistia, Lauriane Kuhn, Aline Rimoldi Ribeiro, Leoš Shivaya Valášek, and Yaser Hashem. Structural Differences in Translation Initiation between Pathogenic Trypanosomatids and Their Mammalian Hosts. *Cell Reports*, 33(12):108534, dec 2020.

[110] Andreas Schedlbauer, Idoia Iturrioz, Borja Ochoa-Lizarralde, Tammo Diercks, Jorge Pedro López-Alonso, José Luis Lavin, Tatsuya Kaminishi, Retina Çapuni, Neha Dhimole, Elisa de Astigarraga, David Gil-Carton, Paola Fucini, and Sean R. Connell. A conserved rRNA switch is central to decoding site maturation on the small ribosomal subunit. *Science Advances*, 7(23), jun 2021.

[111] Shuai Yuan, Lei Peng, Jonathan J. Park, Yingxia Hu, Swapnil C. Devarkar, Matthew B. Dong, Qi Shen, Shenping Wu, Sidi Chen, Ivan B. Lomakin, and Yong Xiong. Nonstructural Protein 1 of SARS-CoV-2 Is a Potent Pathogenicity Factor Redirecting Host Protein Synthesis Machinery toward Viral RNA. *Molecular Cell*, 80(6):1055–1066.e6, dec 2020.

[112] Ming Shi, Longfei Wang, Pietro Fontana, Setu Vora, Ying Zhang, Tian-Min Fu, Judy Lieberman, and Hao Wu. SARS-CoV-2 Nsp1 suppresses host but not viral translation through a bipartite mechanism. sep 2020.

[113] Margarita Belinite, Iskander Khusainov, Heddy Soufari, Stefano Marzi, Pascale Romby, Marat Yusupov, and Yaser Hashem. Stabilization of Ribosomal RNA of the Small Subunit by Spermidine in Staphylococcus aureus. *Frontiers in Molecular Biosciences*, 8, nov 2021.

[114] Zhemin Zhang, Christopher E. Morgan, Robert A. Bonomo, and Edward W. Yu. Cryo-EM Determination of Eravacycline-Bound Structures of the Ribosome and the Multidrug Efflux Pump AdeJ of Acinetobacter baumannii. *mBio*, 12(3), jun 2021.

[115] Elena M. Maksimova, Alexey P. Korepanov, Olesya V. Kravchenko, Timur N. Baymukhametov, Alexander G. Myasnikov, Konstantin S. Vassilenko, Zhanna A. Afonina, and Elena A. Stolboushkina. RbfA Is Involved in Two Important Stages of 30S Subunit Assembly: Formation of the Central Pseudoknot and Docking of Helix 44 to the Decoding Center. *International Journal of Molecular Sciences*, 22(11):6140, jun 2021.

[116] Liang Xue, Swantje Lenz, Maria Zimmermann-Kogadeeva, Dimitry Tegunov, Patrick Cramer, Peer Bork, Juri Rappsilber, and Julia Mahamid. Visualizing translation dynamics at atomic detail inside a bacterial cell. *Nature*, 610(7930):205–211, sep 2022.

[117] Yuzuru Itoh, Anas Khawaja, Ivan Laptev, Miriam Cipullo, Ilian Atanassov, Petr Sergiev, Joanna Rorbach, and Alexey Amunts. Mechanism of mitoribosomal small subunit biogenesis and preinitiation. *Nature*, 606(7914):603–608, jun 2022.

[118] Disha-Gajanan Hiregange, Andre Rivalta, Tanaya Bose, Elinor Breiner-Goldstein, Sarit Samiya, Giuseppe Cimicata, Liudmila Kulakova, Ella Zimmerman, Anat Bashan, Osnat Herzberg, and Ada Yonath. Cryo-EM structure of the ancient eukaryotic ribosome from the human parasite Giardia lamblia. *Nucleic Acids Research*, 50(3):1770–1782, jan 2022.

[119] Sung-Hui Yi, Valentyn Petrychenko, Jan Erik Schliep, Akanksha Goyal, Andreas Linden, Ashwin Chari, Henning Urlaub, Holger Stark, Marina V Rodnina, Sarah Adio, and Niels Fischer. Conformational rearrangements upon start codon recognition in human 48S translation initiation complex. *Nucleic Acids Research*, 50(9):5282–5298, apr 2022.

[120] Zuben P Brown, Irina S Abaeva, Swastik De, Christopher U T Hellen, Tatyana V Pestova, and Joachim Frank. Molecular architecture of 40S translation initiation complexes on the hepatitis C virus IRES. *The EMBO Journal*, 41(16), jul 2022.

[121] Christopher P. Lapointe, Rosslyn Grosely, Masaaki Sokabe, Carlos Alvarado, Jinfan Wang, Elizabeth Montabana, Nancy Villa, Byung-Sik Shin, Thomas E. Dever, Christopher S. Fraser, Israel S. Fernández, and Joseph D. Puglisi. eIF5B and eIF1A reorient initiator tRNA to allow ribosomal subunit joining. *Nature*, 607(7917):185–190, jun 2022.

[122] Do Soon Kim, Andrew Watkins, Erik Bidstrup, Joongoo Lee, Ved Topkar, Camila Kofman, Kevin J. Schwarz, Yan Liu, Grigore Pintilie, Emily Roney, Rhiju Das, and Michael C. Jewett. Three-dimensional structure-guided evolution of a ribosome with tethered subunits. *Nature Chemical Biology*, 18(9):990–998, jul 2022.

[123] Juhi Singh, Rahul Raina, Kutti R. Vinothkumar, and Ruchi Anand. Decoding the Mechanism of Specific RNA Targeting by Ribosomal Methyltransferases. *ACS Chemical Biology*, 17(4):829–839, mar 2022.

[124] Ramy Kazan, Gabrielle Bourgeois, Christine Lazennec-Schurdevin, Eric Larquet, Yves Mechulam, Pierre-Damien Coureux, and Emmanuelle Schmitt. Role of aIF5B in archaeal translation initiation. *Nucleic Acids Research*, 50(11):6532–6548, jun 2022.
