# Supplementary material for: Ratchet, swivel, tilt and roll: a complete description of subunit rotation in the ribosome
Source: Nucleic Acids Res. 2022 Dec 30;51(2):919–34. doi: 10.1093/nar/gkac1211 (PMC9881166; doi:10.1093/nar/gkac1211)
Supplement: gkac1211_Supplemental_Files [file gkac1211_supplemental_files.zip › AppendixC.pdf]

Appendix C: Ribosomes identified with the RAD method: LSU-only models

Below are tables that provide all 375 LSUs identified, obtained from 369 RCSB accession codes.
For each entry, the organism name, reference, method and resolution were extracted directly from the RCSB database query system using the following protocol:

-*Organism names*: From the query system, each entry has a list of `polymer_entities`. The longest nucleic acid chain was determined through the `entity_poly.rcsb_entity_polymer_type` and `entity_poly.rcsb_sample_sequence_length` values of each polymer in the RCSB entry. In the tables below, the organism name corresponds to the `rcsb_entity_source_organism.[0].ncbi_scientific_name` value for the longest nucleic acid chain. While many ribosome structures have molecular elements from multiple organisms, this protocol was applied, so that the organism name corresponds to that of the LSU rRNA.

-*Experimental details*: For each entry in the RCSB database, the method listed is from the `exptl.[0].method` element, while the resolution listed in the table is `rcsb_entry_info.resolution_combined.[0]`.

-*Mitoribosomes*: Since there is not a specific entry that indicates whether a ribosome is a mitoribosome, the following process was used to identify mitoribosomes: If the `struct.title` element of the RCSB entry contained "mitoribo" or "mitochond" (case-insensitive), then a * is included in the tables to indicate the structure is a mitoribosome.

-*References*: References are given in the following format: Last name of first author (year) *Journal*

The listed citations are obtained from the RCSB query system by checking the array of references given under the `citation` element for the entry. The value of `rcsb_is_primary` was checked for each citation listed. If Yes was found, the reference was used for the table. For space considerations, only one author is listed for each reference. The author listed in each table entry corresponds to the `rcsb_authors.[0]` element. If the journal or year is not vailable in the RCSB system, it is omitted from the table.

Abbreviations: In the table, the following acronyms are used for journal names:

- AAC: Antimicrobial Agents and Chemotherapy
- ACDBC: Acta Crystallogr D Biol Crystallogr
- ACIEE: Angew Chem Int Ed Engl
- JMB: Journal of Molecular Biology
- NAR: Nucleic Acid Research
- NCB: Nature Chemical Biology
- NComm: Nature Communications
- NMb: Nature Microbiology
- NSMB: Nature Structural and Molecular Biology
- PNAS: Proceedings of the National Academy of Sciences USA

Notes: When using VMD v1.9.4 (pre-release alpha version), the RADtool -download option was unable to automatically align the RCSB entries: 4ADX, 5ZZM, 6W6L, 7AZY. 6W6L, 7AZY failed due to an issue with how v1.9.4alpha identifies chains in CIF files. The entries were analyzed using VMD v1.9.3 without issues. 4ADX, 5ZZM only contain P atoms, which required manual assignment of the chains IDs.

Table 1 of 8

PDB		PRUNED	RMSD	EXP DETAILS		ORGANISM		REFERENCE
ID	chain	LSU	LSU	method	res.	name	mito	
1FFK	0	1564	1.19	XRAY	2.4	Haloarcula marismortui		Ban, et al. (2000) *Science*[1]
1J5A	A	2056	1.10	XRAY	3.5	Deinococcus radiodurans		Schlunzen, et al. (2001) *Nature*[2]
1JJ2	0	1562	1.18	XRAY	2.4	Haloarcula marismortui		Klein, et al. (2001) *EMBO J*[3]
1JZX	A	2057	1.09	XRAY	3.1	Deinococcus radiodurans		Schlunzen, et al. (2001) *Nature*[2]
1JZY	A	2060	1.09	XRAY	3.5	Deinococcus radiodurans		Schlunzen, et al. (2001) *Nature*[2]
1JZZ	A	2055	1.09	XRAY	3.8	Deinococcus radiodurans		Schlunzen, et al. (2001) *Nature*[2]
1K01	A	2060	1.09	XRAY	3.5	Deinococcus radiodurans		Schlunzen, et al. (2001) *Nature*[2]
1K73	A	1571	1.18	XRAY	3.0	Haloarcula marismortui		Hansen, et al. (2003) *JMB*[4]
1K8A	A	1591	1.18	XRAY	3.0	Haloarcula marismortui		Hansen, et al. (2002) *Mol Cell*[5]
1K9M	A	1576	1.18	XRAY	3.0	Haloarcula marismortui		Hansen, et al. (2002) *Mol Cell*[5]
1KC8	A	1584	1.18	XRAY	3.0	Haloarcula marismortui		Hansen, et al. (2003) *JMB*[4]
1KD1	A	1573	1.18	XRAY	3.0	Haloarcula marismortui		Hansen, et al. (2002) *Mol Cell*[5]
1KQS	0	1579	1.17	XRAY	3.1	Haloarcula marismortui		Schmeing, et al. (2002) *Nat Struct Biol*[6]
1M1K	A	1574	1.19	XRAY	3.2	Haloarcula marismortui		Hansen, et al. (2002) *Mol Cell*[5]
1M90	A	1571	1.18	XRAY	2.8	Haloarcula marismortui		Hansen, et al. (2002) *PNAS*[7]
1N8R	A	1587	1.18	XRAY	3.0	Haloarcula marismortui		Hansen, et al. (2003) *JMB*[4]
1NJI	A	1580	1.18	XRAY	3.0	Haloarcula marismortui		Hansen, et al. (2003) *JMB*[4]
1NJM	0	1826	1.25	XRAY	3.6	Deinococcus radiodurans		Bashan, et al. (2003) *Mol Cell*[8]
1NJN	0	1812	1.25	XRAY	3.7	Deinococcus radiodurans		Bashan, et al. (2003) *Mol Cell*[8]
1NJO	0	1822	1.25	XRAY	3.7	Deinococcus radiodurans		Bashan, et al. (2003) *Mol Cell*[8]
1NJP	0	1825	1.25	XRAY	3.5	Deinococcus radiodurans		Bashan, et al. (2003) *Mol Cell*[8]
1NKW	0	2000	1.08	XRAY	3.1	Deinococcus radiodurans		Harms, et al. (2001) *Cell*[9]
1NWX	0	1972	1.12	XRAY	3.5	Deinococcus radiodurans		Schlunzen, et al. (2003) *Structure*[10]
1NWY	0	1967	1.12	XRAY	3.3	Deinococcus radiodurans		Schlunzen, et al. (2003) *Structure*[10]
1OND	0	2021	1.07	XRAY	3.4	Deinococcus radiodurans		Berisio, et al. (2003) *Nat Struct Biol*[11]
1P9X	0	1990	1.07	XRAY	3.4	Deinococcus radiodurans		Berisio, et al. (2003) *J Bacteriol*[12]
1Q7Y	A	1586	1.18	XRAY	3.2	Haloarcula marismortui		Hansen, et al. (2002) *PNAS*[7]
1Q81	A	1576	1.17	XRAY	3.0	Haloarcula marismortui		Hansen, et al. (2002) *PNAS*[7]
1Q82	A	1585	1.18	XRAY	3.0	Haloarcula marismortui		Hansen, et al. (2002) *PNAS*[7]
1Q86	A	1569	1.19	XRAY	3.0	Haloarcula marismortui		Hansen, et al. (2002) *PNAS*[7]
1QVF	0	1571	1.19	XRAY	3.1	Haloarcula marismortui		Schmeing, et al. (2003) *RNA*[13]
1QVG	0	1593	1.19	XRAY	2.9	Haloarcula marismortui		Schmeing, et al. (2003) *RNA*[13]
1S72	0	1564	1.18	XRAY	2.4	Haloarcula marismortui		Klein, et al. (2004) *JMB*[14]
1SM1	0	1904	1.13	XRAY	3.4	Deinococcus radiodurans		Harms, et al. (2004) *BMC Biol*[15]
1VQ4	0	1576	1.19	XRAY	2.7	Haloarcula marismortui		Schmeing, et al. (2005) *Mol Cell*[16]
1VQ5	0	1560	1.18	XRAY	2.6	Haloarcula marismortui		Schmeing, et al. (2005) *Mol Cell*[16]
1VQ6	0	1567	1.18	XRAY	2.7	Haloarcula marismortui		Schmeing, et al. (2005) *Nature*[17]
1VQ7	0	1572	1.18	XRAY	2.5	Haloarcula marismortui		Schmeing, et al. (2005) *Nature*[17]
1VQ8	0	1563	1.18	XRAY	2.2	Haloarcula marismortui		Schmeing, et al. (2005) *Mol Cell*[16]
1VQ9	0	1564	1.17	XRAY	2.4	Haloarcula marismortui		Schmeing, et al. (2005) *Mol Cell*[16]
1VQK	0	1560	1.19	XRAY	2.3	Haloarcula marismortui		Schmeing, et al. (2005) *Mol Cell*[16]
1VQL	0	1567	1.18	XRAY	2.3	Haloarcula marismortui		Schmeing, et al. (2005) *Mol Cell*[16]
1VQM	0	1571	1.18	XRAY	2.3	Haloarcula marismortui		Schmeing, et al. (2005) *Mol Cell*[16]
1VQN	0	1560	1.18	XRAY	2.4	Haloarcula marismortui		Schmeing, et al. (2005) *Nature*[17]
1VQO	0	1559	1.19	XRAY	2.2	Haloarcula marismortui		Schmeing, et al. (2005) *Mol Cell*[16]
1VQP	0	1568	1.18	XRAY	2.2	Haloarcula marismortui		Schmeing, et al. (2005) *Mol Cell*[16]
1W2B	0	1564	1.19	XRAY	3.5	Haloarcula marismortui		Ferbitz, et al. (2004) *Nature*[18]
1XBP	0	1974	1.12	XRAY	3.5	Deinococcus radiodurans		Schluenzen, et al. (2004) *Mol Microbiol*[19]
1Y69	0	2091	1.02	XRAY	3.3	Deinococcus radiodurans R1		Wilson, et al. (2005) *EMBO J*[20]
1YHQ	0	1556	1.18	XRAY	2.4	Haloarcula marismortui		Tu, et al. (2005) *Cell*[21]

PDB ID	PDB chain	PRUNED LSU	RMSD LSU	EXP DETAILS method	EXP DETAILS res.	ORGANISM name	ORGANISM mito	REFERENCE
1YI2	0	1565	1.18	XRAY	2.6	Haloarcula marismortui		Tu, et al. (2005) *Cell*[21]
1YIJ	0	1576	1.19	XRAY	2.6	Haloarcula marismortui		Tu, et al. (2005) *Cell*[21]
1YIT	0	1585	1.18	XRAY	2.8	Haloarcula marismortui		TU, et al. (2005) *Cell*[21]
1YJ9	0	1574	1.20	XRAY	2.8	Haloarcula marismortui		Tu, et al. (2005) *Cell*[21]
1YJN	0	1574	1.18	XRAY	3.0	Haloarcula marismortui		Tu, et al. (2005) *Cell*[21]
1YJW	0	1580	1.19	XRAY	2.9	Haloarcula marismortui		Tu, et al. (2005) *Cell*[21]
1Z58	2	2015	1.11	XRAY	3.8	Deinococcus radiodurans		Amit, et al. (2005) *FEBS Lett*[22]
2AAR	0	2117	0.98	XRAY	3.5	Deinococcus radiodurans		Baram, et al. (2005) *PNAS*[23]
2D3O	0	2060	1.04	XRAY	3.4	Deinococcus radiodurans		Schlunzen, et al. (2005) *Structure*[24]
2J28	B	2636	0.63	EM	8.0	E. coli		Halic, et al. (2006) *Nature*[25]
2O43	A	1965	1.11	XRAY	3.6	Deinococcus radiodurans		Pyetan, et al. *To be published*
2O44	A	1894	1.18	XRAY	3.3	Deinococcus radiodurans		Pyetan, et al. *To be published*
2O45	A	2039	1.10	XRAY	3.6	Deinococcus radiodurans		Baram, et al. *To be published*
2OGM	0	2055	1.14	XRAY	3.5	Deinococcus radiodurans		Davidovich, et al. (2007) *PNAS*[26]
2OGN	0	2038	1.07	XRAY	3.6	Deinococcus radiodurans		Davidovich, et al. (2007) *PNAS*[26]
2OGO	0	2068	1.06	XRAY	3.7	Deinococcus radiodurans		Davidovich, et al. (2007) *PNAS*[26]
2OTJ	0	1578	1.18	XRAY	2.9	Haloarcula marismortui		Schroeder, et al. (2007) *JMB*[27]
2OTL	0	1568	1.18	XRAY	2.7	Haloarcula marismortui		Schroeder, et al. (2007) *JMB*[27]
2QA4	0	1550	1.20	XRAY	3.0	Haloarcula marismortui		Kavran, et al. (2007) *JMB*[28]
2QEX	0	1432	1.27	XRAY	2.9	Haloarcula marismortui		Schroeder, et al. (2007) *AAC*[29]
2ZJP	X	2131	1.04	XRAY	3.7	Deinococcus radiodurans		Harms, et al. (2008) *Mol Cell*[30]
2ZJQ	X	2118	1.02	XRAY	3.3	Deinococcus radiodurans		Harms, et al. (2008) *Mol Cell*[30]
2ZJR	X	2124	1.00	XRAY	2.9	Deinococcus radiodurans		Harms, et al. (2008) *Mol Cell*[30]
3BBX	B	520	1.44	EM	10.0	E. coli		Jiang, et al. (2009) *JMB*[31]
3CC2	0	1568	1.19	XRAY	2.4	Haloarcula marismortui		Blaha, et al. (2008) *JMB*[32]
3CC4	0	1577	1.18	XRAY	2.7	Haloarcula marismortui		Blaha, et al. (2008) *JMB*[32]
3CC7	0	1570	1.18	XRAY	2.7	Haloarcula marismortui		Blaha, et al. (2008) *JMB*[32]
3CCE	0	1575	1.18	XRAY	2.8	Haloarcula marismortui		Blaha, et al. (2008) *JMB*[32]
3CCJ	0	1585	1.18	XRAY	3.3	Haloarcula marismortui		Blaha, et al. (2008) *JMB*[32]
3CCL	0	1548	1.19	XRAY	2.9	Haloarcula marismortui		Blaha, et al. (2008) *JMB*[32]
3CCM	0	1551	1.19	XRAY	2.5	Haloarcula marismortui		Blaha, et al. (2008) *JMB*[32]
3CCQ	0	1562	1.18	XRAY	2.9	Haloarcula marismortui		Blaha, et al. (2008) *JMB*[32]
3CCR	0	1566	1.18	XRAY	3.0	Haloarcula marismortui		Blaha, et al. (2008) *JMB*[32]
3CCS	0	1568	1.17	XRAY	3.0	Haloarcula marismortui		Blaha, et al. (2008) *JMB*[32]
3CCU	0	1567	1.17	XRAY	2.8	Haloarcula marismortui		Blaha, et al. (2008) *JMB*[32]
3CCV	0	1574	1.17	XRAY	2.9	Haloarcula marismortui		Blaha, et al. (2008) *JMB*[32]
3CD6	0	1566	1.17	XRAY	2.8	Haloarcula marismortui		Blaha, et al. (2008) *JMB*[32]
3CF5	X	2126	1.00	XRAY	3.3	Deinococcus radiodurans		Harms, et al. (2008) *Mol Cell*[30]
3CMA	0	1567	1.18	XRAY	2.8	Haloarcula marismortui		Simonovic, et al. (2008) *RNA*[33]
3CME	0	1578	1.18	XRAY	3.0	Haloarcula marismortui		Simonovic, et al. (2008) *RNA*[33]
3CPW	0	1554	1.18	XRAY	2.7	Haloarcula marismortui		Ippolito, et al. (2008) *J Med Chem*[34]
3CXC	0	1574	1.18	XRAY	3.0	Haloarcula marismortui		Zhou, et al. (2008) *Bioorg Med Chem Lett*[35]
3DLL	X	2016	1.05	XRAY	3.5	Deinococcus radiodurans		Wilson, et al. (2008) *PNAS*[36]
3FWO	A	1837	1.25	XRAY	3.7	Deinococcus radiodurans R1		Auerbach, et al. (2009) *BioTechnologia*
3G4S	0	1585	1.19	XRAY	3.2	Haloarcula marismortui		Gurel, et al. (2009) *JMB*[37]
3G6E	0	1573	1.17	XRAY	2.7	Haloarcula marismortui		Gurel, et al. (2009) *JMB*[37]
3G71	0	1580	1.18	XRAY	2.9	Haloarcula marismortui		Gurel, et al. (2009) *JMB*[37]
3I55	0	1576	1.17	XRAY	3.1	Haloarcula marismortui ATCC 43049		Gurel, et al. (2009) *AAC*[38]
3I56	0	1560	1.19	XRAY	2.9	Haloarcula marismortui ATCC 43049		Gurel, et al. (2009) *AAC*[38]
3J3V	A	999	1.45	EM	13.3	B. subtilis subsp. subtilis str. 168		Li, et al. (2013) *NAR*[39]

PDB		PRUNED	RMSD	EXP DETAILS		ORGANISM		REFERENCE
ID	chain	LSU	LSU	method	res.	name	mito	
3J3W	A	1116	1.37	EM	10.7	B. subtilis subsp. subtilis str. 168		Li, et al. (2013) *NAR*[39]
3J5L	A	2669	0.62	EM	6.6	E. coli K-12		Arenz, et al. (2014) *NComm*[40]
3J6B	A	1511	1.09	EM	3.2	S. cerevisiae	*	Amunts, et al. (2014) *Science*[41]
3J79	A	1428	1.19	EM	3.2	Plasmodium falciparum 3D7		Wong, et al. (2014) *Elife*[42]
3J7O	5	1410	1.21	EM	3.5	Sus scrofa		Voorhees, et al. (2014) *Cell*[43]
3J7Q	5	1410	1.21	EM	3.5	Sus scrofa		Voorhees, et al. (2014) *Cell*[43]
3J7Y	A	1002	1.02	EM	3.4	Homo sapiens	*	Brown, et al. (2014) *Science*[44]
3J7Z	A	2623	0.72	EM	3.9	E. coli K-12		Arenz, et al. (2014) *Mol Cell*[45]
3J8G	B	2011	1.29	EM	5.0	E. coli K-12		Zhang, et al. (2014) *NAR*[46]
3J92	5	1409	1.21	EM	3.6	Oryctolagus cuniculus		Shao, et al. (2015) *Mol Cell*[47]
3JCS	1	661	1.25	EM	2.8	Leishmania donovani		Shalev-Benami, et al. (2016) *Cell Rep*[48]
3JCS	2	555	1.14	EM	2.8	Leishmania donovani		Shalev-Benami, et al. (2016) *Cell Rep*[48]
3JCT	1	771	1.32	EM	3.1	S. cerevisiae S288C		Wu, et al. (2016) *Nature*[49]
3JQ4	A	2098	1.02	XRAY	3.5	Deinococcus radiodurans R1		Auerbach, et al. (2010) *PNAS*[50]
3OW2	0	1567	1.18	XRAY	2.7	Haloarcula marismortui		N/A *None*
3PIO	X	2098	1.05	XRAY	3.2	Deinococcus radiodurans R1		Belousoff, et al. (2011) *PNAS*[51]
3PIP	X	2099	1.05	XRAY	3.5	Deinococcus radiodurans R1		Belousoff, et al. (2011) *PNAS*[51]
4ADX	0	1559	1.18	EM	6.6	Methanothermobacter thermautotrophicus str. Delta H		Greber, et al. (2012) *JMB*[52]
4CE4	A	852	1.21	EM	4.9	Sus scrofa domesticus	*	Greber, et al. (2014) *Nature*[53]
4CSU	B	1910	1.30	EM	5.5	E. coli K-12		Feng, et al. (2014) *PLoS Biol*[54]
4D5Y	2	1153	1.35	EM	9.0	Oryctolagus cuniculus		Muhs, et al. (2015) *Mol Cell*[55]
4D67	2	1153	1.35	EM	9.0	Oryctolagus cuniculus		Muhs, et al. (2015) *Mol Cell*[55]
4IO9	X	2123	1.00	XRAY	3.2	Deinococcus radiodurans		Magee, et al. (2013) *Bioorg Med Chem Lett*[56]
4IOA	X	2138	1.01	XRAY	3.2	Deinococcus radiodurans		Magee, et al. (2013) *Bioorg Med Chem Lett*[56]
4IOC	X	2095	1.00	XRAY	3.6	Deinococcus radiodurans		Magee, et al. (2013) *Bioorg Med Chem Lett*[56]
4U67	X	2131	1.03	XRAY	3.6	Deinococcus radiodurans R1		Wekselman, et al. (2017) *Structure*[57]
4UY8	A	2623	0.72	EM	3.8	E. coli		Bischoff, et al. (2014) *Cell Rep*[58]
4V19	A	1007	1.05	EM	3.4	Sus scrofa	*	Greber, et al. (2014) *Nature*[59]
4V7F	1	1086	1.25	EM	8.7	S. cerevisiae		Leidig, et al. (2014) *NComm*[60]
4V8P	A1	1318	1.24	XRAY	3.5	Tetrahymena thermophila		Klinge, et al. (2011) *Science*[61]
4V8P	D1	1315	1.24	XRAY	3.5	Tetrahymena thermophila		Klinge, et al. (2011) *Science*[61]
4V8P	F1	1318	1.24	XRAY	3.5	Tetrahymena thermophila		Klinge, et al. (2011) *Science*[61]
4V8P	H1	1315	1.24	XRAY	3.5	Tetrahymena thermophila		Klinge, et al. (2011) *Science*[61]
4V8T	5	1358	1.26	EM	8.1	S. cerevisiae S288C		Greber, et al. (2012) *NSMB*[62]
4V91	1	1353	1.26	EM	3.7	Kluyveromyces lactis		Fernandez, et al. (2014) *Cell*[63]
4V9F	0	1575	1.19	XRAY	2.4	Haloarcula marismortui ATCC 43049		Gabdulkhakov, et al. (2013) *ACDBC*[64]
4WCE	X	2250	0.99	XRAY	3.5	Staph. aureus subsp. aureus NCTC 8325		Eyal, et al. (2015) *PNAS*[65]
4WF9	X	2204	1.08	XRAY	3.4	Staph. aureus subsp. aureus NCTC 8325		Eyal, et al. (2015) *PNAS*[65]
4WFA	X	2244	0.97	XRAY	3.4	Staph. aureus subsp. aureus NCTC 8325		Eyal, et al. (2015) *PNAS*[65]
4WFB	X	2234	1.05	XRAY	3.4	Staph. aureus subsp. aureus NCTC 8325		Eyal, et al. (2015) *PNAS*[65]
4WFN	X	2135	1.02	XRAY	3.5	Deinococcus radiodurans R1		Wekselman, et al. (2017) *Structure*[57]
5ADY	B	2362	1.14	EM	4.5	E. coli K-12		Zhang, et al. (2015) *NSMB*[66]
5AKA	B	2524	0.64	EM	5.7	E. coli K-12		von Loeffelholz, et al. (2015) *PNAS*[67]
5AN9	N	652	1.12	EM	3.3	Dictyostelium discoideum		Weis, et al. (2015) *NSMB*[68]
5ANB	N	639	1.19	EM	4.1	Dictyostelium discoideum		Weis, et al. (2015) *NSMB*[68]
5ANC	N	630	1.20	EM	4.2	Dictyostelium discoideum		Weis, et al. (2015) *NSMB*[68]
5APN	5	1268	1.30	EM	3.9	S. cerevisiae S288C		Greber, et al. (2015) *Cell*[69]
5APO	5	1253	1.31	EM	3.4	S. cerevisiae S288C		Greber, et al. (2016) *Cell*[69]
5DM6	X	2133	0.99	XRAY	2.9	Deinococcus radiodurans		Kaminishi, et al. (2015) *NAR*[70]
5DM7	X	2138	1.01	XRAY	3.0	Deinococcus radiodurans		Kaminishi, et al. (2015) *NAR*[70]

Table 4 of 8

PDB		PRUNED	RMSD	EXP DETAILS		ORGANISM		REFERENCE
ID	chain	LSU	LSU	method	res.	name	mito	
5FL8	x	565	1.48	EM	9.5	S. cerevisiae S288C		Barrio-Garcia, et al. (2016) *NSMB*[71]
5GAD	A	2613	0.72	EM	3.7	E. coli		Jomaa, et al. (2016) *NComm*[72]
5GAE	A	2552	0.83	EM	3.3	E. coli		Jomaa, et al. (2016) *NComm*[72]
5GAF	A	2613	0.72	EM	4.3	E. coli		Jomaa, et al. (2016) *NComm*[72]
5GAG	A	2609	0.75	EM	3.8	E. coli		Jomaa, et al. (2016) *NComm*[72]
5GAH	A	2613	0.72	EM	3.8	E. coli		Jomaa, et al. (2016) *NComm*[72]
5GAK	1	1275	1.31	EM	3.9	S. cerevisiae		Schmidt, et al. (2016) *NAR*[73]
5H1S	A	2049	1.05	EM	3.5	Spinacia oleracea		Ahmed, et al. (2016) *Sci Rep*[74]
5H4P	1	1247	1.28	EM	3.1	S. cerevisiae		Ma, et al. (2017) *NSMB*[75]
5HKV	X	2251	0.97	XRAY	3.7	Staph. aureus subsp. aureus NCTC 8325		Matzov, et al. (2017) *NAR*[76]
5HL7	X	2229	1.01	XRAY	3.5	Staph. aureus subsp. aureus NCTC 8325		Eyal, et al. (2016) *Sci Rep*[77]
5JCS	x	648	1.49	EM	9.5	S. cerevisiae		Barrio-Garcia, et al. (2016) *NSMB*[71]
5JVG	X	2121	1.05	XRAY	3.4	Deinococcus radiodurans R1		Krupkin, et al. (2016) *PNAS*[78]
5JVH	X	2113	0.99	XRAY	3.6	Deinococcus radiodurans R1		Krupkin, et al. (2016) *PNAS*[78]
5MLC	A	2038	1.13	EM	3.9	Spinacia oleracea		Graf, et al. (2017) *NAR*[79]
5MMI	A	2064	1.09	EM	3.2	Spinacia oleracea		Bieri, et al. (2017) *EMBO J*[80]
5NCO	A	2613	0.72	EM	4.8	E. coli		Jomaa, et al. (2017) *NComm*[81]
5NRG	X	2253	0.94	XRAY	3.4	Staph. aureus subsp. aureus NCTC 8325		Matzov, et al. (2017) *NAR*[76]
5O60	A	2289	1.05	EM	3.2	Mycolicibacterium smegmatis MC2 155		Hentschel, et al. (2017) *Cell Rep*[82]
5OOL	A	990	1.03	EM	3.1	Homo sapiens	*	Brown, et al. (2017) *NSMB*[83]
5OOM	A	644	1.14	EM	3.0	Homo sapiens	*	Brown, et al. (2017) *NSMB*[83]
5T5H	A	649	1.27	EM	2.5	Trypanosoma cruzi		Liu, et al. (2016) *PNAS*[84]
5T5H	B	529	1.10	EM	2.5	Trypanosoma cruzi		Liu, et al. (2016) *PNAS*[84]
5T62	A	892	1.40	EM	3.3	S. cerevisiae S288C		Malyutin, et al. (2017) *EMBO J*[85]
5T6R	A	955	1.41	EM	4.5	S. cerevisiae		Malyutin, et al. (2017) *EMBO J*[85]
5UMD	A	1440	1.20	EM	3.2	Plasmodium falciparum 3D7		Wong, et al. (2017) *NMb*[86]
5V7Q	A	2241	1.15	EM	3.7	Mycobacterium tuberculosis		Yang, et al. (2017) *NAR*[87]
5X8T	A	2103	1.03	EM	3.3	Spinacia oleracea		Ahmed, et al. (2017) *NAR*[88]
5XXB	1	1243	1.29	EM	3.2	Toxoplasma gondii		Li, et al. (2017) *Cell Res*[89]
5XY3	1	1197	1.30	EM	3.2	Trichomonas vaginalis		Li, et al. (2017) *Cell Res*[89]
5XYM	A	2133	1.16	EM	3.1	Mycolicibacterium smegmatis MC2 155		Li, et al. (2018) *Protein Cell*[90]
5Z3G	A	373	1.29	EM	3.6	S. cerevisiae		Zhou, et al. (2019) *Protein Cell*[91]
5ZET	A	2346	0.97	EM	3.2	Mycolicibacterium smegmatis MC2 155		Mishra, et al. (2018) *Sci Rep*[92]
5ZZM	N	292	1.48	EM	8.1	E. coli		Dey, et al. (2018) *J Cell Biol*[93]
6AZ3	1	638	1.27	EM	2.5	Leishmania donovani		Shalev-Benami, et al. (2017) *NComm*[94]
6AZ3	2	611	1.10	EM	2.5	Leishmania donovani		Shalev-Benami, et al. (2017) *NComm*[94]
6C0F	1	325	1.25	EM	3.7	S. cerevisiae BY4741		Sanghai, et al. (2018) *Nature*[95]
6C4H	A	618	0.48	EM	3.1	E. coli		Zeng, et al. (2018) *Sci Rep*[96]
6CB1	1	326	1.26	EM	4.6	S. cerevisiae BY4741		Sanghai, et al. (2018) *Nature*[95]
6DDD	1	2226	1.10	EM	3.1	Staph. aureus		Belousoff, et al. (2019) *ChemMedChem*[97]
6DDG	1	2256	1.08	EM	3.1	Staph. aureus		Belousoff, et al. (2019) *ChemMedChem*[97]
6DZP	A	2176	1.18	EM	3.4	Mycolicibacterium smegmatis MC2 155		Li, et al. (2018) *PNAS*[98]
6ELZ	1	536	1.30	EM	3.3	S. cerevisiae S288C		Kater, et al. (2017) *Cell*[99]
6EM1	1	358	1.21	EM	3.6	S. cerevisiae S288C		Kater, et al. (2017) *Cell*[99]
6EM3	1	364	1.23	EM	3.2	S. cerevisiae S288C		Kater, et al. (2017) *Cell*[99]
6EM4	1	364	1.24	EM	4.1	S. cerevisiae		Kater, et al. (2017) *Cell*[99]
6EM5	1	535	1.30	EM	4.3	S. cerevisiae		Kater, et al. (2017) *Cell*[99]
6FRK	5	1382	1.25	EM	3.7	Oryctolagus cuniculus		Kobayashi, et al. (2018) *Science*[100]
6FT6	1	794	1.36	EM	3.9	S. cerevisiae S288C		Schuller, et al. (2018) *Science*[101]
6FTG	u	1427	1.20	EM	9.1	Oryctolagus cuniculus		Braunger, et al. (2018) *Science*[102]

PDB		PRUNED	RMSD	EXP DETAILS		ORGANISM		REFERENCE
ID	chain	LSU	LSU	method	res.	name	mito	
6FTI	u	1427	1.20	EM	4.2	Oryctolagus cuniculus		Braunger, et al. (2018) *Science*[102]
6FTJ	u	1427	1.20	EM	4.7	Oryctolagus cuniculus		Braunger, et al. (2018) *Science*[102]
6GB2	BA	1054	1.00	EM	3.2	Sus scrofa	*	Kummer, et al. (2018) *Nature*[103]
6GBZ	A	2301	0.92	EM	3.8	E. coli K-12		Nikolay, et al. (2018) *Mol Cell*[104]
6GC0	A	2285	0.92	EM	3.8	E. coli		Nikolay, et al. (2018) *Mol Cell*[104]
6GC4	A	1796	1.16	EM	4.3	E. coli K-12		Nikolay, et al. (2018) *Mol Cell*[104]
6GC6	A	1307	1.27	EM	4.3	E. coli K-12		Nikolay, et al. (2018) *Mol Cell*[104]
6GC7	A	1055	1.31	EM	4.3	E. coli K-12		Nikolay, et al. (2018) *Mol Cell*[104]
6GC8	A	2534	0.97	EM	3.8	E. coli K-12		Nikolay, et al. (2018) *Mol Cell*[104]
6HD7	1	1194	1.35	EM	3.4	S. cerevisiae		Knorr, et al. (2019) *NSMB*[105]
6HMA	A	1134	1.45	EM	2.6	Staph. aureus		Eyal, et al. *To be published*
6I0Y	A	2623	0.72	EM	3.2	E. coli		Tian, et al. (2018) *PNAS*[106]
6I9R	A	997	1.09	EM	3.9	Homo sapiens	*	Sighel, et al. (2021) *Cell Rep*[107]
6LQM	2	1232	1.30	EM	3.1	Homo sapiens		Liang, et al. (2020) *NComm*[108]
6LSR	2	1193	1.30	EM	3.1	Homo sapiens		Liang, et al. (2020) *NComm*[108]
6LSS	2	961	1.31	EM	3.2	Homo sapiens		Liang, et al. (2020) *NComm*[108]
6LU8	2	987	1.32	EM	3.1	Homo sapiens		Liang, et al. (2020) *NComm*[108]
6M62	1	776	1.33	EM	3.2	S. cerevisiae S288C		Micic, et al. (2020) *NComm*[109]
6N8J	1	857	1.38	EM	3.5	S. cerevisiae S288C		Zhou, et al. (2019) *NComm*[110]
6N8K	1	922	1.37	EM	3.6	S. cerevisiae S288C		Zhou, et al. (2019) *NComm*[110]
6N8L	1	987	1.37	EM	3.6	S. cerevisiae S288C		Zhou, et al. (2019) *NComm*[110]
6N8M	A	1047	1.36	EM	3.5	S. cerevisiae S288C		Zhou, et al. (2019) *NComm*[110]
6N8N	A	1025	1.37	EM	3.8	S. cerevisiae S288C		Zhou, et al. (2019) *NComm*[110]
6N8O	A	1061	1.36	EM	3.5	S. cerevisiae S288C		Zhou, et al. (2019) *NComm*[110]
6OIG	5	512	1.48	EM	3.8	S. cerevisiae		Rai, et al. (2021) *RNA*[111]
6PC5	I	2576	0.77	EM	2.7	E. coli		Li, et al. (2020) *Nature*[112]
6PC6	I	2578	0.78	EM	2.5	E. coli		Li, et al. (2020) *Nature*[112]
6PC7	I	2579	0.75	EM	2.5	E. coli		Li, et al. (2020) *Nature*[112]
6PC8	I	2559	0.84	EM	2.9	E. coli		Li, et al. (2020) *Nature*[112]
6PCH	I	2565	0.84	EM	2.9	E. coli		Li, et al. (2020) *Nature*[112]
6PCQ	I	2567	0.79	EM	2.6	E. coli		Li, et al. (2020) *Nature*[112]
6PCR	I	2574	0.77	EM	2.5	E. coli		Li, et al. (2020) *Nature*[112]
6PCS	I	2583	0.78	EM	2.8	E. coli		Li, et al. (2020) *Nature*[112]
6PCT	I	2561	0.80	EM	2.8	E. coli		Li, et al. (2020) *Nature*[112]
6PJ6	I	2670	0.68	EM	2.2	E. coli		Stojkovic, et al. (2020) *NAR*[113]
6PPF	A	1337	1.38	EM	3.4	B. subtilis		Seffouh, et al. (2019) *NAR*[114]
6PPK	A	1985	1.13	EM	4.4	B. subtilis		Seffouh, et al. (2019) *NAR*[114]
6PVK	A	1151	1.37	EM	3.4	B. subtilis		Seffouh, et al. (2019) *NAR*[114]
6QDW	b	2597	0.63	EM	2.8	E. coli BL21(DE3)		Schulte, et al. (2020) *NComm*[115]
6QIK	A	1291	1.29	EM	3.1	S. cerevisiae		Kargas, et al. (2019) *Elife*[116]
6QKL	N	658	1.15	EM	3.3	Dictyostelium discoideum		Weis, et al. (2015) *NSMB*[68]
6QT0	A	1345	1.25	EM	3.4	S. cerevisiae		Kargas, et al. (2019) *Elife*[116]
6QTZ	A	1319	1.29	EM	3.5	S. cerevisiae		Kargas, et al. (2019) *Elife*[116]
6QUL	A	2586	0.74	EM	3.0	E. coli		Scaiola, et al. (2019) *Sci Rep*[117]
6R84	1	1379	1.26	EM	3.6	S. cerevisiae		Su, et al. (2019) *Nature*[118]
6R86	1	1398	1.26	EM	3.4	S. cerevisiae		Su, et al. (2019) *Nature*[118]
6R87	1	1399	1.25	EM	3.4	S. cerevisiae		Su, et al. (2019) *Nature*[118]
6RI5	A	1325	1.27	EM	3.3	S. cerevisiae		Kargas, et al. (2019) *Elife*[116]
6RZZ	A	1306	1.27	EM	3.2	S. cerevisiae		Kargas, et al. (2019) *Elife*[116]
6S05	A	1262	1.29	EM	3.9	S. cerevisiae		Kargas, et al. (2019) *Elife*[116]

PDB ID	PDB chain	PRUNED LSU	RMSD LSU	EXP DETAILS method	EXP DETAILS res.	ORGANISM name	ORGANISM mito	REFERENCE
6S0K	A	2609	0.75	EM	3.1	E. coli		Wang, et al. (2019) *NSMB*[119]
6S0Z	A	1879	1.26	EM	2.3	Staph. aureus		Halfon, et al. (2019) *Sci Rep*[120]
6S12	A	1502	1.35	EM	3.2	Staph. aureus		Halfon, et al. (2019) *Sci Rep*[120]
6SJ6	A	588	1.49	EM	3.2	Staph. aureus subsp. aureus NCTC 8325		Khusainov, et al. (2020) *NComm*[121]
6SPB	A	2456	0.81	EM	2.8	Pseudomonas aeruginosa		Halfon, et al. (2019) *PNAS*[122]
6SPD	A	1259	1.48	EM	3.3	Pseudomonas aeruginosa		Halfon, et al. (2019) *PNAS*[122]
6SWA	q	1311	1.27	EM	3.1	Mus musculus		Kraushar, et al. (2021) *Mol Cell*[123]
6T59	54	1431	1.19	EM	3.1	Oryctolagus cuniculus		Lin, et al. (2020) *Science*[124]
6TNN	U	1235	1.46	EM	3.1	B. subtilis subsp. subtilis str. 168		Oerum, et al. (2020) *Mol Cell*[125]
6TPQ	U	1298	1.45	EM	3.1	B. subtilis subsp. subtilis str. 168		Oerum, et al. (2020) *Mol Cell*[125]
6U48	CA	2713	0.77	EM	2.9	E. coli		Travin, et al. (2019) *NComm*[126]
6V3D	AN1	1358	1.45	EM	3.0	Acinetobacter baumannii AB0057		Morgan, et al. (2020) *mBio*[127]
6W6L	t	1135	1.30	EM	3.8	Homo sapiens		McGilvray, et al. (2020) *Elife*[128]
6WNT	4	2405	0.86	EM	3.1	E. coli		Huang, et al. (2020) *NComm*[129]
6WQN	1	908	1.50	EM	2.9	Staph. aureus		Wright, et al. (2020) *Acs Pharmacol Transl Sci*[130]
6WQQ	1	1099	1.48	EM	3.1	Staph. aureus		Wright, et al. (2020) *Acs Pharmacol Transl Sci*[130]
6WRS	1	942	1.49	EM	3.2	Staph. aureus		Wright, et al. (2020) *Acs Pharmacol Transl Sci*[130]
6WRU	1	1739	1.34	EM	3.1	Staph. aureus		Wright, et al. (2020) *Acs Pharmacol Transl Sci*[130]
6WU9	A	1094	1.47	EM	2.9	Enterococcus faecalis OG1RF		Murphy, et al. (2020) *Sci Rep*[131]
6WYV	I	2540	0.89	EM	2.8	E. coli		Li, et al. (2020) *Nature*[112]
6XZ7	A	2632	0.63	EM	2.1	E. coli K-12		Pichkur, et al. (2020) *RNA*[132]
6Y6X	L5	1415	1.19	EM	2.8	Homo sapiens		Osterman, et al. (2020) *NCB*[133]
6YHS	1	1786	1.36	EM	2.7	Acinetobacter baumannii ATCC 19606 = CIP 70.34 = JCM 6841		Nicholson, et al. (2020) *Structure*[134]
6YLG	1	847	1.35	EM	3.0	S. cerevisiae		Kater, et al. (2020) *Mol Cell*[135]
6YLH	1	847	1.35	EM	3.1	S. cerevisiae		Kater, et al. (2020) *Mol Cell*[135]
6YLX	1	646	1.37	EM	3.9	S. cerevisiae		Kater, et al. (2020) *Mol Cell*[135]
6YLY	1	777	1.37	EM	3.8	S. cerevisiae		Kater, et al. (2020) *Mol Cell*[135]
6YS3	b	2597	0.63	EM	2.6	E. coli BL21(DE3)		Schulte, et al. (2020) *NComm*
6YSI	1	2413	0.89	EM	2.5	Acinetobacter baumannii ATCC 19606 = CIP 70.34 = JCM 6841		Nicholson, et al. (2020) *Structure*[134]
6YWS	A	1355	1.15	EM	2.7	Neurospora crassa OR74A	*	Itoh, et al. (2020) *NComm*[136]
6YWV	A	1109	1.21	EM	3.0	Neurospora crassa OR74A	*	Itoh, et al. (2020) *NComm*[136]
7A0R	X	2118	1.02	XRAY	3.3	Deinococcus radiodurans R1		Breiner-Goldstein, et al. (2021) *NAR*[137]
7A0S	X	2090	1.03	XRAY	3.2	Deinococcus radiodurans R1		Breiner-Goldstein, et al. (2021) *NAR*[137]
7A18	X	2077	1.04	XRAY	3.4	Deinococcus radiodurans R1		Breiner-Goldstein, et al. (2021) *NAR*[137]
7A5H	A	1016	1.01	EM	3.3	Homo sapiens	*	Desai, et al. (2020) *Science*[138]
7A5J	A	996	1.01	EM	3.1	Homo sapiens	*	Desai, et al. (2020) *Science*[138]
7AIH	1	395	1.28	EM	3.6	Leishmania major	*	Soufari, et al. (2020) *PNAS*[139]
7ANE	1	395	1.28	EM	3.9	Leishmania major	*	Soufari, et al. (2020) *PNAS*[139]
7AQC	A	2295	1.03	EM	3.0	B. subtilis subsp. subtilis str. 168		Filbeck, et al. (2021) *Mol Cell*[140]
7AQD	A	2295	1.03	EM	3.1	B. subtilis subsp. subtilis str. 168		Filbeck, et al. (2021) *Mol Cell*[140]
7AS8	A	2320	1.00	EM	2.9	B. subtilis subsp. subtilis str. 168		Crowe-McAuliffe, et al. (2021) *Mol Cell*[141]
7AS9	A	2228	1.09	EM	3.5	B. subtilis subsp. subtilis str. 168		Crowe-McAuliffe, et al. (2021) *Mol Cell*[141]
7ASM	A	1339	1.42	EM	2.5	Staph. aureus		Cimicata, et al. *To be published*
7ASN	A	1101	1.38	EM	2.7	Staph. aureus		Cimicata, et al. *To be published*
7AZY	E	1403	1.23	EM	2.9	S. cerevisiae S288C		Svetlov, et al. (2021) *NComm*[142]
7BHP	L5	1260	1.28	EM	3.3	Homo sapiens		Bhaskar, et al. (2021) *RNA*[143]
7BL2	A	2039	1.06	EM	3.7	E. coli K-12		Nikolay, et al. (2021) *Mol Cell*[144]
7BL3	A	2283	1.02	EM	3.5	E. coli K-12		Nikolay, et al. (2021) *Mol Cell*[144]
7BL4	A	2546	0.83	EM	2.4	E. coli K-12		Nikolay, et al. (2021) *Mol Cell*[144]
7BL5	A	2170	1.05	EM	3.3	E. coli str. K-12 substr. MG1655		Nikolay, et al. (2021) *Mol Cell*[144]

Table 7 of 8

PDB		PRUNED	RMSD	EXP DETAILS		ORGANISM		REFERENCE
ID	chain	LSU	LSU	method	res.	name	mito	
7BL6	A	2521	0.93	EM	4.0	E. coli K-12		Nikolay, et al. (2021) *Mol Cell*[144]
7BT6	1	462	1.47	EM	3.1	S. cerevisiae S288C		Wilson, et al. (2020) *NComm*[145]
7BTB	1	472	1.48	EM	3.2	S. cerevisiae S288C		Wilson, et al. (2020) *NComm*[145]
7BV8	A	2515	0.95	EM	3.1	E. coli K-12		Wang, et al. (2020) *PNAS*[146]
7F0D	A	2197	1.10	EM	3.3	Mycobacterium tuberculosis H37Ra		Zhang, et al. (2022) *Emerg Microbes Infect*[147]
7F5S	L5	1447	1.18	EM	2.7	Homo sapiens		Matsuura-Suzuki, et al. (2022) *Elife*[148]
7L20	A	950	1.12	EM	3.1	Homo sapiens	*	Koripella, et al. (2021) *NComm*[149]
7LVK	I	2673	0.68	EM	2.2	E. coli		Tsai, et al. *To be published*
7M4V	A	1558	1.42	EM	2.5	Acinetobacter baumannii AB0057		Zhang, et al. (2021) *mBio*[150]
7NFX	5	1370	1.25	EM	3.2	Oryctolagus cuniculus		Lee, et al. (2021) *Sci Adv*[151]
7NSH	BA	1027	1.01	EM	3.2	Sus scrofa	*	Kummer, et al. (2021) *Mol Cell*[152]
7O9K	A	751	1.13	EM	3.1	Homo sapiens	*	Cipullo, et al. (2021) *NComm*[153]
7O9M	A	824	1.08	EM	2.5	Homo sapiens	*	Cipullo, et al. (2021) *NComm*[153]
7OBR	5	1279	1.27	EM	2.8	Oryctolagus cuniculus		Jomaa, et al. (2021) *Cell Rep*[154]
7ODE	I	1533	0.98	EM	2.8	E. coli K-12		Larsson, et al. *To be published*
7ODR	A	839	1.08	EM	2.9	Homo sapiens	*	Lenarcic, et al. (2021) *NComm*[155]
7ODS	A	807	1.14	EM	3.1	Homo sapiens	*	Lenarcic, et al. (2021) *NComm*[155]
7ODT	A	919	1.07	EM	3.1	Homo sapiens	*	Lenarcic, et al. (2021) *NComm*[155]
7OF0	A	794	1.10	EM	2.2	Homo sapiens	*	Hillen, et al. (2021) *NComm*[156]
7OF1	1	933	1.33	EM	3.1	S. cerevisiae S288C		Poll, et al. (2021) *PLoS One*[157]
7OF2	A	949	1.04	EM	2.7	Homo sapiens	*	Hillen, et al. (2021) *NComm*[156]
7OF3	A	842	1.09	EM	2.7	Homo sapiens	*	Hillen, et al. (2021) *NComm*[156]
7OF4	A	969	1.05	EM	2.7	Homo sapiens	*	Hillen, et al. (2021) *NComm*[156]
7OF5	A	933	1.05	EM	2.9	Homo sapiens	*	Hillen, et al. (2021) *NComm*[156]
7OF6	A	975	1.05	EM	2.6	Homo sapiens	*	Hillen, et al. (2021) *NComm*[156]
7OF7	A	916	1.06	EM	2.5	Homo sapiens	*	Hillen, et al. (2021) *NComm*[156]
7OH3	1	909	1.35	EM	3.4	S. cerevisiae S288C		Poll, et al. (2021) *PLoS One*[157]
7OHP	1	348	1.30	EM	3.9	S. cerevisiae S288C		Poll, et al. (2021) *PLoS One*[157]
7OHQ	1	911	1.34	EM	3.1	S. cerevisiae S288C		Poll, et al. (2021) *PLoS One*[157]
7OHR	1	479	1.33	EM	4.7	S. cerevisiae S288C		Poll, et al. (2021) *PLoS One*[157]
7OHS	1	316	1.27	EM	4.4	S. cerevisiae S288C		Poll, et al. (2021) *PLoS One*[157]
7OHU	1	369	1.29	EM	3.7	S. cerevisiae S288C		Poll, et al. (2021) *PLoS One*[157]
7OHV	1	399	1.30	EM	3.9	S. cerevisiae S288C		Poll, et al. (2021) *PLoS One*[157]
7OHW	1	315	1.23	EM	3.5	S. cerevisiae S288C		Poll, et al. (2021) *PLoS One*[157]
7OHX	1	329	1.28	EM	3.3	S. cerevisiae S288C		Poll, et al. (2021) *PLoS One*[157]
7OHY	1	382	1.31	EM	3.9	S. cerevisiae S288C		Poll, et al. (2021) *PLoS One*[157]
7OI6	A	397	1.31	EM	5.7	Homo sapiens	*	Cheng, et al. (2021) *NComm*[158]
7OI7	A	593	1.21	EM	3.5	Homo sapiens	*	Cheng, et al. (2021) *NComm*[158]
7OI8	A	646	1.16	EM	3.5	Homo sapiens	*	Cheng, et al. (2021) *NComm*[158]
7OI9	A	642	1.17	EM	3.3	Homo sapiens	*	Cheng, et al. (2021) *NComm*[158]
7OIA	A	642	1.15	EM	3.2	Homo sapiens	*	Cheng, et al. (2021) *NComm*[158]
7OIB	A	640	1.16	EM	3.3	Homo sapiens	*	Cheng, et al. (2021) *NComm*[158]
7OIC	A	938	1.04	EM	3.1	Homo sapiens	*	Cheng, et al. (2021) *NComm*[158]
7OID	A	1006	1.09	EM	3.7	Homo sapiens	*	Cheng, et al. (2021) *NComm*[158]
7OIE	A	1002	1.05	EM	3.5	Homo sapiens	*	Cheng, et al. (2021) *NComm*[158]
7OOD	3	2068	1.16	EM	3.4	Mycoplasma pneumoniae M129		Xue, et al. (2022) *Nature*[159]
7OPE	A	2322	0.99	EM	3.2	B. subtilis subsp. subtilis str. 168		Takada, et al. (2021) *NAR*[160]
7OW7	A	1375	1.20	EM	2.2	Homo sapiens		Faille, et al. *To be published*
7PAT	3	1739	1.30	EM	9.2	Mycoplasma pneumoniae M129		Xue, et al. (2022) *Nature*[159]
7PAU	3	1802	1.30	EM	8.3	Mycoplasma pneumoniae M129		Xue, et al. (2022) *Nature*[159]

Table 8 of 8

PDB		PRUNED	RMSD	EXP DETAILS		ORGANISM		REFERENCE
ID	chain	LSU	LSU	method	res.	name	mito	
7PD3	A	789	1.05	EM	3.4	Homo sapiens	*	Chandrasekaran, et al. (2021) *Elife*[161]
7PKT	7	410	1.20	EM	3.0	Chlamydomonas reinhardtii	*	Waltz, et al. (2021) *NComm*[162]
7PO4	A	574	1.18	EM	2.6	Homo sapiens	*	Itoh, et al. (2022) *Nature*[163]
7PWG	1	895	1.41	EM	2.8	Giardia lamblia ATCC 50803		Hiregange, et al. (2022) *NAR*[164]
7QH6	A	605	1.18	EM	3.1	Homo sapiens	*	Rebelo-Guiomar, et al. (2022) *NComm*[165]
7QH7	A	773	1.14	EM	2.9	Homo sapiens	*	Rebelo-Guiomar, et al. (2022) *NComm*[165]
7QIW	2	1344	1.23	EM	2.4	Solanum lycopersicum		Cottilli, et al. (2022) *Plant Commun*[166]
7QWQ	5	1213	1.31	EM	2.8	Oryctolagus cuniculus		Jomaa, et al. (2022) *Science*[167]
7QWR	5	1191	1.33	EM	2.9	Oryctolagus cuniculus		Jomaa, et al. (2022) *Science*[167]
7QWS	5	1399	1.23	EM	3.4	Oryctolagus cuniculus		Jomaa, et al. (2022) *Science*[167]
7S0S	C	2261	1.05	EM	3.0	Mycolicibacterium smegmatis		Laughlin, et al. (2022) *PNAS*[168]
7S9U	A	735	1.47	EM	3.2	B. subtilis		Seffouh, et al. (2022) *NAR*[169]
7SAE	A	801	1.47	EM	3.0	B. subtilis		Seffouh, et al. (2022) *NAR*[169]
7TM3	K	1412	1.21	EM	3.2	Oryctolagus cuniculus		Smalinskaite, et al. (2022) *Nature*
7TOO	A25S	1174	1.33	EM	2.7	S. cerevisiae		Loveland, et al. (2022) *NComm*[170]
7TOP	A25S	1377	1.27	EM	2.4	S. cerevisiae		Loveland, et al. (2022) *NComm*[170]
7TTU	1	960	1.48	EM	3.0	Staph. aureus		Perlaza-Jimenez, et al. (2022) *Microbiol Spectr*[171]
7TTW	1	905	1.49	EM	2.9	Staph. aureus		Perlaza-Jimenez, et al. (2022) *Microbiol Spectr*[171]
7TUT	K	1397	1.21	EM	3.9	Oryctolagus cuniculus		Smalinskaite, et al. (2022) *Nature*
7Z20	b	2585	0.81	EM	2.3	E. coli		Ahn, et al. (2022) *NComm*[172]
7Z34	1	863	1.37	EM	3.8	S. cerevisiae S288C		Prattes, et al. (2022) *NSMB*[173]
7ZOD	b	2567	0.77	EM	2.6	E. coli		Ahn, et al. (2022) *NComm*[172]
7ZP8	b	2524	0.99	EM	2.2	E. coli		Ahn, et al. (2022) *NComm*[172]
7ZQ5	b	2602	0.67	EM	2.7	E. coli		Ahn, et al. (2022) *NComm*[172]
7ZQ6	b	2584	0.79	EM	2.8	E. coli		Ahn, et al. (2022) *NComm*[172]

References

[1] Nenad Ban, Poul Nissen, Jeffrey Hansen, Peter B. Moore, and Thomas A. Steitz. The Complete Atomic Structure of the Large Ribosomal Subunit at 2.4 Å Resolution. *Science*, 289(5481):905–920, aug 2000.

[2] Frank Schlünzen, Raz Zarivach, Jörg Harms, Anat Bashan, Ante Tocilj, Renate Albrecht, Ada Yonath, and François Franceschi. Structural basis for the interaction of antibiotics with the peptidyl transferase centre in eubacteria. *Nature*, 413(6858):814–821, oct 2001.

[3] D.J. Klein. The kink-turn: a new RNA secondary structure motif. *The EMBO Journal*, 20(15):4214–4221, aug 2001.

[4] Jeffrey L. Hansen, Peter B. Moore, and Thomas A. Steitz. Structures of Five Antibiotics Bound at the Peptidyl Transferase Center of the Large Ribosomal Subunit. *Journal of Molecular Biology*, 330(5):1061–1075, jul 2003.

[5] Jeffrey L. Hansen, Joseph A. Ippolito, Nenad Ban, Poul Nissen, Peter B. Moore, and Thomas A. Steitz. The Structures of Four Macrolide Antibiotics Bound to the Large Ribosomal Subunit. *Molecular Cell*, 10(1):117–128, jul 2002.

[6] T. Martin Schmeing, Amy C. Seila, Jeffrey L. Hansen, Betty Freeborn, Juliane K. Soukup, Stephen A. Scaringe, Scott A. Strobel, Peter B. Moore, and Thomas A. Steitz. A pre-translocational intermediate in protein synthesis observed in crystals of enzymatically active 50S subunits. *Nature Structural Biology*, feb 2002.

[7] Jeffrey L. Hansen, T. Martin Schmeing, Peter B. Moore, and Thomas A. Steitz. Structural insights into peptide bond formation. *Proceedings of the National Academy of Sciences*, 99(18):11670–11675, aug 2002.

[8] Anat Bashan, Ilana Agmon, Raz Zarivach, Frank Schluenzen, Joerg Harms, Rita Berisio, Heike Bartels, Francois Franceschi, Tamar Auerbach, Harly A.S Hansen, Elizaveta Kossoy, Maggie Kessler, and Ada Yonath. Structural Basis of the Ribosomal Machinery for Peptide Bond Formation, Translocation, and Nascent Chain Progression. *Molecular Cell*, 11(1):91–102, jan 2003.

[9] Joerg Harms, Frank Schluenzen, Raz Zarivach, Anat Bashan, Sharon Gat, Ilana Agmon, Heike Bartels, François Franceschi, and Ada Yonath. High Resolution Structure of the Large Ribosomal Subunit from a Mesophilic Eubacterium. *Cell*, 107(5):679–688, nov 2001.

[10] Frank Schlünzen, Jörg M Harms, Francois Franceschi, Harly A.S Hansen, Heike Bartels, Raz Zarivach, and Ada Yonath. Structural Basis for the Antibiotic Activity of Ketolides and Azalides. *Structure*, 11(3):329–338, mar 2003.

[11] Rita Berisio, Frank Schluenzen, Joerg Harms, Anat Bashan, Tamar Auerbach, David Baram, and Ada Yonath. Structural insight into the role of the ribosomal tunnel in cellular regulation. *Nature Structural & Molecular Biology*, 10(5):366–370, mar 2003.

[12] Rita Berisio, Joerg Harms, Frank Schluenzen, Raz Zarivach, Harly A. S. Hansen, Paola Fucini, and Ada Yonath. Structural Insight into the Antibiotic Action of Telithromycin against Resistant Mutants. *Journal of Bacteriology*, 185(14):4276–4279, jul 2003.

[13] T. MARTIN SCHMEING, PETER B. MOORE, and THOMAS A. STEITZ. Structures of deacylated tRNA mimics bound to the E site of the large ribosomal subunit. *RNA*, 9(11):1345–1352, oct 2003.

[14] D.J. Klein, P.B. Moore, and T.A. Steitz. The Roles of Ribosomal Proteins in the Structure Assembly, and Evolution of the Large Ribosomal Subunit. *Journal of Molecular Biology*, 340(1):141–177, jun 2004.

[15] Jörg M Harms, Frank Schlünzen, Paola Fucini, Heike Bartels, and Ada Yonath. *BMC Biology*, 2(1):4, 2004.

[16] T. Martin Schmeing, Kevin S. Huang, David E. Kitchen, Scott A. Strobel, and Thomas A. Steitz. Structural Insights into the Roles of Water and the 2' Hydroxyl of the P Site tRNA in the Peptidyl Transferase Reaction. *Molecular Cell*, 20(3):437–448, nov 2005.

[17] T. Martin Schmeing, Kevin S. Huang, Scott A. Strobel, and Thomas A. Steitz. An induced-fit mechanism to promote peptide bond formation and exclude hydrolysis of peptidyl-tRNA. *Nature*, 438(7067):520–524, nov 2005.

[18] Lars Ferbitz, Timm Maier, Holger Patzelt, Bernd Bukau, Elke Deuerling, and Nenad Ban. Trigger factor in complex with the ribosome forms a molecular cradle for nascent proteins. *Nature*, 431(7008):590–596, aug 2004.

[19] Frank Schlünzen, Erez Pyetan, Paola Fucini, Ada Yonath, and Jörg M. Harms. Inhibition of peptide bond formation by pleuromutilins: the structure of the 50S ribosomal subunit from Deinococcus radiodurans in complex with tiamulin. *Molecular Microbiology*, 54(5):1287–1294, oct 2004.

[20] Daniel N Wilson, Frank Schluenzen, Joerg M Harms, Takuya Yoshida, Tadayasu Ohkubo, Renate Albrecht, Joerg Buerger, Yuji Kobayashi, and Paola Fucini. X-ray crystallography study on ribosome recycling: the mechanism of binding and action of RRF on the 50S ribosomal subunit. *The EMBO Journal*, 24(2):251–260, dec 2005.

[21] Daqi Tu, Gregor Blaha, Peter B. Moore, and Thomas A. Steitz. Structures of MLSBK Antibiotics Bound to Mutated Large Ribosomal Subunits Provide a Structural Explanation for Resistance. *Cell*, 121(2):257–270, apr 2005.

[22] Maya Amit, Rita Berisio, David Baram, Joerg Harms, Anat Bashan, and Ada Yonath. A crevice adjoining the ribosome tunnel: Hints for cotranslational folding. *FEBS Letters*, 579(15):3207–3213, mar 2005.

[23] David Baram, Erez Pyetan, Assa Sittner, Tamar Auerbach-Nevo, Anat Bashan, and Ada Yonath. Structure of trigger factor binding domain in biologically homologous complex with eubacterial ribosome reveals its chaperone action. *Proceedings of the National Academy of Sciences*, 102(34):12017–12022, aug 2005.

[24] Frank Schlünzen, Daniel N. Wilson, Pingsheng Tian, Jörg M. Harms, Stuart J. McInnes, Harly A.S. Hansen, Renate Albrecht, Jörg Buerger, Sigurd M. Wilbanks, and Paola Fucini. The Binding Mode of the Trigger Factor on the Ribosome: Implications for Protein Folding and SRP Interaction. *Structure*, 13(11):1685–1694, nov 2005.

[25] Mario Halic, Michael Blau, Thomas Becker, Thorsten Mielke, Martin R. Pool, Klemens Wild, Irmgard Sinning, and Roland Beckmann. Following the signal sequence from ribosomal tunnel exit to signal recognition particle. *Nature*, 444(7118):507–511, oct 2006.

[26] Chen Davidovich, Anat Bashan, Tamar Auerbach-Nevo, Rachel D. Yaggie, Richard R. Gontarek, and Ada Yonath. Induced-fit tightens pleuromutilins binding to ribosomes and remote interactions enable their selectivity. *Proceedings of the National Academy of Sciences*, 104(11):4291–4296, mar 2007.

[27] Susan J. Schroeder, Gregor Blaha, Julian Tirado-Rives, Thomas A. Steitz, and Peter B. Moore. The Structures of Antibiotics Bound to the E Site Region of the 50 S Ribosomal Subunit of Haloarcula marismortui: 13-Deoxytedanolide and Girodazole. *Journal of Molecular Biology*, 367(5):1471–1479, apr 2007.

[28] Jennifer M. Kavran and Thomas A. Steitz. Structure of the Base of the L7/L12 Stalk of the Haloarcula marismortui Large Ribosomal Subunit: Analysis of L11 Movements. *Journal of Molecular Biology*, 371(4):1047–1059, aug 2007.

[29] Susan J. Schroeder, Gregor Blaha, and Peter B. Moore. Negamycin Binds to the Wall of the Nascent Chain Exit Tunnel of the 50S Ribosomal Subunit. *Antimicrobial Agents and Chemotherapy*, 51(12):4462–4465, dec 2007.

[30] Joerg M. Harms, Daniel N. Wilson, Frank Schluenzen, Sean R. Connell, Torsten Stachelhaus, Zaneta Zaborowska, Christian M.T. Spahn, and Paola Fucini. Translational Regulation via L11: Molecular Switches on the Ribosome Turned On and Off by Thiostrepton and Micrococcin. *Molecular Cell*, 30(1):26–38, apr 2008.

[31] Linhua Jiang, Christiane Schaffitzel, Rouven Bingel-Erlenmeyer, Nenad Ban, Philipp Korber, Roman I. Koning, Daniël C. de Geus, Jasper R. Plaisier, and Jan Pieter Abrahams. Recycling of Aborted Ribosomal 50S Subunit-Nascent Chain-tRNA Complexes by the Heat Shock Protein Hsp15. *Journal of Molecular Biology*, 386(5):1357–1367, mar 2009.

[32] Gregor Blaha, Güliz Gürel, Susan J. Schroeder, Peter B. Moore, and Thomas A. Steitz. Mutations Outside the Anisomycin-Binding Site Can Make Ribosomes Drug-Resistant. *Journal of Molecular Biology*, 379(3):505–519, jun 2008.

[33] Miljan Simonović and Thomas A. Steitz. Peptidyl-CCA deacylation on the ribosome promoted by induced fit and the O3'-hydroxyl group of A76 of the unacylated A-site tRNA. *RNA*, 14(11):2372–2378, sep 2008.

[34] Joseph A. Ippolito, Zoltan F. Kanyo, Deping Wang, Francois J. Franceschi, Peter B. Moore, Thomas A. Steitz, and Erin M. Duffy. Crystal Structure of the Oxazolidinone Antibiotic Linezolid Bound to the 50S Ribosomal Subunit. *Journal of Medicinal Chemistry*, 51(12):3353–3356, may 2008.

[35] Jiacheng Zhou, Ashoke Bhattacharjee, Shili Chen, Yi Chen, Erin Duffy, Jay Farmer, Joel Goldberg, Roger Hanselmann, Joseph A. Ippolito, Rongliang Lou, Alia Orbin, Ayomi Oyelere, Joe Salvino, Dane Springer, Jennifer Tran, Deping Wang, Yusheng Wu, and Graham Johnson. Design at the atomic level: Design of biaryloxazolidinones as potent orally active antibiotics. *Bioorganic & Medicinal Chemistry Letters*, 18(23):6175–6178, dec 2008.

[36] Daniel N. Wilson, Frank Schluenzen, Joerg M. Harms, Agata L. Starosta, Sean R. Connell, and Paola Fucini. The oxazolidinone antibiotics perturb the ribosomal peptidyl-transferase center and effect tRNA positioning. *Proceedings of the National Academy of Sciences*, 105(36):13339–13344, sep 2008.

[37] Güliz Gürel, Gregor Blaha, Peter B. Moore, and Thomas A. Steitz. U2504 Determines the Species Specificity of the A-Site Cleft Antibiotics:. *Journal of Molecular Biology*, 389(1):146–156, may 2009.

[38] Güliz Gürel, Gregor Blaha, Thomas A. Steitz, and Peter B. Moore. Structures of Triacetyloleandomycin and Mycalamide A Bind to the Large Ribosomal Subunit of Haloarcula marismortui. *Antimicrobial Agents and Chemotherapy*, 53(12):5010–5014, dec 2009.

[39] N. Li, Y. Chen, Q. Guo, Y. Zhang, Y. Yuan, C. Ma, H. Deng, J. Lei, and N. Gao. Cryo-EM structures of the late-stage assembly intermediates of the bacterial 50S ribosomal subunit. *Nucleic Acids Research*, 41(14):7073–7083, may 2013.

[40] Stefan Arenz, Haripriya Ramu, Pulkit Gupta, Otto Berninghausen, Roland Beckmann, Nora Vázquez-Laslop, Alexander S. Mankin, and Daniel N. Wilson. Molecular basis for erythromycin-dependent ribosome stalling during translation of the ErmBL leader peptide. *Nature Communications*, 5(1), mar 2014.

[41] Alexey Amunts, Alan Brown, Xiao chen Bai, Jose L. Llácer, Tanweer Hussain, Paul Emsley, Fei Long, Garib Murshudov, Sjors H. W. Scheres, and V. Ramakrishnan. Structure of the Yeast Mitochondrial Large Ribosomal Subunit. *Science*, 343(6178):1485–1489, mar 2014.

[42] Wilson Wong, Xiao chen Bai, Alan Brown, Israel S Fernandez, Eric Hanssen, Melanie Condron, Yan Hong Tan, Jake Baum, and Sjors HW Scheres. Cryo-EM structure of the Plasmodium falciparum 80S ribosome bound to the anti-protozoan drug emetine. *eLife*, 3, jun 2014.

[43] Rebecca M. Voorhees, Israel S. Fernández, Sjors H.W. Scheres, and Ramanujan S. Hegde. Structure of the Mammalian Ribosome-Sec61 Complex to 3.4 Å Resolution. *Cell*, 157(7):1632–1643, jun 2014.

[44] Alan Brown, Alexey Amunts, Xiao chen Bai, Yoichiro Sugimoto, Patricia C. Edwards, Garib Murshudov, Sjors H. W. Scheres, and V. Ramakrishnan. Structure of the large ribosomal subunit from human mitochondria. *Science*, 346(6210):718–722, nov 2014.

[45] Stefan Arenz, Sezen Meydan, Agata L. Starosta, Otto Berninghausen, Roland Beckmann, Nora Vázquez-Laslop, and Daniel N. Wilson. Drug Sensing by the Ribosome Induces Translational Arrest via Active Site Perturbation. *Molecular Cell*, 56(3):446–452, nov 2014.

[46] Xiaoxiao Zhang, Kaige Yan, Yixiao Zhang, Ningning Li, Chengying Ma, Zhifei Li, Yanqing Zhang, Boya Feng, Jing Liu, Yadong Sun, Yanji Xu, Jianlin Lei, and Ning Gao. Structural insights into the function of a unique tandem GTPase EngA in bacterial ribosome assembly. *Nucleic Acids Research*, 42(21):13430–13439, nov 2014.

[47] Sichen Shao, Alan Brown, Balaji Santhanam, and Ramanujan S. Hegde. Structure and Assembly Pathway of the Ribosome Quality Control Complex. *Molecular Cell*, 57(3):433–444, feb 2015.

[48] Moran Shalev-Benami, Yan Zhang, Donna Matzov, Yehuda Halfon, Arie Zackay, Haim Rozenberg, Ella Zimmerman, Anat Bashan, Charles L. Jaffe, Ada Yonath, and Georgios Skiniotis. 2.8-Å Cryo-EM Structure of the Large Ribosomal Subunit from the Eukaryotic Parasite Leishmania. *Cell Reports*, 16(2):288–294, jul 2016.

[49] Shan Wu, Beril Tutuncuoglu, Kaige Yan, Hailey Brown, Yixiao Zhang, Dan Tan, Michael Gamalinda, Yi Yuan, Zhifei Li, Jelena Jakovljevic, Chengying Ma, Jianlin Lei, Meng-Qiu Dong, John L. Woolford, and Ning Gao. Diverse roles of assembly factors revealed by structures of late nuclear pre-60S ribosomes. *Nature*, 534(7605):133–137, may 2016.

[50] Tamar Auerbach, Inbal Mermershtain, Chen Davidovich, Anat Bashan, Matthew Belousoff, Itai Wekselman, Ella Zimmerman, Liqun Xiong, Dorota Klepacki, Kenji Arakawa, Haruyasu Kinashi, Alexander S. Mankin, and Ada Yonath. The structure of ribosome-lankacidin complex reveals ribosomal sites for synergistic antibiotics. *Proceedings of the National Academy of Sciences*, 107(5):1983–1988, jan 2010.

[51] Matthew J. Belousoff, Tal Shapira, Anat Bashan, Ella Zimmerman, Haim Rozenberg, Kenji Arakawa, Haruyasu Kinashi, and Ada Yonath. Crystal structure of the synergistic antibiotic pair, lankamycin and lankacidin, in complex with the large ribosomal subunit. *Proceedings of the National Academy of Sciences*, 108(7):2717–2722, jan 2011.

[52] Basil J. Greber, Daniel Boehringer, Vlatka Godinic-Mikulcic, Ana Crnkovic, Michael Ibba, Ivana Weygand-Durasevic, and Nenad Ban. Cryo-EM Structure of the Archaeal 50S Ribosomal Subunit in Complex with Initiation Factor 6 and Implications for Ribosome Evolution. *Journal of Molecular Biology*, 418(3-4):145–160, may 2012.

[53] Basil J. Greber, Daniel Boehringer, Alexander Leitner, Philipp Bieri, Felix Voigts-Hoffmann, Jan P. Erzberger, Marc Leibundgut, Ruedi Aebersold, and Nenad Ban. Architecture of the large subunit of the mammalian mitochondrial ribosome. *Nature*, 505(7484):515–519, dec 2014.

[54] Boya Feng, Chandra Sekhar Mandava, Qiang Guo, Jie Wang, Wei Cao, Ningning Li, Yixiao Zhang, Yanqing Zhang, Zhixin Wang, Jiawei Wu, Suparna Sanyal, Jianlin Lei, and Ning Gao. Structural and Functional Insights into the Mode of Action of a Universally Conserved Obg GTPase. *PLoS Biology*, 12(5):e1001866, may 2014.

[55] Margarita Muhs, Tarek Hilal, Thorsten Mielke, Maxim A. Skabkin, Karissa Y. Sanbonmatsu, Tatyana V. Pestova, and Christian M.T. Spahn. Cryo-EM of Ribosomal 80S Complexes with Termination Factors Reveals the Translocated Cricket Paralysis Virus IRES. *Molecular Cell*, 57(3):422–432, feb 2015.

[56] Thomas V. Magee, Seungil Han, Sandra P. McCurdy, Thuy-Trinh Nguyen, Karl Granskog, Eric S. Marr, Bruce A. Maguire, Michael D. Huband, Jinshan Michael Chen, Timothy A. Subashi, and Veerabahu Shanmugasundaram. Novel 3-O-carbamoyl erythromycin A derivatives (carbamolides) with activity against resistant staphylococcal and streptococcal isolates. *Bioorganic & Medicinal Chemistry Letters*, 23(6):1727–1731, mar 2013.

[57] Itai Wekselman, Ella Zimmerman, Chen Davidovich, Matthew Belousoff, Donna Matzov, Miri Krupkin, Haim Rozenberg, Anat Bashan, Gilgi Friedlander, Jette Kjeldgaard, Hanne Ingmer, Lasse Lindahl, Janice M. Zengel, and Ada Yonath. The Ribosomal Protein uL22 Modulates the Shape of the Protein Exit Tunnel. *Structure*, 25(8):1233–1241.e3, aug 2017.

[58] Lukas Bischoff, Otto Berninghausen, and Roland Beckmann. Molecular Basis for the Ribosome Functioning as an L-Tryptophan Sensor. *Cell Reports*, 9(2):469–475, oct 2014.

[59] Basil J. Greber, Daniel Boehringer, Marc Leibundgut, Philipp Bieri, Alexander Leitner, Nikolaus Schmitz, Ruedi Aebersold, and Nenad Ban. The complete structure of the large subunit of the mammalian mitochondrial ribosome. *Nature*, 515(7526):283–286, oct 2014.

[60] Christoph Leidig, Matthias Thoms, Iris Holdermann, Bettina Bradatsch, Otto Berninghausen, Gert Bange, Irmgard Sinning, Ed Hurt, and Roland Beckmann. 60S ribosome biogenesis requires rotation of the 5S ribonucleoprotein particle. *Nature Communications*, 5(1), mar 2014.

[61] Sebastian Klinge, Felix Voigts-Hoffmann, Marc Leibundgut, Sofia Arpagaus, and Nenad Ban. Crystal Structure of the Eukaryotic 60 S Ribosomal Subunit in Complex with Initiation Factor 6. *Science*, 334(6058):941–948, nov 2011.

[62] Basil J Greber, Daniel Boehringer, Christian Montellese, and Nenad Ban. Cryo-EM structures of Arx1 and maturation factors Rei1 and Jjj1 bound to the 60S ribosomal subunit. *Nature Structural & Molecular Biology*, 19(12):1228–1233, nov 2012.

[63] Israel S. Fernández, Xiao-Chen Bai, Garib Murshudov, Sjors H.W. Scheres, and V. Ramakrishnan. Initiation of Translation by Cricket Paralysis Virus IRES Requires Its Translocation in the Ribosome. *Cell*, 157(4):823–831, may 2014.

[64] Azat Gabdulkhakov, Stanislav Nikonov, and Maria Garber. Revisiting theHaloarcula marismortui50S ribosomal subunit model. *Acta Crystallographica Section D Biological Crystallography*, 69(6):997–1004, may 2013.

[65] Zohar Eyal, Donna Matzov, Miri Krupkin, Itai Wekselman, Susanne Paukner, Ella Zimmerman, Haim Rozenberg, Anat Bashan, and Ada Yonath. Structural insights into species-specific features of the ribosome from the pathogen Staphylococcus aureus. *Proceedings of the National Academy of Sciences*, 112(43), oct 2015.

[66] Yanqing Zhang, Chandra Sekhar Mandava, Wei Cao, Xiaojing Li, Dejiu Zhang, Ningning Li, Yixiao Zhang, Xiaoxiao Zhang, Yan Qin, Kaixia Mi, Jianlin Lei, Suparna Sanyal, and Ning Gao. HflX is a ribosome-splitting factor rescuing stalled ribosomes under stress conditions. *Nature Structural & Molecular Biology*, 22(11):906–913, oct 2015.

[67] Ottilie von Loeffelholz, Qiyang Jiang, Aileen Ariosa, Manikandan Karuppasamy, Karine Huard, Imre Berger, Shu ou Shan, and Christiane Schaffitzel. Ribosome–SRP–FtsY cotranslational targeting complex in the closed state. *Proceedings of the National Academy of Sciences*, 112(13):3943–3948, mar 2015.

[68] Félix Weis, Emmanuel Giudice, Mark Churcher, Li Jin, Christine Hilcenko, Chi C Wong, David Traynor, Robert R Kay, and Alan J Warren. Mechanism of eIF6 release from the nascent 60S ribosomal subunit. *Nature Structural & Molecular Biology*, 22(11):914–919, oct 2015.

[69] Basil Johannes Greber, Stefan Gerhardy, Alexander Leitner, Marc Leibundgut, Michèle Salem, Daniel Boehringer, Nicolas Leulliot, Ruedi Aebersold, Vikram Govind Panse, and Nenad Ban. Insertion of the Biogenesis Factor Rei1 Probes the Ribosomal Tunnel during 60S Maturation. *Cell*, 164(1-2):91–102, jan 2016.

[70] Tatsuya Kaminishi, Andreas Schedlbauer, Attilio Fabbretti, Letizia Brandi, Borja Ochoa-Lizarralde, Cheng-Guang He, Pohl Milón, Sean R. Connell, Claudio O. Gualerzi, and Paola Fucini. Crystallographic characterization of the ribosomal binding site and molecular mechanism of action of Hygromycin A. *Nucleic Acids Research*, page gkv975, oct 2015.

[71] Clara Barrio-Garcia, Matthias Thoms, Dirk Flemming, Lukas Kater, Otto Berninghausen, Jochen Baßler, Roland Beckmann, and Ed Hurt. Architecture of the Rix1–Rea1 checkpoint machinery during pre-60S-ribosome remodeling. *Nature Structural & Molecular Biology*, 23(1):37–44, nov 2016.

[72] Ahmad Jomaa, Daniel Boehringer, Marc Leibundgut, and Nenad Ban. Structures of the E. coli translating ribosome with SRP and its receptor and with the translocon. *Nature Communications*, 7(1), jan 2016.

[73] Christian Schmidt, Thomas Becker, André Heuer, Katharina Braunger, Vivekanandan Shanmuganathan, Markus Pech, Otto Berninghausen, Daniel N. Wilson, and Roland Beckmann. Structure of the hypusinylated eukaryotic translation factor eIF-5A bound to the ribosome. *Nucleic Acids Research*, 44(4):1944–1951, dec 2016.

[74] Tofayel Ahmed, Zhan Yin, and Shashi Bhushan. Cryo-EM structure of the large subunit of the spinach chloroplast ribosome. *Scientific Reports*, 6(1), oct 2016.

[75] Chengying Ma, Shan Wu, Ningning Li, Yan Chen, Kaige Yan, Zhifei Li, Lvqin Zheng, Jianlin Lei, John L Woolford, and Ning Gao. Structural snapshot of cytoplasmic pre-60S ribosomal particles bound by Nmd3, Lsg1, Tif6 and Reh1. *Nature Structural & Molecular Biology*, 24(3):214–220, jan 2017.

[76] Donna Matzov, Zohar Eyal, Raphael I. Benhamou, Moran Shalev-Benami, Yehuda Halfon, Miri Krupkin, Ella Zimmerman, Haim Rozenberg, Anat Bashan, Micha Fridman, and Ada Yonath. Structural insights of lincosamides targeting the ribosome of Staphylococcus aureus. *Nucleic Acids Research*, 45(17):10284–10292, jul 2017.

[77] Zohar Eyal, Donna Matzov, Miri Krupkin, Susanne Paukner, Rosemarie Riedl, Haim Rozenberg, Ella Zimmerman, Anat Bashan, and Ada Yonath. A novel pleuromutilin antibacterial compound, its binding mode and selectivity mechanism. *Scientific Reports*, 6(1), dec 2016.

[78] Miri Krupkin, Itai Wekselman, Donna Matzov, Zohar Eyal, Yael Diskin Posner, Haim Rozenberg, Ella Zimmerman, Anat Bashan, and Ada Yonath. Avilamycin and evernimicin induce structural changes in rProteins uL16 and CTC that enhance the inhibition of A-site tRNA binding. *Proceedings of the National Academy of Sciences*, 113(44), oct 2016.

[79] Michael Graf, Stefan Arenz, Paul Huter, Alexandra Dönhöfer, Jiř
’ı Nov
’aček, and Daniel N. Wilson. Cryo-EM structure of the spinach chloroplast ribosome reveals the location of plastid-specific ribosomal proteins and extensions. *Nucleic Acids Research*, page gkw1272, dec 2017.

[80] Philipp Bieri, Marc Leibundgut, Martin Saurer, Daniel Boehringer, and Nenad Ban. The complete structure of the chloroplast 70S ribosome in complex with translation factor pY. *The EMBO Journal*, 36(4):475–486, dec 2017.

[81] Ahmad Jomaa, Yu-Hsien Hwang Fu, Daniel Boehringer, Marc Leibundgut, Shu ou Shan, and Nenad Ban. Structure of the quaternary complex between SRP, SR, and translocon bound to the translating ribosome. *Nature Communications*, 8(1), may 2017.

[82] Jendrik Hentschel, Chloe Burnside, Ingrid Mignot, Marc Leibundgut, Daniel Boehringer, and Nenad Ban. The Complete Structure of the Mycobacterium smegmatis 70S Ribosome. *Cell Reports*, 20(1):149–160, jul 2017.

[83] Alan Brown, Sorbhi Rathore, Dari Kimanius, Shintaro Aibara, Xiao chen Bai, Joanna Rorbach, Alexey Amunts, and V Ramakrishnan. Structures of the human mitochondrial ribosome in native states of assembly. *Nature Structural & Molecular Biology*, 24(10):866–869, sep 2017.

[84] Zheng Liu, Cristina Gutierrez-Vargas, Jia Wei, Robert A. Grassucci, Madhumitha Ramesh, Noel Espina, Ming Sun, Beril Tutuncuoglu, Susan Madison-Antenucci, John L. Woolford, Liang Tong, and Joachim Frank. Structure and assembly model for the Trypanosoma cruzi 60S ribosomal subunit. *Proceedings of the National Academy of Sciences*, 113(43):12174–12179, oct 2016.

[85] Andrey G Malyutin, Sharmishtha Musalgaonkar, Stephanie Patchett, Joachim Frank, and Arlen W Johnson. Nmd3 is a structural mimic ofeIF5A, and activates the cpGTPase Lsg1 during 60S ribosome biogenesis. *The EMBO Journal*, 36(7):854–868, feb 2017.

[86] Wilson Wong, Xiao-Chen Bai, Brad E. Sleebs, Tony Triglia, Alan Brown, Jennifer K. Thompson, Katherine E. Jackson, Eric Hanssen, Danushka S. Marapana, Israel S. Fernandez, Stuart A. Ralph, Alan F. Cowman, Sjors H. W. Scheres, and Jake Baum. Mefloquine targets the Plasmodium falciparum 80S ribosome to inhibit protein synthesis. *Nature Microbiology*, 2(6), mar 2017.

[87] Kailu Yang, Jeng-Yih Chang, Zhicheng Cui, Xiaojun Li, Ran Meng, Lijun Duan, Jirapat Thongchol, Joanita Jakana, Christoph M. Huwe, James C. Sacchettini, and Junjie Zhang. Structural insights into species-specific features of the ribosome from the human pathogen Mycobacterium tuberculosis. *Nucleic Acids Research*, 45(18):10884–10894, sep 2017.

[88] Tofayel Ahmed, Jian Shi, and Shashi Bhushan. Unique localization of the plastid-specific ribosomal proteins in the chloroplast ribosome small subunit provides mechanistic insights into the chloroplastic translation. *Nucleic Acids Research*, 45(14):8581–8595, jun 2017.

[89] Zhifei Li, Qiang Guo, Lvqin Zheng, Yongsheng Ji, Yi-Ting Xie, De-Hua Lai, Zhao-Rong Lun, Xun Suo, and Ning Gao. Cryo-EM structures of the 80S ribosomes from human parasites Trichomonas vaginalis and Toxoplasma gondii. *Cell Research*, 27(10):1275–1288, aug 2017.

[90] Zhifei Li, Xueliang Ge, Yixiao Zhang, Lvqin Zheng, Suparna Sanyal, and Ning Gao. Cryo-EM structure of Mycobacterium smegmatis ribosome reveals two unidentified ribosomal proteins close to the functional centers. *Protein & Cell*, sep 2018.

[91] Dejian Zhou, Xing Zhu, Sanduo Zheng, Dan Tan, Meng-Qiu Dong, and Keqiong Ye. Cryo-EM structure of an early precursor of large ribosomal subunit reveals a half-assembled intermediate. *Protein & Cell*, 10(2):120–130, mar 2019.

[92] Satabdi Mishra, Tofayel Ahmed, Anu Tyagi, Jian Shi, and Shashi Bhushan. Structures of Mycobacterium smegmatis 70S ribosomes in complex with HPF, tmRNA, and P-tRNA. *Scientific Reports*, 8(1), sep 2018.

[93] Sandip Dey, Chiranjit Biswas, and Jayati Sengupta. The universally conserved GTPase HflX is an RNA helicase that restores heat-damaged Escherichia coli ribosomes. *Journal of Cell Biology*, 217(7):2519–2529, jun 2018.

[94] Moran Shalev-Benami, Yan Zhang, Haim Rozenberg, Yuko Nobe, Masato Taoka, Donna Matzov, Ella Zimmerman, Anat Bashan, Toshiaki Isobe, Charles L. Jaffe, Ada Yonath, and Georgios Skiniotis. Atomic resolution snapshot of Leishmania ribosome inhibition by the aminoglycoside paromomycin. *Nature Communications*, 8(1), nov 2017.

[95] Zahra Assur Sanghai, Linamarie Miller, Kelly R. Molloy, Jonas Barandun, Mirjam Hunziker, Malik Chaker-Margot, Junjie Wang, Brian T. Chait, and Sebastian Klinge. Modular assembly of the nucleolar pre-60S ribosomal subunit. *Nature*, 556(7699):126–129, mar 2018.

[96] Fuxing Zeng and Hong Jin. Conformation of methylated GGQ in the Peptidyl Transferase Center during Translation Termination. *Scientific Reports*, 8(1), feb 2018.

[97] Matthew J. Belousoff, Hari Venugopal, Alexander Wright, Samuel Seoner, Isabella Stuart, Chris Stubenrauch, Rebecca S. Bamert, David W. Lupton, and Trevor Lithgow. cryoEM-Guided Development of Antibiotics for Drug-Resistant Bacteria. *ChemMedChem*, 14(5):527–531, feb 2019.

[98] Yunlong Li, Manjuli R. Sharma, Ravi K. Koripella, Yong Yang, Prem S. Kaushal, Qishan Lin, Joseph T. Wade, Todd A. Gray, Keith M. Derbyshire, Rajendra K. Agrawal, and Anil K. Ojha. Zinc depletion induces ribosome hibernation in mycobacteria. *Proceedings of the National Academy of Sciences*, 115(32):8191–8196, jul 2018.

[99] Lukas Kater, Matthias Thoms, Clara Barrio-Garcia, Jingdong Cheng, Sherif Ismail, Yasar Luqman Ahmed, Gert Bange, Dieter Kressler, Otto Berninghausen, Irmgard Sinning, Ed Hurt, and Roland Beckmann. Visualizing the Assembly Pathway of Nucleolar Pre-60S Ribosomes. *Cell*, 171(7):1599–1610.e14, dec 2017.

[100] Kan Kobayashi, Ahmad Jomaa, Jae Ho Lee, Sowmya Chandrasekar, Daniel Boehringer, Shu ou Shan, and Nenad Ban. Structure of a prehandover mammalian ribosomal SRP·SRP receptor targeting complex. *Science*, 360(6386):323–327, apr 2018.

[101] Jan Michael Schuller, Sebastian Falk, Lisa Fromm, Ed Hurt, and Elena Conti. Structure of the nuclear exosome captured on a maturing preribosome. *Science*, 360(6385):219–222, apr 2018.

[102] Katharina Braunger, Stefan Pfeffer, Shiteshu Shrimal, Reid Gilmore, Otto Berninghausen, Elisabet C. Mandon, Thomas Becker, Friedrich Förster, and Roland Beckmann. Structural basis for coupling protein transport and N-glycosylation at the mammalian endoplasmic reticulum. *Science*, 360(6385):215–219, apr 2018.

[103] Eva Kummer, Marc Leibundgut, Oliver Rackham, Richard G. Lee, Daniel Boehringer, Aleksandra Filipovska, and Nenad Ban. Unique features of mammalian mitochondrial translation initiation revealed by cryo-EM. *Nature*, 560(7717):263–267, aug 2018.

[104] Rainer Nikolay, Tarek Hilal, Bo Qin, Thorsten Mielke, Jörg Bürger, Justus Loerke, Kathrin Textoris-Taube, Knud H. Nierhaus, and Christian M.T. Spahn. Structural Visualization of the Formation and Activation of the 50S Ribosomal Subunit during In Vitro Reconstitution. *Molecular Cell*, 70(5):881–893.e3, jun 2018.

[105] Alexandra G. Knorr, Christian Schmidt, Petr Tesina, Otto Berninghausen, Thomas Becker, Birgitta Beatrix, and Roland Beckmann. Ribosome–NatA architecture reveals that rRNA expansion segments coordinate N-terminal acetylation. *Nature Structural & Molecular Biology*, 26(1):35–39, dec 2019.

[106] Pengfei Tian, Annette Steward, Renuka Kudva, Ting Su, Patrick J. Shilling, Adrian A. Nickson, Jeffrey J. Hollins, Roland Beckmann, Gunnar von Heijne, Jane Clarke, and Robert B. Best. Folding pathway of an Ig domain is conserved on and off the ribosome. *Proceedings of the National Academy of Sciences*, 115(48), nov 2018.

[107] Denise Sighel, Michela Notarangelo, Shintaro Aibara, Angela Re, Gianluca Ricci, Marianna Guida, Alessia Soldano, Valentina Adami, Chiara Ambrosini, Francesca Broso, Emanuele Filiberto Rosatti, Sara Longhi, Mariachiara Buccarelli, Quintino G. D'Alessandris, Stefano Giannetti, Simone Pacioni, Lucia Ricci-Vitiani, Joanna Rorbach, Roberto Pallini, Sandrine Roulland, Alexey Amunts, Ines Mancini, Angelika Modelska, and Alessandro Quattrone. Inhibition of mitochondrial translation suppresses glioblastoma stem cell growth. *Cell Reports*, 35(4):109024, apr 2021.

[108] Xiaomeng Liang, Mei-Qing Zuo, Yunyang Zhang, Ningning Li, Chengying Ma, Meng-Qiu Dong, and Ning Gao. Structural snapshots of human pre-60S ribosomal particles before and after nuclear export. *Nature Communications*, 11(1), jul 2020.

[109] Jelena Micic, Yu Li, Shan Wu, Daniel Wilson, Beril Tutuncuoglu, Ning Gao, and John L. Woolford. Coupling of 5S RNP rotation with maturation of functional centers during large ribosomal subunit assembly. *Nature Communications*, 11(1), jul 2020.

[110] Yi Zhou, Sharmishtha Musalgaonkar, Arlen W. Johnson, and David W. Taylor. Tightly-orchestrated rearrangements govern catalytic center assembly of the ribosome. *Nature Communications*, 10(1), feb 2019.

[111] Jay Rai, Melissa D. Parker, Haina Huang, Stefan Choy, Homa Ghalei, Matthew C. Johnson, Katrin Karbstein, and M. Elizabeth Stroupe. An open interface in the pre-80S ribosome coordinated by ribosome assembly factors Tsr1 and Dim1 enables temporal regulation of Fap7. *RNA*, 27(2):221–233, nov 2021.

[112] Qi Li, Jenna Pellegrino, D. John Lee, Arthur A. Tran, Hector A. Chaires, Ruoxi Wang, Jesslyn E. Park, Kaijie Ji, David Chow, Na Zhang, Axel F. Brilot, Justin T. Biel, Gydo van Zundert, Kenneth Borrelli, Dean Shinabarger, Cindy Wolfe, Beverly Murray, Matthew P. Jacobson, Estelle Mühle, Olivier Chesneau, James S. Fraser, and Ian B. Seiple. Synthetic group A streptogramin antibiotics that overcome Vat resistance. *Nature*, 586(7827):145–150, sep 2020.

[113] Vanja Stojković, Alexander G Myasnikov, Iris D Young, Adam Frost, James S Fraser, and Danica Galonić Fujimori. Assessment of the nucleotide modifications in the high-resolution cryo-electron microscopy structure of the Escherichia coli 50S subunit. *Nucleic Acids Research*, 48(5):2723–2732, jan 2020.

[114] Amal Seffouh, Nikhil Jain, Dushyant Jahagirdar, Kaustuv Basu, Aida Razi, Xiaodan Ni, Alba Guarné, Robert A Britton, and Joaquin Ortega. Structural consequences of the interaction of RbgA with a 50S ribosomal subunit assembly intermediate. *Nucleic Acids Research*, 47(19):10414–10425, sep 2019.

[115] Linda Schulte, Jiafei Mao, Julian Reitz, Sridhar Sreeramulu, Denis Kudlinzki, Victor-Valentin Hodirnau, Jakob Meier-Credo, Krishna Saxena, Florian Buhr, Julian D. Langer, Martin Blackledge, Achilleas S. Frangakis, Clemens Glaubitz, and Harald Schwalbe. Cysteine oxidation and disulfide formation in the ribosomal exit tunnel. *Nature Communications*, 11(1), nov 2020.

[116] Vasileios Kargas, Pablo Castro-Hartmann, Norberto Escudero-Urquijo, Kyle Dent, Christine Hilcenko, Carolin Sailer, Gertrude Zisser, Maria J Marques-Carvalho, Simone Pellegrino, Leszek Wawiórka, Stefan MV Freund, Jane L Wagstaff, Antonina Andreeva, Alexandre Faille, Edwin Chen, Florian Stengel, Helmut Bergler, and Alan John Warren. Mechanism of completion of peptidyltransferase centre assembly in eukaryotes. *eLife*, 8, may 2019.

[117] Alain Scaiola, Marc Leibundgut, Daniel Boehringer, Patrick Caspers, Daniel Bur, Hans H. Locher, Georg Rueedi, and Daniel Ritz. Structural basis of translation inhibition by cadazolid, a novel quinoxolidinone antibiotic. *Scientific Reports*, 9(1), apr 2019.

[118] Ting Su, Toshiaki Izawa, Matthias Thoms, Yui Yamashita, Jingdong Cheng, Otto Berninghausen, F. Ulrich Hartl, Toshifumi Inada, Walter Neupert, and Roland Beckmann. Structure and function of Vms1 and Arb1 in RQC and mitochondrial proteome homeostasis. *Nature*, 570(7762):538–542, jun 2019.

[119] Shuai Wang, Ahmad Jomaa, Mateusz Jaskolowski, Chien-I Yang, Nenad Ban, and Shu ou Shan. The molecular mechanism of cotranslational membrane protein recognition and targeting by SecA. *Nature Structural & Molecular Biology*, 26(10):919–929, sep 2019.

[120] Yehuda Halfon, Donna Matzov, Zohar Eyal, Anat Bashan, Ella Zimmerman, Jette Kjeldgaard, Hanne Ingmer, and Ada Yonath. Exit tunnel modulation as resistance mechanism of S. aureus erythromycin resistant mutant. *Scientific Reports*, 9(1), aug 2019.

[121] Iskander Khusainov, Bulat Fatkhullin, Simone Pellegrino, Aydar Bikmullin, Wen ti Liu, Azat Gabdulkhakov, Amr Al Shebel, Alexander Golubev, Denis Zeyer, Natalie Trachtmann, Georg A. Sprenger, Shamil Validov, Konstantin Usachev, Gulnara Yusupova, and Marat Yusupov. Mechanism of ribosome shutdown by RsfS in Staphylococcus aureus revealed by integrative structural biology approach. *Nature Communications*, 11(1), apr 2020.

[122] Yehuda Halfon, Alicia Jimenez-Fernandez, Ruggero La Rosa, Rocio Espinosa Portero, Helle Krogh Johansen, Donna Matzov, Zohar Eyal, Anat Bashan, Ella Zimmerman, Matthew Belousoff, Søren Molin, and Ada Yonath. Structure of Pseudomonas aeruginosa ribosomes from an aminoglycoside-resistant clinical isolate. *Proceedings of the National Academy of Sciences*, 116(44):22275–22281, oct 2019.

[123] Matthew L. Kraushar, Ferdinand Krupp, Dermot Harnett, Paul Turko, Mateusz C. Ambrozkiewicz, Thiemo Sprink, Koshi Imami, Manuel Günnigmann, Ulrike Zinnall, Carlos H. Vieira-Vieira, Theres Schaub, Agnieszka Münster-Wandowski, Jörg Bürger, Ekaterina Borisova, Hiroshi Yamamoto, Mladen-Roko Rasin, Uwe Ohler, Dieter Beule, Thorsten Mielke, Victor Tarabykin, Markus Landthaler, Günter Kramer, Imre Vida, Matthias Selbach, and Christian M.T. Spahn. Protein Synthesis in the Developing Neocortex at Near-Atomic Resolution Reveals Ebp1-Mediated Neuronal Proteostasis at the 60S Tunnel Exit. *Molecular Cell*, 81(2):304–322.e16, jan 2021.

[124] Zhewang Lin, Ivana Gasic, Viswanathan Chandrasekaran, Niklas Peters, Sichen Shao, Timothy J. Mitchison, and Ramanujan S. Hegde. TTC5 mediates autoregulation of tubulin via mRNA degradation. *Science*, 367(6473):100–104, jan 2020.

[125] Stephanie Oerum, Tom Dendooven, Marjorie Catala, Laetitia Gilet, Clément Dégut, Aude Trinquier, Maxime Bourguet, Pierre Barraud, Sarah Cianferani, Ben F. Luisi, Ciarán Condon, and Carine Tisné. Structures of B. subtilis Maturation RNases Captured on 50S Ribosome with Pre-rRNAs. *Molecular Cell*, 80(2):227–236.e5, oct 2020.

[126] Dmitrii Y. Travin, Zoe L. Watson, Mikhail Metelev, Fred R. Ward, Ilya A. Osterman, Irina M. Khven, Nelli F. Khabibullina, Marina Serebryakova, Peter Mergaert, Yury S. Polikanov, Jamie H. D. Cate, and Konstantin Severinov. Structure of ribosome-bound azole-modified peptide phazolicin rationalizes its species-specific mode of bacterial translation inhibition. *Nature Communications*, 10(1), oct 2019.

[127] Christopher E. Morgan, Wei Huang, Susan D. Rudin, Derek J. Taylor, James E. Kirby, Robert A. Bonomo, and Edward W. Yu. Cryo-electron Microscopy Structure of the Acinetobacter baumannii 70S Ribosome and Implications for New Antibiotic Development. *mBio*, 11(1), feb 2020.

[128] Philip T McGilvray, S Andrei Anghel, Arunkumar Sundaram, Frank Zhong, Michael J Trnka, James R Fuller, Hong Hu, Alma L Burlingame, and Robert J Keenan. An ER translocon for multi-pass membrane protein biogenesis. *eLife*, 9, aug 2020.

[129] Shijie Huang, Nikolay A. Aleksashin, Anna B. Loveland, Dorota Klepacki, Kaspar Reier, Amira Kefi, Teresa Szal, Jaanus Remme, Luc Jaeger, Nora Vázquez-Laslop, Andrei A. Korostelev, and Alexander S. Mankin. Ribosome engineering reveals the importance of 5S rRNA autonomy for ribosome assembly. *Nature Communications*, 11(1), jun 2020.

[130] Alexander Wright, Kieran Deane-Alder, Edward Marschall, Rebecca Bamert, Hari Venugopal, Trevor Lithgow, David W. Lupton, and Matthew J. Belousoff. Characterization of the Core Ribosomal Binding Region for the Oxazolidone Family of Antibiotics Using Cryo-EM. *ACS Pharmacology & Translational Science*, 3(3):425–432, may 2020.

[131] Eileen L. Murphy, Kavindra V. Singh, Bryant Avila, Torsten Kleffmann, Steven T. Gregory, Barbara E. Murray, Kurt L. Krause, Reza Khayat, and Gerwald Jogl. Cryo-electron microscopy structure of the 70S ribosome from Enterococcus faecalis. *Scientific Reports*, 10(1), oct 2020.

[132] Evgeny B. Pichkur, Alena Paleskava, Andrey G. Tereshchenkov, Pavel Kasatsky, Ekaterina S. Komarova, Dmitrii I. Shiriaev, Alexey A. Bogdanov, Olga A. Dontsova, Ilya A. Osterman, Petr V. Sergiev, Yury S. Polikanov, Alexander G. Myasnikov, and Andrey L. Konevega. Insights into the improved macrolide inhibitory activity from the high-resolution cryo-EM structure of dirithromycin bound to the E. coli 70S ribosome. *RNA*, 26(6):715–723, mar 2020.

[133] Ilya A. Osterman, Maximiliane Wieland, Tinashe P. Maviza, Kseniya A. Lashkevich, Dmitrii A. Lukianov, Ekaterina S. Komarova, Yuliya V. Zakalyukina, Robert Buschauer, Dmitrii I. Shiriaev, Semen A. Leyn, Jaime E. Zlamal, Mikhail V. Biryukov, Dmitry A. Skvortsov, Vadim N. Tashlitsky, Vladimir I. Polshakov, Jingdong Cheng, Yury S. Polikanov, Alexey A. Bogdanov, Andrei L. Osterman, Sergey E. Dmitriev, Roland Beckmann, Olga A. Dontsova, Daniel N. Wilson, and Petr V. Sergiev. Tetracenomycin X inhibits translation by binding within the ribosomal exit tunnel. *Nature Chemical Biology*, 16(10):1071–1077, jun 2020.

[134] David Nicholson, Thomas A. Edwards, Alex J. O'Neill, and Neil A. Ranson. Structure of the 70S Ribosome from the Human Pathogen Acinetobacter baumannii in Complex with Clinically Relevant Antibiotics. *Structure*, 28(10):1087–1100.e3, oct 2020.

[135] Lukas Kater, Valentin Mitterer, Matthias Thoms, Jingdong Cheng, Otto Berninghausen, Roland Beckmann, and Ed Hurt. Construction of the Central Protuberance and L1 Stalk during 60S Subunit Biogenesis. *Molecular Cell*, 79(4):615–628.e5, aug 2020.

[136] Yuzuru Itoh, Andreas Naschberger, Narges Mortezaei, Johannes M. Herrmann, and Alexey Amunts. Analysis of translating mitoribosome reveals functional characteristics of translation in mitochondria of fungi. *Nature Communications*, 11(1), oct 2020.

[137] Elinor Breiner-Goldstein, Zohar Eyal, Donna Matzov, Yehuda Halfon, Giuseppe Cimicata, Moti Baum, Assaf Rokney, Analia V Ezernitchi, Andrew N Lowell, Jennifer J Schmidt, Haim Rozenberg, Ella Zimmerman, Anat Bashan, Lea Valinsky, Yojiro Anzai, David H Sherman, and Ada Yonath. Ribosome-binding and anti-microbial studies of the mycinamicins, 16-membered macrolide antibiotics from Micromonospora griseorubida. *Nucleic Acids Research*, 49(16):9560–9573, aug 2021.

[138] Nirupa Desai, Hanting Yang, Viswanathan Chandrasekaran, Razina Kazi, Michal Minczuk, and V. Ramakrishnan. Elongational stalling activates mitoribosome-associated quality control. *Science*, 370(6520):1105–1110, nov 2020.

[139] Heddy Soufari, Florent Waltz, Camila Parrot, Stéphanie Durrieu-Gaillard, Anthony Bochler, Lauriane Kuhn, Marie Sissler, and Yaser Hashem. Structure of the mature kinetoplastids mitoribosome and insights into its large subunit biogenesis. *Proceedings of the National Academy of Sciences*, 117(47):29851–29861, nov 2020.

[140] Sebastian Filbeck, Federico Cerullo, Helge Paternoga, George Tsaprailis, Claudio A.P. Joazeiro, and Stefan Pfeffer. Mimicry of Canonical Translation Elongation Underlies Alanine Tail Synthesis in RQC. *Molecular Cell*, 81(1):104–114.e6, jan 2021.

[141] Caillan Crowe-McAuliffe, Hiraku Takada, Victoriia Murina, Christine Polte, Sergo Kasvandik, Tanel Tenson, Zoya Ignatova, Gemma C. Atkinson, Daniel N. Wilson, and Vasili Hauryliuk. Structural Basis for Bacterial Ribosome-Associated Quality Control by RqcH and RqcP. *Molecular Cell*, 81(1):115–126.e7, jan 2021.

[142] Maxim S. Svetlov, Timm O. Koller, Sezen Meydan, Vaishnavi Shankar, Dorota Klepacki, Norbert Polacek, Nicholas R. Guydosh, Nora Vázquez-Laslop, Daniel N. Wilson, and Alexander S. Mankin. Context-specific action of macrolide antibiotics on the eukaryotic ribosome. *Nature Communications*, 12(1), may 2021.

[143] Varun Bhaskar, Jessica Desogus, Alexandra Graff-Meyer, Andreas D. Schenk, Simone Cavadini, and Jeffrey A. Chao. Dynamic association of human Ebp1 with the ribosome. *RNA*, 27(4):411–419, jan 2021.

[144] Rainer Nikolay, Tarek Hilal, Sabine Schmidt, Bo Qin, David Schwefel, Carlos H. Vieira-Vieira, Thorsten Mielke, Jörg Bürger, Justus Loerke, Kazuaki Amikura, Timo Flügel, Takuya Ueda, Matthias Selbach, Elke Deuerling, and Christian M.T. Spahn. Snapshots of native pre-50S ribosomes reveal a biogenesis factor network and evolutionary specialization. *Molecular Cell*, 81(6):1200–1215.e9, mar 2021.

[145] Daniel M. Wilson, Yu Li, Amber LaPeruta, Michael Gamalinda, Ning Gao, and John L. Woolford. Structural insights into assembly of the ribosomal nascent polypeptide exit tunnel. *Nature Communications*, 11(1), oct 2020.

[146] Wei Wang, Wanqiu Li, Xueliang Ge, Kaige Yan, Chandra Sekhar Mandava, Suparna Sanyal, and Ning Gao. Loss of a single methylation in 23S rRNA delays 50S assembly at multiple late stages and impairs translation initiation and elongation. *Proceedings of the National Academy of Sciences*, 117(27):15609–15619, jun 2020.

[147] Wen Zhang, ZhiFei Li, Yufan Sun, Peng Cui, Jianhua Liang, Qinghe Xing, Jing Wu, Yanhui Xu, Wenhong Zhang, Ying Zhang, Lin He, and Ning Gao. Cryo-EM structure of Mycobacterium tuberculosis 50S ribosomal subunit bound with clarithromycin reveals dynamic and specific interactions with macrolides. *Emerging Microbes & Infections*, 11(1):293–305, jan 2022.

[148] Eriko Matsuura-Suzuki, Tadahiro Shimazu, Mari Takahashi, Kaoru Kotoshiba, Takehiro Suzuki, Kazuhiro Kashiwagi, Yoshihiro Sohtome, Mai Akakabe, Mikiko Sodeoka, Naoshi Dohmae, Takuhiro Ito, Yoichi Shinkai, and Shintaro Iwasaki. METTL18-mediated histidine methylation of RPL3 modulates translation elongation for proteostasis maintenance. *eLife*, 11, jun 2022.

[149] Ravi Kiran Koripella, Ayush Deep, Ekansh K. Agrawal, Pooja Keshavan, Nilesh K. Banavali, and Rajendra K. Agrawal. Distinct mechanisms of the human mitoribosome recycling and antibiotic resistance. *Nature Communications*, 12(1), jun 2021.

[150] Zhemin Zhang, Christopher E. Morgan, Robert A. Bonomo, and Edward W. Yu. Cryo-EM Determination of Eravacycline-Bound Structures of the Ribosome and the Multidrug Efflux Pump AdeJ of Acinetobacter baumannii. *mBio*, 12(3), jun 2021.

[151] Jae Ho Lee, Ahmad Jomaa, SangYoon Chung, Yu-Hsien Hwang Fu, Ruilin Qian, Xuemeng Sun, Hao-Hsuan Hsieh, Sowmya Chandrasekar, Xiaotian Bi, Simone Mattei, Daniel Boehringer, Shimon Weiss, Nenad Ban, and Shu ou Shan. Receptor compaction and GTPase rearrangement drive SRP-mediated cotranslational protein translocation into the ER. *Science Advances*, 7(21), may 2021.

[152] Eva Kummer, Katharina Noel Schubert, Tanja Schoenhut, Alain Scaiola, and Nenad Ban. Structural basis of translation termination, rescue, and recycling in mammalian mitochondria. *Molecular Cell*, 81(12):2566–2582.e6, jun 2021.

[153] Miriam Cipullo, Gen
’ıs Valent
’ın Ges
’e, Anas Khawaja, B. Martin Hällberg, and Joanna Rorbach. Structural basis for late maturation steps of the human mitoribosomal large subunit. *Nature Communications*, 12(1), jun 2021.

[154] Ahmad Jomaa, Simon Eitzinger, Zikun Zhu, Sowmya Chandrasekar, Kan Kobayashi, Shu ou Shan, and Nenad Ban. Molecular mechanism of cargo recognition and handover by the mammalian signal recognition particle. *Cell Reports*, 36(2):109350, jul 2021.

[155] Tea Lenarčič, Mateusz Jaskolowski, Marc Leibundgut, Alain Scaiola, Tanja Schönhut, Martin Saurer, Richard G. Lee, Oliver Rackham, Aleksandra Filipovska, and Nenad Ban. Stepwise maturation of the peptidyl transferase region of human mitoribosomes. *Nature Communications*, 12(1), jun 2021.

[156] Hauke S. Hillen, Elena Lavdovskaia, Franziska Nadler, Elisa Hanitsch, Andreas Linden, Katherine E. Bohnsack, Henning Urlaub, and Ricarda Richter-Dennerlein. Structural basis of GTPase-mediated mitochondrial ribosome biogenesis and recycling. *Nature Communications*, 12(1), jun 2021.

[157] Gisela Pöll, Michael Pilsl, Joachim Griesenbeck, Herbert Tschochner, and Philipp Milkereit. Analysis of subunit folding contribution of three yeast large ribosomal subunit proteins required for stabilisation and processing of intermediate nuclear rRNA precursors. *PLOS ONE*, 16(11):e0252497, nov 2021.

[158] Jingdong Cheng, Otto Berninghausen, and Roland Beckmann. A distinct assembly pathway of the human 39S late pre-mitoribosome. *Nature Communications*, 12(1), jul 2021.

[159] Liang Xue, Swantje Lenz, Maria Zimmermann-Kogadeeva, Dimitry Tegunov, Patrick Cramer, Peer Bork, Juri Rappsilber, and Julia Mahamid. Visualizing translation dynamics at atomic detail inside a bacterial cell. *Nature*, 610(7930):205–211, sep 2022.

[160] Hiraku Takada, Caillan Crowe-McAuliffe, Christine Polte, Zhanna Yu Sidorova, Victoriia Murina, Gemma C Atkinson, Andrey L Konevega, Zoya Ignatova, Daniel N Wilson, and Vasili Hauryliuk. RqcH and RqcP catalyze processive poly-alanine synthesis in a reconstituted ribosome-associated quality control system. *Nucleic Acids Research*, 49(14):8355–8369, jul 2021.

[161] Viswanathan Chandrasekaran, Nirupa Desai, Nicholas O Burton, Hanting Yang, Jon Price, Eric A Miska, and V Ramakrishnan. Visualizing formation of the active site in the mitochondrial ribosome. *eLife*, 10, oct 2021.

[162] Florent Waltz, Thalia Salinas-Gieg
’e, Robert Englmeier, Herrade Meichel, Heddy Soufari, Lauriane Kuhn, Stefan Pfeffer, Friedrich Förster, Benjamin D. Engel, Philippe Gieg
’e, Laurence Drouard, and Yaser Hashem. How to build a ribosome from RNA fragments in Chlamydomonas mitochondria. *Nature Communications*, 12(1), dec 2021.

[163] Yuzuru Itoh, Anas Khawaja, Ivan Laptev, Miriam Cipullo, Ilian Atanassov, Petr Sergiev, Joanna Rorbach, and Alexey Amunts. Mechanism of mitoribosomal small subunit biogenesis and preinitiation. *Nature*, 606(7914):603–608, jun 2022.

[164] Disha-Gajanan Hiregange, Andre Rivalta, Tanaya Bose, Elinor Breiner-Goldstein, Sarit Samiya, Giuseppe Cimicata, Liudmila Kulakova, Ella Zimmerman, Anat Bashan, Osnat Herzberg, and Ada Yonath. Cryo-EM structure of the ancient eukaryotic ribosome from the human parasite Giardia lamblia. *Nucleic Acids Research*, 50(3):1770–1782, jan 2022.

[165] Pedro Rebelo-Guiomar, Simone Pellegrino, Kyle C. Dent, Aldema Sas-Chen, Leonor Miller-Fleming, Caterina Garone, Lindsey Van Haute, Jack F. Rogan, Adam Dinan, Andrew E. Firth, Byron Andrews, Alexander J. Whitworth, Schraga Schwartz, Alan J. Warren, and Michal Minczuk. A late-stage assembly checkpoint of the human mitochondrial ribosome large subunit. *Nature Communications*, 13(1), feb 2022.

[166] Patrick Cottilli, Yuzuru Itoh, Yuko Nobe, Anton S. Petrov, Purificación Lisón, Masato Taoka, and Alexey Amunts. Cryo-EM structure and rRNA modification sites of a plant ribosome. *Plant Communications*, 3(5):100342, sep 2022.

[167] Ahmad Jomaa, Martin Gamerdinger, Hao-Hsuan Hsieh, Annalena Wallisch, Viswanathan Chandrasekaran, Zeynel Ulusoy, Alain Scaiola, Ramanujan S. Hegde, Shu ou Shan, Nenad Ban, and Elke Deuerling. Mechanism of signal sequence handover from NAC to SRP on ribosomes during ER-protein targeting. *Science*, 375(6583):839–844, feb 2022.

[168] Zane T. Laughlin, Suparno Nandi, Debayan Dey, Natalia Zelinskaya, Marta A. Witek, Pooja Srinivas, Ha An Nguyen, Emily G. Kuiper, Lindsay R. Comstock, Christine M. Dunham, and Graeme L. Conn. 50S subunit recognition and modification by the Mycobacterium tuberculosis ribosomal RNA methyltransferase TlyA. *Proceedings of the National Academy of Sciences*, 119(14), mar 2022.

[169] Amal Seffouh, Chirstian Trahan, Tanzila Wasi, Nikhil Jain, Kaustuv Basu, Robert A Britton, Marlene Oeffinger, and Joaquin Ortega. RbgA ensures the correct timing in the maturation of the 50S subunits functional sites. *Nucleic Acids Research*, feb 2022.

[170] Anna B. Loveland, Egor Svidritskiy, Denis Susorov, Soojin Lee, Alexander Park, Sarah Zvornicanin, Gabriel Demo, Fen-Biao Gao, and Andrei A. Korostelev. Ribosome inhibition by C9ORF72-ALS/FTD-associated poly-PR and poly-GR proteins revealed by cryo-EM. *Nature Communications*, 13(1), may 2022.

[171] Laura Perlaza-Jiménez, Kher-Shing Tan, Sarah J. Piper, Rachel M. Johnson, Rebecca S. Bamert, Christopher J. Stubenrauch, Alexander Wright, David Lupton, Trevor Lithgow, and Matthew J. Belousoff. A Structurally Characterized Staphylococcus aureus Evolutionary Escape Route from Treatment with the Antibiotic Linezolid. *Microbiology Spectrum*, 10(4), aug 2022.

[172] Minkoo Ahn, Tomasz Włodarski, Alkistis Mitropoulou, Sammy H. S. Chan, Haneesh Sidhu, Elena Plessa, Thomas A. Becker, Nediljko Budisa, Christopher A. Waudby, Roland Beckmann, Anaïs M. E. Cassaignau, Lisa D. Cabrita, and John Christodoulou. Modulating co-translational protein folding by rational design and ribosome engineering. *Nature Communications*, 13(1), jul 2022.

[173] Michael Prattes, Irina Grishkovskaya, Victor-Valentin Hodirnau, Christina Hetzmannseder, Gertrude Zisser, Carolin Sailer, Vasileios Kargas, Mathias Loibl, Magdalena Gerhalter, Lisa Kofler, Alan J. Warren, Florian Stengel, David Haselbach, and Helmut Bergler. Visualizing maturation factor extraction from the nascent ribosome by the AAA-ATPase Drg1. *Nature Structural & Molecular Biology*, 29(9):942–953, sep 2022.
