# Supplementary material for: Ratchet, swivel, tilt and roll: a complete description of subunit rotation in the ribosome
Source: Nucleic Acids Res. 2022 Dec 30;51(2):919–34. doi: 10.1093/nar/gkac1211 (PMC9881166; doi:10.1093/nar/gkac1211)
Supplement: gkac1211_Supplemental_Files [file gkac1211_supplemental_files.zip › AppendixD.pdf]

Appendix D: Ribosome structures not described by the RAD method

Below are the RCSB IDs of the 35 entries that could not be analyzed using the RAD method. Brief explanations are provided.
7AFL - only contains an SSU body
7AFO - only contains an SSU body
7BGD - only contains an SSU body
7BOD - only contains an SSU body
7BOF - only contains an SSU body
7BOG - only contains an SSU body
7BOI - only contains an SSU body
7O5H - only contains an SSU body
7OI0 - only contains an SSU body
7ASA - only contains a few residues from the LSU
1C2W - early cryo-EM reconstruction. alignment failed
2FTC - early model of a mitoribosome LSU. alignment failed
2RDO - early cryo-EM model of an LSU. alignment failed
3J2C - assembly intermediate. alignment failed
6HIV - Trypanosoma bruci. alignment failed
6HIW - Trypanosoma bruci. alignment failed
6HIX - Trypanosoma bruci. alignment failed
6HIY - Trypanosoma bruci. alignment failed
6HIZ - Trypanosoma bruci. alignment failed
6SGB - Trypanosoma bruci. alignment failed
7AOR - Trypanosoma cruzi. alignment failed.
7PUA - Trypanosoma bruci. contact-based identification of head failed
7PUB - Trypanosoma bruci. contact-based identification of head failed
7QIX - only contains an SSU body
7QIY - only contains an SSU head
7WTL - human ribosome, pre-40S assembly intermediate. alignment failed
7WTM - human ribosome, pre-40S assembly intermediate. alignment failed
7WTN - human ribosome, pre-40S assembly intermediate. alignment failed
7WTO - human ribosome, pre-40S assembly intermediate. alignment failed
7WTP - human ribosome, pre-40S assembly intermediate. alignment failed
7WTQ - human ribosome, pre-40S assembly intermediate. alignment failed
7WTR - human ribosome, pre-40S assembly intermediate. alignment failed
7WTS - human ribosome, pre-40S assembly intermediate. alignment failed
7WTW - human ribosome, pre-40S assembly intermediate. alignment failed
