# Supplementary material for: Ratchet, swivel, tilt and roll: a complete description of subunit rotation in the ribosome
Source: Nucleic Acids Res. 2022 Dec 30;51(2):919–34. doi: 10.1093/nar/gkac1211 (PMC9881166; doi:10.1093/nar/gkac1211)
Supplement: gkac1211_Supplemental_Files [file gkac1211_supplemental_files.zip › AppendixE.pdf]

Appendix E: RCSB validation data for ribosome structures described with the RAD method - Assembled LSU-SSU pairs

Below are tables that provide summaries of validation statistics for 1208 structures.

For each rRNA chain, the average values were calculated for all residues that were identied as being part of a “core”. Averages were calculated from the values provided by the RCSB validation reports, which contain per-residue values.

If a validation report did not contain any values for the core residues, then the entry is listed with a “-”.

If validation quantities were only available for a fraction of the core residues, then the number of found values and the number of core residues is shown in parentheses.

Quantities calculated

- ⟨rscc⟩: average rscc value of core residues (X-RAY)
- ⟨rsr⟩: average rsr value of core residues (X-RAY)
- ⟨rsrz⟩: average rsrz value of core residues (X-RAY)
- ⟨Q_score⟩: average Q-score value of core residues (EM)
- ⟨inclusion⟩: average inclusion value of core residues (EM)

Table 1 of 27

PDB ID	chains	LSU ⟨rscc⟩	LSU ⟨rsr⟩	LSU ⟨rsrz⟩	LSU ⟨Q_score⟩	LSU ⟨inclusion⟩	SSU BODY ⟨rscc⟩	SSU BODY ⟨rsr⟩	SSU BODY ⟨rsrz⟩	SSU BODY ⟨Q_score⟩	SSU BODY ⟨inclusion⟩	SSU HEAD ⟨rscc⟩	SSU HEAD ⟨rsr⟩	SSU HEAD ⟨rsrz⟩	SSU HEAD ⟨Q_score⟩	SSU HEAD ⟨inclusion⟩
1ML5	a,A	-	-	-	-	0.21	-	-	-	-	0.22	-	-	-	-	0.25
1VVJ	RA,QA	0.95	0.17	-0.22	-	-	0.94	0.18	-0.11	-	-	0.87	0.22	0.46	-	-
1VVJ	YA,XA	0.96	0.16	-0.34	-	-	0.94	0.17	-0.16	-	-	0.91	0.19	0.11	-	-
1VY4	BA,AA	0.97	0.21	0.61	-	-	0.95	0.18	0.04	-	-	0.93	0.18	0.03	-	-
1VY4	DA,CA	0.96	0.17	-0.14	-	-	0.94	0.17	-0.17	-	-	0.86	0.21	0.61	-	-
1VY5	BA,AA	0.97	0.21	0.74	-	-	0.95	0.19	0.35	-	-	0.92	0.19	0.32	-	-
1VY5	DA,CA	0.96	0.18	0.15	-	-	0.94	0.18	0.19	-	-	0.86	0.21	0.80	-	-
1VY6	BA,AA	0.97	0.19	0.34	-	-	0.95	0.17	0.10	-	-	0.93	0.17	0.03	-	-
1VY6	DA,CA	0.96	0.17	0.02	-	-	0.94	0.16	-0.04	-	-	0.89	0.18	0.12	-	-
1VY7	BA,AA	0.97	0.18	0.22	-	-	0.94	0.18	0.22	-	-	0.92	0.17	0.04	-	-
1VY7	DA,CA	0.96	0.16	-0.12	-	-	0.93	0.17	-0.01	-	-	0.86	0.19	0.35	-	-
3DG0	B,A	-	-	-	-	0.68	-	-	-	-	0.65	-	-	-	-	0.67
3DG2	B,A	-	-	-	-	0.53	-	-	-	-	0.60	-	-	-	-	0.77
3DG4	B,A	-	-	-	-	0.62	-	-	-	-	0.57	-	-	-	-	0.65
3DG5	B,A	-	-	-	-	0.67	-	-	-	-	0.62	-	-	-	-	0.71
3J6X	2S,1S	-	-	-	-	0.98	-	-	-	-	0.98	-	-	-	-	0.96
3J6Y	2S,1S	-	-	-	-	0.98	-	-	-	-	0.98	-	-	-	-	0.96
3J77	2S,1S	-	-	-	-	0.95	-	-	-	-	0.93	-	-	-	-	0.93
3J78	2S,1S	-	-	-	-	0.95	-	-	-	-	0.92	-	-	-	-	0.93
3J7P	5,S2	-	-	-	-	0.94	-	-	-	-	0.94	-	-	-	-	0.89
3J7R	5,S2	-	-	-	-	0.91	-	-	-	-	0.88	-	-	-	-	0.90
3J9M	A,AA	-	-	-	-	0.89	-	-	-	-	0.88	-	-	-	-	0.75
3J9W	BA,AA	-	-	-	-	0.90	-	-	-	-	0.85	-	-	-	-	0.78
3J9Y	A,a	-	-	-	-	0.78 (2613/2624)	-	-	-	-	0.69 (985/989)	-	-	-	-	0.74 (443/446)
3J9Z	LA,SA	-	-	-	-	0.87	-	-	-	-	0.88	-	-	-	-	0.92
3JA1	LA,SA	-	-	-	-	0.91	-	-	-	-	0.91	-	-	-	-	0.95
3JAG	5,9	-	-	-	-	0.92	-	-	-	-	0.92	-	-	-	-	0.92
3JAH	5,9	-	-	-	-	0.92	-	-	-	-	0.93	-	-	-	-	0.93
3JAI	5,9	-	-	-	-	0.90	-	-	-	-	0.90	-	-	-	-	0.91
3JAJ	5,S2	-	-	-	-	0.93	-	-	-	-	0.93	-	-	-	-	0.92
3JAN	5,S2	-	-	-	-	0.89	-	-	-	-	0.91	-	-	-	-	0.88
3JBN	AA,A	-	-	-	-	0.80	-	-	-	-	0.82	-	-	-	-	0.85
3JBO	AA,A	-	-	-	-	0.92	-	-	-	-	0.92	-	-	-	-	0.93
3JBP	AA,A	-	-	-	-	0.90	-	-	-	-	0.90	-	-	-	-	0.89
3JBU	b,A	-	-	-	-	0.87	-	-	-	-	0.72	-	-	-	-	0.58
3JBV	b,A	-	-	-	-	0.83	-	-	-	-	0.70	-	-	-	-	0.63
3JCD	A,a	-	-	-	-	0.61	-	-	-	-	0.68	-	-	-	-	0.61
3JCE	A,a	-	-	-	-	0.35	-	-	-	-	0.35	-	-	-	-	0.36
3JCJ	A,g	-	-	-	-	0.84	-	-	-	-	0.79	-	-	-	-	0.71
3JCN	A,a	-	-	-	-	0.81	-	-	-	-	0.74	-	-	-	-	0.67
4L47	YA,XA	0.96	0.17	-0.18	-	-	0.94	0.18	-0.05	-	-	0.91	0.20	0.15	-	-
4L47	RA,QA	0.95	0.18	-0.10	-	-	0.94	0.18	-0.06	-	-	0.88	0.22	0.37	-	-
4L71	YA,XA	0.95	0.16	-0.40	-	-	0.94	0.17	-0.30	-	-	0.93	0.17	-0.26	-	-
4L71	RA,QA	0.94	0.16	-0.37	-	-	0.93	0.17	-0.27	-	-	0.87	0.21	0.18	-	-
4LEL	YA,XA	0.93	0.17	-0.24	-	-	0.91	0.18	-0.13	-	-	0.91	0.18	-0.11	-	-
4LEL	RA,QA	0.92	0.18	-0.19	-	-	0.91	0.18	-0.10	-	-	0.83	0.22	0.40	-	-
4LFZ	YA,XA	0.95	0.17	-0.24	-	-	0.94	0.18	-0.11	-	-	0.92	0.19	-0.03	-	-
4LFZ	RA,QA	0.95	0.17	-0.22	-	-	0.94	0.18	-0.18	-	-	0.88	0.22	0.33	-	-
4LNT	YA,XA	0.97	0.16	-0.09	-	-	0.95	0.16	-0.08	-	-	0.94	0.16	-0.07	-	-
4LNT	RA,QA	0.96	0.17	-0.02	-	-	0.95	0.16	-0.16	-	-	0.90	0.18	0.21	-	-
4LSK	YA,XA	0.95	0.17	-0.22	-	-	0.93	0.18	-0.02	-	-	0.92	0.19	0.08	-	-
4LSK	RA,QA	0.94	0.18	-0.09	-	-	0.93	0.19	-0.00	-	-	0.88	0.22	0.44	-	-
4LT8	YA,XA	0.96	0.24	0.62	-	-	0.94	0.27	1.10	-	-	0.92	0.29	1.23	-	-
4LT8	RA,QA	0.95	0.24	0.72	-	-	0.94	0.25	0.79	-	-	0.88	0.33	1.75	-	-
4P6F	YA,XA	0.95	0.19	-0.02	-	-	0.94	0.20	0.06	-	-	0.92	0.21	0.20	-	-

PDB		LSU					SSU BODY					SSU HEAD				
ID	chains	⟨rscc⟩	⟨rsr⟩	⟨rsrz⟩	⟨Q_score⟩	⟨inclusion⟩	⟨rscc⟩	⟨rsr⟩	⟨rsrz⟩	⟨Q_score⟩	⟨inclusion⟩	⟨rscc⟩	⟨rsr⟩	⟨rsrz⟩	⟨Q_score⟩	⟨inclusion⟩
4P6F	RA,QA	0.94	0.20	0.06	-	-	0.93	0.19	0.04	-	-	0.88	0.23	0.44	-	-
4P70	YA,XA	0.96	0.22	0.38	-	-	0.94	0.24	0.64	-	-	0.92	0.23	0.46	-	-
4P70	RA,QA	0.95	0.22	0.36	-	-	0.94	0.22	0.33	-	-	0.90	0.25	0.73	-	-
4TUA	RA,QA	0.95	0.18	-0.10	-	-	0.94	0.18	-0.12	-	-	0.90	0.20	0.07	-	-
4TUA	YA,XA	0.96	0.18	-0.14	-	-	0.94	0.19	-0.06	-	-	0.92	0.19	0.01	-	-
4TUB	RA,QA	0.94	0.18	-0.09	-	-	0.93	0.18	-0.09	-	-	0.87	0.21	0.22	-	-
4TUB	YA,XA	0.95	0.19	-0.05	-	-	0.93	0.19	0.01	-	-	0.92	0.20	0.04	-	-
4TUC	RA,QA	0.94	0.20	0.09	-	-	0.93	0.20	0.16	-	-	0.88	0.25	0.71	-	-
4TUC	YA,XA	0.95	0.19	0.04	-	-	0.93	0.20	0.06	-	-	0.92	0.20	0.16	-	-
4TUD	RA,QA	0.95	0.20	0.14	-	-	0.93	0.20	0.13	-	-	0.90	0.22	0.40	-	-
4TUD	YA,XA	0.95	0.20	0.16	-	-	0.93	0.20	0.16	-	-	0.90	0.24	0.57	-	-
4TUE	RA,QA	-	-	-	-	-	-	-	-	-	-	-	-	-	-	-
4TUE	YA,XA	-	-	-	-	-	-	-	-	-	-	-	-	-	-	-
4U1U	BA,AA	0.96	0.17	0.01	-	-	0.94	0.17	-0.03	-	-	0.92	0.16	-0.06	-	-
4U1U	DA,CA	0.84	0.21	0.59	-	-	0.89	0.18	0.22	-	-	0.80	0.22	0.70	-	-
4U1V	BA,AA	0.96	0.16	-0.35	-	-	0.95	0.15	-0.47	-	-	0.91	0.15	-0.41	-	-
4U1V	DA,CA	0.88	0.18	-0.09	-	-	0.92	0.16	-0.34	-	-	0.83	0.19	0.06	-	-
4U20	BA,AA	0.96	0.16	-0.05	-	-	0.95	0.16	-0.12	-	-	0.93	0.16	-0.18	-	-
4U20	DA,CA	0.86	0.20	0.41	-	-	0.91	0.17	0.08	-	-	0.81	0.21	0.61	-	-
4U24	BA,AA	0.97	0.15	-0.26	-	-	0.95	0.15	-0.27	-	-	0.91	0.16	-0.06	-	-
4U24	DA,CA	0.87	0.20	0.53	-	-	0.92	0.17	0.03	-	-	0.81	0.23	0.90	-	-
4U25	BA,AA	0.97	0.15	-0.21	-	-	0.95	0.15	-0.23	-	-	0.92	0.15	-0.20	-	-
4U25	DA,CA	0.88	0.19	0.29	-	-	0.92	0.17	-0.00	-	-	0.82	0.20	0.48	-	-
4U26	BA,AA	0.96	0.16	-0.13	-	-	0.95	0.15	-0.22	-	-	0.93	0.15	-0.19	-	-
4U26	DA,CA	0.86	0.19	0.36	-	-	0.91	0.17	0.01	-	-	0.82	0.20	0.41	-	-
4U27	BA,AA	0.97	0.15	-0.18	-	-	0.96	0.15	-0.28	-	-	0.94	0.15	-0.27	-	-
4U27	DA,CA	0.89	0.18	0.17	-	-	0.93	0.16	-0.14	-	-	0.85	0.19	0.26	-	-
4U3M	1,2	-	-	-	-	-	-	-	-	-	-	-	-	-	-	-
4U3M	5,6	-	-	-	-	-	-	-	-	-	-	-	-	-	-	-
4U3N	1,2	-	-	-	-	-	-	-	-	-	-	-	-	-	-	-
4U3N	5,6	-	-	-	-	-	-	-	-	-	-	-	-	-	-	-
4U3U	1,2	-	-	-	-	-	-	-	-	-	-	-	-	-	-	-
4U3U	5,6	-	-	-	-	-	-	-	-	-	-	-	-	-	-	-
4U4N	1,2	-	-	-	-	-	-	-	-	-	-	-	-	-	-	-
4U4N	5,6	-	-	-	-	-	-	-	-	-	-	-	-	-	-	-
4U4O	1,2	-	-	-	-	-	-	-	-	-	-	-	-	-	-	-
4U4O	5,6	-	-	-	-	-	-	-	-	-	-	-	-	-	-	-
4U4Q	1,2	-	-	-	-	-	-	-	-	-	-	-	-	-	-	-
4U4Q	5,6	-	-	-	-	-	-	-	-	-	-	-	-	-	-	-
4U4R	1,2	-	-	-	-	-	-	-	-	-	-	-	-	-	-	-
4U4R	5,6	-	-	-	-	-	-	-	-	-	-	-	-	-	-	-
4U4U	1,2	-	-	-	-	-	-	-	-	-	-	-	-	-	-	-
4U4U	5,6	-	-	-	-	-	-	-	-	-	-	-	-	-	-	-
4U4Y	1,2	-	-	-	-	-	-	-	-	-	-	-	-	-	-	-
4U4Y	5,6	-	-	-	-	-	-	-	-	-	-	-	-	-	-	-
4U4Z	1,2	-	-	-	-	-	-	-	-	-	-	-	-	-	-	-
4U4Z	5,6	-	-	-	-	-	-	-	-	-	-	-	-	-	-	-
4U50	1,2	-	-	-	-	-	-	-	-	-	-	-	-	-	-	-
4U50	5,6	-	-	-	-	-	-	-	-	-	-	-	-	-	-	-
4U51	1,2	-	-	-	-	-	-	-	-	-	-	-	-	-	-	-
4U51	5,6	-	-	-	-	-	-	-	-	-	-	-	-	-	-	-
4U52	1,2	-	-	-	-	-	-	-	-	-	-	-	-	-	-	-
4U52	5,6	-	-	-	-	-	-	-	-	-	-	-	-	-	-	-
4U53	1,2	-	-	-	-	-	-	-	-	-	-	-	-	-	-	-
4U53	5,6	-	-	-	-	-	-	-	-	-	-	-	-	-	-	-
4U55	1,2	-	-	-	-	-	-	-	-	-	-	-	-	-	-	-

Table 3 of 27

PDB		LSU					SSU BODY					SSU HEAD				
ID	chains	⟨rscc⟩	⟨rsr⟩	⟨rsrz⟩	⟨Q_score⟩	⟨inclusion⟩	⟨rscc⟩	⟨rsr⟩	⟨rsrz⟩	⟨Q_score⟩	⟨inclusion⟩	⟨rscc⟩	⟨rsr⟩	⟨rsrz⟩	⟨Q_score⟩	⟨inclusion⟩
4U55	5,6	-	-	-	-	-	-	-	-	-	-	-	-	-	-	-
4U56	1,2	-	-	-	-	-	-	-	-	-	-	-	-	-	-	-
4U56	5,6	-	-	-	-	-	-	-	-	-	-	-	-	-	-	-
4U6F	1,2	-	-	-	-	-	-	-	-	-	-	-	-	-	-	-
4U6F	5,6	-	-	-	-	-	-	-	-	-	-	-	-	-	-	-
4UG0	L5,S2	-	-	-	-	0.97	-	-	-	-	0.96	-	-	-	-	0.94
4UJC	A2,C1	-	-	-	-	0.97	-	-	-	-	0.97	-	-	-	-	0.97
4UJD	A2,C1	-	-	-	-	0.87	-	-	-	-	0.85	-	-	-	-	0.78
4UJE	A2,B1	-	-	-	-	0.85	-	-	-	-	0.88	-	-	-	-	0.95
4V3P	L1,S1	-	-	-	-	0.62	-	-	-	-	0.57	-	-	-	-	0.78
4V42	BA,AA	-	-	-	-	-	-	-	-	-	-	-	-	-	-	-
4V47	A0,BA	-	-	-	-	-	-	-	-	-	-	-	-	-	-	-
4V48	A0,BA	-	-	-	-	-	-	-	-	-	-	-	-	-	-	-
4V49	B0,AA	-	-	-	-	-	-	-	-	-	-	-	-	-	-	-
4V4A	B0,AA	-0.60	6.72	62.72	-	-	-0.62	4.62	42.97	-	-	-0.55	2.65	23.81	-	-
4V4B	B3,AA	-	-	-	-	0.74	-	-	-	-	0.75	-	-	-	-	0.87
4V4G	BB,AA	-	-	-	-	-	-	-	-	-	-	-	-	-	-	-
4V4G	DB,CA	-	-	-	-	-	-	-	-	-	-	-	-	-	-	-
4V4G	FB,EA	-	-	-	-	-	-	-	-	-	-	-	-	-	-	-
4V4G	HB,GA	-	-	-	-	-	-	-	-	-	-	-	-	-	-	-
4V4G	JB,IA	-	-	-	-	-	-	-	-	-	-	-	-	-	-	-
4V4H	BB,AA	0.91	0.20	0.12	-	-	0.89	0.20	0.20	-	-	0.88	0.20	0.18	-	-
4V4H	DB,CA	0.92	0.19	0.09	-	-	0.93	0.18	-0.02	-	-	0.89	0.19	0.05	-	-
4V4I	w,y	0.91	0.22	0.37	-	-	0.91	0.22	0.34<br>(694/701)	-	-	0.91	0.22	0.30<br>(301/304)	-	-
4V4J	w,y	0.94	0.19	-0.02	-	-	0.93	0.19	0.05<br>(735/741)	-	-	0.94	0.19	-0.02<br>(312/314)	-	-
4V4N	A1,B2	-	-	-	-	0.93	-	-	-	-	0.95	-	-	-	-	0.97
4V4P	AA,BA	0.90	0.13	-0.54	-	-	0.90	0.13	-0.46	-	-	0.89	0.11	-0.68	-	-
4V4Q	BB,AA	0.93	0.18	-0.13	-	-	0.92	0.17	-0.24	-	-	0.91	0.15	-0.51	-	-
4V4Q	DB,CA	0.94	0.17	-0.20	-	-	0.94	0.16	-0.29	-	-	0.91	0.14	-0.63	-	-
4V4R	BA,AA	0.91	0.22	0.33	-	-	0.88	0.22	0.40	-	-	0.85	0.26	0.71	-	-
4V4S	BA,AA	0.90	0.21	0.25	-	-	0.87	0.23	0.52	-	-	0.83	0.27	0.81	-	-
4V4T	BA,AA	0.89	0.22	0.37	-	-	0.85	0.24	0.54	-	-	0.82	0.22	0.34	-	-
4V4V	B0,AA	-	-	-	-	0.53	-	-	-	-	0.55	-	-	-	-	0.51
4V4W	B0,AA	-	-	-	-	0.70	-	-	-	-	0.71	-	-	-	-	0.40
4V4X	BA,AA	0.91	0.12	-0.57	-	-	0.91	0.12	-0.57	-	-	0.85	0.12	-0.55	-	-
4V4Y	BA,AA	0.91	0.15	-0.28	-	-	0.91	0.14	-0.36	-	-	0.87	0.14	-0.38	-	-
4V4Z	BA,AA	0.88	0.10	-0.80	-	-	0.89	0.10	-0.76	-	-	0.84	0.09	-0.91	-	-
4V50	BB,AA	0.93	0.20	0.14	-	-	0.90	0.20	0.19	-	-	0.85	0.18	-0.05	-	-
4V50	DB,CA	0.95	0.19	0.00	-	-	0.89	0.16	-0.32	-	-	0.84	0.15	-0.46	-	-
4V51	BA,AA	0.95	0.12	-0.62	-	-	0.95	0.12	-0.62	-	-	0.92	0.14	-0.32	-	-
4V51	DA,CA	0.95	0.12	-0.61	-	-	0.95	0.13	-0.56	-	-	0.91	0.15	-0.22	-	-
4V52	BB,AA	0.92	0.15	-0.42	-	-	0.92	0.14	-0.59	-	-	0.91	0.11	-0.90	-	-
4V52	DB,CA	0.94	0.15	-0.41	-	-	0.94	0.14	-0.59	-	-	0.91	0.12	-0.83	-	-
4V53	BB,AA	0.91	0.17	-0.33	-	-	0.89	0.15	-0.53	-	-	0.87	0.12	-0.88	-	-
4V53	DB,CA	0.93	0.17	-0.30	-	-	0.93	0.15	-0.53	-	-	0.88	0.12	-0.85	-	-
4V54	BB,AA	0.92	0.18	-0.11	-	-	0.91	0.17	-0.16	-	-	0.90	0.17	-0.24	-	-
4V54	DB,CA	0.93	0.18	-0.12	-	-	0.94	0.17	-0.24	-	-	0.90	0.17	-0.24	-	-
4V55	BB,AA	0.92	0.16	-0.18	-	-	0.92	0.15	-0.31	-	-	0.91	0.12	-0.55	-	-
4V55	DB,CA	0.94	0.16	-0.22	-	-	0.94	0.14	-0.36	-	-	0.92	0.12	-0.58	-	-
4V56	BB,AA	0.93	0.15	-0.49	-	-	0.92	0.13	-0.81	-	-	0.92	0.13	-0.78	-	-
4V56	DB,CA	0.94	0.15	-0.53	-	-	0.94	0.13	-0.71	-	-	0.93	0.12	-0.87	-	-
4V57	BB,AA	0.92	0.15	-0.51	-	-	0.91	0.13	-0.80	-	-	0.90	0.12	-0.86	-	-
4V57	DB,CA	0.94	0.15	-0.50	-	-	0.94	0.14	-0.67	-	-	0.91	0.12	-0.86	-	-
4V5A	BA,AA	0.92	0.23	0.49	-	-	0.93	0.22	0.37	-	-	0.89	0.25	0.77	-	-

PDB		LSU					SSU BODY					SSU HEAD				
ID	chains	⟨rscc⟩	⟨rsr⟩	⟨rsrz⟩	⟨Q_score⟩	⟨inclusion⟩	⟨rscc⟩	⟨rsr⟩	⟨rsrz⟩	⟨Q_score⟩	⟨inclusion⟩	⟨rscc⟩	⟨rsr⟩	⟨rsrz⟩	⟨Q_score⟩	⟨inclusion⟩
4V5A	DA,CA	0.92	0.23	0.45	-	-	0.92	0.23	0.47	-	-	0.86	0.27	1.02	-	-
4V5B	AB,BA	0.94	0.15	-0.51	-	-	0.93	0.15	-0.49	-	-	0.89	0.17	-0.23	-	-
4V5B	CB,DA	0.95	0.14	-0.66	-	-	0.95	0.13	-0.71	-	-	0.91	0.18	-0.20	-	-
4V5C	BA,AA	0.95	0.17	-0.21	-	-	0.95	0.16	-0.30	-	-	0.92	0.17	-0.21	-	-
4V5C	DA,CA	0.94	0.17	-0.15	-	-	0.94	0.17	-0.23	-	-	0.86	0.20	0.21	-	-
4V5D	BA,AA	0.96	0.18	-0.15	-	-	0.94	0.18	-0.16	-	-	0.92	0.18	-0.08	-	-
4V5D	DA,CA	0.94	0.19	-0.06	-	-	0.93	0.18	-0.12	-	-	0.89	0.21	0.18	-	-
4V5E	BA,AA	0.96	0.17	-0.25	-	-	0.93	0.18	-0.11	-	-	0.87	0.22	0.36	-	-
4V5E	DA,CA	0.96	0.16	-0.26	-	-	0.94	0.17	-0.14	-	-	0.93	0.18	-0.12	-	-
4V5F	BA,AA	0.96	0.16	-0.41	-	-	0.97	0.16	-0.37	-	-	0.96	0.17	-0.32	-	-
4V5F	DA,CA	0.96	0.15	-0.56	-	-	0.96	0.15	-0.56	-	-	0.95	0.14	-0.64	-	-
4V5G	BA,AA	0.91	0.18	-0.09	-	-	0.93	0.18	-0.09	-	-	0.92	0.18	-0.13	-	-
4V5G	DA,CA	0.91	0.18	-0.14	-	-	0.93	0.18	-0.20	-	-	0.92	0.17	-0.25	-	-
4V5H	BB,AA	-	-	-	-	0.80	-	-	-	-	0.78	-	-	-	-	0.77
4V5J	BA,AA	0.95	0.17	-0.24	-	-	0.94	0.17	-0.21	-	-	0.85	0.21	0.28	-	-
4V5J	DA,CA	0.97	0.16	-0.37	-	-	0.93	0.17	-0.15	-	-	0.90	0.18	-0.03	-	-
4V5K	BA,AA	0.95	0.18	-0.10	-	-	0.94	0.18	-0.13	-	-	0.86	0.21	0.35	-	-
4V5K	DA,CA	0.94	0.18	-0.02	-	-	0.82	0.24	0.63	-	-	0.77	0.27	1.03	-	-
4V5L	BA,AA	0.94	0.19	0.05	-	-	0.96	0.18	-0.08	-	-	0.95	0.18	-0.02	-	-
4V5M	BA,AA	-	-	-	-	0.86	-	-	-	-	0.85	-	-	-	-	0.85
4V5N	BA,AA	-	-	-	-	0.00	-	-	-	-	0.00	-	-	-	-	0.00
4V5P	BA,AA	0.94	0.18	-0.08	-	-	0.95	0.17	-0.14	-	-	0.95	0.19	0.07	-	-
4V5P	DA,CA	0.93	0.17	-0.17	-	-	0.94	0.16	-0.29	-	-	0.93	0.16	-0.29	-	-
4V5Q	BA,AA	0.94	0.16	-0.29	-	-	0.95	0.16	-0.38	-	-	0.95	0.16	-0.30	-	-
4V5Q	DA,CA	0.93	0.17	-0.26	-	-	0.94	0.16	-0.36	-	-	0.93	0.17	-0.23	-	-
4V5R	BA,AA	0.93	0.17	-0.22	-	-	0.95	0.15	-0.39	-	-	0.93	0.16	-0.36	-	-
4V5R	DA,CA	0.94	0.18	-0.13	-	-	0.95	0.17	-0.22	-	-	0.95	0.19	0.04	-	-
4V5S	BA,AA	0.95	0.15	-0.39	-	-	0.97	0.16	-0.34	-	-	0.96	0.16	-0.27	-	-
4V5S	DA,CA	0.95	0.15	-0.40	-	-	0.96	0.15	-0.40	-	-	0.93	0.15	-0.42	-	-
4V5Y	BB,AA	0.92	0.17	-0.09	-	-	0.91	0.18	-0.05	-	-	0.89	0.19	0.12	-	-
4V5Y	DB,CA	0.93	0.16	-0.17	-	-	0.94	0.16	-0.19	-	-	0.90	0.18	0.00	-	-
4V5Z	B0,AA	-	-	-	-	0.93	-	-	-	-	0.90	-	-	-	-	0.93
4V61	BA,AA	-	-	-	-	0.71	-	-	-	-	0.75	-	-	-	-	0.77
4V63	BA,AA	0.92	0.19	-0.01	-	-	0.91	0.19	0.01	-	-	0.86	0.22	0.37	-	-
4V63	DA,CA	0.92	0.19	0.01	-	-	0.88	0.21	0.36	-	-	0.83	0.23	0.58	-	-
4V64	BB,AA	0.93	0.15	-0.51	-	-	0.92	0.14	-0.68	-	-	0.89	0.11	-0.96	-	-
4V64	DB,CA	0.95	0.15	-0.52	-	-	0.95	0.13	-0.72	-	-	0.89	0.12	-0.89	-	-
4V65	BB,A1	-	-	-	-	0.43	-	-	-	-	0.28	-	-	-	-	0.28
4V66	BB,A1	-	-	-	-	0.41	-	-	-	-	0.27	-	-	-	-	0.27
4V67	BA,AA	0.94	0.15	-0.44	-	-	0.94	0.15	-0.50	-	-	0.90	0.18	-0.10	-	-
4V67	DA,CA	0.93	0.16	-0.37	-	-	0.92	0.16	-0.30	-	-	0.86	0.20	0.17	-	-
4V68	BA,AA	-	-	-	-	0.77	-	-	-	-	0.80	-	-	-	-	0.84
4V69	BB,AA	-	-	-	-	0.91	-	-	-	-	0.89	-	-	-	-	0.91
4V6A	DA,CA	0.93	0.19	0.06	-	-	0.93	0.19	0.01	-	-	0.90	0.20	0.22	-	-
4V6A	BA,AA	0.95	0.19	0.01	-	-	0.93	0.18	-0.05	-	-	0.90	0.20	0.18	-	-
4V6C	BA,AA	0.97	0.14	-0.58	-	-	0.97	0.14	-0.63	-	-	0.94	0.15	-0.47	-	-
4V6C	DA,CA	0.86	0.22	0.36	-	-	0.92	0.17	-0.16	-	-	0.81	0.23	0.54	-	-
4V6D	BA,AA	0.96	0.15	-0.51	-	-	0.95	0.14	-0.58	-	-	0.93	0.16	-0.46	-	-
4V6D	DA,CA	0.90	0.18	-0.12	-	-	0.93	0.16	-0.36	-	-	0.91	0.17	-0.30	-	-
4V6E	BA,AA	0.96	0.16	-0.46	-	-	0.94	0.15	-0.57	-	-	0.91	0.16	-0.40	-	-
4V6E	DA,CA	0.83	0.21	0.24	-	-	0.89	0.18	-0.21	-	-	0.88	0.18	-0.18	-	-
4V6F	DA,CA	0.97	0.17	-0.25	-	-	0.95	0.15	-0.45	-	-	0.93	0.15	-0.49	-	-
4V6F	AA,BA	0.95	0.16	-0.26	-	-	0.95	0.15	-0.50	-	-	0.89	0.13	-0.63	-	-
4V6G	DA,CA	0.96	0.14	-0.60	-	-	0.94	0.12	-0.84	-	-	0.90	0.13	-0.79	-	-
4V6G	BA,AA	0.95	0.13	-0.72	-	-	0.94	0.13	-0.80	-	-	0.89	0.12	-0.90	-	-
4V6I	DA,CA	-	-	-	-	0.84	-	-	-	-	0.83	-	-	-	-	0.82

Table 5 of 27

PDB		LSU					SSU BODY					SSU HEAD				
ID	chains	⟨rscc⟩	⟨rsr⟩	⟨rsrz⟩	⟨Q_score⟩	⟨inclusion⟩	⟨rscc⟩	⟨rsr⟩	⟨rsrz⟩	⟨Q_score⟩	⟨inclusion⟩	⟨rscc⟩	⟨rsr⟩	⟨rsrz⟩	⟨Q_score⟩	⟨inclusion⟩
4V6K	AB,BA	-	-	-	-	1.00 (2085/2092)	-	-	-	-	1.00 (830/834)	-	-	-	-	1.00 (365/367)
4V6L	BB,AA	-	-	-	-	1.00 (1673/1679)	-	-	-	-	0.99 (609/613)	-	-	-	-	0.99 (276/279)
4V6M	B8,AA	-	-	-	-	0.99	-	-	-	-	0.99	-	-	-	-	0.99
4V6N	AB,BA	-	-	-	-	1.00 (1654/1662)	-	-	-	-	1.00 (512/514)	-	-	-	-	1.00 (265/266)
4V6O	BB,AA	-	-	-	-	1.00 (1345/1349)	-	-	-	-	1.00 (476/478)	-	-	-	-	1.00 (204/206)
4V6P	BB,AA	-	-	-	-	1.00 (1211/1215)	-	-	-	-	1.00	-	-	-	-	1.00 (133/135)
4V6Q	BB,AA	-	-	-	-	1.00 (1490/1494)	-	-	-	-	1.00 (456/457)	-	-	-	-	1.00 (219/222)
4V6R	BB,AA	-	-	-	-	1.00 (1482/1489)	-	-	-	-	1.00	-	-	-	-	1.00 (169/171)
4V6S	AB,BA	-	-	-	-	1.00 (1465/1471)	-	-	-	-	1.00 (515/518)	-	-	-	-	1.00 (211/214)
4V6T	BA,AA	-	-	-	-	0.92	-	-	-	-	0.90	-	-	-	-	0.91
4V6U	B1,A2	-	-	-	-	0.73	-	-	-	-	0.75	-	-	-	-	0.61
4V6V	BA,AA	-	-	-	-	1.00 (2194/2203)	-	-	-	-	1.00 (861/865)	-	-	-	-	1.00 (391/393)
4V6W	A5,B2	-	-	-	-	0.96	-	-	-	-	0.94	-	-	-	-	0.93
4V6X	A5,B2	-	-	-	-	0.80	-	-	-	-	0.79	-	-	-	-	0.75
4V6Y	BA,AA	-	-	-	-	0.62	-	-	-	-	0.65	-	-	-	-	0.59
4V6Z	BA,AA	-	-	-	-	0.57	-	-	-	-	0.69	-	-	-	-	0.67
4V70	BA,AA	-	-	-	-	0.90	-	-	-	-	0.90	-	-	-	-	0.93
4V71	BA,AA	-	-	-	-	0.77	-	-	-	-	0.86	-	-	-	-	0.88
4V72	BA,AA	-	-	-	-	0.83	-	-	-	-	0.87	-	-	-	-	0.87
4V73	BA,AA	-	-	-	-	0.82	-	-	-	-	0.85	-	-	-	-	0.87
4V74	BA,AA	-	-	-	-	0.87	-	-	-	-	0.83	-	-	-	-	0.86
4V75	BA,AA	-	-	-	-	0.85	-	-	-	-	0.85	-	-	-	-	0.86
4V76	BA,AA	-	-	-	-	0.72	-	-	-	-	0.79	-	-	-	-	0.82
4V77	BA,AA	-	-	-	-	0.79	-	-	-	-	0.82	-	-	-	-	0.84
4V78	BA,AA	-	-	-	-	0.91	-	-	-	-	0.93	-	-	-	-	0.90
4V79	BA,AA	-	-	-	-	0.71	-	-	-	-	0.75	-	-	-	-	0.88
4V7A	BA,AA	-	-	-	-	0.49	-	-	-	-	0.53	-	-	-	-	0.48
4V7B	BA,AA	-	-	-	-	0.79	-	-	-	-	0.80	-	-	-	-	0.81
4V7C	BA,AA	-	-	-	-	0.98	-	-	-	-	0.97	-	-	-	-	0.95
4V7D	AA,BA	-	-	-	-	0.98	-	-	-	-	0.97	-	-	-	-	0.95
4V7E	Aa,Ad	-	-	-	-	0.82	-	-	-	-	0.81	-	-	-	-	0.82
4V7H	B5,AA	-	-	-	-	0.73	-	-	-	-	0.78	-	-	-	-	0.78
4V7I	A8,BA	-	-	-	-	1.00	-	-	-	-	1.00	-	-	-	-	1.00
4V7J	AA,Aa	0.95	0.17	-0.18	-	-	0.94	0.18	-0.07	-	-	0.88	0.20	0.16	-	-
4V7J	BA,Ba	0.95	0.18	-0.01	-	-	0.93	0.18	-0.12	-	-	0.88	0.20	0.17	-	-
4V7K	AA,Aa	0.95	0.17	-0.21	-	-	0.93	0.18	-0.10	-	-	0.86	0.22	0.30	-	-
4V7K	BA,Ba	0.95	0.19	-0.04	-	-	0.93	0.18	-0.12	-	-	0.88	0.21	0.25	-	-
4V7L	BA,AA	0.95	0.17	-0.18	-	-	0.94	0.17	-0.19	-	-	0.92	0.18	-0.06	-	-
4V7L	DA,CA	0.94	0.18	-0.11	-	-	0.94	0.17	-0.19	-	-	0.90	0.19	0.09	-	-
4V7M	BA,AA	0.95	0.16	-0.31	-	-	0.94	0.17	-0.20	-	-	0.91	0.18	-0.04	-	-
4V7M	DA,CA	0.88	0.22	0.42	-	-	0.92	0.19	-0.00	-	-	0.87	0.21	0.34	-	-
4V7P	BA,AA	0.93	0.20	0.07	-	-	0.91	0.21	0.21	-	-	0.83	0.24	0.62	-	-
4V7P	CA,DA	0.93	0.20	0.15	-	-	0.90	0.22	0.35	-	-	0.88	0.21	0.26	-	-
4V7R	B1,A1	0.93	0.18	-0.07	-	-	0.91	0.17	-0.12	-	-	0.90	0.17	-0.15	-	-
4V7R	D1,C1	0.92	0.18	-0.02	-	-	0.91	0.18	-0.07	-	-	0.91	0.17	-0.13	-	-
4V7S	BA,AA	0.96	0.15	-0.44	-	-	0.95	0.15	-0.48	-	-	0.90	0.15	-0.43	-	-
4V7S	DA,CA	0.82	0.22	0.47	-	-	0.91	0.18	-0.11	-	-	0.78	0.20	0.21	-	-

Table 6 of 27

PDB		LSU					SSU BODY					SSU HEAD				
ID	chains	⟨rscc⟩	⟨rsr⟩	⟨rsrz⟩	⟨Q_score⟩	⟨inclusion⟩	⟨rscc⟩	⟨rsr⟩	⟨rsrz⟩	⟨Q_score⟩	⟨inclusion⟩	⟨rscc⟩	⟨rsr⟩	⟨rsrz⟩	⟨Q_score⟩	⟨inclusion⟩
4V7T	BA,AA	0.98	0.14	-0.63	-	-	0.97	0.13	-0.70	-	-	0.93	0.14	-0.63	-	-
4V7T	DA,CA	0.86	0.20	0.11	-	-	0.92	0.16	-0.29	-	-	0.80	0.20	0.14	-	-
4V7U	BA,AA	0.97	0.14	-0.56	-	-	0.96	0.14	-0.63	-	-	0.91	0.14	-0.55	-	-
4V7U	DA,CA	0.85	0.20	0.18	-	-	0.91	0.17	-0.15	-	-	0.77	0.21	0.29	-	-
4V7V	BA,AA	0.97	0.14	-0.58	-	-	0.97	0.14	-0.63	-	-	0.93	0.14	-0.54	-	-
4V7V	DA,CA	0.87	0.20	0.17	-	-	0.92	0.16	-0.27	-	-	0.80	0.21	0.29	-	-
4V7W	BA,AA	0.95	0.21	0.31	-	-	0.90	0.22	0.38	-	-	0.71	0.35	2.03	-	-
4V7W	DA,CA	0.94	0.19	0.07	-	-	0.91	0.21	0.33	-	-	0.74	0.32	1.72	-	-
4V7X	BA,AA	0.96	0.20	0.20	-	-	0.90	0.22	0.43	-	-	0.72	0.35	2.05	-	-
4V7X	DA,CA	0.95	0.19	0.05	-	-	0.90	0.22	0.39	-	-	0.73	0.35	2.05	-	-
4V7Y	BA,AA	0.96	0.18	-0.07	-	-	0.92	0.19	0.05	-	-	0.78	0.30	1.37	-	-
4V7Y	DA,CA	0.95	0.16	-0.29	-	-	0.92	0.18	-0.03	-	-	0.77	0.31	1.48	-	-
4V7Z	BA,AA	0.95	0.21	0.30	-	-	0.91	0.21	0.33	-	-	0.77	0.31	1.58	-	-
4V7Z	DA,CA	0.94	0.20	0.12	-	-	0.90	0.21	0.29	-	-	0.77	0.33	1.72	-	-
4V83	BA,AA	0.94	0.18	-0.16	-	-	0.93	0.18	-0.20	-	-	0.84	0.22	0.37	-	-
4V83	DA,CA	0.95	0.18	-0.18	-	-	0.89	0.20	0.11	-	-	0.80	0.24	0.54	-	-
4V84	BA,AA	0.95	0.18	-0.08	-	-	0.92	0.20	0.14	-	-	0.83	0.23	0.55	-	-
4V84	DA,CA	0.95	0.19	0.02	-	-	0.90	0.20	0.23	-	-	0.80	0.24	0.70	-	-
4V85	BA,AA	0.94	0.20	0.11	-	-	0.91	0.20	0.14	-	-	0.81	0.27	1.04	-	-
4V87	AA,BA	0.96	0.22	0.49	-	-	0.94	0.23	0.55	-	-	0.90	0.24	0.69	-	-
4V87	DA,CA	0.95	0.23	0.51	-	-	0.94	0.23	0.50	-	-	0.87	0.26	0.93	-	-
4V88	A1,A2	0.97	0.19	0.07	-	-	0.96	0.18	-0.07	-	-	0.95	0.18	-0.10	-	-
4V88	A5,A6	0.97	0.20	0.13	-	-	0.96	0.19	0.01	-	-	0.95	0.18	-0.05	-	-
4V89	BA,AA	0.93	0.18	-0.11	-	-	0.91	0.19	-0.02	-	-	0.84	0.22	0.36	-	-
4V8A	AA,DA	0.95	0.18	-0.12	-	-	0.92	0.18	-0.07	-	-	0.80	0.30	1.46	-	-
4V8A	BA,CA	0.96	0.19	0.03	-	-	0.92	0.18	-0.01	-	-	0.78	0.32	1.60	-	-
4V8B	BA,AA	0.97	0.16	-0.33	-	-	0.95	0.15	-0.48	-	-	0.92	0.13	-0.75	-	-
4V8B	DA,CA	0.95	0.16	-0.31	-	-	0.94	0.14	-0.52	-	-	0.87	0.13	-0.68	-	-
4V8C	AA,CA	0.96	0.18	-0.07	-	-	0.94	0.16	-0.33	-	-	0.92	0.15	-0.43	-	-
4V8C	BA,DA	0.95	0.17	-0.17	-	-	0.94	0.16	-0.36	-	-	0.90	0.14	-0.51	-	-
4V8D	BA,AA	0.96	0.18	-0.09	-	-	0.95	0.16	-0.36	-	-	0.91	0.16	-0.38	-	-
4V8D	DA,CA	0.95	0.16	-0.29	-	-	0.95	0.16	-0.35	-	-	0.89	0.16	-0.33	-	-
4V8E	AA,BA	0.96	0.16	-0.27	-	-	0.94	0.15	-0.48	-	-	0.92	0.14	-0.52	-	-
4V8E	CA,DA	0.94	0.15	-0.46	-	-	0.93	0.14	-0.62	-	-	0.87	0.14	-0.62	-	-
4V8F	AA,BA	0.96	0.16	-0.35	-	-	0.94	0.15	-0.47	-	-	0.92	0.13	-0.71	-	-
4V8F	DA,CA	0.95	0.16	-0.28	-	-	0.94	0.15	-0.51	-	-	0.89	0.13	-0.64	-	-
4V8G	DA,CA	0.96	0.16	-0.33	-	-	0.93	0.18	-0.04	-	-	0.76	0.34	1.89	-	-
4V8G	BA,AA	0.97	0.18	-0.07	-	-	0.91	0.19	0.10	-	-	0.61	0.44	3.18	-	-
4V8H	BA,AA	0.97	0.16	-0.34	-	-	0.93	0.16	-0.26	-	-	0.82	0.22	0.45	-	-
4V8H	DA,CA	0.96	0.14	-0.56	-	-	0.94	0.15	-0.38	-	-	0.80	0.24	0.64	-	-
4V8I	BA,AA	0.98	0.17	-0.21	-	-	0.95	0.17	-0.15	-	-	0.93	0.17	-0.22	-	-
4V8I	DA,CA	0.96	0.14	-0.60	-	-	0.94	0.16	-0.27	-	-	0.85	0.22	0.71	-	-
4V8J	BA,AA	0.91	0.22	0.36	-	-	0.93	0.20	0.06	-	-	0.91	0.21	0.18	-	-
4V8J	DA,CA	0.92	0.22	0.32	-	-	0.93	0.19	0.03	-	-	0.91	0.20	0.14	-	-
4V8N	BA,AA	0.94	0.20	0.16	-	-	0.90	0.21	0.32	-	-	0.84	0.24	0.70	-	-
4V8N	DA,CA	0.92	0.20	0.21	-	-	0.90	0.20	0.21	-	-	0.78	0.27	1.03	-	-
4V8O	BA,AA	0.88	0.20	0.06	-	-	0.89	0.18	-0.11	-	-	0.92	0.17	-0.30	-	-
4V8Q	AA,BA	0.96	0.16	-0.32	-	-	0.94	0.17	-0.21	-	-	0.92	0.19	0.06	-	-
4V8U	BA,AA	0.94	0.18	-0.09	-	-	0.95	0.18	-0.18	-	-	0.95	0.18	-0.16	-	-
4V8U	DA,CA	0.93	0.18	-0.09	-	-	0.94	0.18	-0.19	-	-	0.93	0.19	-0.08	-	-
4V8X	BA,AA	0.95	0.18	-0.05	-	-	0.94	0.18	-0.12	-	-	0.88	0.20	0.21	-	-
4V8X	DA,CA	0.95	0.18	-0.04	-	-	0.91	0.19	0.05	-	-	0.85	0.21	0.34	-	-
4V8Y	B5,B2	-	-	-	-	0.77	-	-	-	-	0.70	-	-	-	-	0.50
4V8Z	B5,B2	-	-	-	-	0.77	-	-	-	-	0.68	-	-	-	-	0.40
4V90	BA,AA	0.96	0.17	-0.02	-	-	0.95	0.17	0.01	-	-	0.92	0.18	0.20	-	-
4V95	BA,AA	0.98	0.15	-0.51	-	-	0.95	0.16	-0.27	-	-	0.93	0.18	-0.03	-	-

PDB		LSU					SSU BODY					SSU HEAD				
ID	chains	⟨rscc⟩	⟨rsr⟩	⟨rsrz⟩	⟨Q_score⟩	⟨inclusion⟩	⟨rscc⟩	⟨rsr⟩	⟨rsrz⟩	⟨Q_score⟩	⟨inclusion⟩	⟨rscc⟩	⟨rsr⟩	⟨rsrz⟩	⟨Q_score⟩	⟨inclusion⟩
4V95	DA,CA	0.96	0.16	-0.37	-	-	0.95	0.17	-0.23	-	-	0.89	0.21	0.32	-	-
4V97	DA,CA	0.95	0.19	-0.05	-	-	0.93	0.20	0.07	-	-	0.92	0.20	0.12	-	-
4V97	BA,AA	0.94	0.19	0.04	-	-	0.93	0.19	0.03	-	-	0.90	0.21	0.17	-	-
4V9A	BA,AA	0.96	0.16	-0.35	-	-	0.94	0.14	-0.61	-	-	0.92	0.14	-0.52	-	-
4V9A	DA,CA	0.95	0.15	-0.41	-	-	0.94	0.14	-0.63	-	-	0.87	0.12	-0.86	-	-
4V9B	BA,AA	0.96	0.15	-0.42	-	-	0.94	0.13	-0.67	-	-	0.92	0.12	-0.78	-	-
4V9B	DA,CA	0.96	0.14	-0.62	-	-	0.95	0.13	-0.74	-	-	0.90	0.12	-0.82	-	-
4V9C	BA,AA	0.94	0.15	-0.48	-	-	0.94	0.15	-0.49	-	-	0.92	0.14	-0.56	-	-
4V9C	DA,CA	0.96	0.15	-0.48	-	-	0.95	0.14	-0.54	-	-	0.92	0.15	-0.42	-	-
4V9D	CA,AA	0.97	0.14	-0.60	-	-	0.96	0.13	-0.72	-	-	0.94	0.14	-0.63	-	-
4V9D	DA,BA	0.95	0.14	-0.60	-	-	0.95	0.13	-0.66	-	-	0.91	0.15	-0.40	-	-
4V9H	BA,AA	0.97	0.13	-0.50	-	-	0.97	0.13	-0.47	-	-	0.96	0.15	-0.31	-	-
4V9I	BA,AA	0.97	0.16	-0.38	-	-	0.94	0.18	-0.13	-	-	0.92	0.19	0.07	-	-
4V9I	DA,CA	0.95	0.17	-0.18	-	-	0.94	0.17	-0.14	-	-	0.85	0.24	0.70	-	-
4V9J	BA,AA	0.94	0.21	0.23	-	-	0.94	0.23	0.45	-	-	0.94	0.23	0.51	-	-
4V9J	DA,CA	0.94	0.21	0.23	-	-	0.94	0.22	0.41	-	-	0.94	0.24	0.64	-	-
4V9K	BA,AA	0.97	0.15	-0.57	-	-	0.97	0.14	-0.58	-	-	0.96	0.14	-0.58	-	-
4V9K	DA,CA	0.97	0.15	-0.55	-	-	0.96	0.14	-0.62	-	-	0.95	0.15	-0.52	-	-
4V9L	BA,AA	-	-	-	-	-	-	-	-	-	-	-	-	-	-	-
4V9L	DA,CA	-	-	-	-	-	-	-	-	-	-	-	-	-	-	-
4V9M	BA,AA	0.96	0.16	-0.23	-	-	0.96	0.19	0.08	-	-	0.94	0.19	0.11	-	-
4V9M	DA,CA	0.97	0.16	-0.25	-	-	0.96	0.18	0.04	-	-	0.94	0.18	0.01	-	-
4V9N	BA,AA	0.94	0.19	0.00	-	-	0.93	0.18	-0.03	-	-	0.86	0.22	0.43	-	-
4V9N	DA,CA	0.95	0.18	-0.06	-	-	0.91	0.20	0.17	-	-	0.84	0.23	0.60	-	-
4V9O	AA,BA	0.97	0.16	-0.12	-	-	0.97	0.15	-0.28	-	-	0.96	0.15	-0.25	-	-
4V9O	CA,DA	0.96	0.15	-0.28	-	-	0.95	0.14	-0.35	-	-	0.90	0.15	-0.20	-	-
4V9O	EA,FA	0.97	0.16	-0.13	-	-	0.95	0.15	-0.23	-	-	0.95	0.15	-0.26	-	-
4V9O	GA,HA	0.93	0.16	-0.17	-	-	0.91	0.17	0.04	-	-	0.78	0.24	1.03	-	-
4V9P	AA,BA	0.96	0.15	-0.31	-	-	0.95	0.14	-0.36	-	-	0.94	0.15	-0.24	-	-
4V9P	CA,DA	0.96	0.15	-0.32	-	-	0.95	0.14	-0.35	-	-	0.94	0.14	-0.32	-	-
4V9P	EA,FA	0.97	0.15	-0.31	-	-	0.96	0.14	-0.33	-	-	0.96	0.14	-0.35	-	-
4V9P	GA,HA	0.96	0.14	-0.33	-	-	0.96	0.15	-0.29	-	-	0.93	0.16	-0.09	-	-
4V9Q	AA,BA	0.94	0.21	0.28	-	-	0.93	0.21	0.30	-	-	0.87	0.22	0.46	-	-
4V9Q	CA,DA	0.95	0.21	0.24	-	-	0.91	0.22	0.48	-	-	0.85	0.24	0.64	-	-
4V9R	BA,AA	0.98	0.17	-0.25	-	-	0.95	0.16	-0.26	-	-	0.91	0.19	0.00	-	-
4V9R	DA,CA	0.97	0.15	-0.50	-	-	0.95	0.16	-0.35	-	-	0.89	0.20	0.20	-	-
4V9S	BA,AA	0.98	0.16	-0.28	-	-	0.95	0.16	-0.31	-	-	0.92	0.17	-0.15	-	-
4V9S	DA,CA	0.97	0.14	-0.54	-	-	0.95	0.15	-0.39	-	-	0.90	0.19	0.06	-	-
4W29	BA,AA	0.91	0.15	-0.53	-	-	0.91	0.15	-0.47	-	-	0.91	0.15	-0.56	-	-
4W29	DA,CA	0.91	0.15	-0.56	-	-	0.91	0.15	-0.56	-	-	0.90	0.16	-0.45	-	-
4W2E	A,a	0.95	0.15	-0.27	-	-	0.95	0.15	-0.31	-	-	0.95	0.15	-0.27	-	-
4W2F	BA,AA	0.97	0.21	0.66	-	-	0.94	0.18	0.15	-	-	0.92	0.18	0.22	-	-
4W2F	DA,CA	0.95	0.17	0.04	-	-	0.93	0.17	0.03	-	-	0.87	0.21	0.64	-	-
4W2G	BA,AA	0.97	0.20	0.47	-	-	0.95	0.18	0.14	-	-	0.94	0.18	0.08	-	-
4W2G	DA,CA	0.96	0.16	-0.18	-	-	0.94	0.17	-0.03	-	-	0.89	0.18	0.09	-	-
4W2H	BA,AA	0.98	0.21	0.59	-	-	0.95	0.18	0.09	-	-	0.92	0.18	-0.00	-	-
4W2H	DA,CA	0.97	0.18	-0.05	-	-	0.95	0.17	-0.07	-	-	0.88	0.19	0.29	-	-
4W2I	BA,AA	0.97	0.23	0.97	-	-	0.96	0.21	0.53	-	-	0.94	0.20	0.46	-	-
4W2I	DA,CA	0.96	0.20	0.35	-	-	0.95	0.20	0.36	-	-	0.89	0.19	0.29	-	-
4W4G	RA,QA	0.96	0.17	-0.15	-	-	0.95	0.18	-0.13	-	-	0.91	0.20	0.23	-	-
4W4G	YA,XA	0.96	0.17	-0.16	-	-	0.95	0.18	-0.11	-	-	0.93	0.19	0.04	-	-
4WF1	BA,AA	0.97	0.17	-0.18	-	-	0.95	0.16	-0.28	-	-	0.89	0.19	0.06	-	-
4WF1	DA,CA	0.85	0.23	0.50	-	-	0.90	0.19	0.04	-	-	0.77	0.27	1.06	-	-
4WOI	BA,AA	0.97	0.14	-0.63	-	-	0.97	0.13	-0.68	-	-	0.95	0.13	-0.65	-	-
4WOI	CA,DA	0.96	0.14	-0.57	-	-	0.96	0.13	-0.64	-	-	0.95	0.13	-0.66	-	-
4WPO	AA,BA	0.98	0.15	-0.31	-	-	0.95	0.16	-0.16	-	-	0.91	0.17	0.10	-	-

Table 8 of 27

PDB		LSU					SSU BODY					SSU HEAD				
ID	chains	⟨rscc⟩	⟨rsr⟩	⟨rsrz⟩	⟨Q_score⟩	⟨inclusion⟩	⟨rscc⟩	⟨rsr⟩	⟨rsrz⟩	⟨Q_score⟩	⟨inclusion⟩	⟨rscc⟩	⟨rsr⟩	⟨rsrz⟩	⟨Q_score⟩	⟨inclusion⟩
4WPO	CA,DA	0.96	0.15	-0.24	-	-	0.95	0.15	-0.19	-	-	0.86	0.20	0.44	-	-
4WQ1	1H,13	0.97	0.17	-0.18	-	-	0.95	0.15	-0.43	-	-	0.94	0.13	-0.64	-	-
4WQ1	14,1G	0.96	0.16	-0.30	-	-	0.95	0.17	-0.25	-	-	0.90	0.16	-0.34	-	-
4WQF	AA,BA	0.98	0.17	-0.02	-	-	0.94	0.17	0.05	-	-	0.86	0.22	0.75	-	-
4WQF	CA,DA	0.96	0.17	-0.00	-	-	0.94	0.17	0.04	-	-	0.81	0.25	1.14	-	-
4WQR	1H,13	0.96	0.16	-0.35	-	-	0.94	0.15	-0.51	-	-	0.93	0.11	-0.92	-	-
4WQR	14,1G	0.95	0.16	-0.35	-	-	0.94	0.14	-0.58	-	-	0.88	0.12	-0.83	-	-
4WQU	AA,BA	0.98	0.15	-0.22	-	-	0.94	0.16	-0.12	-	-	0.88	0.19	0.33	-	-
4WQU	CA,DA	0.96	0.15	-0.19	-	-	0.93	0.16	-0.11	-	-	0.79	0.24	0.97	-	-
4WQY	AA,BA	0.97	0.17	0.07	-	-	0.93	0.18	0.23	-	-	0.84	0.24	0.98	-	-
4WQY	CA,DA	0.95	0.18	0.22	-	-	0.93	0.18	0.24	-	-	0.79	0.28	1.56	-	-
4WR6	1H,13	0.97	0.20	0.12	-	-	0.95	0.19	0.08	-	-	0.93	0.19	0.05	-	-
4WR6	14,1G	0.96	0.19	0.07	-	-	0.94	0.19	0.04	-	-	0.88	0.21	0.27	-	-
4WRA	1H,13	0.97	0.20	0.18	-	-	0.95	0.20	0.18	-	-	0.94	0.20	0.16	-	-
4WRA	14,1G	0.96	0.20	0.14	-	-	0.95	0.19	0.11	-	-	0.91	0.20	0.15	-	-
4WRO	1H,13	-	-	-	-	-	-	-	-	-	-	-	-	-	-	-
4WRO	14,1G	-	-	-	-	-	-	-	-	-	-	-	-	-	-	-
4WSD	1H,13	0.97	0.20	0.46	-	-	0.95	0.19	0.37	-	-	0.94	0.18	0.23	-	-
4WSD	14,1G	0.96	0.19	0.38	-	-	0.95	0.19	0.35	-	-	0.87	0.21	0.65	-	-
4WSM	1H,13	0.96	0.15	-0.39	-	-	0.95	0.14	-0.52	-	-	0.93	0.12	-0.81	-	-
4WSM	14,1G	0.96	0.16	-0.36	-	-	0.95	0.14	-0.58	-	-	0.91	0.12	-0.84	-	-
4WT1	1H,13	0.96	0.21	0.35	-	-	0.94	0.21	0.27	-	-	0.93	0.21	0.27	-	-
4WT1	14,1G	0.95	0.21	0.26	-	-	0.93	0.20	0.17	-	-	0.87	0.23	0.52	-	-
4WT8	C1,Ab	0.96	0.16	-0.32	-	-	0.95	0.16	-0.32	-	-	0.92	0.18	-0.09	-	-
4WT8	D1,Bb	0.96	0.16	-0.31	-	-	0.95	0.16	-0.27	-	-	0.91	0.19	0.06	-	-
4WU1	1H,13	0.96	0.21	0.35	-	-	0.94	0.20	0.23	-	-	0.91	0.20	0.20	-	-
4WU1	14,1G	0.96	0.21	0.31	-	-	0.94	0.21	0.25	-	-	0.90	0.20	0.23	-	-
4WWW	RA,QA	0.97	0.15	-0.50	-	-	0.95	0.14	-0.52	-	-	0.93	0.15	-0.45	-	-
4WWW	YA,XA	0.87	0.19	0.09	-	-	0.93	0.16	-0.33	-	-	0.85	0.19	0.01	-	-
4WZD	1H,13	0.96	0.16	-0.30	-	-	0.94	0.14	-0.54	-	-	0.91	0.14	-0.60	-	-
4WZD	14,1G	0.96	0.15	-0.46	-	-	0.95	0.14	-0.54	-	-	0.89	0.14	-0.60	-	-
4WZO	1H,13	0.96	0.16	-0.32	-	-	0.94	0.15	-0.45	-	-	0.94	0.12	-0.76	-	-
4WZO	14,1G	0.95	0.16	-0.36	-	-	0.94	0.14	-0.59	-	-	0.90	0.12	-0.77	-	-
4XEJ	A23S,A16S	0.92	0.20	0.05	-	-	0.92	0.19	0.00	-	-	0.82	0.23	0.49	-	-
4XEJ	B23S,B16S	0.93	0.19	0.04	-	-	0.90	0.19	0.03	-	-	0.83	0.23	0.48	-	-
4Y4O	1A,1a	0.97	0.19	0.88 (2109/2120)	-	-	0.96	0.15	0.27 (847/856)	-	-	0.94	0.15	0.25 (415/418)	-	-
4Y4O	2A,2a	0.97	0.15	0.19 (2136/2147)	-	-	0.95	0.15	0.16 (871/880)	-	-	0.89	0.18	0.66 (415/418)	-	-
4Y4P	1A,1a	-	-	-	-	-	-	-	-	-	-	-	-	-	-	-
4Y4P	2A,2a	-	-	-	-	-	-	-	-	-	-	-	-	-	-	-
4YBB	DA,AA	0.94	0.20	0.18 (2654/2677)	-	-	0.93	0.17	-0.22 (908/916)	-	-	0.88	0.18	-0.04 (390/393)	-	-
4YBB	CA,BA	0.74	0.25	0.76 (2745/2767)	-	-	0.83	0.21	0.25 (1024/1032)	-	-	0.63	0.30	1.33 (429/432)	-	-
4YPB	RA,QA	0.95	0.17	-0.21	-	-	0.95	0.17	-0.25	-	-	0.89	0.20	0.20	-	-
4YPB	YA,XA	0.95	0.17	-0.24	-	-	0.95	0.17	-0.21	-	-	0.92	0.19	0.00	-	-
4YZV	RA,QA	0.96	0.22	0.43	-	-	0.95	0.23	0.51	-	-	0.90	0.27	1.01	-	-
4YZV	YA,XA	0.96	0.22	0.45	-	-	0.95	0.23	0.51	-	-	0.93	0.24	0.61	-	-
4Z3S	1A,1a	0.98	0.19	0.23 (2083/2094)	-	-	0.95	0.17	-0.08 (849/858)	-	-	0.94	0.17	-0.09 (410/413)	-	-
4Z3S	2A,2a	0.96	0.16	-0.31 (2112/2123)	-	-	0.94	0.16	-0.34 (880/889)	-	-	0.87	0.17	-0.07 (414/417)	-	-
4Z8C	2A,2a	0.96	0.19	0.30	-	-	0.95	0.19	0.37	-	-	0.89	0.20	0.50	-	-
4Z8C	1A,1a	0.98	0.22	0.78	-	-	0.95	0.19	0.38	-	-	0.93	0.19	0.28	-	-
4ZER	2A,2a	0.95	0.17	-0.25 (2129/2140)	-	-	0.94	0.16	-0.35 (882/891)	-	-	0.88	0.21	0.24 (414/417)	-	-

Table 9 of 27

PDB		LSU					SSU BODY					SSU HEAD				
ID	chains	⟨rscc⟩	⟨rsr⟩	⟨rsrz⟩	⟨Q_score⟩	⟨inclusion⟩	⟨rscc⟩	⟨rsr⟩	⟨rsrz⟩	⟨Q_score⟩	⟨inclusion⟩	⟨rscc⟩	⟨rsr⟩	⟨rsrz⟩	⟨Q_score⟩	⟨inclusion⟩
4ZER	1A,1a	0.97	0.17	-0.17 (2082/2093)	-	-	0.94	0.17	-0.26 (867/876)	-	-	0.91	0.18	-0.07 (411/414)	-	-
4ZSN	RA,QA	0.95	0.19	-0.05	-	-	0.94	0.18	-0.10	-	-	0.90	0.21	0.24	-	-
4ZSN	YA,XA	0.95	0.19	-0.07	-	-	0.95	0.19	-0.08	-	-	0.93	0.19	-0.02	-	-
5A9Z	AA,BA	-	-	-	-	0.93	-	-	-	-	0.91	-	-	-	-	0.91
5AA0	AA,BA	-	-	-	-	0.97	-	-	-	-	0.96	-	-	-	-	0.97
5AFI	A,a	-	-	-	-	0.90 (2613/2624)	-	-	-	-	0.89 (986/990)	-	-	-	-	0.86 (443/446)
5AJ0	A2,B1	-	-	-	-	0.00	-	-	-	-	0.00	-	-	-	-	0.00
5AJ4	BA,AA	-	-	-	-	0.97	-	-	-	-	0.97	-	-	-	-	0.94
5CZP	YA,XA	0.94	0.22	0.38 (2151/2162)	-	-	0.92	0.19	0.00 (886/895)	-	-	0.90	0.20	0.13 (413/416)	-	-
5CZP	RA,QA	0.94	0.20	0.21 (2162/2173)	-	-	0.92	0.18	-0.04 (874/883)	-	-	0.88	0.20	0.22 (415/418)	-	-
5D8B	VC,UC	0.91	0.21	0.28	-	-	0.91	0.21	0.25	-	-	0.83	0.23	0.45	-	-
5D8B	ZC,YC	0.93	0.21	0.25	-	-	0.87	0.24	0.63	-	-	0.82	0.25	0.69	-	-
5DAT	5,6	0.97	0.23	0.54	-	-	0.95	0.23	0.55	-	-	0.93	0.24	0.66	-	-
5DAT	1,2	0.96	0.23	0.50	-	-	0.94	0.25	0.75	-	-	0.92	0.25	0.75	-	-
5DC3	5,6	0.95	0.26	0.96	-	-	0.94	0.25	0.76	-	-	0.91	0.23	0.53	-	-
5DC3	1,2	0.95	0.26	0.94	-	-	0.91	0.26	0.92	-	-	0.90	0.25	0.73	-	-
5DFE	YA,XA	0.97	0.17	-0.17 (2139/2150)	-	-	0.95	0.18	-0.12 (882/891)	-	-	0.91	0.20	0.12 (414/417)	-	-
5DFE	RA,QA	0.96	0.17	-0.16 (2149/2159)	-	-	0.95	0.18	-0.12 (867/876)	-	-	0.90	0.20	0.12 (414/417)	-	-
5DGE	1,2	0.96	0.20	0.16	-	-	0.93	0.20	0.14	-	-	0.92	0.21	0.25	-	-
5DGE	5,6	0.97	0.20	0.17	-	-	0.95	0.20	0.13	-	-	0.92	0.21	0.23	-	-
5DGF	5,6	0.95	0.21	0.26	-	-	0.93	0.21	0.25	-	-	0.90	0.21	0.26	-	-
5DGF	1,2	0.95	0.21	0.24	-	-	0.91	0.22	0.36	-	-	0.89	0.21	0.24	-	-
5DGV	1,2	-	-	-	-	-	-	-	-	-	-	-	-	-	-	-
5DGV	5,6	-	-	-	-	-	-	-	-	-	-	-	-	-	-	-
5DOX	1A,1a	0.96	0.27	1.03 (2091/2102)	-	-	0.91	0.26	0.96 (850/859)	-	-	0.78	0.47	3.54 (408/411)	-	-
5DOX	2A,2a	0.95	0.24	0.63 (2145/2156)	-	-	0.91	0.26	0.95 (879/888)	-	-	0.78	0.42	2.91 (409/412)	-	-
5DOY	1A,1a	0.97	0.21	0.60 (2064/2075)	-	-	0.95	0.19	0.28 (854/863)	-	-	0.93	0.19	0.17 (411/414)	-	-
5DOY	2A,2a	0.96	0.18	0.06 (2092/2103)	-	-	0.94	0.18	-0.04 (878/887)	-	-	0.88	0.19	0.14 (412/415)	-	-
5E7K	14,1G	0.95	0.16	-0.36	-	-	0.94	0.14	-0.60	-	-	0.90	0.12	-0.77	-	-
5E7K	1H,13	0.96	0.15	-0.40	-	-	0.94	0.14	-0.57	-	-	0.94	0.12	-0.82	-	-
5E81	1H,13	0.97	0.17	-0.02	-	-	0.95	0.16	-0.11	-	-	0.94	0.14	-0.44	-	-
5E81	14,1G	0.96	0.16	-0.07	-	-	0.95	0.15	-0.24	-	-	0.89	0.15	-0.28	-	-
5EL4	14,1G	0.96	0.16	-0.29	-	-	0.94	0.15	-0.50	-	-	0.89	0.13	-0.71	-	-
5EL4	1H,13	0.97	0.16	-0.31	-	-	0.95	0.16	-0.35	-	-	0.94	0.12	-0.78	-	-
5EL5	14,1G	0.95	0.16	-0.33	-	-	0.94	0.14	-0.51	-	-	0.88	0.17	-0.18	-	-
5EL5	1H,13	0.96	0.16	-0.36	-	-	0.94	0.14	-0.52	-	-	0.93	0.12	-0.87	-	-
5EL6	14,1G	0.96	0.15	-0.47	-	-	0.95	0.13	-0.65	-	-	0.90	0.11	-0.93	-	-
5EL6	1H,13	0.96	0.16	-0.36	-	-	0.95	0.14	-0.60	-	-	0.95	0.11	-0.90	-	-
5EL7	14,1G	0.96	0.14	-0.58	-	-	0.95	0.12	-0.79	-	-	0.91	0.11	-1.00	-	-
5EL7	1H,13	0.97	0.14	-0.58	-	-	0.95	0.13	-0.71	-	-	0.94	0.11	-1.00	-	-
5F8K	1A,1a	-	-	-	-	-	-	-	-	-	-	-	-	-	-	-
5F8K	2A,2a	-	-	-	-	-	-	-	-	-	-	-	-	-	-	-
5FCI	5,6	0.95	0.18	-0.12	-	-	0.93	0.19	0.05	-	-	0.90	0.21	0.32	-	-
5FCI	1,2	0.95	0.18	-0.11	-	-	0.91	0.20	0.20	-	-	0.89	0.22	0.42	-	-
5FCJ	5,6	0.96	0.17	-0.15	-	-	0.95	0.19	-0.02	-	-	0.92	0.21	0.27	-	-
5FCJ	1,2	0.96	0.18	-0.12	-	-	0.93	0.19	0.04	-	-	0.91	0.21	0.24	-	-

PDB		LSU					SSU BODY					SSU HEAD				
ID	chains	⟨rscc⟩	⟨rsr⟩	⟨rsrz⟩	⟨Q_score⟩	⟨inclusion⟩	⟨rscc⟩	⟨rsr⟩	⟨rsrz⟩	⟨Q_score⟩	⟨inclusion⟩	⟨rscc⟩	⟨rsr⟩	⟨rsrz⟩	⟨Q_score⟩	⟨inclusion⟩
5FDU	1A,1a	0.98	0.15	-0.19 (2102/2112)	-	-	0.96	0.16	-0.16 (858/867)	-	-	0.93	0.16	-0.16 (407/410)	-	-
5FDU	2A,2a	0.97	0.15	-0.18 (2133/2144)	-	-	0.96	0.15	-0.22 (881/890)	-	-	0.92	0.17	0.06 (416/419)	-	-
5FDV	1A,1a	-	-	-	-	-	-	-	-	-	-	-	-	-	-	-
5FDV	2A,2a	-	-	-	-	-	-	-	-	-	-	-	-	-	-	-
5H5U	A,h	-	-	-	-	1.00	-	-	-	-	1.00	-	-	-	-	1.00
5HAU	1A,1a	0.98	0.16	-0.27	-	-	0.95	0.16	-0.26	-	-	0.93	0.18	-0.09	-	-
5HAU	2A,2a	0.97	0.14	-0.51	-	-	0.95	0.16	-0.34	-	-	0.90	0.19	0.07	-	-
5HCP	2A,2a	0.96	0.14	-0.35	-	-	0.95	0.16	-0.13	-	-	0.89	0.20	0.48	-	-
5HCP	1A,1a	0.98	0.17	0.01	-	-	0.95	0.16	-0.07	-	-	0.93	0.17	0.07	-	-
5HCQ	2A,2a	0.97	0.13	-0.47	-	-	0.95	0.15	-0.26	-	-	0.89	0.19	0.28	-	-
5HCQ	1A,1a	0.98	0.16	-0.05	-	-	0.96	0.15	-0.22	-	-	0.94	0.16	-0.14	-	-
5HCR	2A,2a	0.96	0.14	-0.44	-	-	0.95	0.15	-0.24	-	-	0.89	0.19	0.26	-	-
5HCR	1A,1a	0.98	0.16	-0.06	-	-	0.95	0.15	-0.22	-	-	0.93	0.16	-0.13	-	-
5HD1	2A,2a	0.96	0.14	-0.72	-	-	0.94	0.15	-0.42	-	-	0.88	0.20	0.32	-	-
5HD1	1A,1a	0.97	0.16	-0.24	-	-	0.95	0.16	-0.40	-	-	0.93	0.17	-0.22	-	-
5I4L	1,2	-	-	-	-	-	-	-	-	-	-	-	-	-	-	-
5I4L	5,6	-	-	-	-	-	-	-	-	-	-	-	-	-	-	-
5IB7	1H,13	0.97	0.16	-0.04	-	-	0.95	0.16	-0.09	-	-	0.94	0.14	-0.46	-	-
5IB7	14,1G	0.96	0.17	-0.02	-	-	0.94	0.16	-0.14	-	-	0.88	0.15	-0.18	-	-
5IB8	1H,13	0.97	0.17	-0.26	-	-	0.94	0.15	-0.40	-	-	0.93	0.13	-0.73	-	-
5IB8	14,1G	0.96	0.17	-0.24	-	-	0.95	0.15	-0.41	-	-	0.89	0.14	-0.58	-	-
5IBB	1H,13	0.97	0.16	-0.09	-	-	0.95	0.14	-0.36	-	-	0.93	0.13	-0.52	-	-
5IBB	14,1G	0.96	0.16	-0.11	-	-	0.95	0.14	-0.41	-	-	0.90	0.13	-0.57	-	-
5IMQ	D,A	-	-	-	-	0.91	-	-	-	-	0.88	-	-	-	-	0.84
5IMR	D,A	-	-	-	-	0.73	-	-	-	-	0.76	-	-	-	-	0.62
5IQR	1,2	-	-	-	-	0.93 (2517/2527)	-	-	-	-	0.91 (971/974)	-	-	-	-	0.91 (432/435)
5IT7	5,2	-	-	-	-	0.89	-	-	-	-	0.83	-	-	-	-	0.77
5IT8	DA,AA	0.97	0.17	-0.25 (2639/2662)	-	-	0.95	0.17	-0.26 (913/921)	-	-	0.91	0.19	-0.00 (393/396)	-	-
5IT8	CA,BA	0.77	0.27	0.99 (2742/2764)	-	-	0.87	0.20	0.21 (1022/1030)	-	-	0.78	0.25	0.78 (429/432)	-	-
5J30	RA,QA	-	-	-	-	-	-	-	-	-	-	-	-	-	-	-
5J30	YA,XA	-	-	-	-	-	-	-	-	-	-	-	-	-	-	-
5J3C	RA,QA	-	-	-	-	-	-	-	-	-	-	-	-	-	-	-
5J3C	YA,XA	-	-	-	-	-	-	-	-	-	-	-	-	-	-	-
5J4B	1A,1a	0.98	0.20	0.44 (2071/2082)	-	-	0.96	0.17	-0.07 (831/840)	-	-	0.94	0.16	-0.37 (412/415)	-	-
5J4B	2A,2a	0.96	0.17	-0.19 (2097/2108)	-	-	0.95	0.17	-0.13 (874/883)	-	-	0.88	0.16	-0.35 (407/410)	-	-
5J4C	1A,1a	0.97	0.19	0.28 (2069/2080)	-	-	0.96	0.17	0.04 (838/847)	-	-	0.95	0.16	-0.17 (410/413)	-	-
5J4C	2A,2a	0.96	0.15	-0.20 (2102/2113)	-	-	0.95	0.16	-0.09 (880/889)	-	-	0.90	0.19	0.35 (407/410)	-	-
5J4D	B,A	0.96	0.20	0.21 (2105/2116)	-	-	0.94	0.21	0.25 (883/892)	-	-	0.91	0.23	0.58 (397/400)	-	-
5J4D	GB,FB	0.94	0.21	0.31 (2112/2123)	-	-	0.94	0.20	0.23 (882/891)	-	-	0.88	0.24	0.71 (398/401)	-	-
5J5B	DA,AA	0.96	0.19	0.29 (2654/2677)	-	-	0.94	0.18	0.19 (913/921)	-	-	0.87	0.22	0.72 (389/392)	-	-
5J5B	CA,BA	0.75	0.28	1.64 (2746/2768)	-	-	0.84	0.22	0.81 (1022/1030)	-	-	0.67	0.33	2.37 (430/433)	-	-
5J7L	DA,AA	0.97	0.17	-0.24 (2642/2663)	-	-	0.96	0.16	-0.30 (909/917)	-	-	0.88	0.21	0.31 (391/394)	-	-

Table 11 of 27

PDB		LSU					SSU BODY					SSU HEAD				
ID	chains	⟨rscc⟩	⟨rsr⟩	⟨rsrz⟩	⟨Q_score⟩	⟨inclusion⟩	⟨rscc⟩	⟨rsr⟩	⟨rsrz⟩	⟨Q_score⟩	⟨inclusion⟩	⟨rscc⟩	⟨rsr⟩	⟨rsrz⟩	⟨Q_score⟩	⟨inclusion⟩
5J7L	CA,BA	0.86	0.23	0.53 (2745/2768)	-	-	0.91	0.19	0.06 (1021/1029)	-	-	0.77	0.29	1.22 (432/435)	-	-
5J88	DA,AA	0.97	0.18	-0.05 (2642/2664)	-	-	0.94	0.18	-0.03 (905/913)	-	-	0.79	0.27	1.05 (389/392)	-	-
5J88	CA,BA	0.85	0.23	0.54 (2735/2757)	-	-	0.91	0.19	0.04 (1017/1025)	-	-	0.64	0.39	2.53 (420/423)	-	-
5J8A	DA,AA	0.97	0.18	-0.07 (2643/2665)	-	-	0.96	0.17	-0.15 (899/907)	-	-	0.88	0.23	0.54 (390/393)	-	-
5J8A	CA,BA	0.87	0.23	0.59 (2745/2767)	-	-	0.92	0.19	0.06 (1023/1031)	-	-	0.76	0.31	1.50 (430/433)	-	-
5J8B	A,a	0.96	0.17	-0.06	-	-	0.95	0.18	0.00	-	-	0.95	0.18	0.06	-	-
5J91	DA,AA	0.96	0.20	0.47 (2654/2677)	-	-	0.94	0.19	0.39 (913/921)	-	-	0.85	0.24	1.08 (389/392)	-	-
5J91	CA,BA	0.76	0.29	1.80 (2746/2768)	-	-	0.85	0.23	0.88 (1022/1030)	-	-	0.67	0.36	2.74 (430/433)	-	-
5JC9	DA,AA	0.97	0.17	-0.16 (2636/2657)	-	-	0.96	0.17	-0.22 (895/903)	-	-	0.88	0.21	0.35 (388/391)	-	-
5JC9	CA,BA	0.86	0.23	0.53 (2747/2769)	-	-	0.87	0.21	0.29 (1023/1031)	-	-	0.82	0.25	0.74 (435/438)	-	-
5JTE	BA,AA	-	-	-	-	0.47	-	-	-	-	0.34	-	-	-	-	0.24
5JU8	BA,AA	-	-	-	-	0.69	-	-	-	-	0.65	-	-	-	-	0.57
5JUO	B,A	-	-	-	-	0.74	-	-	-	-	0.62	-	-	-	-	0.45
5JUP	B,A	-	-	-	-	0.91	-	-	-	-	0.75	-	-	-	-	0.74
5JUS	B,A	-	-	-	-	0.75	-	-	-	-	0.57	-	-	-	-	0.54
5JUT	B,A	-	-	-	-	0.84	-	-	-	-	0.77	-	-	-	-	0.65
5JUU	B,A	-	-	-	-	0.86	-	-	-	-	0.83	-	-	-	-	0.81
5KCR	1A,1a	-	-	-	-	0.86 (2669/2678)	-	-	-	-	0.85	-	-	-	-	0.84
5KCS	1A,1a	-	-	-	-	0.72 (2612/2622)	-	-	-	-	0.64 (976/980)	-	-	-	-	0.63 (442/445)
5KPS	28,27	-	-	-	-	1.00	-	-	-	-	1.00	-	-	-	-	1.00
5KPV	27,26	-	-	-	-	0.99	-	-	-	-	0.99	-	-	-	-	0.99
5KPW	27,26	-	-	-	-	0.99	-	-	-	-	0.99	-	-	-	-	0.99
5KPX	27,26	-	-	-	-	1.00	-	-	-	-	0.99	-	-	-	-	1.00
5L3P	A,a	-	-	-	-	0.47 (2606/2617)	-	-	-	-	0.45 (975/979)	-	-	-	-	0.59 (433/436)
5LI0	A,a	-	-	-	-	0.80	-	-	-	-	0.74	-	-	-	-	0.64
5LKS	L5,S2	-	-	-	-	0.96	-	-	-	-	0.96	-	-	-	-	0.93
5LYB	1,2	0.96	0.21	0.28	-	-	0.94	0.20	0.11	-	-	0.92	0.19	0.04	-	-
5LYB	5,6	0.96	0.21	0.32	-	-	0.95	0.21	0.27	-	-	0.93	0.17	-0.24	-	-
5LZA	A,a	-	-	-	-	0.91 (2607/2617)	-	-	-	-	0.90 (983/989)	-	-	-	-	0.88 (436/439)
5LZB	A,a	-	-	-	-	0.93 (2584/2593)	-	-	-	-	0.92 (964/967)	-	-	-	-	0.90 (429/431)
5LZC	A,a	-	-	-	-	0.93 (2571/2580)	-	-	-	-	0.93 (972/975)	-	-	-	-	0.91 (429/432)
5LZD	A,a	-	-	-	-	0.91 (2599/2608)	-	-	-	-	0.90 (974/977)	-	-	-	-	0.89 (436/439)
5LZE	A,a	-	-	-	-	0.92 (2611/2621)	-	-	-	-	0.90 (950/953)	-	-	-	-	0.89 (437/440)
5LZF	A,a	-	-	-	-	0.94 (2503/2512)	-	-	-	-	0.92 (924/927)	-	-	-	-	0.90 (414/417)
5LZS	5,9	-	-	-	-	0.89	-	-	-	-	0.87	-	-	-	-	0.86
5LZT	5,9	-	-	-	-	0.88	-	-	-	-	0.88	-	-	-	-	0.87
5LZU	5,9	-	-	-	-	0.86	-	-	-	-	0.85	-	-	-	-	0.85
5LZV	5,9	-	-	-	-	0.92	-	-	-	-	0.92	-	-	-	-	0.92

PDB		LSU					SSU BODY					SSU HEAD				
ID	chains	⟨rscc⟩	⟨rsr⟩	⟨rsrz⟩	⟨Q_score⟩	⟨inclusion⟩	⟨rscc⟩	⟨rsr⟩	⟨rsrz⟩	⟨Q_score⟩	⟨inclusion⟩	⟨rscc⟩	⟨rsr⟩	⟨rsrz⟩	⟨Q_score⟩	⟨inclusion⟩
5LZW	5,9	-	-	-	-	0.89	-	-	-	-	0.88	-	-	-	-	0.87
5LZX	5,9	-	-	-	-	0.50	-	-	-	-	0.42	-	-	-	-	0.38
5LZY	5,9	-	-	-	-	0.89	-	-	-	-	0.88	-	-	-	-	0.87
5LZZ	5,9	-	-	-	-	0.93	-	-	-	-	0.93	-	-	-	-	0.93
5M1J	14,22	-	-	-	-	0.90	-	-	-	-	0.90	-	-	-	-	0.88
5MC6	BQ,2	-	-	-	-	0.87	-	-	-	-	0.82	-	-	-	-	0.84
5MDV	1,2	-	-	-	-	0.99 (2663/2673)	-	-	-	-	0.99 (999/1003)	-	-	-	-	0.99 (437/440)
5MDW	1,2	-	-	-	-	0.98 (2641/2651)	-	-	-	-	0.98 (1000/1004)	-	-	-	-	0.98 (435/438)
5MDY	1,2	-	-	-	-	0.95 (2645/2654)	-	-	-	-	0.95 (998/1002)	-	-	-	-	0.94 (434/437)
5MDZ	1,2	-	-	-	-	0.97 (2643/2653)	-	-	-	-	0.96 (999/1003)	-	-	-	-	0.94 (436/439)
5MEI	1,A	0.97	0.17	-0.29	-	-	0.95	0.17	-0.23	-	-	0.94	0.16	-0.37	-	-
5MEI	AR,6	0.97	0.17	-0.27	-	-	0.96	0.17	-0.25	-	-	0.94	0.16	-0.34	-	-
5MGP	A,a	-	-	-	-	0.84	-	-	-	-	0.84	-	-	-	-	0.79
5MMM	A,a	-	-	-	-	0.98	-	-	-	-	0.98	-	-	-	-	0.97
5MRC	A,aa	-	-	-	-	0.88	-	-	-	-	0.80	-	-	-	-	0.52
5MRE	A,aa	-	-	-	-	0.99	-	-	-	-	0.99	-	-	-	-	0.99
5MRF	A,aa	-	-	-	-	0.99	-	-	-	-	0.99	-	-	-	-	0.99
5MYJ	BA,AA	-	-	-	-	0.98	-	-	-	-	0.95	-	-	-	-	0.96
5ND8	A,a	-	-	-	-	0.94	-	-	-	-	0.92	-	-	-	-	0.83
5ND9	A,a	-	-	-	-	0.91	-	-	-	-	0.88	-	-	-	-	0.74
5NDG	5,6	0.95	0.21	0.27	-	-	0.92	0.20	0.09	-	-	0.88	0.21	0.15	-	-
5NDG	1,2	0.94	0.21	0.24	-	-	0.91	0.20	0.13	-	-	0.93	0.18	-0.16	-	-
5NDJ	1H,13	0.96	0.21	0.34	-	-	0.94	0.21	0.36	-	-	0.93	0.25	0.81	-	-
5NDJ	14,1G	0.95	0.22	0.37	-	-	0.95	0.21	0.31	-	-	0.90	0.25	0.77	-	-
5NDK	1H,13	0.97	0.17	-0.01	-	-	0.95	0.16	-0.17	-	-	0.93	0.15	-0.23	-	-
5NDK	14,1G	0.96	0.16	-0.08	-	-	0.95	0.16	-0.13	-	-	0.90	0.14	-0.33	-	-
5NDV	5,6	0.96	0.21	0.26	-	-	0.93	0.20	0.18	-	-	0.90	0.20	0.14	-	-
5NDV	1,2	0.97	0.21	0.26	-	-	0.93	0.20	0.16	-	-	0.90	0.21	0.30	-	-
5NDW	1,2	0.95	0.21	0.21	-	-	0.93	0.18	-0.11	-	-	0.92	0.16	-0.34	-	-
5NDW	5,6	0.95	0.21	0.16	-	-	0.93	0.19	-0.09	-	-	0.91	0.16	-0.38	-	-
5NGM	AA,Aa	-	-	-	-	0.96	-	-	-	-	0.83	-	-	-	-	0.47
5NJT	U,A	-	-	-	-	0.86	-	-	-	-	0.85	-	-	-	-	0.82
5NP6	Y,D	-	-	-	-	1.00 (2348/2358)	-	-	-	-	1.00 (905/909)	-	-	-	-	1.00 (413/416)
5NWY	N,0	-	-	-	-	0.96	-	-	-	-	0.95	-	-	-	-	0.93
5O2R	A,a	-	-	-	-	0.76	-	-	-	-	0.74	-	-	-	-	0.71
5O61	A,BA	-	-	-	-	-	-	-	-	-	-	-	-	-	-	-
5OBM	5,6	0.97	0.20	0.20	-	-	0.95	0.19	0.01	-	-	0.96	0.16	-0.27	-	-
5OBM	1,2	0.96	0.20	0.10	-	-	0.93	0.18	-0.04	-	-	0.91	0.18	-0.13	-	-
5ON6	1,A	0.96	0.20	0.15	-	-	0.94	0.20	0.19	-	-	0.93	0.20	0.12	-	-
5ON6	AR,6	0.96	0.21	0.23	-	-	0.95	0.20	0.15	-	-	0.93	0.20	0.22	-	-
5OT7	4,1	-	-	-	-	0.94	-	-	-	-	0.93	-	-	-	-	0.84
5T2C	A,AA	-	-	-	-	0.89	-	-	-	-	0.74	-	-	-	-	0.39
5T7V	B,A	-	-	-	-	0.97	-	-	-	-	0.91	-	-	-	-	0.74
5TBW	1,A	0.97	0.18	-0.08	-	-	0.95	0.18	-0.13	-	-	0.95	0.17	-0.24	-	-
5TBW	AR,sR	0.97	0.18	-0.06	-	-	0.96	0.18	-0.10	-	-	0.94	0.17	-0.17	-	-
5TCU	B,A	-	-	-	-	0.87	-	-	-	-	0.81	-	-	-	-	0.71
5TGA	1,2	0.96	0.20	0.19	-	-	0.94	0.19	0.07	-	-	0.93	0.21	0.35	-	-
5TGA	5,6	0.96	0.21	0.24	-	-	0.96	0.19	0.09	-	-	0.94	0.21	0.25	-	-
5TGM	1,2	-	-	-	-	-	-	-	-	-	-	-	-	-	-	-
5TGM	5,6	-	-	-	-	-	-	-	-	-	-	-	-	-	-	-
5U4I	A,a	-	-	-	-	0.55 (2458/2464)	-	-	-	-	0.48 (864/867)	-	-	-	-	0.45 (415/418)

PDB		LSU					SSU BODY					SSU HEAD				
ID	chains	⟨rscc⟩	⟨rsr⟩	⟨rsrz⟩	⟨Q_score⟩	⟨inclusion⟩	⟨rscc⟩	⟨rsr⟩	⟨rsrz⟩	⟨Q_score⟩	⟨inclusion⟩	⟨rscc⟩	⟨rsr⟩	⟨rsrz⟩	⟨Q_score⟩	⟨inclusion⟩
5U9F	01,A	-	-	-	-	0.99	-	-	-	-	0.98	-	-	-	-	0.98
5U9G	01,A	-	-	-	-	0.99	-	-	-	-	0.99	-	-	-	-	0.99
5UQ7	A,a	-	-	-	-	0.62 (1936/1943)	-	-	-	-	0.59 (810/815)	-	-	-	-	0.50 (402/404)
5UQ8	A,a	-	-	-	-	0.80 (1939/1946)	-	-	-	-	0.76 (818/823)	-	-	-	-	0.71 (402/404)
5UYK	01,A	-	-	-	-	0.96	-	-	-	-	0.95	-	-	-	-	0.95
5UYL	01,A	-	-	-	-	0.96	-	-	-	-	0.96	-	-	-	-	0.96
5UYM	01,A	-	-	-	-	0.98	-	-	-	-	0.98	-	-	-	-	0.98
5UYN	01,A	-	-	-	-	0.94	-	-	-	-	0.93	-	-	-	-	0.92
5UYP	01,A	-	-	-	-	0.95	-	-	-	-	0.94	-	-	-	-	0.93
5UYQ	01,A	-	-	-	-	0.95	-	-	-	-	0.94	-	-	-	-	0.94
5V8I	1A,1a	0.97	0.15	-0.39 (2114/2122)	-	-	0.93	0.17	-0.19 (846/855)	-	-	0.91	0.18	-0.07 (415/418)	-	-
5V8I	2A,2a	0.95	0.16	-0.29 (2167/2178)	-	-	0.93	0.17	-0.20 (862/871)	-	-	0.84	0.22	0.40 (416/419)	-	-
5V93	A,a	-	-	-	-	0.76	-	-	-	-	0.58	-	-	-	-	0.25
5VP2	1A,1a	0.97	0.21	0.64 (2097/2108)	-	-	0.95	0.20	0.47 (843/852)	-	-	0.93	0.20	0.42 (412/415)	-	-
5VP2	2A,2a	0.95	0.20	0.51 (2099/2110)	-	-	0.93	0.20	0.48 (878/887)	-	-	0.87	0.22	0.77 (405/408)	-	-
5VPO	YA,XA	0.96	0.16	-0.32	-	-	0.92	0.18	-0.04	-	-	0.81	0.25	0.84	-	-
5VPO	RA,QA	0.95	0.17	-0.19	-	-	0.92	0.19	0.02	-	-	0.79	0.28	1.15	-	-
5VPP	YA,XA	-	-	-	-	-	-	-	-	-	-	-	-	-	-	-
5VPP	RA,QA	-	-	-	-	-	-	-	-	-	-	-	-	-	-	-
5W4K	1A,1a	0.98	0.21	0.62 (2086/2097)	-	-	0.96	0.20	0.42 (847/856)	-	-	0.94	0.19	0.21 (408/411)	-	-
5W4K	2A,2a	0.96	0.20	0.46 (2102/2113)	-	-	0.94	0.20	0.36 (879/888)	-	-	0.88	0.21	0.62 (406/409)	-	-
5WDT	A,a	-	-	-	-	0.99 (2573/2583)	-	-	-	-	0.99 (959/963)	-	-	-	-	0.99 (417/420)
5WE4	A,a	-	-	-	-	1.00 (2589/2599)	-	-	-	-	0.99 (966/970)	-	-	-	-	0.99 (418/421)
5WE6	A,a	-	-	-	-	0.99 (2568/2578)	-	-	-	-	0.99 (936/940)	-	-	-	-	0.99 (404/407)
5WF0	A,a	-	-	-	-	0.99 (2583/2594)	-	-	-	-	0.99 (984/988)	-	-	-	-	0.99 (422/425)
5WFK	A,a	-	-	-	-	0.98 (2576/2587)	-	-	-	-	0.98 (978/984)	-	-	-	-	0.98 (423/426)
5WFS	A,a	-	-	-	-	1.00 (2586/2597)	-	-	-	-	0.99 (951/955)	-	-	-	-	0.99 (420/423)
5WIS	1A,1a	0.98	0.20	0.39 (2087/2098)	-	-	0.95	0.19	0.13 (846/855)	-	-	0.94	0.18	0.03 (416/419)	-	-
5WIS	2A,2a	0.96	0.19	0.24 (2100/2111)	-	-	0.94	0.19	0.18 (881/890)	-	-	0.87	0.21	0.50 (408/411)	-	-
5WIT	1A,1a	0.97	0.19	0.31 (2076/2087)	-	-	0.95	0.19	0.21 (845/854)	-	-	0.94	0.18	0.04 (415/418)	-	-
5WIT	2A,2a	0.95	0.19	0.22 (2090/2101)	-	-	0.94	0.18	0.02 (874/883)	-	-	0.87	0.22	0.74 (416/419)	-	-
5X8P	A,a	-	-	-	-	0.84	-	-	-	-	0.76	-	-	-	-	0.61
5ZEB	A,a	-	-	-	-	0.89	-	-	-	-	0.76	-	-	-	-	0.74
5ZEP	A,a	-	-	-	-	0.99	-	-	-	-	0.99	-	-	-	-	0.99
5ZLU	V,G	-	-	-	-	0.94	-	-	-	-	0.95	-	-	-	-	0.93
6B4V	B,A	-	-	-	-	-	-	-	-	-	-	-	-	-	-	-
6B4V	FB,EB	-	-	-	-	-	-	-	-	-	-	-	-	-	-	-
6BOH	B,A	-	-	-	-	-	-	-	-	-	-	-	-	-	-	-

Table 14 of 27

PDB		LSU					SSU BODY					SSU HEAD				
ID	chains	⟨rscc⟩	⟨rsr⟩	⟨rsrz⟩	⟨Q_score⟩	⟨inclusion⟩	⟨rscc⟩	⟨rsr⟩	⟨rsrz⟩	⟨Q_score⟩	⟨inclusion⟩	⟨rscc⟩	⟨rsr⟩	⟨rsrz⟩	⟨Q_score⟩	⟨inclusion⟩
6BOH	GB,FB	-	-	-	-	-	-	-	-	-	-	-	-	-	-	-
6BOK	B,A	-	-	-	-	-	-	-	-	-	-	-	-	-	-	-
6BOK	EB,DB	-	-	-	-	-	-	-	-	-	-	-	-	-	-	-
6BU8	01,A	-	-	-	-	0.99	-	-	-	-	0.99	-	-	-	-	0.99
6BUW	YA,XA	-	-	-	-	-	-	-	-	-	-	-	-	-	-	-
6BUW	RA,QA	-	-	-	-	-	-	-	-	-	-	-	-	-	-	-
6BY1	DA,BA	0.93	0.16	-0.43	-	-	0.91	0.17	-0.32	-	-	0.88	0.18	-0.14	-	-
6BY1	CA,AA	0.95	0.15	-0.55	-	-	0.92	0.16	-0.35	-	-	0.89	0.18	-0.17	-	-
6BZ6	YA,XA	-	-	-	-	-	-	-	-	-	-	-	-	-	-	-
6BZ6	RA,QA	-	-	-	-	-	-	-	-	-	-	-	-	-	-	-
6BZ7	YA,XA	-	-	-	-	-	-	-	-	-	-	-	-	-	-	-
6BZ7	RA,QA	-	-	-	-	-	-	-	-	-	-	-	-	-	-	-
6BZ8	YA,XA	-	-	-	-	-	-	-	-	-	-	-	-	-	-	-
6BZ8	RA,QA	-	-	-	-	-	-	-	-	-	-	-	-	-	-	-
6C4I	A,a	-	-	-	-	0.73 (2657/2667)	-	-	-	-	0.50 (997/1000)	-	-	-	-	0.28 (437/440)
6C5L	BA,AA	0.95	0.16	-0.35	-	-	0.94	0.15	-0.44	-	-	0.86	0.18	-0.11	-	-
6C5L	DA,CA	0.95	0.16	-0.31	-	-	0.92	0.16	-0.38	-	-	0.89	0.17	-0.26	-	-
6CAE	1A,1a	0.98	0.21	0.58 (2093/2104)	-	-	0.95	0.19	0.28 (865/874)	-	-	0.94	0.18	0.08 (416/419)	-	-
6CAE	2A,2a	0.96	0.20	0.35 (2117/2128)	-	-	0.94	0.19	0.18 (881/890)	-	-	0.86	0.21	0.52 (412/415)	-	-
6CFJ	1A,1a	0.98	0.17	0.08 (2085/2096)	-	-	0.96	0.17	-0.01 (850/859)	-	-	0.95	0.17	0.09 (419/422)	-	-
6CFJ	2A,2a	0.96	0.17	0.05 (2112/2123)	-	-	0.95	0.16	-0.04 (878/887)	-	-	0.90	0.20	0.47 (407/410)	-	-
6CFK	1A,1a	0.98	0.20	0.46 (2092/2102)	-	-	0.96	0.19	0.19 (840/849)	-	-	0.95	0.18	-0.03 (408/411)	-	-
6CFK	2A,2a	0.97	0.19	0.29 (2152/2163)	-	-	0.95	0.18	0.12 (872/881)	-	-	0.90	0.19	0.18 (407/410)	-	-
6CFL	1A,1a	0.98	0.20	0.33 (2084/2094)	-	-	0.96	0.18	0.01 (832/841)	-	-	0.94	0.17	-0.18 (410/413)	-	-
6CFL	2A,2a	0.96	0.18	0.09 (2144/2155)	-	-	0.95	0.18	-0.00 (877/886)	-	-	0.90	0.20	0.34 (412/415)	-	-
6CZR	1A,1a	0.96	0.21	0.34 (2096/2107)	-	-	0.91	0.22	0.41 (864/873)	-	-	0.70	0.39	2.51 (410/413)	-	-
6CZR	2A,2a	0.93	0.22	0.39 (2146/2157)	-	-	0.90	0.23	0.55 (874/883)	-	-	0.70	0.38	2.44 (406/409)	-	-
6D90	5,2	-	-	-	-	0.98	-	-	-	-	0.98	-	-	-	-	0.99
6D9J	5,2	-	-	-	-	0.99	-	-	-	-	0.99	-	-	-	-	0.99
6DNC	B,A	-	-	-	-	0.79	-	-	-	-	0.77	-	-	-	-	0.75
6DZI	A,h	-	-	-	-	0.99	-	-	-	-	0.98	-	-	-	-	0.96
6ENF	A,a	-	-	-	-	0.76	-	-	-	-	0.73	-	-	-	-	0.71
6ENJ	A,a	-	-	-	-	0.67	-	-	-	-	0.63	-	-	-	-	0.64
6ENU	A,a	-	-	-	-	0.83	-	-	-	-	0.82	-	-	-	-	0.82
6ERI	AA,BA	-	-	-	-	0.98	-	-	-	-	0.96	-	-	-	-	0.94
6FKR	1A,1a	0.97	0.16	-0.37 (2098/2109)	-	-	0.95	0.17	-0.26 (856/865)	-	-	0.92	0.18	-0.12 (412/415)	-	-
6FKR	2A,2a	0.96	0.16	-0.35 (2147/2158)	-	-	0.95	0.16	-0.27 (878/887)	-	-	0.90	0.19	0.01 (406/409)	-	-
6FXC	AA,Aa	-	-	-	-	0.90	-	-	-	-	0.87	-	-	-	-	0.87
6FXC	BA,Ba	-	-	-	-	0.90	-	-	-	-	0.87	-	-	-	-	0.87
6GAW	BA,AA	-	-	-	-	0.96	-	-	-	-	0.90	-	-	-	-	0.76
6GQ1	1,2	-	-	-	-	0.93	-	-	-	-	0.92	-	-	-	-	0.86
6GQB	1,2	-	-	-	-	0.92	-	-	-	-	0.91	-	-	-	-	0.86
6GQV	1,2	-	-	-	-	0.96	-	-	-	-	0.97	-	-	-	-	0.94

PDB		LSU					SSU BODY					SSU HEAD				
ID	chains	⟨rscc⟩	⟨rsr⟩	⟨rsrz⟩	⟨Q_score⟩	⟨inclusion⟩	⟨rscc⟩	⟨rsr⟩	⟨rsrz⟩	⟨Q_score⟩	⟨inclusion⟩	⟨rscc⟩	⟨rsr⟩	⟨rsrz⟩	⟨Q_score⟩	⟨inclusion⟩
6GSJ	1H,13	0.97	0.19	0.28	-	-	0.95	0.17	0.10	-	-	0.94	0.17	-0.03	-	-
6GSJ	14,1G	0.96	0.19	0.26	-	-	0.95	0.17	0.02	-	-	0.91	0.15	-0.18	-	-
6GSK	1H,13	0.96	0.19	0.06	-	-	0.94	0.19	0.06	-	-	0.93	0.18	-0.02	-	-
6GSK	14,1G	0.95	0.20	0.23	-	-	0.94	0.17	-0.21	-	-	0.89	0.17	-0.21	-	-
6GSL	1H,13	-	-	-	-	-	-	-	-	-	-	-	-	-	-	-
6GSL	14,1G	-	-	-	-	-	-	-	-	-	-	-	-	-	-	-
6GWT	A,a	-	-	-	-	0.81	-	-	-	-	0.82	-	-	-	-	0.80
6GXM	A,a	-	-	-	-	0.79	-	-	-	-	0.81	-	-	-	-	0.79
6GXN	A,a	-	-	-	-	0.78	-	-	-	-	0.78	-	-	-	-	0.77
6GXO	A,a	-	-	-	-	0.78	-	-	-	-	0.80	-	-	-	-	0.78
6GXP	A,a	-	-	-	-	0.74	-	-	-	-	0.58	-	-	-	-	0.21
6GZ3	A2,B1	-	-	-	-	0.86	-	-	-	-	0.82	-	-	-	-	0.79
6GZ4	A2,B1	-	-	-	-	0.87	-	-	-	-	0.85	-	-	-	-	0.81
6GZ5	A2,B1	-	-	-	-	0.88	-	-	-	-	0.86	-	-	-	-	0.78
6GZQ	A1,A2	-	-	-	-	0.95	-	-	-	-	0.95	-	-	-	-	0.93
6GZX	A1,A3	-	-	-	-	0.84	-	-	-	-	0.88	-	-	-	-	0.86
6GZX	A2,A4	-	-	-	-	0.84	-	-	-	-	0.87	-	-	-	-	0.85
6GZZ	A1,A3	-	-	-	-	0.91	-	-	-	-	0.96	-	-	-	-	0.93
6GZZ	A2,A4	-	-	-	-	0.91	-	-	-	-	0.96	-	-	-	-	0.93
6H4N	A,a	-	-	-	-	0.79	-	-	-	-	0.78	-	-	-	-	0.76
6H58	A,a	-	-	-	-	0.97	-	-	-	-	0.95	-	-	-	-	0.94
6H58	AA,aa	-	-	-	-	0.97	-	-	-	-	0.96	-	-	-	-	0.95
6HA1	A,a	-	-	-	-	0.96	-	-	-	-	0.96	-	-	-	-	0.94
6HA8	A,a	-	-	-	-	0.85	-	-	-	-	0.67	-	-	-	-	0.70
6HCF	52,A1	-	-	-	-	0.93	-	-	-	-	0.92	-	-	-	-	0.92
6HCJ	51,A2	-	-	-	-	0.86	-	-	-	-	0.79	-	-	-	-	0.66
6HCM	52,A1	-	-	-	-	0.77	-	-	-	-	0.82	-	-	-	-	0.84
6HCQ	51,A2	-	-	-	-	0.88	-	-	-	-	0.85	-	-	-	-	0.83
6HHQ	1,A	0.97	0.17	-0.17	-	-	0.95	0.18	-0.08	-	-	0.94	0.19	0.06	-	-
6HHQ	AR,sR	0.97	0.17	-0.16	-	-	0.96	0.18	-0.10	-	-	0.94	0.19	0.03	-	-
6HTQ	A,a	-	-	-	-	0.94	-	-	-	-	0.94	-	-	-	-	0.94
6I7O	BQ,2	-	-	-	-	1.00	-	-	-	-	0.99	-	-	-	-	1.00
6I7O	YQ,2b	-	-	-	-	1.00	-	-	-	-	0.99	-	-	-	-	1.00
6I7V	DA,AA	0.98	0.14	-0.46 (2671/2694)	-	-	0.96	0.13	-0.50 (941/949)	-	-	0.94	0.14	-0.44 (391/394)	-	-
6I7V	CA,BA	0.86	0.18	0.20	-	-	0.93	0.15	-0.27	-	-	0.86	0.17	0.01	-	-
6IP5	1A,2m	-	-	-	-	0.96	-	-	-	-	0.96	-	-	-	-	0.97
6IP6	1A,2m	-	-	-	-	0.76	-	-	-	-	0.80	-	-	-	-	0.78
6IP8	1A,2m	-	-	-	-	0.89	-	-	-	-	0.88	-	-	-	-	0.88
6LKQ	t,s	0.94	0.19	0.04	-	-	0.92	0.19	0.06	-	-	0.81	0.26	0.88	-	-
6MTB	5,9	-	-	-	-	0.93 (1416/1424)	-	-	-	-	0.93 (604/607)	-	-	-	-	0.93 (258/260)
6MTC	5,9	-	-	-	-	0.93 (1415/1422)	-	-	-	-	0.93 (610/613)	-	-	-	-	0.91 (264/266)
6MTD	5,9	-	-	-	-	0.94 (1401/1408)	-	-	-	-	0.92	-	-	-	-	0.92 (254/255)
6MTE	5,9	-	-	-	-	0.95 (1394/1401)	-	-	-	-	0.94 (604/607)	-	-	-	-	0.92 (223/224)
6N1D	A23S,A16S	0.92	0.18	-0.13	-	-	0.92	0.18	-0.08	-	-	0.85	0.20	0.15	-	-
6N1D	B23S,B16S	0.92	0.19	-0.01	-	-	0.92	0.17	-0.16	-	-	0.84	0.20	0.23	-	-
6N9E	1A,1a	0.95	0.22	0.34 (2061/2072)	-	-	0.93	0.21	0.19 (860/869)	-	-	0.89	0.18	-0.09 (417/420)	-	-
6N9E	2A,2a	0.95	0.21	0.18 (2122/2133)	-	-	0.94	0.19	-0.04 (880/889)	-	-	0.89	0.17	-0.31 (411/414)	-	-
6N9F	1A,1a	0.95	0.22	0.38 (2084/2095)	-	-	0.93	0.19	0.02 (871/880)	-	-	0.88	0.18	-0.18 (415/418)	-	-

Table 16 of 27

PDB		LSU					SSU BODY					SSU HEAD				
ID	chains	⟨rscc⟩	⟨rsr⟩	⟨rsrz⟩	⟨Q_score⟩	⟨inclusion⟩	⟨rscc⟩	⟨rsr⟩	⟨rsrz⟩	⟨Q_score⟩	⟨inclusion⟩	⟨rscc⟩	⟨rsr⟩	⟨rsrz⟩	⟨Q_score⟩	⟨inclusion⟩
6N9F	2A,2a	0.95	0.22	0.37 (2134/2145)	-	-	0.94	0.20	0.05 (876/885)	-	-	0.89	0.17	-0.32 (413/416)	-	-
6ND5	1A,1a	0.97	0.20	0.45 (2083/2094)	-	-	0.95	0.18	0.01 (862/871)	-	-	0.94	0.17	-0.09 (409/412)	-	-
6ND5	2A,2a	0.96	0.19	0.15 (2113/2124)	-	-	0.94	0.17	-0.13 (878/887)	-	-	0.87	0.21	0.52 (407/410)	-	-
6ND6	1A,1a	0.97	0.17	0.07 (2108/2119)	-	-	0.95	0.16	-0.07 (857/866)	-	-	0.93	0.16	-0.04 (412/415)	-	-
6ND6	2A,2a	0.95	0.17	0.02 (2123/2134)	-	-	0.94	0.16	-0.07 (888/897)	-	-	0.88	0.19	0.30 (406/409)	-	-
6NDK	RA,QA	-	-	-	-	-	-	-	-	-	-	-	-	-	-	-
6NDK	YA,XA	-	-	-	-	-	-	-	-	-	-	-	-	-	-	-
6NSH	YA,XA	-	-	-	-	-	-	-	-	-	-	-	-	-	-	-
6NSH	RA,QA	-	-	-	-	-	-	-	-	-	-	-	-	-	-	-
6NTA	YA,XA	-	-	-	-	-	-	-	-	-	-	-	-	-	-	-
6NTA	RA,QA	-	-	-	-	-	-	-	-	-	-	-	-	-	-	-
6NU2	A,AA	-	-	-	-	0.97	-	-	-	-	0.99	-	-	-	-	1.00
6NU3	A,AA	-	-	-	-	0.98	-	-	-	-	1.00	-	-	-	-	0.98
6NUO	YA,XA	-	-	-	-	-	-	-	-	-	-	-	-	-	-	-
6NUO	RA,QA	-	-	-	-	-	-	-	-	-	-	-	-	-	-	-
6NWY	YA,XA	-	-	-	-	-	-	-	-	-	-	-	-	-	-	-
6NWY	RA,QA	-	-	-	-	-	-	-	-	-	-	-	-	-	-	-
6O3M	YA,XA	-	-	-	-	-	-	-	-	-	-	-	-	-	-	-
6O3M	RA,QA	-	-	-	-	-	-	-	-	-	-	-	-	-	-	-
6O8W	A,a	-	-	-	-	0.99	-	-	-	-	1.00	-	-	-	-	1.00
6O8X	A,a	-	-	-	-	0.99	-	-	-	-	1.00	-	-	-	-	0.98
6O8Y	A,a	-	-	-	-	1.00	-	-	-	-	1.00	-	-	-	-	0.99
6O8Z	A,a	-	-	-	-	1.00	-	-	-	-	1.00	-	-	-	-	1.00
6O90	A,a	-	-	-	-	0.99	-	-	-	-	1.00	-	-	-	-	1.00
6O97	1A,1a	0.97	0.20	0.37 (2102/2113)	-	-	0.95	0.19	0.18 (844/853)	-	-	0.94	0.18	0.05 (412/415)	-	-
6O97	2A,2a	0.95	0.19	0.25 (2099/2110)	-	-	0.94	0.19	0.18 (872/881)	-	-	0.90	0.20	0.42 (409/412)	-	-
6O9J	B,a	-	-	-	-	0.93	-	-	-	-	0.94	-	-	-	-	0.76
6O9K	A,a	-	-	-	-	0.81	-	-	-	-	0.80	-	-	-	-	0.84
6OF1	1A,1a	0.97	0.20	0.42 (2060/2071)	-	-	0.95	0.19	0.30 (857/866)	-	-	0.94	0.18	0.11 (407/410)	-	-
6OF1	2A,2a	0.96	0.19	0.34 (2092/2103)	-	-	0.95	0.18	0.20 (882/891)	-	-	0.88	0.18	0.19 (411/414)	-	-
6OF6	YA,XA	-	-	-	-	-	-	-	-	-	-	-	-	-	-	-
6OF6	RA,QA	-	-	-	-	-	-	-	-	-	-	-	-	-	-	-
6OFX	1,3	-	-	-	-	0.98	-	-	-	-	1.00	-	-	-	-	0.98
6OG7	1,3	-	-	-	-	0.97	-	-	-	-	0.99	-	-	-	-	0.97
6OGF	1,3	-	-	-	-	0.98	-	-	-	-	0.99	-	-	-	-	0.97
6OGG	1,3	-	-	-	-	0.97	-	-	-	-	0.96	-	-	-	-	0.90
6OGI	1,3	-	-	-	-	0.98	-	-	-	-	0.98	-	-	-	-	0.91
6OJ2	YA,XA	-	-	-	-	-	-	-	-	-	-	-	-	-	-	-
6OJ2	RA,QA	-	-	-	-	-	-	-	-	-	-	-	-	-	-	-
6OLE	t,S2	-	-	-	-	0.96	-	-	-	-	0.93 (594/597)	-	-	-	-	0.93 (246/248)
6OLF	t,S2	-	-	-	-	0.86	-	-	-	-	0.78 (582/585)	-	-	-	-	0.75 (230/232)
6OLG	A2,B1	-	-	-	-	0.86	-	-	-	-	0.81	-	-	-	-	0.73
6OLI	t,S2	-	-	-	-	0.90	-	-	-	-	0.86 (591/594)	-	-	-	-	0.84 (243/245)
6OLZ	A2,B1	-	-	-	-	0.85	-	-	-	-	0.79	-	-	-	-	0.60

Table 17 of 27

PDB		LSU					SSU BODY					SSU HEAD				
ID	chains	⟨rscc⟩	⟨rsr⟩	⟨rsrz⟩	⟨Q_score⟩	⟨inclusion⟩	⟨rscc⟩	⟨rsr⟩	⟨rsrz⟩	⟨Q_score⟩	⟨inclusion⟩	⟨rscc⟩	⟨rsr⟩	⟨rsrz⟩	⟨Q_score⟩	⟨inclusion⟩
6OM0	t,S2	-	-	-	-	0.97	-	-	-	-	0.95 (579/582)	-	-	-	-	0.94 (239/241)
6OM6	1,2	-	-	-	-	0.97 (2590/2598)	-	-	-	-	0.97 (999/1003)	-	-	-	-	0.93 (405/408)
6OM7	t,S2	-	-	-	-	0.73	-	-	-	-	0.74 (571/574)	-	-	-	-	0.62 (233/234)
6OPE	YA,XA	-	-	-	-	-	-	-	-	-	-	-	-	-	-	-
6OPE	RA,QA	-	-	-	-	-	-	-	-	-	-	-	-	-	-	-
6ORD	RA,QA	-	-	-	-	-	-	-	-	-	-	-	-	-	-	-
6ORD	YA,XA	-	-	-	-	-	-	-	-	-	-	-	-	-	-	-
6ORE	1,2	-	-	-	-	0.98 (2628/2638)	-	-	-	-	0.96 (999/1002)	-	-	-	-	0.95 (431/434)
6ORL	1,2	-	-	-	-	1.00 (2531/2541)	-	-	-	-	1.00 (938/942)	-	-	-	-	1.00 (422/425)
6OSI	YA,XA	-	-	-	-	-	-	-	-	-	-	-	-	-	-	-
6OSI	RA,QA	-	-	-	-	-	-	-	-	-	-	-	-	-	-	-
6OSK	1,2	-	-	-	-	0.90 (2585/2595)	-	-	-	-	0.87 (980/983)	-	-	-	-	0.83 (436/439)
6OSQ	1,2	-	-	-	-	0.75 (2620/2630)	-	-	-	-	0.73 (1000/1003)	-	-	-	-	0.70 (429/432)
6OST	1,2	-	-	-	-	1.00 (2604/2614)	-	-	-	-	1.00 (988/991)	-	-	-	-	1.00 (427/430)
6OT3	1,2	-	-	-	-	1.00 (2573/2582)	-	-	-	-	1.00 (958/961)	-	-	-	-	1.00 (421/424)
6OTR	RA,QA	-	-	-	-	-	-	-	-	-	-	-	-	-	-	-
6OTR	YA,XA	-	-	-	-	-	-	-	-	-	-	-	-	-	-	-
6OUO	1,2	-	-	-	-	0.99 (2573/2582)	-	-	-	-	0.99 (958/961)	-	-	-	-	0.99 (421/424)
6OXA	RA,QA	-	-	-	-	-	-	-	-	-	-	-	-	-	-	-
6OXA	YA,XA	-	-	-	-	-	-	-	-	-	-	-	-	-	-	-
6OXI	RA,QA	-	-	-	-	-	-	-	-	-	-	-	-	-	-	-
6OXI	YA,XA	-	-	-	-	-	-	-	-	-	-	-	-	-	-	-
6P5I	5,2	-	-	-	-	0.94	-	-	-	-	0.89	-	-	-	-	0.74
6P5J	5,2	-	-	-	-	0.84	-	-	-	-	0.72	-	-	-	-	0.65
6P5K	5,2	-	-	-	-	0.93	-	-	-	-	0.86	-	-	-	-	0.81
6P5N	5,2	-	-	-	-	0.93	-	-	-	-	0.91	-	-	-	-	0.84
6Q8Y	BQ,2	-	-	-	-	0.98	-	-	-	-	0.98	-	-	-	-	0.97
6Q95	1,2	-	-	-	-	0.90	-	-	-	-	0.90	-	-	-	-	0.89
6Q97	1,2	-	-	-	-	0.20 (2087/2096)	-	-	-	-	0.20 (892/896)	-	-	-	-	0.14 (413/416)
6Q98	1,2	-	-	-	-	0.78 (2548/2558)	-	-	-	-	0.75 (977/981)	-	-	-	-	0.57 (338/341)
6Q9A	1,2	-	-	-	-	0.96 (2604/2614)	-	-	-	-	0.97 (991/995)	-	-	-	-	0.97 (420/423)
6QNQ	14,1G	0.96	0.12	-0.84 (2084/2095)	-	-	0.95	0.12	-0.86 (880/889)	-	-	0.90	0.11	-0.96 (391/394)	-	-
6QNQ	1H,13	0.96	0.13	-0.74 (2079/2090)	-	-	0.95	0.12	-0.93 (870/879)	-	-	0.91	0.12	-0.85 (403/406)	-	-
6QNR	1H,13	0.97	0.16	-0.34 (2080/2091)	-	-	0.96	0.14	-0.58 (857/866)	-	-	0.94	0.13	-0.66 (405/408)	-	-
6QNR	14,1G	0.96	0.16	-0.38 (2102/2113)	-	-	0.96	0.14	-0.59 (881/890)	-	-	0.91	0.13	-0.76 (407/410)	-	-
6QZP	L5,S2	-	-	-	-	0.99 (1383/1390)	-	-	-	-	0.99 (581/584)	-	-	-	-	0.99 (252/254)
6R5Q	5,K	-	-	-	-	0.90	-	-	-	-	0.79	-	-	-	-	0.65
6R6G	5,K	-	-	-	-	0.82	-	-	-	-	0.77	-	-	-	-	0.74

PDB		LSU					SSU BODY					SSU HEAD				
ID	chains	⟨rscc⟩	⟨rsr⟩	⟨rsrz⟩	⟨Q_score⟩	⟨inclusion⟩	⟨rscc⟩	⟨rsr⟩	⟨rsrz⟩	⟨Q_score⟩	⟨inclusion⟩	⟨rscc⟩	⟨rsr⟩	⟨rsrz⟩	⟨Q_score⟩	⟨inclusion⟩
6R6P	5,K	-	-	-	-	0.90	-	-	-	-	0.79	-	-	-	-	0.72
6R7Q	5,K	-	-	-	-	0.79	-	-	-	-	0.71	-	-	-	-	0.62
6RM3	L50,S60	-	-	-	-	0.92	-	-	-	-	0.87	-	-	-	-	0.68
6S0X	A,a	-	-	-	-	0.93	-	-	-	-	0.40	-	-	-	-	0.16
6S13	A,a	-	-	-	-	0.49	-	-	-	-	0.35	-	-	-	-	0.24
6S47	AA,BA	-	-	-	-	0.91	-	-	-	-	0.90	-	-	-	-	0.88
6SGC	54,A1	-	-	-	-	0.98	-	-	-	-	0.97	-	-	-	-	0.96
6SKF	BA,Aa	-	-	-	-	0.92 (880/911)	-	-	-	-	0.87 (426/442)	-	-	-	-	0.72 (213/215)
6SKG	BA,Aa	-	-	-	-	0.94 (895/902)	-	-	-	-	0.90 (424/433)	-	-	-	-	0.77 (219/225)
6SNT	1,2	-	-	-	-	0.99	-	-	-	-	0.96	-	-	-	-	0.92
6SPF	A,a	-	-	-	-	0.96	-	-	-	-	0.70	-	-	-	-	0.35
6SPG	A,a	-	-	-	-	0.93	-	-	-	-	0.89	-	-	-	-	0.75
6SV4	BQ,2	-	-	-	-	0.93	-	-	-	-	0.95	-	-	-	-	0.97
6SV4	YQ,2b	-	-	-	-	0.97	-	-	-	-	0.95	-	-	-	-	0.97
6SV4	ZQ,2c	-	-	-	-	0.97	-	-	-	-	0.94	-	-	-	-	0.96
6SZS	A,a	-	-	-	-	0.92	-	-	-	-	0.93	-	-	-	-	0.92
6T4Q	C1,C2	-	-	-	-	0.99	-	-	-	-	0.99	-	-	-	-	0.97
6T7I	C1,C2	-	-	-	-	0.99	-	-	-	-	0.98	-	-	-	-	0.98
6T7T	C1,C2	-	-	-	-	0.98	-	-	-	-	0.94	-	-	-	-	0.91
6T83	1b,2b	-	-	-	-	0.99	-	-	-	-	0.99	-	-	-	-	0.99
6T83	Aa,a	-	-	-	-	0.97	-	-	-	-	0.95	-	-	-	-	0.96
6TB3	BQ,2	-	-	-	-	0.99	-	-	-	-	0.98	-	-	-	-	0.99
6TBV	23S1,16S1	-	-	-	-	0.97 (2531/2541)	-	-	-	-	0.94 (960/963)	-	-	-	-	0.91 (428/431)
6TC3	23S1,16S1	-	-	-	-	0.96 (2511/2521)	-	-	-	-	0.93 (959/962)	-	-	-	-	0.90 (428/431)
6TH6	BA,Aa	-	-	-	-	0.93 (898/902)	-	-	-	-	0.89 (434/440)	-	-	-	-	0.72
6TNU	BQ,2	-	-	-	-	0.98	-	-	-	-	0.96	-	-	-	-	0.97
6UCQ	1A,1a	0.95	0.20	0.16 (2111/2120)	-	-	0.92	0.18	-0.12 (857/866)	-	-	0.79	0.21	0.19 (400/403)	-	-
6UCQ	2A,2a	0.92	0.19	0.02 (2084/2094)	-	-	0.89	0.20	0.09 (864/873)	-	-	0.72	0.21	0.26 (388/391)	-	-
6UO1	1A,1a	0.96	0.21	0.67 (2072/2083)	-	-	0.94	0.18	0.25 (854/863)	-	-	0.93	0.17	-0.02 (415/418)	-	-
6UO1	2A,2a	0.94	0.20	0.43 (2089/2100)	-	-	0.93	0.18	0.13 (876/885)	-	-	0.90	0.15	-0.28 (410/413)	-	-
6UZ7	5,2	-	-	-	-	0.82	-	-	-	-	0.68	-	-	-	-	0.34
6V39	AN1,sN1	-	-	-	-	0.96 (1456/1461)	-	-	-	-	0.94 (573/574)	-	-	-	-	0.94 (312/315)
6V3A	AN1,sN1	-	-	-	-	0.97 (1498/1503)	-	-	-	-	0.96 (572/573)	-	-	-	-	0.95 (329/332)
6V3B	AN1,sN1	-	-	-	-	0.98 (1482/1487)	-	-	-	-	0.96 (562/563)	-	-	-	-	0.94 (328/331)
6VLZ	A,AA	-	-	-	-	0.89	-	-	-	-	0.90	-	-	-	-	0.78
6VMI	A,AA	-	-	-	-	0.89	-	-	-	-	0.91	-	-	-	-	0.87
6VU3	a,D	-	-	-	-	0.45	-	-	-	-	0.48	-	-	-	-	0.40
6VWL	2,1	-	-	-	-	0.93	-	-	-	-	0.88	-	-	-	-	0.73
6VWM	2,1	-	-	-	-	0.94	-	-	-	-	0.92	-	-	-	-	0.75
6VWN	2,1	-	-	-	-	0.95	-	-	-	-	0.92	-	-	-	-	0.76
6VYQ	a,D	-	-	-	-	0.71	-	-	-	-	0.74	-	-	-	-	0.67
6VYR	a,D	-	-	-	-	0.65	-	-	-	-	0.67	-	-	-	-	0.63
6VYS	a,D	-	-	-	-	0.92	-	-	-	-	0.94	-	-	-	-	0.94
6VYT	a,D	-	-	-	-	0.68	-	-	-	-	0.74	-	-	-	-	0.74

Table 19 of 27

PDB		LSU					SSU BODY					SSU HEAD				
ID	chains	⟨rscc⟩	⟨rsr⟩	⟨rsrz⟩	⟨Q_score⟩	⟨inclusion⟩	⟨rscc⟩	⟨rsr⟩	⟨rsrz⟩	⟨Q_score⟩	⟨inclusion⟩	⟨rscc⟩	⟨rsr⟩	⟨rsrz⟩	⟨Q_score⟩	⟨inclusion⟩
6VYU	a,D	-	-	-	-	0.98	-	-	-	-	0.98	-	-	-	-	0.98
6VYW	a,D	-	-	-	-	0.96	-	-	-	-	0.96	-	-	-	-	0.95
6VYX	a,D	-	-	-	-	0.69	-	-	-	-	0.77	-	-	-	-	0.74
6VYY	a,D	-	-	-	-	0.69	-	-	-	-	0.76	-	-	-	-	0.74
6VYZ	a,D	-	-	-	-	0.71	-	-	-	-	0.77	-	-	-	-	0.75
6VZ2	a,D	-	-	-	-	0.98	-	-	-	-	0.99	-	-	-	-	0.99
6VZ3	a,D	-	-	-	-	0.99	-	-	-	-	0.99	-	-	-	-	1.00
6VZ5	a,D	-	-	-	-	0.70	-	-	-	-	0.75	-	-	-	-	0.73
6VZ7	a,D	-	-	-	-	0.00	-	-	-	-	0.00	-	-	-	-	0.00
6VZJ	a,D	-	-	-	-	0.50	-	-	-	-	0.50	-	-	-	-	0.43
6W6P	A,a	-	-	-	-	0.98	-	-	-	-	0.95	-	-	-	-	0.85
6WD0	1,3	-	-	-	-	0.99	-	-	-	-	0.99	-	-	-	-	0.99
6WD1	1,3	-	-	-	-	0.99	-	-	-	-	0.97	-	-	-	-	0.93
6WD2	1,3	-	-	-	-	0.99	-	-	-	-	0.99	-	-	-	-	0.98
6WD3	1,3	-	-	-	-	0.99	-	-	-	-	0.98	-	-	-	-	0.98
6WD4	1,3	-	-	-	-	0.99	-	-	-	-	0.98	-	-	-	-	0.98
6WD5	1,3	-	-	-	-	0.99	-	-	-	-	0.98	-	-	-	-	0.98
6WD6	1,3	-	-	-	-	0.99	-	-	-	-	0.98	-	-	-	-	0.97
6WD7	1,3	-	-	-	-	0.98	-	-	-	-	0.97	-	-	-	-	0.97
6WD8	1,3	-	-	-	-	0.99	-	-	-	-	0.98	-	-	-	-	0.97
6WD9	1,3	-	-	-	-	0.99	-	-	-	-	0.98	-	-	-	-	0.98
6WDA	1,3	-	-	-	-	0.99	-	-	-	-	0.98	-	-	-	-	0.97
6WDB	1,3	-	-	-	-	0.96	-	-	-	-	0.94	-	-	-	-	0.94
6WDC	1,3	-	-	-	-	0.97	-	-	-	-	0.96	-	-	-	-	0.95
6WDD	1,3	-	-	-	-	0.99	-	-	-	-	0.98	-	-	-	-	0.98
6WDE	1,3	-	-	-	-	0.99	-	-	-	-	0.99	-	-	-	-	0.99
6WDF	1,3	-	-	-	-	0.99	-	-	-	-	0.98	-	-	-	-	0.96
6WDG	1,3	-	-	-	-	0.99	-	-	-	-	0.98	-	-	-	-	0.96
6WDH	1,3	-	-	-	-	0.95	-	-	-	-	0.93	-	-	-	-	0.93
6WDI	1,3	-	-	-	-	0.96	-	-	-	-	0.95	-	-	-	-	0.93
6WDJ	1,3	-	-	-	-	0.98	-	-	-	-	0.98	-	-	-	-	0.98
6WDK	1,3	-	-	-	-	0.99	-	-	-	-	0.98	-	-	-	-	0.98
6WDL	1,3	-	-	-	-	0.98	-	-	-	-	0.98	-	-	-	-	0.97
6WDM	1,3	-	-	-	-	0.99	-	-	-	-	0.99	-	-	-	-	0.98
6WNV	4,3	-	-	-	-	0.99	-	-	-	-	0.98	-	-	-	-	0.85
6WNW	4,3	-	-	-	-	0.99	-	-	-	-	0.99	-	-	-	-	0.99
6WOO	5,2	-	-	-	-	0.88	-	-	-	-	0.84	-	-	-	-	0.82
6X6T	a,D	-	-	-	-	0.53	-	-	-	-	0.84	-	-	-	-	0.82
6X7F	a,D	-	-	-	-	0.69	-	-	-	-	0.97	-	-	-	-	0.96
6X7K	a,D	-	-	-	-	0.72	-	-	-	-	0.99	-	-	-	-	0.98
6X9Q	a,D	-	-	-	-	0.91	-	-	-	-	0.93	-	-	-	-	0.92
6XA1	L5,S2	-	-	-	-	0.93 (1357/1362)	-	-	-	-	0.83 (597/599)	-	-	-	-	0.68 (221/222)
6XDQ	a,D	-	-	-	-	0.99	-	-	-	-	0.99	-	-	-	-	0.99
6XDR	a,D	-	-	-	-	0.93	-	-	-	-	0.94	-	-	-	-	0.96
6XGF	a,D	-	-	-	-	0.97	-	-	-	-	0.97	-	-	-	-	0.97
6XHV	1A,1a	0.97	0.20	0.49 (2031/2043)	-	-	0.95	0.18	0.17 (854/863)	-	-	0.93	0.18	0.08 (410/413)	-	-
6XHV	2A,2a	0.95	0.19	0.28 (2047/2059)	-	-	0.94	0.18	0.13 (879/888)	-	-	0.89	0.19	0.39 (408/411)	-	-
6XHW	1A,1a	0.97	0.20	0.51 (2060/2071)	-	-	0.95	0.19	0.26 (844/853)	-	-	0.93	0.18	0.18 (411/414)	-	-
6XHW	2A,2a	0.95	0.19	0.33 (2089/2100)	-	-	0.93	0.19	0.27 (870/879)	-	-	0.88	0.21	0.64 (414/417)	-	-
6XHX	1A,1a	0.98	0.19	0.32 (2089/2100)	-	-	0.95	0.17	-0.01 (848/857)	-	-	0.94	0.16	-0.20 (412/415)	-	-

Table 20 of 27

PDB		LSU					SSU BODY					SSU HEAD				
ID	chains	⟨rscc⟩	⟨rsr⟩	⟨rsrz⟩	⟨Q_score⟩	⟨inclusion⟩	⟨rscc⟩	⟨rsr⟩	⟨rsrz⟩	⟨Q_score⟩	⟨inclusion⟩	⟨rscc⟩	⟨rsr⟩	⟨rsrz⟩	⟨Q_score⟩	⟨inclusion⟩
6XHX	2A,2a	0.96	0.18	0.08 (2134/2145)	-	-	0.95	0.17	-0.04 (879/888)	-	-	0.90	0.18	0.08 (415/418)	-	-
6XHY	1A,1a	0.98	0.20	0.35 (2076/2087)	-	-	0.96	0.19	0.15 (855/864)	-	-	0.95	0.18	-0.01 (414/417)	-	-
6XHY	2A,2a	0.96	0.19	0.22 (2095/2106)	-	-	0.95	0.18	0.06 (885/894)	-	-	0.91	0.19	0.28 (411/414)	-	-
6XII	a,D	-	-	-	-	0.92	-	-	-	-	0.92	-	-	-	-	0.91
6XIJ	a,D	-	-	-	-	0.84	-	-	-	-	0.83	-	-	-	-	0.80
6XIQ	1,2	-	-	-	-	0.97	-	-	-	-	0.99	-	-	-	-	0.99
6XIR	1,2	-	-	-	-	0.99	-	-	-	-	0.95	-	-	-	-	0.92
6XQD	1A,1a	0.97	0.20	0.45 (2043/2054)	-	-	0.95	0.20	0.54 (860/869)	-	-	0.93	0.20	0.52 (409/412)	-	-
6XQD	2A,2a	0.96	0.19	0.40 (2058/2069)	-	-	0.95	0.19	0.35 (872/881)	-	-	0.89	0.22	0.70 (406/409)	-	-
6XQE	1A,1a	0.96	0.19	0.10 (2074/2085)	-	-	0.94	0.19	0.09 (854/863)	-	-	0.92	0.20	0.15 (411/414)	-	-
6XQE	2A,2a	0.95	0.20	0.12 (2106/2117)	-	-	0.94	0.19	0.09 (879/888)	-	-	0.90	0.21	0.31 (412/415)	-	-
6XU6	A5,B2	-	-	-	-	1.00	-	-	-	-	1.00	-	-	-	-	0.99
6XU7	A5,B2	-	-	-	-	0.97	-	-	-	-	0.97	-	-	-	-	0.98
6XU8	A5,B2	-	-	-	-	0.99	-	-	-	-	0.96	-	-	-	-	0.93
6XYW	1,2	-	-	-	-	0.94	-	-	-	-	0.94	-	-	-	-	0.93
6XZA	A2,A1	-	-	-	-	0.92 (2624/2634)	-	-	-	-	0.83	-	-	-	-	0.86
6XZB	A2,A1	-	-	-	-	0.91 (2624/2634)	-	-	-	-	0.86	-	-	-	-	0.86
6Y0G	L5,S2	-	-	-	-	1.00	-	-	-	-	1.00	-	-	-	-	1.00
6Y2L	L5,S2	-	-	-	-	1.00	-	-	-	-	1.00	-	-	-	-	1.00
6Y57	L5,S2	-	-	-	-	1.00	-	-	-	-	1.00	-	-	-	-	1.00
6Y69	A,a	-	-	-	-	0.89	-	-	-	-	0.81	-	-	-	-	0.66
6YDP	BA,AA	-	-	-	-	0.96	-	-	-	-	0.95	-	-	-	-	0.89
6YDW	BA,AA	-	-	-	-	0.86	-	-	-	-	0.85	-	-	-	-	0.56
6YEF	A,a	-	-	-	-	0.98 (1547/1550)	-	-	-	-	0.91 (634/637)	-	-	-	-	0.85
6YSR	A,a	-	-	-	-	1.00 (2613/2622)	-	-	-	-	1.00 (995/998)	-	-	-	-	1.00 (435/438)
6YSS	A,a	-	-	-	-	0.96 (2617/2627)	-	-	-	-	0.95 (999/1002)	-	-	-	-	0.94 (431/434)
6YST	A,a	-	-	-	-	1.00 (2595/2605)	-	-	-	-	1.00 (914/917)	-	-	-	-	1.00 (347/350)
6YSU	A,a	-	-	-	-	1.00 (2587/2597)	-	-	-	-	1.00 (980/983)	-	-	-	-	1.00 (417/420)
6YWE	A,aa	-	-	-	-	0.97	-	-	-	-	0.98	-	-	-	-	0.99
6YWX	A,aa	-	-	-	-	0.98	-	-	-	-	0.99	-	-	-	-	0.99
6YWY	A,aa	-	-	-	-	0.69	-	-	-	-	0.70	-	-	-	-	0.65
6Z1P	Ab,Bb	-	-	-	-	0.83	-	-	-	-	0.83	-	-	-	-	0.75
6Z6J	C1,C2	-	-	-	-	0.89	-	-	-	-	0.86	-	-	-	-	0.64
6Z6K	C1,C2	-	-	-	-	0.94	-	-	-	-	0.92	-	-	-	-	0.85
6Z6L	L5,S2	-	-	-	-	1.00	-	-	-	-	1.00	-	-	-	-	1.00
6Z6M	L5,S2	-	-	-	-	1.00	-	-	-	-	1.00	-	-	-	-	1.00
6Z6N	L5,S2	-	-	-	-	1.00	-	-	-	-	1.00	-	-	-	-	1.00
6ZM5	A,AA	-	-	-	-	0.93	-	-	-	-	0.91	-	-	-	-	0.91
6ZM6	A,AA	-	-	-	-	0.96	-	-	-	-	0.93	-	-	-	-	0.93
6ZM7	L5,S2	-	-	-	-	1.00	-	-	-	-	1.00	-	-	-	-	0.99
6ZME	L5,S2	-	-	-	-	1.00	-	-	-	-	1.00	-	-	-	-	0.99
6ZMI	L5,S2	-	-	-	-	1.00	-	-	-	-	1.00	-	-	-	-	1.00

PDB		LSU					SSU BODY					SSU HEAD				
ID	chains	⟨rscc⟩	⟨rsr⟩	⟨rsrz⟩	⟨Q_score⟩	⟨inclusion⟩	⟨rscc⟩	⟨rsr⟩	⟨rsrz⟩	⟨Q_score⟩	⟨inclusion⟩	⟨rscc⟩	⟨rsr⟩	⟨rsrz⟩	⟨Q_score⟩	⟨inclusion⟩
6ZMO	L5,S2	-	-	-	-	1.00	-	-	-	-	1.00	-	-	-	-	0.99
6ZS9	XA,AA	-	-	-	-	0.90	-	-	-	-	0.90	-	-	-	-	0.81
6ZSA	XA,AA	-	-	-	-	0.91	-	-	-	-	0.92	-	-	-	-	0.92
6ZSB	XA,AA	-	-	-	-	0.88	-	-	-	-	0.91	-	-	-	-	0.91
6ZSC	XA,AA	-	-	-	-	0.93	-	-	-	-	0.92	-	-	-	-	0.91
6ZSD	XA,AA	-	-	-	-	0.92	-	-	-	-	0.92	-	-	-	-	0.92
6ZSE	XA,AA	-	-	-	-	0.89	-	-	-	-	0.89	-	-	-	-	0.83
6ZSG	XA,AA	-	-	-	-	0.94	-	-	-	-	0.95	-	-	-	-	0.95
6ZTJ	BA,AA	-	-	-	-	0.60 (2608/2617)	-	-	-	-	0.73 (976/979)	-	-	-	-	0.80 (439/442)
6ZTL	BA,AA	-	-	-	-	0.88 (2597/2606)	-	-	-	-	0.81 (959/962)	-	-	-	-	0.80 (433/436)
6ZTM	BA,AA	-	-	-	-	0.88 (2597/2606)	-	-	-	-	0.95 (957/960)	-	-	-	-	0.96 (440/443)
6ZTN	BA,AA	-	-	-	-	0.90 (2308/2317)	-	-	-	-	0.88 (842/845)	-	-	-	-	0.86 (429/432)
6ZTO	BA,AA	-	-	-	-	0.80 (2612/2621)	-	-	-	-	0.87 (989/992)	-	-	-	-	0.93 (435/438)
6ZTP	BA,AA	-	-	-	-	0.80 (2612/2621)	-	-	-	-	0.87 (989/992)	-	-	-	-	0.93 (435/438)
6ZU1	BA,AA	-	-	-	-	0.80 (2612/2621)	-	-	-	-	0.87 (989/992)	-	-	-	-	0.93 (435/438)
6ZU5	L50,S60	-	-	-	-	0.99	-	-	-	-	0.96	-	-	-	-	0.93
6ZVK	e2,K3	-	-	-	-	0.92	-	-	-	-	0.91	-	-	-	-	0.88
7A01	e2,K3	-	-	-	-	0.94	-	-	-	-	0.74	-	-	-	-	0.60
7A5F	A3,A6	-	-	-	-	0.80	-	-	-	-	0.77	-	-	-	-	0.74
7A5G	A3,A6	-	-	-	-	0.78	-	-	-	-	0.78	-	-	-	-	0.78
7A5I	A3,A6	-	-	-	-	0.88	-	-	-	-	0.83	-	-	-	-	0.80
7A5K	A3,A6	-	-	-	-	0.89	-	-	-	-	0.76	-	-	-	-	0.30
7ABZ	1,2	-	-	-	-	-	-	-	-	-	-	-	-	-	-	-
7AC7	1,2	-	-	-	-	-	-	-	-	-	-	-	-	-	-	-
7ACJ	1,2	-	-	-	-	-	-	-	-	-	-	-	-	-	-	-
7ACR	1,2	-	-	-	-	-	-	-	-	-	-	-	-	-	-	-
7ASO	Y,X	-	-	-	-	0.98	-	-	-	-	0.88	-	-	-	-	0.76
7ASP	Y,X	-	-	-	-	0.99	-	-	-	-	0.98	-	-	-	-	0.85
7AZO	23SA,16SA	0.95	0.18	-0.08 (2104/2115)	-	-	0.93	0.16	-0.36 (866/875)	-	-	0.92	0.16	-0.37 (407/410)	-	-
7AZO	23SB,16SB	0.94	0.17	-0.22 (2123/2134)	-	-	0.93	0.15	-0.50 (892/901)	-	-	0.88	0.14	-0.54 (411/414)	-	-
7AZS	23SA,16SA	0.94	0.19	0.10 (2110/2121)	-	-	0.87	0.19	0.10 (859/868)	-	-	0.85	0.19	0.02 (410/413)	-	-
7AZS	23SB,16SB	0.93	0.18	-0.04 (2117/2128)	-	-	0.86	0.19	0.03 (885/894)	-	-	0.76	0.19	0.07 (410/413)	-	-
7B5K	A,a	-	-	-	-	0.84 (2479/2488)	-	-	-	-	0.63 (900/903)	-	-	-	-	0.58 (430/433)
7B7D	LA,2	-	-	-	-	-	-	-	-	-	-	-	-	-	-	-
7CPJ	A,a	-	-	-	-	0.83 (2612/2622)	-	-	-	-	0.80 (982/985)	-	-	-	-	0.70 (440/443)
7CPU	L5,S2	-	-	-	-	0.99	-	-	-	-	0.91	-	-	-	-	0.89
7CPV	L5,S2	-	-	-	-	0.97	-	-	-	-	0.81	-	-	-	-	0.78
7D6Z	A,f	-	-	-	-	1.00	-	-	-	-	1.00	-	-	-	-	1.00
7D80	A,B	-	-	-	-	1.00	-	-	-	-	1.00	-	-	-	-	1.00
7JIL	1,2	-	-	-	-	0.96	-	-	-	-	0.92	-	-	-	-	0.82
7JQL	1A,1a	0.97	0.21	0.33 (2085/2096)	-	-	0.95	0.21	0.25 (854/863)	-	-	0.93	0.22	0.37 (414/417)	-	-
7JQL	2A,2a	0.96	0.21	0.24 (2102/2113)	-	-	0.95	0.21	0.24 (879/888)	-	-	0.91	0.24	0.65 (409/412)	-	-

Table 22 of 27

PDB		LSU					SSU BODY					SSU HEAD				
ID	chains	⟨rscc⟩	⟨rsr⟩	⟨rsrz⟩	⟨Q_score⟩	⟨inclusion⟩	⟨rscc⟩	⟨rsr⟩	⟨rsrz⟩	⟨Q_score⟩	⟨inclusion⟩	⟨rscc⟩	⟨rsr⟩	⟨rsrz⟩	⟨Q_score⟩	⟨inclusion⟩
7JQM	1A,1a	0.97	0.21	0.25 (2072/2083)	-	-	0.95	0.19	0.05 (849/858)	-	-	0.95	0.17	-0.15 (416/419)	-	-
7JQM	2A,2a	0.96	0.20	0.16 (2101/2112)	-	-	0.94	0.19	0.06 (873/882)	-	-	0.89	0.20	0.18 (406/409)	-	-
7JSS	1,3	-	-	-	-	0.95	-	-	-	-	0.94	-	-	-	-	0.86
7JSW	1,3	-	-	-	-	0.99	-	-	-	-	1.00	-	-	-	-	0.98
7JSZ	1,3	-	-	-	-	0.99	-	-	-	-	0.99	-	-	-	-	0.97
7JT1	1,3	-	-	-	-	1.00	-	-	-	-	0.99	-	-	-	-	0.99
7JT2	1,3	-	-	-	-	1.00	-	-	-	-	0.99	-	-	-	-	0.99
7JT3	1,3	-	-	-	-	0.98	-	-	-	-	0.95	-	-	-	-	0.88
7K00	a,A	-	-	-	-	0.93 (2616/2625)	-	-	-	-	0.85 (992/995)	-	-	-	-	0.91 (426/429)
7K50	1,3	-	-	-	-	0.98	-	-	-	-	0.97	-	-	-	-	0.96
7K51	1,3	-	-	-	-	0.98	-	-	-	-	0.94	-	-	-	-	0.84
7K52	1,3	-	-	-	-	0.98	-	-	-	-	0.93	-	-	-	-	0.80
7K53	1,3	-	-	-	-	0.99	-	-	-	-	0.97	-	-	-	-	0.95
7K54	1,3	-	-	-	-	0.99	-	-	-	-	0.95	-	-	-	-	0.79
7K55	1,3	-	-	-	-	0.99	-	-	-	-	0.97	-	-	-	-	0.93
7KGB	A,a	-	-	-	-	0.99 (2151/2154)	-	-	-	-	0.97 (856/858)	-	-	-	-	0.97 (409/411)
7L08	A,AA	-	-	-	-	0.94	-	-	-	-	0.95	-	-	-	-	0.87
7LH5	BA,AA	0.95	0.16	-0.27	-	-	0.95	0.15	-0.43	-	-	0.90	0.14	-0.58	-	-
7LH5	DA,CA	0.96	0.16	-0.27	-	-	0.95	0.15	-0.43	-	-	0.92	0.15	-0.44	-	-
7LS1	A2,m2	-	-	-	-	0.87 (1338/1343)	-	-	-	-	0.82 (520/521)	-	-	-	-	0.87
7LS2	A2,m2	-	-	-	-	0.92 (1346/1351)	-	-	-	-	0.91 (562/563)	-	-	-	-	0.92
7LV0	1,3	-	-	-	-	0.96	-	-	-	-	0.93	-	-	-	-	0.89
7M4W	A,a	-	-	-	-	0.96 (1570/1574)	-	-	-	-	0.95 (719/720)	-	-	-	-	0.98 (356/359)
7M4X	A,a	-	-	-	-	0.97 (1529/1532)	-	-	-	-	0.96 (721/722)	-	-	-	-	0.98 (380/383)
7M4Y	A,a	-	-	-	-	0.97 (1574/1579)	-	-	-	-	0.97 (721/723)	-	-	-	-	0.99 (351/354)
7M4Z	A,a	-	-	-	-	0.96 (1564/1569)	-	-	-	-	0.96 (739/741)	-	-	-	-	0.98 (355/358)
7M5D	1,2	-	-	-	0.59	0.85 (2659/2669)	-	-	-	0.57	0.76 (997/1001)	-	-	-	0.54	0.72 (428/431)
7MD7	1A,1a	0.97	0.21	0.56 (2084/2095)	-	-	0.95	0.19	0.38 (843/852)	-	-	0.93	0.19	0.29 (413/416)	-	-
7MD7	2A,2a	0.96	0.20	0.50 (2136/2147)	-	-	0.95	0.19	0.39 (871/880)	-	-	0.89	0.23	0.88 (418/421)	-	-
7MDZ	5,9	-	-	-	-	0.88	-	-	-	-	0.74	-	-	-	-	0.62
7MPI	A1,B5	-	-	-	-	0.99 (1390/1394)	-	-	-	-	0.99 (598/599)	-	-	-	-	1.00 (249/251)
7MPJ	A1,B5	-	-	-	-	0.97 (1429/1433)	-	-	-	-	0.96 (619/620)	-	-	-	-	0.98 (230/231)
7MSC	A,a	-	-	-	-	0.96 (2146/2147)	-	-	-	-	0.95 (855/856)	-	-	-	-	0.93 (410/412)
7MSH	A,a	-	-	-	-	0.93 (2078/2079)	-	-	-	-	0.91 (791/792)	-	-	-	-	0.87 (406/407)
7MSM	A,a	-	-	-	-	0.96 (2126/2127)	-	-	-	-	0.93 (864/865)	-	-	-	-	0.91 (405/407)
7MSZ	A,a	-	-	-	-	0.93 (2073/2074)	-	-	-	-	0.90 (794/795)	-	-	-	-	0.88 (403/405)
7MT2	A,a	-	-	-	-	0.96 (2146/2147)	-	-	-	-	0.95 (858/859)	-	-	-	-	0.93 (406/408)

Table 23 of 27

PDB		LSU					SSU BODY					SSU HEAD				
ID	chains	⟨rscc⟩	⟨rsr⟩	⟨rsrz⟩	⟨Q_score⟩	⟨inclusion⟩	⟨rscc⟩	⟨rsr⟩	⟨rsrz⟩	⟨Q_score⟩	⟨inclusion⟩	⟨rscc⟩	⟨rsr⟩	⟨rsrz⟩	⟨Q_score⟩	⟨inclusion⟩
7MT3	A,a	-	-	-	-	0.96 (2081/2082)	-	-	-	-	0.93 (815/816)	-	-	-	-	0.89 (408/410)
7MT7	A,a	-	-	-	-	0.97 (2170/2171)	-	-	-	-	0.95 (867/868)	-	-	-	-	0.93 (415/417)
7N1P	23,16	-	-	-	-	0.96 (2599/2608)	-	-	-	-	0.93 (976/979)	-	-	-	-	0.91 (437/440)
7N2C	23,16	-	-	-	-	0.98 (2539/2548)	-	-	-	-	0.90 (921/924)	-	-	-	-	0.87 (385/387)
7N2U	23,16	-	-	-	-	0.97 (2528/2537)	-	-	-	-	0.87 (961/964)	-	-	-	-	0.86 (435/438)
7N2V	23,16	-	-	-	-	0.99 (2507/2517)	-	-	-	-	0.98 (930/934)	-	-	-	-	0.95 (433/435)
7N30	23,16	-	-	-	-	-	-	-	-	-	-	-	-	-	-	-
7N31	23,16	-	-	-	-	0.93 (2589/2600)	-	-	-	-	0.89 (991/995)	-	-	-	-	0.84 (441/444)
7N8B	A1,B5	-	-	-	-	0.99 (1419/1423)	-	-	-	-	0.99 (608/609)	-	-	-	-	0.99 (244/246)
7NBU	a,A	-	-	-	-	0.94 (2632/2642)	-	-	-	-	0.91 (992/995)	-	-	-	-	0.88 (430/433)
7NHK	A,a	-	-	-	-	0.85	-	-	-	-	0.74	-	-	-	-	0.75
7NHL	A,a	-	-	-	-	0.88	-	-	-	-	0.83	-	-	-	-	0.86
7NHM	A,a	-	-	-	-	0.94	-	-	-	-	0.90	-	-	-	-	0.91
7NHN	A,a	-	-	-	-	0.88	-	-	-	-	0.65	-	-	-	-	0.61
7NQH	BA,AA	-	-	-	-	0.87	-	-	-	-	0.87	-	-	-	-	0.85
7NQL	BA,AA	-	-	-	-	0.89	-	-	-	-	0.85	-	-	-	-	0.80
7NRC	LA,S2	-	-	-	-	0.96	-	-	-	-	0.95	-	-	-	-	0.94
7NRD	LA,S2	-	-	-	-	0.93	-	-	-	-	0.95	-	-	-	-	0.95
7NSI	BA,AA	-	-	-	-	0.86	-	-	-	-	0.79	-	-	-	-	0.61
7NSJ	BA,AA	-	-	-	-	0.91	-	-	-	-	0.92	-	-	-	-	0.89
7NSO	A,a	-	-	-	-	0.90	-	-	-	-	0.88	-	-	-	-	0.83
7NSP	A,a	-	-	-	-	0.72	-	-	-	-	0.67	-	-	-	-	0.66
7NSQ	A,a	-	-	-	-	0.86	-	-	-	-	0.83	-	-	-	-	0.83
7NWG	51,A2	-	-	-	-	0.88	-	-	-	-	0.87	-	-	-	-	0.86
7NWH	5,9	-	-	-	-	0.90	-	-	-	-	0.81	-	-	-	-	0.62
7NWI	5,9	-	-	-	-	0.88	-	-	-	-	0.92	-	-	-	-	0.90
7NWT	1,2	-	-	-	-	1.00 (2582/2592)	-	-	-	-	0.99 (986/990)	-	-	-	-	1.00 (417/420)
7NWW	1,2	-	-	-	-	1.00 (2597/2607)	-	-	-	-	1.00 (998/1001)	-	-	-	-	1.00 (427/430)
7O19	BA,AA	-	-	-	-	-	-	-	-	-	-	-	-	-	-	-
7O1A	BA,AA	-	-	-	-	1.00 (2508/2518)	-	-	-	-	0.99 (962/965)	-	-	-	-	0.98 (427/430)
7O1C	BA,AA	-	-	-	-	-	-	-	-	-	-	-	-	-	-	-
7O5B	X,A	-	-	-	-	0.93	-	-	-	-	0.92	-	-	-	-	0.90
7O7Y	B5,A2	-	-	-	-	0.98 (1417/1421)	-	-	-	-	0.94 (601/603)	-	-	-	-	0.86 (225/226)
7O7Z	B5,A2	-	-	-	-	0.99 (1412/1416)	-	-	-	-	0.99 (597/599)	-	-	-	-	0.99 (224/225)
7O80	B5,A2	-	-	-	-	0.90 (1432/1436)	-	-	-	-	0.85 (631/633)	-	-	-	-	0.77 (208/209)
7O81	B5,A2	-	-	-	-	0.95 (1370/1374)	-	-	-	-	0.94 (617/619)	-	-	-	-	0.94 (200/201)
7OG4	XA,AA	-	-	-	-	0.91	-	-	-	-	0.89	-	-	-	-	0.87
7OIF	1,2	-	-	-	-	1.00 (2606/2616)	-	-	-	-	1.00 (998/1001)	-	-	-	-	1.00 (425/428)
7OIG	1,2	-	-	-	-	1.00 (2597/2607)	-	-	-	-	1.00 (997/1000)	-	-	-	-	1.00 (425/428)

Table 24 of 27

PDB		LSU					SSU BODY					SSU HEAD				
ID	chains	⟨rscc⟩	⟨rsr⟩	⟨rsrz⟩	⟨Q_score⟩	⟨inclusion⟩	⟨rscc⟩	⟨rsr⟩	⟨rsrz⟩	⟨Q_score⟩	⟨inclusion⟩	⟨rscc⟩	⟨rsr⟩	⟨rsrz⟩	⟨Q_score⟩	⟨inclusion⟩
7OII	1,2	-	-	-	-	1.00<br>(2603/2613)	-	-	-	-	1.00<br>(998/1001)	-	-	-	-	1.00<br>(430/433)
7OIZ	a,A	-	-	-	-	-	-	-	-	-	-	-	-	-	-	-
7OJ0	a,A	-	-	-	-	-	-	-	-	-	-	-	-	-	-	-
7OLC	1,2	-	-	-	-	1.00	-	-	-	-	1.00	-	-	-	-	0.98
7OLD	1,2	-	-	-	-	0.99	-	-	-	-	0.98	-	-	-	-	0.96
7OSA	25S,18S	0.96	0.16	-0.27	-	-	0.96	0.15	-0.47	-	-	0.94	0.14	-0.53	-	-
7OSM	25S,18S	0.95	0.14	-0.55	-	-	0.96	0.13	-0.71	-	-	0.89	0.13	-0.73	-	-
7OT5	1,2	-	-	-	-	-	-	-	-	-	-	-	-	-	-	-
7OTC	A,a	-	-	-	-	0.97<br>(2594/2604)	-	-	-	-	0.97<br>(964/967)	-	-	-	-	0.92<br>(371/373)
7OYA	51,22	-	-	-	-	0.98	-	-	-	-	1.00	-	-	-	-	0.98
7OYB	51,22	-	-	-	-	1.00	-	-	-	-	0.98	-	-	-	-	0.96
7OYC	51,22	-	-	-	-	0.99	-	-	-	-	0.98	-	-	-	-	0.97
7OYD	5,9	-	-	-	-	0.99<br>(1314/1322)	-	-	-	-	0.79<br>(604/607)	-	-	-	-	0.49<br>(223/224)
7P3K	a,A	-	-	-	-	1.00<br>(2597/2607)	-	-	-	-	0.98<br>(945/948)	-	-	-	-	0.99<br>(439/442)
7P48	A,a	-	-	-	-	0.99	-	-	-	-	0.82	-	-	-	-	0.88
7P6Z	3,5	-	-	-	0.45	0.89	-	-	-	0.35	0.75	-	-	-	0.33	0.64
7P7Q	A,a	-	-	-	-	0.85<br>(2303/2305)	-	-	-	-	0.39	-	-	-	-	0.28
7P7R	A,a	-	-	-	-	0.22	-	-	-	-	0.18	-	-	-	-	0.09
7P7S	A,a	-	-	-	-	0.99	-	-	-	-	0.97	-	-	-	-	0.94
7P7T	A,a	-	-	-	-	0.99	-	-	-	-	0.98	-	-	-	-	0.97
7P7U	A,a	-	-	-	-	0.97	-	-	-	-	0.95	-	-	-	-	0.93
7PAH	3,5	-	-	-	-	0.97	-	-	-	-	0.95	-	-	-	-	0.95
7PAI	3,5	-	-	-	0.23	0.96	-	-	-	0.22	0.95	-	-	-	0.21	0.95
7PAJ	3,5	-	-	-	0.22	0.97	-	-	-	0.21	0.96	-	-	-	0.20	0.96
7PAK	3,5	-	-	-	0.30	0.95	-	-	-	0.29	0.94	-	-	-	0.28	0.94
7PAL	3,5	-	-	-	0.35	0.90	-	-	-	0.33	0.87	-	-	-	0.31	0.84
7PAM	3,5	-	-	-	0.23	0.97	-	-	-	0.22	0.96	-	-	-	0.19	0.95
7PAN	3,5	-	-	-	0.16	0.96	-	-	-	0.16	0.95	-	-	-	0.15	0.95
7PAO	3,5	-	-	-	0.22	0.97	-	-	-	0.21	0.96	-	-	-	0.20	0.96
7PAQ	3,5	-	-	-	0.17	0.98	-	-	-	0.17	0.97	-	-	-	0.16	0.96
7PAR	3,5	-	-	-	0.19	0.96	-	-	-	0.18	0.93	-	-	-	0.15	0.87
7PAS	3,5	-	-	-	0.08	1.00	-	-	-	0.08	0.98	-	-	-	0.07	0.97
7PH9	3,5	-	-	-	-	0.98	-	-	-	-	0.97	-	-	-	-	0.98
7PHA	3,5	-	-	-	-	0.99	-	-	-	-	0.99	-	-	-	-	0.99
7PHB	3,5	-	-	-	0.32	0.86	-	-	-	0.30	0.82	-	-	-	0.28	0.78
7PHC	3,5	-	-	-	-	0.98	-	-	-	-	0.96	-	-	-	-	0.96
7PI8	3,5	-	-	-	-	0.93	-	-	-	-	0.91	-	-	-	-	0.91
7PI9	3,5	-	-	-	-	0.95	-	-	-	-	0.93	-	-	-	-	0.94
7PIA	3,5	-	-	-	-	1.00	-	-	-	-	0.96	-	-	-	-	0.97
7PIB	3,5	-	-	-	-	0.90	-	-	-	-	0.88	-	-	-	-	0.82
7PIC	3,5	-	-	-	0.16	0.98	-	-	-	0.16	0.97	-	-	-	0.15	0.96
7PIO	3,5	-	-	-	-	0.95	-	-	-	-	0.93	-	-	-	-	0.92
7PIP	3,5	-	-	-	-	0.96	-	-	-	-	0.95	-	-	-	-	0.95
7PIQ	3,5	-	-	-	-	0.97	-	-	-	-	0.94	-	-	-	-	0.81
7PIR	3,5	-	-	-	-	1.00	-	-	-	-	0.99	-	-	-	-	0.99
7PIS	3,5	-	-	-	-	1.00	-	-	-	-	1.00	-	-	-	-	1.00
7PIT	3,5	-	-	-	-	0.90	-	-	-	-	0.88	-	-	-	-	0.85
7PJS	A,a	-	-	-	-	0.99<br>(2621/2631)	-	-	-	-	1.00<br>(976/979)	-	-	-	-	0.99<br>(431/434)
7PJT	A,a	-	-	-	-	0.97<br>(2518/2526)	-	-	-	-	0.97<br>(882/885)	-	-	-	-	0.97<br>(395/398)

Table 25 of 27

PDB		LSU					SSU BODY					SSU HEAD				
ID	chains	⟨rscc⟩	⟨rsr⟩	⟨rsrz⟩	⟨Q_score⟩	⟨inclusion⟩	⟨rscc⟩	⟨rsr⟩	⟨rsrz⟩	⟨Q_score⟩	⟨inclusion⟩	⟨rscc⟩	⟨rsr⟩	⟨rsrz⟩	⟨Q_score⟩	⟨inclusion⟩
7PJU	A,a	-	-	-	-	0.91 (2541/2550)	-	-	-	-	0.90 (945/948)	-	-	-	-	0.92 (416/419)
7PJV	A,a	-	-	-	-	0.99 (2543/2552)	-	-	-	-	0.98 (940/943)	-	-	-	-	0.98 (420/423)
7PJW	A,a	-	-	-	-	0.52 (2540/2549)	-	-	-	-	0.44 (938/941)	-	-	-	-	0.38 (414/417)
7PJX	A,a	-	-	-	-	0.95 (2544/2553)	-	-	-	-	0.92 (932/935)	-	-	-	-	0.93 (421/424)
7PJY	A,a	-	-	-	-	0.98 (2588/2598)	-	-	-	-	0.97 (874/877)	-	-	-	-	0.96 (374/377)
7PJZ	A,a	-	-	-	-	0.96 (2543/2552)	-	-	-	-	0.97 (815/817)	-	-	-	-	0.97 (360/362)
7PWO	1,2	-	-	-	-	1.00 (910/912)	-	-	-	-	1.00 (454/456)	-	-	-	-	0.99 (241/242)
7PZY	1,A	-	-	-	-	0.99	-	-	-	-	0.79	-	-	-	-	0.20
7Q08	1,A	-	-	-	-	1.00	-	-	-	-	0.99	-	-	-	-	0.95
7Q0F	1,A	-	-	-	-	1.00	-	-	-	-	0.99	-	-	-	-	0.94
7Q0P	1,A	-	-	-	-	0.92	-	-	-	-	0.64	-	-	-	-	0.24
7Q0R	1,A	-	-	-	-	0.96	-	-	-	-	0.69	-	-	-	-	0.24
7QEP	1,3	-	-	-	-	0.99	-	-	-	-	0.96	-	-	-	-	0.86
7QG8	N,0	-	-	-	-	0.97	-	-	-	-	0.97	-	-	-	-	0.93
7QGG	t,S2	-	-	-	0.60	0.98	-	-	-	0.55	0.93 (523/526)	-	-	-	0.50	0.91 (222/223)
7QGH	N,0	-	-	-	-	0.96	-	-	-	-	0.97	-	-	-	-	0.94
7QGN	N,0	-	-	-	-	0.96	-	-	-	-	0.92	-	-	-	-	0.95
7QGR	N,0	-	-	-	-	0.77	-	-	-	-	0.85	-	-	-	-	0.84
7QGU	A,W	-	-	-	-	0.95	-	-	-	-	0.95	-	-	-	-	0.98
7QH4	A,W	-	-	-	-	0.94	-	-	-	-	0.94	-	-	-	-	0.97
7QI4	A,AA	-	-	-	-	0.93 (1008/1009)	-	-	-	-	0.94 (341/342)	-	-	-	-	0.90
7QIZ	2,S2	-	-	-	-	0.91 (1342/1345)	-	-	-	-	0.86 (596/598)	-	-	-	-	0.83 (247/249)
7QV1	V,a	-	-	-	-	0.91	-	-	-	-	0.95	-	-	-	-	0.83
7QV2	V,a	-	-	-	-	0.92	-	-	-	-	0.94	-	-	-	-	0.93
7QV3	V,a	-	-	-	-	0.98	-	-	-	-	0.99	-	-	-	-	0.98
7QVP	L5,S2	-	-	-	0.56	1.00	-	-	-	0.53	1.00	-	-	-	0.49	1.00
7QVP	L6,S3	-	-	-	0.52	1.00	-	-	-	0.46	1.00	-	-	-	0.43	1.00
7R81	A1,A2	-	-	-	-	0.94	-	-	-	-	0.82	-	-	-	-	0.75
7RQ8	1A,1a	0.98	0.19	0.41 (2091/2102)	-	-	0.95	0.18	0.11 (846/855)	-	-	0.94	0.17	-0.01 (411/414)	-	-
7RQ8	2A,2a	0.96	0.18	0.13 (2104/2115)	-	-	0.93	0.18	0.09 (882/891)	-	-	0.84	0.21	0.79 (411/414)	-	-
7RQ9	1A,1a	0.97	0.20	0.37 (2075/2087)	-	-	0.95	0.18	0.09 (849/858)	-	-	0.94	0.19	0.25 (411/414)	-	-
7RQ9	2A,2a	0.96	0.19	0.22 (2103/2115)	-	-	0.94	0.17	-0.08 (886/895)	-	-	0.90	0.20	0.42 (407/410)	-	-
7RQA	1A,1a	0.96	0.21	0.76 (2115/2126)	-	-	0.93	0.20	0.51 (851/860)	-	-	0.90	0.20	0.51 (412/415)	-	-
7RQA	2A,2a	0.94	0.20	0.49 (2150/2161)	-	-	0.93	0.19	0.40 (877/886)	-	-	0.87	0.21	0.82 (410/413)	-	-
7RQB	1A,1a	0.97	0.20	0.53 (2116/2127)	-	-	0.95	0.18	0.15 (849/858)	-	-	0.93	0.18	0.07 (414/417)	-	-
7RQB	2A,2a	0.96	0.19	0.26 (2144/2155)	-	-	0.94	0.18	0.15 (874/883)	-	-	0.87	0.22	0.87 (415/418)	-	-
7RQC	1A,1a	0.97	0.19	0.31 (2108/2119)	-	-	0.95	0.17	0.00 (844/853)	-	-	0.93	0.18	0.06 (418/421)	-	-

PDB		LSU					SSU BODY					SSU HEAD				
ID	chains	⟨rscc⟩	⟨rsr⟩	⟨rsrz⟩	⟨Q_score⟩	⟨inclusion⟩	⟨rscc⟩	⟨rsr⟩	⟨rsrz⟩	⟨Q_score⟩	⟨inclusion⟩	⟨rscc⟩	⟨rsr⟩	⟨rsrz⟩	⟨Q_score⟩	⟨inclusion⟩
7RQC	2A,2a	0.96	0.18	0.10 (2122/2133)	-	-	0.94	0.17	-0.01 (872/881)	-	-	0.89	0.19	0.37 (418/421)	-	-
7RQD	1A,1a	0.97	0.21	0.68 (2110/2121)	-	-	0.94	0.19	0.37 (851/860)	-	-	0.92	0.20	0.45 (409/412)	-	-
7RQD	2A,2a	0.95	0.20	0.48 (2130/2141)	-	-	0.93	0.19	0.38 (866/875)	-	-	0.88	0.21	0.81 (413/416)	-	-
7RQE	1A,1a	0.97	0.19	0.29 (2114/2125)	-	-	0.95	0.17	-0.04 (844/853)	-	-	0.93	0.17	-0.11 (418/421)	-	-
7RQE	2A,2a	0.96	0.17	0.03 (2133/2144)	-	-	0.94	0.17	-0.07 (870/879)	-	-	0.88	0.20	0.53 (415/418)	-	-
7RR5	C1,C2	-	-	-	-	0.94	-	-	-	-	0.94	-	-	-	-	0.94
7RYF	A,a	-	-	-	-	0.97 (1289/1293)	-	-	-	-	0.96 (536/537)	-	-	-	-	0.94 (332/335)
7RYG	A,a	-	-	-	-	0.98 (1312/1317)	-	-	-	-	0.96 (552/553)	-	-	-	-	0.96 (333/336)
7RYH	A,a	-	-	-	-	0.98 (1290/1295)	-	-	-	-	0.96 (539/540)	-	-	-	-	0.96 (337/340)
7S1G	I,C	-	-	-	-	0.85 (2603/2613)	-	-	-	-	0.84	-	-	-	-	0.80
7S1H	I,C	-	-	-	-	0.86 (2599/2609)	-	-	-	-	0.83	-	-	-	-	0.73
7S1I	I,C	-	-	-	-	0.88 (2600/2610)	-	-	-	-	0.88	-	-	-	-	0.85
7S1J	I,C	-	-	-	-	0.92 (2599/2609)	-	-	-	-	0.92	-	-	-	-	0.87
7S1K	I,C	-	-	-	-	0.86 (2592/2602)	-	-	-	-	0.86	-	-	-	-	0.83
7SA4	1,2	-	-	-	-	0.98 (2620/2630)	-	-	-	-	0.97 (994/997)	-	-	-	-	0.95 (431/434)
7SFR	A,a	-	-	-	0.61	0.92 (2089/2091)	-	-	-	0.41	0.03 (825/827)	-	-	-	0.22	0.00 (385/387)
7SS9	1,3	-	-	-	-	0.93	-	-	-	-	0.89	-	-	-	-	0.54
7SSD	1,3	-	-	-	-	0.98	-	-	-	-	0.96	-	-	-	-	0.93
7SSL	1,3	-	-	-	-	0.95	-	-	-	-	0.93	-	-	-	-	0.90
7SSN	1,3	-	-	-	-	0.98	-	-	-	-	0.96	-	-	-	-	0.94
7SSO	1,3	-	-	-	-	0.99	-	-	-	-	0.98	-	-	-	-	0.95
7SSW	1,3	-	-	-	-	0.98	-	-	-	-	0.97	-	-	-	-	0.94
7ST2	1,3	-	-	-	-	1.00	-	-	-	-	1.00	-	-	-	-	1.00
7ST6	1,3	-	-	-	-	1.00	-	-	-	-	1.00	-	-	-	-	0.99
7ST7	1,3	-	-	-	-	0.97	-	-	-	-	0.95	-	-	-	-	0.94
7TOQ	A25S,A18S	-	-	-	-	0.98	-	-	-	-	0.87	-	-	-	-	0.82
7TOR	A28S,A18S	-	-	-	-	1.00	-	-	-	-	1.00	-	-	-	-	1.00
7TOS	23S,16S	-	-	-	-	1.00	-	-	-	-	1.00	-	-	-	-	0.99
7U2H	1A,1a	0.98	0.20	0.48 (2062/2073)	-	-	0.95	0.18	0.11 (839/848)	-	-	0.93	0.17	-0.08 (415/418)	-	-
7U2H	2A,2a	0.96	0.18	0.19 (2091/2102)	-	-	0.94	0.17	0.03 (880/889)	-	-	0.85	0.21	0.71 (410/413)	-	-
7U2I	1A,1a	0.97	0.22	0.93 (2075/2086)	-	-	0.94	0.20	0.51 (838/847)	-	-	0.92	0.19	0.35 (417/420)	-	-
7U2I	2A,2a	0.95	0.20	0.61 (2104/2115)	-	-	0.93	0.19	0.43 (875/884)	-	-	0.85	0.22	0.86 (411/414)	-	-
7U2J	1A,1a	0.98	0.19	0.27 (2105/2116)	-	-	0.95	0.17	-0.06 (869/878)	-	-	0.92	0.18	0.06 (414/417)	-	-
7U2J	2A,2a	0.96	0.18	0.11 (2120/2131)	-	-	0.94	0.16	-0.15 (879/888)	-	-	0.86	0.20	0.57 (413/416)	-	-
7UCJ	5,9	-	-	-	-	0.89 (1207/1214)	-	-	-	-	0.86 (554/557)	-	-	-	-	0.80 (249/251)

Table 27 of 27

PDB		LSU					SSU BODY					SSU HEAD				
ID	chains	⟨rscc⟩	⟨rsr⟩	⟨rsrz⟩	⟨Q_score⟩	⟨inclusion⟩	⟨rscc⟩	⟨rsr⟩	⟨rsrz⟩	⟨Q_score⟩	⟨inclusion⟩	⟨rscc⟩	⟨rsr⟩	⟨rsrz⟩	⟨Q_score⟩	⟨inclusion⟩
7UCK	5,9	-	-	-	-	0.98 (1202/1210)	-	-	-	-	0.98 (556/559)	-	-	-	-	0.96 (251/252)
7UG7	23,16	-	-	-	-	0.99 (2593/2603)	-	-	-	-	0.97 (893/896)	-	-	-	-	0.92 (363/365)
7UNR	A,a	-	-	-	-	0.97 (2036/2045)	-	-	-	-	0.93 (801/804)	-	-	-	-	0.91 (413/416)
7UNU	A,a	-	-	-	-	0.97 (2046/2055)	-	-	-	-	0.94 (818/821)	-	-	-	-	0.91 (408/411)
7UNV	A,a	-	-	-	-	1.00 (2041/2050)	-	-	-	-	1.00 (771/774)	-	-	-	-	0.99 (407/410)
7UNW	A,a	-	-	-	-	1.00 (2056/2065)	-	-	-	-	1.00 (774/777)	-	-	-	-	0.99 (405/407)
7ZJW	L5,S2	-	-	-	0.61	1.00 (1370/1374)	-	-	-	0.57	0.99 (627/629)	-	-	-	0.55	0.99 (197/198)
7ZJX	L5,S2	-	-	-	-	0.99 (1436/1440)	-	-	-	-	0.98 (602/604)	-	-	-	-	0.98 (186/187)
7ZW0	LA,2	-	-	-	0.70	1.00	-	-	-	0.68	1.00	-	-	-	0.67	1.00
8CVJ	1A,1a	0.96	0.19	0.43 (2019/2029)	-	-	0.93	0.19	0.37 (821/830)	-	-	0.91	0.20	0.52 (409/412)	-	-
8CVJ	2A,2a	0.94	0.19	0.40 (2071/2082)	-	-	0.92	0.19	0.39 (883/892)	-	-	0.87	0.23	1.08 (410/413)	-	-
8CVK	1A,1a	0.97	0.19	0.40 (2074/2085)	-	-	0.94	0.19	0.35 (847/856)	-	-	0.93	0.20	0.55 (409/412)	-	-
8CVK	2A,2a	0.95	0.19	0.39 (2083/2094)	-	-	0.93	0.19	0.32 (878/887)	-	-	0.89	0.23	1.08 (411/414)	-	-
8CVL	1A,1a	0.96	0.20	1.17 (2076/2087)	-	-	0.92	0.20	1.13 (853/862)	-	-	0.91	0.20	1.22 (409/412)	-	-
8CVL	2A,2a	0.94	0.20	1.05 (2103/2114)	-	-	0.92	0.20	1.06 (880/889)	-	-	0.88	0.22	1.58 (410/413)	-	-
