# Supplementary material for: Ratchet, swivel, tilt and roll: a complete description of subunit rotation in the ribosome
Source: Nucleic Acids Res. 2022 Dec 30;51(2):919–34. doi: 10.1093/nar/gkac1211 (PMC9881166; doi:10.1093/nar/gkac1211)
Supplement: gkac1211_Supplemental_Files [file gkac1211_supplemental_files.zip › AppendixF.pdf]

# Appendix F: RCSB validation data for ribosome structures described with the RAD method - Isolated SSUs

Below are tables that provide summaries of validation statistics for 334 structures.

For each rRNA chain, the average values were calculated for all residues that were identied as being part of a “core”. Averages were calculated from the values provided by the RCSB validation reports, which contain per-residue values.

If a validation report did not contain any values for the core residues, then the entry is listed with a “-”.

If validation quantities were only available for a fraction of the core residues, then the number of found values and the number of core residues is shown in parentheses.

**Quantities calculated**

- ⟨rscc⟩: average rscc value of core residues (X-RAY)
- ⟨rsr⟩: average rsr value of core residues (X-RAY)
- ⟨rsrz⟩: average rsrz value of core residues (X-RAY)
- ⟨Q_score⟩: average Q-score value of core residues (EM)
- ⟨inclusion⟩: average inclusion value of core residues (EM)

**Table 1 of 8**

| PDB | | SSU BODY | | | | | SSU HEAD | | | | |
|---|---|---|---|---|---|---|---|---|---|---|---|
| ID | chain | ⟨rscc⟩ | ⟨rsr⟩ | ⟨rsrz⟩ | ⟨Q_score⟩ | ⟨inclusion⟩ | ⟨rscc⟩ | ⟨rsr⟩ | ⟨rsrz⟩ | ⟨Q_score⟩ | ⟨inclusion⟩ |
| 1FJG | A | 0.96 | 0.24 | 0.64 | - | - | 0.94 | 0.24 | 0.69 | - | - |
| 1FKA | A | - | - | - | - | - | - | - | - | - | - |
| 1HNW | A | 0.95 | 0.19 | 0.11 | - | - | 0.93 | 0.21 | 0.34 | - | - |
| 1HNX | A | 0.93 | 0.27 | 1.00 | - | - | 0.90 | 0.29 | 1.29 | - | - |
| 1HNZ | A | 0.94 | 0.21 | 0.34 | - | - | 0.92 | 0.23 | 0.51 | - | - |
| 1HR0 | A | 0.95 | 0.21 | 0.36 | - | - | 0.92 | 0.23 | 0.54 | - | - |
| 1I94 | A | - | - | - | - | - | - | - | - | - | - |
| 1I95 | A | - | - | - | - | - | - | - | - | - | - |
| 1I96 | A | - | - | - | - | - | - | - | - | - | - |
| 1I97 | A | - | - | - | - | - | - | - | - | - | - |
| 1IBK | A | 0.96 | 0.23 | 0.61 | - | - | 0.93 | 0.24 | 0.67 | - | - |
| 1IBL | A | 0.96 | 0.22 | 0.40 | - | - | 0.93 | 0.21 | 0.34 | - | - |
| 1IBM | A | 0.96 | 0.24 | 0.63 | - | - | 0.93 | 0.23 | 0.55 | - | - |
| 1J5E | A | 0.96 | 0.23 | 0.55 | - | - | 0.93 | 0.25 | 0.84 | - | - |
| 1JGO | A | - | - | - | - | - | - | - | - | - | - |
| 1JGP | A | - | - | - | - | - | - | - | - | - | - |
| 1JGQ | A | - | - | - | - | - | - | - | - | - | - |
| 1N32 | A | 0.95 | 0.26 | 0.94 | - | - | 0.91 | 0.29 | 1.32 | - | - |
| 1N33 | A | 0.93 | 0.22 | 0.46 | - | - | 0.89 | 0.25 | 0.82 | - | - |
| 1N34 | A | 0.90 (824/825) | 0.33 (824/825) | 1.68 (824/825) | - | - | 0.84 | 0.41 | 2.65 | - | - |
| 1N36 | A | 0.93 | 0.24 | 0.64 | - | - | 0.87 | 0.30 | 1.34 | - | - |
| 1XMO | A | 0.93 | 0.35 | 2.08 | - | - | 0.89 | 0.36 | 2.18 | - | - |
| 1XMQ | A | 0.96 | 0.26 | 0.87 | - | - | 0.92 | 0.26 | 0.96 | - | - |
| 1XNQ | A | 0.96 | 0.26 | 0.93 | - | - | 0.92 | 0.26 | 0.94 | - | - |
| 1XNR | A | 0.96 | 0.22 | 0.46 | - | - | 0.93 | 0.21 | 0.35 | - | - |
| 2E5L | A | 0.95 | 0.19 | 0.00 | - | - | 0.94 | 0.19 | 0.10 | - | - |
| 2F4V | A | - | - | - | - | - | - | - | - | - | - |
| 2HHH | A | 0.96 | 0.13 | -0.68 | - | - | 0.94 | 0.15 | -0.51 | - | - |
| 2UU9 | A | 0.97 | 0.18 | -0.06 | - | - | 0.94 | 0.18 | -0.07 | - | - |
| 2UUA | A | 0.96 | 0.21 | 0.69 | - | - | 0.93 | 0.21 | 0.64 | - | - |
| 2UUB | A | - | - | - | - | - | - | - | - | - | - |
| 2UUC | A | 0.97 | 0.18 | -0.10 | - | - | 0.94 | 0.19 | 0.11 | - | - |
| 2UXB | A | 0.96 | 0.14 | -0.51 | - | - | 0.92 | 0.16 | -0.27 | - | - |
| 2UXC | A | 0.96 | 0.18 | 0.22 | - | - | 0.93 | 0.20 | 0.45 | - | - |
| 2UXD | A | 0.93 | 0.23 | 0.55 | - | - | 0.88 | 0.25 | 0.83 | - | - |
| 2VQE | A | 0.95 | 0.16 | -0.24 | - | - | 0.91 | 0.18 | 0.12 | - | - |
| 2VQF | A | 0.96 | 0.17 | 0.08 | - | - | 0.92 | 0.19 | 0.37 | - | - |
| 2YKR | A | - | - | - | - | 1.00 | - | - | - | - | 1.00 |
| 2ZM6 | A | 0.96 | 0.16 | -0.35 | - | - | 0.94 | 0.17 | -0.16 | - | - |
| 3J28 | N | - | - | - | - | 0.98 | - | - | - | - | 0.98 |
| 3J29 | N | - | - | - | - | 0.98 | - | - | - | - | 0.93 |
| 3J2A | N | - | - | - | - | 0.97 | - | - | - | - | 0.99 |
| 3J2B | N | - | - | - | - | 0.97 | - | - | - | - | 0.96 |
| 3J2D | N | - | - | - | - | 0.99 | - | - | - | - | 0.99 |
| 3J2E | N | - | - | - | - | 0.98 | - | - | - | - | 0.94 |
| 3J2F | N | - | - | - | - | 0.93 | - | - | - | - | 0.95 |
| 3J2G | N | - | - | - | - | 0.97 | - | - | - | - | 0.93 |
| 3J2H | N | - | - | - | - | 1.00 | - | - | - | - | 0.89 |
| 3J7A | A | - | - | - | - | 0.49 | - | - | - | - | 0.13 |
| 3J80 | 2 | - | - | - | - | 0.97 | - | - | - | - | 0.97 |
| 3J81 | 2 | - | - | - | - | 0.98 | - | - | - | - | 0.98 |
| 3JAM | 2 | - | - | - | - | 0.92 | - | - | - | - | 0.89 |
| 3JAP | 2 | - | - | - | - | 0.94 | - | - | - | - | 0.93 |
| 3JAQ | 2 | - | - | - | - | 0.97 | - | - | - | - | 0.97 |

**Table 2 of 8**

| PDB | | SSU BODY | | | | | SSU HEAD | | | | |
|---|---|---|---|---|---|---|---|---|---|---|---|
| ID | chain | ⟨rscc⟩ | ⟨rsr⟩ | ⟨rsrz⟩ | ⟨Q_score⟩ | ⟨inclusion⟩ | ⟨rscc⟩ | ⟨rsr⟩ | ⟨rsrz⟩ | ⟨Q_score⟩ | ⟨inclusion⟩ |
| 3JD5 | A | - | - | - | - | 0.95 | - | - | - | - | 0.93 |
| 3OTO | A | 0.96 | 0.15 | -0.46 | - | - | 0.93 | 0.17 | -0.24 | - | - |
| 3T1H | A | 0.96 | 0.26 | 0.91 | - | - | 0.92 | 0.27 | 1.03 | - | - |
| 3T1Y | A | 0.95 | 0.26 | 1.28 | - | - | 0.91 | 0.27 | 1.41 | - | - |
| 4A2I | A | - | - | - | - | 0.80 | - | - | - | - | 0.82 |
| 4ADV | A | - | - | - | - | 0.88 | - | - | - | - | 0.81 |
| 4AQY | A | 0.95 | 0.18 | -0.13 | - | - | 0.93 | 0.19 | -0.04 | - | - |
| 4B3M | A | 0.96 | 0.16 | -0.11 | - | - | 0.93 | 0.18 | 0.13 | - | - |
| 4B3R | A | 0.96 | 0.16 | -0.30 | - | - | 0.94 | 0.18 | -0.11 | - | - |
| 4B3S | A | 0.96 | 0.17 | -0.25 | - | - | 0.94 | 0.18 | -0.11 | - | - |
| 4B3T | A | 0.96 | 0.16 | -0.31 | - | - | 0.94 | 0.17 | -0.15 | - | - |
| 4BTS | AA | - | - | - | - | - | - | - | - | - | - |
| 4BTS | BA | - | - | - | - | - | - | - | - | - | - |
| 4BTS | CA | - | - | - | - | - | - | - | - | - | - |
| 4BTS | DA | - | - | - | - | - | - | - | - | - | - |
| 4D5L | 1 | - | - | - | - | 0.95 | - | - | - | - | 0.95 |
| 4D61 | 1 | - | - | - | - | 0.94 | - | - | - | - | 0.91 |
| 4DR1 | A | 0.96 | 0.16 | -0.44<br>(815/824) | - | - | 0.93 | 0.18 | -0.17<br>(405/408) | - | - |
| 4DR2 | A | 0.97 | 0.16 | -0.35<br>(825/834) | - | - | 0.95 | 0.16 | -0.28<br>(401/404) | - | - |
| 4DR3 | A | 0.96 | 0.16 | -0.28<br>(791/797) | - | - | 0.92 | 0.19 | 0.07<br>(387/390) | - | - |
| 4DR4 | A | 0.97 | 0.16 | -0.44<br>(816/825) | - | - | 0.96 | 0.16 | -0.45<br>(410/413) | - | - |
| 4DR5 | A | 0.97 | 0.15 | -0.40<br>(825/834) | - | - | 0.96 | 0.17 | -0.21<br>(409/412) | - | - |
| 4DR6 | A | 0.97 | 0.16 | -0.35<br>(814/821) | - | - | 0.95 | 0.16 | -0.35<br>(404/407) | - | - |
| 4DR7 | A | 0.96 | 0.15 | -0.46<br>(818/827) | - | - | 0.95 | 0.15 | -0.46<br>(398/401) | - | - |
| 4DUY | A | 0.96 | 0.15 | -0.40<br>(816/825) | - | - | 0.95 | 0.16 | -0.30<br>(407/410) | - | - |
| 4DUZ | A | 0.96 | 0.15 | -0.54<br>(810/817) | - | - | 0.93 | 0.17 | -0.29<br>(401/404) | - | - |
| 4DV0 | A | 0.95 | 0.16 | -0.45<br>(822/831) | - | - | 0.93 | 0.17 | -0.25<br>(403/406) | - | - |
| 4DV1 | A | 0.96 | 0.15 | -0.50<br>(807/814) | - | - | 0.93 | 0.17 | -0.23<br>(379/382) | - | - |
| 4DV2 | A | 0.96 | 0.15 | -0.46<br>(799/808) | - | - | 0.92 | 0.19 | -0.04<br>(384/387) | - | - |
| 4DV3 | A | 0.96 | 0.15 | -0.54<br>(779/786) | - | - | 0.93 | 0.18 | -0.12<br>(388/391) | - | - |
| 4DV4 | A | 0.96 | 0.16 | -0.42<br>(811/820) | - | - | 0.94 | 0.18 | -0.16<br>(398/401) | - | - |
| 4DV5 | A | 0.96 | 0.15 | -0.57<br>(786/792) | - | - | 0.93 | 0.17 | -0.25<br>(392/395) | - | - |
| 4DV6 | A | 0.96 | 0.17 | -0.21<br>(816/825) | - | - | 0.95 | 0.17 | -0.18<br>(410/413) | - | - |
| 4DV7 | A | 0.96 | 0.16 | -0.30<br>(784/790) | - | - | 0.91 | 0.19 | 0.09<br>(393/396) | - | - |
| 4GKJ | A | 0.95 | 0.25 | 0.79 | - | - | 0.93 | 0.25 | 0.83 | - | - |
| 4GKK | A | 0.95 | 0.22 | 0.40 | - | - | 0.94 | 0.23 | 0.56 | - | - |
| 4JI0 | A | 0.96 | 0.16 | -0.35<br>(813/822) | - | - | 0.92 | 0.18 | -0.12<br>(395/398) | - | - |

| PDB | | SSU BODY | | | | | SSU HEAD | | | | |
|---|---|---|---|---|---|---|---|---|---|---|---|
| ID | chain | ⟨rscc⟩ | ⟨rsr⟩ | ⟨rsrz⟩ | ⟨Q_score⟩ | ⟨inclusion⟩ | ⟨rscc⟩ | ⟨rsr⟩ | ⟨rsrz⟩ | ⟨Q_score⟩ | ⟨inclusion⟩ |
| 4JI1 | A | 0.97 | 0.15 | -0.43 (787/794) | - | - | 0.94 | 0.18 | -0.09 (393/396) | - | - |
| 4JI2 | A | 0.96 | 0.16 | -0.34 (815/824) | - | - | 0.93 | 0.19 | 0.00 (399/402) | - | - |
| 4JI3 | A | 0.96 | 0.15 | -0.44 (788/795) | - | - | 0.93 | 0.17 | -0.15 (385/388) | - | - |
| 4JI4 | A | 0.95 | 0.17 | -0.25 (811/820) | - | - | 0.93 | 0.20 | 0.05 (402/405) | - | - |
| 4JI5 | A | 0.94 | 0.19 | -0.01 (822/831) | - | - | 0.93 | 0.20 | 0.09 (408/411) | - | - |
| 4JI6 | A | 0.96 | 0.16 | -0.34 (802/811) | - | - | 0.95 | 0.16 | -0.41 (411/414) | - | - |
| 4JI7 | A | 0.96 | 0.17 | -0.23 (804/813) | - | - | 0.95 | 0.17 | -0.29 (414/417) | - | - |
| 4JI8 | A | 0.95 | 0.17 | -0.24 (822/831) | - | - | 0.94 | 0.17 | -0.30 (413/416) | - | - |
| 4JV5 | A | 0.97 | 0.15 | -0.42 | - | - | 0.94 | 0.16 | -0.27 | - | - |
| 4JYA | A | 0.97 | 0.15 | -0.39 | - | - | 0.95 | 0.16 | -0.32 | - | - |
| 4K0K | A | 0.97 | 0.15 | -0.40 | - | - | 0.95 | 0.18 | -0.10 | - | - |
| 4KHP | A | 0.97 | 0.15 | -0.40 | - | - | 0.95 | 0.16 | -0.27 | - | - |
| 4KVB | A | 0.93 | 0.19 | 0.04 (759/768) | - | - | 0.93 | 0.19 | 0.04 (417/420) | - | - |
| 4KZX | i | 0.86 | 0.29 | 1.05 | - | - | 0.81 | 0.33 | 1.45 | - | - |
| 4KZY | i | 0.84 | 0.29 | 1.04 | - | - | 0.79 | 0.22 | 0.38 | - | - |
| 4KZZ | i | 0.84 | 0.32 | 1.31 | - | - | 0.80 | 0.34 | 1.53 | - | - |
| 4LF4 | A | 0.96 | 0.16 | -0.33 (808/815) | - | - | 0.95 | 0.17 | -0.23 (408/411) | - | - |
| 4LF5 | A | 0.95 | 0.15 | -0.47 (802/809) | - | - | 0.92 | 0.18 | -0.16 (386/389) | - | - |
| 4LF6 | A | 0.96 | 0.16 | -0.35 (802/809) | - | - | 0.95 | 0.16 | -0.29 (404/407) | - | - |
| 4LF7 | A | 0.97 | 0.16 | -0.34 (807/814) | - | - | 0.96 | 0.16 | -0.29 (407/410) | - | - |
| 4LF8 | A | 0.97 | 0.16 | -0.34 (807/814) | - | - | 0.96 | 0.16 | -0.29 (407/410) | - | - |
| 4LF9 | A | 0.95 | 0.17 | -0.23 (803/812) | - | - | 0.94 | 0.18 | -0.10 (411/414) | - | - |
| 4LFA | A | 0.95 | 0.17 | -0.33 (807/816) | - | - | 0.93 | 0.18 | -0.11 (405/408) | - | - |
| 4LFB | A | 0.97 | 0.16 | -0.36 (807/816) | - | - | 0.95 | 0.17 | -0.26 (412/415) | - | - |
| 4LFC | A | 0.96 | 0.16 | -0.43 (816/825) | - | - | 0.94 | 0.17 | -0.28 (406/409) | - | - |
| 4NXM | A | 0.96 | 0.15 | -0.52 (812/820) | - | - | 0.93 | 0.17 | -0.22 (401/404) | - | - |
| 4NXN | A | 0.96 | 0.16 | -0.42 (785/790) | - | - | 0.91 | 0.19 | -0.01 (392/395) | - | - |
| 4OX9 | A | 0.92 | 0.19 | -0.03 | - | - | 0.87 | 0.21 | 0.17 | - | - |
| 4UER | A | - | - | - | - | 1.00 | - | - | - | - | 1.00 |
| 4V5O | AA | 0.94 | 0.20 | 0.11 | - | - | 0.94 | 0.20 | 0.11 | - | - |
| 4V5O | BA | 0.94 | 0.20 | 0.04 | - | - | 0.94 | 0.21 | 0.19 | - | - |
| 4V8M | AA | - | - | - | - | 0.84 | - | - | - | - | 0.79 |
| 4V92 | A2 | - | - | - | - | 0.92 | - | - | - | - | 0.86 |
| 4X62 | A | 0.96 | 0.16 | -0.26 (799/808) | - | - | 0.95 | 0.17 | -0.22 (413/416) | - | - |

| PDB | | SSU BODY | | | | | SSU HEAD | | | | |
|---|---|---|---|---|---|---|---|---|---|---|---|
| ID | chain | ⟨rscc⟩ | ⟨rsr⟩ | ⟨rsrz⟩ | ⟨Q_score⟩ | ⟨inclusion⟩ | ⟨rscc⟩ | ⟨rsr⟩ | ⟨rsrz⟩ | ⟨Q_score⟩ | ⟨inclusion⟩ |
| 4X64 | A | 0.97 | 0.16 | -0.36 (817/826) | - | - | 0.95 | 0.17 | -0.19 (412/415) | - | - |
| 4X65 | A | 0.96 | 0.16 | -0.31 (812/821) | - | - | 0.94 | 0.18 | -0.14 (411/414) | - | - |
| 4X66 | A | 0.96 | 0.17 | -0.25 (807/816) | - | - | 0.94 | 0.17 | -0.18 (411/414) | - | - |
| 4YHH | A | 0.94 | 0.14 | -0.62 | - | - | 0.94 | 0.15 | -0.51 | - | - |
| 4YY3 | A | 0.93 | 0.23 | 0.44 | - | - | 0.91 | 0.23 | 0.52 | - | - |
| 5A2Q | 2 | - | - | - | - | 0.97 | - | - | - | - | 0.94 |
| 5AJ3 | A | - | - | - | - | 0.97 | - | - | - | - | 0.96 |
| 5BR8 | A | - | - | - | - | - | - | - | - | - | - |
| 5FLX | 1 | - | - | - | - | 0.85 | - | - | - | - | 0.72 |
| 5IT9 | 2 | - | - | - | - | 0.85 | - | - | - | - | 0.83 |
| 5IWA | A | 0.95 | 0.25 | 0.70 | - | - | 0.95 | 0.23 | 0.52 | - | - |
| 5JB3 | 2 | - | - | - | - | 0.98 | - | - | - | - | 0.94 |
| 5JBH | 2 | - | - | - | - | 0.98 | - | - | - | - | 0.99 |
| 5K0Y | A | - | - | - | - | 0.99 | - | - | - | - | 1.00 |
| 5LMN | A | - | - | - | - | 0.94 | - | - | - | - | 0.94 |
| 5LMO | A | - | - | - | - | 0.99 | - | - | - | - | 0.97 |
| 5LMP | A | - | - | - | - | 0.97 | - | - | - | - | 0.97 |
| 5LMQ | A | - | - | - | - | 0.97 | - | - | - | - | 0.96 |
| 5LMR | A | - | - | - | - | 0.96 | - | - | - | - | 0.95 |
| 5LMS | A | - | - | - | - | 0.98 | - | - | - | - | 0.98 |
| 5LMT | A | - | - | - | - | 0.94 | - | - | - | - | 0.93 |
| 5LMU | A | - | - | - | - | 0.90 | - | - | - | - | 0.90 |
| 5LMV | A | - | - | - | - | 0.82 | - | - | - | - | 0.81 |
| 5ME0 | A | - | - | - | - | 0.99 (913/916) | - | - | - | - | 1.00 (390/393) |
| 5ME1 | A | - | - | - | - | 0.99 (913/916) | - | - | - | - | 0.99 (390/393) |
| 5MMJ | a | - | - | - | - | 0.99 | - | - | - | - | 0.99 |
| 5MY1 | A | - | - | - | - | 0.97 | - | - | - | - | 0.94 |
| 5NO2 | A | - | - | - | - | 0.97 (873/876) | - | - | - | - | 0.93 (376/379) |
| 5NO3 | A | - | - | - | - | 0.98 (878/881) | - | - | - | - | 0.97 (361/363) |
| 5NO4 | A | - | - | - | - | 0.97 (868/871) | - | - | - | - | 0.96 (389/392) |
| 5O5J | A | - | - | - | - | 0.86 | - | - | - | - | 0.81 |
| 5OA3 | 2 | - | - | - | - | 0.93 | - | - | - | - | 0.79 |
| 5OPT | E | - | - | - | - | 0.83 | - | - | - | - | 0.72 |
| 5T2A | 2 | - | - | - | - | 0.80 | - | - | - | - | 0.41 |
| 5UZ4 | A | - | - | - | - | 0.89 | - | - | - | - | 0.84 |
| 5VYC | i1 | 0.83 | 0.16 | -0.18 | - | - | 0.81 | 0.17 | -0.13 | - | - |
| 5VYC | i2 | 0.84 | 0.16 | -0.21 | - | - | 0.80 | 0.16 | -0.18 | - | - |
| 5VYC | i3 | 0.83 | 0.16 | -0.26 | - | - | 0.79 | 0.15 | -0.31 | - | - |
| 5VYC | i4 | 0.82 | 0.16 | -0.20 | - | - | 0.75 | 0.16 | -0.22 | - | - |
| 5VYC | i5 | 0.83 | 0.16 | -0.26 | - | - | 0.80 | 0.16 | -0.20 | - | - |
| 5VYC | i6 | 0.82 | 0.15 | -0.27 | - | - | 0.79 | 0.17 | -0.15 | - | - |
| 5WNP | A | 0.95 | 0.15 | -0.48 (820/829) | - | - | 0.94 | 0.16 | -0.35 (413/416) | - | - |
| 5WNQ | A | 0.94 | 0.16 | -0.42 (803/812) | - | - | 0.91 | 0.19 | -0.09 (404/407) | - | - |
| 5WNR | A | - | - | - | - | - | - | - | - | - | - |
| 5WNS | A | - | - | - | - | - | - | - | - | - | - |

**Table 5 of 8**

| PDB | | SSU BODY | | | | | SSU HEAD | | | | |
|---|---|---|---|---|---|---|---|---|---|---|---|
| ID | chain | ⟨rscc⟩ | ⟨rsr⟩ | ⟨rsrz⟩ | ⟨Q_score⟩ | ⟨inclusion⟩ | ⟨rscc⟩ | ⟨rsr⟩ | ⟨rsrz⟩ | ⟨Q_score⟩ | ⟨inclusion⟩ |
| 5WNT | A | 0.96 | 0.14 | -0.56 (818/827) | - | - | 0.95 | 0.15 | -0.48 (413/416) | - | - |
| 5WNU | A | 0.96 | 0.13 | -0.74 (819/828) | - | - | 0.94 | 0.14 | -0.59 (411/414) | - | - |
| 5WNV | A | 0.96 | 0.14 | -0.55 (817/826) | - | - | 0.95 | 0.15 | -0.45 (413/416) | - | - |
| 5X8R | a | - | - | - | - | 0.91 | - | - | - | - | 0.91 |
| 5XXU | 2 | - | - | - | - | 0.91 | - | - | - | - | 0.82 |
| 5XYI | 2 | - | - | - | - | 0.83 | - | - | - | - | 0.85 |
| 5XYU | A | - | - | - | - | 0.84 | - | - | - | - | 0.80 |
| 5ZEU | a | - | - | - | - | 0.88 | - | - | - | - | 0.85 |
| 6AWB | A | - | - | - | - | 0.96 | - | - | - | - | 0.96 |
| 6AWC | A | - | - | - | - | 0.95 | - | - | - | - | 0.97 |
| 6AWD | A | - | - | - | - | 1.00 | - | - | - | - | 1.00 |
| 6AZ1 | 1 | - | - | - | - | 0.94 (626/628) | - | - | - | - | 0.92 (164/165) |
| 6CAO | A | 0.96 | 0.19 | 0.02 (816/824) | - | - | 0.95 | 0.20 | 0.15 (412/415) | - | - |
| 6CAP | A | 0.95 | 0.18 | -0.12 (807/816) | - | - | 0.94 | 0.18 | -0.12 (408/411) | - | - |
| 6CAQ | A | 0.95 | 0.18 | -0.08 (807/816) | - | - | 0.93 | 0.20 | 0.17 (408/411) | - | - |
| 6CAR | A | 0.96 | 0.19 | 0.04 (812/821) | - | - | 0.94 | 0.20 | 0.18 (410/413) | - | - |
| 6CAS | A | 0.95 | 0.16 | -0.38 (816/825) | - | - | 0.93 | 0.18 | -0.19 (406/409) | - | - |
| 6DTI | A | 0.95 | 0.19 | -0.04 | - | - | 0.93 | 0.19 | -0.05 | - | - |
| 6DZK | A | - | - | - | - | 0.97 | - | - | - | - | 0.97 |
| 6EML | 2 | - | - | - | - | 0.87 | - | - | - | - | 0.58 |
| 6FAI | 2 | - | - | - | - | 1.00 | - | - | - | - | 1.00 |
| 6FEC | A | - | - | - | - | 0.98 | - | - | - | - | 0.97 |
| 6FYX | 2 | - | - | - | - | 0.92 (524/526) | - | - | - | - | 0.91 (276/279) |
| 6FYY | 2 | - | - | - | - | 0.93 (530/532) | - | - | - | - | 0.93 (278/281) |
| 6G18 | 2 | - | - | - | - | 0.92 | - | - | - | - | 0.89 |
| 6G4S | 2 | - | - | - | - | 0.94 | - | - | - | - | 0.89 |
| 6G4W | 2 | - | - | - | - | 0.90 | - | - | - | - | 0.92 |
| 6G51 | 2 | - | - | - | - | 0.96 | - | - | - | - | 0.91 |
| 6G53 | 2 | - | - | - | - | 0.90 | - | - | - | - | 0.86 |
| 6G5H | 2 | - | - | - | - | 0.93 | - | - | - | - | 0.88 |
| 6G5I | 2 | - | - | - | - | 0.99 | - | - | - | - | 0.98 |
| 6GAZ | AA | - | - | - | - | 0.98 | - | - | - | - | 0.96 |
| 6GSM | 2 | - | - | - | - | 0.86 | - | - | - | - | 0.81 |
| 6GSN | 2 | - | - | - | - | 0.98 | - | - | - | - | 0.97 |
| 6HRM | 1 | - | - | - | - | 0.78 (927/931) | - | - | - | - | 0.49 (415/418) |
| 6MKN | A | 0.95 | 0.19 | 0.00 | - | - | 0.93 | 0.19 | 0.03 | - | - |
| 6MPF | A | 0.96 | 0.18 | -0.08 | - | - | 0.94 | 0.18 | -0.12 | - | - |
| 6MPI | A | 0.95 | 0.18 | -0.03 | - | - | 0.94 | 0.18 | -0.07 | - | - |
| 6NEQ | A | - | - | - | - | 0.93 | - | - | - | - | 0.96 |
| 6NF8 | A | - | - | - | - | 0.97 | - | - | - | - | 0.97 |
| 6NQB | A | - | - | - | - | 0.92 | - | - | - | - | 0.63 |
| 6NY6 | A | 0.95 | 0.15 | -0.46 | - | - | 0.92 | 0.16 | -0.35 | - | - |
| 6O7K | g | - | - | - | - | 0.99 | - | - | - | - | 0.99 |

**Table 6 of 8**

| PDB | | SSU BODY | | | | | SSU HEAD | | | | |
|---|---|---|---|---|---|---|---|---|---|---|---|
| ID | chain | ⟨rscc⟩ | ⟨rsr⟩ | ⟨rsrz⟩ | ⟨Q_score⟩ | ⟨inclusion⟩ | ⟨rscc⟩ | ⟨rsr⟩ | ⟨rsrz⟩ | ⟨Q_score⟩ | ⟨inclusion⟩ |
| 6OKK | A | - | - | - | - | 0.71 | - | - | - | - | 0.21 |
| 6P4G | 2 | - | - | - | - | 0.94 | - | - | - | - | 0.93 |
| 6P4H | 2 | - | - | - | - | 0.95 | - | - | - | - | 0.95 |
| 6RBD | 2 | - | - | - | - | 1.00 | - | - | - | - | 0.94 |
| 6RBE | 2 | - | - | - | - | 0.92 | - | - | - | - | 0.80 |
| 6RW4 | A | - | - | - | - | 0.89 (347/348) | - | - | - | - | 0.87 |
| 6RW5 | A | - | - | - | - | 0.94 (323/324) | - | - | - | - | 0.93 |
| 6SPC | a | - | - | - | - | 0.99 | - | - | - | - | 0.93 |
| 6SPE | a | - | - | - | - | 1.00 | - | - | - | - | 1.00 |
| 6SW9 | 2 | - | - | - | - | 1.00 (334/345) | - | - | - | - | 1.00 (163/170) |
| 6SWC | 2 | - | - | - | - | 1.00 (346/355) | - | - | - | - | 1.00 (187/194) |
| 6TMF | A | - | - | - | - | 0.98 | - | - | - | - | 0.97 |
| 6V3E | sN1 | - | - | - | - | 0.86 (550/551) | - | - | - | - | 0.96 (314/317) |
| 6W2S | A | - | - | - | - | 0.97 | - | - | - | - | 0.93 |
| 6W2T | a | - | - | - | - | 0.97 | - | - | - | - | 0.94 |
| 6W6K | A | - | - | - | - | 0.73 | - | - | - | - | 0.66 |
| 6W77 | A | - | - | - | - | 0.86 | - | - | - | - | 0.64 |
| 6W7M | A | - | - | - | - | 0.74 | - | - | - | - | 0.64 |
| 6W7N | A | - | - | - | - | 0.87 | - | - | - | - | 0.54 |
| 6WDR | 2 | - | - | - | - | 0.99 | - | - | - | - | 0.99 |
| 6XE0 | W | - | - | - | - | 0.99 | - | - | - | - | 0.99 |
| 6Y7C | 2 | - | - | - | - | 0.98 | - | - | - | - | 0.78 |
| 6YAL | 2 | - | - | - | - | 0.93 | - | - | - | - | 0.91 |
| 6YAM | 2 | - | - | - | - | 0.84 | - | - | - | - | 0.83 |
| 6YAN | 2 | - | - | - | - | 0.96 | - | - | - | - | 0.93 |
| 6YW5 | aa | - | - | - | - | 0.84 | - | - | - | - | 0.82 |
| 6ZCE | A | - | - | - | - | 0.94 | - | - | - | - | 0.95 |
| 6ZJ3 | S1 | - | - | - | - | 0.96 (652/653) | - | - | - | - | 0.96 |
| 6ZLW | 2 | - | - | - | - | 1.00 | - | - | - | - | 1.00 |
| 6ZMT | 2 | - | - | - | - | 0.99 | - | - | - | - | 0.98 |
| 6ZMW | A | - | - | - | - | 0.97 (580/582) | - | - | - | - | 0.93 |
| 6ZN5 | 2 | - | - | - | - | 0.93 | - | - | - | - | 0.97 |
| 6ZOJ | 2 | - | - | - | - | 0.97 | - | - | - | - | 0.99 |
| 6ZON | 2 | - | - | - | - | 1.00 | - | - | - | - | 0.90 |
| 6ZP4 | 2 | - | - | - | - | 1.00 | - | - | - | - | 1.00 |
| 6ZU9 | 2 | - | - | - | - | 1.00 | - | - | - | - | 1.00 |
| 6ZUO | 2 | - | - | - | - | 0.97 | - | - | - | - | 0.94 |
| 6ZV6 | 2 | - | - | - | - | 0.98 (554/555) | - | - | - | - | 0.98 |
| 6ZVH | 2 | - | - | - | - | 1.00 | - | - | - | - | 0.99 |
| 6ZVI | h | - | - | - | - | 0.96 | - | - | - | - | 0.96 |
| 6ZVJ | 2 | - | - | - | - | 0.97 | - | - | - | - | 0.91 |
| 6ZXD | 2 | - | - | - | - | 0.98 | - | - | - | - | 0.96 |
| 6ZXE | 2 | - | - | - | - | 0.99 | - | - | - | - | 0.89 |
| 6ZXF | 2 | - | - | - | - | 1.00 | - | - | - | - | 1.00 |
| 6ZXG | 2 | - | - | - | - | 0.99 (563/565) | - | - | - | - | 0.99 (194/195) |
| 6ZXH | 2 | - | - | - | - | 0.98 (557/559) | - | - | - | - | 0.98 (179/180) |

**Table 7 of 8**

| PDB | | SSU BODY | | | | | SSU HEAD | | | | |
|---|---|---|---|---|---|---|---|---|---|---|---|
| ID | chain | ⟨rscc⟩ | ⟨rsr⟩ | ⟨rsrz⟩ | ⟨Q_score⟩ | ⟨inclusion⟩ | ⟨rscc⟩ | ⟨rsr⟩ | ⟨rsrz⟩ | ⟨Q_score⟩ | ⟨inclusion⟩ |
| 7A09 | 2 | - | - | - | - | 1.00 | - | - | - | - | 0.98 |
| 7A1G | 2 | - | - | - | - | 0.78 | - | - | - | - | 0.61 |
| 7ASE | 0 | - | - | - | - | 0.89 | - | - | - | - | 0.86 |
| 7BOE | A | - | - | - | - | 0.97 (946/949) | - | - | - | - | 0.33 (408/411) |
| 7BOH | A | - | - | - | - | 0.98 (909/910) | - | - | - | - | 0.46 (411/414) |
| 7DUG | A | - | - | - | - | - | - | - | - | - | - |
| 7DUH | A | - | - | - | - | - | - | - | - | - | - |
| 7DUI | A | 0.96 | 0.12 | -0.84 (808/817) | - | - | 0.95 | 0.13 | -0.74 (406/409) | - | - |
| 7DUJ | A | - | - | - | - | - | - | - | - | - | - |
| 7DUK | A | - | - | - | - | - | - | - | - | - | - |
| 7DUL | A | 0.96 | 0.12 | -0.89 (806/815) | - | - | 0.95 | 0.13 | -0.77 (405/408) | - | - |
| 7JQB | A | - | - | - | - | 0.99 | - | - | - | - | 0.89 |
| 7JQC | A | - | - | - | - | 0.94 | - | - | - | - | 0.88 |
| 7K5I | 2 | - | - | - | - | 0.98 | - | - | - | - | 0.97 |
| 7KWG | a | - | - | - | - | 0.95 | - | - | - | - | 0.75 |
| 7M4U | a | - | - | - | - | 0.96 (717/719) | - | - | - | - | 0.98 (362/365) |
| 7NAR | A | - | - | - | - | 1.00 (888/890) | - | - | - | - | 1.00 (396/399) |
| 7NAT | A | - | - | - | - | 1.00 (784/785) | - | - | - | - | 0.99 (395/398) |
| 7NAU | A | - | - | - | - | 1.00 | - | - | - | - | 0.99 (395/398) |
| 7NAV | A | - | - | - | - | 1.00 | - | - | - | - | 0.99 (401/404) |
| 7NAX | A | - | - | - | - | - | - | - | - | - | - |
| 7OE0 | A | - | - | - | - | 0.99 | - | - | - | - | 0.88 |
| 7OE1 | A | - | - | - | - | 0.96 | - | - | - | - | 0.75 |
| 7OOC | 5 | - | - | - | - | 0.00 | - | - | - | - | 0.00 |
| 7P2E | A | - | - | - | - | 0.92 (327/329) | - | - | - | - | 0.90 |
| 7PNT | A | - | - | - | - | 0.87 | - | - | - | - | 0.82 |
| 7PNU | A | - | - | - | - | 0.88 | - | - | - | - | 0.81 |
| 7PNV | A | - | - | - | - | 0.92 | - | - | - | - | 0.91 |
| 7PNW | A | - | - | - | - | 0.88 | - | - | - | - | 0.83 |
| 7PNX | A | - | - | - | - | 0.93 | - | - | - | - | 0.90 |
| 7PNY | A | - | - | - | - | 0.92 (340/341) | - | - | - | - | 0.91 |
| 7PNZ | A | - | - | - | - | 0.91 (344/345) | - | - | - | - | 0.90 |
| 7PO0 | A | - | - | - | - | 0.91 (345/346) | - | - | - | - | 0.89 |
| 7PO1 | A | - | - | - | - | 0.91 (315/316) | - | - | - | - | 0.88 |
| 7PO2 | A | - | - | - | - | 0.90 (330/331) | - | - | - | - | 0.88 |
| 7PO3 | A | - | - | - | - | 0.87 (322/323) | - | - | - | - | 0.84 |
| 7PWF | 2 | - | - | - | - | 0.93 (469/471) | - | - | - | - | 0.74 (243/244) |
| 7QP6 | A | - | - | - | - | 0.99 (594/595) | - | - | - | - | 0.99 |

**Table 8 of 8**

| PDB | | SSU BODY | | | | | SSU HEAD | | | | |
|---|---|---|---|---|---|---|---|---|---|---|---|
| ID | chain | ⟨rscc⟩ | ⟨rsr⟩ | ⟨rsrz⟩ | ⟨Q_score⟩ | ⟨inclusion⟩ | ⟨rscc⟩ | ⟨rsr⟩ | ⟨rsrz⟩ | ⟨Q_score⟩ | ⟨inclusion⟩ |
| 7QP7 | A | - | - | - | - | 0.99 (615/616) | - | - | - | - | 0.99 (258/259) |
| 7SYG | 2 | - | - | - | - | 0.94 | - | - | - | - | 0.87 |
| 7SYH | 2 | - | - | - | - | 0.86 | - | - | - | - | 0.74 |
| 7SYI | 2 | - | - | - | - | 0.89 | - | - | - | - | 0.78 |
| 7SYJ | 2 | - | - | - | - | 0.98 | - | - | - | - | 0.96 |
| 7SYK | 2 | - | - | - | - | 0.97 | - | - | - | - | 0.94 |
| 7SYL | 2 | - | - | - | - | 0.94 | - | - | - | - | 0.90 |
| 7SYM | 2 | - | - | - | - | 0.90 | - | - | - | - | 0.89 |
| 7SYN | 2 | - | - | - | - | 0.94 | - | - | - | - | 0.89 |
| 7SYO | 2 | - | - | - | - | 0.87 | - | - | - | - | 0.84 |
| 7SYP | 2 | - | - | - | - | 0.90 | - | - | - | - | 0.86 |
| 7SYQ | 2 | - | - | - | - | 0.97 | - | - | - | - | 0.97 |
| 7SYR | 2 | - | - | - | - | 0.92 | - | - | - | - | 0.90 |
| 7SYS | 2 | - | - | - | - | 0.96 | - | - | - | - | 0.96 |
| 7SYT | 2 | - | - | - | - | 0.88 | - | - | - | - | 0.87 |
| 7SYU | 2 | - | - | - | - | 0.92 | - | - | - | - | 0.91 |
| 7SYV | 2 | - | - | - | - | 0.92 | - | - | - | - | 0.89 |
| 7SYW | 2 | - | - | - | - | 0.96 | - | - | - | - | 0.96 |
| 7SYX | 2 | - | - | - | - | 0.88 | - | - | - | - | 0.85 |
| 7TQL | 2 | - | - | - | - | 0.94 | - | - | - | - | 0.95 |
| 7UPH | I | - | - | - | - | 0.90 (755/756) | - | - | - | - | 0.78 (391/394) |
| 7V2L | A | - | - | - | - | 0.92 | - | - | - | - | 0.93 |
| 7V2M | A | - | - | - | - | 0.94 | - | - | - | - | 0.93 |
| 7V2N | A | - | - | - | - | 0.96 | - | - | - | - | 0.84 |
| 7V2O | A | - | - | - | - | 0.94 | - | - | - | - | 0.92 |
| 7V2P | A | - | - | - | - | 0.98 | - | - | - | - | 0.97 |
| 7V2Q | A | - | - | - | - | 0.98 | - | - | - | - | 0.95 |
| 7WTT | 2 | - | - | - | 0.59 | 1.00 | - | - | - | 0.52 | 1.00 |
| 7WTU | 2 | - | - | - | 0.60 | 1.00 | - | - | - | 0.50 | 1.00 |
| 7WTV | 2 | - | - | - | 0.55 | 0.99 | - | - | - | 0.37 | 0.97 |
| 7WTX | 2 | - | - | - | 0.63 | 1.00 | - | - | - | 0.49 | 0.99 |
| 7WTZ | 2 | - | - | - | 0.63 | 1.00 | - | - | - | 0.51 | 0.98 |
| 7WU0 | 2 | - | - | - | 0.57 | 1.00 | - | - | - | 0.39 | 0.99 |
| 7ZAG | 2 | - | - | - | - | 1.00 (393/411) | - | - | - | - | 1.00 (222/228) |
| 7ZAH | 2 | - | - | - | 0.66 | 1.00 (388/405) | - | - | - | 0.66 | 1.00 (222/228) |
| 7ZAI | 2 | - | - | - | - | 1.00 (391/409) | - | - | - | - | 1.00 (222/228) |
| 7ZHG | 2 | - | - | - | - | 1.00 (435/456) | - | - | - | - | 1.00 (224/230) |
