# Supplementary material for: Ratchet, swivel, tilt and roll: a complete description of subunit rotation in the ribosome
Source: Nucleic Acids Res. 2022 Dec 30;51(2):919–34. doi: 10.1093/nar/gkac1211 (PMC9881166; doi:10.1093/nar/gkac1211)
Supplement: gkac1211_Supplemental_Files [file gkac1211_supplemental_files.zip › AppendixG.pdf]

Appendix G: RCSB validation data for ribosome structures described with the RAD method - Isolated LSUs

Below are tables that provide summaries of validation statistics for 375 structures.

For each rRNA chain, the average values were calculated for all residues that were identied as being part of a "core". Averages were calculated from the values provided by the RCSB validation reports, which contain per-residue values.

If a validation report did not contain any values for the core residues, then the entry is listed with a "-".

If validation quantities were only available for a fraction of the core residues, then the number of found values and the number of core residues is shown in parentheses.

Quantities calculated

- ⟨rscc⟩: average rscc value of core residues (X-RAY)
- ⟨rsr⟩: average rsr value of core residues (X-RAY)
- ⟨rsrz⟩: average rsrz value of core residues (X-RAY)
- ⟨Q_score⟩: average Q-score value of core residues (EM)
- ⟨inclusion⟩: average inclusion value of core residues (EM)

Table 1 of 9

PDB		LSU				
ID	chain	⟨rscc⟩	⟨rsr⟩	⟨rsrz⟩	⟨Q_score⟩	⟨inclusion⟩
1FFK	0	-	-	-	-	-
1J5A	A	-	-	-	-	-
1JJ2	0	0.98	0.15	-0.49	-	-
1JZX	A	-	-	-	-	-
1JZY	A	-	-	-	-	-
1JZZ	A	-	-	-	-	-
1K01	A	-	-	-	-	-
1K73	A	0.97	0.16	-0.36	-	-
1K8A	A	0.96	0.17	-0.19	-	-
1K9M	A	0.96	0.17	-0.25	-	-
1KC8	A	0.97	0.15	-0.43	-	-
1KD1	A	0.96	0.17	-0.25	-	-
1KQS	0	0.97	0.15	-0.42	-	-
1M1K	A	0.96	0.17	-0.22	-	-
1M90	A	0.98	0.15	-0.24	-	-
1N8R	A	-	-	-	-	-
1NJI	A	0.97	0.14	-0.51	-	-
1NJM	0	-	-	-	-	-
1NJN	0	-	-	-	-	-
1NJO	0	-	-	-	-	-
1NJP	0	-	-	-	-	-
1NKW	0	-	-	-	-	-
1NWX	0	-	-	-	-	-
1NWY	0	-	-	-	-	-
1OND	0	-	-	-	-	-
1P9X	0	-	-	-	-	-
1Q7Y	A	0.96	0.16	-0.33	-	-
1Q81	A	0.97	0.16	-0.07	-	-
1Q82	A	0.97	0.16	-0.11	-	-
1Q86	A	0.93	0.19	0.03	-	-
1QVF	0	0.97	0.16	-0.30	-	-
1QVG	0	0.97	0.15	-0.28	-	-
1S72	0	0.98	0.15	-0.47 (1559/1564)	-	-
1SM1	0	-	-	-	-	-
1VQ4	0	0.98	0.14	-0.69 (1571/1576)	-	-
1VQ5	0	0.93	0.18	0.01 (1555/1560)	-	-
1VQ6	0	0.97	0.15	-0.53 (1562/1567)	-	-
1VQ7	0	0.97	0.14	-0.63 (1567/1572)	-	-
1VQ8	0	0.97	0.12	-0.36 (1558/1563)	-	-
1VQ9	0	0.97	0.13	-0.73 (1559/1564)	-	-
1VQK	0	0.97	0.13	-0.20 (1555/1560)	-	-
1VQL	0	0.97	0.13	-0.21 (1562/1567)	-	-
1VQM	0	0.97	0.13	-0.23 (1566/1571)	-	-
1VQN	0	0.97	0.13	-0.75 (1555/1560)	-	-

PDB		LSU				
ID	chain	⟨rscc⟩	⟨rsr⟩	⟨rsrz⟩	⟨Q_score⟩	⟨inclusion⟩
1VQO	0	0.97	0.12	-0.36 (1554/1559)	-	-
1VQP	0	0.97	0.13	-0.29 (1563/1568)	-	-
1W2B	0	-	-	-	-	-
1XBP	0	-	-	-	-	-
1Y69	0	-	-	-	-	-
1YHQ	0	0.97	0.14	-0.68 (1551/1556)	-	-
1YI2	0	0.98	0.14	-0.66 (1560/1565)	-	-
1YIJ	0	0.98	0.14	-0.63 (1571/1576)	-	-
1YIT	0	0.97	0.16	-0.11 (1580/1585)	-	-
1YJ9	0	0.97	0.14	-0.32 (1569/1574)	-	-
1YJN	0	0.98	0.16	-0.35 (1569/1574)	-	-
1YJW	0	0.98	0.14	-0.36 (1575/1580)	-	-
1Z58	2	-	-	-	-	-
2AAR	0	-	-	-	-	-
2D3O	0	-	-	-	-	-
2J28	B	-	-	-	-	0.89
2O43	A	-	-	-	-	-
2O44	A	-	-	-	-	-
2O45	A	-	-	-	-	-
2OGM	0	-	-	-	-	-
2OGN	0	-	-	-	-	-
2OGO	0	-	-	-	-	-
2OTJ	0	0.95	0.13	-0.49 (1573/1578)	-	-
2OTL	0	0.97	0.16	-0.40 (1563/1568)	-	-
2QA4	0	0.95	0.17	-0.22 (1546/1550)	-	-
2QEX	0	0.97	0.11	-0.79 (1427/1432)	-	-
2ZJP	X	0.89	0.20	0.15	-	-
2ZJQ	X	0.94	0.15	-0.40	-	-
2ZJR	X	0.95	0.14	-0.42	-	-
3BBX	B	-	-	-	-	0.89
3CC2	0	-	-	-	-	-
3CC4	0	0.97	0.16	-0.40 (1572/1577)	-	-
3CC7	0	-	-	-	-	-
3CCE	0	-	-	-	-	-
3CCJ	0	0.96	0.14	-0.54 (1580/1585)	-	-
3CCL	0	0.98	0.12	-0.64 (1543/1548)	-	-
3CCM	0	0.97	0.16	-0.25 (1546/1551)	-	-
3CCQ	0	0.97	0.14	-0.34 (1557/1562)	-	-

PDB		LSU				
ID	chain	⟨rscc⟩	⟨rsr⟩	⟨rsrz⟩	⟨Q_score⟩	⟨inclusion⟩
3CCR	0	0.97	0.12	-0.79 (1561/1566)	-	-
3CCS	0	0.97	0.13	-0.58 (1563/1568)	-	-
3CCU	0	0.98	0.12	-0.74 (1562/1567)	-	-
3CCV	0	0.98	0.12	-0.67 (1569/1574)	-	-
3CD6	0	0.97	0.13	-0.82 (1561/1566)	-	-
3CF5	X	0.95	0.14	-0.53	-	-
3CMA	0	0.97	0.11	-0.75 (1562/1567)	-	-
3CME	0	0.97	0.13	-0.58 (1573/1578)	-	-
3CPW	0	0.97	0.15	-0.47	-	-
3CXC	0	0.97	0.14	-0.64	-	-
3DLL	X	0.95	0.14	-0.64	-	-
3FWO	A	-	-	-	-	-
3G4S	0	0.96	0.14	-0.61 (1580/1585)	-	-
3G6E	0	0.97	0.16	-0.40 (1568/1573)	-	-
3G71	0	0.97	0.12	-0.66 (1575/1580)	-	-
3I55	0	0.93	0.16	-0.31 (1571/1576)	-	-
3I56	0	0.97	0.12	-0.71 (1555/1560)	-	-
3J3V	A	-	-	-	-	0.96
3J3W	A	-	-	-	-	0.98
3J5L	A	-	-	-	-	0.69
3J6B	A	-	-	-	-	0.85
3J79	A	-	-	-	-	0.67
3J7O	5	-	-	-	-	0.31
3J7Q	5	-	-	-	-	0.91
3J7Y	A	-	-	-	-	0.98
3J7Z	A	-	-	-	-	0.75
3J8G	B	-	-	-	-	0.78
3J92	5	-	-	-	-	0.86
3JCS	1	-	-	-	-	0.93
3JCS	2	-	-	-	-	0.88
3JCT	1	-	-	-	-	0.92
3JQ4	A	-	-	-	-	-
3OW2	0	-	-	-	-	-
3PIO	X	0.94	0.16	-0.28	-	-
3PIP	X	0.93	0.19	0.05	-	-
4ADX	0	-	-	-	-	0.62
4CE4	A	-	-	-	-	0.59
4CSU	B	-	-	-	-	1.00
4D5Y	2	-	-	-	-	0.96
4D67	2	-	-	-	-	0.95
4IO9	X	0.95	0.16	-0.32	-	-
4IOA	X	0.95	0.16	-0.34	-	-
4IOC	X	0.93	0.17	-0.21	-	-

PDB		LSU				
ID	chain	⟨rscc⟩	⟨rsr⟩	⟨rsrz⟩	⟨Q_score⟩	⟨inclusion⟩
4U67	X	0.94	0.15	-0.58	-	-
4UY8	A	-	-	-	-	0.47
4V19	A	-	-	-	-	0.95
4V7F	1	-	-	-	-	0.92
4V8P	A1	0.94	0.19	-0.07	-	-
4V8P	D1	0.95	0.19	-0.05	-	-
4V8P	F1	0.94	0.20	0.05	-	-
4V8P	H1	0.90	0.22	0.31	-	-
4V8T	5	-	-	-	-	0.87
4V91	1	-	-	-	-	0.67
4V9F	0	0.98	0.16	-0.26 (1570/1575)	-	-
4WCE	X	0.95	0.15	-0.46	-	-
4WF9	X	0.95	0.16	-0.37	-	-
4WFA	X	0.94	0.15	-0.47	-	-
4WFB	X	0.95	0.16	-0.28	-	-
4WFN	X	0.94	0.18	-0.19	-	-
5ADY	B	-	-	-	-	0.90
5AKA	B	-	-	-	-	0.83
5AN9	N	-	-	-	-	0.91
5ANB	N	-	-	-	-	0.93
5ANC	N	-	-	-	-	0.93
5APN	5	-	-	-	-	0.95
5APO	5	-	-	-	-	0.91
5DM6	X	0.94	0.14	-0.36	-	-
5DM7	X	0.90	0.16	-0.28	-	-
5FL8	x	-	-	-	-	0.85
5GAD	A	-	-	-	-	1.00
5GAE	A	-	-	-	-	0.99
5GAF	A	-	-	-	-	0.90
5GAG	A	-	-	-	-	0.99
5GAH	A	-	-	-	-	0.96
5GAK	1	-	-	-	-	0.29
5H1S	A	-	-	-	-	0.85
5H4P	1	-	-	-	-	0.49
5HKV	X	0.95	0.16	-0.36	-	-
5HL7	X	0.95	0.15	-0.49	-	-
5JCS	x	-	-	-	-	0.85
5JVG	X	0.95	0.13	-0.65	-	-
5JVH	X	0.94	0.17	-0.25	-	-
5MLC	A	-	-	-	-	0.75
5MMI	A	-	-	-	-	0.96
5NCO	A	-	-	-	-	0.94
5NRG	X	0.94	0.18	-0.14	-	-
5O60	A	-	-	-	-	0.88
5OOL	A	-	-	-	-	0.72
5OOM	A	-	-	-	-	0.79
5T5H	A	-	-	-	-	0.95 (645/649)
5T5H	B	-	-	-	-	0.92 (527/529)
5T62	A	-	-	-	-	0.98
5T6R	A	-	-	-	-	0.88
5UMD	A	-	-	-	-	0.87
5V7Q	A	-	-	-	-	0.58

Table 5 of 9

PDB		LSU				
ID	chain	⟨rscc⟩	⟨rsr⟩	⟨rsrz⟩	⟨Q_score⟩	⟨inclusion⟩
5X8T	A	-	-	-	-	0.82
5XXB	1	-	-	-	-	0.91
5XY3	1	-	-	-	-	0.88
5XYM	A	-	-	-	-	0.88
5Z3G	A	-	-	-	-	0.89
5ZET	A	-	-	-	-	0.93
5ZZM	N	-	-	-	-	0.94
6AZ3	1	-	-	-	-	0.96
6AZ3	2	-	-	-	-	0.92
6C0F	1	-	-	-	-	0.95
6C4H	A	-	-	-	-	0.87 (611/618)
6CB1	1	-	-	-	-	0.83
6DDD	1	-	-	-	-	0.88
6DDG	1	-	-	-	-	0.95
6DZP	A	-	-	-	-	0.95
6ELZ	1	-	-	-	-	0.87
6EM1	1	-	-	-	-	0.93
6EM3	1	-	-	-	-	0.95
6EM4	1	-	-	-	-	0.94
6EM5	1	-	-	-	-	0.96
6FRK	5	-	-	-	-	0.93
6FT6	1	-	-	-	-	0.99
6FTG	u	-	-	-	-	0.76
6FTI	u	-	-	-	-	0.82
6FTJ	u	-	-	-	-	0.80
6GB2	BA	-	-	-	-	0.91
6GBZ	A	-	-	-	-	0.88
6GC0	A	-	-	-	-	0.88
6GC4	A	-	-	-	-	0.85
6GC6	A	-	-	-	-	0.91
6GC7	A	-	-	-	-	0.91
6GC8	A	-	-	-	-	0.90
6HD7	1	-	-	-	-	0.96
6HMA	A	-	-	-	-	0.88 (1132/1134)
6I0Y	A	-	-	-	-	0.91
6I9R	A	-	-	-	-	0.87 (993/997)
6LQM	2	-	-	-	-	0.99 (1225/1232)
6LSR	2	-	-	-	-	0.97 (1186/1193)
6LSS	2	-	-	-	-	0.97 (957/961)
6LU8	2	-	-	-	-	0.98 (982/987)
6M62	1	-	-	-	-	0.95
6N8J	1	-	-	-	-	0.96
6N8K	1	-	-	-	-	0.94
6N8L	1	-	-	-	-	0.94
6N8M	A	-	-	-	-	0.96
6N8N	A	-	-	-	-	0.93
6N8O	A	-	-	-	-	0.96
6OIG	5	-	-	-	-	0.99

Table 6 of 9

PDB		LSU				
ID	chain	⟨rscc⟩	⟨rsr⟩	⟨rsrz⟩	⟨Q_score⟩	⟨inclusion⟩
6PC5	I	-	-	-	-	0.94 (2568/2576)
6PC6	I	-	-	-	-	0.83 (2570/2578)
6PC7	I	-	-	-	-	0.87 (2571/2579)
6PC8	I	-	-	-	-	0.88 (2551/2559)
6PCH	I	-	-	-	-	0.90 (2557/2565)
6PCQ	I	-	-	-	-	0.86 (2559/2567)
6PCR	I	-	-	-	-	0.96 (2566/2574)
6PCS	I	-	-	-	-	0.94 (2575/2583)
6PCT	I	-	-	-	-	0.95 (2553/2561)
6PJ6	I	-	-	-	-	0.60 (2660/2670)
6PPF	A	-	-	-	-	0.87
6PPK	A	-	-	-	-	0.96
6PVK	A	-	-	-	-	0.88
6QDW	b	-	-	-	-	1.00
6QIK	A	-	-	-	-	0.94
6QKL	N	-	-	-	-	0.93
6QT0	A	-	-	-	-	0.93
6QTZ	A	-	-	-	-	0.92
6QUL	A	-	-	-	-	0.80
6R84	1	-	-	-	-	0.98
6R86	1	-	-	-	-	0.98
6R87	1	-	-	-	-	0.99
6RI5	A	-	-	-	-	0.94
6RZZ	A	-	-	-	-	0.95
6S05	A	-	-	-	-	0.87
6S0K	A	-	-	-	-	0.99
6S0Z	A	-	-	-	-	0.90
6S12	A	-	-	-	-	0.92
6SJ6	A	-	-	-	-	0.89
6SPB	A	-	-	-	-	0.98
6SPD	A	-	-	-	-	0.73
6SWA	q	-	-	-	-	0.86
6T59	54	-	-	-	-	0.30
6TNN	U	-	-	-	-	0.98
6TPQ	U	-	-	-	-	0.99
6U48	CA	-	-	-	-	0.59 (2703/2713)
6V3D	AN1	-	-	-	-	0.97 (1353/1358)
6W6L	t	-	-	-	-	0.93
6WNT	4	-	-	-	-	0.99
6WQN	1	-	-	-	-	0.99
6WQQ	1	-	-	-	-	0.98
6WRS	1	-	-	-	-	0.98
6WRU	1	-	-	-	-	0.99

Table 7 of 9

PDB		LSU				
ID	chain	⟨rscc⟩	⟨rsr⟩	⟨rsrz⟩	⟨Q_score⟩	⟨inclusion⟩
6WU9	A	-	-	-	-	0.98
6WYV	I	-	-	-	-	0.74 (2532/2540)
6XZ7	A	-	-	-	-	0.97 (2622/2632)
6Y6X	L5	-	-	-	-	0.99
6YHS	1	-	-	-	-	0.98
6YLG	1	-	-	-	-	0.96
6YLH	1	-	-	-	-	0.88
6YLX	1	-	-	-	-	0.86
6YLY	1	-	-	-	-	0.89
6YS3	b	-	-	-	-	1.00
6YSI	1	-	-	-	-	0.97
6YWS	A	-	-	-	-	0.82
6YWV	A	-	-	-	-	0.84
7A0R	X	0.95	0.17	-0.27	-	-
7A0S	X	0.95	0.14	-0.61	-	-
7A18	X	0.91	0.21	0.29	-	-
7A5H	A	-	-	-	-	0.88
7A5J	A	-	-	-	-	0.89
7AIH	1	-	-	-	-	0.72
7ANE	1	-	-	-	-	0.73
7AQC	A	-	-	-	-	0.98
7AQD	A	-	-	-	-	0.99
7AS8	A	-	-	-	-	0.98
7AS9	A	-	-	-	-	0.95
7ASM	A	-	-	-	-	1.00 (1337/1339)
7ASN	A	-	-	-	-	0.96 (1100/1101)
7AZY	E	-	-	-	-	0.70
7BHP	L5	-	-	-	-	1.00
7BL2	A	-	-	-	-	0.78
7BL3	A	-	-	-	-	0.85
7BL4	A	-	-	-	-	0.92
7BL5	A	-	-	-	-	0.84
7BL6	A	-	-	-	-	1.00
7BT6	1	-	-	-	-	0.94
7BTB	1	-	-	-	-	0.91
7BV8	A	-	-	-	-	0.90
7F0D	A	-	-	-	-	0.81
7F5S	L5	-	-	-	-	1.00 (1442/1447)
7L20	A	-	-	-	-	0.96
7LVK	I	-	-	-	-	0.76 (2663/2673)
7M4V	A	-	-	-	-	0.98 (1553/1558)
7NFX	5	-	-	-	-	0.87
7NSH	BA	-	-	-	-	0.96
7O9K	A	-	-	-	-	0.99
7O9M	A	-	-	-	-	0.99
7OBR	5	-	-	-	-	0.94
7ODE	I	-	-	-	-	0.91 (1531/1533)

PDB		LSU				
ID	chain	⟨rscc⟩	⟨rsr⟩	⟨rsrz⟩	⟨Q_score⟩	⟨inclusion⟩
7ODR	A	-	-	-	-	0.98
7ODS	A	-	-	-	-	0.99
7ODT	A	-	-	-	-	0.96
7OF0	A	-	-	-	-	-
7OF1	1	-	-	-	-	0.91
7OF2	A	-	-	-	-	1.00
7OF3	A	-	-	-	-	0.99
7OF4	A	-	-	-	-	1.00
7OF5	A	-	-	-	-	0.99
7OF6	A	-	-	-	-	1.00
7OF7	A	-	-	-	-	1.00
7OH3	1	-	-	-	-	0.89
7OHP	1	-	-	-	-	0.94
7OHQ	1	-	-	-	-	0.92
7OHR	1	-	-	-	-	0.94
7OHS	1	-	-	-	-	0.94
7OHU	1	-	-	-	-	0.96
7OHV	1	-	-	-	-	0.94
7OHW	1	-	-	-	-	0.95
7OHX	1	-	-	-	-	0.97
7OHY	1	-	-	-	-	0.96
7OI6	A	-	-	-	-	0.94
7OI7	A	-	-	-	-	0.90
7OI8	A	-	-	-	-	0.99
7OI9	A	-	-	-	-	0.95
7OIA	A	-	-	-	-	0.98
7OIB	A	-	-	-	-	0.97
7OIC	A	-	-	-	-	0.99
7OID	A	-	-	-	-	0.96
7OIE	A	-	-	-	-	0.97
7OOD	3	-	-	-	-	0.00
7OPE	A	-	-	-	-	-
7OW7	A	-	-	-	0.60 (1374/1375)	0.99 (1371/1375)
7PAT	3	-	-	-	0.17	1.00
7PAU	3	-	-	-	0.18	1.00
7PD3	A	-	-	-	-	0.80
7PKT	7	-	-	-	-	0.53
7PO4	A	-	-	-	-	0.81
7PWG	1	-	-	-	-	0.91 (893/895)
7QH6	A	-	-	-	-	0.98
7QH7	A	-	-	-	-	0.99
7QIW	2	-	-	-	-	0.92 (1341/1344)
7QWQ	5	-	-	-	-	0.88
7QWR	5	-	-	-	-	0.86
7QWS	5	-	-	-	-	0.34
7S0S	C	-	-	-	-	0.86
7S9U	A	-	-	-	-	0.94
7SAE	A	-	-	-	-	0.96
7TM3	K	-	-	-	0.45	0.99
7TOO	A25S	-	-	-	-	1.00
7TOP	A25S	-	-	-	-	0.99
7TTU	1	-	-	-	-	0.92

Table 9 of 9

PDB		LSU				
ID	chain	⟨rscc⟩	⟨rsr⟩	⟨rsrz⟩	⟨Q_score⟩	⟨inclusion⟩
7TTW	1	-	-	-	-	0.98
7TUT	K	-	-	-	0.35	0.99
7Z20	b	-	-	-	-	0.97
7Z34	1	-	-	-	0.36	0.97
7ZOD	b	-	-	-	-	0.93
7ZP8	b	-	-	-	-	0.91
7ZQ5	b	-	-	-	-	0.91
7ZQ6	b	-	-	-	-	0.89
