# Supplementary material for: Ratchet, swivel, tilt and roll: a complete description of subunit rotation in the ribosome
Source: Nucleic Acids Res. 2022 Dec 30;51(2):919–34. doi: 10.1093/nar/gkac1211 (PMC9881166; doi:10.1093/nar/gkac1211)
Supplement: gkac1211_Supplemental_Files [file gkac1211_supplemental_files.zip › SI-revised113022.pdf]

Supporting Information for "Ratchet, swivel, tilt and roll: A complete description of subunit rotation in the ribosome"

Asem Hassan, Sandra Byju, Frederico Campos Freitas, Claude Roc, Nissa Pender, Kien Nguyen, Evelyn Kimbrough, Jacob Mattingly, Ronaldo Junio de Oliveira, Ruben Gonzalez Jr., Christine Dunham, and Paul C. Whitford*

E-mail: p.whitford@northeastern.edu

Supporting Methods

Methodological Overview

The following steps were applied to identify all ribosome structures (LSU-SSU pairs, LSUs and SSUs) that are available in the RCSB repository. All analysis was performed using the command-line interface of RADtool v1.0, which is available for download at http://radtool.org. Below, each step of analysis that is described is also accompanied with the associate flags/options for RADtool. Detailed descriptions of each algorithm are provided in subsequent sections.

1. All RCSB entries that contain at least one RNA chain were considered. This included 6395 entries (queried on 10/30/2022).

2. Of all entries containing RNA, only those that had at least one RNA chain longer than 500 residues in length were considered further. This reduced the list to 1749 entries.

3. All 1749 entries were analyzed with the RAD method. The `download`, `bundle`, `findhead` and `stamp` methods (implemented in RADtool) were used. The `download` option is used to download the structure files (PDB bundles, individual PDBs, or mmCIF files) from RCSB. The `bundle` method automatically identifies all LSU-SSU rRNA pairs (see description in next section) with the given accession code. The `findhead` method identifies the SSU head domain by performing sequence alignment, followed by the application of a contact-based criterion (see below for details). The `stamp` method applies STAMP structure alignment[1] of the reference *E. coli* structures to the model, where alignment is performed separately for the SSU head, SSU body and LSU rRNA. For some accession codes, this approach was unable to automatically identify the LSU-SSU pairs, and analysis had to be performed interactively. Together, this step identified 1208 LSU-SSU pairs in 901 RCSB entries. See Appendix A for listing of all calculated values and analysis notes.

4. For the entries that were not found to contain an LSU-SSU pair, we applied the `bundle`, `findhead`, `stamp` and `SSUonly` methods. In contrast to the previous step, the `SSUonly` method attempts STAMP structure alignment of the reference SSU structure to all chains that contain more than 500 residues. Only 3 entries were analyzed manually. In total, this step identified 334 SSUs in 325 RCSB entries. Of these, two structures are "stapled" ribosomes that contain LSU rRNA. However, since the LSU sequences are permuted, STAMP alignment was unreliable for these two LSU-SSU structures. See Appendix B for listing of all calculated values.

5. For entries that were not identified as containing an LSU-SSU pair, or SSU, we applied the `bundle`, `findhead`, `stamp` and `LSUonly` methods. This is identical to the previous step, except that alignment of the reference LSU rRNA was attempted. This identified 375 LSUs in 369 entries. Two entries had to be analyzed manually. See Appendix C for listing of alignment information.

6. After applying the RAD method, there were an additional 154 RCSB candidate entries (i.e. containing RNA chains longer than 500 residues) for which a ribosome was not identified. Each of these was visualized manually to check for the possibility of false-negatives. Based on visual inspection, 35 entries were found to contain ribosomes. However, these were all atypical structures that are incomplete (e.g. partially assembled), contain fragmented rRNA molecules (e.g. in *Euglena gracilis*), or are associated with more exotic species (e.g. Trypanosomes). For a complete list, with brief explanations, see Appendix D.

The "`bundle`" method

default behavior

The `bundle` method identifies LSU-SSU pairs within a single PDB bundle (or CIF file) using the following series of steps:

- Identify every RNA chain that contains at least 500 residues. If multiple fragments are found to have identical chain IDs, only the longest contiguous set of residues is considered.
- Starting with the largest RNA chain (presumably an LSU rRNA), which we will refer to as chain i, identify the number of contacts formed with all other long RNA molecules. The number of contacts between RNA molecules is defined based on a 10 Å distance cutoff.
- Identify the chain j that has the most contacts with chain i. Assign pair $i - j$ as a candidate LSU-SSU pair.
- After assigning a pair, no longer consider those two chains. Return to step 1 and repeat steps until all chains have been designated as part of a candidate pair.

`bundle` with `SSUonly`

When using the `SSUonly` method, the `bundle` algorithm omits the contact-based analysis, and simply considers all chains with at least 500 residues to be candidate SSUs.

`bundle` with `LSUonly`

When using the `LSUonly` method, the `bundle` algorithm omits the contact-based analysis, and simply considers all chains with at least 500 residues to be candidate LSUs.

The `findhead` protocol

When performing STAMP structure alignment, the algorithm may fail if the two domains are insufficiently similar. To reduce the chance of STAMP failures, the `findhead` protocol was developed to automatically determine which residues should be used to define the SSU head. To do this, the model sequence is aligned to *E. coli* using the ClustalW algorithm.[2] The alignment is then used to determine which residue n in the model most closely aligns

to residue 900 of the *E. coli* structure. Then, the structure of residues $n-200$ to $n+500$ is analyzed, where all contacts between side chains are evaluated. A contact is defined as any two atoms that are within 3 Å. Each contact is defined by atom indices i and j. The contact for which $|i-j|$ is the greatest is used to define the first and last residue of the head. This ensures that the largest loop (closed by sidechain contacts) is used to define the head. If no contacts were found using the 3 Å threshold, then a 24 Å cutoff was used. This longer cutoff was necessary for models that only contain P atoms.

Defining the rotation axis $\vec{R}$ and direction of $\psi = 0$

The direction of the rotation axis is defined such that the tilt difference between reference models is minimal. The following protocol is applied separately for the body and head.

1. Define $\vec{x}_{\rm i}$, $\vec{x}_{\rm j}$ and $\vec{x}_{\rm k}$ as the coordinates of the P atoms of residues $\rm i, j, k$.
2. Calculate the vectors $\Delta\vec{x}_1 = \vec{x}_{\rm k} - \vec{x}_{\rm i}$ and $\Delta\vec{x}_2 = \vec{x}_{\rm j} - \vec{x}_{\rm i}$.
3. $\vec{R}$ is then defined to be parallel to $\Delta\vec{x}_1 \times \Delta\vec{x}_2$. According to this definition, the plane of rotation M intersects the P atoms of residues $\rm i, j, k$
4. $\vec{R}$ is then defined to intersect with the center of rotation, as defined by the two reference models.

This protocol defines the position of $\vec{R}$ for each rigid-body orientation. That is, these axes remain fixed to the rigid bodies. For the body domain $(\rm i, j, k) = (U13, C436, C823)$, while for the head domain $(\rm i, j, k) = (U1007, G1153, U1315)$. Residues are shown in Fig. S10. With these definitions, the tilt differences between the reference models are 0.00489° (body) and 0.0321° (head).

$\vec{R}$ then defines the plane of rotation M. To define the direction that corresponds to the tilt direction $\psi = 0$, the following steps are applied:

- Define the geometric centers of two groups of residues $\vec{x}_{\rm g1}$ and $\vec{x}_{\rm g2}$.

- Calculate the vector $\Delta x_{\mathrm{g}} = \vec{x}_{\mathrm{g2}} - \vec{x}_{\mathrm{g1}}$.
- Project Δx_{g} onto the plane M. The direction of the projection defines $\psi = 0$.

For the body, g1 is defined as residues G1426 and U1474, while g2 is defined as residues C1449 and G1454. For g1, the positions used correspond to the unrotated/untilted reference model. For the head, g1 is defined as residue 693, while g2 is defined as residue 519. For the head, the g2 positions correspond to those found after rigid-body alignment of the body domain. Residues are shown in Fig. S10.

Pruning of core residues

To reduce the structural deviations between core residues in each model and the reference *E. coli* structure, the RAD method applies an iterative alignment process (after STAMP alignment) that identifies a subset of residues for which the RMSD is small. Since this step is similar to the "pruning" protocol implemented in the Matchmaker tool in ChimeraX,[3] we will also refer to this process as pruning. The pruning step identifies all the P atoms within each core that can structurally align with *E. coli* to within 2 Å. After pruning, the mean RMSD values were 1.0, 1.1 and 1.0 (±0.2) Å for the LSU, SSU body and SSU head. The mean number of core residues was 1980 (LSU), 789 (body) and 360 (head). For a complete set of calculated values for all models, see Appendix A. In the current manuscript, the term "core" is used exclusively to refer to the pruned sets of residues in the LSU, SSU body and SSU head.

Since all angle calculations are performed based on the core residues, we also considered various metrics for assessing the quality, or possible issues, with the identified core of each structural model. First, as discussed in the main text, we considered the experimental resolution. For example, when assessing body tilting/rolling in bacterial ribosomes, we repeated the comparative analysis for structures that had a resolution of 5Å or better. Next, we assessed the quality of each structural model, relative to the experimental data.

For cryo-EM reconstructions, we calculated the average value of the atom inclusion (i.e. fraction of each residue that is within the high-density regions of the EM map). Since our angle decomposition approach only considers residues that are identified as being part of a "core," we calculated the average residue inclusion value for each core, while non-core residues (e.g. peripheral regions) were not considered. For cryo-EM structures, we also calculated the average Q-score[4] value for each core. For crystallographic structures, we calculated the average rscc, rsr and rsrz values for the residues in each core. Core residue validation summaries are provided in Appendixes E (LSU-SSU assemblies), F (isolated SSUs) and G (isolated LSUs). While most structures have some of these quantities provided, there were also structures that did not contain validation data for the rRNA chains. For completeness, these structures are also listed in the relevant appendix files. A final consideration was the number of residues identified as being part of the core. While the LSU, SSU body and SSU head of most bacterial ribosomes align very well with the reference *E. coli* structure (RCSB ID: 4V9D), we also find some structures where few residues align. These cases indicate that there are internal deformations in the structural models, and they are often associated with low-resolution data.

Supporting Results

Comparing RAD analysis with earlier methods

There have been several strategies proposed to quantify subunit orientations in the ribosome. In most cases, the specific metrics were inspired by a particular type of rearrangement that was of interest in each study. As discussed in the main text, we approach this topic by constructing a set of coordinates that provides a complete description of any rigid-body orientation (i.e SSU body or head), so that every possible type of domain orientation will manifest in distinguishable values. To place this description within the context of the field, we discuss some of the similarities and differences between RAD analysis and earlier approaches. We also compare with values that have been reported for sets of structures with these methods. In particular, we compare our measures with those of Mohan, Donahue and Noller,[5] Agirrezabala et al.,[6,7] Bock et al.[8] and Whitford et al.[9] While some common features of our approach yield generally consistent values with these methods, there are clear and interesting differences that allow RAD to provide a more comprehensive description.

Comparison with Euler-Rodrigues angle calculations

The most well-known method for describing domain orientations in the ribosome is probably that of Mohan et al.[5] In their study, the orientation of each domain (i.e. SSU head or body) is described in terms of a single rotation. In the Euler-Rodrigues formalism, the difference between any two orientations is described as a rotation about a single axis. Since the direction of the E-R rotation axis is a two-dimensional quantity[i], the E-R angle combined with the rotation axis may be used to describe all three rotational degrees of freedom. Thus, even though the E-R angle is highly degenerate, one may infer the character of the rotation (e.g. tilt and tilt direction) by inspecting the rotation vectors. However, there is no direct method for separating the rotation and tilt, or tilt direction, when using E-R angles. To

[i]It is a vector with three components, though, since it is a unit vector, the direction is two-dimensional.

compare with the results of Mohan et al., we compare their reported E-R angles with RAD angle values (Tabs. S1 and S2). With only a few exceptions, the rotation angles of Mohan and RAD angles are highly correlated (Fig. S5). This agreement is expected, since the selected set of structures exhibited only a small degree of tilting.

Comparison with tensor-based angles

An alternate set of rotation coordinates were proposed by Agirrezabala et al.,[6,7] where angles were calculated based on the directions of principal moments of inertia for various domains (e.g. SSU body, shoulder, head). For each domain, a chosen principal axis is projected onto a pre-defined plane, in order to obtain a relative rotation angle (e.g. head swivel angle). In contrast to the E-R approach, projecting onto a plane necessarily reduces the dimensionality of the description, and the calculated angle does not formally correspond directly a domain rotation. Rather, the angular measure describes changes in the projection of a rotation onto a plane. To address this limit, the authors defined two analogous measures that are sensitive to different forms of rotation (i.e. tilting). In that particular case, the study was motivated by the desire to describe head "closure", where the SSU head moves towards the LSU. In the RAD approach, this direction of tilting would correspond to $\psi_{\text{head}} \sim 180^\circ$. Thus, for systems where head rearrangements only involve the primary rotation (i.e. ϕ_{body} or ϕ_{head} in RAD) and tilting about the mRNA binding track, then their approach should yield qualitatively similar results. While their head swivel metric H_{swiv} is correlated with ϕ_{head} (Fig. S7, Tabs. S3 and S4), there is only a weak correlation between the RAD tilt angle and tensor-based tilt measures.

As a note, Agirrezabala et al. reported nearly identical swivel values (difference of 0.1°) for the structural models of Ratje et al.[10] They also noted that these angles are not consistent with the visible differences between the structures (see SI of Ref. 7). It was suggested that the inability to distinguish between these orientations may stem from the low-resolution of the associated cryo-EM reconstructions, combined with the application of crude modeling

techniques. While these do represent significant limitations of those structural models, RAD is able to provide a clear separation, in terms of the rotation angles (Tab. S4). Since those structures were not considered when developing the RAD approach, this comparison effectively serves as a blind test of our approach. Unlike the tensor-based approach, RAD is able to distinguish between the structures, which highlights the robustness of the RAD protocols to possible issues with the analyzed structural models.

Comparison with simulation-based methods

In contrast to the above methods, the protocol presented by Whitford et al.[9] only considered a single rotation of the body (e.g. ratchet-like) and head (e.g. swivel-like), where there was no metric for describing tilt or linear displacements. The definition of the rotation angles were very similar to Agirrezabala et al.,[6] where a vector associated with the head or body was projected onto a predefined plane. While the use of a one-dimensional projection for the head and body represents clear limitations of the approach by Whitford et al., that study was aimed at describing short-timescale rotation-like fluctuations that were apparent in simulations. For that specific application, this very limited description is probably sufficient, though the coordinates can not be extended to describe other degrees of freedom. For completeness, the angles calculated in that study and those with RAD are shown in Table S5. As expected, since the structural models were associated with minimal tilting, those rotation angles are correlated with RAD values.

The RAD method also shares some characteristics of the method presented by Bock et al.[11] Specifically, in their effort to describe explicit-solvent simulations, they applied a least-squares-based approach for rigid-body approximations, which is qualitatively similar to the rigid-body approximation applied by Whitford et al., Mohan et al., Agirrezabala et al. and RAD. In the study of Bock et al., they used these rigid-body representations to estimate the degree of body rotation, head rotation/swivel, as well as a head-tilt measure. While the body rotation and head swivel measures are defined similarly to the angles ϕ_{head} and

ϕ_{body}, the reported head tilting measure was fundamentally distinct from the RAD head tilting coordinate. In RAD, head tilting is defined with respect to the body. Accordingly, the RAD head tilting measure θ_{head} describes intra-subunit tilting. In contrast, Bock et al. defined their head tilting measure with respect to the LSU. As a result, their measures of body rotation and head tilting are not independent. That is, if the SSU were to move as a perfectly rigid body, the head tilting angle and body rotation angle would be linearly proportional, whereas RAD would report a fixed value for the head tilt. Since these measures were designed to measure different structural features, it is not meaningful to compare the numerical values provided by the two strategies.

Supporting Tables

Table S1: Angles calculated with RAD, compared with the Euler-Rodrigues angles reported by Mohan et al.[5] Since tilting is minimal for these models, there is a good correlation between body rotation angles of Mohan et al. and RAD values. The most notable difference is that body rotation angles are approximately 1° larger in RAD, while head rotation angles are approximately 2° smaller in RAD. These shifts may be attributed to the use of different reference structures. The E-R method defined 4V51 as the origin, while RAD defined 4V9D as the origin. The only structures that yield inconsistent values with the two methods are 4V5O:BA and 4V50:DB,CA, though the RAD values are consistent with visual inspection of those structures (Fig. S6). "ND" indicates that a given tilt direction is not defined, which occurs when the tilt angle is 0.

PDB		E-R		RAD					
ID	chains	Body Rot	Head Rot	ϕ_{body}	θ_{body}	ψ_{body}	ϕ_{head}	θ_{head}	ψ_{head}
4V51	BA,AA	0	0	0.9	0.8	-3.6	-1.1	1.1	-61.2
4V6F	DA,CA	1	1.3	1.7	0.3	-19.1	-0.8	1.2	-24.9
4V6E	BA,AA	5.2	1.4	6.0	1.1	107.9	-0.4	1.5	-60.6
4V7L	DA,CA	-0.5	1.4	0.4	0.6	-16.8	-0.5	1.0	-27.2
4V9D	DA,BA	-1.2	1.5	0.0	0.0	ND	0.0	0.0	ND
4V6D	BA,AA	4.9	2	5.8	1.1	98.0	-0.4	1.9	-31.5
4V50	DB,CA	4.3	2	-1.2	1.2	26.2	2.8	1.0	-93.7
4V8U	BA,AA	-1	2.8	0.4	0.3	-158.4	0.6	1.3	-32.3
4V63	BA,AA	-1.7	3	-0.6	0.7	-36.4	0.8	1.2	-51.0
4V42	BA,AA	-2.3	3.1	-1.5	0.8	22.1	1.1	0.3	-116.2
4V5F	BA,AA	-1.2	3.1	0.6	0.5	-172.2	0.7	1.7	-35.1
4V67	DA,CA	-1.2	3.1	0.1	0.3	-62.8	0.9	1.3	-39.7
4V6E	DA,CA	-1.5	3.2	-0.5	0.3	45.2	1.2	0.1	31.5
4V6N	AB,BA	1.7	3.3	2.0	0.7	43.7	1.4	1.5	-25.2
4V6S	AB,BA	0.6	4	-0.2	1.2	-21.1	2.4	2.3	-3.4
4V50	BB,AA	-2.7	4.3	-1.9	1.3	18.3	2.8	1.0	-95.3
4V7D	AA,BA	9.7	4.8	10.4	2.5	7.5	3.3	3.0	-33.2
4V9D	CA,AA	8.4	4.8	9.3	0.0	ND	2.7	1.7	-33.1
4V8O	BA,AA	8.8	6	9.5	2.5	5.1	3.5	2.2	-32.8
4V6O	BB,AA	2.9	5.9	3.4	0.2	56.6	1.4	3.0	5.4
4V5M	BA,AA	6	6.1	6.8	2.4	35.5	3.8	2.2	-31.5
4V90	BA,AA	7	6.1	7.0	1.5	40.7	3.6	1.4	-37.5
4V9H	BA,AA	6.3	6.5	7.2	1.6	36.2	3.9	1.6	-30.1
4V9O	AA,BA	3.1	6.9	4.1	1.0	31.4	4.5	2.5	-5.4
4V6Q	BB,AA	8.5	6.9	8.5	1.3	34.9	3.8	2.8	-23.3

Table S2: Comparison with Mohan et al. 2014 (continued)

PDB		E-R		RAD					
ID	chains	Body Rot	Head Rot	ϕ_{body}	θ_{body}	ψ_{body}	ϕ_{head}	θ_{head}	ψ_{head}
4V6R	BB,AA	7.7	7	7.7	1.0	53.0	3.0	2.2	23.7
4V6P	BB,AA	5.1	7.2	6.1	0.5	40.5	3.2	3.6	39.9
4V9O	EA,FA	2.1	7.4	2.9	0.9	39.0	5.6	1.4	-0.6
4V9O	CA,DA	2.3	7.6	4.7	0.9	70.7	5.6	1.6	-8.7
4V56	BB,AA	-3.3	7.9	-2.5	1.4	22.7	5.9	1.6	132.1
4V64	BB,AA	-3.3	8	-2.4	1.5	23.4	6.0	1.7	142.5
4V4H	BB,AA	-3.3	8.2	-2.5	1.6	25.0	6.0	2.1	142.8
4V4Q	BB,AA	-3.3	8.2	-2.4	1.6	24.5	6.0	2.1	142.5
4V54	BB,AA	-3.5	8.2	-2.6	1.6	26.1	6.2	2.0	143.1
4V6C	DA,CA	-1.3	8.8	-0.3	0.6	54.7	6.0	2.5	55.5
4V9P	CA,DA	2.4	10.1	4.3	1.3	58.6	7.0	2.3	48.5
4V9P	AA,BA	3.8	10.3	4.6	1.6	80.6	8.1	1.8	105.7
4V6C	BA,AA	5	11.6	5.7	1.3	112.1	9.6	1.7	38.0
4V9P	GA,HA	7.2	12.5	7.9	1.0	44.9	9.2	5.3	171.8
4V9P	EA,FA	-2.6	12.6	-1.8	1.5	19.3	10.9	1.7	-135.9
4V9O	GA,HA	6.2	13.1	6.8	1.7	82.6	10.7	2.6	120.6
4V88	A1,A2	-	16	4.1	2.8	88.4	14.0	8.7	63.4
4V64	DB,CA	-2.3	16.1	-1.3	0.1	-49.7	14.5	0.3	138.0
4V54	DB,CA	-2.5	16.2	-1.3	0.6	-149.5	14.6	0.4	11.0
4V85	BA,AA	6.8	16.3	7.3	2.6	44.1	13.8	3.3	72.2
4V4H	DB,CA	-2.3	16.4	-1.4	0.1	-105.3	14.9	0.1	-104.7
4V4Q	DB,CA	-2.3	16.4	-1.3	0.1	-104.6	14.9	0.0	ND
4V9K	BA,AA	1.3	18.2	2.1	0.1	18.8	16.0	3.9	34.3
4V7B	BA,AA	2.5	18.4	3.3	1.2	44.4	16.9	4.2	4.3
4V9L	BA,AA	1.3	18.5	1.9	0.1	-150.2	16.0	4.2	39.5
4V9M	BA,AA	1.1	18.6	1.8	0.0	ND	16.2	4.0	37.5
4V5N	BA,AA	3	20.7	3.5	1.4	67.4	18.1	4.7	25.1
4V9J	BA,AA	3.4	21.3	4.1	1.1	82.7	19.0	3.8	25.1
4V5O	AA	-	1.7	-	-	-	-0.2	2.5	-47.5
4V5O	BA	-	16.2	-	-	-	1.4	0.4	-26.1

Table S3: Angles calculated with RAD, compared with a tensor-based projection method. Values listed were reported in Agirrezabala et al. (2011).[12] For large rotation angles, the tensor-based method values are approximately half the magnitude of RAD values (Fig. S7), and they are defined with a negative sign. "ND" indicates that a given tilt direction is not defined (i.e. when the tilt angle is 0).

PDB		Tensor-based			RAD		
ID	chains	H_{swiv}	H_{tilt}	H_{opcl}	ϕ_{head}	θ_{head}	ψ_{head}
4V4Q	BB,AA	-2.97	-1.87	0.44	6.0	2.1	142.5
4V4Q	DB,CA	-7.09	-6.4	-4.27	14.9	0.0	ND
4V6C	DA,CA	-2.26	-3.31	-0.02	6.0	2.5	55.5
4V6D	DA,CA	-0.01	-0.25	0.78	2.2	0.3	58.0
4V50	DB,CA	0.0	0.0	0.0	2.8	1.0	-93.7
4V50	BB,AA	-0.02	0.01	0.0	2.8	1.0	-95.3
4V6E	DA,CA	0.71	0.59	1.08	1.2	0.1	31.5
4V4I	w,y	1.7	1.54	2.13	-0.9	0.7	130.0
4V51	BA,AA	2.53	2.06	1.59	-1.1	1.1	-61.2
4V51	DA,CA	2.82	2.23	1.73	-1.6	1.2	-52.0
4V5G	BA,AA	2.19	1.64	1.47	-0.6	1.0	-37.4
4V5G	DA,CA	2.32	1.57	1.66	-0.9	1.1	-19.4
4V69	BB,AA	2.42	1.9	1.34	-0.8	1.0	-54.7
4V6K	AB,BA	2.44	1.96	1.41	-1.0	1.4	-58.5
4V6L	BB,AA	2.13	1.62	1.19	-1.4	1.8	-27.2
1IBM	A	1.8	-0.81	-0.71	1.6	3.5	-19.5
1N34	A	0.78	-2.52	-0.26	2.8	4.0	9.0
1N36	A	0.26	-3.06	-0.13	3.6	4.2	16.7
1IBK	A	1.97	-0.47	-0.92	1.3	3.7	-30.1
1IBL	A	2.35	0.14	0.42	0.3	3.3	-11.3
1N32	A	2.29	0.01	0.43	0.4	3.2	-10.0
1N33	A	1.91	-0.53	0.11	1.0	3.5	-8.0
1J5E	A	1.79	-0.96	-1.12	1.7	3.8	-25.5

Table S4: Angles calculated with RAD, compared with a tensor-based projection method. Values listed were reported in Agirrezabala et al. (2012).[7] In that study, it was noted that the similar Rot values for 4V5N and 4V5M were not consistent with visual inspection of the structures. While the tensor-based projection method could not distinguish between these two orientations, RAD angles show a clear difference in the body rotation angles (3.5 and 6.8°).

PDB		Tensor-based				RAD			
ID	chains	Rot	H_{swiv}	H_{tilt}	H_{opcl}	ϕ_{body}	ϕ_{head}	θ_{head}	ψ_{head}
4V6C	DA,CA	0.8	-2.3	-3.3	0.0	-0.3	6.0	2.5	55.5
4V6D	DA,CA	-0.9	0	-0.3	0.8	-1.6	2.2	0.3	58.0
4V6E	DA,CA	-0.6	0.7	0.6	1.1	-0.5	1.2	0.1	31.5
4V6C	BA,AA	6.4	-4	-4.2	-1.6	5.7	9.6	1.7	38.0
4V6D	BA,AA	5.1	2.2	1.4	1.1	5.8	-0.4	1.9	-31.5
4V6E	BA,AA	5.1	2	1.8	1.1	6.0	-0.4	1.5	-60.6
4V5N	BA,AA	8.6	-7.8	-6.5	-6	3.5	18.1	4.7	25.1
4V5M	BA,AA	8.5	0	-1.5	-0.9	6.8	3.8	2.2	-31.5
4V5F	BA,AA	0.4	1.5	0.3	0.4	0.6	0.7	1.7	-35.1
4V5F	DA,CA	0.7	1.7	0.4	0.7	0.6	0.4	1.7	-26.3
4V9D	DA,BA	-0.3	1.4	1.4	1.8	0.0	0.0	0.0	ND
4V9D	CA,AA	8.3	0.2	-0.4	0	9.3	2.7	1.7	-33.1

Table S5: Angles calculated with RAD, compared with the projection-based method of Whitford et al.[9] Since most of these structures have only a small degree of tilting, there is good agreement between the rotation angles in both methods. However, the projection-based method is limited and it can not separately describe rotation and tilting. "ND" indicates that a given tilt direction is not defined, which occurs when the tilt angle is 0.

PDB		Projected Angles		RAD					
ID	chains	Body Rot	Head Rot	ϕ_{body}	θ_{body}	ψ_{body}	ϕ_{head}	θ_{head}	ψ_{head}
4V9D	DA,BA	0.0	0.0	0.0	0.0	ND	0.0	0.0	ND
4V9D	CA,AA	9.1	3.4	9.3	0.0	ND	2.7	1.7	-33.1
4V4Q	DB,CA	-1.2	15.3	-1.3	0.1	-104.6	14.9	0.0	ND
4V4Q	BB,AA	-2.3	6.5	-2.4	1.6	24.5	6.0	2.1	142.5
4V6C	BA,AA	5.7	10.0	5.7	1.3	112.1	9.6	1.7	38.0
4V6C	DA,CA	-0.2	6.9	-0.3	0.6	54.7	6.0	2.5	55.5
4V6D	BA,AA	5.7	0.1	5.8	1.1	98.0	-0.4	1.9	-31.5
4V6D	DA,CA	-1.6	2.9	-1.6	0.8	36.1	2.2	0.3	58.0
4V6E	BA,AA	6.0	0.0	6.0	1.1	107.9	-0.4	1.5	-60.6
4V6E	DA,CA	-0.4	1.8	-0.5	0.3	45.2	1.2	0.1	31.5
4V49	B0,AA	-1.7	1.3	-1.8	0.9	11.4	0.8	0.6	-175.2
4V4A	B0,AA	-1.2	1.0	-1.3	1.2	4.3	0.4	0.9	-154.4
4V4H	BB,AA	-2.3	6.5	-2.5	1.6	25.0	6.0	2.1	142.8
4V4H	DB,CA	-1.3	15.3	-1.4	0.1	-105.3	14.9	0.1	-104.7
4V54	BB,AA	-2.4	6.6	-2.6	1.6	26.1	6.2	2.0	143.1
4V54	DB,CA	-1.2	15	-1.3	0.6	-149.5	14.6	0.4	11.0
4V64	BB,AA	-2.3	6.5	-2.4	1.5	23.4	6.0	1.7	142.5
4V64	DB,CA	-1.2	14.9	-1.3	0.1	-49.7	14.5	0.3	138.0
4V50	BB,AA	-1.7	3.1	-1.9	1.3	18.3	2.8	1.0	-95.3
4V51	BA,AA	0.8	-1.3	0.9	0.8	-3.6	-1.1	1.1	-61.2
4V5L	BA,AA	0.8	-1.0	0.8	0.8	-2.4	-0.9	1.1	-31.2
4V5M	BA,AA	6.6	4.3	6.8	2.4	35.5	3.8	2.2	-31.5
4V5N	BA,AA	3.3	18.7	3.5	1.4	67.4	18.1	4.7	25.1
4V5P	BA,AA	0.0	0.7	0.2	0.2	-45.1	0.3	0.7	-30.6
4V6F	AA,BA	0.2	0.0	0.3	0.7	-20.4	-0.3	0.5	-31.5
4V7P	BA,AA	-0.8	1.3	-0.6	0.5	-43.3	0.7	1.6	-51.2

Supporting Figures

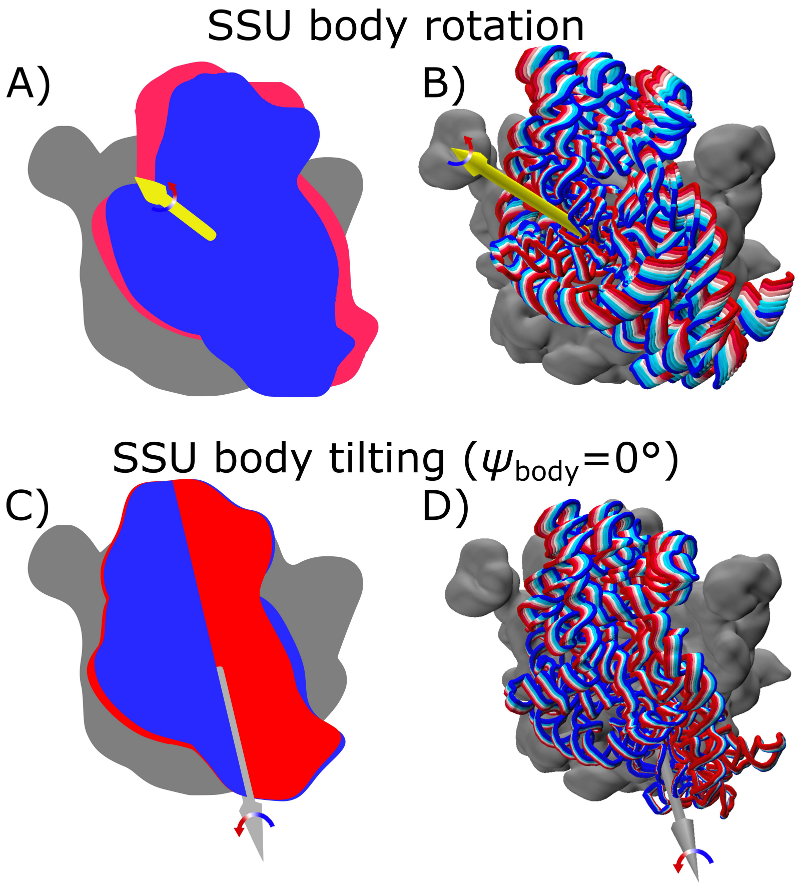

Figure S1: SSU body rotation and tilting/rolling in the ribosome. A) Schematic representation of the SSU body in unrotated (blue) and rotated (red) orientations. LSU is shown in gray. The axis of rotation is shown as a yellow arrow. B) Structural depiction of SSU rotation, with unrotated (blue) and rotated (red) configurations shown, along with intermediate rotation angles (linearly spaced along $\phi_{\rm body}$). C) Schematic representation of the SSU body in untilted (blue) and tilted (red) orientations. LSU is shown in gray. The axis of tilting/rolling is shown as a gray arrow. The tilting axis is in the direction $\psi_{\rm body} = 0$. D) Structural depiction of SSU tilting/rolling, with untilted (blue) and tilted (red) orientations shown, along with intermediate tilt angles (linearly spaced along $\theta_{\rm body}$).

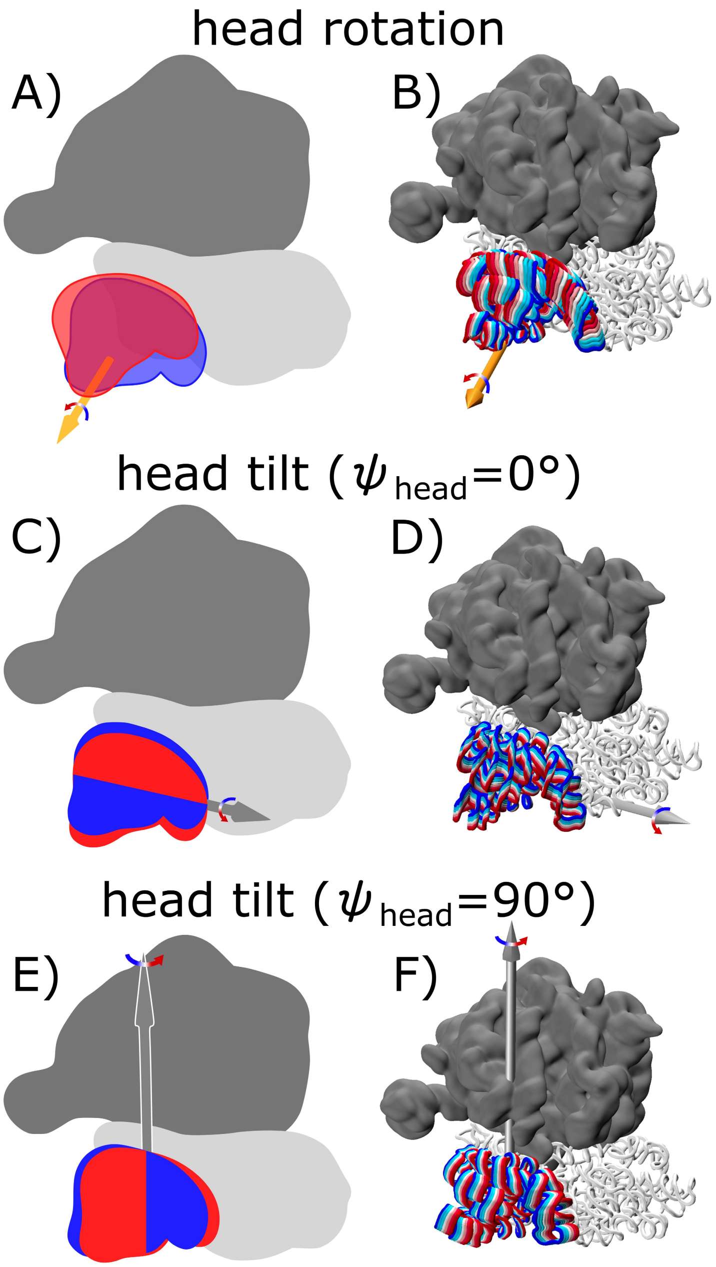

Figure S2: SSU head rotation and tilting/rolling in the ribosome. A) Schematic representation of the SSU head in unrotated (blue) and rotated (red) orientations. LSU is shown in gray and SSU body in light gray. The axis of rotation is shown as an orange arrow. B) Structural depiction of SSU head rotation, with unrotated (blue) and rotated (red) configurations shown, along with intermediate rotation angles (linearly spaced along ϕ_{head}). C) Schematic representation of the SSU head in untilted (blue) and tilted (red) orientations. LSU is shown in gray and SSU body in light gray. The axis of tilting/rolling is shown as a gray arrow. The tilting axis is in the direction $\psi_{\text{head}} = 0$. D) Structural depiction of SSU head tilting/rolling, with untilted (blue) and tilted (red) orientations shown, along with intermediate tilt angles (linearly spaced along θ_{head}). E) Same as panel C, shown for tilting direction $\psi_{\text{head}} = 90$. F) Same as panel D, shown for tilting direction $\psi_{\text{head}} = 90$.

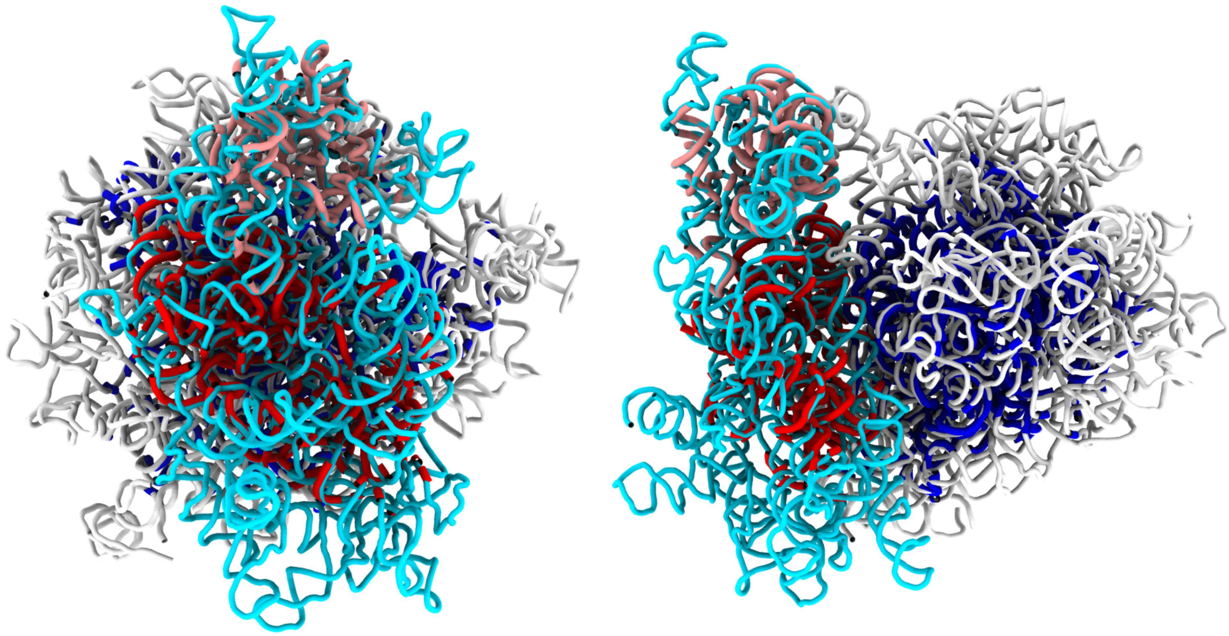

Figure S3: rRNA of a yeast ribosome structure (RCSB ID: 3J77), with SSU in cyan and LSU in white. Structurally-conserved cores shown in blue (LSU), red (SSU body) and pink (SSU head). The RAD method identified 1226, 524 and 236 residues in the cores of the LSU, SSU body and SSU head. Shown cores are the aligned regions of the *E. coli* reference model. Analysis and visualization generated with the RADtool plugin for VMD. VMD[13] was used to generate all structural representations.

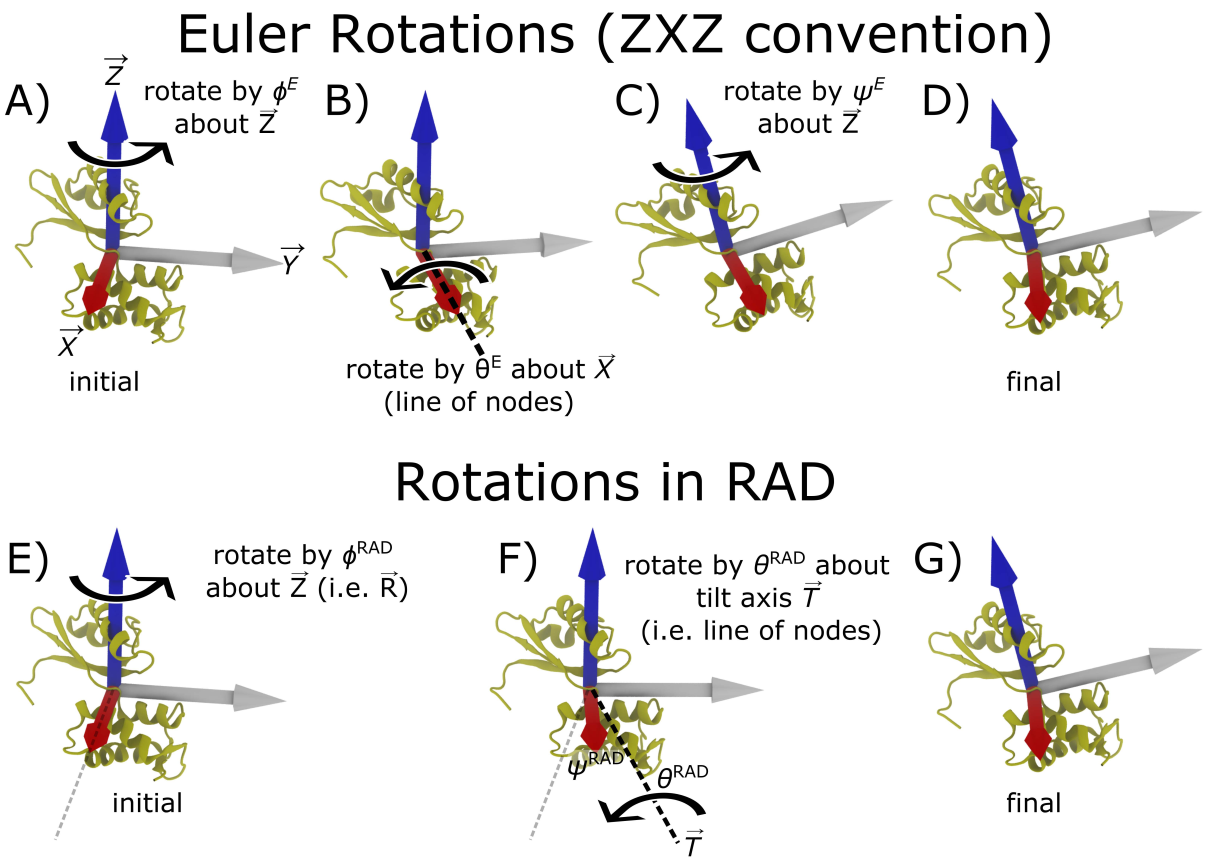

Figure S4: Relationship between standard Euler angles and RAD angles. In both Euler angles and RAD angles, a set of axes is affixed to each rigid body. That is, the $X-Y-Z$ coordinate system is internal to each rigid body. For this comparison, Euler angles will be denoted with the symbols ϕ^{E}, θ^{E} and ψ^{E}, whereas RAD angles are labeled ϕ^{RAD}, θ^{RAD} and ψ^{RAD}. When using Euler angles to describe the orientation of a rigid body, one considers A) rotation of magnitude ϕ^{E} about the axis $\vec{Z}$, B) rotation of magnitude θ^{E} about the rotated direction of $\vec{X}$, and the C) an additional rotation of magnitude ψ^{E} about $\vec{Z}$ (where $\vec{Z}$ is rotated from its initial direction). D) Final orientation after all three Euler rotations are applied. To simplify this representation, RAD angles describe E) a single rotation of magnitude $\phi^{\mathrm{RAD}} = \phi^{\mathrm{E}} + \psi^{\mathrm{E}}$ about $\vec{Z}$, followed by F) a secondary rotation (i.e. tilt) of magnitude $\theta^{\mathrm{RAD}} = \theta^{\mathrm{E}}$ about the tilt axis $\vec{T}$, which forms an angle of $\psi^{\mathrm{RAD}} = \phi^{\mathrm{E}}$ with the initial direction of $\vec{X}$. $\vec{T}$ is in the plane perpendicular to the initial direction of $\vec{Z}$. When applying RAD angles to the body or head domain, $\vec{Z}$ is defined as $\vec{R}_{\mathrm{body}}$ or $\vec{R}_{\mathrm{head}}$, while the initial orientation is defined by the reference model and the final orientation is defined by the structural model of interest. G) Final orientation after both RAD rotations are applied. Orientations shown in panels D and G are identical.

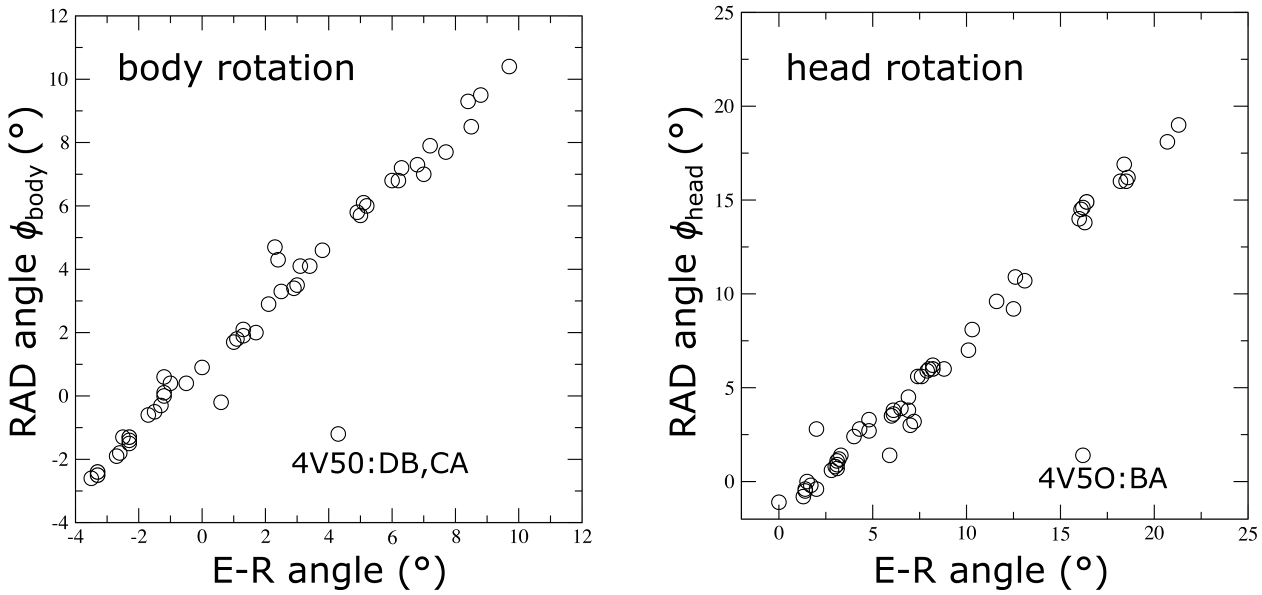

Figure S5: Comparison between E-R angles reported in Mohan et al.[5] and RAD angles. There is excellent agreement, except for a couple of structures, which are shown in Fig. S6.

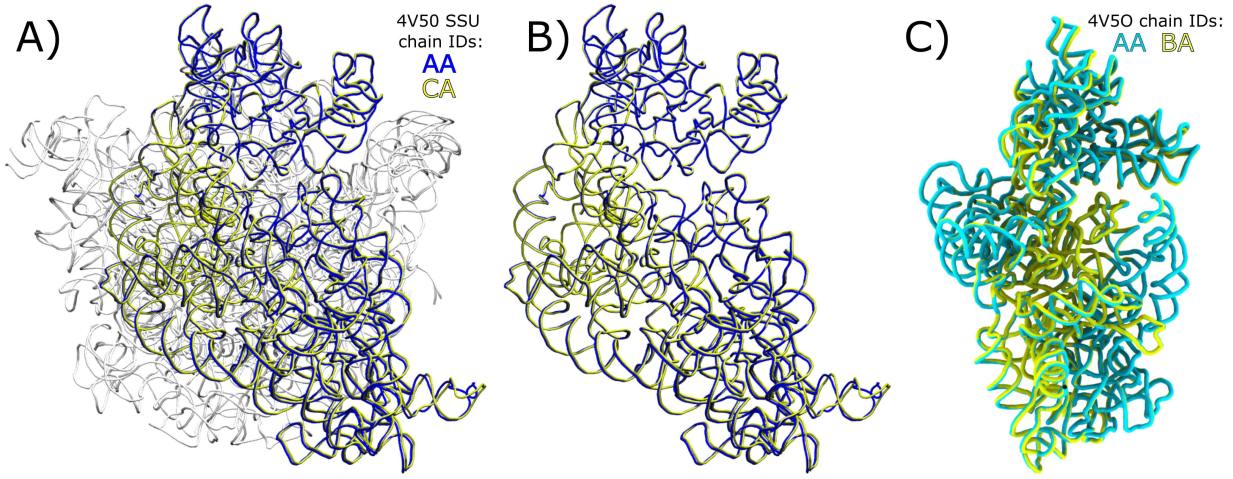

Figure S6: Structures for which there is disagreement between Euler-Rodrigues angles of Mohan et al.[5] and RAD angles. A) Structures of Berk et al. (RCSB ID: 4V50), shown after alignment using RAD. LSU chains are both shown in white. SSU chains are shown in blue (AA) and yellow (CA). For these structures, Mohan et al. reported body rotation angles of 4.3° and -2.7°, though the body orientations are very similar. RAD body angles were -1.2 and -1.9°. For the head, E-R angles reported were 2.0° and 4.3°, whereas RAD yielded a head rotation angle of 2.8° for both models. B) Same as panel A with LSU not shown. C) SSU rRNA for a ribosome from Tetrahymena thermophila (RCSB ID: 4V5O) with chain AA in cyan and chain BA in yellow, after alignment with RAD. While the two configurations are very similar, Mohan et al. reported head rotation angles of 1.7° and 16.2°, whereas RAD head rotation angles were -0.2° and 1.4°.

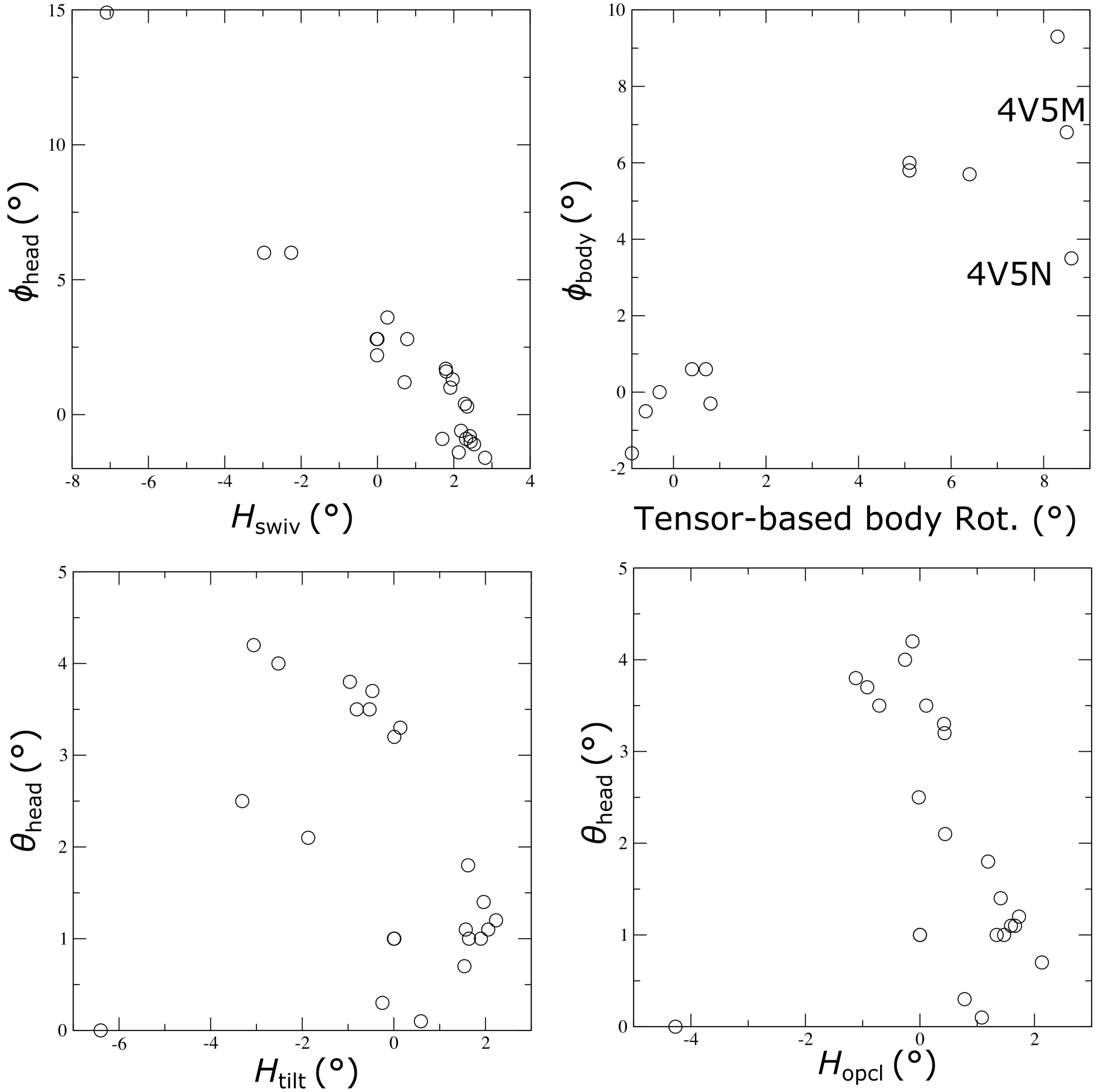

Figure S7: Comparison between tensor-based angles reported in Agirrezabala et al.[12] and RAD angles. H_{swiv} and ϕ_{head} are correlated, though with a slope of approximately -0.5. The body rotation angles exhibit a good correlation. θ_{head} is poorly correlated with H_{tilt} and H_{opcl}.

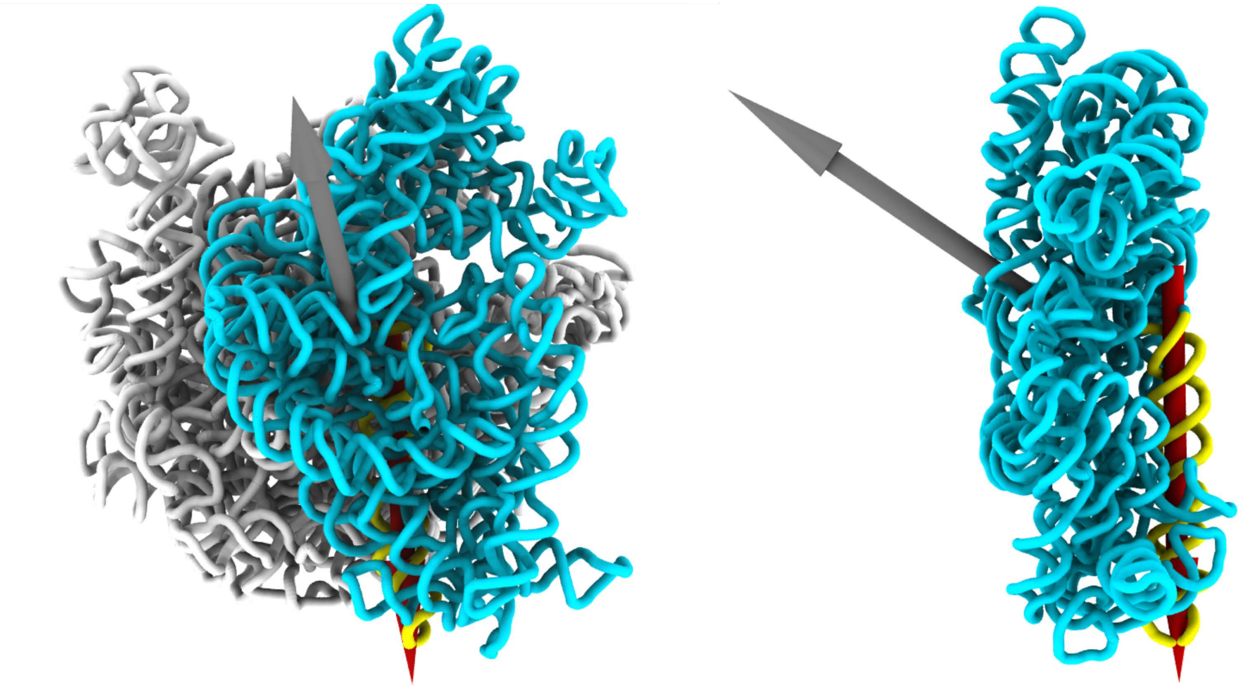

Figure S8: Body rotation axis (gray arrow) and the first principal axis of h44. Since RAD defines tilting as rotation about a secondary, orthogonal axis, it is not possible to define tilting in terms of rotation about h44. The reference *E. coli* structure is shown.

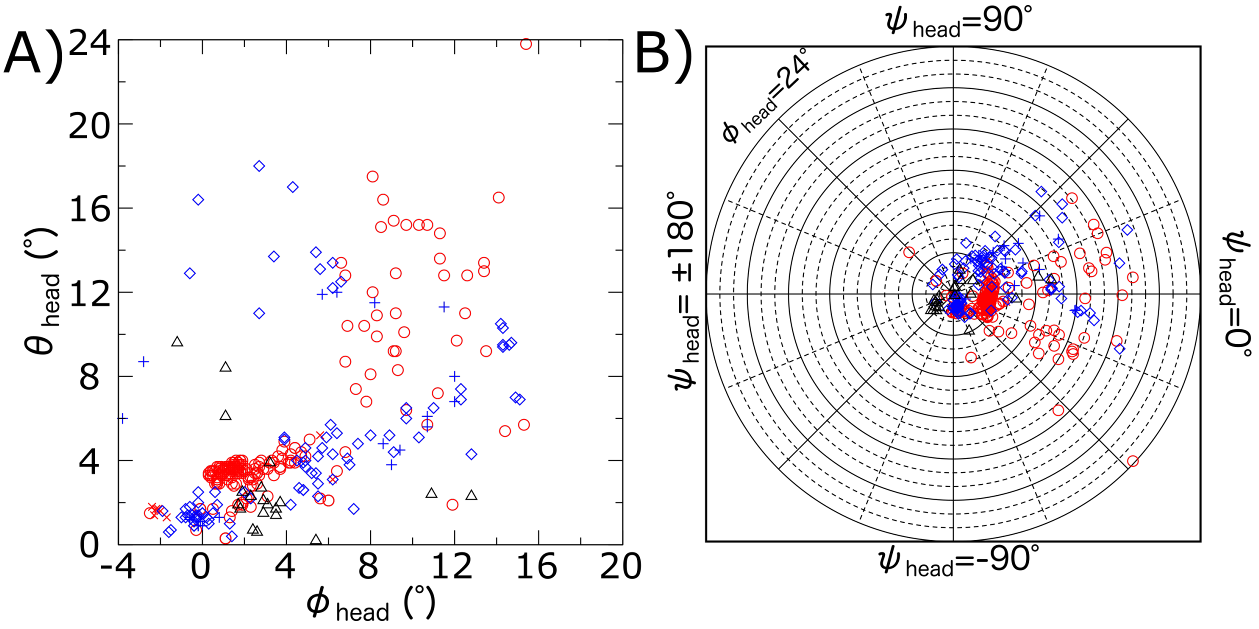

Figure S9: Analysis of isolated SSUs. A) Distribution of head rotation angles ϕ_{head} and tilting angles θ_{head}. B) Distribution of head tilting θ_{head} and tilt direction ψ_{head}. Relative to the analysis of LSU-SSU assemblies (Fig. 6), the scale of tilting and the range of tilt directions is larger in isolated SSUs. This is primarily due to the presence of late assembly intermediates in the dataset.

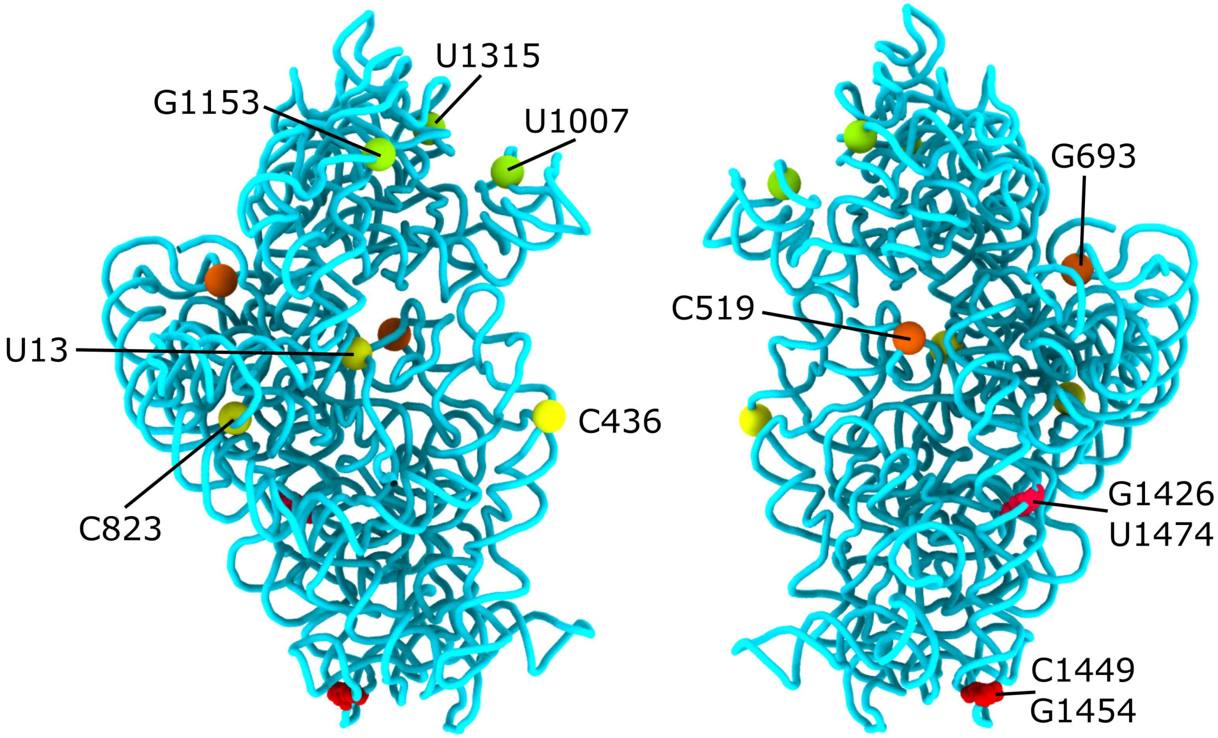

Figure S10: rRNA of the SSU shown with various residues highlighted. Residues shown were used to define rotation axes and the directions of $\psi = 0$ for the body and head. The reference *E. coli* structure is shown. U13, C436 and C823 (yellow) were used to define the rotation plane (normal to the rotation axis) for the SSU body. G1426, C1449, G1454 and U1474 (red) were used to define the direction of $\psi_{\text{body}} = 0$. For the SSU head, U1007, G1153 and U1315 (green) defined the plane of rotation, while C519 and G693 (orange) were used to define the direction of $\psi_{\text{head}} = 0$.

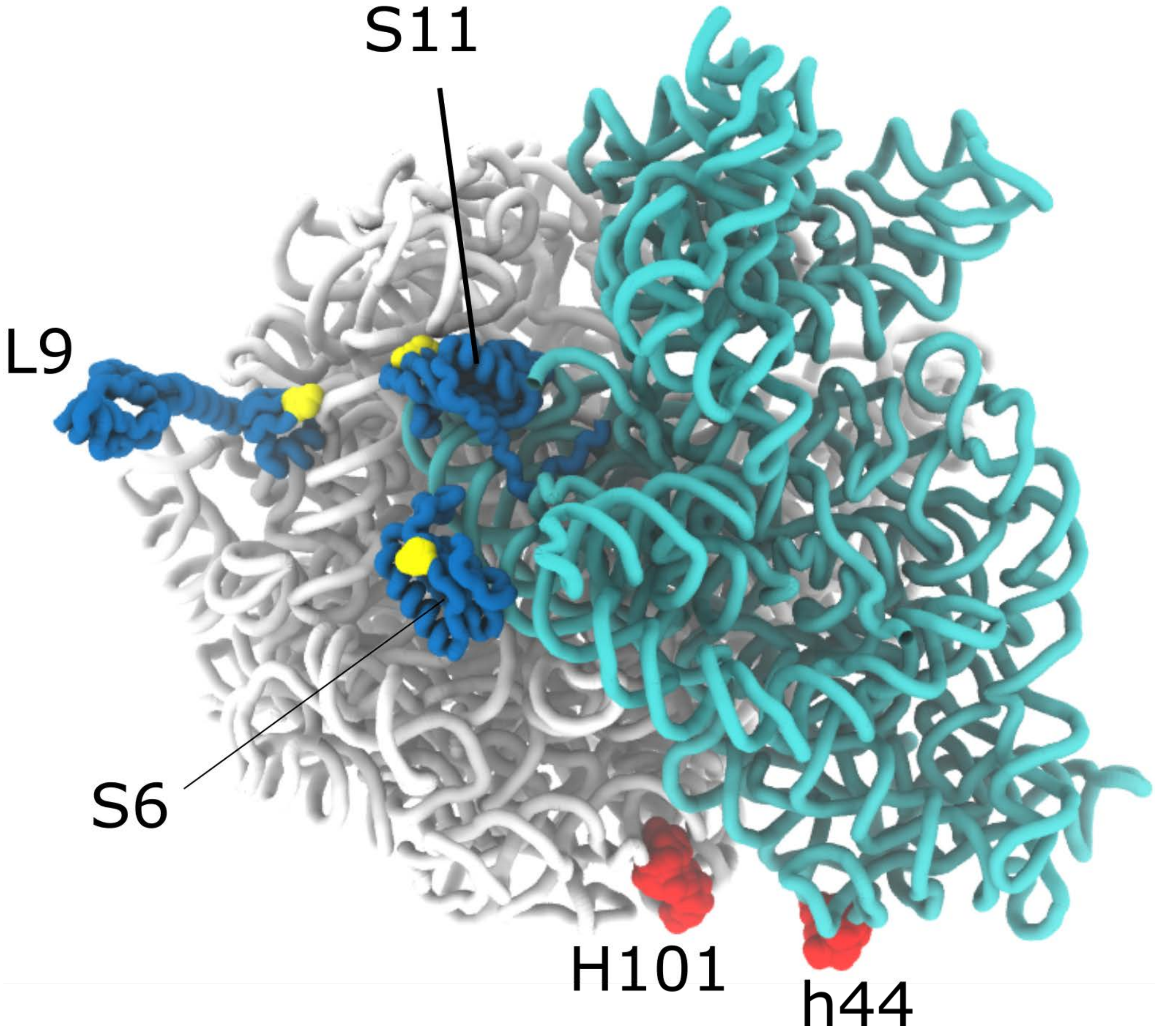

Figure S11: Labeling sites used in experiments to measure SSU rotation. Cornish et al.[14] labeled ribosomal proteins L9 (residue 11), S6 (residue 41) and S11 (residue 75). Proteins are shown in blue. Labeled residues are shown as yellow spheres. Marshall et al.[15] mutated/extended the rRNA helices H101 and h44 (red), in order to measure their relative positions.

References

(1) Russell, R. B.; Barton, G. J. Multiple protein sequence alignment from tertiary structure comparison. *Proteins* **1992**, *14*, 309–323.

(2) Thompson, J. D.; Higgins, D. G.; Gibson, T. J. CLUSTAL W: improving the sensitivity of progressive multiple sequence alignment through sequence weighting, position-specific gap penalties and weight matrix choice. *Nucleic Acid Res.* **1994**, *22*, 4673–4680.

(3) Pettersen, E. F.; Goddard, T. D.; Huang, C. C.; Meng, E. C.; Couch, G. S.; Croll, T. I.; Morris, J. H.; Ferrin, T. E. UCSF ChimeraX: Structure visualization for researchers, educators, and developers. *Protein Science* **2021**, *30*, 70–82.

(4) Pintilie, G.; Zhang, K.; Su, Z.; Li, S.; Schmid, M. F.; Chiu, W. Measurement of atom resolvability in cryo-EM maps with Q-scores. *Nature Methods* **2020**, *17*, 328–334.

(5) Mohan, S.; Donohue, J. P.; Noller, H. F. Molecular mechanics of 30S subunit head rotation. *Proc. Natl. Acad. Sci. USA* **2014**, *111*, 13325–13330.

(6) Agirrezabala, X.; Schreiner, E.; Trabuco, L. G.; Lei, J.; Ortiz-Meoz, R. F.; Schulten, K.; Green, R.; Frank, J. Structural insights into cognate versus near-cognate discrimination during decoding. *The EMBO Journal* **2011**, *30*, 1497–1507.

(7) Agirrezabala, X.; Liao, H. Y.; Schreiner, E.; Fu, J.; Ortiz-Meoz, R. F.; Schulten, K.; Green, R.; Frank, J. Structural characterization of mRNA-tRNA translocation intermediates. *Proceedings of the National Academy of Sciences* **2012**, *109*, 6094–6099.

(8) Bock, L. V.; Blau, C.; Schröder, G. F.; Davydov, I. I.; Fischer, N.; Stark, H.; Rodnina, M. V.; Vaiana, A. C.; Grubmüller, H. Energy barriers and driving forces in tRNA translocation through the ribosome. 2013.

(9) Whitford, P. C.; Blanchard, S. C.; Cate, J. H. D.; Sanbonmatsu, K. Y. Connecting

the Kinetics and Energy Landscape of tRNA Translocation on the Ribosome. *PLoS Comput. Biol.* **2013**, *9*, e1003003.

(10) Ratje, A.; Loerke, J.; Mikolajka, A.; Brünner, M.; Hildebrand, P. W.; Starosta, A.; Dönhöfer, A.; Connell, S.; PFucini,; Mielke, T. et al. Head swivel on the ribosome facilitates translocation by means of intra-subunit tRNA hybrid sites. *Nature* **2010**, *468*, 713–716.

(11) Bock, L. V.; Blau, C.; Schröder, G. F.; Davydov, I. I.; Fischer, N.; Stark, H.; Rodnina, M. V.; Vaiana, A. C.; Grubmüller, H. Energy barriers and driving forces in tRNA translocation through the ribosome. *Nat. Struct. Mol. Biol.* **2013**, *20*, 1390–1396.

(12) Agirrezabala, X.; Schreiner, E.; Trabuco, L. G.; Lei, J.; Ortiz-Meoz, R.; Schulten, K.; Green, R.; Frank, J. Structural insights into cognate versus near-cognate discrimination during decoding. *EMBO J* **2011**,

(13) Humphrey, W.; Dalke, A.; Schulten, K. VMD: Visual molecular dynamics. *J. Mol. Graph.* **1996**, *14*, 33–38.

(14) Cornish, P. V.; Ermolenko, D. N.; Noller, H. F.; Ha, T. Spontaneous intersubunit rotation in single ribosomes. *Mol. Cell* **2008**, *30*, 578–88.

(15) Marshall, R.; Dorywalska, M.; Puglisi, J. Irreversible chemical steps control intersubunit dynamics during translation. *Proc Nat Acad Sci USA* **2008**, *105*, 15364–9.
