# Supplementary material for: Ratchet, swivel, tilt and roll: a complete description of subunit rotation in the ribosome
Source: Nucleic Acids Res. 2022 Dec 30;51(2):919–34. doi: 10.1093/nar/gkac1211 (PMC9881166; doi:10.1093/nar/gkac1211)
Supplement: gkac1211_Supplemental_Files [file gkac1211_supplemental_files.zip › Supplementary legends.pdf]

Supplementary legends

SI.pdf : Supplementary Methods and Results
MovieS1.mov : visual depiction of head rotation, tilting and translation
AppendixA.pdf : Tabulated list of all calculated angles and translations for all 1208 LSU-SSU assemblies.
AppendixB.pdf : Tabulated list of all calculated angles and translations for 334 structures of isolated SSUs.
AppendixC.pdf : Tabulated list of alignment data for 375 isolated LSUs.
AppendixD.pdf : List of 35 ribosome and pre-ribosome structures that were not analyzed.
AppendixE.pdf : Tabulated summary of experimental validation data for all LSU-SSU assemblies.
AppendixF.pdf : Tabulated summary of experimental validation data for all isolate SSUs.
AppendixG.pdf : Tabulated summary of experimental validation data for all isolated LSUs.
Appendix A, B and C contain information about organisms, experimental methods and resolution, original references and an indication of which structures are from mitochondria.
